# Comparative Genomics Reveals the Origins and Diversity of Arthropod Immune Systems

**DOI:** 10.1093/molbev/msv093

**Published:** 2015-04-22

**Authors:** William J. Palmer, Francis M. Jiggins

**Affiliations:** ^1^Department of Genetics, University of Cambridge, Cambridge, United Kingdom

**Keywords:** innate immunity, evolution, genomics

## Abstract

Insects are an important model for the study of innate immune systems, but remarkably little is known about the immune system of other arthropod groups despite their importance as disease vectors, pests, and components of biological diversity. Using comparative genomics, we have characterized the immune system of all the major groups of arthropods beyond insects for the first time—studying five chelicerates, a myriapod, and a crustacean. We found clear traces of an ancient origin of innate immunity, with some arthropods having Toll-like receptors and C3-complement factors that are more closely related in sequence or structure to vertebrates than other arthropods. Across the arthropods some components of the immune system, such as the Toll signaling pathway, are highly conserved. However, there is also remarkable diversity. The chelicerates apparently lack the Imd signaling pathway and beta-1,3 glucan binding proteins—a key class of pathogen recognition receptors. Many genes have large copy number variation across species, and this may sometimes be accompanied by changes in function. For example, we find that peptidoglycan recognition proteins have frequently lost their catalytic activity and switch between secreted and intracellular forms. We also find that there has been widespread and extensive duplication of the cellular immune receptor Dscam (Down syndrome cell adhesion molecule), which may be an alternative way to generate the high diversity produced by alternative splicing in insects. In the antiviral short interfering RNAi pathway Argonaute 2 evolves rapidly and is frequently duplicated, with a highly variable copy number. Our results provide a detailed analysis of the immune systems of several important groups of animals for the first time and lay the foundations for functional work on these groups.

## Introduction

All animals must defend themselves against a battery of natural enemies, ranging from pathogens such as viruses, bacteria and fungi, to macroscopic parasites such as parasitic worms or insects. The immune defenses that have evolved in response to this challenge must distinguish self from nonself, and produce effectors that target and kill these invaders. All the major groups of animals possess an innate immune system, where immune receptors are genetically hard-coded and the response is typically relatively nonspecific with respect to individual pathogen strains or previous exposure (Hoffmann et al. 1999; Kimbrell and Beutler 2001). The innate immune system originated early in animal evolution before the split between protostomes and deuterostomes, as some components of the vertebrate innate immune system show clear homology to insect immune molecules (Hoffmann and Reichhart 2002; Zhu et al. 2005; Wang, Tan, et al. 2006). In addition to an innate immune system, vertebrates possess an adaptive or acquired immune system, where receptor diversity is generated somatically and there is immunological memory (Hoffmann et al. 1999; Kimbrell and Beutler 2001; Hoffmann and Reichhart 2002; Janeway and Medzhitov 2002; Smith et al. 2011). Although key components of the adaptive immune system are not found much beyond vertebrates (Söderhäll 2011), some invertebrates have independently evolved both immune memory and mechanisms to generate receptor diversity somatically (Zhang et al. 2004; Kurtz 2005).

Arthropods have a powerful innate immune response, our understanding of which comes largely from insects, especially *Drosophila* and mosquitoes. In these species, pathogen-associated molecular patterns (PAMPs) (Janeway and Medzhitov 2002) such as bacterial peptidoglycan or fungal beta-1,3 glucan are recognized by pattern recognition receptors such as peptidoglycan recognition proteins (PGRPs) and beta-1,3 glucan recognition proteins (βGRPs) (Kimbrell and Beutler 2001; Lemaitre and Hoffmann 2007; Waterhouse et al. 2007). Following recognition, these receptors then activate the Toll and Imd signaling pathways, leading to the translocation of Nf-κb transcription factors into the nucleus and a humoral response characterized by the expression of antimicrobial peptides (AMPs) (Lemaitre and Hoffmann 2007). In addition, there is a melanization response that kills parasites by depositing the dark pigment melanin along with the production of toxic molecules (Lemaitre and Hoffmann 2007). Alongside the humoral response, there are cellular responses where blood cells called plasmatocytes phagocytose pathogens and specialized flattened cells called lamellocytes can encapsulate larger targets such as parasitoid wasp eggs (Lemaitre and Hoffmann 2007). The main defense against viruses is RNAi, where short RNAs are generated from double-stranded viral RNA and loaded into Argonaut proteins to guide the degradation of viral RNA (Obbard, Gordon, et al. 2009).

Whole-genome analyses have revealed much conservation of key immune pathways and gene families between insect species. The Toll, Imd, JAK/STAT, and JNK signaling pathways are remarkably well conserved, often in 1:1 orthologous relationships between species (Evans et al. 2006; Waterhouse et al. 2007; Zou et al. 2007; Tanaka et al. 2008; Gerardo et al. 2010). A notable exception to this pattern is the pea aphid, which appears to have lost the Imd pathway (Gerardo et al. 2010). Despite this, much variation in presence/absence, copy number, and sequence divergence is observed in other genes, particularly those encoding recognition and effector molecules (Sackton et al. 2007; Waterhouse et al. 2007; Gerardo et al. 2010). For example, mosquitoes show extensive duplications in gene families associated with the response to the malaria parasite *Plasmodium* (Waterhouse et al. 2007).

Beyond the insects, Toll-like receptors (TLRs), their associated signaling components, and Nf-κb transcription factors all have mammalian homologs, suggesting that the origin of these genes predates the protostome/deuterostome split over 600 Ma (Hoffmann et al. 1999; Hoffmann and Reichhart 2002; Janeway and Medzhitov 2002; Lemaitre and Hoffmann 2007). The same is true for the PGRPs and thioester-containing proteins (TEPs), which show similarities to vertebrate alpha-2 macroglobulins and complement factors (Zhu et al. 2005; Sekiguchi et al. 2012). Components of the Imd pathway resemble the tumor necrosis factor receptor pathway of mammals (Hoffmann 2003).

The evolution and diversity of innate immune systems across the arthropods remain poorly understood, despite the importance of arthropods as disease vectors, pests, and components of biodiversity. As yet, the only detailed whole-genome analysis of a noninsect arthropod investigated the crustacean *Daphnia,* which is the sister group to the insects (McTaggart et al. 2009). This found a repertoire of immune genes that is remarkably insect-like, with the notable absence of PGRPs. However, there is a lack of genome-level studies of the more divergent myriapods and chelicerates (Gerardo et al. 2010; Grbić et al. 2011). As such the timing and nature of many key innovations in arthropods remain unresolved, and we lack an overview of the immune system in major arthropod groups. The large variation in the copy number of some immunity gene families in insects would suggest that great variation in arthropod immune systems might occur when looking over larger phylogenetic distances.

The recent sequencing of multiple whole arthropod genomes, some of which are unpublished, provides an opportunity to examine the arthropod immunity gene repertoire in a systematic and consistent fashion. In this study, we have used these genomes to characterize the evolution of the innate immune system across all the main arthropod taxa. Our results show both remarkable diversification of the immune response across the arthropods, and unexpected conservation and similarities to mammalian genes.

## Results and Discussion

To investigate the evolution and origins of the arthropod innate immune system, we identified homologs of insect immunity genes in species that diverged early in the evolution of the arthropods. The arthropods are a phylum that contains four extant subphyla, with the chelicerates, the myriapods, and the crustaceans sequentially diverging from the lineage leading to the insects. This allows us to identify which components of the immune system were present in the ancestral arthropod, and which have been gained or lost later in evolution.

The timing of events early in the evolution of the arthropods is highly uncertain, but it is clear that these four major groups all diverged very early in the evolution of animals. The common ancestor of the arthropods existed an estimated 543 Ma, and by 511 Ma all four of the subphyla had formed (Rota-Stabelli et al. 2013; see figures below). In our analysis we included the genomes of the insect *Drosophila melanogaster*, the crustacean *Daphnia pulex* (water flea)*,* the myriapod *Strigamia maritima* (coastal centipede) and five species of chelicerate: *Mesobuthus martensii* (Chinese scorpion) (Cao et al. 2013)*, Parasteatoda tepidariorum* (house spider), *Ixodes scapularis* (deer tick), *Metaseiulus occidentalis* (western orchard predatory mite), and *Tetranychus urticae* (red spider mite) (Grbić et al. 2011).

To identify homologs of immunity genes across these great phylogenetic distances, we combined methods based on sequence similarity with the predicted cellular location of proteins and analyses of domains, motifs, and residues that are known to be essential for the immune function of the encoded proteins (supplementary table S1, Supplementary Material online). These features can often be identified across very distantly related species, which allows us to guard against the inevitable loss of power to detect sequence similarity when looking at distantly related species.

### Arthropod TLRs Are a Dynamically Evolving Gene Family that Includes Relatives of Vertebrate TLRs

TLRs are transmembrane proteins that play a central role in the immune response of insects and vertebrates. They have an extracellular leucine-rich repeat (LRR) region at the N-terminal, and a cytosolic Toll/interleukin-1 receptor (TIR) domain at the C-terminal (Leulier and Lemaitre 2008). In mammals, different TLRs directly recognize a variety of PAMPs. In *Drosophila*, Toll-1 plays key roles in both development and immunity with the immune function relying on Toll-1 binding to an endogenous cytokine rather than directly to pathogen molecules (see below; Leulier and Lemaitre 2008). Several other *Drosophila* TLRs have been suggested to have immune functions, although these are poorly characterized, and it is likely that most have primarily developmental roles (Leulier and Lemaitre 2008).

**Fig. 1.**
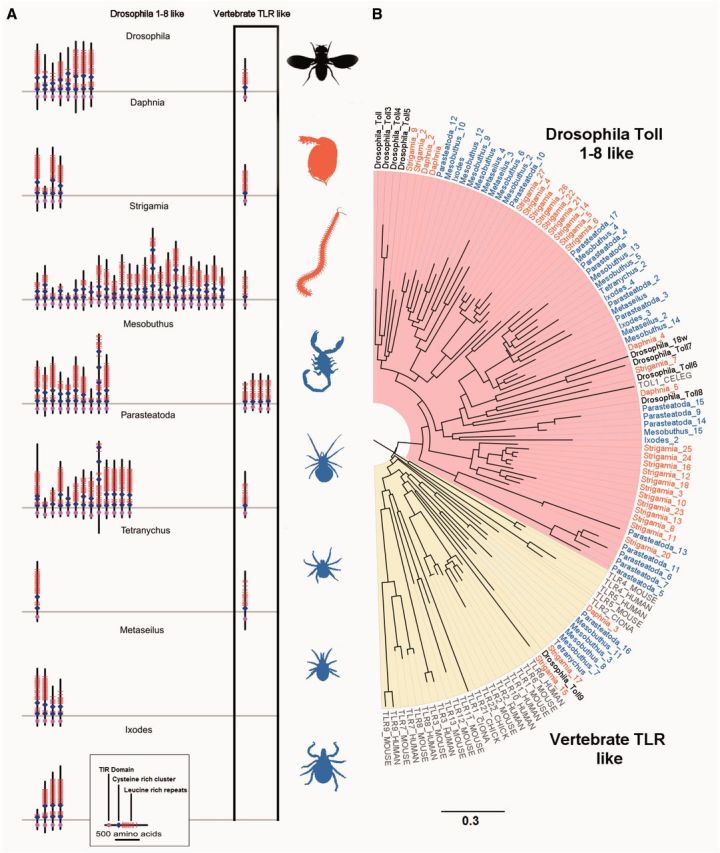
Arthropod TLRs. (*A*) Phylogenetic tree of TLRs from seven species of arthropods, four chordates (human, mouse, chicken, and *Ciona*), and the nematode *Caenorhabditis.* The tree was reconstructed using the maximum-likelihood method from the TIR domains and is midpoint rooted. Myriapod and crustacean taxon labels are orange, chelicerates blue, and *Drosophila* black. Scale bar is substitutions per site. Bootstrap support values can be found in supplementary figure S1, Supplementary Material online. (*B*) The domain structure of arthropod TLRs. TLRs in the yellow vertebrate clade of panel (*A*) are shown in the black box. Red bars are LRRs, blue diamonds are cysteine clusters, magenta the TIR domain, and the gray line represents the plasma membrane. Domain locations are all to scale.

In all the arthropod genomes we find multiple TLRs, with a TIR domain separated from LRRs by a transmembrane helix. On a tree reconstructed from the sequence of the TIR domain, Toll-1 clusters with three other *Drosophila* TLRs (albeit with weak support; fig. 1 and supplementary fig. S1, Supplementary Material online), so there are no clear 1:1 homologs of Toll-1 outside of the insects. Therefore, it cannot be predicted which if any of the TLRs beyond the insects are likely to have a role in immune signaling.

The TLRs have been frequently lost and duplicated during arthropod evolution, as there is extensive variation in copy number and little congruence between the gene and species trees (fig. 1). Within the myriapods there have been two large copy number expansions, resulting in a total of 27 TLRs in the *Strigamia* genome (fig. 1). The spider *Parasteatoda* and scorpion *Mesobuthus* also have a high number of TLRs (16 and 14, respectively), whereas at the other extreme the mite *Tetranychus* has just two (fig. 1, supplementary table S1, Supplementary Material online).

There are two major structural classes of TLRs and both are widespread in arthropods. The single cysteine cluster TLRs (sccTLRs) have a single cysteine cluster at the end of the LRRs adjacent to the cell membrane, whereas the multiple cysteine cluster TLRs (mccTLRs) have multiple cysteine clusters (Imler and Zheng 2004; Leulier and Lemaitre 2008). The vertebrate TLRs are largely sccTLRs, whereas *Drosophila* Toll receptors other than Toll 9 are mccTLRs (Imler and Zheng 2004; Leulier and Lemaitre 2008). The immune function the *Drosophila* sccTLR (Toll 9) is unclear, with overexpression of Toll-9 upregulating AMPs (Ooi et al. 2002), whereas a *Toll-9* knock out did not affect the immune response (Narbonne-Reveau et al. 2011). We found that although mccTLRs are most common in arthropods, vertebrate-like sccTLRs are also widespread (fig. 1).

The division between sccTLRs and mccTLRs is reflected in their evolutionary relationships, with the two structural classes forming two major clades on the TLR phylogeny (fig. 1). In most cases, the arthropod sccTLRs are more closely related to vertebrate sccTLRs than they are to arthropod mccTLRs (fig. 1, yellow shading). The mccTLR clade (fig. 1, pink shading) contains most of the arthropod TLRs and the *Caenorhabditis elegans* TOL1 (nematodes are protostomes like arthropods). This pattern of the structure of the TLRs being reflected in their phylogeny is especially striking as the phylogeny is reconstructed using the sequence of the intracellular TIR domain, whereas the structural classification is based on extracellular sequences. The structural classification thus provides corroboration for this phylogenetic division, despite the tree being poorly resolved (supplementary fig. S1, Supplementary Material online). Therefore, throughout the arthropods there are a small number of TLRs that are more similar to vertebrate TLRs than other arthropod TLRs.

The presence of arthropod TLRs clustering with vertebrates shows that mccTLRs and sccTLRs diverged early in animal evolution, but it is unclear whether the common ancestor of protostomes and deuterostomes had both types of TLR. Our tree places arthropod sccTLRs sequences interspersed among vertebrate TLRs (fig. 1). However, this may simply reflect error in the tree reconstruction, as bootstrap support for these relationships is low (supplementary fig. S1, Supplementary Material online) and we were unable to reject a tree where all the deuterostome and protostome TLRs were monophyletic (Shimodaira–Hasegawa Test: 2Δ*l* = 6, *P* > 0.05). As we cannot reliably root the tree (fig. 1), the most parsimonious interpretation of the data is that the common ancestor had sccTLRs, with mccTLRs appearing later in protostome evolution. We also find a few cases of unusual extracellular domain structures, such as short truncated TLRs in the mccTLR clade that have only have a single cysteine cluster and TLRs with more than two cysteine clusters (fig. 1 and supplementary table S7, Supplementary Material online).

### The Toll Signaling Pathway Is Conserved across Arthropods

The humoral immune response of *Drosophila* and other insects centers on the Toll and Imd pathways, both of which result in Nf-κb transcription factors being activated and translocated into the nucleus, where they upregulate the expression of AMPs and other genes. In *Drosophila,* recognition of Gram-positive bacteria and fungi by βGRPs and short-chain PGRPs causes cleavage of Spätzle, which subsequently binds to and activates Toll-1, initiating the Toll pathway (Bosco-Drayon et al. 2012; Gendrin et al. 2013). Following binding by cleaved Spätzle, the Toll-1 TIR domain initiates a signaling cascade that culminates in the translocation of the Nf-κb transcription factors Dorsal, and Dorsal-related immunity factor (Dif) to the nucleus (Imler and Zheng 2004; Lemaitre and Hoffmann 2007; Leulier and Lemaitre 2008).

**Fig. 2.**
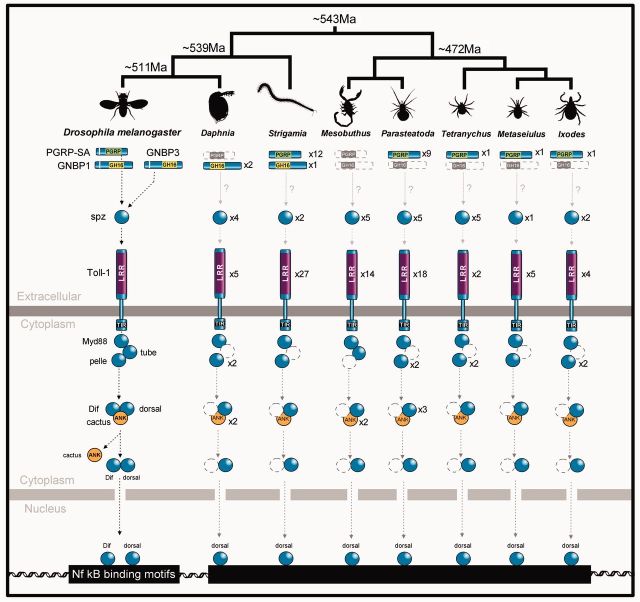
Presence or absence of Toll pathway members across the arthropods. Dashed gray symbols represent proteins where no homolog was detected. Only key domains are shown (Ank: ankyrin repeat). Topology of the phylogeny is taken from Sharma et al. (2014), although it should be noted that relationships within the chelicerata (*Mesobuthus, Parasteatoda, Tetranychus, Metaseiulus,* and *Ixodes*) remain poorly resolved. Divergence dates are in millions of years before present (Ma) and from Rota-Stabelli et al. (2013).

We found Toll pathway members to be highly conserved across the arthropods (fig. 2), with homologs of *Spätzle*, *Myd88*, *pelle*, *cactus*,** and *dorsal* in all species. We failed to find a *Tube* homolog in any of the species studied except *Mesobuthus*, but this is likely to be a lack of power to detect the gene, as a previous analysis of *Tube* suggested that it is homologous to *IRAK-4*, which occupies an equivalent position in the vertebrate Toll pathway (Towb et al. 2009). Indeed we find multiple proteins across species with the IRAK-like death domains that characterize Tube. Most genes are present in single copies, although *Dorsal* (encoding the Nf-κb transcription factor) has been duplicated twice in the spider *Parasteatoda* (as well as being duplicated in *Drosophila*)*,* and *Cactus* (encoding its inhibitor) is duplicated in *Daphnia* and *Mesobuthus.*

### The Imd Signaling Pathway Is Highly Reduced in Chelicerates

In *Drosophila* the systemic humoral immune response to Gram-negative bacteria is controlled by the Imd pathway, which is initiated by the binding of the transmembrane protein PGRP-LC to peptidoglycan. The intracellular RHIM motif of PGRP-LC interacts with Imd, initiating the signaling cascade (Kaneko et al. 2006). Imd in turn activates TAK1, which together with the IkB kinase complex (IKK)b/y complex, Fadd and DREDD (a caspase-8 homolog) activate the Nf-κb transcription factor Relish. Relish is translocated into the nucleus, upregulating AMPs, and other genes. Imd also activates the JNK pathway through Tak1 (Hoffmann 2003; Lemaitre and Hoffmann 2007; Waterhouse et al. 2007). The Imd pathway is especially important in the epithelial immune response, where it regulates genes such as *Drosomycin*, which are controlled by the Toll pathway in the systemic response (Tzou et al. 2000). Furthermore, some components of the pathway also have roles outside the immune response, such as the elimination of mutant or unfit cells (Meyer et al. 2014).

**Fig. 3.**
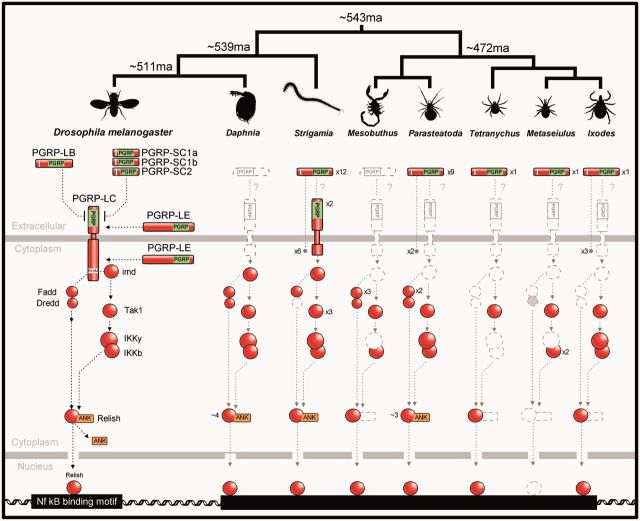
Presence or absence of Imd pathway members across the arthropods. Dashed gray symbols represent genes where no homolog was detected. Only key domains are shown (ANK: ankyrin repeats).

*Relish* is an unusual and easily identifiable gene, as the Nf-κb transcription factor and its inhibitor are combined in a single protein (Wang, Tan, et al. 2006). We find clear Relish homologs in all species but *Metaseiulus*, suggesting that a Relish-based immune response may have been present in the common ancestor of the arthropods (fig. 3). The IKK is required for the cleavage and activation of Relish (Hoffmann 2003; Lemaitre and Hoffmann 2007; Waterhouse et al. 2007), and we find homologs of both the catalytic subunit, IKKb, and the regulatory subunit, IKKg, in most species (fig. 3). The Relish homologs all have an N-terminal relish-like domain that shows closest sequence similarity to *Drosophila* Relish; however, some lack the distinctive C-terminal ankyrin repeat region that plays the role of the Nf-κb inhibitor (fig. 3). In *Daphnia* the ankyrin repeat region is absent from the public gene model due to an error in the automated gene model prediction, as manual annotation identified an additional portion of the gene with ankyrin repeats (McTaggart et al. 2009). It is unclear whether the three chelicerate sequences lacking the ankryin repeats are also annotation errors or reflect true loss of this region.

*Relish* appears to have been lost entirely in the mite *Metaseiulus* (fig. 3)*,* where no homologues could be discerned by sequence similarity or conserved domain searches (RHD-n relish domain). This is especially striking as *Metaseiulus* has a small (Hoy 2009) and well-assembled genome so this is unlikely to be an artifact of an incomplete genome sequence. Overall, this species is missing more Imd pathway components than any of the other species (fig. 3), supporting the hypothesis that this branch of the immune response may have been lost. This would not be a unique occurrence, as Relish and other Imd pathway components have also been independently lost in the pea aphid (an insect) (Gerardo et al. 2010).

Despite the conservation of *Relish* in most species, many other key components of the Imd pathway were only found in the mandibulates and were absent from the chelicerates (fig. 3). In the mites and ticks (*Tetranychus, Metaseiulus*,** and *Ixodes*), we fail to find any likely *Imd*, *Fadd* or *Dredd* homologues. In arachnids (*Parasteatoda* and *Mesobuthus*) we find possible *Fadd* and *Dredd* homologues, although they apparently lack N-terminal DED domains, so they may not have the same function as in *Drosophila.* As Fadd homologues are known to be widespread in the animal kingdom, these results suggests that secondary losses of *Fadd* and *Dredd* may have occurred in the chelicerates.

**Fig. 4.**
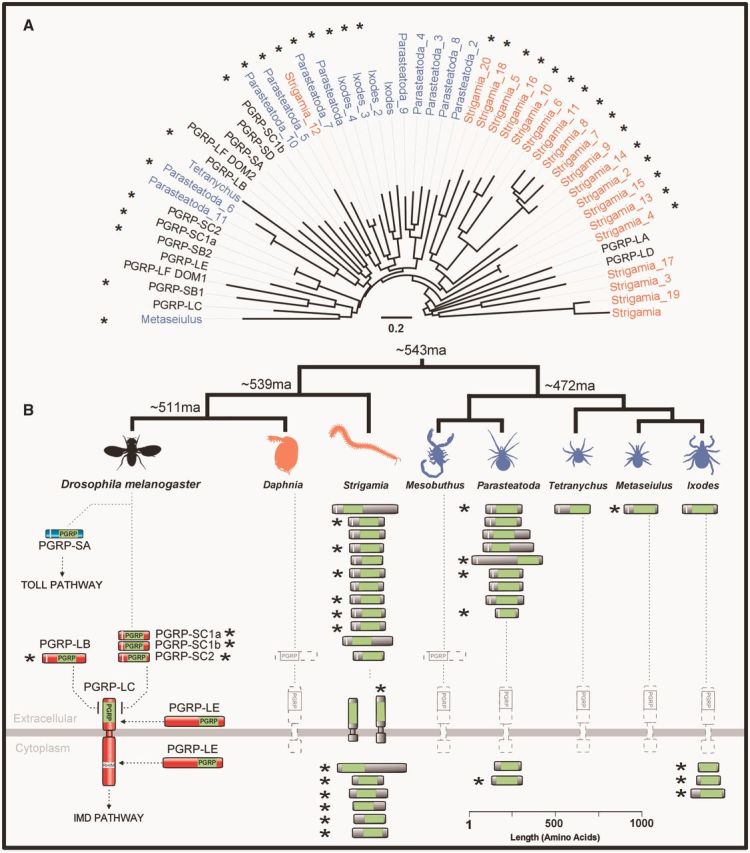
Gene tree, copy number, cellular location, and predicted catalytic activity of arthropod PGRPs. (*A*) PGRP tree was reconstructed using maximum likelihood from the PGRP domain sequences, and is midpoint rooted. Myriapod taxon labels are orange, chelicerates blue, and *Drosophila* black. Scale bar is substitutions per site. Bootstrap support values can be found in supplementary figure S2, Supplementary Material online. (*B*) Scale drawing and predicted cellular location of PGRPs. The PGRP domain is shown in lime green, signal peptides in white. Predicted catalytic PGRPs are denoted by an*.

The absence of an intact Imd pathway in the chelicerates is supported by the distribution of transmembrane PGRPs, which are found at the start of the pathway in *Drosophila.* The PGRP domain can clearly be detected across these phylogenetic distances (see below), and transmembrane proteins can be robustly predicted. Transmembrane PGRPs were entirely absent from the chelicerates (figs. 3 and 4).

Although the Imd pathway is largely intact in both the myriapods and crustaceans, transmembrane PGRPs are not. *Daphnia* does not possess any PGRPs (figs. 3 and 4; McTaggart et al. 2009), suggesting that the Imd pathway is either not functional or is activated in a different way in this species. In the myriapod *Strigamia* there are two transmembrane PGRPs, but we failed to identify the Imd-interacting RHIM domain using HMMER. Furthermore, the transmembrane PGRPs identified in *Strigamia* possess only one external recognition domain—unlike PGRP-LF in *Drosophila* which has two. Therefore, the activation of the Imd pathway by a transmembrane PGRP could either represent an innovation acquired in the insect lineage or it may have been lost in the crustaceans.

In contrast to the absence of many Imd pathway components in chelicerates, the JNK pathway is highly conserved across the arthropods with *Basket* and *Jun* universally present. This may be a consequence of this pathway playing a role in many key cellular processes in addition to its role in Imd-related signaling.

### The JAK/STAT Signaling Pathway Is Highly Conserved

The JAK/STAT pathway plays a role in the immune response of both mammals and *Drosophila.* In *Drosophila* and *Anopheles* mosquitoes, following immune challenge activated STAT translocates to the nucleus where it alters the expression of many genes, including upregulating the *Drosophila* immunity protein TEP1 (Agaisse and Perrimon 2004). We find clear homologues of *Domeless*, *Hop*, and *Stat92e* in most species (table 1). *Parasteatoda* and *Mesobuthus* and *Metaseiulus* lack a clear *Hop* homologue however and *Domeless* would also appear to be absent from *Mesobuthus.*

**Table 1.** Immunity Gene Copy Number in *Drosophila* (Waterhouse et al. 2007; Jang et al. 2008; Pierre et al. 2014) and Seven Other Arthropods as Determined in This Study.

	Drosophila	Daphnia	Strigamia	Metaseiulus	Tetranychus	Ixodes	Mesobuthus	Parasteatoda
Recognition and related								
PGRP	13	0	20	1	1	4	0	11
βGRP	3	2	1	0	0	0	0	0
FREP	13	36	19	1	2	26	25	20
TEP	6	7	4	2	3	3	1	8
DSCAM	3	4	60	4	14	13	12	35
Draper like Nimrods	1	1	1	0	0	1	0	3
Toll pathway								
TLR	9	5	27	5	2	4	14	18
SPZ	6	4	2	1	5	2	5	7
MyD88	1	1	1	1	1	1	1	1
Tube	1	0	0	0	0	0	1	0
Pelle	1	2	1	1	2	2	0	2
Dorsal	1	1	1	1	1	1	1	3
Cactus	1	2	1	1	1	1	2	1
Dif	1	0	0	0	0	0	0	0
Imd pathway								
IMD	1	1	1	0	0	0	0	0
TAK1	1	1	3	1	1	1	1	1
IKKg (Kenny)	1	1	1	0	0	1	0	1
IKKb (Ird5)	1	1	1	2	0	1	1	1
FADD	1	1	1	0	0	0	3	2
DREDD	1	0	0	0	0	0	1	1
Relish	1	4	1	0	1	1	1	3
JAK STAT								
Dome (less)	1	1	2	1	1	2	0	2
Hop	1	1	1	0	1	1	0	0
Stat92	1	1	1	5	2	1	2	2
JNK								
Hemipterous	1	1	1	1	1	1	1	1
Bsk (JNK)	1	2	1	3	1	1	2	2
RNAi								
Dicer 2	1	2	1	5	1	1	0	1
Argonaut 2	1	1	2	1	6	3	6	5
Other								
Clip serine proteases	24	3	4	5	3	4	7	8
Serpins	30	6	11	16	23	44	30	33
PPO-like (predicted tyrosinase activity)	2 (1)	1 (0)	1 (0)	0	0	0	8 (7)	13 (9)
DUOX	1	2	1	1	2	1	2	3
Lysozymes	13	1	5	5	3	4	9	3

Note.—A complete list of immunity genes identified in this study appears in supplementary tables S5 and S6, Supplementary Material online.

### Peptidoglycan-Recognition Proteins

PGRPs bind to bacterial peptidoglycan and can act as pathogen recognition receptors, negative regulators of the immune response, or effectors that kill bacteria (Lemaitre and Hoffmann 2007; Bosco-Drayon et al. 2012; Gendrin et al. 2013). They fall into two main groups. The noncatalytic PGRPs can function as pattern recognition receptors, and play a key role in activating the Toll and Imd pathways of *Drosophila* following infection (PGRP-LC/LE in the Imd pathway and PGRP-SA in the Toll pathway)*.* The catalytic PGRPs possess amidase activity that allows them to enzymatically break down peptidoglycan (in *Drosophila:* PGRP-SC1/2, SB1/2, and LB), and can they function either as negative regulators of the immune response by removing immunogenic peptidoglycan or as effectors that kill bacteria by degrading their peptidoglycan bacteria (Lemaitre and Hoffmann 2007; Bosco-Drayon et al. 2012; Gendrin et al. 2013). PGRPs are also found in mammals, and were therefore presumably present in the common ancestor of the arthropods (Bischoff et al. 2006; Zaidman-Rémy et al. 2006; Lemaitre and Hoffmann 2007; Zaidman-Rémy et al. 2011).

The copy number of PGRPs varies greatly across the arthropods (fig. 4; table 1). They have been entirely lost from both the crustacean *Daphnia* (see also McTaggart et al. 2009) and the chelicerate *Mesobuthus*, whereas *Metaseiulus* and *Tetranychus* each have just a single PGRP. At the other extreme, the myriapod *Strigamia* has 20 PGRPs. All bar one of the *Strigamia* genes cluster together on the gene tree, which suggests that they have probably resulted from one or two ancestral PGRPs that duplicated extensively within the myriapods (fig. 4).

It is possible to predict whether a PGRP has catalytic activity from the sequence of its PGRP domain. The catalytic (amidase) activity of insect PGRPs and bacteriophage T7 lysozyme is zinc-dependant, and the noncatalytic PGRPs have lost residues required for zinc binding (Mellroth et al. 2003; Reiser et al. 2004; Gendrin et al. 2013). To predict the function of the PGRPs, we aligned their PGRP domains and identified a cysteine and two histidine residues (see fig. 3 of Reiser et al. 2004) that are required for zinc binding (Mellroth et al. 2003; Reiser et al. 2004). In support of the functional relationship between these sites, we found that the presence of these three residues was strongly correlated—18 of the 21 sequences with the cysteine also had the two histidines, whereas only one of the 16 sequences without the cysteine had both histidines (supplementary table S2, Supplementary Material online).

Catalytic and noncatalytic PGRPs are scattered across the arthropods, and every species with more than one PGRP has both types (fig. 4, catalytic forms marked*). There have been frequent gains or losses of catalytic activity during evolution, as the two forms are interspersed on the gene tree, and closely related pairs of sequences can differ in whether they have predicted catalytic activity (fig. 4). Together these results suggest that in other arthropods, as in *Drosophila,* PGRPs are likely to play a variety of roles. However, as catalytic or noncatalytic PGRPs are absent from taxa across the tree, it seems likely that these functions may be replaced by other molecules in these groups.

In chelicerates and myriapods, many of the PGRPs do not have a signal peptide and are therefore predicted to be intracellular (fig. 4). In *Drosophila,* intracellular isoforms of PGRP-LE are important in responses to intracellular bacteria (Kaneko et al. 2006), and regulating gut immune responses to both pathogens and the microbiota (Bosco-Drayon et al. 2012). It is unlikely the intracellular PGRPs that we identified perform similar roles, as they differ from PGRP-LE in that they are predicted to be catalytic and do not contain a predicted RHIM motif that could interact with Imd. Therefore, these intracellular catalytic PGRPs may have a novel function such as killing intracellular bacteria.

### βGRPs Have Been Lost from Chelicerates

βGRPs, which are also known as Gram-negative binding proteins (GNBPs), bind β-1,3 glucan in microbial cell walls (particularly fungi), and can act as coreceptors with PGRP-SA to recognize Gram-positive bacteria and initiate the *Drosophila* Toll pathway through Spätzle. Similar to pattern recognition receptor PGRPs, βGRPs originate from enzymes that degrade glucan (β-1,3-glucanases) that have lost their enzymatic activity (Hughes 2012). Many insects have both β-1,3-glucanases and βGRPs, although the *Drosophila* genome encodes only βGRPs (Hughes 2012). We identified β-1,3-glucanases and βGRPs using the diagnostic *O*-Glycosyl hydrolase 16 domain (GH16-superfamily) that they share. Glucanases can be separated by the presence of two amino acid residues that are essential for catalytic activity (Hughes 2012).

**Fig. 5.**
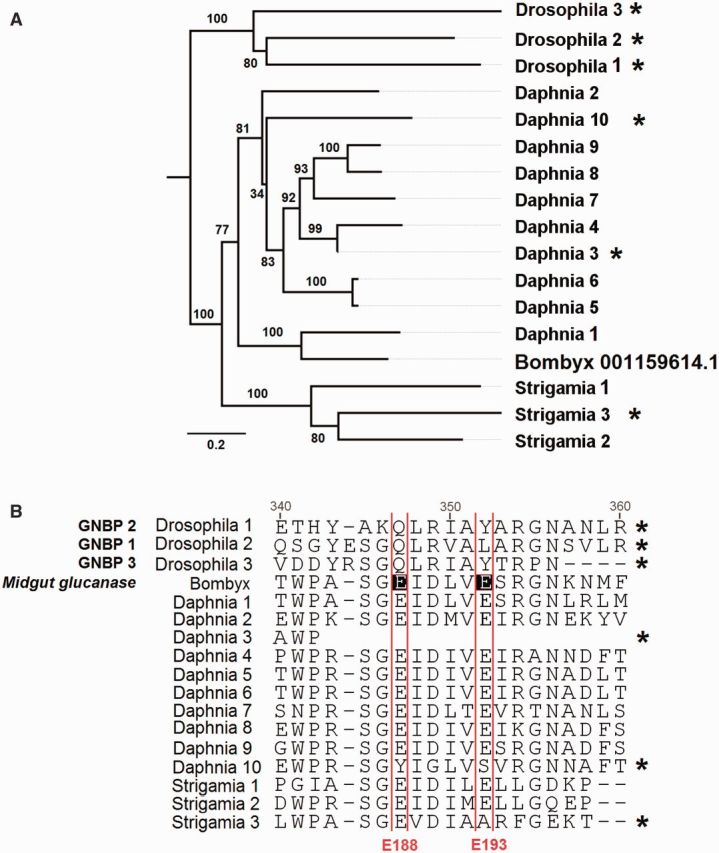
(*A*) BGRPs and β-1,3-glucanases have variable copy number in the Mandibulata but are absent from the chelicerates. The tree is midpoint rooted and was reconstructed by maximum likelihood. Node labels are bootstrap support from 1000 replicates. Scale bar is substitutions per site. (*B*) Alignment of BGRPs and glucanases identified in this study together with those from *Drosophila melanogaster* (Waterhouse et al. 2007) and a midgut β-1,3 glucanase from *Bombyx mori* (NP_001159614.1). Two conserved Glu active site residues found in all insect glucanases are labeled E188 and E193 after their position in NP_001159614.1 following Hughes (2012). Proteins that have lost one or both Glu residues, and so are expected to have lost glucanase activity, are labeled with an asterisk in both (*A*) and (*B*). The complete alignment may be found in the supplementary material, Supplementary Material online.

We find that proteins bearing the GH16 domain are limited to the Mandibulata (insects, crustaceans, and myriapods) and entirely absent from all of the chelicerates (table 1; fig. 5). This suggests a single loss event on the branch leading to chelicerates, as βGRPs and β-1,3-glucanases are present in other invertebrates such as mollusks (Zhang et al. 2012). *Drosophila* and *Strigamia* both possess three GH16-bearing proteins, whereas *Daphnia* has ten. From phylogenetic analysis, it would appear that these are the result of lineage-specific expansions (fig. 5).

Of the 13 GH16-bearing proteins that we identified, ten of them contain the two glutamate residues that are essential for enzymatic activity, suggesting that they are β-1,3-glucanases that may function in digestion or pathogen killing (supplementary table S5, Supplementary Material online, and fig. 5). There are two proteins in *Daphnia* and one in *Strigamia* that lack one or both of these residues and therefore presumably lack glucanase activity. The nonenzymatic proteins are not related to the *Drosophila* βGRPs, and, in a pattern similar to the PGRPs, the loss of glucanase activity has occurred on multiple occasions. Therefore, if these proteins function as pattern recognition receptors, our results would suggest that they have arisen independently by convergent evolution.

### Arthropod TEPs Include Relatives of Vertebrate C3 Complement Factors and Proteins Lacking the Thioester Motif

**Fig. 6.**
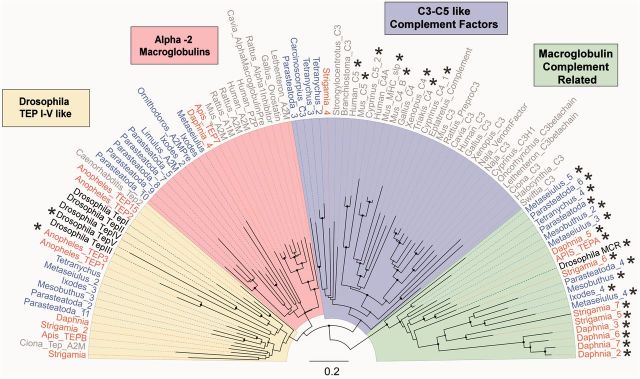
Gene tree of the TEP family. Sequences include arthropod TEPs, MCR proteins, the vertebrate C3, C4 and C5 complement factors, and alpha-2 macroglobulins. Genes without a thioester motif are shown with an “*.” Myriapod and crustacean taxon labels are orange, chelicerates blue, and *Drosophila* black. Taxa in gray are all deuterostomes with the exception of the nematode *Caenorhabditis*, which is a protostome related to arthropods, and the coral *Swiftia*, which diverged before the split of vertebrates and arthropods. In addition to the chelicerate sequences we annotated, we included two arthropod sequences from horseshoe crabs (*Limulus* and *Carcinoscorpius*) and a sequence from the tick *Ornithodoros.* The tree is midpoint rooted and was reconstructed using the maximum-likelihood method. Scale bar is substitutions per site. The additional taxa are taken from the previous analyses of Zhu et al. (2005), Wu et al. (2012), and Sekiguchi et al. (2012). Bootstrap support values can be found in supplementary figure S3, Supplementary Material online.

The TEPs include the vertebrate complement factors C3, C4, and C5, the insect TEPs, and a family of vertebrate protease inhibitors called alpha-2-macroglobulins. We found members of the TEPs family in all species, confirming that they were present in the common ancestor of the arthropods and have been retained in all the major arthropod lineages (fig. 6).

In *Drosophila* and mosquitoes*,* TEPs can covalently bind to the surface of pathogens and parasites, and mark them for destruction by phagocytosis or melanotic encapsulation (Levashina et al. 2001; Blandin et al. 2004; Stroschein-Stevenson et al. 2006). The TEPs have a characteristic thioester motif, which once the TEP has been cleaved into its active form can covalently bind to pathogens (Blandin et al. 2004; Sekiguchi et al. 2012). We found TEPs bearing the thioester motif in all species (fig. 6).

All the arthropod genomes also encode TEPs that lack the canonical thioester motif GCGEQ, and therefore presumably lack the ability to form covalent thioester bonds to microbial surfaces (fig. 6 and supplementary table S3, Supplementary Material online). Importantly, most of these proteins lack the critical cysteine required for the formation of thioester bonds (supplementary table S4, Supplementary Material online). All but two of these fall into a single clade, all the members of which lack this motif (fig. 6, highlighted green). This clade includes the *Drosophila* protein MCR (macroglobulin complement related or Tep VI), so we have named these MCR proteins.

Despite lacking the thioester motif, in *Drosophila* MCR can bind to the fungus *Candida albicans* and promote phagocytosis (Stroschein-Stevenson et al. 2006). In addition to this, MCR may also play a role in epithelial barrier formation preventing the spread of pathogens across tissues (Bätz et al. 2014). Our results show that MCR proteins were present in the common ancestor of arthropods and all eight of the species that we studied have at least one copy, with *Daphnia* having four copies and *Strigamia* and *Parasteatoda* having three copies (fig. 6; table 1)*.*

Phylogenetic analysis revealed proteins related to vertebrate C3 complement factors in the myriapod *Strigamia* and the chelicerates *Tetranychus* and *Parasteatoda* (fig. 6). The complement factors fall into a monophyletic group containing a single clade of vertebrate complement factors, a clade of arthropod sequences, and a basal lineage found in corals (fig. 6). The relationships of these clades therefore mirror the phylogeny of these three groups, and indicate an ancient origin of the C3 complement factors. These results support a previous finding of a sequence from the chelicerate *Carcinoscorpius* that was most similar to C3 (Zhu et al. 2005; fig. 6). Therefore C3-like proteins are widely scattered across the arthropods, but have been lost from most lineages including *Drosophila.*

The remaining two clades include the vertebrate alpha2 macroglobulins and the *Drosophila* TEPs (fig. 6, pink and yellow). Members of the *Drosophila* TEP clade are found in all the arthropod genomes we analyzed, although species other than *Drosophila* only have one or two copies (fig. 6, yellow). The function of these proteins has been best studied in *Anopheles* mosquitoes where they can bind bacteria and eukaryotic parasites, promoting phagocytosis and encapsulation (Levashina et al. 2001; Blandin et al. 2004). Interestingly *C. elegans Tep2* is a sister to this monophyletic group of arthropod TEPs. Sister to this group of insect TEPs we found a clear monophyletic grouping of vertebrate alpha-2 macroglobulins and arthropod sequences (fig. 6, pink). Of our sequences, we found five *Parasteatoda*, two *Ixodes*, one *Metaseiulus*,** and one *Daphnia* sequence to be alpha-2 macroglobulin-like. An insect sequence—TEP7 annotated in the honey bee genome project (Evans et al. 2006)—also fell into this clade.

### Gene Duplication Generates Diversity in the Immune Receptor Down Syndrome Cell Adhesion Molecule

Dscam (Down syndrome cell adhesion molecule) is a protein with a well-established function in neuronal patterning (Hattori et al. 2007) that is also thought to play a role in immunity. In *Drosophila* it is expressed in hemocytes, can bind to bacteria and knocking down its expression greatly reduces the rate at which bacteria are phagocytosed (Watson et al. 2005). In *Anopheles gambiae,* silencing *Dscam* compromises both the mosquito’s bacterial and malarial resistance and affects phagocytosis (Dong et al. 2006). Furthermore, in a number of different arthropods Dscam is upregulated after infection (Armitage et al. 2014). The *Dscam* gene has a remarkable level of alternative splicing—generating up to a possible 38,000 isoforms in *Drosophila*—which has led to speculation that this diversity contributes to a pathogen pattern-recognition repertoire in arthropods resembling that of vertebrate antibodies (Watson et al. 2005; Brites et al. 2008, 2013; Chou et al. 2009). However, little evidence has emerged to support this hypothesis, so it is unclear whether this extreme diversity is important for its immune function (Armitage et al. 2014).

There are multiple *Dscam* copies in all the species we studied, and frequent duplications or losses of the gene (fig. 6). Lineage-specific duplications of *Dscam* have resulted in 60 copies in *Strigamia* (a similar number were previously reported in this species; Brites et al. 2013) and 35 in the spider *Parasteatoda*, whereas in the other species we find 4 to 14 copies (fig. 6). Although we did not characterize the diversity generated through alternative splicing, these results suggest that *Dscam* diversity is an important trait that is generated in different ways across the arthropods. The Dscam paralogs we identified are an excellent system to test whether Dscam diversity plays a role in immunity or is important only to neuronal patterning.

### Fibrinogen-Related Proteins and Nimrod-Like Proteins

Fibrinogen-related proteins (FREPs) are found in taxa ranging from arthropods to mammals, and have been implicated in several antipathogen processes. These include vertebrate complement activation and binding to pathogens to act as pattern recognition receptors (Dong and Dimopoulos 2009). In mosquitoes, FREPs are upregulated following infection and key to effective anti-*plasmodium* and antibacterial responses (Dong and Dimopoulos 2009). They are also thought to contribute to the specificity of pathogen recognition in snails, where different FREPs bind to different classes of pathogens (Zhang et al. 2008). The copy number of FREPs in insects varies widely—in the genus *Drosophila* species have between 14 and 43 FREPs (Middha and Wang 2008), whereas *A. gambiae* has 59 (Waterhouse et al. 2007). Based on the conserved fibrinogen domain (Wang et al. 2005), we found all species to have *FREP*s, with copy number being highly variable. Notably, the mites *Metaseiulus* and *Tetranychus* contained only one and two fibrinogen domain containing proteins, respectively, whereas all other genomes contain between 19 and 36 (table 1).

Genes of the Nimrod superfamily are characterized by the presence of NIM-repeats, a specialized EGF-domain (Somogyi et al. 2008), and have roles in phagocytosis in insects, nematodes and humans (Somogyi et al. 2008). In *A. gambiae,* Nimrods are induced by bacteria and are important in bacterial killing (Estévez-Lao and Hillyer 2014). In addition to this immune role, in *Drosophila* the Nimrod *draper* is involved in the phagocytosis of apoptotic cells (Manaka et al. 2004).

We found multiple Draper-like proteins in all genomes except *Metaseiulus* and *Mesobuthus*, which may represent secondary losses or a lack of detection power. We failed to identify any B-type or C-type (e.g., Eater in *Drosophila*) Nimrod proteins in any species, perhaps due to the HMM-approach used being insufficiently sensitive.

**Fig. 7.**
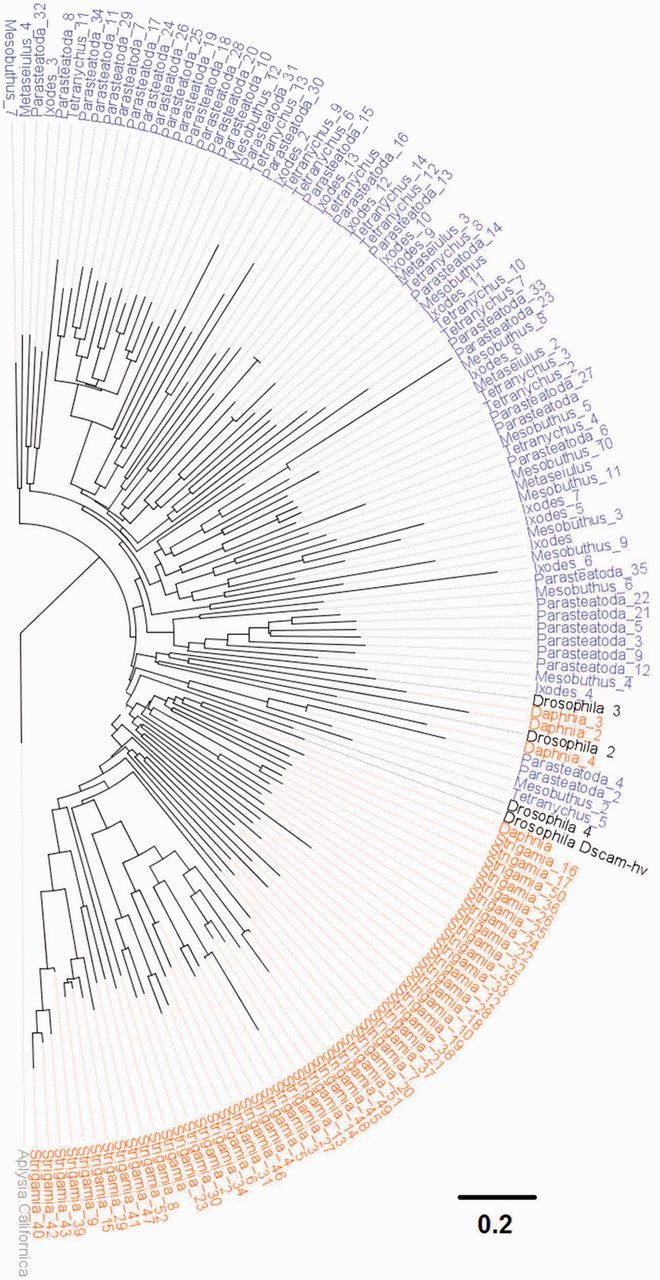
The diversity of Dscam in the arthropods. The tree is reconstructed using the maximum-likelihood method based on a complete Dscam amino acid sequence alignment and is rooted using the mollusk *Aplysia californica.* Myriapod and crustacean taxon labels are orange, chelicerates blue, and *Drosophila* black. Scale bar is substitutions per site. Bootstrap support values can be found in supplementary figure S4, Supplementary Material online.

### Prophenoloxidase and Melanization

Melanin is essential in the encapsulation of parasites, wound healing and the hardening of cuticle. Melanin is produced when the inactive zymogen prophenoloxidase (PPO) is cleaved into its active form, phenoloxide (PO), by a serine protease cascade and catalyses the hydroxylation and oxidation of phenols producing quinones. Quinones are toxic and play an important role in killing pathogens, and are the precursors of melanin, which physically encapsulates parasites (Jiang et al. 1998). The three *D. melanogaster* PPOs each bear Hemocyanin N, Hemocyanin M, and Hemocyanin C domains in that order. Hemocyanin M additionally has a tyrosinase motif, which may distinguish it from closely related proteins such as hexamarins and larval storage proteins (Burmester 2001). We find proteins bearing the three syntenic domains in *Daphnia, Strigamia, Mesobuthus,* and *Parasteatoda* (although conserved domain searches only predict tyrosinase activity in the *Parasteatoda* and *Mesobuthus* homologs; table 1). This protein family was greatly expanded in the arachnids, with 13 copies in *Parasteatoda* and 8 in *Mesobuthus.* These proteins may be functioning as PPOs or oxygen-carrying hemocyanins.

As PO activity generates toxic compounds, it is tightly regulated by a serine protease cascade and associated serine protease inhibitors (serpins) (Smith and DeLotto 1992; Jiang et al. 1998; Reichhart et al. 2011). Serine proteases and serpins are also important in other aspects of the immune response, such as the Toll signaling pathway (Smith and DeLotto 1992; Jiang et al. 1998; Jang et al. 2008; Reichhart et al. 2011). The main group of serine proteinases involved in immunity are the CLIP-domain serine proteinases, and these are found in all the species (table 1). The family appears to be greatly expanded in *Drosophila,* which has 24 copies (Jang et al. 2008), compared with the three to eight copies that we found other arthropods. Serpins are also found in all the species, with the myriapod *Strigamia* and the crustacean *Daphnia* having a lower copy number than *Drosophila* and the chelicerates (table 1).

### Dual Oxidase

Dual oxidase (*DUOX*) was found to be present in all taxa (table 1). DUOX generates reactive oxygen species (ROS) that are important intestinal microbicidal agents in *Drosophila* that maintain homeostasis between infectious and commensal bacteria (Ha et al. 2009; Chakrabarti et al. 2014). The expression of DUOX and ROS production is controlled by the stress-related p38 pathway (Chakrabarti et al. 2014). Beyond generating ROS to kill pathogens, DUOX plays a role in wound repair, the renewal of intestinal epithelial cells and signal transduction (Kim and Lee 2014). We found DUOX in all the species, with two copies in *Daphnia, Tetranychus,* and *Mesobuthus,* and three copies in the spider *Parasteatoda.*

### AMPs and Lysozymes

Lysozymes are enzymes that can cleave peptidoglycan in bacterial cell walls, and function both as immune effectors and digestive enzymes. They are found across the metazoa (Callewaert and Michiels 2010), and in insects are typically expressed in the gut and salivary glands (Daffre et al. 1994; Nayduch and Joyner 2013)*.* We found lysozymes in all taxa studied, with between one and five genes in the different species (table 1). This is less than the 13 found in *Drosophila* (Waterhouse et al. 2007)*,* which may be because fruit flies feed on bacteria-rich rotting fruit and require lysozymes for digestion (although some other arthropods have additional genes with the lysozyme-like domain, cl00222, which we conservatively did not include in our list).

AMPs are short peptides that are produced by animals and plants and kill a wide range of pathogens (Lemaitre and Hoffmann 2007). Many AMPs have been identified, and they are often restricted to a single clade (Gerardo et al. 2010). Furthermore, the small size of AMPs makes their identification by sequence similarity problematic across large phylogenetic distances. We searched for the functional domains associated with all the main *Drosophila* AMPs, and only found proteins bearing the Defensin2 (cl03093) functional domain in *Ixodes* and *Mesobuthus.*

### Antiviral RNAi

The primary antiviral defense of arthropods and many other animals is RNAi (Obbard, Gordon, et al. 2009). Double-stranded viral RNA is cleaved into short interfering RNAs (siRNAs) by Dicer, and then loaded into Argonaute proteins to direct the degradation of viral RNA. There are three RNAi pathways in insects. The piRNA pathway is Dicer-independent and involves PIWI family Argonautes, whereas the siRNA and miRNA pathways both rely on Argonaute-like Argonautes and Dicer proteins. It is the siRNA pathway that acts as a defense against viruses and somatic transposable element activity, and this involves the Argonaute Ago2 and the Dicer Dcr2 in *Drosophila* (Wang, Aliyari, et al. 2006; Ghildiyal et al. 2008; Obbard, Gordon, et al. 2009).

**Fig. 8.**
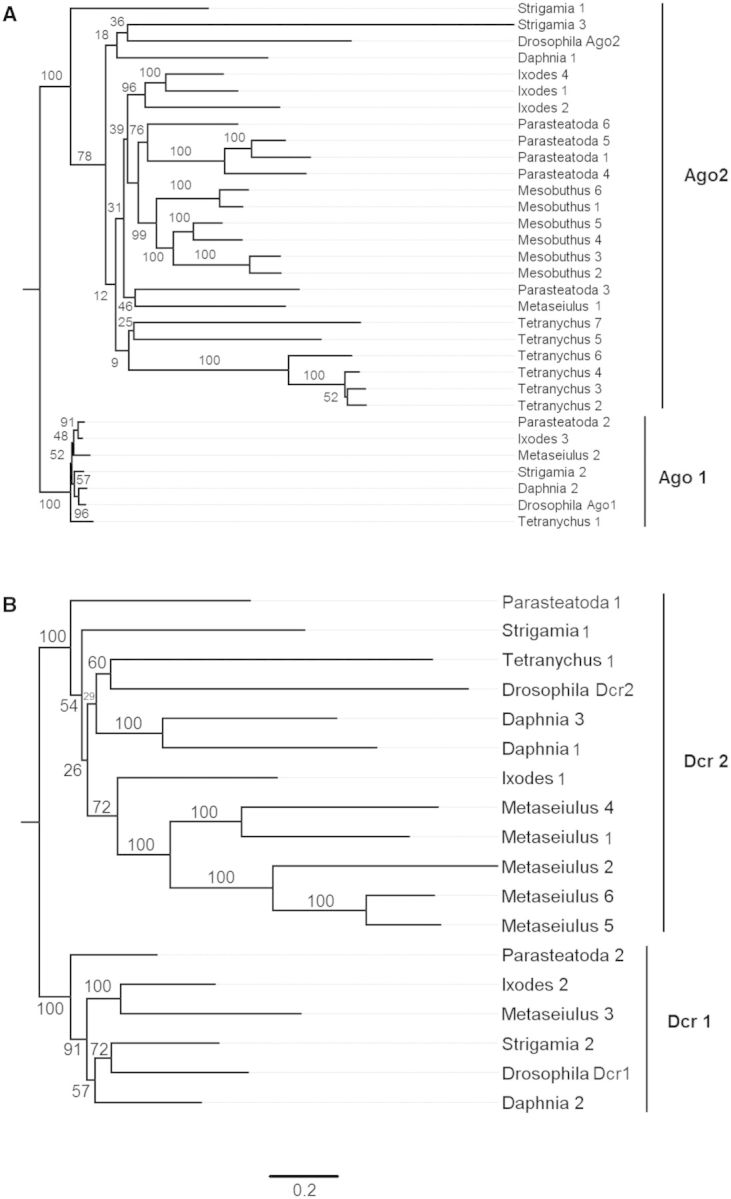
Proteins in the miRNA and antiviral siRNAi pathways. (*A*) Argonaute-family Argonaute proteins. (*B*) Dicer proteins. The trees are reconstructed by maximum likelihood and rooted following Schnettler et al. (2014). Node labels are bootstrap support from 1,000 replicates. Scale bar is substitutions per site.

Across the arthropods there has been considerable diversification of the antiviral siRNAi pathway, whereas the paralogous miRNA pathway is conserved (fig. 8*A*). This is most striking in *Ago2,* which has a highly variable copy number—*Drosophila, Daphnia*,** and *Metaseiulus* have a single copy, whereas in all the other species there are multiple paralogs, with *Tetranychus* and *Mesobuthus* having six copies (copy numbers in four of the species have been reported previously by Schnettler et al. (2014). Furthermore, these duplications of *Ago2* have occurred independently in the different species (fig. 8*A*). In common with previous work (Obbard et al. 2006), we also found that the rate of molecular evolution of *Ago2* is dramatically higher than its paralog *Ago1* (fig. 8*A*)*.*

*Dicer 2* has a single copy in most species, but it has been duplicated in *Daphnia* and in the mite *Metaseiulus* there are five copies (fig. 8*B*, supplementary table S5, Supplementary Material online, table 1). The absence of *Dicer* in *Mesobuthus* is likely an error in the genome assembly or annotation, as we were able to find a truncated *Dicer-*like gene.

## Conclusions

Our results confirm an ancient origin for the innate immune system, predating the split between protostomes and deuterostomes. We find striking examples of conservation between vertebrates and arthropods, despite these two groups having diverged before the Cambrian explosion some 543 Ma. These include a group of arthropod TLRs that share structural similarity with vertebrate TLRs and cluster with them phylogenetically. Similarly, several arthropods have C3-complement like factors that have been lost from *Drosophila.*

Despite such conservation, we also find remarkable diversity in the immune system of different arthropods. The Imd pathway—essential for recognition and response to Gram-negative bacteria in *Drosophila*—is almost entirely absent from the chelicerates. We also observe extensive copy number variation in recognition and effector genes, suggesting complex evolutionary dynamics in these functional categories. Detailed analysis of PGRPs suggests that this change in gene copy number is accompanied by changes in function.

Several factors may be driving the design and diversification of arthropod immune systems. Evolution involves tinkering with existing processes, and this is clear in the immune system. For example, the ability of enzymes to specifically bind molecules associated with pathogens has led to them losing their enzymatic activity and becoming pattern recognition receptors such as PGRPs and βGRPs. Melanin is produced by many species for various functions, and has been recruited to the immune system in arthropods. Here, the production of toxic compounds during melanin synthesis has been exploited for pathogen killing. Immune pathways may have arisen by combining modules of proteins with different functions—the Toll pathway in *Drosophila* looks more like a classical Nf-κb pathway combined with an extracellular serine protease cascade analogous to that controlling PPO activity. Similarly, the downstream part of the Imd pathway resembles a classical Nf-κb pathway, whereas the upstream part is similar to the mammalian tumor necrosis factor-receptor pathway.

Many components of the immune system have diversified very rapidly, and this likely reflects the need to counter an ever-changing array of parasites, as existing pathogens evolve to evade or sabotage immune responses, and entirely new pathogens appear in populations. For example, the widespread production of suppressors of RNAi by insect viruses may explain why the antiviral siRNA pathways evolve and diversify much faster than paralogous proteins in the micro-RNA pathway (Obbard et al. 2006; Obbard, Gordon, et al. 2009). Functional constraints are also likely to be important. For example, signaling pathway members relatively rarely show much copy number variation, despite natural selection driving rapid sequence evolution (Waterhouse et al. 2007; Obbard, Welch, et al. 2009). This may be because these pathways have many functions, so any change in copy number would have damaging pleiotropic effects.

The noninsect arthropods comprise a significant proportion of the earth’s biodiversity and include many species of economic and medical importance. Ours is the first detailed genome-wide analysis of arthropod immune systems outside of insects, and characterizing the function of the immune genes that we described remains an important challenge for the future. It is likely that many arthropods have immune defenses that are absent in *Drosophila* and therefore missing from our analysis, and these can only be discovered by experimental work on these species.

## Materials and Methods

### Data and Query Set

We retrieved the complete predicted-peptide sets for *D. melanogaster* (r5.54, Flybase) and seven additional noninsect arthropods: *S. maritima* (v1.20, Ensembl Genomes), *Met. occidentalis* (v1.0, NCBI Refseq), *M. martensii* (v1.0, Science Data Sharing Platform Bioinformation; Cao et al. 2013), *T. urticae* (v1.2, Ensembl Genomes; Grbić et al. 2011), *I. scapularis* (v1.2, Vectorbase), *Daphnia pulex* (r20, Ensembl Genomes; Colbourne et al. 2011), *Parasteatoda tepidariorum* (Augustus 3, Spiderweb) and performed all analyses on these data. A set of key immunity genes in *D. melanogaster* was compiled from Immunodb (Waterhouse et al. 2007), IIID (Brucker et al. 2012), Flybase (Pierre et al. 2014) and Obbard, Welch, et al. 2009, to be used as query sequences for homology searches (supplementary table S5, Supplementary Material online).

### Identification of Homologs

Broadly, to identify sequence homologues of a query set of *D. melanogaster* immunity genes (supplementary table S4, Supplementary Material online) we used multiple BLAST (Basic Local Alignment Search Tool)-based and hidden Markov model based approaches, to compile a redundant list of candidate homologues in each of the seven additional noninsect arthropod species. This list was then filtered by similarity, quality, *e* value, best reciprocal *Drosophila* hit, presence/absence of conserved domains known to be essential to function, and additionally by tree-based similarity measures, producing a final nonredundant list of high confidence predicted *Drosophila* innate immunity functional homologs in each peptide set.

### Ortholog Clustering

To identify clusters of orthologous genes, we performed all-versus-all BLAST-based clustering of protein sequences using Orthomcl V1.4 (Li et al. 2003) and the predicted peptide sets from all eight arthropod species. Default parameters were used, and homologues from each of the seven noninsect arthropods were assigned to immune homology groups based on clustering with a *Drosophila* innate immunity peptide from our query set (supplementary table S5, Supplementary Material online).

### HMMER

For a subset of genes, we searched for homologs in each full peptide-set using the hidden Markov model based HMMER (Eddy 2011). HMMER aims to be more sensitive than a traditional whole-sequence based search such as BLAST, by utilizing profile HMMs. Profile HMMs are statistical models of multiple sequence alignments whereby each residue in the alignment is determined to be more or less relevant to homology based on its conservation between sequences. Hits with an *e* value of less than 10^−^^5^ were considered significant and retained.

We built profile HMMs for each of the 18 signaling pathway peptides (Toll, Imd, JNK, and JAK/STAT), using multiple alignments of the *D. melanogaster* gene and its high-confidence orthologs in the immune-annotated insects *Bombyx mori, Tribolium castaneum,* and *Apis melifera* (Evans et al. 2006; Zou et al. 2007; Tanaka et al. 2008)*.* Multiple alignments were built using MAFFT (Katoh et al. 2009), and orthologous sequences were retrieved from Tanaka et al. (2008) (*B. mori*), Zou et al. (2007) (*T. castaneum*), and Evans et al. (2006) (*A*p*. melifera*).

For the highly variable and divergent Nimrod genes (for which BLAST/orthomcl does not find significant homologues, and no single diagnostic domain exists), we built a profile HMM of the conserved NIM motif CXPXCXXXCXNGXCXXPXXCXCXXGY (Kurucz et al. 2007; Somogyi et al. 2008), using the multiple alignment of Somogyi et al. (2008) and searched the complete peptide sets using HMMER (Eddy 2011).

### BLASTp

We also searched all seven non-*Drosophila* peptide-sets for homologues of *Drosophila* immune genes using BLASTp, retaining hits with an *e* value less than 10^−^^6^, greater than 20% identity, and a bit score greater than 80. We additionally queried each species’ complete peptide-set against a BLAST database of all *D. melanogaster* peptides, in order to identify the best *Drosophila* BLASTp hit for each gene in each other species.

### Identification of Protein Domains

Some gene classes rely on a conserved protein domain to function in the manner described in *D. melanogaster,* without which the protein was assumed to be nonfunctional and therefore discarded. We searched each whole non-*Drosophila* peptide-set for domains known to function in innate immune pathways using the NCBI BatchConservedDomain tool and identified proteins containing domains listed in the query set (supplementary table S1, Supplementary Material online).

### Combining Results and Further Analyses

We finally compiled results from all the above techniques into a single list of potential immunity homologs. Examining these results individually, we filtered out hits where either an essential protein domain was absent (supplementary table S1, Supplementary Material online) or the reciprocal top *D. melanogaster* blast hit was to a nonimmunity-related gene.

For PGRPs, GNBPs/βGRPs, and TLRs, we scanned putative homologues for transmembrane helices and signal peptides using the TMHMM Server v. 2.0 (Krogh et al. 2001) and the TargetP 1.1 Server (Emanuelsson et al. 2007), respectively. We also built a profile HMM of the PGRP RHIM (Imd-binding) domain after Kaneko et al. (2006) and Meister et al. (2009), and scanned putative PGRPs in all species to predict presence/absence of the RHIM domain.

Toll-like proteins/receptors are characterized by an N-Terminal TIR domain, a transmembrane helix, and a variable number of LRRs extending to the C-terminal. TIR domains and transmembrane helices were identified as above, whereas LRRs were identified using the web-interface of LRR-finder (Offord et al. 2010).

We additionally built trees of gene families where duplications were suspected to be assembly errors (e.g., haplotypes annotated as separate contigs), or to distinguish orthologs and paralogs. Multiple alignments were assembled using MAFFT (Katoh et al. 2009), with phylogenetic tree construction performed in PHYML (Guindon et al. 2010) using the WAG+G model. Shimodaira–Hasegawa tests to compare the log-likelihood of differing tree topologies were performed in RAxML (Stamatakis 2014) using the WAG+G model and multiple alignments created as above in MAAFT.

## Supplementary Material

Supplementary alignments, figures S1–S4, and tables S1–S7 are available at *Molecular Biology and Evolution* online (http://www.mbe.oxfordjournals.org/).

Supplementary Data
