# Supplementary material for: Comparative Genomics Reveals the Origins and Diversity of Arthropod Immune Systems
Source: Mol Biol Evol. 2015 Apr 22;32(8):2111–29. doi: 10.1093/molbev/msv093 (PMC4833078; doi:10.1093/molbev/msv093)
Supplement: Supplementary Data [file supp_32_8_2111__index.html]

Comparative genomics reveals the origins and diversity of arthropod immune systems — Comparative Genomics Reveals the Origins and Diversity of Arthropod Immune Systems — Comparative Genomics Reveals the Origins and Diversity of Arthropod Immune Systems — Supplementary Data 

# Comparative Genomics Reveals the Origins and Diversity of Arthropod Immune Systems

## Supplementary Data

files

**Files in this Data Supplement:**

- Supplementary Data - pdf file
- Supplementary Data - pdf file
