# Supplementary material for: Comparative Genomics Reveals the Origins and Diversity of Arthropod Immune Systems
Source: Mol Biol Evol. 2015 Apr 22;32(8):2111–29. doi: 10.1093/molbev/msv093 (PMC4833078; doi:10.1093/molbev/msv093)
Supplement: Supplementary Data [file supp_msv093_SUPPLEMENTAL_Alignments_Rev1_March15.pdf]

Comparative genomics reveals the origins and diversity of arthropod immune systems

Supplementary Alignments

William J. Palmer* and Francis M. Jiggins

Department of Genetics, University of Cambridge, Downing Street, Cambridge CB2 3EH UK

* corresponding author; w.palmer@gen.cam.ac.uk

TIR Domain Alignment for Figure 1

>Drosophila_Toll9 FBpp0077898 ProteinLength=900 DomainCoordinates=754-884 DomainLength=130 Accession=pfam01582 ID=8

----ISY-CQNDR---TWVLNELLPNVEET--------GD---------------VSICLHERDFQ-IGV--T-ILDN-----------
IISCMDRSYSLMLIISSKFLLSHWC-QFEMYLAQHRIFEVSK-------------EHLIL-VFLEDIPRRKRPKT---------LQ--
YLMDVKTYIK--WPTAKEDRKLFWKRLK-------

>Drosophila_Toll6 FBpp0297411 ProteinLength=1514 DomainCoordinates=1114-1247 DomainLength=133 Accession=smart00255 ID=5

--AYFAY-SLQDE---HFVNQILAQTLEND--------IG---------------YRLCLHYRDVN-INA--Y-ITDA-----------
LIEAAESAKQFVLVLSKNFLYNEWS-RFEYKSALHELV-KRR-------------KRVVF-ILYGDLPQRD-IDMD--------MR--
HYLRTSTCIE--W-----DDKKFWQKLRLALPLPN

>Drosophila_Toll7 FBpp0085638 ProteinLength=1446 DomainCoordinates=1101-1232 DomainLength=131 Accession=pfam01582 ID=6

----LLH-SAKDS---EFVCQHLAAQLETG--------RPP--------------LRVCLQHRDLAHDATHYQ----------------
LLEATRVSRRVVILLTRNFLQTEWA-RCELRRSVHDALRGRP-------------QKLVI-IEEPEVAFEAESDIE--------LL--
PYLKTSAVHR-----IRRSDRHFWEKLRYAL----

>Drosophila_Toll4 FBpp0079369 ProteinLength=1125 DomainCoordinates=974-1110 DomainLength=136 Accession=smart00255 ID=3

YDAFLSF-THKDE---DLIEE-FVDRLENG--------RHK--------------FRLCFYLRDWL-VGE--S-IPDC-----------
INQSVKGSRRIIILMTKNFLKSTWG-RLEFRLALHATS-RDR------------CKRLIV-VLYPDVEHFDDLDSE--------LR--
AYMVLNTYLD--R-----NNPNFWNKLMYSMPHA-

>Drosophila_Toll8 FBpp0075360 ProteinLength=1346 DomainCoordinates=1076-1211 DomainLength=135 Accession=smart00255 ID=7

-DAFVSY-SSKDE---LFVNEELAPMLEMG--------EHR--------------YKLCLHQRDFP-VGG--Y-LPET-----------
IVQAIDSSRRTIMVVSENFIKSEWC-RFEFKSAHQSVL-RDR------------RRRLIV-IVLGEVPQKE-LDPD--------LR--
LYLKTNTYLQ--W-----GDKLFWQKLRFALPDV-

>Drosophila_Toll5 FBpp0080080 ProteinLength=795 DomainCoordinates=646-776 DomainLength=130 Accession=pfam01582 ID=4

---FISY-SHKDE---ELISK-LLPKLESG--------P---HP-----------FRLCLHDRDWL-VGD--C-IPEQ-----------
IVRTVDDSKRVIIVLSQHFIDSVWA-RMEFRIAYQATL-QDK------------RKRIII-ILYRELEHMNGIDSE--------LR--
AYLKLNTYLK--W-----GDPLFWSKLYYAM----

>Drosophila_Toll3 FBpp0081104 ProteinLength=964 DomainCoordinates=826-956 DomainLength=130 Accession=pfam01582 ID=2

----LAF-THKDE---ALLEE-FVDRLERG--------RPR--------------FQLCFYLRDWL-AGE--S-IPDC-----------
IGQSIKDSRRIIVLMTENFMNSTWG-RLEFRLALHATS-RDR------------CKRLIV-VLYPNVKNFDSLDSE--------LR--
TYMAFNTYLE--R-----SHPNFWNKLIYSMP---

>Drosophila_18w FBpp0085620 ProteinLength=1385 DomainCoordinates=1048-1179 DomainLength=131 Accession=pfam01582 ID=1

----ILH-SEKDY---EFVCRNIAAELEHG--------RPP--------------FRLCIQQRDLPPQASHLQ----------------LVEGARASRKIILVLTRNLLATEWN-RIEFRNAFHESLRGLA-------------QKLVI-IEETSVSAEAEDVAE--------LS--PYLKSVPSNR-----LLTCDRYFWEKLRYAI----

>Drosophila_Toll FBpp0303187 ProteinLength=1116 DomainCoordinates=858-996 DomainLength=138 Accession=smart00255 ID=0

FDAFISY-SHKDQ---SFIEDYLVPQLEHG--------P---QK-----------FQLCVHERDWL-VGG--H-IPEN-----------IMRSVADSRRTIIVLSQNFIKSEWA-RLEFRAAHRSAL-NEG------------RSRIIV-IIYSDIGDVEKLDEE--------LK--AYLKMNTYLK--W-----GDPWFWDKLRFALPHRR

>TLR10_HUMAN ProteinLength=811 DomainCoordinates=633-775 DomainLength=142 Accession=cl17458 ID=0

FHAFISY-SEHDS---LWVKNELIPNLEKE--------DGS--------------ILICLYESYFD-PGK--S-ISEN-----------IVSFIEKSYKSIFVLSPNFVQNEWC-HYEFYFAHHNLFHENS-------------DHIIL-ILLEPIPFYCIPTRY------HKLK--ALLEKKAYLE--WPKDRRKCGLFWANLRAAIN---

>TLR13_MOUSE ProteinLength=991 DomainCoordinates=834-976 DomainLength=142 Accession=cl17458 ID=1

-DAFVSF-SATDE---AWVYKELVPALEQG--------SQTT-------------FKLCLHQRDFE-PGI--D-IFEN-----------IQNAINTSRKTLCVVSNHYLHSEWC-RLEVQLASMKMFYEHK-------------DVIIL-IFLEEIPNYKLSSYH-------RLR--KLINKQTFIT--WPDSVHQQPLFWARIRNALGK--

>TLR12_MOUSE ProteinLength=906 DomainCoordinates=761-902 DomainLength=141 Accession=cl17458 ID=2

-DVFVSH-CRQDQ---GWVIEELLPALEGF--------LPAGLG-----------LRLCLPERDFE-PGK--D-VVDN-----------VVDSMLSSRTTLCVLSGQALCNPRC-RLELRLATSLLLAAPS-------------PPVLLLVFLEPISRHQLPGYH-------RLA--RLLRRGDYCL--WPEEEERKSGFWTWLRS------

>TLR1_MOUSE ProteinLength=795 DomainCoordinates=639-782 DomainLength=143 Accession=cl17458 ID=3

FHAFVSY-SGHDS---AWVKNELLPNLEKD--------D----------------IQICLHERNFV-PGK--S-IVEN-----------IINFIEKSYKSIFVLSPHFIQSEWC-HYELYFAHHNLFHEGS-------------DNLIL-ILLAPIPQYSIPTNY------HKLK--TLMSRRTYLE--WPTEKNKHGLFWANLRASINVKL

>TLR21_CHICK ProteinLength=793 DomainCoordinates=649-793 DomainLength=144 Accession=cl17458 ID=4

YDAFVSY-SENDS---NWVENIMVQQLEQA--------CPP--------------FRLCLHKRDFV-PGK--W-IVDN-----------IIDSIEKSHKTLFVLSEHFVQSEWC-KYELDFSHFRLFDENN------------DVAIL-ILLEPIQSQAIPKRF------CKLR--KIMNTKTYLE--WPPDEEQQQMFWENLKAALKS--

>TLR1_HUMAN ProteinLength=786 DomainCoordinates=636-779 DomainLength=143 Accession=cl17458 ID=5

FHAFISY-SGHDS---FWVKNELLPNLEKE--------G---------------MQICLHERNFV-PGK--S-IVEN-----------
IITCIEKSYKSIFVLSPNFVQSEWC-HYELYFAHHNLFHEGS-------------NSLIL-ILLEPIPQYSIPSSY------HKLK--
SLMARRTYLE--WPKEKSKRGLFWANLRAAINIKL

>TLR22_CHICK ProteinLength=781 DomainCoordinates=637-781 DomainLength=144 Accession=cl17458 ID=6

YDAFVSY-SENDS---NWVENIMVQQLEQA--------CPP--------------FRLCLHKRDFV-PGK--W-IVDN----------
IIDSIEKSHKTLFVLSEHFVQSEWC-KYELDFSHFRLFDENN-------------DVAIL-ILLEPIQSQAIPKRF------CKLR--
KIMNTKTYLE--WPPDEEQQQMFWENLKAALKS--

>TLR11_MOUSE ProteinLength=926 DomainCoordinates=776-856 DomainLength=80 Accession=cl17458 ID=7

---FISY-CEEDQ---AWVLEELVPVLEKA--------PPEGEG-----------LRLCLPARDFG-IGN--D-RMES-----------
MIASMGKSRATLCVLTGQALASPWC-NLELRLAT--

>TLR2_MOUSE ProteinLength=784 DomainCoordinates=640-784 DomainLength=144 Accession=cl17458 ID=8

YDAFVSY-SEQDS---HWVENLMVQQLENS--------DPP--------------FKLCLHKRDFV-PGK--W-IIDN----------
IIDSIEKSHKTVFVLSENFVRSEWC-KYELDFSHFRLFDENN-------------DAAIL-VLLEPIERKAIPQRF------CKLR--
KIMNTKTYLE--WPLDEGQQEVFWVNLRTAIKS--

>TLR2_HUMAN ProteinLength=784 DomainCoordinates=640-784 DomainLength=144 Accession=cl17458 ID=9

YDAFVSY-SERDA---YWVENLMVQELENF--------NPP--------------FKLCLHKRDFI-PGK--W-IIDN----------
IIDSIEKSHKTVFVLSENFVKSEWC-KYELDFSHFRLFDENN-------------DAAIL-ILLEPIEKKAIPQRF------CKLR--
KIMNTKTYLE--WPMDEAQREGFWVNLRAAIKS--

>TLR3_MOUSE ProteinLength=905 DomainCoordinates=759-897 DomainLength=138 Accession=cl17458 ID=10

---YIIH-AHKDR---DWVWEHFSPMEEQD--------QS---------------LKFCLEERDFE-AGV--L-GLEA-----------
IVNSIKRSRKIIFVITHHLLKDPLCRRFKVHHAVQQAIEQNL-------------DSIIL-IFLQNIPDYKLNHAL------CLRR--
GMFKSHCILN--WPVQKERINAFHHKLQVAL----

>TLR3_HUMAN ProteinLength=904 DomainCoordinates=758-896 DomainLength=138 Accession=cl17458 ID=11

---YIIH-AYKDK---DWVWEHFSSMEKED--------QS---------------LKFCLEERDFE-AGV--F-ELEA-----------
IVNSIKRSRKIIFVITHHLLKDPLCKRFKVHHAVQQAIEQNL-------------DSIIL-VFLEEIPDYKLNHAL------CLRR--
GMFKSHCILN--WPVQKERIGAFRHKLQVAL----

>TLR4_HUMAN ProteinLength=839 DomainCoordinates=676-814 DomainLength=138 Accession=cl17458 ID=12

---FVIY-SSQDE---DWVRNELVKNLEEG--------VPP--------------FQLCLHYRDFI-PGV--AIAANI-----------IHEGFHKSRKVIVVVSQHFIQSRWC-IFEYEIAQTWQFLSSR-------------AGIIF-IVLQKVEKTLLRQQ---------VELYRLLSRNTYLE--WEDSVLGRHIFWRRLRKAL----

>TLR5_MOUSE ProteinLength=859 DomainCoordinates=696-833 DomainLength=137 Accession=cl17458 ID=13

---YFCF-SSKDF---EWAQNALLKHLDAH--------YSSRNR-----------LRLCFEERDFI-PGE--NHISN------------IQAAVWGSRKTVCLVSRHFLKDGWC-LEAFRYAQSRSLSDLK-------------SILIV-VVVGSLSQYQLMRHE-------TIR--GFLQKQQYLR--WPEDLQDVGWFLDKLS-------

>TLR5_HUMAN ProteinLength=858 DomainCoordinates=695-832 DomainLength=137 Accession=cl17458 ID=14

---YLCF-SSKDF---TWVQNALLKHLDTQ--------YSDQNR-----------FNLCFEERDFV-PGE--NRIAN------------IQDAIWNSRKIVCLVSRHFLRDGWC-LEAFSYAQGRCLSDLN-------------SALIM-VVVGSLSQYQLMKHQ-------SIR--GFVQKQQYLR--WPEDFQDVGWFLHKLS-------

>TLR6_MOUSE ProteinLength=795 DomainCoordinates=641-780 DomainLength=139 Accession=cl17458 ID=15

FHAFVSY-SEHDS---AWVKNELLPNLEKD--------D----------------IRVCLHERNFV-PGK--S-IVEN-----------IINFIEKSYKAIFVLSPHFIQSEWC-HYELYFAHHNLFHEGS-------------DNLIL-ILLEPILQNNIPSRY------HKLR--ALMAQRTYLE--WPTEKGKRGLFWANLRASF----

>TLR7_MOUSE ProteinLength=1050 DomainCoordinates=894-1035 DomainLength=141 Accession=cl17458 ID=16

---FIVY-DTKNSAVTEWVLQELVAKLEDP--------REKH-------------FNLCLEERDWL-PGQ--P-VLEN-----------LSQSIQLSKKTVFVMTQKYAKTESF-KMAFYLSHQRLLDEKV-------------DVIIL-IFLEKPLQKSKFLQ---------LR--KRLCRSSVLE--WPANPQAHPYFWQCLKNALTT--

>TLR7_HUMAN ProteinLength=1049 DomainCoordinates=893-1034 DomainLength=141 Accession=cl17458 ID=17

---FIVY-DTKDPAVTEWVLAELVAKLEDP--------REKH-------------FNLCLEERDWL-PGQ--P-VLEN-----------LSQSIQLSKKTVFVMTDKYAKTENF-KIAFYLSHQRLMDEKV-------------DVIIL-IFLEKPFQKSKFLQ---------LR--KRLCGSSVLE--WPTNPQAHPYFWQCLKNALAT--

>TLR4_MOUSE ProteinLength=835 DomainCoordinates=674-812 DomainLength=138 Accession=cl17458 ID=18

---FVIY-SSQNE---DWVRNELVKNLEEG--------VPR--------------FHLCLHYRDFI-PGV--AIAANI-----------IQEGFHKSRKVIVVVSRHFIQSRWC-IFEYEIAQTWQFLSSR-------------SGIIF-IVLEKVEKSLLRQQ---------VELYRLLSRNTYLE--WEDNPLGRHIFWRRLKNAL----

>TLR6_HUMAN ProteinLength=796 DomainCoordinates=641-780 DomainLength=139 Accession=cl17458 ID=19

FHAFISY-SEHDS---AWVKSELVPYLEKE--------D----------------IQICLHERNFV-PGK--S-IVEN-----------IINCIEKSYKSIFVLSPNFVQSEWC-HYELYFAHHNLFHEGS-------------NNLIL-ILLEPIPQNSIPNKY------HKLK--ALMTQRTYLQ--WPKEKSKRGLFWANIRAAF----

>TLR8_MOUSE ProteinLength=1032 DomainCoordinates=873-1006 DomainLength=133 Accession=cl17458 ID=20

---YISY-DTKDASVTDWVINELRYHLEES--------EDKS-------------VLLCLEERDWD-PGL--P-IIDN-----------LMQSINQSKKTIFVLTKKYAKSWNF-KTAFYLALQRLMDENM-------------DVIIF-ILLEPVLQYSQYLR---------LR--QRICKSSILQ--WPNNPKAENLFWQ----------

>TLR8_HUMAN ProteinLength=1041 DomainCoordinates=882-1020 DomainLength=138 Accession=cl17458 ID=21

---YISY-DTKDASVTDWVINELRYHLEES--------RDKN-------------VLLCLEERDWD-PGL--A-IIDN-----------LMQSINQSKKTVFVLTKKYAKSWNF-KTAFYLALQRLMDENM-------------DVIIF-ILLEPVLQHSQYLR---------LR--QRICKSSILQ--WPDNPKAEGLFWQTLRNV-----

>TLR9_MOUSE ProteinLength=1032 DomainCoordinates=872-1012 DomainLength=140 Accession=cl17458 ID=22

---FVVF-DKAQSAVADWVYNELRVRLEER--------RGRRA------------LRLCLEDRDWL-PGQ--T-LFEN-----------LWASIYGSRKTLFVLAHTDRVSGLL-RTSFLLAQQRLLEDRK-------------DVVVL-VILRPDAHRSRYVR---------LR--QRLCRQSVLF--WPQQPNGQGGFWAQLSTAL----

>TLR9_HUMAN ProteinLength=1032 DomainCoordinates=872-1009 DomainLength=137 Accession=cl17458 ID=23

---FVVF-DKTQSAVADWVYNELRGQLEEC--------RGRWA------------LRLCLEERDWL-PGK--T-LFEN-----------LWASVYGSRKTLFVLAHTDRVSGLL-RASFLLAQQRLLEDRK-------------DVVVL-VILSPDGRRSRYVR---------LR--QRLCRQSVLL--WPHQPSGQRSFWAQLG-------

>TOL1_CELEG ProteinLength=1221 DomainCoordinates=1056-1194 DomainLength=138 Accession=cl17458 ID=0

-HAFVSY-SKKDE---KMVIDQLCRPLEDE--------D----------------YQLCLLHRDGPTYCSNLHAISDE-----------LIAQMDSSQCLILVLTKHFLENEWK-TLQIKTSH-QLFAKNR-------------AKRVI-AVLGDGVDANLLDDE--------LG--QILRKHTRIE--M-----RSHLFWTLLHSSLPSRL

>TLR2_CIONA ProteinLength=947 DomainCoordinates=802-906 DomainLength=104 Accession=cl17458 ID=2

YDAFVSYVSDSDD---VEFVYKMLEEMEEK--------RE---------------RKMCIHERDFT-PGR--G-IADN-----------IVECISTSRRMVLVVSRKYASSAWC-QYEVQIALTELHAKRR-------------GRLLVPILLEDVTRDE--

>TLR1_CIONA ProteinLength=882 DomainCoordinates=735-879 DomainLength=144 Accession=cl17458 ID=1

---YIS--CVPDSVDEAWVVRQLLCAIENP--------PYELTP-----------MKLCFPSRDFK-PGC--PKMVSA------------ANNLRLSKHALVILSKDYVANSWT-RFELSMVSEMWRNSER-----------SAESLIV-VYLKRHGESLVGVER--------LPV-LGVRRNAWLV--WPTDVADRPSFWMKLRRSL----

>Daphnia DappuP190084 ProteinLength=453 DomainCoordinates=238-371 DomainLength=133 Accession=cl17458 ID=316

---FISF-SHNDE---KFVDE-LVAQLERP--------PVGLPN-----------YQLCLHHRDWL-AGE--W-IPDQ-----------IVRSVASSKRTVVILTENFLDSFWG-KLEFRTAYQQVL-KDK------------RMRLIV-IVKGELPPKDKMDTE--------LQ--TYLSLNTYLK--Y-----DDPFFMERLRYAL----

>Daphnia_2 DappuP97929 ProteinLength=1093 DomainCoordinates=882-1016 DomainLength=134 Accession=cl17458 ID=4117

---FISF-SHHDE---IFVNEVLVPQLERP--------PIGLPH-----------YQLCIHYRDWL-AGE--W-IADQ-----------IVRSVATSKRTIVVLTENFLDSLWG-KLEFRTAYKQVL-TDK------------RMRLII-IVKGELPPFDKMDQE--------LQ--TYLSLNTYLK--Y-----DDPFFMDRLRYAL----

>Daphnia_3 DappuP314608 ProteinLength=854 DomainCoordinates=701-840 DomainLength=139 Accession=cl17458 ID=5793

YDAFVSY-SNVDH---AFVAR-MVGMLENA--------PPH--------------YKLCVYERDFT-AGN--V-LNDC-----------IMQSIATSRKVVLVISENFIQSHWC-LWELHLAQHSLLEDKR-------------NGLVL-VVVGKLKLNQCPPT---------LR--FLMKTRIYLE--WDLDPSKQRVFWERLRDALAP--

>Daphnia_4 DappuP128614 ProteinLength=1332 DomainCoordinates=1057-1211 DomainLength=154 Accession=cl17458 ID=11512

FDAFVSY-SLKDE---QFVSQVLAAELEHS--------AEAGSS-----------FRLCLQHRDFP-TSHSGSSSTNSSNSSSNPGGDPLTLGLAASRRIVLVISQSFIESEWT-RPEVRTALTGFLRLPR-------------SRLVA-VLLTPWTDDQ-SDPE--------LS--LLLRSSIIIR--W-----GERNFWSKIRYYLPDPT

>Daphnia_5 DappuP65779 ProteinLength=1305 DomainCoordinates=1073-1225 DomainLength=152 Accession=cl17458 ID=25686

-DAFLSY-SAKDD---AFVQQMLATNLEYG--------SPT--------------YKLCLQHRDCP-SGGGAYGLSET-----------ISQAVDSSRRTVMIISPNFIKAEWC-RFEYKSALHQLFGTSRHCQQQQTKSAKQTKRLIV-ILIGDVTHKD-LDAD--------LK--LYLKTNTYLQ--W-----GEDGFWDKLRFALPDPV

>Strigamia SMAR012279-PA ProteinLength=930 DomainCoordinates=771-906 DomainLength=135 Accession=cl17458 ID=128387

-DAFVSY-SSEDE---DWIADFLVPGLESG--------I---PS-----------YKLCLHNRDWP-AGE--F-ITDQ-----------IVRSVGNSRRTILVLTDNYFKSGWS-RLEFDIAYQQGL-KDK------------VNRLIA-VVPNEVPDLSKIDQD--------FK--TFITLTTYVE--A-----KKPYFWRKLRASMPR--

>Strigamia_2 SMAR011407-PA ProteinLength=1015 DomainCoordinates=847-978 DomainLength=131 Accession=cl17458 ID=129379

---FISF-SQEDM---DWVATNLMPGLEQH--------EPH--------------YRLCIHHRDWL-VGE--W-IPDQ-----------
IVRSVEDSCRTIVVLSTNFIRSVWG-RLEFKTAHHQAL-QDR------------MNRVIV-IVLGEVPPKDEMDPD--------
MR--MYVGLNTYLR--W-----EDPWFWKKLRYAM----

>Strigamia_3 SMAR011269-PA ProteinLength=1080 DomainCoordinates=933-1065 DomainLength=132 Accession=cl17458 ID=129588

-DAFISY-ANEDD---EWITEELVHRLNE--------------------------YRLCIHQRDFP-VGG--L-IADS-----------
IAHAVQNSCRTLIVLTPNFLQSQWC-QFEFKTAHLQSL-ENK------------CQRVIV-IVLERVKNLE-LDKN--------LR--
AYLKTNTYLD--I-----NDINFWRKLKSALPDLK

>Strigamia_4 SMAR010912-PA ProteinLength=921 DomainCoordinates=763-898 DomainLength=135 Accession=cl17458 ID=129997

-DAFVSY-SGEDE---DWIVDFLVPGLETG--------I---PS-----------YKLCLHSRDWR-AGE--F-ITDQ-----------
IVRSVESSRRTLIVLTDSYLKSEWS-RLEFDVAYQQAL-RDQ------------VRRLIA-IVPNEVPDLSKIDQE--------FK--
TFITLTTYIE--A-----KKPYFWRKLRASMPR--

>Strigamia_5 SMAR014647-PA ProteinLength=853 DomainCoordinates=707-845 DomainLength=138 Accession=cl17458 ID=130618

YDAFVSY-SSADG---EWVRDVLVAGLEEG--------Q---PA-----------YKLCLHERDWL-AGE--F-IPEQ-----------
IVLSVQESRRTIIVLTPDFLSSAWS-QIEFNVAYHKAF-EDR------------VRRLIV-VVPKEMPKLDMARDD--------LR--
AFLATTTYLE--A-----SKPHFWTKLRCSMPRIR

>Strigamia_6 SMAR008553-PA ProteinLength=1472 DomainCoordinates=1282-1417 DomainLength=135 Accession=cl17458 ID=132841

-DAFLSY-SSEDS---EIIVNDLLPGLEKA--------D----------------FKVCVHERDWL-GGQ--F-ISEQ-----------
IVISVHTSRRTIIVLSESFLRSPWA-TMEFKVALQQAL-EDR------------VNRIII-VVPGKLPSKDQMDPD--------LR--
SFVTMNTYLF--L-----SDSRFWDKLMFAMPRKT

>Strigamia_7 SMAR008376-PA ProteinLength=1169 DomainCoordinates=1031-1166 DomainLength=135 Accession=cl17458 ID=132962

-DAYICY-SSNDE---CFVRNILAPDLERG--------GEHS-------------YFLVLHHRDCP-VGS--F-ENDI-----------
VIEASDNSKRFILVLTQNFIKNEWS-RNQVKENFIQLF-KER------------HTPLIV-VVFGVVSKRD-MDQE--------LR--
LCLKNSTCIT--W-----GDKLFWAKLKLALPE--

>Strigamia_8 SMAR006691-PA ProteinLength=882 DomainCoordinates=738-870 DomainLength=132 Accession=cl17458 ID=134928

-GAFISY-AEEDE---DWITEELLPQLED--------------------------YKLCILQRDFP-IGD--K-V-ES-----------
LLQAVESSCRTVVILTPNYLKSDWC-QFEFDTAHMQSL-QDK------------CERLVV-VRLEAVDNCE-MNTN--------
LG--AYLKTNTYLDINI-----NDGLFWEKLKFALPDV-

>Strigamia_9 SMAR005841-PA ProteinLength=1734 DomainCoordinates=1563-1694 DomainLength=131 Accession=cl17458 ID=135837

---FVSY-ANEDT---DFVTETLVPELEGK--------PPH-------------YRLCIHERDWK-VGE--Q-IVEQ-----------IEESVLNSRRVIIILTQNFINSMWS-SLEFKTAHYHSM-QEK------------ANRIIV-IVIGELPAKETLDSD--------LR--MHINLNTYLK--W-----EDKQFWNKLRYAM----

>Strigamia_10 SMAR014575-PA ProteinLength=1109 DomainCoordinates=968-1093 DomainLength=125 Accession=cl17458 ID=136445

----ISY-ANEDG---EWVAQRLVPRLTE-------------------------YRLCIHQRDFP-VGG--F-IAES-----------IVHAVENSCRTVIVLTPNFLKSEWC-RFEFESAHIQSL-EDR------------CKRLIV-IVLEQVDNGG-LNKN--------LL--AYMKTNTYLD--I-----NDVNFWRKLKSAL----

>Strigamia_11 SMAR005346-PA ProteinLength=1069 DomainCoordinates=934-1060 DomainLength=126 Accession=cl17458 ID=136446

---FISY-ASEDE---QWMVENLLPQLED-------------------------YKLCIHNRDFP-VGE--F-ISDS-----------IMSAVKNSCRTVILLSRHFLKSRWC-QFEFDTAHLQSM-QDK------------CKRLVV-VRLEKMDESE-IDKN--------VS--AYLKTNTYLD--I-----NDAFFWDKLKYAL----

>Strigamia_12 SMAR005007-PA ProteinLength=1137 DomainCoordinates=993-1125 DomainLength=132 Accession=cl17458 ID=136857

-DAFISY-ANEDD---EWVIEELVHRLNE-------------------------YRLCIHQRDFP-VGG--F-IAES-----------IALAVENSCRTVIVLTPNFLQSQWC-RFEFETAHLLSL-ENK------------CKRVVV-IVLEQVKSLE-LDKN--------LR--AYLKTNTYLD--I-----NDANFWRKLKNVLPDLK

>Strigamia_13 SMAR005008-PA ProteinLength=939 DomainCoordinates=797-927 DomainLength=130 Accession=cl17458 ID=136858

-SAFISY-ADEDE---DWMIEELLPRLDD-------------------------YKLCIHQRDFP-FGD--K-I-ES-----------LLQAVESSCRTVVILTPNYLKSDWC-LFEFDTAHMQSL-QDK------------CKRLVV-VLLEAVDKCE-MNKN--------LS--AYLKTNTYLD--I-----NDALVWEKLKFALPNN-

>Strigamia_14 SMAR005010-PA ProteinLength=928 DomainCoordinates=771-906 DomainLength=135 Accession=cl17458 ID=136861

-DAFVSY-SSEDE---DWITDFLVPGLETG--------T---PT-----------YSLCLHNRDWR-AGE--F-ITDQ-----------IVKSVESSRRTIIVLTDNYLKSGWS-RLEFDIAYQQGL-KDK------------VRRVIA-VVPNDVPDLSQIDAN--------FR--TFITLTTYIQ--A-----NKPHFWSKLRASLPR--

>Strigamia_15 SMAR004813-PA ProteinLength=697 DomainCoordinates=544-640 DomainLength=96 Accession=cl17458 ID=137048

---FTIH-SPDAT---DWIESDMIPNLKNL--------EA---------------GRSVAFVEGLP-AGK--D-FIEE-----------LTTAIENSGKIVFLLDNEFLENDWC-KWELSYAHFCII-DSR-----------IRNRMIF-IVLEDL---

>Strigamia_16 SMAR004578-PA ProteinLength=1033 DomainCoordinates=891-1014 DomainLength=123 Accession=cl17458 ID=137341

---FISY-ANEDD---EWVTEELVHRLNE-------------------------YRLCIHQRDFP-VGG--L-IAES-----------
IAFAVENSCRTVIVLTPNFLQSQWC-RFEFETAHLQSL-ENK------------CKRVVV-IVLEQVKSLE-LDKN--------LG--
AYLKTNTYLD--I-----NDVNFWRKLK-------

>Strigamia_17 SMAR004491-PA ProteinLength=805 DomainCoordinates=650-792 DomainLength=142 Accession=cl17458 ID=137430

YDAFISY-NAHDQ---DWMITNLLPKLELE--------EPK--------------FKICLHERDFV-IGK--E-IISN-----------
IVESIDNSRYIVLLLSNNFLASQWC-TWEMNMAQFMSVDGCR-------------DALIL-IMIEPIKRKSMSST---------LK--
YLIKTRTYLE--WTDNAQGQKLFWERLKFAMQRPD

>Strigamia_18 SMAR003805-PA ProteinLength=1074 DomainCoordinates=928-1060 DomainLength=132 Accession=cl17458 ID=138181

-DAFISY-ANEDD---EWVTEELVHRLNA-------------------------YKFCIHQRDFP-VGG--L-IADS-----------
IAHAVQNSCKTVIVLTPNFLQSQWC-QFEFKTAHLQSL-ENK------------CQRVIV-IVLERVKNLE-LDKN--------LR--
AYLKTNTYLD--I-----NDVNFWRKLKSALPDLK

>Strigamia_19 SMAR015400-PA ProteinLength=584 DomainCoordinates=416-547 DomainLength=131 Accession=cl17458 ID=138574

---FVSY-SCEDE---DWIVDFLVPGLETG--------I---PS-----------YKLCLHNRDWR-AGQ--F-ITDQ-----------
IVQSVESSRRTIIVLTDNYLKSNWS-RLEFDIAYQQAL-KDQ------------VRRVIA-VVPNEVPDLSKIDKE--------FK--
SFITLTTYVE--A-----KKPYFWRKLRASM----

>Strigamia_20 SMAR002710-PA ProteinLength=518 DomainCoordinates=406-450 DomainLength=44 Accession=cl17458 ID=139433

-DAFISY-ANEDD---EWVTKELVHRLNE-------------------------YKLCIHQRDFS-VGG--L-NADS-----------IA---------
----

>Strigamia_21 SMAR002611-PA ProteinLength=835 DomainCoordinates=654-789 DomainLength=135 Accession=cl17458 ID=139543

-DAFVSY-SGEDE---DWIRDFLVPGLEAR--------N---PK-----------YNLCLHNRDWL-AGE--F-ITDQ-----------
IVKSVQTSRRTIIVLTDNYLKSPWS-RLEFDLAYQQGL-KDQ------------VRRVMV-IVPNEVPDLSQIDAT--------FK--
TFISLTTYIQ--A-----NKPHFWSKLRASMPR--

>Strigamia_22 SMAR002366-PA ProteinLength=610 DomainCoordinates=459-594 DomainLength=135 Accession=cl17458 ID=139827

-DAFVSY-SSADE---DWITDFLVPGLEAS--------I---PP-----------YKLCLHNRDWI-GGE--F-IIDQ-----------
IIRSVESSRRNIIVLTDNYLKSKWS-RLEFDVAYEQGL-KDQ------------VRRVMI-IVPNEVPDLSQIEPE--------FK--
TFITLTTYIQ--A-----NKPHFWRTLRASMPR--

>Strigamia_23 SMAR002249-PA ProteinLength=317 DomainCoordinates=176-299 DomainLength=123 Accession=cl17458 ID=139946

---FISC-APEDE---EWIIEEPLPRLDK-------------------------HKLCILQRDFP-AGG--Y-IAES-----------
IIYAVENSCRTVIILTPNYLKSQWC-KFEFDTAQMQAQ-KDK------------CKRHVV-VLLEAVEKCE-MNKN--------LG-
-SYLKTNTYLD--I-----NDGLFWEKLK-------

>Strigamia_24 SMAR002076-PA ProteinLength=513 DomainCoordinates=371-497
DomainLength=126 Accession=cl17458 ID=140123

---FISY-ANEDD---EWIAEELVPRLNE-------------------------YRLCIHQRDFP-VGG--L-NAES-----------
VAHAVENSCRTLIVLTPNFLQSQWC-QFEFETAHLQLL-ENK------------CQRVVV-IVLERVKSFE-LDKK--------LC--
AYLRTNIYLD--V-----NDANFWRKLKSAL----

>Strigamia_25 SMAR001938-PA ProteinLength=406 DomainCoordinates=291-372 DomainLength=81
Accession=cl17458 ID=140255

---FISY-ANEDG---HWIAGTLVPQLTD-------------------------YKLCIHQRDFP-VGG--F-IAES-----------IVEAVE------
--------KSQWC-QFEFNTAHMQSL-QDK------------CKRLIV-VLYQEV------DKN--
------

>Strigamia_26 SMAR001266-PA ProteinLength=933 DomainCoordinates=763-898
DomainLength=135 Accession=cl17458 ID=140975

-DAFVSY-SSEDE---DWITDFLVPGMESG--------N---PS-----------YNLCLHNRDWV-GGE--F-ITDQ-----------
IVKSVESSKRTIIVLTDNYLKSKWS-RLEFDVAYQQGL-KDQ------------VRRVMV-IVPNEVPDLSQIDNE--------FK--
TFITLTTYIQ--A-----NKPHFWRKLRASMPR--

>Strigamia_27 SMAR000970-PA ProteinLength=765 DomainCoordinates=604-739
DomainLength=135 Accession=cl17458 ID=141279

-DAFVSY-SGEDE---DWIVDFLVPGLEMG--------I---PS-----------YKLCLHSRDWR-AGE--F-ITDQ-----------
IVKSVESSRRTIIVLTDNYLKSNWS-RLEFDVAYQQAL-KDQ------------VRRVIA-VVPDEVPDLSKIDKE--------FK--
SFITLTTYVE--A-----KKPYFWRKLRASMPR--

>Metaseilus gi_PIPE_391327659_PIPE_ref_PIPE_XP_003738314.1_PIPE_ ProteinLength=1222
DomainCoordinates=1048-1187 DomainLength=139 Accession=cl17458 ID=84737

-DAFISY-CKKDE---PFVAQLLAPELECG--------GST-AP-----------YRLCLRYRDLP-MSG--Y-VAEA-----------
ITEAIECSRRSIVVLSEQFLRSEWC-RFELKAAARDAQIRGT-----------GGSSLVV-IVLDKGAMRL-LDAE--------AR--
LSLRDAPIIH--Y-----EDKRFWEKLRYNLPDA-

>Metaseilus_2 gi_PIPE_391335441_PIPE_ref_PIPE_XP_003742102.1_PIPE_ ProteinLength=1191
DomainCoordinates=1037-1173 DomainLength=136 Accession=cl17458 ID=88526

-DAFVSY-SKKDE---AFVAQMLAPGLECG--------NPP--------------FRLCLHYRDLPMVGG--Y-LSEA-----------
IQEAVESSRRTIVVLSEHFLKSEWC-RYEFKSAHHEVL-NNS------------NHKLVV-IFLGRVSYRE-LDPD--------IR--
MWLKHSTFLH--W-----KEKRFWDKLRYSLPDA-

>Metaseilus_3 gi_PIPE_391337512_PIPE_ref_PIPE_XP_003743111.1_PIPE_ ProteinLength=836
DomainCoordinates=670-828 DomainLength=158 Accession=cl17458 ID=89514

FDAFLSY-HTDDR---EMAME-ILRELESD--------VAVHDALRRERDAGVGPFELAIHERDFV-AGK--E-LTWN--------
---INELVKNSHRSIIIMSEKFLDSRWF-SIEFLAAYGQTL-EDH------------VNRLII-
IIKGDLPSDEGLSERFDRQLCETLK--GVLKNRLYLT--W-----GERWFWEKLLYAMPHG-

>Metaseilus_4 gi_PIPE_391342046_PIPE_ref_PIPE_XP_003745335.1_PIPE_ ProteinLength=993 DomainCoordinates=821-964 DomainLength=143 Accession=cl17458 ID=91743

-DAFLSF-SAKDR---DVAME-IFQRLELDAADNETIAEP---------------FKLCIHERDFM-PGQ--T-ITWN-----------
ILHAVRSSRRTILILSKEFLESTWF-KIEFQATHDQML-DDH------------IDRLIV-VIKGQLPPFESLHEN--------LR--
AVLKTKTYLV--W-----GERWFWKKLLFAMPHKN

>Tetranychus tetur36g00940.1 ProteinLength=1239 DomainCoordinates=958-1070 DomainLength=112 Accession=cl17458 ID=144773

---LCVYDRDFI-AGR--P-ISEC-----------
ITESIRNSRKVILIISNNFAQSPWC-RFETDLAHNTLLDQNR-------------EGLIL-IKLEEMSSEVLEKVA------PQLH--
FLLKTRIYLS--WSDDSSEQEIFWKKLRRALGF--

>Tetranychus_2 tetur09g04990.1 ProteinLength=1272 DomainCoordinates=1109-1243 DomainLength=134 Accession=cl17458 ID=151851

-DGFVSY-CKKDE---AFISQILAPELECG--------HPP--------------YRLCLRYRDLP-VTE--Y-VAEA-----------
ISEAIESSHRTIILLSDQYYKSDSC-HFELKVAHQECQ-VNL------------NHKIIV-VVLDKNSLNH-LDAD--------SK--
LCIRSSPIIH--W-----GDRRFWEKLRYAMPP--

>Ixodes ISCW018193-RA ProteinLength=1086 DomainCoordinates=859-996 DomainLength=137 Accession=cl17458 ID=32126

YDAFVSY-SSADR---DIAMG-LLNSLESN--------EEM--------------FKLCIHERDWL-PGY--N-ISWN-----------
IVNSVQNSRRTILVVSKDFLESVWF-QVEFHTAYYQML-EDR------------VDRLIV-IVRGELPAKETLDKE--------LK--
FLLTTKTYLV--W-----GERWFWEKLKYAMPHRR

>Ixodes_2 ISCW007724-RA ProteinLength=421 DomainCoordinates=263-397 DomainLength=134 Accession=cl17458 ID=44868

-DVFLSF-SSKDS---MWAYEQLIPGVEAH--------G----------------FSVCTYDRNFK-GGF--L-LQDI-----------
IHEAVSCSRRTLLLLTKNFVESEWC-RWEFRVAHHQAL-EDK------------INRLIL-VLVDELAPGL-VDEE--------LQ--
LYMQATNYLR--W-----GEPHFWDKLIYSLPKKD

>Ixodes_3 ISCW022740-RA ProteinLength=1344 DomainCoordinates=1186-1322 DomainLength=136 Accession=cl17458 ID=47585

-DAFVSY-SKKDE---AFVAQILAPELECG--------QPP--------------YRLCLHYRDLPMAGG--Y-LTDA-----------
ITEAVESSRRTIVILSEHFLKSEWC-RYEFKSAHHEVL-HSC------------THRLVV-IFLGRVSYKE-LDPD--------IR--
LWLKSSTFLR--W-----GEKRFWDKLRYAMPDT-

>Ixodes_4 ISCW020989-RA ProteinLength=1226 DomainCoordinates=1052-1188 DomainLength=136 Accession=cl17458 ID=50832

-DAFVSY-CKKDE---AFVAQILAPELECGS-------HPP--------------FRLCLRYRDLP-MSG--Y-VAEA-----------
ITEAVECSHRTLVVLSEQFLKSEWC-RFELKTAHHELR-CNS------------RHRLVV-VLLDDVAVKE-MDAD--------AR-
-QCLRSAVLLR--W-----GDKRFWEKLRYALPDA-

>Mesobuthus MMa46071 ProteinLength=1683 DomainCoordinates=1523-1653 DomainLength=130 Accession=cl17458 ID=51418

---FVSY-TGSDR---EIAFA-ILQELEEK--------FPF--------------FKLCIHERDWL-PGY--P-IISQ-----------
IENSIQNSRKTIIILSEEFLNSVWC-ETEFQVAYLQCL-EDK------------VDRLII-VIKGKMPTSDKMPLD--------MQ--
KLLATKTYIV--W-----GERWFWEKLRYSL----

>Mesobuthus_2 MMa46071 ProteinLength=1683 DomainCoordinates=613-721 DomainLength=108 Accession=cl17458 ID=51418

-DAFLCY-NSADN---DIMME-MMNKLEPP-------------------------YRLCIHERDWL-VGY--P-ISQH-----------
IVSSVLHSRKTVILLSSNFLQSIWF-QVEFRVAFNQMM-EDQ------------RHRLVL-VIIGELGDVHQLDKD--------LR--
-------HLI-------------------------

>Mesobuthus_3 MMa34077 ProteinLength=778 DomainCoordinates=679-734 DomainLength=55 Accession=cl17458 ID=51939

---FVSY-NSVDV---AWVAQELLPNLENH--------HSN--------------FRLCIHDRDFE-VGR--L-ITEN-----------
ILDSIDRSRKH---

>Mesobuthus_4 MMa52435 ProteinLength=1232 DomainCoordinates=1079-1213 DomainLength=134 Accession=cl17458 ID=55803

-DAFLSY-SKKDE---VFVSEILASELENG--------EPP--------------YRLCLHYRDLP-SGG--Y-LADA-----------
VFEAIESSRRIIILLSENFLRTEWS-RYDPKSTQHNML-RTC------------RSKLIL-IFCG-TSPRD-IDPE--------LK--
DCCKSGTLLH--W-----GEKRFWEKLRYAMPDV-

>Mesobuthus_5 MMa37636 ProteinLength=1192 DomainCoordinates=1036-1171 DomainLength=135 Accession=cl17458 ID=59190

-DAFVSY-CKKDE---AFIAQILAPELECG--------NPP--------------YRLCLRYRDLP-SAG--Y-VAEA-----------
ISEAIECSHRTIVVLSEQFLKSEWC-RFELKTAHRESQ-RNP------------KHRLIV-TLLDRISFKE-MDPD--------AK--
MCFHSSPVVR--W-----GDKRFWEKLRYSLPDG-

>Mesobuthus_6 MMa37517 ProteinLength=737 DomainCoordinates=583-716 DomainLength=133 Accession=cl17458 ID=59533

-DAFICY-NSADR---EVMMD-ILEHLEPT-------------------------YRLCVHERDWI-PGY--P-ICHQ-----------
IVKSVQDSRKTVILLSKDFLDSLWF-QVEFRTAYNQMM-EDK------------KHRLVL-VIIGKLDNLNNLEKD--------LR--
HLLSTKTYLQ--W-----GEKWFWEKFRYTLRHRH

>Mesobuthus_7 MMa02436 ProteinLength=634 DomainCoordinates=484-626 DomainLength=142 Accession=cl17458 ID=60875

YDAFISY-NANDS---EWVFNILLPHLESN--------SSD--------------LKLCVYDRDFL-AGS--G-ISEC-----------
IMDSIKCSRRTILILSNSFLQSPWC-KFETDLAHHVLIDEER-------------EGLLL-IKLEELSEHLLSPQ---------LN--
YLLKTKIYLE--WSNKPKEQDLFWKKLRRALQSKK

>Mesobuthus_8 MMa51810 ProteinLength=789 DomainCoordinates=643-776 DomainLength=133 Accession=cl17458 ID=62594

---FVSY-DSLNC---DWIVNQLLPTLETD--------E----------------MHFCLHDRDCP-AGN--D-VTEE-----------
VLEYVQKCRKIMLVISESYIKNQWC-MFELQMAQHRLFEERR-------------DSLIL-IHLGSIQELQLPSN---------LK--
YLMNTRTYIP--WTDHPMGQKLFWKRLRKAL----

>Mesobuthus_9 MMa42574 ProteinLength=507 DomainCoordinates=354-490 DomainLength=136 Accession=cl17458 ID=65093

-DAFISY-STSDR---DIVMC-LIEELEEK--------EPN--------------FCLCIHERDWI-PGN--L-ICSN-----------
IANSVSNSRRTIIILSEQFLNSIWF-PVELHSAYYKML-EDK------------VNRIIF-ILRGQLPPMNTLDKD--------LQ--
VLLKTKTYLV--W-----GERWFWEKLRLALPHKN

>Mesobuthus_10 MMa11502 ProteinLength=1027 DomainCoordinates=810-946 DomainLength=136 Accession=cl17458 ID=65244

-DAFVSY-SSNDR---DVMLT-LLNELENK--------EPS--------------FKLCIHERDWM-PGN--L-ISLN-----------
IVNSVQYSIRTILVLTEDFLESVWF-QVEFQTAYHQML-EDK------------INRLIV-IVKGQLPPKETLDKN--------LQ--
AILSTKTYLV--W-----GERWFWEKLRYAMPHKK

>Mesobuthus_11 MMa34079 ProteinLength=817 DomainCoordinates=679-811 DomainLength=132 Accession=cl17458 ID=67517

---FVSY-NSVDV---AWVAQELLPNLENH--------HSN--------------FRLCIHDRDFE-VGR--L-ITEN-----------
ILDSIDRSRKVILVLTESFVKSEWC-LFELHVAQHQLFYNAR-------------DALIL-IQVGPIDKNCLSKN---------LK--
YLMKTRSLVI--WPDDPDKKNEFWDRLH-------

>Mesobuthus_12 MMa22782 ProteinLength=874 DomainCoordinates=710-846 DomainLength=136 Accession=cl17458 ID=71375

YDAFVSY-SMTDR---DVVLC-LVRELEEK--------EPK--------------FSLCIHERDWI-PGN--P-ICLN-----------
IANSVLCSKRTIIILSNEFLKSIWF-PVELHSAYYQML-EDK------------VNHLII-IVRGKLPPVHTLDRD--------LQ--
VLLKTKTYLI--W-----GERWFWEKLRLALPHN-

>Mesobuthus_13 MMa07778 ProteinLength=1108 DomainCoordinates=945-1081 DomainLength=136 Accession=cl17458 ID=71550

-DAFVSY-CKKDE---AFVAQILAPELECG--------HQP--------------YRLCLRYRDLP-MVG--Y-VADA-----------
ISEAIECSHRTIAVVSEQFLKNEWG-RFELKTAYHESQCNDR------------RHRLVV-VLLDKISFKE-LDPD--------AR--
LCLRSSIVVH--W-----GDKRFWEKLRYAIPDN-

>Mesobuthus_14 MMa13223 ProteinLength=1193 DomainCoordinates=1032-1155 DomainLength=123 Accession=cl17458 ID=79149

-DAFVSY-SKKDE---AFIAQILAPELECG--------NPP--------------YRLCLHYRDLS-VGG--Y-LSDA-----------IAEAMENSRRTILVLSENFLKSEWC-RYEFKTAHHEVL-SSR------------KHSLIV-IVLGRIAYRD-LEPD--------VK--MWLKTSVFLR--W-----ALET-------------

>Mesobuthus_15 MMa40477 ProteinLength=970 DomainCoordinates=815-951 DomainLength=136 Accession=cl17458 ID=80132

FDAFVSY-SSLDE---SFVLFKLVPTLESG--------E---KP-----------YYLCLHSRNFV-PGS--Y-IQDN-----------IINAVKASKRTILVLSENFLSSEWC-RLEFKSAHHQVL-EER------------LNRLIV-IVLGDLPAGDDIDPE--------LL--LYLKTTTYLR--W-----GEKNFWNKLRYAMPK--

>Parasteatoda aug3.g494.t1 ProteinLength=1236 DomainCoordinates=1070-1204 DomainLength=134 Accession=cl17458 ID=95710

-DAFVSY-CKKDE---AFVTQMVAPELECG--------VPS--------------QRLCLRYRDLP-ASR--Y-MAET-----------ISEAVECSSCSIAVISEQYLKSEWC-LFELKACHHESQ-CNR------------RHKIII-ILLNKVDFKE-LDGD--------VR--ACFKAAVIIH--W-----GDRRFWEKLRFSLPE--

>Parasteatoda_2 aug3.g1692.t1 ProteinLength=1166 DomainCoordinates=1023-1158 DomainLength=135 Accession=cl17458 ID=97154

-DAFVSY-CNKDE---TFVLQFLTPELECG--------EPS--------------YRLCLRYRDLP-LSE--Y-VAEA-----------VTEAIECSQRTIIVLSEHFLRNERC-RFELKTAYRESQ-CNH------------KHKLLI-VVVGKISFKS-LDSD--------AK--QCLSNAHIIH--W-----GDKRFWQKVKFIMPNV-

>Parasteatoda_3 aug3.g2549.t1 ProteinLength=1239 DomainCoordinates=1058-1194 DomainLength=136 Accession=cl17458 ID=98154

-DAFVSY-SKKDE---AFVTQILAPELECG--------MPN--------------YRLCLHYRDLP-VSG--YIMSEA-----------IMEAMESSRRTILILSENFLKSEWC-RFEFKSAHREVL-SAC------------QHRLLV-IELGKVDPQE-LDPD--------IR--LWLRQCPVVR--W-----GEKQFWDRLKYAMPDV-

>Parasteatoda_4 aug3.g6063.t1 ProteinLength=1202 DomainCoordinates=1037-1171 DomainLength=134 Accession=cl17458 ID=102307

-DAFLSY-CKKDE---AFVSQMLAPELEDG--------TYP--------------HRLCLRYRDLP-MAE--Y-VAEA-----------ISEAIECSRCTVAVISEQYLKSEWC-IYELKAAHHETQ-INR------------RHRVIV-VLLDKISFKQ-LEPD--------VR--ICLRSATLVH--W-----GDRRFWEKLRFAMPE--

>Parasteatoda_5 aug3.g11043.t2 ProteinLength=1559 DomainCoordinates=1396-1535 DomainLength=139 Accession=cl17458 ID=108129

-DAYISF-SDEDA---EMVRHHFLPELEEK--------HPF--------------YKLFVPQRDLK-AGN--F-EINN-----------LMEQVLDSKRAIILLSRHYLKNEFC-MEIFRVAFANSLEEKL-------------HRVIL-VKFGPLPPMNHEPST--------HEPSTMMESSRSLK--F-----GTRLFWDMLQYEMPEKR

>Parasteatoda_6 aug3.g12127.t1 ProteinLength=409 DomainCoordinates=314-386 DomainLength=72 Accession=cl17458 ID=109396

--YLRNEFC-MEIFRVAFANSLEKKL-------------HRIIL-VKFGPLPPQKEMDQS--------LK--LVMEFSRCLK--F-----RQKLFWEMLRYEMPEKS

>Parasteatoda_7 aug3.g12128.t3 ProteinLength=620 DomainCoordinates=459-596 DomainLength=137 Accession=cl17458 ID=109399

-DAYISF-SDVDA---SDVRKHFLPVLELK--------HPF--------------YKLFVPQRDIK-TGN--F-EINN-----------LMRRMVDCKRTVVLLTKNYLENEYC-MGIFRMAFANSLEEKL-------------HRIIP-VKFGPLPPLKEMDQS--------LK--TVMESTIWLE--F-----GDKLFWDMLRYEMSEKS

>Parasteatoda_8 aug3.g17600.t3 ProteinLength=1889 DomainCoordinates=784-915 DomainLength=131 Accession=cl17458 ID=115752

---FISY-SDKDF---DAVQN-LIRTIEAK--------QPM--------------SRLHFHFRDFL-GGA--P-IEQN-----------IIHAVQNSKRTIVVLSKNYQESEWC-IFEFQRARSQTL-KDK------------VNRILI-IRMGELP--DNLDDN--------IK--AHLKSTTYLI--W-----GEKFFWEKLFYALPTS-

>Parasteatoda_9 aug3.g17600.t3 ProteinLength=1889 DomainCoordinates=1732-1866 DomainLength=134 Accession=cl17458 ID=115752

-DAFLSF-SHKDQ---ELVVTDIISVIEVK--------QPM--------------TRLCLHYKHFK-AGD--F-IDQN-----------IFNAVQNSKRTVIMLSKNFLESEWC-IYEFRAAHLQAL-KDK------------INRVII-IKLGELP--DDLHPD--------IK--MSLENTTYLT--W-----GEKYFWDKLFYVLPTS-

>Parasteatoda_10 aug3.g18079.t1 ProteinLength=614 DomainCoordinates=480-613 DomainLength=133 Accession=cl17458 ID=116285

-DAFVAY-SSSDK---RLVMN-LLNELEQN--------APF--------------FKLCIHERDWI-PGE--I-ITET-----------IVKSVRSSRRTIIVLSESFISSPWF-KVELKVAISQ----EQ------------MNPIIV-IMVDKSISLNELNRE--------LR--DAISKRTYLE--W-----GERWFWEKLRYAMPHKV

>Parasteatoda_11 aug3.g18263.t1 ProteinLength=850 DomainCoordinates=689-826 DomainLength=137 Accession=cl17458 ID=116494

-DAYISL-SDEDA---SEARKHFLPVLELK--------HPF--------------YKLFVPQRDIK-TGN--F-EIDN-----------LMRRMLDCKRTVFLLTKNYLENEFC-MEIFRVAFENSLEEKW-------------HRIIL-VKYGPLPPLKEMDQS--------LK--TVMVSSRCLK--F-----GDKLFWDMLRYEMSEKR

>Parasteatoda_12 aug3.g19333.t1 ProteinLength=1037 DomainCoordinates=845-981 DomainLength=136 Accession=cl17458 ID=117686

-DAFVSY-SCSDR---DVAME-LIEELEKK--------DPR--------------FNLCIHERNWI-AGN--Q-ISWN-----------IFNSVHNSKRTILVISKAFLESMWF-QVEFHTAYYQML-EDK------------IDRLII-IVKGDLPPKENMDKD--------LQ--YLLSTKTYLI--W-----EEKWFWEKLKYAMPHKK

>Parasteatoda_13 aug3.g20004.t2 ProteinLength=988 DomainCoordinates=888-973 DomainLength=85 Accession=cl17458 ID=118423

--ADCCKVIVILSSNFLRNSQC-MQLMKTAMACSIEKTA-------------QRFIF-IITDSIPPELKLDAV--------LN--CLVKRSTLIK--W-----GECLFWTKLKFSLPKKS

>Parasteatoda_14 aug3.g20240.t1 ProteinLength=1951 DomainCoordinates=1457-1570 DomainLength=113 Accession=cl17458 ID=118683

-DAFISF-SHNDQ---DLVITDIISVLEVK--------QPM--------------TRLCLHYKHFI-AGE--Y-IDAN-----------IFTAVQNSKRTVIVLSKNFLESEWC-VYEFRAAHMQAL-KDK------------VNRVII-IKLGELP--DDAHPD--------IK--LYLKKD-----------------------------

>Parasteatoda_15 aug3.g20240.t1 ProteinLength=1951 DomainCoordinates=355-485 DomainLength=130 Accession=cl17458 ID=118683

-DAFLSF-SHKDQ---DLVIMDIISVIEVK--------QPM--------------TRLCLHYKHFK-AGD--F-IDQN-----------IFNAVQNSKRTVIMLSKNFLESEWC-IYEFRAAHLQAL-KDK------------INRVII-IKLGELP--DDLHPD--------IK--MSLENTTYLT--W-----DFDLEDDNIIKK-----

>Parasteatoda_16 aug3.g20941.t1 ProteinLength=976 DomainCoordinates=814-956 DomainLength=142 Accession=cl17458 ID=119431

YDAFVSY-NSSDT---PWIVSFLIPALENQ--------DPK--------------LKLCIHDRDFK-VGW--L-ITDN-----------ILDAIENSRKVILILTEEFVKSEWC-MFELHMAQHRLFDETR-------------DSLIL-IKLKQVDKKFYTKN---------LK--YLEKTRTCLL--WPDNLPDQKLFWLKVRKLLGHPH

>Parasteatoda_17 aug3.g25912.t1 ProteinLength=1202 DomainCoordinates=1047-1183 DomainLength=136 Accession=cl17458 ID=124776

-DAFISY-CKKDE---AFVVQVLANELEYG--------SPH--------------FRLCLHYRDLP-IGG--Y-LSEA-----------IIEAIESSRRTILILSENFVRSEWF-RYEFKSAHYEMV-RTC------------KQKLIV-IFVGCLSQED-LDPD--------LR--LWLKSSTFLH--W-----DDKKFWEKLRFALPDIR

PGRP Domain Alignment, for Figure 4

>Drosophila FBgn0030310 ProteinLength=203 DomainCoordinates=60-186 DomainLength=126 Accession=cd06583 ID=0

------------------IRYVVIHHTVTG-E--CSGLLKCAEILQNMQAYHQNE--------------------------
LDFNDISYNFLIGNDGIVYEGTGWGLRGAHTYG--YN-AIGTGIAFIGNFVDK-
LPSDAALQAAKDLLACGVQQGELSEDYALIAGSQVI--STQSPG--L

>Drosophila_2 FBgn0030695 ProteinLength=345 DomainCoordinates=198-326 DomainLength=128 Accession=cd06583 ID=1

------------------VKYVVILHTATE-S--SEKRAINVRLIRDMQCFHIES--------------------------
RGWNDIAYNFLVGCDGNIYEGRGWKTVGAHTLG--YN-RISLGISFIGCFMKE-
LPTADALNMCRNLLARGVEDGHISTDYRLICHCQCN--STESPGRRL

>Drosophila_3 FBgn0043577 ProteinLength=191 DomainCoordinates=40-124 DomainLength=84 Accession=cd06583 ID=2

------------------VRLIIIHHTVTA-P--CFNPHQCQLVLRQIRADHMR---------------------------
RKFRDIGYNFLIGGDGRIYEGLGFGIRGEHAPR--YN-SQSIGIAFIGNFQNA---
PG---

>Drosophila_4 FBgn0043578 ProteinLength=190 DomainCoordinates=48-176 DomainLength=128 Accession=cd06583 ID=3

-------------------DYVIIHHSDNPNG--CSTSEQCKRMIKNIQSDHKGR--------------------------
RNFSDIGYNFIVAGDGKVYEGRGFGLQGSHSPN--YN-RKSIGIVFIGNFERS-
APSAQMLQNAKDLIELAKQRGYLKDNYTLFGHRQTK--ATSCPGDAL

>Drosophila_5 FBgn0035975 ProteinLength=368 DomainCoordinates=205-331 DomainLength=126 Accession=cd06583 ID=4

------------------IPYVLITHIGVQ-SLPCDNIYKCSIKMRTIQDSAIAE--------------------------
KGLPDIQSNFYVSEEGNIYVGRGWDWANT------YA-NQTLAITFMGDYGRF-
KPGPKQLEGVQFLLAHAVANRNIDVDYKLVAQNQTK--VTRSPGAYV

>Drosophila_6 FBgn0035977_DOM1 ProteinLength=369 DomainCoordinates=80-208 DomainLength=128 Accession=cd06583 ID=5

------------------VSNIIIHHTATE-G--CEQEDVCIYRMKTIQAFHMKS--------------------------
FGWVDIGYNFLVGGDGQIYVGRGWHIQGQHVNG--YG-AISVSIAFIGTFVNM-
EPPARQIEAAKRLMDEGVRLHRLQPDYHIYAHRQLS--PTESPGQKL

>Drosophila_7 FBgn0035977_DOM2 ProteinLength=369 DomainCoordinates=266-363 DomainLength=97 Accession=cd06583 ID=5

---------------------------NTP-S--CFTQAECTFRVRLLQNWHIES--------------------------
NGYKDINYNFVAAGDENIYEARGWDHSCE--PP--KD-ADELVVAFIG-------
PSSSNKKIALELIKQGIKLGHISKNYSLID---------------

>Drosophila_8 FBgn0035806 ProteinLength=186 DomainCoordinates=48-171 DomainLength=123 Accession=cd06583 ID=6

-----------------------IAHTAGG-A--CADDVTCSQHMQNLQNFQMSK--------------------------
QKFSDIGYHYLIGGNGKVYEGRSPSQRGAFAGP--NN-DGSLGIAFIGNFEER-
APNKEALDAAKELLEQAVKQAQLVEGYKLLGHRQVS--ATKSPGEAL

>Drosophila_9 FBgn0037906 ProteinLength=255 DomainCoordinates=75-204 DomainLength=129 Accession=cd06583 ID=7

------------------APYVIIHHSYMPAV--CYSTPDCMKSMRDMQDFHQLE--------------------------
RGWNDIGYSFGIGGDGMIYTGRGFNVIGAHAPK--YN-DKSVGIVLIGDWRTE-
LPPKQMLDAAKNLIAFGVFKGYIDPAYKLLGHRQVR--DTECPGGRL

>Drosophila_10 FBgn0043576 ProteinLength=185 DomainCoordinates=46-170 DomainLength=124 Accession=cd06583 ID=8

--------------------YAIIHHTAGS-Y--CETRAQCNAVLQSVQNYHMDS--------------------------
LGWPDIGYNFLIGGDGNVYEGRGWNNMGAHAAE--WN-PYSIGISFLGNYNWD-
TLEPNMISAAQQLLNDAVNRGQLSSGYILYGHRQVS--ATECPGT--

>Drosophila_11 FBgn0033327 ProteinLength=185 DomainCoordinates=46-170 DomainLength=124 Accession=cd06583 ID=9

--------------------YAIIHHTAGS-Y--CETRAQCNAVLQSVQNYHMDS--------------------------
LGWPDIGYNFLIGGDGNVYEGRGWNNMGAHAAE--WN-PYSIGISFLGNYNWD-
TLEPNMISAAQQLLNDAVNRGQLSSGYILYGHRQVS--ATECPGT--

>Drosophila_12 FBgn0043575 ProteinLength=184 DomainCoordinates=45-169 DomainLength=124 Accession=cd06583 ID=10

--------------------YAVIHHTAGN-Y--CSTKAACITQLQNIQAYHMDS--------------------------
LGWADIGYNFLIGGDGNVYEGRGWNVMGAHATN--WN-SKSIGISFLGNYNTN-
TLTSAQITAAKGLLSDAVSRGQIVSGYILYGHRQVG--STECPGT--

>Drosophila_13 FBgn0035976 ProteinLength=520 DomainCoordinates=376-500 DomainLength=124 Accession=cd06583 ID=11

------------------VGLVIALPTNSE-N--CSTQAICVLRVRLLQTYDIES--------------------------
SQKCDIAYNFLIGGDGNVYVGRGWNKMGAHMNNINYD-SQSLSFAYIGSFKTI-
QPSAKQLSVTRLLLERGVKLGKIAPSYRFTASSKLM--------PSV

>Drosophila_14 FBgn0260458 ProteinLength=327 DomainCoordinates=194-301 DomainLength=107 Accession=cd06583 ID=12

-------------------GTVIFTHTGSN-E--CHD--DCPDVLHKLERSHV-------------------------------
GELPYNFLVAGDCQVFEAQGWHYRSQYPRD--LNGIDSLVMAFVGNFSGR-
PPIDCQLMAAQALILESLKRRILQPIYQLFV----------------

>Strigamia SMAR001446-PA ProteinLength=370 DomainCoordinates=56-167 DomainLength=111 Accession=cl02712 ID=140796

------------------------HMTTKD-D--CTKPMLCKEEIKEMEKYFM----------------------------
QNRTGTWLQFYIDSNGIIHKR--FMRSDSKGNS--SS-LYNISITLLGNYTVG-
PIPDKMMYIIRELLKCFIPSIQLTHFIKPRSLN---------YGQSF

>Strigamia_2 SMAR007557-PA ProteinLength=202 DomainCoordinates=49-174 DomainLength=125 Accession=cl02712 ID=133917

--------------------NVFIHHSKEK-T--CWNLEECSERARLLEYRHIKL--------------------------
YRFLDFGYNFMIGGDGRVYEGRGWKRFGAHTTG--FN--YDIGILFLGNFDDY-
RPPKRMIETAKSLLECGRERGVLQPGYSVYGHRDVG--CTSCPGRYL

>Strigamia_3 SMAR007558-PA ProteinLength=191 DomainCoordinates=47-148 DomainLength=101 Accession=cl02712 ID=133920

----------------------FFTQTGGP-T--CH-IYNCMDKVINLRALDIS---------------------------
NNFTDIRDNFLIGGDGIVYEGLGWKAKNLRYNH--YK--NDLSVAFIGDFENK-
TLPEGMQLAAKNLVLCGAQSRHIDY----------------------

>Strigamia_4 SMAR007559-PA ProteinLength=191 DomainCoordinates=46-169 DomainLength=123 Accession=cl02712 ID=133923

----------------------FIHHTSTA-E--CFNIVACSQLMKEMQKRDVES--------------------------
GVWTDIRFNFVIGGDGHVYEGTGWEGQGAHTKG--YE--NDLGIALIGNYNGE-
EPVDGMLELTEALITHGVLSGKIKRNFGLYAHSDVN--CVSCPGGAL

>Strigamia_5 SMAR007560-PA ProteinLength=176 DomainCoordinates=22-149 DomainLength=127 Accession=cl02712 ID=133924

------------------AARIFIHQTATS-P--CRNLDHCKARVRQIQENDAIV--------------------------
LNHPDITFHFLIGGDGYVYEGLGWDTAGDHTSH--YP--FALGIAFIGQFERN-
RPSEIAWRALNNLIQCGTHHGKLQNLYSVHSHKDVS--CTSCPGEAL

>Strigamia_6 SMAR007561-PA ProteinLength=266 DomainCoordinates=118-245 DomainLength=127 Accession=cl02712 ID=133925

------------------VNFVVIHHTRGD-N--CSTNTECKQRVREIQRFDMDV--------------------------
LEKKDIMMHFLIGGDNRVYEGRGWGVVEEDIPE--HP--FALSIGFIGDYDTM-
NVPNAMLYSAQKLIQCGVKKKMIFKQHRVYGHRDVR--CTTCPGDKL

>Strigamia_7 SMAR007562-PA ProteinLength=194 DomainCoordinates=2-124 DomainLength=122 Accession=cl02712 ID=133927

-----------------------VHHTSGG-S--CKTTEACKKLIKKIETYEMTE--------------------------
QHAKEILSHFFIGGDGQIYEGRGWYYPDEYIPG--KG--LSLSISFIGDYNKH-
DAPANMLEAFDKFAQCFMKKVSGYCNHIFSVHSDHR--CTDCPGSHL

>Strigamia_8 SMAR007563-PA ProteinLength=399 DomainCoordinates=2-150 DomainLength=148 Accession=cl02712 ID=133928

-----------------------IHHTAGA-S--
CTTTDECKKLIKDIETYEIKILEDDVMMTMSTFGSLRTKFSNVLAKPHNTKEILSHFFIGGDGEIYEGRGWYYPDDHIPG--YG--LVVSVSFIGDYRTK-DAPKNMLTALDKFIKCFMKKIAGYCQHVFSVHNDHR--CTDCPGRHL

>Strigamia_9 SMAR007564-PA ProteinLength=175 DomainCoordinates=61-152 DomainLength=91 Accession=cl02712 ID=133929

-----------------------VHHTAGA-S--CTTTDECKKLIKDIETFEMTV--------------------------
QHANEILSHFFIGGDGNIYEGRGWYYADEYIPG--HG--LFLSVSFIGDYRIK-NAPANMLAAFDKLQ---------------------------------

>Strigamia_10 SMAR007565-PA ProteinLength=189 DomainCoordinates=47-173 DomainLength=126 Accession=cl02712 ID=133930

-------------------ILVVIHHTNGK-H--CKTKEECKRIIKDIEKFDIEL--------------------------
MNKRDILMHFFIGGENMVFEGRGWGVEAEVIPD--TSASLMINIAFIGNFTSE-
TVPTKALHLAEKLIQCGIQRKQISKQYKVYGHNDLA--CTDCPGK--

>Strigamia_11 SMAR007566-PA ProteinLength=219 DomainCoordinates=35-153 DomainLength=118 Accession=cl02712 ID=133931

--------------------WLMVHHTAGA-S--CNKPDECKKLIKAIETLEMTQ--------------------------
LDAKEILSHFFIGGDGSIYEGRGWYYADEYIPG--HG--LFMSISFIGDYRNK-DAPEIMLTAFHKFV-------
AGYCQHVFSAHNDHR--CTDCPGKHL

>Strigamia_12 SMAR009813-PA ProteinLength=197 DomainCoordinates=48-176 DomainLength=128 Accession=cl02712 ID=131343

------------------VPYAFIHHTAMT-E--CNDFESCCAEMRIIQNFHMDD--------------------------
RGWDDIGYSFLVGGDGRVYIGRDWGVVGAHTYR--YN-TVGYGISFMGTFTNK-
LPNENAMEAVKKLIGCGVVQKYLQSDYGLYGHRDGR--CTECPGDKF

>Strigamia_13 SMAR010214-PA ProteinLength=197 DomainCoordinates=48-176 DomainLength=128 Accession=cl02712 ID=130871

------------------ADKIFFSHTVTK-T--CSDRPSCEEEMRNIQDYHVNT--------------------------
LGFDDIGYNFVIGGDGNIYEGRGWDRIGAHTKG--QN-TGSLGIAFVGEYSKK-
DPTAEMLKAARDLIVCGVLSDKLSRDYGLYGHRDGT--CTASPGNHF

>Strigamia_14 SMAR013411-PA ProteinLength=200 DomainCoordinates=49-175 DomainLength=126 Accession=cl02712 ID=133918

-------------------KFVFIHHTESE-T--CSTQNTCEEYLRNLQLYDTNI--------------------------
LGRKDVGMNFLIGGDGRIYEGRGWNTVGDHTPG--YP--QSIGISFIGNYDDF-
LPTSSMIKAAKNLLNCGVKKGTLDFEHSVNGHRDAS--CSHCPGNAL

>Strigamia_15 SMAR014273-PA ProteinLength=242 DomainCoordinates=93-221 DomainLength=128 Accession=cl02712 ID=130872

-----------------ADKIFFSHTVTK-T--CSDRPSCEEEMRNIQDYHVNT------------------------
LGFDDVGYNFVIGGDGNIYEGRGWDRIGAHTKG--RN-TGSLGIAFVGEYSKK-
DPTAEMLKAARDLIVCGVLSDKLSRDYGLYGHRDGT--CTTSPGNHF

>Strigamia_16 SMAR014565-PA ProteinLength=199 DomainCoordinates=50-176 DomainLength=126 Accession=cl02712 ID=133926

-----------------VIAVVIHHTATS-T--CKTEEECKKIIKDIEKFDLEI------------------------
MKKRDILMHFFIGGENKTFEGRGWGVEDEAIPG--YS-STAINIAFIGNFTSE-
PVPTKALYIAQNLILCGIKMKQISKAHKVYGHNDLR--CTDCPGK--

>Strigamia_17 SMAR014870-PA ProteinLength=181 DomainCoordinates=37-150 DomainLength=113 Accession=cl02712 ID=133919

-----------------AGRVFFSQTGGS-S--CLDHYACYNRLGF--SYTATS------------------------KKYWD---
NFFIGGNGKVYTGTGWEGKNLRYNG--FE--YDLSIVFIGDFSDK-
PLPKIMADTAKQLIECGKDEDYIQHYYSIYTANDFE----------

>Strigamia_18 SMAR014909-PA ProteinLength=196 DomainCoordinates=48-172 DomainLength=124 Accession=cl02712 ID=133922

---------------------FVHHTNTR-E--CITDDECMSSVKEFEAFHREI------------------------
LGDEDLDVHFLIGGNGLIYEGLGWDAQGLHTPE--FP--NDFGVAFIGTFNDS-
PANELMMNAFEDLIECGIENGKIDVFHSLYGHKDARC-STMCPGAHL

>Strigamia_19 SMAR015150-PA ProteinLength=281 DomainCoordinates=61-141 DomainLength=80 Accession=cl02712 ID=140795

----------------------HMTAKH-D--CTKPLVCKEEIKQMEKYIT--------------------------
QNWTGTWLQFYIDKNGNIHKG--FLRSNSLVNY--RS-SYNVSITLLGNYANR-TIPEKMM----------------------------
----

>Strigamia_20 SMAR015602-PA ProteinLength=178 DomainCoordinates=54-171 DomainLength=117 Accession=cl02712 ID=133921

-----------------PGRIFVHHTNTE-E--CKTTTDCIKLVRGEEKFHKEL------------------------
LGQGDIDVQFLIGGDGKVYEGRGWDATGPHTLG--YP--NDFGVAFIGTFNKN-
FASVKMMNAFRELIICGIQHGAIKKDHSIYGHRDA-----------

>Metaseiulus gi_PIPE_391339223_PIPE_ref_PIPE_XP_003743951.1_PIPE_ ProteinLength=193 DomainCoordinates=50-178 DomainLength=128 Accession=cl02712 ID=90387

-----------------VSNVILHHSLGP-T--CITEPTCRSIVRTTQLQHIKI------------------------
KGWDDIGYNFLVSENGQVFEGRGWGVEAAAVMG--LT-DRAVHIAIIGSFNHR-
TPADAAMVAVSRLIQCGMGLGKVHEDYKISAHRDVE--PTACPGHKL

>Tetranychus tetur14g01760.1 ProteinLength=206 DomainCoordinates=86-201 DomainLength=115 Accession=cl02712 ID=157668

------------------VDKIFIDYTYSE-R--CSSLYLCSFNLKSTQEYHKKQ--------------------------
IGLFDIAYNFMIGDDSKVYIGRDFMVARAFDDD--YN-
NNSLLIGIIGDYENPYGPLPRSLDIIYKLIRCGQEKGIITDNITIYD----------------

>Ixodes ISCW024689-RA ProteinLength=131 DomainCoordinates=24-126 DomainLength=102 Accession=cl02712 ID=34241

-------------------GLVFYHHTEGN-E--CFSPETCSSIVRHWPGYHQKS--------------------------
KGWFDIGYQYLIGGDGSIYEGRGFGAIGAHTLR--YN-DKSVSIAFIGNFTYK-VPRQEMLTSAQRLIDCGVEL---------
----

>Ixodes_2 ISCW004389-RA ProteinLength=199 DomainCoordinates=58-185 DomainLength=127 Accession=cl02712 ID=36262

-------------------TIVFYHHTEGL-E--CNSVETCAKIIRQWQDYHMDT--------------------------
KRWDDIAYNFVIGGDGRVYEGRGFDGIGAHTLS--YN-SKSVSLGFVGNFTFN-
VPNSKMLAAAGVLIECGVKSGKIQAKYSLHGQRDAN--LRDCPGEAF

>Ixodes_3 ISCW024175-RA ProteinLength=105 DomainCoordinates=1-93 DomainLength=92 Accession=cl02712 ID=45968

WDDIGYNFMIGGNGMVLEGRGWNHVGAHTVG--FN-NKSVSLGFVGDYSRQ-
VPNERMIMAAMQLIECGIRLKKISPGYTLHGQTDAN--CRRCPGEAF

>Ixodes_4 ISCW022212-RA ProteinLength=197 DomainCoordinates=49-177 DomainLength=128 Accession=cl02712 ID=50914

------------------VPYVFIHHTTGS-G--CDSKVSCSRSIRGHQNYHMDK--------------------------
NGWSDIGYSFLVGGDGRVYEGRGWGTVGAHTRG--YN-SNGIAISFVGNFMAQ-
NPNQAMLNAAQKLIACGIKMGKISSTHSLHGHRDAN--CTACPGNML

>Parasteatoda aug3.g1176.t1 ProteinLength=407 DomainCoordinates=258-384 DomainLength=126 Accession=cl02712 ID=96542

------------------VTHVFIHHTAGA-T--CNSKDTCSKLVRQVQNYHMDT--------------------------
NKWADIGYSFLVGGDGRIYEGRGWKAVGAHTYN--FN-SKAIGIAFMGNFDEK-
EPGSAMLNAARSLIDCGVQKRFITANHEIHGHRDAK--CTTCPGT--

>Parasteatoda_2 aug3.g20166.t1 ProteinLength=204 DomainCoordinates=55-183 DomainLength=128 Accession=cl02712 ID=118602

------------------QKHIIISHTVTP-Q--CHSKTQCANRMRSMQEYHMHQ--------------------------
LGWPNIGYNFVIGGDGRVYEGTGWTKEGIHTYG--WN-PKSYGISFIGDYRFQ-
KPNNAMIKAAHSLTICGIKKGYISKSRELHGARDAT--CTESPGNAL

>Parasteatoda_3 aug3.g20281.t1 ProteinLength=200 DomainCoordinates=51-177 DomainLength=126 Accession=cl02712 ID=118726

------------------VNHVIILHTDTT-F--CMKDWHCVREVQTMQNYWLDE-------------------------
KGLWDLGYNFLISGNGRVYEVRGWNLTGAHVPN--YN-TNSYGIAFIGDFEKD-
IPSKLMLNSALQLIDCGVKRGYLTPAREIHGHRDCI--CTESPGK--

>Parasteatoda_4 aug3.g21135.t1 ProteinLength=263 DomainCoordinates=57-185 DomainLength=128 Accession=cl02712 ID=119633

------------------VNHVIIMHTATK-Y--CRTEVLCEREVRAMQDYHLDT-------------------------
KGFFDLAYNFLVGGDGRVYEARGWNIIESAAPN--YN-FNSHGIAFIGNFSLN-
IPSKSMLDVALRLIDCGVQKGYITPGRAIHGHRDCR--CTDSPGENL

>Parasteatoda_5 aug3.g21165.t1 ProteinLength=182 DomainCoordinates=23-154 DomainLength=131 Accession=cl02712 ID=119668

------------------VSLLFIHRTGGEHE--YDDEKSSIRKMKRLQIIDMDT-------------------------
KGHDDIGYNFVIGGDARVYVGRGWDKIGKHTFG--YN-HISLGVAFMGNFMEE-
KSSIPMIEAGKSLVEYAVSEGYLARDYKLYGHRVVSPGTIECPGNKL

>Parasteatoda_6 aug3.g21166.t1 ProteinLength=285 DomainCoordinates=48-133 DomainLength=85 Accession=cl02712 ID=119669

SDWGAKEPTGVLQCLPGPVEYIIIHHTVTP-S--CTSFTECSKSMRTIQLEHQKD-------------------------N--------------
--NPSMLDLIPKIAWCGVEKGYLRSDFKVIAHRDA------------

>Parasteatoda_7 aug3.g1177.t1 ProteinLength=192 DomainCoordinates=43-171 DomainLength=128 Accession=cl02712 ID=96544

------------------VSHVFIHHTAGA-T--CNSKDTCAKVARQVQNYHMNT-------------------------
NKWADIGYSFLVGGDGRIYEGRGWKAVGAHTYN--FN-SKAIGISFMGNFDKK-
EPGSAMLNAARSLIDCGVEKKFITANREIHGHRDAK--CTACPGAAL

>Parasteatoda_8 aug3.g12678.t1 ProteinLength=201 DomainCoordinates=51-179 DomainLength=128 Accession=cl02712 ID=110024

------------------INKVIIAHTVTT-F--CRTKPQCVRNVQMIQDFCMDY-------------------------
NGMFDISYNFLIGGDGRVYEGGGWRQVGSHTIK--YN-YISIGIGFIGNYNIE-
KPTQKVINAALSLINCGVKQGFLTPTREIHGHRDVI--CTESPGNNL

>Parasteatoda_9 aug3.g12679.t1 ProteinLength=219 DomainCoordinates=53-181 DomainLength=128 Accession=cl02712 ID=110025

------------------VNHVVILHTVTA-F--CKKQVECMRQVQLIQDLHLDE-------------------------
RGWWDIAYSFLVGGDGRVYEGRGWNLTAAHAVN--YN-TNSYGVAFMGNFNND-
APTKPMIQAALRLVDCGVRKGYLTPTREIHGHRDVA--CTESPGEKL

>Parasteatoda_10 aug3.g18552.t1 ProteinLength=136 DomainCoordinates=22-112 DomainLength=90 Accession=cl02712 ID=116812

WDDIGYNFLIGGDGKVYVGRGWNRVGAHTYG--YN-RNAVALSLMGDFSNK-EPSSVMLNTTKSLIHHAMSENYVMENFKLHGHRDAG--PTECPGT--

>Parasteatoda_11 aug3.g18554.t1 ProteinLength=151 DomainCoordinates=35-139 DomainLength=104 Accession=cl02712 ID=116815

------------------VEYIIIHHTVTP-S--CTSFTECSKSMRTIQLQHQKD--------------------------NKWLDIGYHFVIGGDGHVYEGRPWYKVGAHTKY--HN-KKSIGIALIGDFDKE-APDPSMLDLIPKIAWCGVEKV--------------------------

BGRP/Glucanase alignment supporting figure 5

>Drosophila FBgn0040322|FBtr0075094

MRWEF-----LPCLLLLISNNKIFGFKVPSINFEMLKDEGFEVSIPDEPGIQRVFYMFQIDDTCPALMD------YITEA

VNGSWVSKQKM-SLQNNDKLQISMLVQFNEEIFEKSETRVII---NTRLLTTKDSSSRGITFLTGEGECQAYLAPAQQAK

RCKAAQTIVSNG---RHTCQGELIFEDNFSEAQLNKTTWKHDIRQR-----MYHVEEELVAF-DDAARNCFVKEGELHIV

PTIATE------VTDGSFKLG-----DRCTAVESPEQECN--IAHGIFYSIKPPVFSAQIHTRNSFSFKFGKIVVRAKLP

KGDWLFPYLMLQPV-STYAETHY-AKQLRIAYARGNANLR-----TKQGDDISGNHLYGGGVVWHHGNAVQ---FLKDKI

--SNSHYGDDFHNYTMIWQRDKITLMVDDEVYGELYDG---------------------------------LPFFN-EK

CFIIFGVTVGGF--LNFDDSLLAKD-----VKPYKNREP-RAALSFWQHRDAWAPTWGRH---------SAMVIDYVRVY

AE---------------------------------

>Drosophila _2 FBgn0040323|FBtr0075050

------MPGLCIGILLLIGFGCTTAYKIPTPTVELL-ETGFSVSIPDEEGVKVVAFNVNRNRNFTSFINEGQYNVRLTEP

QNGRWTTNFSSVPLRSQDVLYLWTSVQHQKAVYQDLAQPLPVCNLGGEYRPRGCSPGDDDFTDDNQLSTEDSALEPTAPS

VCEPSESQVSPQIG-VSICKGQLLFEETFD--QLNESLWIHDVRLP-----LDSKDAEFVLY----DGKAKVHDGNLVIE

PLLWSSYRPDLSIANSRLDLS-----ERCTGTHNRIKECILHSTGSGPSGIMPPIVTPRISTKETFAFQYGRIEIRAKLP

KGDWIVPLLLLEPLTEWYGQSGYESGQLRVALARGNSVLR-----MPRGKLVDGRSLYGGPVLSTDAHQREDLWLSKRKI

----SHFGDDFHTYSLDWSSNRLLFSVDGQVYGEMLNG---FTELDEN-------------PRW---KQGGPMAPFD-KM

FYISLGVSVGGFGDFV-DHLRTATY-----EKPWANYHP-QAKLQFHQAQDQWLPTWKQ----------PALKIDYVRVF

AN--------------------------------

>Drosophila _3 FBgn0040321|FBtr0076510

MADALRFVAWSCCLQLLFLLLGVQGYEVPKAKIDVFYPKGFEVSIPDEEGITLFAFHGKLNEEMEGL-EAGTWARDIVKA

KNGRWTFRDRITALKPGDTLYYWTYVIYNGLGYREDDGSFVV---NG-YSGNNASPHPPVVPVSTTPWTPPADPDIDIRL

GCTTPKTEVNGA---PTRCAGQLVFVDEFNAAKLDPNKWKAERRFS------GQPDYEFNVYVDDAPETLCLANGHVVLS

TNTMKKQFKKG--SGESLDLG-----EKCTGQAN-THDCV--RNGRTLNDGLPPMVTAQF-SSKDFSFKYGRVEVRAKMP

RAQWVTPQIWLQPRRPIYGVDDYRSGQLRIAYTRPN------------GGNLD---LYGAAVLFADEPLRSVKNCLKPGT

GNNSEDWSDSFHNYTLEWTPRELRWLVDGKEWCVQGSAKGSFSETTAAGKSLPQA------QKL---EEGTGLAPFD-QE

FYLTFGLSVGGFNEYQHE------------IKPWNERAP-QAQKAFWKEVKKIRDHWLDE---------GHMKIDYVKVY

SL--------------------------------

>Bombyx gi|261245087|ref|NP_001159614.1| beta-1,3-glucan recognition protein 4 precursor [Bombyx mori]

--MWLLTLGVVALISASK

ACTPSVTTVSGTHAPVTVCSGQLIFADDFV--DFDLEKWQHEN-TL-----AGGGNWEFQYY-NNNRTNSFTNNGLLYIR

PSLTSDQFGSAFLHSGRLNIEGGAPADRCTNPQW--YGC---ERVGTPTNILNPIKSARIRTVNSFSFQYGKVEVRAKMP

SGDWLWPAIWLMPAYNKYGTWPA-SGEIDLVESRGNKNMFLNG--LHIGTQEAGSTLHYGPFPGLSGWERAH--WVRRNS

----AGYDTNFHRYQLEWTPDFISFRIDDSEIGRVAPGNGGFWEYGGF----NNR--PGIHNPW---RYGSKMAPFD-QK

FYLIINLAVGGTNGFFPDGVKNP--I----PKPWWNNSP-TAATDFWNGQGGWLPTWNLNVNDGQD---ASLQVDYVRVW

AL--------------------------------

>Daphnia DappuP303036 pep:novel scaffold:Dappu1:scaffold_2:2839616:2841424:1 gene:DappuG303036 transcript:DappuT303036 description:""

--MKLPSIVVVAWAVIGS

A--YGQTTVNG----VEAVPGSLIFSDDFD--KLDFTVWQHEK-TM-----SGGGNWEFQVY-DNSRSNSFTKDSILHIK

PTLTEDRYGAGFVSTGTLDLNGGAPADECTNPSF--FGC-----
QRGGGNTINPAMAARIRTVNSFAFKYGRVEVNAKMP

TGDWLWPAIWMLPRHNAYGTWPA-SGEIDLVESRGNLRLMQNG--
VNIGVEQAGQTLHWGPYWPYNGYGNTA--WTKSST

----PGYNSAFHRYQLEWTPDYLKFSIDDVETGRITPGAGGFWDLGVS----TGAFPAEIENPW---RFATKMAPFD-EE

FYLIINLAVGGTNGFFPDDAVNEGGA----PKPWTSTSG-NALGDFWNGRGGWYPTWQAA---GEE---
AAIQVDYVRVW

AL----------------------------------

>Daphnia_2 DappuP342112 pep:novel scaffold:Dappu1:scaffold_6790:867:1819:-1
gene:DappuG342112 transcript:DappuT342112 description:""

----LWPSMWLLPTDNAYGEWPK-SGEIDMVEIRGNEKYVCDG--MQSGNKRANSTLHWGASVTQDKYTKTR--
WTKLLS

--N-GSFATEFHTYSLSWLPTGISFLIDGQVIGNITQPAGGFWKLGGS----NGT------NIW---ANGTIMAPFD-KR

FHLILNVAAGGD--YFPDRCFNYNDTGALVSKPWSTNAT-VQMKPFWAAKNQWYPTWTRN---PED---
SHMLVDYIRVW

SL----------------------------------

>Daphnia_3 DappuP28775 pep:novel scaffold:Dappu1:scaffold_3:1249539:1249968:-1
gene:DappuG28775 transcript:DappuT28775 description:""

--RNGVLYIK

PTLTADRFGEDFLYNGTLDMW----KEGCNVNYN--GGC----IATSAEDIINPIQSARMRTLNSFSFTYGTVEVRAKMP

RGDWIWPAIWMMPTENRYGAWP--

>Daphnia_4 DappuP310821 pep:novel scaffold:Dappu1:scaffold_3:1251646:1254046:-1 gene:DappuG310821 transcript:DappuT310821 description:""

--MEGKDAVVIVWLLLLTVAS

D------RVKGTWSQFYHLRGRLIFQEEFD--TLNTSRWQHII-TA-----WRGGNNEFEYY-TDRPENSYVRDGVLHIR

PTLTADRFGQDFLYNGTLDLW----PEGCNVNYN--GGC----VATSDEDIINPIQSARMRTINSFSFTYGTVEIRAKMP

RGDWIWPAMWMMPTENRFGPWPR-SGEIDIVEIRANNDFTCRN--KQMGNTLMGSTLHFGTDAQHNVWRPTH--YEAVLE

--E-GDFASDFHVFGLQRLPQSIRFYVDGILIGEITPPDGGFWEVGQLDRDPGGP------NIW---GNGTTMTPFD-YP

FHFILNVAVGGN--FFPDGCINHQ-F----QKPWSTKIK-QQMLPFWEKRHEWLPTWNLQ---HGDGASNALQVDYIRVY

EYDPNDTVD------WYWYQRGLASNSLVDSQRLNV

>Daphnia_5 DappuP216163 pep:novel scaffold:Dappu1:scaffold_79:102779:104338:-1 gene:DappuG216163 transcript:DappuT216163 description:""

---MKHLFLVFSILQCF

V--------------ISVFAQNLIFEENFN--DFNRTRWMHLI-TA-----WRGGNSEFQYY-TNRPENSYVKNGMLFIK

PTLTADRFDNNFLYSGTLDLN----KEGCNLNLD--NGC----SVTAGAEIINPIQSARLVTSNSFSFTYGTVEVRAKMP

RGDWIWPAIWMLPTDSAYGTWPR-SGEIDIVEIRGNADLTCNDGQGKIGNSKMFSTLHWGPGFSQNMYHKTS--WPKSLT

--DGSTFSSDFHVYRLEWLSTGFTFKVDGQVIGSMSPPAGGFWEFSGL----SGT------NPW---ASGTKMAPFD-KR

FHFVLNVAVGGH--FFPDGCVNVP-Y----DKPWAKSSS-TPMRNFWEKRCQWYPTWYKT---SRDD--AAMQVDYIKVW

SA----------------------------------

>Daphnia_6 DappuP201160 pep:novel scaffold:Dappu1:scaffold_79:140914:142474:-1 gene:DappuG201160 transcript:DappuT201160 description:""

---MKHLLLVFSILQCF

V--------------ISVFAQNLIFEENFN--DFNRTRWMHLI-TA-----WRGGNSEFQYY-TNRPENSYVKNGMLFIK

PTLTADRFDNNFLYSGTLDLN----KEGCNINWE--NGC----SVTAGAEIINPIQSARLVTSNSFSFTYGTVEVRAKMP

RGDWIWPAIWMLPTDSAYGTWPR-SGEIDIVEIRGNADLTCNDGQGKIGNSKMFSTLHWGPGFSQNMFQKTS--WAKSLT

--DGSTFSSGFHVYRLEWLSTGITFKVDGQVIGSVSPPAGGFWELSGL----TGT------NPW---ASGTKMAPFD-KS

FHFVLNVAVGGN--FFPDGCVNVP-Y----DKPWAKSSS-TPMRNFWEKRCQWYPTWYKT---SRDD--AAMQVDYIKVW

SA---------------------------------

>Daphnia_7 DappuP329544 pep:novel scaffold:Dappu1:scaffold_121:364366:365718:-1 gene:DappuG329544 transcript:DappuT329544 description:""

--MI-SG-----WRGSN-SFQIY-VNRSENLYVRDGHLFIK

PTLTADRFSPEFLYNGTLDLT----LEGCNVNWG--GGC----FLEAGDDIIQPIQSARIHTKKSFSFTYGIVEVRAKMP

KGDWIWPAVWMSPTDSVYGSNPR-SGEIDLTEVRTNANLSCNG--KPYGRRLSGTTLHWGPDAQHNGHRMTY--WQKFLS

--H-PDFSSDFHLFGLEWSPTGFEFSVDNILIGSMFPPPGGFWEMGGF----EGE------NIWNLTGNGTRIAPFD-HP

FHFILDVAVGGN--MFPDWCVNQP-FGQPLEKPWKMSDP-VQMRPFWENREHWLPTWNIE---TED---NAMRIDYIRVY

ALNQYNKT----------------------------

>Daphnia_8 DappuP228840 pep:known scaffold:Dappu1:scaffold_121:373742:375196:1 gene:DappuG228840 transcript:DappuT228840 description:""

---MMKLSIFALFALTVCH

-----------GFSIDNQARGPILWQDDFE--SLDTSKWKHLI-TA-----WRGGNSEFQYY-DNLPENSYVRDGILYIK

PTLTADRFGEAFLYNGTLDLY----KEGCNVDID--GGC----YVVASAEIINPIQSARMVTSDSFSFTNGTIEIRAKMP

KGDWIWPAIWMLPTDSVYGEWPR-SGEIDIVEIKGNADFSCNG--YPIGRQLAGCTLHWGPDPGQNRYPLTH--WEKIMQ

--D-PDFSSDFHIFRVEWFPNGFQFFIDDEMIGEVYPPPGGFSELGGF----GEQ------NLW---STGTKMAPFD-QP

FHFVLNVVAGGN--FFPDGCQNAD-Y----EKPWNAYDP-TQMKTFWESRDKWLPTWNAD---TED---NAMQVDYIRVY

ELN--------------------------------

>Daphnia_9 DappuP203138 pep:novel scaffold:Dappu1:scaffold_121:375595:379407:1 gene:DappuG203138 transcript:DappuT203138 description:""

---MELTILALFFLVVCQ

A----------AINIDSQERGPIIWQDEFE--FLDYSKWMHLI-TA-----WRGGNQEFQYY-HNLTENSYVRDGILYIK

PTLTADRFGEDFLYNGELDLN----QEGCNVDWE--GGC----YVAAGEEIINPIQSARMVTSDSFSFTYGTIEVRAKMP

KGDWIWPAIWMLPTDEIYGGWPR-SGEIDIVESRGNADFSCNG--YPIGRQLAGSTLHWGPDPNQNRFDLTH--WEKITQ

--N-PDFASDFHLYRVEWLPTGFQFFMDDQMIGEMYPPAGGFWELGGF----QGQ------NLW---SGGTVMAPFD-QK

FHFLLNVAVGGN--YFPDGCQNAV-Y----DKPWTASDP-TQMKTFWESRDKWLPTWNAA---TED---NTMQVDYIRVY

QLG--------------------------------

>Daphnia_10 DappuP332783 pep:novel scaffold:Dappu1:scaffold_207:53297:54787:1 gene:DappuG332783 transcript:DappuT332783 description:""

---MTLAIALIAHIVLVV

-----------GFARPTSAERRLLLREEFD----SMERWDYVV-TS-----YRMNENQFQYY-TRRPENSFIKDGKLFIK

PSLTTERFGEKFLHNGKYNLK----REGCNLAVD--GGC----VLKANHDIANPIQSAALVTKSTFTFTYGTVEIRAKMP

RGDWLWPEISLMPANNVYGEWPR-SGYIGLVSVRGNNAFTCNG--QSAGNNVMESSLEWG--LNNDHIKSIT--WTKEAQ

--GNASFSSEFRTYRFEWNPSSMHYFVDDQMVGSLEPPIEGFWELSRF----NDT----VDNPW---INGTSMAPFD-RE

FFIAINVAVGGD--FFPDSCENYP-Y----PKPWNNSSPDASMGSFWEKKDQWYPTWSQF--SVDD---SALQIDYVRVY

AHPTMG-----------------------------

>Strigamia SMAR012410-PA pep:novel scaffold:Smar1:JH432134:224141:226940:-1 gene:SMAR012410 transcript:SMAR012410-RA description:""

--MQVQIVVFITFITVNV

ILL-----------LCNGEPELIWSDEFDGQSIDRTKWAYYTGKA--------PNNELEQY-TNRTENVFVENGNLVLK

AIKEI--YNGHNFTSGEIYSQYKGDFLYKTVKVRAKLP

YGRGVWPAIWMLPTYYKHGPGIA-SGEIDILELLGDKP------------NKMYSTCHYGKTWTDRDSKSSN--YSLPH-

----GDFSDDFHVFSMKWSPDLIRIFVDDVQIINFRPSD----------------------------VPKKYTYPFD-DY

FYIILNVAVGGN----------------------WPGSP-DASTVF--------P--------------QVMLIDYVRVY

K-----------------------------------

>Strigamia_2 SMAR009322-PA pep:novel scaffold:Smar1:JH431882:45496:49114:-1 gene:SMAR009322 transcript:SMAR009322-RA description:""

---MKLFLVLFN

FLL------------ANANEWELIWQDNFD-HHIDTQNWVHEIGDGCP-NICGWGNNELQYY-TDSSSNSYVQGGHLVIQ

AKKEN--LHNKEYTSARMITKGKAQWRYGKFEISAKLP

KGSGIWPAIWMLPVNNILGDWPR-SGEIDIMELLGQEP------------SIAHCTAHYGNSYNDKGQKSTS--YNLAT-

----GSFSDDFHLYEIIWNEDKITWYIDGNKVQEVINGQT-----------------------------PPYTYPFNSDD

FFLILNVAVGGY----------------------WPGSP-KDDTTF--------P--------------VKMEVDYIRIY

RNKNSTDTSNDFRGTSHSLLDWLKLLFIVGIGLVTQ

>Strigamia_3 SMAR001813-PA pep:novel scaffold:Smar1:JH430824:75719:76528:1 gene:SMAR001813 transcript:SMAR001813-RA description:""

--MRFFFRVSFLVIIH

DCLFE----------SKSDYWQLVWDDEFEGPLIDTNRWNFEIGDGCKYGICKWGNDEQQLY-TDSIDNAFISDGKLIIQ

AQ---HIDNQYTSARMITKGKGDWKFGKIEIRAKFP

SGEGLLANIWLLPTYSRYGLWPA-SGEVDIAARFGEKT----------GRSVS-TIAHYGAVYPDHRHSRRD--YTVNYP

--Q-PSFTEEFYTFTFEWEENKMQWFINNVKVNELAGDET---------------------------FPSKYIFPFN-EK

FHLTLSIAVGGR----------------------PVFGN-ILETVF--------P--------------QRLEIDYIRVY

QKK---------------------------------

Alignment of TEPs and MCRs for Figure 6

>Drosophila FBgn0041183|FBtr0080811|TepI

-------------------------------------MLWL--------ILSSTI--

---------------------------LHC---
-------------------------------------VLLSNANGLYSVLAPKTLRSNSAYNVVVAIH----NTTR--TTEVSVSLTGPS--------
LNSRKYVDVQS-----------MSSKSVRF--------DIPKLTEGDYELKVMG------SG---GIEFQNSTK---LSFAP-DL-
NWLYIQSDKATYKPGD-KIQFRVLFLDK-NTR--PAVID-----------KPIKIEIR-----------DGDQNLIKS----
WKDIKPAK-G------VYSGEL-------QLSDRPVLGNWTVTATVQ---D---------EGKVTNV-----------------------
LVVDKYVVPK---------------FEVVVLT-AK--NV-AAS---AGYIRATIKARYT-FKKPV-KGHVVATIEGSSTEQSLP--------
--IDGEVNVEFP--
ISA-----------------TAKRLLKITAIVTEELTDIKHNG-TAYVTVHQRHKLEDLFW---PTHYRPGVSSEFKTVVRN-
LDGSPVM-----D-SSKMVNFNVLCCQV------------------------SKNFSASLQN------------------------------------
SIATEHIMLPE---TCQSCLVTS----TFDT-----------------
AENIERYIYKLNKP-LM---------------IAI----------NTKKPQL-------RKLLKINIISDTYL-PY-----------------------
FILTVVAR-------GNIVLSLFQEMKEKKK---SQEIEFEPTFALVPQATIFVHYI-
-----IDGV-LMSDEKTVDI-ERDFEN----T----IEIL-----------TTNEALPRDEVSLKVKTNPH-S--FVGLLGVDQSV--------
--------LLLRS--GNDLNRD------------------------------------L-----ILNNL-ATYSTDLVI-------------------------------------
---LTNANINIYRSSGGCY---------TNPGYTNCTGS-------LIGRTMFKNEPTKN--
----
SGPVPIVGSTRAQASLPPVRKLFPETWLFSNITDV-----GANGE-YIIKE--TVPDTLTSWVITGFSLSPQSG-LAV-
TRNPSRIRVFQ----------------------------------PFFITTNLPYSVKRGEVIAIPVIVFNY-LGM--DVKAKVLM--------------
------D-NSDGQYEFIETTNKNVSQYL-----RGVRRKKTLW--------IPANTGRGISFMIR-----P-KKV--G-----
LTTLKITAISK--------------------YAGDRLHQILKVEADGVQ--KYVNKAVLINVQRLNRRSLAPPEKTI--------------------
-IIEKADN---VIEGS-E-TVEFEVC---GTSQAP-QLE---H------
LDDL-VHLPCGCGEQNMFNFVPSILALSYLKAKNRQD----QEI--ENKAKRYVETGYQIELNYK-R--NDGSFSAWG----
-QHDAL---GSTWLTAYVIRSFHQAA--------KY--I-DIDKNVLVAGLDFLV-SRQST-DGKFKELGMVIHNS---------
HGS------------PLALTSFVLLTFFENEEY-----MP---K-YKHVIDRA--VEFVVTEVHQ-SN---EPYDLAIAALALSLA----
RNRNAYKVLDKLDKLAT----------------RR---G--DHKWW---------------TGSD------KC------KSSEVETTSYVLL-
ALLEHN--I---SDEPKPIVDWLISK--------
RNSNGGFVSSQ-----DTVVGIMALT---KYELQS-HAS-----TE-AIDIEFWHLN----EDKKHVRVT--KENEFKVQTHQL-
PENTN------EVKLLAK-GQGRAQVQLTYRYNVA---TKEA---RPSFKLTTT---V--
-------------------------------------KKSHKGRLILG-ICGTYTPIAASERNKTTNMALMQVQLPSGYVCDIEPFADIE---------
----

--AISDVKRVETKNED-----TEVHIYFEKLSPGDRKCLTLEAIYTHAVANL-
KPSWVRLYDYYATERSATEFYHV-D-TS-LCDI-CHG------NECGNMC---

>Drosophila_2 FBgn0041182|FBtr0079510|TepII

------------------------------------MFRI--------FLTGII---

--------------------------LQY--
---------------------------------ALLVNATGIYSVVGPGTLRSNSKYNVVVSVH----KADG--PSQIKVSLNGPS--------
YNETKQIELPP-----------MSTQNVEF--------EVPKLATGNYNLSAEG------VS---GVVFKNSTK---LNYAD-KK-
PSVFVQTDKATYKPAD-LVQFRILFLDE-NTR--PAKIE------------KPISVIII------------DGAQNRIKQ----LSDVKLTK-
G------VFSGEL-------QLSEQPVLGTWKISVSVD---G----------DNRETKS----------------------FEVDKYVLPK----------
-----FEVIVDT-PK--AV-VIA---DKVIKATIRAKYT-YGKPV-KGKATVSMERSYGYFGDLNANGNKQEK-----------------
TID--VDGKGHVEFD--IIHWAQ--RG---
-------QYLPPIKLFAVVTEELTGNKQNA-TATVVLHQQRYSIEPYER---PEHFEANKSFIYQVVVKN-VDGSPVT-----N-
SAKNVKIGFDKSYSYFHEPSPKTR--------------INFEAPVNEN------------------------------------GIATFNVRLPDS------
---DSRYYRIFA----SFDGSENTIG-----------SISKFEPTPMSREP-LK---------------
IQV----------NTKKPRL-------GEQVSFDVVSIEDL-PY----------------------FVYTIVAR-------
GNVILSDYVDVPDGQK--TYTVKFTPTFSMVPKATIYVYYV------VNND-
LQFEEKTIDF-EKEFSN----S----IDVS-----------APTNAKPSEEVKLRIKTDAD-S--FVGLLGVDQSV----------------
LLLKS--GNDLSQD------------------------------------D-----IFNSL-NIYQTS---
TPWMN------GYGRY-------PGQTSGLVTLTNANYPYNTGPLVMSYVFEGSRHP----------------
WITRPRYRVGIRGDSGDRISFLSQSLND---
RNLKEILLKQTPQRTTIRKEFPETWFFENVG-------EEE---FTLTK--KIPDTITSWVVTGFSLNPTSG-IAL-

TKNPSKIRVFQ-----------------------------------PFFVSTNLPYSVKRGEVIAIPVVIFNY-LDK--TLDADVVM--------------
------D-NSDQEYEFTEATNEVLEKAI-----DEVRRVKRVT--------IPANSGKSVSFMIR-----P-KNV--G-----
FTTLKITATSA--------------------LAGDAIHQKLKVEPEGVT--LFENRAVFINLKDQPEMS-----QSL--------------------
DADIPNE---VVPQS-E-FIEFSVV--GDLLGP-TLQ---N------
LDNL-VRMPYGCGEQNMVNFVPNILVLKYLEVTGRKL----PSV--ESKARKFLEIGYQRELTYK-H--DDGSYSAFG-----
KSDAS---GSTWLTAYVMRSFHQAG--------TY--T-DIDPKVITAGLDFLV-SKQKE-SGEFPEVGKLFDNA---------
NQN-------------PLALTSFVLLAFFENHEL-----IP---K-YQSAIKKA--VRYVAEEADK-TD---DQYSLAIAAVALQLA----
KHPQSEKVIAKLESVAR------------------KE---N--DRMWWSKA---------TESTGEDGRVFHWKP------
RSNDVEITSYVLL-ALLEKD--P--AEKALPIIKWLISQ-------
-RNSNGGFSSTQ-----DTVIGLQALT---KFAYKT-GSG-----SG-TMDIEFSSAG----ESKNTIKVN--PENSLVLQTHDL-
PKSTR------KVDFTAK-GTGSAMVQLSYRYNLA---EKEK---KPSFKVTPT---V---
--------------------------------------KDTPNQLLIVD-VCAEYVPLEDADKDKDSNMAVMEIALPSGFVGDSTSLGKIQ-------
----

---AVDRVKRVETKNSD-----STVVVYFDSLTPGDVRCLPLEASKAHAVAKQ-
KPASVSLYDYYDTERKATEYYQV-K-SS-LCDI-CEG------ADCGEGCK---
---------------------------------KD---

>Drosophila_3 FBgn0041181|FBtr0079477|TepIII

--------------------------MRLQGADMGAIPVLI--------LVTACL--

--LCQ---
---TSAQGLYSIIAPNTLRPNSQFHVAVSLH----NAPE--SATFKVGILGSS------
--------------YTDFKTVELRPFSTQLLHF--------EIPALRTDRYRLTAEG------LG---GVQFTNETQ---LHFES-KQ-
HTVLVQTDKSIYKPGD-LVHYRVLILDA-NLK--PARGY-----------GRVHVDIK-----------DSGDNIIRS----

YKDIRLTN-S------IYSNEL-------RLSDSPRFGTWSIVVDV----S---------DQEHTQT----------------------
FEILDHILPK---------------FVVDIDT-PK--HA-IYK---DGKIAATVRAHYA-FGQPI-
VGEATLSIYPTFFGSLQPFVNDLITRKVVP--
---------------------IDGNAYFEFD--IENELH--LKQ---------DYERQYLLDALVEEKSTGSVQNY-
STVLTLHLNHYRVEAVKV---PSYYIPGVPFEATARIAR-NDGGQLR-----D-FNPQITAYLTNVYGSSEMY--------------
----NRTAYSLDAS----------------------------------GEIKMKFTVPIGD---
RDEFHSIIV----DYQG----------------VISEVGKIPSKHLH-SK-NY-----------ITA--------KVLNDRPTV-------
NQEISVVVRSFAPI-KY----------------------FMYQVVGR-------GDIILSRNVDVAPGTF--------------------------------
---------------HTIKFLASFAMMPRANLLVYTV------IDGE-FVYDEQVIQL-EENLLN----A----VQVD----------
APIRAPPGQDIDIGISTKPY-S--YVGLMLVDQNA---------------NDLRS--GHDLTHK----------------------------------
R-----LMDAL-RSYELSDVNTP-----------------------------------MGSPG-------------------KESGVITMSNT-----
DYFIEKEAESNPALDR--------EVSTGPEEDKLTTVRKTDIGPAHKIEVNTLPPGKGR-----------------------------------
--------------------------------------YAFSYTPKPFWHNPRVHVMRDPADTWLFLNIS-A-----SSDGR-NSIHR--
RIPSEMTSWVVSAFALDPVNG-LGL-SPPNGKLEAYK--------------------------------
EFYISTELPYSIKRDELIAIPFVVHNN-RDS--DLNVEVTF------------------Y-NSALDFDFPQLDPKATNQPK-----
VELYRRRSLQ--------VPGRSARSVSFIVT-----P-KRV--G-----PLLVKAMAASS-------------------
QAGDTVEQNLLVEHPGAM--ERINRGFLFELNSNAQNR-----RNV-------------------TIAVPRN---AIPES-T-
RIEVSAV---GDLIGS-LVG---N------LDSL-
ILLPTGCGEQTMVNFVPNLIVLRYLGRLRQLT----PEV--ELRATNNLAIGYQRILYYR-H--ENGAFSAFG-----LDIK---
RSSTWLTAYVARSLRQAA--------PF--T-QVDSNVLQKALTYLG-SVQSA-NGGFEERGDVFERF---------GDD--------
-----GISLTAFVTLALMENVDL-----YP---E-YRNNINKA--LDFITRGLDG-SS---NLHAMAIGTYVLSRA----
NHNAKAAFLQRLDSMAT---------------NK---D--GLKWWNKT-----------APAGEQQSPWYNAT----
RSVNIEISAYAAL-ALLENNL---VGDALPVLNWLMDQ--
------RNPKGGFVASQ-----DTVVGLQALL---MFAERF-SSQ-----GN-NLQIGFHYGE----GAETIINVN--
AENSLALQTVEL-PNNLK------NLSVSAT-GRGMALAQVSYTYNTN---VTSA---WPRFVLDPT---VN-----------------
--RNSHADYLHLS-ACASFV-
SVVGENEQRSNMAVMEVHLPSGFVVDRDTLPTLE---

SSERIKKVETQNRN-----TKVVIYFDYL-DRREVCPTLHAYKTVKVTKH-RPVAVVMYDYYDSARRARQFYRA-PKSN-
ICDI-CEH------ANCGDLCE---KAEKRESKRP-
-DDYTAIA-----------GHSSGSRHTAIPLASVVMVLSMLLKTLSC--

>Drosophila_4 FBgn0041180|FBtr0331329|TepIV

------------------------MRRADAFAVSLCV--------ILA--L---

------------------------------LQT--
--------------------------------------MEPVKAEGKYTIVGPGTIHSHRDYNVAVAVH----QTKE--PVTLKVGITGPS----
----YNKTETVELATA----------GEFKQITF--------KLPPLEAGEYNLTAEG----V-KG----LEFKNSTK---LNWEN-FK-
PYIKIQTDKGKYKPGD-TINYRVIFLDE-NLR--PDTAKD-----------E-VVVWFE------------DSKRNRIKQ----
EKHIKTTG-G------VYTGKF-------ELSEFATLGSWSLHVQNG---D----------
QHHDGGIYFGGRKQFGGFGHRWHRSDELVNFEVEKYVLPK---------------YSVKMDA-TQ--QV-SVR---
DGEFNVVLKANYT-YGKPV-NGKVLVNVHLDS-TSSWENVDGKTVQTDYPGHSVVGT-------ADM-----------------
---VGGKAKLTMD--LKDFAS--YLPHKT------SSSY-
AQITATVEEDFTGVKLNE-TGGVQLYPYRYEMSCTDYSS-CFSFKPDKEHELNFKITY-VDGSLIT-----D-
TKSVVKAKFTEGIRRNYAFYAFGTDHQEPELPTIEKKTFVFESHLNAS---------------------------------
GVAPFKVVLPDLPDIAN--FTRYYSIEL----EFVD----------------
EKRDLYTTYPYREP-KQ-IENPSSEEEKEWFRAEV------QRPKDVWNLKI-------GQEYQVILNSSRPL-KY--------------
--------FVYNIVGR-------GNILETKRVDLAEPQT--
TVNVTIKPTFLTTPYGRVYFYYV-D----ETGE-FRYTEETFSV-EVELQN----Q----IEIK-----------
APAEVKPGADVALEIKTSPK-S--FVGLLAVDQSV----------------LLLGS--NNDLNKE----------------------------------
S-----FNWRL-NGYDTSTP--------------------------------------WQGGY------SYYPG---------ERTGVVTMTNA------
-YFFYNR------------------------TAPDYNILTEGFGGSSFAMRKTTVAH-------------DSHVFHSG---------------------
-------------------------AG---GPTQAVGFSAESASASAAPVVRKNFAETWIFADIEST------EEEV-FKWVK--
TIPDTITNWVVTGFSLHPQKG-LGV-TNDQTNIKTFQ--------------------------------
PFFVSVRLPYSVKRGEVINVPALVFNY-LPK--TLDVELTL--------------------D-NEDQEYDF-VDASNEVIGDQ--------
KRTQNIR--------VGANEAAGASFLIR-----P-KVI--G-----NILLKFKAISP-------------------LAGDAIHKPLKVVPEGIT--
QYQNRAFFINLKDTGEFK-----NTF---------------------ELEVPED---VVPDS-E-RVEFGLV------------------------------
-----------------------------GDLLGP-VVK---N------LENL-LRLPSGCGEQTMSKLVPNYLVRDYLKSIKKLT----PAL--
DTRIKRNLQDGYQHMLHYR-H--DDGSFSSFGPTKWRQEDPVR-NGSTWLTAYVLRSFSKIK--------DI--I-
DLDEQILAKGYEFLL-TRQAE-NGSFTEHGEYFYSS---------QRS-------------LLTLTANSLLALLEEEK-------P---N--
QAAIDKA--VAYLSANTAE-SI---ELLPKSIAIYALQKA----KAPEAAKQVASLKSLAK----------------HE---D--
DRTWWTED---------LDKLRASKNCGRWWCWI----WSQDVEITSYALL-SLLDSDQET-------------------------------
---------------------------------ADSVLNTVRWLIAQ--------RNGFGGFASSQ-----DTVVGLTALI---KFAEKS-GYE----
-AA-KWEVTVSNKG----KREKTEKLNTSEENDLLLQTVEF-PQGTK------SLEFEAK-GTGAAMVQISYQYNLV---
EKEP---KPSFKIQTT---VL---PESSPANLELS-
VCVDYV---EEGESKESNMAILEVSLPSGYTADEDSFADIR--

NIERVRLVETKNGD-----SVVVIYFENLAKNEEKCIRIEAYRTHAVANQ-KPSSVVLYDYYDTNKKATEYYSI-K-SK-
LCDI-CEG------DDCKSKC--

>Drosophila_5 FBgn0032808|FBtr0331331|CG13079|Tep5

---------------------------------MVVVYLNNLPKNDTKCIPIAAY---RTHKVDNQSPSSVVLYDYA-------------------------
-----TKIKEDTF--------KVPDISSGDCALTAEGVS---------GLKFQNSIS---LYFQH-DT-PVVIILIDKDDYIPRD-
MINYRVLVLNE-QLR--PDTYA------------EDVVICLL-----------DPERNKVDK----ARKSKVTDK-------VSIRAI-------
SLVQE--GNSWTVGVESRRC-R----------WCRIRSS----------------------IEYIHLSLPK---------------GDVVKG----------
-----LDTFHIDLTDIAK-HKAPI--
---QTTYISTIKAAVEDGDSGEKVTK-
TTYVYLLENRYIALNPFQLIYSEEMEKGDCYFTKLRKVD--
ERKRFEFDGQLNKS-----------------------------------GLYVFQANLSD---
--FP-KNDKY-------LAYYDISVEYKGEEQRIQQTIAQSM-------
GFMIDVTNAYFTPR-IDVN--------------------FFLQIHGRKNDKGFPGALVVGDEFRVTLNSN-------------------------
----------------------------APITHLTAPHFNIYAYYV-D----DQGV-LQYTKSQYKV--------------LMSLS-----------
ALSTGYPEEEIKLRIRTETN-S--YVGILAIDERISGRIKTDDIKHDNLDYQFYTNKLSNALLFN------------------------------
------K-----PPTELPRNHDIDLS--YLEEQ------
LEDVYLHTYEDIPGSRLGLVTMTNA-------QIKERIFRYFDMDK---
--YVSARSISVKYGKSPLRYPMKTTETWLFTDIR-------
KSQKEVTDLEI--KLPDTLGTWIVKGFSLHPEKG-LGIFQSNLTQIRTIK------------------------------
PYSLFIHLPYSVKLGETVRIPVLIVNL-FFTCFFFKVELAL-------------------D-NEAYGFEDS----------------
LEHSQRQLFE--------IDEYGAKSVFFFKFC----P-KYT--GN----FIPLDFRATSS-------------------

VKYTTVYRSLMIIEDKEREIEYSNRALLVNLKDNQEYRSS---FHM-------------------------------DLPNS-R-LVEFSLF----
---SDPLVP-ILQ---N------LDSS-I--
PDGTGELTMSKMLVNFLVWNYLNRTKKLD----KAL--HTRIKSNFREGYQKILNYF-H--EDGSFSYFD-----PPKA---
NGSIWFTSYVLRYLHDIR--------AL--I-YIDRTILQKGYRFLL-SRQHK-DGSFTEDFKYFSR----------SGS------------
TLFLTSSVLLALQKQA--KPNTIAINK----------SAPESDQLVSQLRSVAM------------
---------QENDQIWWAED--------------------------VRNGSPQDVEITLYALLLMASKVDR---------------------------------
--------------------------------------PESAISTVRWFLDQ--------RNIHGEFESSQ-----KTVVGLTALI---EYAEKWDYNP--
-----TAWEVSISNED----ISEKLVAFN------LLTQPINF-HQDTK------RLETTLKSGSLSSKMEAV-------------------------
---VCVEFV----
ERSTNASNMAVLEVSLISGYSSDKSSFKRIR--

---DVEGVGSVDTKNED-
----SVVIVHFESLAKNESKCIPVEAYITHEVTNF-KPSSLVIYDNY--GRKATEYYQY--
----

>Drosophila_6 FBgn0020240|FBtr0079543|Mcr|Tep6

----------------------------------MMWHL--------LRALLV---

VAAVLDALQPAVGQNDNYYNPNQNQQNPQQPLLPNQQWGNNPQTNQYSNNNQNFGQTNPSDRPPYRTDSG
SYNDIAGQDDYNKRVGGGYQDNEEPSLTRGKSSYNIKATFLESLHSR-----------------EP------------
TYFIVASRMVRPGLIYQVSVSIL----QAQY--PITVHASIACD---------------GVQISGDSK-DVKEGIPETLLMRI--------
PPTS-VTGSYKLRVEG----FYQNVFGGLAFLNETR---LDFSQ-RS-MTIFVQTDKPLYMQGE-TVRFRTIPITT-ELK--
GFDNP--------------VDVYML------------DPNRHILKR----WLSRQSNL-G------SVSLEY-------

KLSDQPTFGEWTIRVIAQ---G----------QQEESH-----------------------FTVEEYYQTR---------------FEVNVTM-PA--
YF-FTT---DPFIYGRVMANFT-SGLPV-RGNLTIKATIRPIGYFSNQVLNEKYRLG----------------
RSPLEQTNLYNERWRYNNPNQNPQVQYNVPGQLPQDGADLSQDILYRNQYVVERHYQFDEEWPFWVRKPEYQ
DSSYEAWSGTYRKTLPYLRYFNGTFDFKWP--LRELEL--LVP--N------LANSEVLITATVGEKFYDEIISG-
YSVARVYNSSLRVVFLGDS--PQVFKPAMPFTTYLAVEY-HDGSPIDPNLLRQ-GLMEVSGFVESRNGGRRDW-------
------------PAQRLPMSQQ---------------------------------SDGIWEVKIDIRNDL------NLD-DRPQARDFLN------------
-----------------GVQNMRLQA----NFVDP------R-GE------RIQTELLLVSHYSP-RN-QH------------IKV--------
TTSTEKPVV-------GEYIIFHIRTNFYL-EE-----------------------FNYLIMSK-------GVILVNDRETI---TE------------------
-----------------------------GIKTIAVVLSSEMAPVATIVVWKI-N----QQGQ-VVADSLTFPV-NGISRN----N----FTVY--
--------INNRKARTGEKVEVAIFGEPG-S--YVGLSGIDSAF----------------YTMQA--GNELTYA---------------------------
---------K-----IITKM-SNFD-EQTNGTY--KHIWYS----HE---------G-NPDELVYFPAS-SFGVD------ANRTF---------
EYSGLIVFTDG-------YV-PRRQ--D--TCNRTLGFGECL-----
SG-RCYRLEKQ--CDGLFD----CDDGTDEINCHAR--------NDTELLNYRKYRFNRVLRHYE---NVWLWKDVN-I-----
GPHGRYIFNV---EVPDRPAYWMVSAFSVSPSKG-FGM-MNKALEYVGVQ---------------------------------
PFFINVEMPEACRQGEQVGIRVTVFNY-MIT--PIEAIVVL--------------------HDSPDYKFVHV---
EEDGIVRSYNPRTSFGEHQFFIY--------LEAQGTTVVYVPVV-----P-QRL--G-----NVDVTLHVATL--------------------
LGTDTITRTLHVESDGLP--QYRHQSVLLDLSNRAYVLEYMHVNVT------------------QTPEIPYQVD-RYFVYGS-N-
KARISVV---GDVVGPIFPT------MPVNASSL-
LSLPMESGEQNAFSFAANLYTIMYMRLINQRN----KTL--EKNAFYHMNIGYQRQLSFM-R--
PDGSFSLFRSDWNNSD------SSVWLTSYCLRVFQEAS--FYEWE-NF--I-WIDATIIEKNMRWLL-QHQTPQ-
GSFFEVTWLP--DRKMNRTN--FDK-------------NITLTSHVLITLATVKD-ISGTLGS---R-VALATQRA--
LAYIERNMDF-LRHQAQPFDVAITAYALQLC----NSPIAEEVFAILRRQAR---------------------TIGDFMYWGNQEIP-
-----QPPRKLENQKWFSLPRLPYEYDSLNIETTAYALL-VYVARRE-F---
----------------FVDPIVRWLNSQ--------RLNDGGWASTQ-----DTSAALKALV---EYTVRS-RLR-----
EVSSLTVEIEASS----QGGKTQTLYIDDTNLAKLQSIEI-PDAWG------TIKVQAK-GAGYAILQMHVQYNVD-
IEKFQTKPPVPAFGLHTK---AIFHG-
RNQSHISYVACQNWI---NQNESERSGMAVLDVAIPTGYWIQQQKLDTYV--

------LS---NRVRNLRRARYLE-----RKIVFYFDYL-
DHEDICVNFTIERWYPVANMSRYLPVRIYDYYAPERFNESIFDALPTYLLNICEVCGS-----------------------------------
----

SQCPYCSIYNMGWRASMSMSLLFFSVFIYLLRSRTHLVLNMMQLLT---

>Apis_TEP7 |GB12605|GB12605-RA

---------------------------------------MNKLV--------FLVLFI---
----

------------------------------CSV--
-------------------------------TFLHCCDAVSNVNRGYVFTAPKKLYAGEIETGCLSLH----NLELPAHVHLELVFSFLE-------
-------GQEVLASTSAVVKTGTETCLQLAV--------PSPIYRTAILRLKIK-------FDKYPDYVIKSETD---VEIEH-DS-
LLTFVETDKPTYKPGQ-DVKIRILMLMH-DLK--PWQKS-------------IPEVWIE------------NPSFVKVKQ----
WTNVSTEN-G------MAQLTF-------PLSPEPSLGSWHIKVMKKKPYP----------NLIHSTT-----------------------
FKVEKYVLPK---------------FQMTINS-PQ--YI-LAN---VENVTWNICVKYS-YGKPV-
KGNLLLKLTPQTPSWTRLPNLPAIRYETKLDK---
------------------------GDGCTDFVLSGSVLGLAHWKMDPNN------------IVLIAEFTEAGTGIVETT-
ISRTVVLHEALKLEYEHYT--PKYFKFGLPYHGKLRVLR-YDDTPAP-----N-EKIQICLKVRGKIEWEKDVV---------------
---DCRDFRSSTD------------------------------------GFVDFVVPPQHK---
NIVLLSFVA----TAVDYPTTYYSPEQRWRVFMNQPSTSITVNPWYSP-SD-SY------------LTV------ARGNQPIVCGE-
------KYSFNVMYTTSSNM-NETIS--------------------FHYSINSK-------GSILIYGHVKHKPNRD--------------------
TILNYFEFHNLLGTIESSANKTNKEAIVHRFPLSVKVTPSMAPVSELLLYYV-R----SDGE-IVATTYTIEV-GHCFEN----
K----VKST----------WHTDAQIPGSPTQYHVEAAPR-S--LCAISAVDKST----------------LFLSKSESNLMSST-----------
-------------------------Q-----
TFDALKRFHPTPKFYFPWENSRCKSAIGPEEMKEEINHLPQFLRSKRQTITYSKRVNYVD------AVQAF---------
VDFGVIVMSDL-------VLETRPCPWLFMEY---------TALSRQYISTNEYMSMKDNSEFAVAAAAMDSGIGY----------
---VDQNQAQMATLRSYFPETWLWELVP-I-----
GEEGK-ITIER--TLPHTITDWVGYTTCISPTHG-LGI-A-PPTTITAFQ---------------------------------
SFFLDYNLPYSIKRGEIMRFKVSLFNY-MHH--SLPVKIKL----------------------EEMEKIDL-----------------
HLSEPTASFC--------VKPRDNIVHEYILK-----P-RVI--G-----EVNITVTAFVDIDYPEPCGSETVI-------
FTQDVIVKPILILPEGFP--VEETKSALICPKDSSDDSSFMW------------------------ELTLPKD---AVPDS-G-RAYLNLI--
---GDILGP-ALE---N------LDKL-
IKLPKGCGEQNMILFVPNNHVIKYLDAMRINK----PDL--RAKAIRNMEKGYQRELKYR-F--MDGSYSAF-------EEG-
--ESSIWLTAFVLKSFAQAA--------SL--I-HIDKYVLESSVSWIT-MNQLE-DGCFPVIGTVFHKSMKGGL-QE-HGS----
---------SSALTAYILISLLESGVPLSPSVVN---D-AQKCLEKG----------MN-ND---DLYTTVLTTYVLALL----
EHPKANSSMKSLMNRAT----------------RY---K--NLIWWEDK---------------------SKPS----IGLSIEMTAYVIL-
TLLKLGE--------EN---LSEALKAVRWISKQ--------
RNSEGGFTSTQ-----DTILGLEALT---KYAMIV-HHN-----NITDLSVLVTASK----EVDDVYKLQ--DENRVILKQIRL-
PILPT------IVEIFAQ-GEGCVLIQSNLKYNVA---SSTG---SDAFDLSAE---VR---
-------------------------------SVGYGNECSLQEITICSR---YKMADEESNMALLEVGIISGYVPDRASLHSLL--------------
----

--DPSSKVKLFEEDQ-----DIVTIYFNKL-TGQKTCISFRIIQEYFIDHL-
KPANIKLYDYYQQELTVSTNYKI-PSIC-SSAE-PVD------EQLTTPNE---
----------------------------MIIMKQLSFD--ESPMNSS-----------
FVVNNAELAIPDGMEGPIPVYVKLNTYDYKTEAATTTNGLPDLTSTFVTEDQTVP------------------
TVQTEDEEPIRIDTDNPSITNPLMKEVLRIVKILKINETNVPSPTRINQSCPICMDVLPSNISDIYCSANSAV-------------
----
KVAIRRFRKVRLLLDLHISREVKRLRAMIEFTLSPNCSCSPLDNPGSFALIINKDNDFLTSGNQKQTLNDSFYIYGLPLV
SGVPCKLAEIRASCLNEDNIQCTYEDPPAYG------------------------------

>Apis_TEPB TEPB|GB11563|GB11563-RA

---MRK--
--------IFSNLI--FHVILTI-TS-----
LVN---

-------------------QIRYLSVNK---AY------------VCI-------------------------------------
--VYYR-
-------------SAVAFAF-------------------------------------PSAAGSFSSIALPS---
----

------------------KKTKVRKNFPETWLWQTLD-A-----GHEGK-NELKR--NVPDSITSWVLTAFSVNDVHG-LGL-
IKEPQKLKVFR----------------------------------PFFIAMDLPYSVIRGEIVAIQIVVFNY-MNK--NVVAEVLL--------------

--------TNEGQFDFAEISNEIQDVPK-----LELYRKKKVE--------VKANSGSSISFMII-----P-REL--G-----YITIKATANSI--
------------------LAGNSVNRKLLVKAEGET--QYVNRATFLDLRNTKSTS-----INV---------------------TIDIPKN---
AVPGS-E-HIEISAV---GDILGP-SIL---N------LANL-
IKMPSGCGEQNMLNFVPNIMILNYLKNTNQLT----QAV--QNKALRYMEIGYQRELTYR-H--NDGSFSAFG-----
MSDS---SGSTWY------------------------I---

>APIS_TEPA TEPA|GB18789|GB18789-RA

-----------------------------------MW----------IWILLM---

---------------------------FAC-----------------------------------GNAEFWNGLDNNQTGFGLRN--SRNP--------ND-------
-------------------------------NIIIK-----------------EA------------TYFVVASRMVRPGQIYRLDVNVL----YSAL--
PMMIRASIQRN---------------GVEIAANFQ-EVKEGIPETLMMRL--------PSTS-VNGEYKLRVEG----
TYNSLTGGQAFLNETK---LIFSQ-RS-MTIFIQLDKPVYMQRE-TVRFRTIPIDT-ELK--AFNNP--------------TDIYML---
---------DPYRRIMRR----WLSRQSNL-G------TVSLSY-------QLSDQPVFGEWIIQVIAQ---N----------QIEEKT--------
----------------FLVEEYYQTR---------------FEVNVTM-PA--FF-FDN---DPYIYGTVQANYT-SGAPV-
RGNLTLKAHVRS--------LDRAYTES----------------TEP--

VERYFYFDEYYPAWLKVSSYLENK-----------IPVLRFFNGTYHFRYP--MSELLN--YVP--T------
ANGVEITVTATVGERFLDEIISG-YSMARIFNSTTKIRFLGGS--PQVFKPTMPFILNLVASF-HDDSALRPTQLKE-
AVMEIRADIEMKAGGHRTL--------------------ETQYLKHLQD-----------------------------------NEGIWSTRIDLRKQL---
---GLDHNADQAQQILN------------------------------DISSMKVFA----YLTDG------E-GF------RTQTELLLLAHESP-NQ-
QH------------IKI--------STSTEKPKV-------GEYIIFHVQTNFYI-DT------------------------FNYLIMAK-------
GIILLTGQNIM---EN---NIKTFAVPLSAEMAPVATAVVYHI-G----QYGN-
VVADSLTFSV-NGISRN----N----FTVF----------INNKKARTGENVEIAIYGEPG-A--YVGLSGIDRSF-----------------
FTMQA--GNELTYA-----------------------------------N-----VISKM-AHFD-EDTNGTH--SHTWLY----HE---------G-
DPDEIVYFPSS-TFGID------ANRTF---------EYVGLIVFTDA-------FI-YRRP--
-----------------D--NCNVTQGYGECL-----SG-RCYILDKK--CDGVYD----CDDGTDEAACEFK--------
NATDIALFRKWRFNRLKRQYE---NVWLWKDIN-I-----GPHGRHIFNI---DVPRRPVHWMVMAFSMSPSMG-
FGM-LPKAIGYMGVL---------------------------------PFYINVEMPTHSKQGEQIGIRVSVFNY-LCH--NIEAVVVL-----
---------------ADSKDYKFVHV---EDNGIVQSYKPRTSFGEHQFFIW--------IPAQDAAIVYLPIV-----P-TRL--G-----
DIKVHIYATTV---------------------IGRDSVTRNLHVEADGLP--QYRHQSILLDLSNRAYVFQYMHVNIT------------------
-ETPIIPYDEN-RYYVFGS-N-KAMISLV--GDVVGPIFPT------
MPVNATSL-MNLPMDSAEQNMFSFAANLYTTLYMRLVNQRN----RTQ--EKESFYYMNIGYQRQLSFM-N--
PDGSFSLFRSDWNQSS------PSVWLTAYCARVLQEAR--FYEWE-NY--L-YIDPEVIAQAVSWLL-KYQTPE-
GSFYEVTWLP--DRKMNSSLN-YEY----DVIT-HR--NISLTAHVLITLQSVKD-LPEGLGT---Q-VAVSAVGA--
VKWLERNLKL-LEERGKPYEIAIVSYALLLA----KASTAGQAFNILARHAR----------------------REGGLTYWGREQVP--
----LPPYKLENQKPFLLPRLPYMYDSENIETTAYALL-VHVARQE-I--
---------------MIEPIVKWLNAQ--------RLTDGGWASTQ-----DTAWAMKALM---DYTVRS-RIR-----
DVSSLTVTVEATA----LPGQTKTLFVNDNNLARLQTIEI-PEAWG------TVRVQAK-GAGYAILQMSVQYNVD-
IAKFQTQPPMRSFDLMTR--ANFHG-
RNQSHISYLSCQKWI---NTNESSRSGMAVLDVTIPTGYIIQQQTLDTYI--

RS---KQVRNLQRARFQE-----KKVLFYFDYL-
DQEETCVNFTIERWFPVANMSRYLPIRVYDYYAPERFNETIFDALPTYTLNICEVCGS-------------------------------------
--SQCPYCPIYNTA---
TMLATSSGFLYSVSLLVIVIRYFRTQEFSIS--

>Anopheles_TEP15 AGAP008364-RA|AGAP008364

--------------------MRRSTMFASKKDPAIRILSTSSSSLLAVCLVL--

--SVALVPAVQCEGHYSIVGAKLLRPNSEYHVAVTNQ----DVSE--
PIRFSLAITDAS-----------------SVIAKQEITLNTGETRLVPF--------AIGDISESSYKLVAEG------LS---GLTFKNETD---
LEYQQ-KS-FSVFVQTDKSIYKPGD-TVRFRVLVLDP-NTK--PLQKA------------DNISVHIN------------DAKANRIKQ--
--WKEGKLVK-G------VFESEL-------TLSTAPVLGAWTINVEVL--------------GSKHNKV----------------------
FEVDEYVLPK---------------FEVTVES-PG--IT-TFK---DGKVKAIIRSKYT-YGKPV-
KGEATVSVSPEFQFHYVQPFAKDVITRKVIP--
-----------------------IDGKGSVEFD--LREDIH--LEG---------DYSRNIVIEAVVEEELTGRKQNA-
SAKVMIYDRRYKMELVKS---DDNFKPGLPYTAWLKVSY-QDGAPVQ-----D-QTNPVEVKQSSFESTT---------------
------SVQNYTLDQN-----------------------------------GMAKLEINTEV---
NSSYINVVG----VYLG----------------QEFYLHGISKAESD-VD-SY-----------IRA--------QVLTEMPLV-------
GKDVLVEVTSTSPM-KY----------------------FTYQLLGR-------GDVLLSNTIAVPESKT------------------------------
---------------QSFKFPATFAMVPRAKLVVYYI-A----PNGD-MVSDSKVITF-DSELQN----F----MKVS---------
LSKEQSKPGQDVEISISTNPD-S--YVGLLGVDQSV---------------LLLKS--GNDITKQ-----------------------------------
Q-----VFSEL-EKYEERSYGFYRRKKRFAWNPHA-----------------------------------EHRDF---------
STVGAFVMSNANDPPQIHPVFFSLPALAAPPG----------------------VIITSARPFVAATALSASSPV----------------------
---ASDPIVVRRTFPESWIWESDE-------GFSGE-KTLQK--
KVPDTITSWIITGFSVNPIYG-LGL-TQQPRKLNVFL---------------------------------
PFFVSTNLPYSVKRGEVVAIPIVVFNY-MED--DQTAEVVL--------------------HNDEQEFEFADVENEVVESNK-----
VELFRQKRLD--------IASNTGKSVSFMVK-----P-KKL--G-----HITIKVTAKTK-------------------
IAGDAVERQLLVEPEGLP--QFINKAAFIDLRAAPELT-----KTF---------------------EVEIPKN---AVPDS-T-RIEVAVI---
--GDVMGS-TIQ---N------LDSL-
IRMPYGCGEQNMLNFVPNIVVLDYLKATNKLT----ANI--EAKAKKFMEAGYQRELGYK-H--RDGSFSAFG-----
ENDK---SGSTWLTAFVARSFKQAA--------NH--I-TIDEGVIDKSLEWLS-DHQAP-NGSFPEVGVVSHKDMQGG----
-SGS-------------GVALTAYTLIAFLENINL-----VD---K-YKNTINKA--IDYVYRNTES-LD---DTYALALAAYALQLA----
DHSSKGLILSKLDTKAT---------------TD---S--DSKWWHKP-----------IPETEQKN-PWYSRP----
NSVNVEMSAYGML-AFLE-----------AGL--
DTDALPIMKWLIGQ--------RNDKGGFQSTQ-----DTVVGLQALA---KLAAKI-TSP-----NN-DVTLTAKINE----
NQEKRMTVN--AENGMILQKFEL-PSAAR------NIEIQAT-GSGFAVVQLSYKYNMN---VTGE---WPRFVLDPQ---
VN---ANTNPDYLHLS-VCASFV---
PSAGQNVSNMAVMEVGFPSGFTADSDTLPSLE--

NMPFIKKVETKDGD-----TTVVLYFDSL-DQRELCPTISAFRTHKVAKQ-KPAPVVIYDYYDNSRIARQFYDG-PKAS-
LCDI-CEN------EDCGE-AC--SIRSQKQRSS--
DSPSRQP-----------TVEGTMQSGSQTVSVSFFTFLLATLLVRMFH---

>Anopheles_TEP3 AGAP010816-RA|AGAP010816

------------------------------MPWYV--RI--------LVVISL--

-----------------------------LGS--
--SWGVLVVGPKFVRSNQEYALVISNF----NSGS-SKVNLMLHMEGFS--------
KNQTSVFAIRKPVDVRR-----FMSRIVSF-------DIPNIASPVDIKLTMVG----Q-RG----FSFHEEEH---LVHRS-KS-
ISGLIQIDKPVFRPGD-LVKFRAIVLDT-ELK--PPARIK-----------S-VNVTIQ-----------DPHQNKIRG----
WPAAKLYA-G------VFENDL-------QLAPAPLLGVWNITVQVG---E----------EQLVFKT----------------------
FEVKEYVLTS---------------YDVQVMP-SV--MP-LVE---HQTLNLTIVANYH-FGKPV-QGVAKVELYLVDDTLDQKK-
------------------------ELT--
MYGMGQVELR--FNELLE--LYEDQQ------D----VRVKLTFTEQHTNRTVVK-EQAITVYKHPYRAQLTKE---
SPQFRPGTPFKCTLTLIY-HDGRPAG-----H-VPFFVNVEGEDVD------------------------HQQTYTTGRD--------------
---------------------GTIKLLMRPTE--LTETIDITV----SEDNS----------------
EFTYTERIEKVHAD-TN-VF------------LKL---------ELKSPIKL-------GKLIRLMVTCNERM-TF----------------------
FIYYVISK-------GNIVDAGFVRPNRQTK---
FMFQLTASEKMIPKAYIFVATV------SQDV-VVWDSLEIDL-KQ-FSN----H----LDII----------
IDEKELKPGQEIELLLKGRPS-A--YVGLAAYDKGL----------------LAYSK--QHDLFWE-------------------------------------
D-----VMQVF-DTFH---ATDQN------EFDVF---------NSMGLFARLSG-------
GNRIGASPTTTERF--
-----------------GSAASRPISRLVAYRTNFLESWLWQNVS-I-----GRTGS-RTVHE--VLPDTTTSWYLTGFSIDPVYG-
LGI-IKKPIEFITVK----------------------------------PFYIVDSLPYSIKRGEAAVLQFTLFNN-LEA--EYIADVTL-------------
-------Y-NVANQTEF------------IERPDKDLSYTKSVS--------VPPKVGVPISFGVK-----A-RKL--G-----
EMVVRIKASIM---------------TG---KETDAMEKVIRVIPENIM--FEKTETRFFSMDEYGKQEFN-----M-----------------
----QLDIPKN---I--ST-V-QIKCRIS--SNLLSP-VIH---N------

LDSL-LDVPSASGAPSMINFIPPLVVLDYLKAVSSTT----THL--IEKATGLLRNGYQLELKYR-Q--RDGSFGNWR-----
DS-----KGSVFVTALVGTSLEAAS--------KHI-T-EVDLTLVDRLFEWLA-AKQHS-SGRFDEEQPITYYSLQGG-----
SRN-------------GIALTSFVLIAFLQNTKA-----SA---Q-HRSIIEKG--IQYVANQLES-IA---DVYDLSLATYALMLA----
DHRQKSSALNKLIELGI----------------AT---N--ETRYWPR--------------------------------DTASIETTAYALL-SLVHAKR-
--YADGLMVMHWLVNQ--------QSATGSFPRTQ-----
DTFVGIRALA---ALSEAI-APQ-----KN-DYTAIVLHGK-----ARKVYKVA--ASEADQEYHDVL-PGDSK------
LVRFSAN-GRGFGMFTVAFQYGID---VRNI---EHGFSLRLV---DQ--
----------------------FSNEAYTLQLQ-VCTSFS---PQLMHTRSNLALVEVNFPSGYVVSRKSLVDET-----------------------
----

-----------------------------------RRNPFKDVEVRYGQ-----TSLVIYYETL-GPEENCFSVTANRLFRVAFH-
RQAYVMVHDTYDEKFRAIKFYQV-PHDG-A-DI-QSY------LD---

>Anopheles_TEP2 AGAP008366-RA|AGAP008366

---------------------------------MFSKGGGMRFGGEVKRTV---

---PDPKDHKDGHYSIIGARILRPNSVYRCVVSTF----DTKS--AIVFRISIAAKD-------
-----------KPIATEEITLNSNESRLISF--------TIDSIPEEEYELVAEG------LS---GLEFKTKSR---LDFDN-KF-
CSVLIQTDKSVYKPGD-TVRYRVLVLDR-SMKLLPAGDS-------------GMMVYIR------------DGKGNRIKQ----
WSNASLGECG------VFQAEL-------TLSTEPVLGEWTINVEVV--------------GLKESKT-----------------------
FDVDEYVLPT---------------YEVTVES-PG--YT-FLD---DELLKVVVNSKYT-YGKPV-AGELTVSVKLASSMCFRRE-----
PTETSICQKVLP---
IDGKTDVEFN--LKEILS--SKT----------YIRELTIEAEVCETLTGRTQKG-STTVQLHDERYQVRMIEE---SSYF-

PGLPYNAWIQVTN-LDGSPVQ-----D-GAKEVEIVLRNYNIDL---------------------HKQSSTLDDK---------------------
-------------GMAQLNVKLDEL---DFDYVSVEV----KYRG-----------------
KDYYVQGITKPRDY-EE-AL------------MRV--------RLSEKEPTA-------GKDLTFDVACTKPL-QC----------------------
VSYSLLAR-------GELLAGGAVKGSEAST---TISITIPSTFAMVPRAKLLVHYI-
S----SAGY-IVSSYDTVEF-KRVFEN----Q----IQLT----------LSKDELKPVETLDIDIRTEKD-S--FVGLLAVDQSV---------
-------LLLKS--GNDISRD-----------------------------------E-----VVQQL-EMYESAQNYHWDAY------------------------
---------------------STSDC---------QSVGAVLLSNR-------FIPRDIFPQARLFA-----------------------
CSTSAGGFGAAPMMAACKMK---
GVIMESEMATAPVNEPTVRSKFPETWIWESISKC--------KEMESIRK--IVPDTITSWIITGFSLSKSHG-LGL-
VDNPSKVNVFM----------------------------------PFFLSIDLPYSVKLGETIRIPVVVFNY-MDE--DQLADVIF-------------
-------Y-NNDDEFEFVSDTKDQ----------KEKHRQEQIT--------VPRGTGKTLTFVLK-----P-TKV--G-----
HVTLKITAKCA--------------------LAGDGIERQLLVEPEGLP--QYINKALLVDLRLVKEIK-----QPF---------------------
EVEIPVD---AVPDS-T-NVEVSVI---GDVLGS-SIE---N------
LDSL-IRMPFGCGEQNMLNFVPCIVVLDYLKACKRLT----VEI--ESKAKRCMEIGYQRELTYK-H--QDGSFSAFG-----
ESDK---SGSTWLTAFVAKSFQQAA--------KH--M-TIEEDVIDSALGWLS-KVQTA-DGAFPEVGTICHKDMQGG----
-AGS-------------GMALTAYTVIAFLENPKL-----GE---K-YKASVDKA--LTYVKEHISE-LD---DVYAHALAAYALQIA----
DHPLKNEVYASLLSKSN----------------KQ---G--DIQWWSKE-----------IPEKNDSNCCWWYRP----
CSVNVEMSAYGLL-ATLEASS--------AGL---EGLPIMKWLVSQ-
-------RNDKGGFESTQ-----DTVVGLQALS---KMAAQL-SSS-----EA-DMSLKVIITG----EQEKCLQVN--
GGNILVLQKHEL-AANTR------KLEMIAT-GTGCALFQLSYKYNIK---DVDN---SPRFTLKPE---AK---------------------
---QGSIKSCIDLS-ITTSFI---
PKEDQAVSNMAVMEVDMPSGFIVESDTLKQLK---

QHEMVKKVETKRSD-----TTVVLYFDNI-GEEAVHLQMSAFQKHEVENA-KPANVIIYDYYDNTRCARSFYE----------
----
----------IAV---

>Anopheles_TEP1 AF291654 Anopheles gambiae thioester-containing protein I (TEP-I)

-----------------------------MWQFIRSRI--------LTVIIF---

-----------------------------IGA---
---AHGLLVVGPKFIRANQEYTLVISNF----NSQL-SKVDLLLKLEGET--------
DNGLSVLNVTKMVDVRR-----NMNRMINF-------NMPEELTAGNYKITIDG----Q-RG----FSFHKEAE---LVYLS-KS-
ISGLIQVDKPVFKPGD-TVNFRVILLDT-ELK--PPARVK-----------S-VYVTIR-------------DPQRNVIRK----
WSTAKLYA-G------VFESDL-------QIVPTPMLGVWNISVEVE---G----------EELVSKT-----------------------
FEVKEYVLST---------------FDVQVMP-SV--IP-LEE---HQAVNLTIEANYH-FGKPV-QGVAKVELYLDDDKLNQKK---
-----------------------ELT---
VYGKGQVELR--FDN-FA--MDADQQ------D----VRVKVSFIEQYTNRTVVK-QSQITVYRYAYRVELIKE---
SPQFRPGLPFKCALQFTH-HDGTPAK-----G-IT--GKVEVSDVG-----------------------------FETTTTSDND------------------
---------------------GLIKLELQPSE---GTEQLGINF----NAVDG-----------------
FFFY-EDVNKVETV-TD-AY-------------IKL----------ELKSPIKR-------NKLMRFMVTCTERM-TF------------------------
FVYYVMSK-------GNIIDAGFMRPNKQTK--
YLLQLNATEKMIPKAKILIATV------AGRT-VVYDYADLDF-QE-LRN----N----FDLS----------
IDEQEIKPGRQIELSMSGRPG-A--YVGLAAYDKAL------------------LLFNK--NHDLFWE--
--D-----IGQVF-DGFH--AINEN------EFDIF----------HSLGLFARTLD-------
DILFDSANEKTGRN---
-------------------ALQSGKPIGKLVSYRTNFQESWLWKNVS-I-----GRSGS-RKLIE--VVPDTTTSWYLTGFSIDPVYG-
LGI-IKKPIQFTTVQ--PFYIVENLPYSIKRGEAVVLQFTLFNN-LGA--EYIADVTL------------
----------Y-NVANQTEF-------------VGRPDTDLSYTKSVS---------VPPKVGVPISFLIK-----A-RKL--G-----
EMAVRVKASIM-----------------LG---HETDALEKVIRVMPESLA--QPKMDTSFFCFDDYKNQTFP-----F------------------
-----NLDINKK---ADNGS-K-KIEFRLN---PNLLTM-VIK---N-----
-LDNL-LAVPTGCGEQNMVKFVPNILVLDYLYATGSKE----QHL--IDKATNLLRQGYQNQMRYR-Q--
TDGSFGVWE-----KS-----GSSVFLTAFVATSMQTAS--------KYM-N-DIDAAMVEKALDWLA-SKQHS-
SGRFDETGKVWHKDMQGG-----LRN--------------GVALTSYVLTALLENDIA-----KV---K-HAVVIQNG--
MNYLSNQLAF-IN---NPYDLSIATYAMMLN----GHTMKKEALDKLIDMSI------------------SD---NNKKERYW-G------
----------------------------TTNQIETTAYALL-SFVMAEK--
YLDGIPVMNWLVNQ--------RYVTGSFPRTQ-----DTFVGLKALT---KLAEKI-SPS-----RN-DYTVQLKYKK-----
NTKYFNIN--SEQIDVQNFLEI-PEDTK------KLEINVG-GIGFGLLEVIYQFDLN---LVNF---EHRFKLDLE---KQ---------
---NTGSDYELRLR-VCANYI---
PELTDSQSNMALIEVTLPSGYVVDRNPISEQT---

TVNPIQNMEIRYGG-----TSVVLYYYKM-GTERNCFTVTAYRRFKVALK-RPAYVVVYDYYNTNLNAIKVYEV-DKQN-VCEI-CEE------EDCPAECK--K--

>Caenorhabditis_Tep2 gi|74959685|sp|O46015|O46015_CAEEL Hypothetical protein

--MRLL--------ILNILF---VVW---QIHGVIGQSTN-----------------AAVVSTTAAPVKPATYMLVAPAVVRPDQPFSVCMNLL----KQATDEDMIVRIEVRTER---------------NETIAARVISNLKPGIAQTVSLSE------MPAQSLTPRQSYKLYIRG----E-TL-NAELIFENENE---LKYDQ-KA-LSVFIQTDRAIYRPAS-LVRYRAIVVKS-DLK--PYVGNA-----------T---IKIF------------DPSRNLISQ----TIGVTLDR-G------VYSGEL-------QLAEETLLGDWFIEVETS---N----------GVQDKSS-----------------------FTVDTYVLPK---------------FEVNIKT-SS--FI-TIN---D-DLSVFVDAKYT-YGKGV-AGKAKVSLE----LPWHRWHAMVPTIIDENGVKKEEELMVERTVKLN---RQGEAAVVFS--NDELKR--HKLLHE------WGGGSIRIVASVTEDITEIERNA-THQISTFREEVKLDVEKQ---GDTFKPGLTYNVVVALKQ-MDDTPVK-----A-TLPKRVQVSTFYNYPYNHD-----------TSSLQEEKETKIVEVDAH-----------------------------------GTSVLTLQPPI--NCTSARIEA----HYDIGGKDNFTAT--------PIYSSLYVEAAVSP-TK-SF------------LQL-------LADNEGAVDV-------GKSLSFSLKATQPL-ST-----------------------ITYQVMSR-------SNIVVSQQMTVNSEHA--TISFPATANMAPKSRLIVYAI-I---ESSQE-VLVDALDFKV-EGIFQN----Q----VALS----------IDKQAVEPGQNVKFKVTSDKN-S--FVGLLVVDQSV----------------LLLKT--GNDITRE------------------------------------K-----VEQDL-ENYDSNNVGGGFGGPRPWEAIDRKKRSIWRPWWG--------------IGGSD------AQSIF---------SNAGLVVLTDA-------LLYREPQREFMSER-------------------------RLNTPGGLTVMMMDGAPGM-------------AEAAFAAP---PMGGSSPPPPTVRKFFPHTWIWSDLN-------STSGE-VEMEI--EAPDTITSWVASTFAINEENG-LGV-APTTSKLRVFR---------------------------------PFFIQLNLPYAVRRGEKFALLVLVFNY-MEK--EQDVTVTL-------------------KYDKDSGYDLLKKDGTVVRRDEV----GQQNVRIVSV--------AGGGTSKAVYFPIV-----P-SSI--G-----EIPVHISAIAS-------------------QGGDAVEMNLRVDPQGYK--VDRNIPFVIDLNNNSSD-FS---KNL--------------------ELIWPND---VVDGS-Q-KARLDVI---GDMMGP-VLN---N------AHKL-VQMPYGCGEQNMLNLVPNILVVKYLRATNRNE----SQL--ETKAIKFIEQGIQRELTYK-R--ADNSFSAFG-----DSDK---AGSTWLTAFVVRSFHHAK--------QY--A-FVDPNVISRAVAFLN-SQQME-SGAFAERGEVHHKDMQGGA----QDG-------------GVALTAFVLISILENG------------------MENGKAVTYLEKHLDE-VS-G-NAYTMAVVAYALQLA----

KSKQAGKAFENLKKHKI----------------VE---KSGDVKFASAQ----------KKVEKLKESRAYMFQA----
RPVDIETTSYAVL-SYLAQNQ--TSESLSIIRWLVSQ-----
---RNELGGFTSTQ-----DTVMALQALS---SYAAVT-YSD-----KH-TSQVTILNGK-----HTHSFDIN--IRNAIVLQSYQL-
SSLND------AVSINAN-GTGVVFAQLSYSYYRD---SLND---DAPFFCSQE---IK--
----------------------------------EIRAGNRLQLD-LCCNYT------RPGKSNMALAEIDALSGYRFDAEQVHTLT------------
----

--SIEDLQRVEMEKDD-----TKMNVYFNPL-GGRPVCLSLYSDVTYQVADQ-
KPANFRLVDYYDPEEQLKMTYAA-KQTR-SLQE-KCG------EDCWPPIS---
---------------------------------PSLPPFDEST--VTEIY--

>Swiftia_C3 gi|27357203|gb|AAN86548.1| complement component C3-like protein [Swiftia exserta]

-------------------------------MKMLRAL--------IGFALL--

----------------------------LCL---
--------------------------------------NQCYAAKYFIAAPNLLRVGVEETVSIAVF----DVNV--DVNVQLALQDFP--------
NRRKTFSQVSGNVRA-------QQPGILKIKVNAKDLHDQQSLDKQYVYLIASS--------STAGFQFRDEIK---ILVSY-RS-
AMVFIQTDKPIYNPGQ-TVNLRVVPLSL-DLK--ASVDN-------------VTIEVM-----------NPQGIRVER----
WSNLNTKA-G------FFSRRL-------DLSENVLLGLWTISALYG---H----------GKVQNAS--------------------
IQFEVRKYVLPT---------------FSVKLKG-PS--YI-LES---DPSITIKVTSKYT-YGKAV-
IGSVRVNLAVLDDAGKVERFSTSIHTL---
---------------------RNGEADVIVS--TDLLKA--HAKIPWF-----
PDGKRLVIEAKVIEQATGHEEKALDNTIYFTNTPLKISFKRS---PRFFKPGVPFEIKVDVKY-MNGQPAN-----E-
IPIQIDAKTNDGTVVRERLAAG----------------QVGGDKTNEL----------------------------------GHGRFVVDIPK------
--TFTIAHLVV----KVRATISQGGKDI--------ISEGRFQPSKYRSS-GN-NY-------

-----LFV---------RFLTKPKV-------GQTVDAEAFALSEG-KPNS--------------------LTYMVIAN-------
GKVVFQGQINRDLGVL---TTVRIRVTSAMIPQARFVAYYR------VNNE-
LVADSTIMEV-EEELPN----Q----VSFF---------GDQHSQKIPGDSHAITIQSSPH-S--NVGILAVDQSV---------------
YLLRN--DKHLTSD-----------------------------------E-----VYKRM-KSHDLGCGS--
GAGAD------NKDVL---------NRGGLAVMTTI-------NNLKTDTRAEYSCA---------------------------
ADGKRKRRSTDASVDPQCCILGEDLDPATCLVRAMKFSVSNISSS-----FHSLDACIIEFYKCCYR------
KFEMDWRTRSGEIAIPNNVLDELPFEDEEILKLTLDEAQVRTNFPETWLYEHMK-A-----DKDGR-VSFRV--
TVPDTITTWIMQAIAVSNTTG-FGL-T-PPFNLKAFK---------------------------------
SFFVSLKLPYSAQRGEQVSVIATVFNY-KDQ--AEMVRIYL--------------------FKKPNDDFCTYSNY-------------
GSGSSLYEVL--------VDAHGATSVSFPIV-----P-TEL--G-----DIPIQVKIISR----------------N---
FDNDGEQRILKVVPEGIE--RRETHSVVLDPLDVLRDPSDAKPSAAPTTPSKIQSSPKGNGEQNNRLSLKLPKS---
AIPES-E-YAMLTVI---GTLIGP-SVS---NIIGGRGLDSI-
IKMPTGCGEQTMLKLAPNVFVFNYLRSTKQVT----QQI--EATAFNFIRSGYQRELNYR-R--SDNSFSAFG------NSR--
-AGSTWLTAFVIKTFCAIK--------KLDGI-DIDQNVINTAINWLS-SRQRA-DGAISESSPVIHQEMNGD-----ITG-------
------DIAMTAYVVTAFLECESVAPNSVQT---------VKRA--VAYLENMQPN-VG---
RVYVKAVIAYALALADSPLEVKRQSRTVEQCSLLCR---------------------QEHRRYWHRR-------------------------
SGGNAIEPSKRTSYAL---
ANTNGSEQAWLRRSYRCLADGTKRGGGGFISTQ-----DTCVALQALA---AYSEKTGGDQ---------MDLRIEVST----
DGDYKKTLIVNQKNALVQQQLDISSLIGD------ELFIKTK-GSGVAQLQVETRYNTP-----PTEKEVCQFDLRVI-----
TIERARRMYDQPINDAPKPTKAPKKKKNRPGKGKGRKRNRNKKCRRKNGRRCSGCKGRRCRKPKPTTAAPQVTTR
PPPEDGPVPNSVSIKICTRFK-----KAGASAGMSIIDVGILTGFSVKQESLVELQ---

------------EKVKPGISKFEISD-----RHAILYIDEIPSDHELCFNLELTRDFSVGIV-
QPVPVTVYDYYEPDNKCTKFYGPEPNSLLNLAT-CEH------DTCKCALD--
--------------------------------KCSSCKTSDD--------------------------
SAVVKGLFCTTYDYAFKGKLLIIDEEDQWLHLTFEVVEVYKESVTKK---------------------------
ITKKTARIVYSKKISCDCPVFAGKIDRHFLIMGKDVGLRGSSKVVLGHNVFVKEWPMNDPV--------------------------
----DFFKKFVRLLRKDGC--

>Branchiostoma_C3 gi|13928544|dbj|BAB47146.1| complement component C3 [Branchiostoma belcheri]

---------------------------------MIRSL--------VCVCLL---

---------------------------FAA--
-------------------------------------VLGQQGPAYFISVPNLLKVGTEETVSVNVF----NVAN--PVRVKVYLQDYP--------
DRKTTFSEAEVDVNQDEPSLVTVRVNPDNL--------PESRATKRYVYVVAKS--------DDPQLTFQKEAQ---VLLSY-
QQ-GYVFVQTDKPIYTPNQ-KVKMRIMPLDQ-DMT--PASQP-------------VKLEIL-----------NPQGIIVER-----
KTFPGSATG------FIAETF-------DFPAFPLFGNWTAIAHYG---P----------EMQLNVS--------------------
TQFEVKEYVLPT---------------YGVRII--PSNPYI-LPQ---DDVISGEVEALYT-YGKGV-
DGFLDVKFGIIDLEGNRQLFAQLQTEV---
----------------------NGGFGFYEID--TQRIKD--LDLWF-------
PEGSRLYLEAAVTEEAGGLREMAVLTSVRFETSPFRIGYDLT---ATHFKPGLPFLVKLTLTY-PDTKPAQ-----D-
IPVRVSATAIIPGQDPIVILGRNN-------------EHNSDTTNQY------------------------------------GQASFTVDVPP------
---GTQTLTVTA----KTEQVGLPVAH----------QAQENFEATPYQSP-SG-SY-----
-------LLV-------RVLQRGPVPV-------DEAIDVEAVVTKQN-DIQS--------------------YNYMVVTR-------
GQVTLQGKIVRQGGVL--KTITFRTSAVMAPISRLIVYYI-N----LQGE-
VVADSTLLEI-ENVCRN----K----VMVS----------STEDVEPQEQANIEVNADPN-S--LVGLLAVDQAV---------------
YLLNN--YNRLTSQ------------------------------------K-----MFQAM-AKYDQGCGP-------------------------------------
-GGGQD------SANVF---------KDAGVTVLTNT---QLSPAVRSNAGCGGQSRR-------------------------
KRDLQNSLEAKVMEFNETLQPCCMDGQQWDP--LGRSCLQRAKLNSTS-----QD---
ECYFAFLTCCNHARSLRRLGRGRGRMGGGGGL------------LDIDIDEDESQLVARTEFPETWIFEDVQ-V-----
DDRGQ-AVVPV--TVPGSITTWVIQAVGISTANG-MCV-A-KPFRMKSFK----------------------------------
KFFIHLQLPYSIIRGEQVAIRATIFNY-DQQ--DLRVNVYM--------------------QGVEGVCSGARA---------------
GERSERKTLF--------IKGNDAASVLFPII-----P-LEV--G-----TFPIRVVAFST----------------A---
AGGDIIEKSLQVIPEGVE--RRLVRSIFVDPKGRARDRKREGEEEV--ALPTEHDVDPDNGLQFDVVDVRLPPE---
TIEGS-E-QCAVSIM---GDIMGP-TIT---TTIGG--LGTL-
LRLPTGCGEQTMIKLAPNVYVLSYLHCTDQIT----KDV--EEKAYDFIRQGYNKQLSHR-R--PEGCFSVWG-----
QNNRY--PCSTWLTAFVNKVFCQAK--------KF--VTSIDEEAVCKATEWLL-STQRE-DGAFKEVYKVHHREMTGG---
--VQG-------------DASMTAFVLISLLENCECPIAE--------RSIAIERA--TLFLERQLEQ-LK---RPYVIAIVTYALHLA----
DSPLKGAANEKLRSIAK-------------------YDEGTNSRYWEAD-------------
ASSIADGQQPYWYTRKPSAIAVETTAYALL-TQMHIGD--
---IQYSNPIVVWLTQQ--------RNSAGGFVSTQ-----DTVVALQALS---SYCGSTKVDP---------TQFTCQMTS--
DNDLDYNEEIHVDKDNALINQEKTA-PVGG-------KLFLSTS-GTGIGQMQVEVRYHTP-----
DVHRERCLFEVVVTTEEAE--------------------
GPVEPDEEPEEGQEGDEDYPDYDGELDARERSAFSSRSRIGGLLSSGRSRSRIARQAEDDQSQHFYLVRVCTSYR----
-GQRGASNMAIMDIGMFSGFEPVKQDLEQLL---

NRAGKKAVQRYETTN-----RAVLLYFDEI-TSEEICVSIRMKRVMLVGAV-
QPVPVSVYDYYQPDEACTTFYHPGQGSPLLATL-CDG------SQCVCAEG--
----------------------------------KCPKETSPKK---------------------
LRELTQRDLEEKACNDLDYAFRVIVMEVRQEGSFDRISMIVESPIKKGLDDV---------------------
FDGEERVFWKRKTCSGLQLVEGTTYLLMGKDGTKY----TDEQGFDSFRYVITEQSFVAEWPTGAKAER-------------
--------KKFKRVADKFQQLADKIFLQGCTT--

>Strongylocentrotus_C3 AF025526_Strongylocentrotus purpuratus complement component C3 (Sp064) mRNA, complete cds

----

---------------------------------MGSII--------LIFVLA--

-------------------------ISA---
---------------------------SLVPGGVTQDPPATFFVISPNVFRAGVEEKVVVTLI---RSPPNVQNIEVKVSLMRPG-------
-------STVTFSEDTRLVSPGSSQSFSVVI-----QAGDLMRNEGAFQHMVLKA------ESLNPIYPFEEQTD---ILVTL-QS-
GYVFVQTDKPIYTPNQ-DVMIKVMSLDQ-DML--PSDRE--------------LHVEIM------------
DPSGTSVKRLRETHADVPAS---------IGRLQV-------HNHPASVNGPFRLSMDP----S----------SLSSQVT--------------
---------FVVKEYVLPT---------------FSVSIET-PK--YI-LAG---ADSVVSTIIAEYV-FGKPV-
IGRYTFKYGVVDNGTVQELGTKQGLLG--
-----------------------DSGRAEVTLD-------D--
LSTDFGEDWFVRFRGRHFHANAIVHESATQFSEASINTKAIFVDSPYKFSTKRT---VQHFKAGLNLQVKLDLTF-
ANGDVAP-----N-VPVNVIATATLRDGSEELLRRPDIAGG-----------QEEISNTDDQ-----------------------------------
GGVSMVLNVQS---SITSIHIVA----ATHDPQYPNN-----------
QADISFEVSPGVSA-NGHDY------------LVI-----RPQNTDDRNLNV-------DVSTDFLIQRIGTT-GDQDID-------------
------LHFLCITG-------GKVVLEGVQRSISAAG---
TNLAITIRAYMAPQMRLIVYYV-T----MDGS-VIADSLLLGV-EEKCRQ----D----PQLS-
LDILPRNVGPERDVYEPNGQIQVEVTAPID-S--NVGLLAVDKAV----------------YLLRD--KDRMGKQ-----------------
-------------------K-----MYERM-RSYDTGCGP---------------------------------------GGGQN------TAQIF---------
KDCGMTVLTNA---GLDVPIREDVECMDEDTR-------------------RKRSIDRDQLCLYDPTYLADCLAD----

KPRLRRVLTEGGWLCPRRARVLEAE-----CNLEPDQTQVYRDCCINSMNP--------------------
VVTATSRSGGDGGEQNAAVKVRDDFRETWFFDVVS-M-----PEDGSPYLYPV--SIPSSITDWHLTAVSLSPTQG-
MCV-E-DETTVSVFQ-----------------------------------DFFIQLHLPYSVVRLEQTQVIATIFNY-GFS--DFEVSVNF--------
------------TVDQGLCT-------------------AENPAIRHVF--------VESKRAASTTFHCT-----PCGSR--G------
IPYTVTAGGS--------------------KKRDSVRNNLRVVSQGVM--QRKSRSLTLNPGRVMFSDDVTTPSPN-
NSLGSGPGTIFGEGFQHEEIAISLPGS---SIPDT-E-SCSVKLI--
GNLLGTASTDPIGG------LDHL-VRQPRGCGEQTMIYLAPTLFVYQYLIAVGSDT----AEQ--
EARIYDYIADGVARELTYR-Q--DNGAYAAWKHR----------PGSTWLTAFVVKVFSQAN--------RF--T-
RVEPGHVEGSINWLIDNNQLP-SGAFQESQQVIHQEMIGA-----VKG------------
ETSMTAFVLISLLESRNLLVPANQK-----IDEAIGKA--TEYLVTQVEN-ID---RVYDKALVTSALR------
ESASVGTANGKLWEDRN-----EDGTGAVSFTPDDANYEDGSQPFWLQRKP----------------------------
SAIEIETSGYALL-AQLALLD--YQKAGKIALWLSKQ-----
---QNDGGGFVSPQ-----DTVVALQALA---KYTERPEFNT---------IEMNCDVAT----
EQIPLHRYHIGNDNAKVQEEVDVSPSIGR------SLTFDSR-GTGVAKANVELRYNTE-----KSDIDTCPFHLNIS---------
---AVEVPSDEVSGQNVKGLMITVCTSYN------
GDGTTHMIIVDVGLYSGFKAVEEGLTGLK--

---QVVNGSSLISSYEASS---
--RSVIFYLDTIPSDEDLCFTFSAESDVVVGNV-QAAAVHVYDYYDPEKSCTIFYKPGDGSALLSTL-CSE------
NECICSGG--SLEYCNIDPC---------------------
PGPYTRIDLEGTACASHSSYALKIRIDEVEIKEGFRICKFTVLNPIKTGDEDVP-------------------
HQAQRQLFINEGCDCPKVKGRNIGGTFLLVGQKSLK----YTTEQGEERYRYVYGPTSKLEFWPATRKA----------------
--------RNAAVFDKLVAFEAQMAPASACSNEN---

>Carcinoscorpius_C3 AF517564_Carcinoscorpius rotundicauda complement component 3-like protein (C3) mRNA, complete cds

----

-----------------------------MESRILLFTL--------TVIFLC--

-----------------------------IRI---
---EAANIFVASPNTLRIGEQETISVLLE-------GNKAETVEVYLQDHP----------
GKTKTFSKTVGRVEPRISTEFKVQVNPEDL-PDKDILVATAKHYVSLVAKA-----------GNWFHKETL---LLVNP-RS-
GYVFIQTDKPIYTPKQ-TVHMRIITLNE-DLI--PQNKM-------------ITLQIK------------NPQDITVEH-----
HSWKVGKVGRRIE--FYTFDY-------SFPPYPLFGEWSAIVSYG---Y----------DLLYNTT--------------------
VKFEVKEYVLPT---------------FSVDVTA-PE--II-LES---TKKIIGSAHAKYV-YGEPV-
HGTANFKFGVKLNTGDIIIIGTLYNKQL--
---------------------QDGKVDYQID--VNEFMK--HGKISGFPD---
LEGNHLFVEVSVLEQATGQRESGINENGIFTLSPYDVSFKRC---LNNFMPGYTTLIPVDINF-VSGKPAP-----G-
IPAVITVTDEKGRAVVV---------------------QKSEDTSDER-------------------------------------GRCNFLVNPSK-----------
---DLKEIKIEV----KTNDGRGAQY-----------QRKGQHRMGEQSSE-FG-GV----------
--IAI------DRGTTKKDLKV-------NEEFSASVLTNPAG-GISL---------------------ISYMVISR-------
GKILIHKFLPKVELIG--HKIFFVVNTDMSPSFRLVVYAV------YKGH-
LLTDSILYNV-EPTCKE----S----VKFK-------LETDVIDRPKPGQLVKINIVEATKDT--KIGLLAVDEAV----------------
YILRN--KDRLTRE-------------------------------------K-----MFQEM-EKHDLGCGP---
GGGSN------IQSVL---------ANAGVAILSST---TLTNYKREDYICAVRIRK-----------------------------
KREIMEEIVKKYEKRDKQCCILGMKHDP--DQRSCEERHAIFEKY----DFDGKETCMAAFLGCCNEKH----
LYLLKNIEKEGRGRFGFDPADQLITVGLEEEQELLKQLNVRKDFRETWIFDDVY-V-----GPKGR-VEKEL--
SLPHSITTWVVQAVGISNTGG-MCI-A-EPLKITTFK----------------------------------
SIFVQLNIPYSVVRNEQVEIQATVFNNHAHQ--SVRASVYM--------------------YGVKGLCSGAEE---------------
GQRTERKVLD--------IGRSSAKSVSFPVV-----P-LKV--G-----EFPVRVVVFTV------------------
HGSDFIEKKLNVVPEGAK--DSKLISLQLDPTNQQKRQKRSIHEKY-----YIDSIDPEKKMQISIVELQPPPN---YVPDT-
A-KCLVSVI--ADRFGPVVETALEN------TEKL-
IQHPRGCGEQTMLFMAPTLYTVKYLKVTGQLN----ANT--EKNGYKFIRDGYSRELTFR-K--NDGSYAAWQNR------
----PSSTWLTAFVMRVFCQAQ--------KL--I-EIDEKVICSGMQWLV-QRQKP-DGSFVDEKPVIHQEMIGG-----
VKG-------------ALPMTAFVLMALHECSCTTVPG--------LKLAKIRA--AAYLEIKVPH-IQ---DPYIMSLVAYSLSLA----
GNNAKVEANRKLLSMAT--------------------FQADKNYHYWGDP------------------------------NSPRAIETAGYGLL-
VQILNND--IEYANSIVNWLNSK--------
RTLSGAFKSTQ-----DTVIALYALS---EYSILA---------KKPETDLQCNVTL--NNDPSFFKELHFKESTANILQQFQI-
SNLGG------HLIFNTT-GYGMGQLAVELKYNVP-----
VPPEKLCKFDIDVKVNEVKEEIQQVIDPRMPGNDVFDLLPDALLRNLGFPKRKERSLSDNIQDYARVKRDNRGRVG
DGGRRG---------------NDGKSKLLLEIQICVKYL------SHVDSNMAIIEAGIFTGFKVLIDELKQLV------------------------
----

--------------------------------KEKNSKIARFEASD-----KSVVFYMDSAPHDKPYCFKFRTVRQFIVGNI-
QSSVVKVYDYYKPNESCSQFYSPDNQSPLIRTI-CEG------SVCQCAEG---

-------------------------------GCPPRHPFQG-----------------
VTQIHDISESRKLLLDRACVDHDYVWKGTVESKRKENGFRYISFRVTSVFKEGIEQKQ-------------------
NILHTSKDLMVRDSCSVADLDIQQEYVIMGRDGAQF---KDEDTGILLYRYILDQSTSIFKWTRISVAE------------------
-----NKQLTKAFRWLEKHMVMGEGGCPQ--

>Halocynthia_C3 AB006964_Halocynthia roretzi AsC3 mRNA for C3, complete cds

----

--------------------------------MISFHI--------YICILF---

-------------------------FLT--
--------------------------------RAKLTVSCSYSLVLPKALRVDTENYAYIDFH----NCPP--IAKISTSLHSLP-------------
GIENTHSSDEKVDHPTIDPIKLTYVPKSKMMENRKKTTTPYKKALVVIN------YCFGVNCQFQNPAEKFTIPLNS-TH-
GYVFMTTSRPAYRPGE-NVHISVAGLAQ-SFTAARGDLPL-----------PDVTIKIK------------
TPISLGRMSVQDTLKRMDNEPTG------MLRHTY-------EIPEDPMTGVWWAEAVMN---N----------EVMATTS----
-------------------FVIDKYVLPT---------------FDVKIEMEQH--FI-LPG---QEIINGEIRAHYS-YGEPV-
DGRYYLSCILKYGIVGTEKEFFKIPKSPFGEAII---
----------------------SGGKKKFSIS--TSKILE--ATNFQSLEDFIALDGK-
VIILATVTGRAQAVYESDMVDDIIFSRTPYIIDMSRS---AKFFFPRKIYEIKAIIKDVVSGHPIS-----G-VPVTIKAGDA---
-------------------------EAISKRSGDN-----------------------------------GEIYHAVNLNS--------------------------------------
--------------QRQQEHILI----STNHDTYKLEE----------QSNITLSVDRYDAK-TE---------------LGI--------
QLKKQFVDV-------GDTVSVELRLGDVS-PTD----------------------IRYYVVSR-------GEIVYAQTKEITENGD-----------
--------------------------------------VAIDFEVTHKMVPFSRVVAYYF------LNGE-VTANSAWFDV-TDQCLE----E----
ITIE----------PQSQTAKPGEDFKFTVSGPAN-A--KIEFSAVDRAA----------------YFVHN--SSRLTRN-------------------
-----------------T-----LWRKM-EAYDTGCSR--KGGKN------AMSVF---------
KEAGLLYQSANINNQDLHTDVCAPEGGARKKR----------------------------
AANPNRDFQLVAAAKVEKCRLDGQRLNL--IHLTCEQRLAIISANMQHKDENLRNSCMTAFEEACVALENDFYT----
--------------QESVSRSLSANDNANEFRLRKRSDFKESWISEEKK-I-----GDDGT-LVFRE--
NAPDSITIYEIAAFGMSTVSS-FCI-A-KPKEVKVHK----------------------------------
NVFIQLYLPYSVRVREQAVIRFAVFNY-GPQ--EIEVTVKV--------------------LHSKEICTNFIS---------------
DDFRKLVVLT--------IPSRRSATSYFTIL-----P-VKI--PKGGKSKIELKAEGDSS--------------------
ENSDWIEKMLLVEPPGQH--NDSHGTFLLSPDVNGGVIDF---------------------------EIDLATK---NVLAG-
TIKCNLYFY---GHIMGP-NIEVELEGRLAN-LQNL-
INSPGGCGEQNMIRIAPVVYIHAYRSNLEAFTVT--DAQ--RAQTLKYIQDGYAHELEYKTQVPQGWAFAVWANN--
--------PPSTWLNGFVSRVFASAR--------KY--WPGMEVDRICQSVAWLL-TQQEP-DGHFDEDDPVHHKEMDGQ-

----VTN------------DITMTAFICISLYESRSSCPELSTR-----IQEGVNSA--MEYLKNHFDDAKN---
TPYTVAIVAYAFAIWE--PHGQFAQKWNEQLVAMKI--------------------EDEKFVHWQGTRG-----------------------
----RAADIETTAYGLL-AEISLKKVTENN---
YRWTKQIANWLISQ--------RNDRGGFISTQ-----DTVVAIEALT---KYMTMLETAFQK---
ENVDLRVILSSTSQHSSWFENPEVVGITENDGMTRKTVEV-PKDAIDK----PIRARVD-GTGEGSLSYRCTWRQY-----
-ESADKCHFQVEVS--
VDEAQEEDMKVVKMTVSKN-------GTKAQMSIIDVVMLSGFEADKDSIAALN--

------------KDIAADGIFDRYELTN-----NAVKFYLGSI-GEKEIKFAFKIKQVSVVSKI-
QPASVIVYDYYEPDIRCTKFYTVGDPNLKLRTI-CEDGG----VLCKCAEG--

DCPVCRSQNDQLTNTQCSSHGNHEQICSDMCEDNIGCYNLHVETCKRDYVYIIKVESVEETEGSGYKIFKARIVEVIR
STSKHIDPKDDVTSFRIREDCYAESCKDPRFEEIARKKNNAVEAKFIEEGRHLLVYGSNPTVTEAKNEPVQYI----
YELDDNARLEIVPSGEKCSPEILECAPNDIRCNKKKAKLDKITKLCKINSTLIEAHLE----GCDV---------------------------
----

>Ciona_C3 gi|18074013|emb|CAC85959.1| complement component C3 [Ciona intestinalis]

--------------------------------MVWFSF--------SLLVTL--

----------------------------LAV--
--ATAFDHTVVVPKALRVDADEKIIVNLH-------GYNRATITGYLQDLP--------
GLQTFFSRTGQRVLTPAQCQNPIEMTFRVTRDPQGADGIASFGLTQKVRLTIQV--------TNSNSDFTENID---
VLVSK-QS-GYIYVITDRPIYKPND-TVKISAFLLNQ-NMGHQTGVDA---------------EITIQ------------TPDGIGLVR----
ESFVDLQSN-------RLNHEF-------AINENPIYGTWSIEVKFS---S----------DGYTTSS-------------------
TTSFKIDKYVLPT---------------FDVALQLAQS--HI-LTS---DPRITGTIYANYS-YGEPV-
NGNVYLSATLQKLPGGPAIKFYQIPPRITRTALF--

-------------------------RNGVKPFSIP--LVELIG--
ILQPGETLAQMSDQGAVVSIFATVNGEADGVMESAVISNIPILRTPYRIDKSRA---
LKYHTPGISYLLQVDVQDVVTHQNMA-----N-IPVRIEITGPNGQR------------------------VIRNSTANQN-------------
-----------------------GQVNYPHNFD---TTGTQFIKV----TTAKPNLGAAN-
---------QATVNITVEAYNSP-GQ-RF------------LTV--------TPNPHTVDV-------GREVHITLAFNQPT-PAE------------
----------IRFYVVSR-------GSVVLVGRIVPPANSH--
NIVQIIQVTQAMVPSMRVIAYFL------HGAE-VVSNSAFVNV-VNRCET----E----LTVT----------
TNRNTVKPGAPITYTIEGAQN-A--DVLLYGVDRAA----------------YFLYN--GSRLTRN-----------------------------------
--S-----MFSDM-AAYDQGCVS---NGGSD------GPNVF---------
FGAGLTLTTPTHKPGALDTLDCTAAAQSRKKR-------------------------------
QIQVEQELSIEAKLHKCGEDGKKKSFSECDTCEMQRDRVIYTF-----DDAIPGCSERFYAECIALARLNSGTRRQ-------
-------RVQGRSIGVNGQVERSVASAVRKDFAEALYLDPST-L-----EQAGR-RQITA--
KARDSITTYEIDAMASADTPDGFCI-A-PTTNVKVFK-----------------------------------
NVFVQVYTPYSLKKREQALIKLSVFNY-GDT--LVTVDIMM--------------------RAHPVLCTHFRTD--------------
GSYDLVRTIS--------VGPNSAGSASFAVL-----P-LRIPAGDIPKATVEVYITDNRN--------------------
RTYDSVKKEILIEDEGEL--KDIYETFPIDLKNSRVQQTQI---------------------------NFTFPEQ---FVLGT-R-KCMLYAY--
--MDFMGP-AIEVDPVTQEANNVNSL-
IRQPYGCGEQTMIYAGPTVFALQYLVTTGTITPN--SPE--YNSAVNKIEAAFQREMTYRTTHTNPRVWSVFTHY-----
-----LPSTWLNAFVDKVFYHGR--------RY--DTDMNVGPICNSLNFLI-GEQMA-GEHFRERRPPLHREMHGA-----
VKG-------------PMTLTAHVAISMGEINSICLPELNQ---R-VIASRVSA--MNYLEQHKDH--
ATFQRPYPLSLLAYAAALHN--PRSQLAIEMNARLMAMKQ-------------------TSGNGAYVFWR-----------
AKTLAEISGTNAHAYWYRTRPLALDIETTSYALL-AQVAMSKAKDPLVQAD--
-----------------LDLGRKISLWLISQ--------RNSNGGFRSTQ-----DTVIGLQALSTILSWISTVEPGI-----
ANPRIDFEMEGAAGSAWAPPNAYTNHIDNTNQRLKKQMAVPKNVIEGT----IVDVTSR-GQGQGILSYRCTYRTV-
-----VDEESCHYNLSTN---
VRVMNPGVQGKRIKITITVSKP------LGPQSEATILYVGFMSGYKAVDQTLEDLS---

-------------IDENVAGVVDRYEVTN-----HNVLFYLNRI-TSQSMNISFEMRQMVLVRKP-
QPGKINIYEYYEPVVHCGQFYSLPDTTPDLQTSNCETGANNDDNLCRCAEG---
--GCPTCRTRDDTLFKLHVLE-----
VEVGARCGTCTQNRCYNHRDEGCSAKYVYKVRVIGVREDSVFVNLDAVIEDVYRSGSDNVQ-
NGTVRTFVIRNDCHASCRDTDEDLRLLRNNIPAKDTRLFKEGRVLLITGSLIERRQNRNRLLQTV----
YQVDEQTTAERLVTDVACARAKSVLVRCAGNRPPRQSKCVKSRELNAICENMKRLKESLNDIGCD-------------------
----

>Eptatretus_Complement Z11595_Eptatretus burgeri partial mRNA for hagfish complement

----

--------------------------------------VLVIAPAATSSYDDLAVAILMV------DQKKITEVHVLLVNPH------------
TGATLDEKKVKLQWDNKFIAFTKLQV-----TPKEVEKWKEDFVRLMVKW-----------DGGQHMEID---IPLTS-RR-
GLVFAQTDQPIYTPNN-DVNIRLFPVTR-QLN--PILSS--------------LVVDIM------------NPDGVVVDR---------
IEKNAFEVEKVMELRPF-------HVPAITSLGDWKIVSWMK---D----------KPQFNYT---------------------
SGFKVEEYVLPT---------------FDVSIT--SEQPYLHVY----DKAFTIHIKAMHI-YGKPV-
MGRAYVRYGVKHQSKRTLLSTSSALARF---
-------------------------EQGEAMHTLR--QKHILE--QYPDPKL-----
LLGQSLYVEASVISSDAGEIENSILDDIPIVASPYSIKSKWT---VPFFKPGVPYIYKVLVLN-PDGSPAS-----G-
VPIKVSFSFDSSGNW-----------------------ITQKRKTMDN-----------------------------------GIAMQTINTAR-----------
---NSKKLNIKV----QTEDERLEQSQ----------QAEASFTIASYSSP-SG-SF------------
IHL--------NAHREVKSP-------GEHIVFDVFIKSAA-KDHVLH-------------------FNYLMISN-------
GKIHNFLQEGRKGDTT---SVSLLLTPELVPQFRLVAFFI-L----PSGE-
LVADSIIIDV-KDSCHA----K----LSLD--------VAGGKRLFSPRDNVNFDLSGESD-S--WVAVGVVDKAA----------------
YVLDK--KNKLTAN-------------------------------------K-----VYKAM-EASDLGCSV---
GSGKT------GPLVF---------RDAGLAIMAKE---ISGMDDVKDPGCPNGHTR---------------------
RKRELVLEIAIEKASTYPAELRK--CCRDAAIESP--LRLSCEERTKHIHDE-----GE---
GCQETFLECCKHVEEELLIAMEEE-------------DEDLGRSQGEDFMIQESQVVIRSHFPESFMWEIIK---
LSRSAENGK-SRITK--KMPDSITTWDIQAVEVSQSKG-LCV-G-PSLELTVFK----------------------------------
QFFLKVHTPYALKQYEQVELRVVIYNY-MNQ--DVKGEIQV--------------------KCGDGICTDAE----------------
QNEPLKSRFA--------VEKNSATSFSFMVV-----P-LSS--S-----DSSVSVLARVF---------------GS---
DVHDAVEKDLRVMPEGNY--EEMSRSWSVQPRRHGGQQV----IVV---------------------DNETPQN---VVPGT-
EMSAFLSAQ---GNLVAE-TIQ---NTLKGSKISNL-
LRLPRGCGEQNMMYTSITVMVARYLNRSDQWNKMGDPQL--KKRSFDFITSGFASQLTYR-K--PDYSYAAW-------
LHR---ASSTWLTAFVAKVFSQAR--------QL--V-FIPVSEICGSVRWLM-RKQDK-
DGSFLESKPVVHLNMMGQVTG------------------KVVLTSFVFIALLEARESCINEVEG-----FTVVVEKA--
HGYLTSQAMNGL----EDFPLAITAYALSLWK--VSDGAAKVTMHTLKTSGL---------------------QTEELIHWG---------
-------------------SNKGKAAAVESTAYGLL-AAIQHEE--
GEIAEKATNWLSQS--------ATFGGYFQSTQ-----DTVMALQALTGFESCQSRM---------KKMDLSFKIRAEE----
NGVFDKEFQITNDNAFVQKPFKV-PVHG-------QLTVTAS-GTGQGILTFVKKYREK----VVIKKDCKGFSLEIT---------
--TNLDNQVKQRRRQSINPEFNVYRFIGCFRYL------
RNQEPGMVVMDISLPTGFEAKKKDLDDMK---

---NLVDNYIVQYEIRP-
----GRVFLYLDKVNKDEKNCVGFRLNQVFESNLV-LPVTATVFEYYEPDFRCSKSYHPKMEVNPDAS--CHG------
NICNCLQR--HCVELKGMAD-------------------
----EDRNADRNGNAC---RAEYVFIIGVTKVTKTASYININAALKTVLKKGMDQA---------------------
INVGARRSFVIPMHCGKNLNVSPGDIYLVMGMHNAHW-------RNSDRTQYVLTSDTWFEKFPLESVCRLP---------
------------SPPASCQVSENFK-------GCSLKG---

>Lethenteron_C3betachain gi|1352101|sp|Q00685.1|CO3_LAMJA RecName: Full=Complement C3; Contains: RecName: Full=Complement C3 beta chain; Contains: RecName: Full=Complement C3 alpha chain; Contains: RecName: Full=C3a anaphylatoxin; Contains: RecName: Full=Complement C3 gamma chain; Flags: Precursor

----

---VLLL---

---------------------MSV---
--------------------------------GTSVTQDPMVLLSVPSVILIGSDVNVLVDHA------ASTEDVSVVVRAEEFL------------
TKKQLATQTITLTQLDPAIATLKLGF---DIENPDKTNSASTKHHVRLVA------KVESKSFNKEITAH---ALLSY-RS-
GHVVVQTDKPIYTPDE-KVKYRMFPMNREDVHRIPVRQS-------------MTVDIV-----------NADGVIVER---
QIKTIKATDEGI-----VDGTSF-------TIPAISKHGTWKIFARMS---G---------APNINSS--------------------
AEFDVREYILPT---------------FEVKIN--PKQRVF-HIN---DEEFVVDITANYF-NQELV-SGTAYVRYFLEN--
GDVPKLVDSSSTTL---
VAGEGLSILK--KEKLLK--LFPNAKD-----LLAFSLTIKTTVLSSQAAETEEAELVGIKIVESRYQITATKT---
SRYFKPELPYFIQVEVRN-ADGSPSK-----E-VDVVAKVQVGSATI-----------------------NPQKMRTDSN-------------
----------------------GLTSFTVTPP---NVNQLTVTV----RTDERHPSNE-----
------QGELVYTAQKYASA-SY--------------MHI---------DVTRIMRL-------GETLNVFLTAKTTQ-LNAVTH-----------
-------FTYMVLTR-------GVIVKTNRKTKESGGG---

PSNVRIPITPDMAPRFRFLAYYI-L----PGGE-IVADSVTVEV-TELCKS----Q----VSLSL---------
KGRPTLEPKAMLTLDLIGEPD-A--RVGLLAVDQAV-----------------YAVNR--KHRLTQD-----------------------------------
---R-----VWKAM-ETFDTGCTA---EGGAG------RPGVF---------SDAGLALITSK---
GLNTTDRSEIGCPKVPSR---------------------KPRQLSMLQIRREAEKYTQEFR---KCCVDGLKMSP--
TGQGCEERLKRVTGP-----KE----CVDAFLQCCKKAE----EYRKSESLGAK--------
TVLRRNDFMELDLMNEDEVNMMAYFPQSWGWNKYKNS-----CKYGRHPQIRL--
QLPDTITTWNMQAVSISKTRG-VCL-A-DPLLLVSTK------------------------------------
DFFIKLHLPYSVKRGEQTEIRVILYNY-MEE--SLTILTEM---------------------DIVESICSTSKS-----------------
GAKPSQKST--------VKGKGAMVVSFPIV-----P-LKI--G-----EHHISIRSRVY----------------GR---
TFGDGVQKILRVAPEGVRDIRSESRSVHVEERET---------FFI----------------------KNEISPD---VVPNS-DVLTFISVK---
--GDELAETMV----NCLDAKSISNL-
IQIPTGCGEQNMIKMAPTTLTLIYLDSVQEWEKIG-LHR--REEAIGFLKQGYSRELSYR-K--ADHSYAAF-------IKR---
PSSTWLTAFVVKVYSLAK--------RV--I-IVDNQELCGPVEWIIKNRQNS-DGSYREDGPVIHREMQGGVGG--TEG---
----------HVSMTAFILIGIQQAQEYCGVSVPN-----YKQSMNRA--VQFLASKVSD-LK---RMYTIAITRYALALQD--
PESEAAHSSWKKLENRTT---------------------FESKGHRYWK-----------------------AEETSHVLRMSAISVEATAYGLL-
TYLRKKD--YESAREIVDWLTEQ--------
RNYGGGFQSTQ-----DTILALQAMA---QYKMDS-SSK-----ELIDVQLEITSPK-----NNFEKKMKITEETRFVQEPHKI-
PPGG-------NITIKAS-GRGTFTLSIMSVFNKV----APSSKSCSTFDLKVT--
----MTEADDGESPQGRLGWFDGKRRRRRDIGDEGGVEAVYRMNMCTRYK-
PRKEDLSSESGMTIIEVNMLTGFIPDKNDLIQLK--

ESVDKYISNYEITD-----SVLIIYWDKVPSTEDYCFAFKIKQMLRSDMI-
QPVTASVYDYYSPADKCTRLYNLPGGYVELSPL-CQN------DLCQCVEV---
-----------------------------------SCPAKKPKFD--T--------------------SITVLHRQEAAC-
VAGIDYAYVGIVDNRTEVGSFVYYTVNIQTVIKSGQDQA---------------------
IQPKATRLFIVTRSCDGRLGMETPRQYLLMGRKGETK-------DRNDRFQYVLDASSWVEQWPVDEKCNQP---------
------------NVQTFCAIKREYEFSMQIQ-GCSS---

>Oncorhynchus_C3betachain gi|1352103|sp|P98093.1|CO3_ONCMY RecName: Full=Complement C3; Contains: RecName: Full=Complement C3 beta chain; Contains: RecName: Full=Complement C3 alpha chain; Contains: RecName: Full=C3a anaphylatoxin; Contains: RecName: Full=Complement C3b alpha' chain; Contains: RecName: Full=Complement C3c alpha' chain fragment 1; Contains: RecName: Full=Complement C3dg fragment; Contains: RecName: Full=Complement C3g fragment; Contains: RecName: Full=Complement C3d fragment; Contains: RecName: Full=Complement C3f fragment; Contains: RecName: Full=Complement C3c alpha' chain fragment 2

--------------------------------AALQVLSAPNLLRVGSNENIFVESQ-----DHVGGPLNVKIMVKNHP-----------
TQSKELASKSVVLDQANNFQAMTQLVI-----QRGPLVDDPKQKQYVVLQA-------------QFPDRLLEKVVLVSF-QS-
GYIFIQTDKTIYTPAS-TVHYRVFSMTP-GLE--PLTREIFEDQEVAKNKEIAVSVEIM------------TPENITIFR------
EIVNPDKG------VKSGQF-------KLPDIVSFGTWHVVTRFQ---S----------TPQKTFS---------------------
SEFEVKEYVLPS---------------FEVSLT--PAKAFFYVD----DNDLTVDITARYL-YGKEV-TGTGYVVFGVIT-
TESEKKSFPASLQRVEI--
KDGKGVACLK--KEHITQ--TFPKIHD-----LVKQSIFVSVSVLTEGGGEMVEAEKRGIQIVTSPYSILFKRT---
PKYFKPGMPFDVSVYITN-PDNSPAI-----G-VEVEVTP--------------------------------DHAKGVTRAN---------------------
----------------GFAKIPLNTVA---SATELVITV----KTKDPGDPRQQ---------
-TGGGTMKALPYRT--STKNF------------LHV--------GVDSNELKI-------GDPIKIDLNLGPTTIPNHD---------------------
-LTYMFLSR-------GQLVKVGRFKRQGNA--
LVTLSVPVSKELLPSFRIVAYYH-V----GAAD-LVADSVWVDI-KVSCMG----S----LKVT--------
STRPKASYEPRRAFSLTITGDPG-A--KVGLVAVDKGV----------------YVLNS--KHRLTQT---------------------------------
----K-----IWDTI-EKHDTGCTA--GGGAD------NMGVF---------YDAGLVFETNT---
AKGTGIRTDPSCPVSSRR---------------------RRAVTISDVITSMASKYHGLAKE--CCVDGMRDNT--
MGYTCDRRAQYISDG---------DVCVQAFLVCCTEMASKKIESKQDALL-----------
LSRSEEDDDDDAYMRSEDIVSRSQFPESWMWEDTNLPECPAQNKHCESTSVIRNNFLKDSITTWQITAISLSKTHG
-ICV-A-DPFEMIVLK-----------------------------------EFFIDLKLPYSAVRNEQLEVKAILHNY-SED--PIIVRVEL-----------
---------MENGEVCSSAS----------------KKGKYRQEVN--------MDPMSTRVVPYVII-----P-MKL--G-----
LHSIEVKASVK---------------NS---GSNDGVKRDLRVVAEGVL--VKKETNVLLNPVKHGGEQT----SHI------------------
----PSGVPRN---QVPNS-DADTLISVT---AGEQTSV-LVE---
QAISGDSLGSL-IVQPVGCGEQNMIYMTLPVIATHYLDNTKKWEDIG-LDK--RNTAIKYINIGYQRQLAYR-K--
EDGSYAAW-------VSR---QSSTWLTAYVVKVFAMSS--------TL--I-SVQENVLCTAVKWLILNTQQP-
DGIFNEFAPVIHAEMTGNVRG--SDN-------------DASMTAFVLIAMQEASSVCEQSVNS-----LPGSMAKA--
VAYLEKRLPH-LT---NPYAVAMTSYALANAG-----KLNKETLLKFASPQL---------------------------DHWP----------------
------------VPGGYQYTLEATSYALL-ALVKVKA--
FEEAGPIVRWLNKQ--------KKVGGGYGSTQ-----STIMVFQAVA---EYWSHVKDL------KDFDLNINLEVAG-----
RASVTKWSINNKNQFHTRTDKV-NSIDK------DLTVKAS-GNGEATLSVVTLYYAL---PEEKDSDCESFDLSVT---------
--LTKMDKTSHEDAKESFMLTIEVLYK-----
NSERDATMSILDIGLLTGFIVDTDDLNQLS--

KGRERYIEKFEMDKVLSERGSLILYLDKVSHKLEDRISFKIHRVQEVGVL-QPAAVSVYEYYN-
QKRCVKFYHPQREGGTLSRL-CLG------DVCTCAEE--
-----------SCSMQKKGEP------------------------DVQRIDKAC-
GAGLDYVYKATVVDSKLTTHTDTYTVKIDLVIKPGTDEG---------------------
VEGKNRDFMGLAYCREALGLMQGKTYMIMGKSEDLH---RVEDKGLLQYKYVLGEQTWIEYWPSQQECTSR-------
--------------DYREVCLGIDEFINQITTF-GCPV---

>Cyprinus_C3H1 AB016210_Cyprinus carpio mRNA for complement C3-H1, complete cds

----

---MEVKLLFLTVVL--

----------------------------LSS---
------------------------------------PLLTLCNPLYVLSAPNLLRVGSSENVFVEAQ-----DYSGAAFDVKIIVKNHP-----------
KKDKEILSQSVSLTAANNFQILKDIKI---PDDQNYFSDDPLEKQYVYLQA-------------HFPSVILEKVVLLSF-QS-
GYIFVQTDKPIYTPAS-TVQYRIFSLTP-NLE--PLSQS-------------GITVEIM------------NPQGITVSS------EKIFPVKG--
----MKSGKY-------AIPEMASPGIWKVVTLFS---N----------TPQKTFT---------------------ADFEVKEYVLPT------------
---FEVKLK--PSKSFFYVH----DESLTVDIEAKYL-FGQKV-DGNAFVMFGVME--DEKKTSIPASLQKVQI----------------
---IKGEGTAELT--NQMITK--
TFPNINQ-----LVGRSIYVSVSLLTESGSEMVEAERRGIQIVTSPYTIHFRKT---PQFFKPGMPFDVSVYVTN-
PDQTPAV-----N-VEVEVNP------------------------------GGLRGQTRAN-----------------------------------
GIAKVTVNTPG---GSPTLAITA----KTKDPEIKDERQ---------
QAEKRMTAQAYIPKGGSNNY------------LHI--------GIDAAELQI-------SDPMKVNLNTGQSP-GVKDQD------------
-------YTYMILSK-------GQIVKVDRFKRKGQS---
LVTLPVTVTKDMVPSFRFVAYYH------VGLE-VVSDSVWVDV-KDTCMG----K----LQIK--------
VKNKMNTYGTGDEVKLQITGDPG-A--KVGLVVVDKAV----------------QVLN---KNRLTQT---------------------------
--------Q-----IWDVI-EKHDTGCTA---------------------------------------GGGKD------SMGVF---------

TDAGLMFESNG---AGGTNTRTMPECPKTSKR----------------------KRRAESLLQITSTLAGKYTGELKQ--
CCVDGMRDNK--LGYTCERRATYIVDG---------EECAKAFVDCCNKIKDRKNTETEEEE-----------
MLLARSDDDDDYYTESEEIVSRTQFPESWLWEEID-L-----CDKCPTPATEKVIYLKDSITTWEILAVSLSPTLG-ICV-A-
EPEEMVVFK--------------------------------------HLFIDLKMPYSAVRGEQLEIRAIIHNY-TPN--KQKVRVEF------------------
----METEDVCSFAS----------------KKGKYRTTVS--------VEKDSSISVSYVII-----P-MTL--G-----NHMIEVKASAY------
---------DA---IYTDGVRKPLKVVSEGVL-IPLHRKNVELNPVKTGEKPIVL------------------------------KSDIPVD---
RVPGT-PANTYISIT---GEEIAQ-TVE---QAISGDFMGRL-
IIQPNGCGEQIMIGMTLPVIATHYLDSTNQWETVG-MER--RNEAINHINAGYQRQLGYR-K--SDGSYAAW-------
THR---PSSTWLTAYVAKVFSMAN--------DF--A-AIEENVLCSALKWLVLHKQLP-DGSFKEESAVIHGEMVGDVRG-
-KDA-------------DASLTAFVVIAMQEAREICGGSVGS-----LHDSIKKA--VVFLEGRLPQ-LT---
NPYAVAMTSYAMANAG-----KLNKDILMKYSSQHE---------------------------AGRSWT-------------------------------
VPGQHHHSLEATAYAVL-ALVKAKD--
FDKAGEAVHWLNRQ--------KSHYGGSGTTQ-----ATIMVFQAVA---EYRTQVKDR------QNFNLDVELSVAG-----
RGKPVRYTIKRENAHLTRSDRVDINK--------EFNVTAR-GTGTATLSVLTLYYAR---PVEKKSDCTFFDLTVK------------
--MEKDNEANQGAIETYKITMDFYYK-----
SDKTDATMTILDIGIPTGFTVENRDLEELS---

RGKERYIQKFEMDKVLSERGSLILYLDKVLRKEPQRLAFRMHKMLNVGVL-
QPAAVTIYEYYSPNARCTKFFHPERADGAISRL-CKG------DLCQCAEE---
------------------------------------NCSYQKKN---------------------------HIGDEERFNRAC-
EAGMDYVYKVKVVGMDLQQDADIYDLEVEQVLKEGTDVD------------------------
VEGKRRPFLARPTCRNHLGLVKDKSYLIMGRSVDLP-------DLGGSLQYIFGEQTWIEYWPTRQESQTR-----------------
------EHRDRYIGISDLQNSLLKE-GCAT---

>Xenopus_C3 gi|758425|gb|AAB60608.1| complement component C3, partial [Xenopus laevis/gilli]

--AAGNNGFLGTATVKI----
PS-GDLSKDTKSKQYVYVSV-------------KSNVCALEKVVLLSY-HS-GYIFLQTDKTIYTPGS-TVLYRIYSMNY-KMQ--
PISKT--------------LIIEFL------------TPDGIIVNT----HTILQDSKSG------IISQSH-------KLPELLSLGVWTISAKYE---D-
---------TPQQNYT---------------------INFEVKEYVLPS---------------IEIILK--PDTNYFYSD----AESFGVDIQAQYL-
YGKHV-DGYAFVLFGIMK--DNVKKGIPESLTRIRI---
------------------------------DDGEGRAELK--RKDLVK--YFEKPED-----
MLQFSLYVTVSVVTETGSDMVEAEIEHISIVTTPYKILFTKT---SKYFKPGMPFDMMVYVTN-PDGSPAR-----R-
IRVVVEPG------------------------------N-VEGSTQGD-----------------------------------GTTRLTLNTPA-----------------
--------------------------------NINLLPITV----KTRDPTLPPGR----------QATATMTATAYRPSKAQGNY------------
LHI--------SIAGSDIKP-------GENIPVNFNIRNTD-AGVQKQIEH----------------FTYLIISR-------
GRILKVGRQQRQPGQP--FVTMSIPVTDTLIPSFRIVAYYI-V-SAGGGRD-
IVSDSLWVDV-VDDCMG----T----LSVTGD------KDRDNTIQTPGSPMRLKLRADHK-A--YVGLVAVDKGV----------
------YVLNS--KFKITQK-----------------------------------K-----VWDSV-EKSDIGCTP---------------------------------------
---GSGAN------SEGVF---------SDAGLALQTSF---GINTAQRSDAQCPAPAQR-------------------
RKRSSVALIEIKSGKASEYKDKA-K-KCCLDGMQENL--MGHTCDRRARYILDG---------
KECVDAFVDCCKYYEKKREAEKISKD------------DDTLGRSDEDDEYMLDSDIVSRTEFPESWFWKVEQ-
MVEKPDVNGISSKTLNV--FLKDSITTWEVLAVSLSENKG-LCV-G-QPYEIKVMK---------------------------------
DFFIDLKLPYSVVRNEQVEIRAILYNY-RND--RIKVRVEL-------------------IHNPEFCSLST----------------
AKKKYRQEVW--------IGALSSIAVPIVIV-----P-LTL--G-----QHDIEVKASVS---------------
AQSGFFGADGVRKKLKVVPEGMR-IAQDVKTVILEPEVKGKDGVQE--EKV---------------------EALNPKN---
VVPRT-DIDTTITLQ--GTPISQ-MVE---DAIDGNNMNHL-
IVVPAGCGEQNMISTTPSVIATRYLDASGQWERVG-VNR--RDQALKNMRQGYAQQMAFR-K--PDNSYAAW-----
--KDR---PASTWLTGYVAKVFGMAQ--------EF--I-DIEANVLCGSVKWLILEKQKP-
DGLFQENAPVIHQEMVGGITTGAAEG-------------DSSLTAFILIAMLECQRTCNEHVNN-----LQVSIDKA--
SSYLVGQYPG-LK---KPYSIAITSYALALAG-----KL--PNTNKLLSASI------------------------GNTHWE--------------------
--------EPGKRFISLETTSYALL-TLLKMKE--
FDLTGGIVRWLNEQ--------RYYGAVYGSTQ-----ATIVMFQALA---QYQTDIPSV------NELNLDVSLHLPE-----
RQQPLTYRINHENALLARSAETRLNQ--------DFVVKAK-GKGQGTMRVVTVYHAL---VTEKERKCLNFHLSVK-------
--VEEVELA-RPPDGAKATVSIEACARHL------
KNVDATMSIIDISMMTGFSPDTNSLDRLM---

KGVDKYISKYEVNKGANDKGTLILYLDKVSHIEEECVKFYAHQYFEVGFI-
QPASVTVYDYYTPDNRCTKFYHVEEGSALLGRI-CQG------DLCRCAEE--
--------------------------------NCFMQQQIEG---------------------KITAEMRINMGC-

APGVDFVYKATLTEVQPSDNFDNYVMTIKKVIKQGTDED---------------------
PEDKTRNFISHIKCRKALNMQLNRDYLIWGVTGDLW-------RQPDGYSYIIGKDTWMEWWPNERECQQR---------
------------ENQDLCDDFETVSDNLEIV-GCPN--

>Gallus_C3 gi|82136251|sp|Q90633|Q90633_CHICK Complement C3 precursor

----

---------------------------------------MGLLLLPLLLGVLLL--

------------------------------------HAVPTPAQMVTMVTPAVLRLDTDEKVVLEAP------GLSAPTEANILVQDFP---------
--QKRKVLFQVRKQLNPAEGMMAIATVKV------PVKLLPPVVGKHFVSVVA------------RVGQVTLEKVLLVSL-QS-
GHIFLQTDKPIYTPGS-TVLSRLFALSH-FMQ--PLLKT-------------VIVEVK-----------TPDNVIIKQ----
VPVSSPMRNG------IFSINH-------NLPEVVSLGTWTITAKFE---D----------SQDQVFS-------------------
TQFEVKEYVLPS--------------FEVTLD--PQEKFLYIDP---AEDFRVTITARYL-YGKNL-QGTAFVLFGVVV--
DDEKKTIPQSLQRVKV--
TDGDGQAVLP--MAMLRQ--PFANLQE-----LVGHSLYVTVTVLTESGSDMVEAQRSGIRIVTSPYTIHFTHT---
PKYFKPGMPFDPTVYVTN-PDNSPAA-----AGIPVKADN------------------------------FQGLVSTQRD----------------
------------------GTAKLVLNMPA--NKNSVPITV----RTDQKDLPPER-----
-----QASRQIVAEAYQSQGNSGNY-----------LHL--------AVGASQVQP-------GDNLPINFHLKSNR-DDVRKSVSY--
-------------FTYLILSK-------GHIVHVGRQPREGDQS--
LVTMSLPVTANLIPSFRIVAYYH-V----KPGE-IIADSVWVDV-KDTCMG----S----LVVRGA------
SEADNRVHEPRTPMRLHIEGDHK-A--HVGLVAVDKAV---------------YVLN---KNKLTQS----------------------------
-------K-----VWDTV-ENSDIGCTP--------------------------------------GSGRN------QVGVF---------
ADAGLSLTSNV---NINTEQRSEVQCAKPAKR-------------------KRRSVRLIKHKGTKMAEYSDKNLR-
KCCEDGIRKNL--MGYSCEKRATYVLDA---------KSCTEAFLSCCLYIKGIRDEEREL-------------
QYELARSEVDDAFLSDEDITSRSLFPESWLWQVEE-LTEPPNEQGISMKTLPI--YLKDSITTWEVLAVSISENKG-LCV-
A-DPYEITVMK---------------------------------EFFIDLRLPYSAVRNEQVEVRAILYNY-WTN--KIKVRVEL-------------
------MYNPALCSAST----------------TKTRYQQIFQ--------LEPQSSDAVPFVIV-----P-LEL--G-----
QHDVEVKAAVW---------------NS---FVSDGVKKKLRVVPEGMR-LEKTVKIVELDPKTLGNNGVQE--VKV-----------
----------KAANLSD---IVPNT-ESETKVSIQ--GNPVSI-LVE---
KATDGTKLKHL-IVTPSGCGEQNMIGMTPTVIAVHYLDSTMQWETFG-INR--RTEAIELIKKGYTQQLAYR-K--
EDGSFAAF-------TTR---PSSTWLTAYVAKVFAMAI--------NM--V-DIKPEVVCGAIKWLILEKQQP-
DGLFQEDAPVIHKEMVGGYHG--AEP-------------SVSLTAFVLSALQESQKICKNYVKS-----LDGSIAKA--
SDYLSRKYQS-LT---RPYTVALTSYALALTG-----KLNSEKV--LMKFSK-----------------------DGTHWA----------------

-----------ERNAHTYNIEGTSYALV-ALLQMEK---
AELTGPVVRWLAQQ--------NYFGGGYGSTQ-----ATILVFQALA---QYHVALPRH------VELNLDVSVLLPR-----
RANAITYRIENNNALVARSAETKLNE--------DFTVKAE-GTGKGTMTVVTVYKAK---VPEKENKCDNFDLRVS-------
--VEDVKAG-REVEGVIRSVKITICTRFL------
DTVDATMSILDISMLTAFSPDVQDLKSLS--

EGVERYISKFEIDHALSNRSNLIIYLDKVSHQVEECIAFRAHQHFQVGLI-
QPASVIVYSYYKIDDRCTRFYHPDKAGGQLRKI-CHG------EVC-CAEE---
------------------------------NCFIRVKKDN---------------------PITVNERIDLAC-
KPGVDYVYKVKVVATEETPSHDNYIMSILTVIKMGTDEN--------------------
PGGSNRTFVSHKQCRDALSLQKGQDYLVWGLASDLW-------VTGSRFSYLISKDTWLEAWPLEESCQDA------------
---------DLQPLCQDFTEFSDNLVLF-GCPT--

>Naja_C3 gi|399269|sp|Q01833.1|CO3_NAJNA RecName: Full=Complement C3; Contains: RecName: Full=Complement C3 beta chain; Contains: RecName: Full=Complement C3 alpha chain; Contains: RecName: Full=C3a anaphylatoxin; Contains: RecName: Full=Complement C3b alpha' chain; Contains: RecName: Full=Complement C3f fragment; Flags: Precursor

----

------------------------------------MEGMALYLVAALLIG---

-----------------------------------FPGSSHGALYTLITPAVLRTDTEEQILVEAH------GDSTPKSLDIFVHDFP-----------
RKQKTLFQSRVDMNQAGSMFVTPTIKV-----PAKELNKDSKQNQYVVVKV-------------TGPQVALEKVVLLSY-QS-
GFVFIQTDKGIYTPGS-PVRYRVFSVDH-NMH--RMDKT-------------VIVEFQ------------TPEGIVVSS--------
KPVNPS------GSIRPY-------NLPELVSFGTWKAVAKYE---H----------SPEESYT--------------------AYFDVREYVLPS-
-------------FEVRLQ--PSDKFLYIDG---NKNFHVSITARYL-YGKKV-EGVAFVVFGVKI--DDAKKSIPDSLTRIPI--------

--IDGDGEATLK--RDTLRS--
RFQDLNQ-----LVGHTLYVSVTVITESGSDMVVTEQGGIHIVTSPYQIYFTKT---PKYFKPGMPYELTVYVTN-
PDGSPAA-----H-VPVVSEA--------------------------------IHSEGTTLSD-------------------------------------
GTAKLILNTPL---NIQSLPITV----RTNHGDLPRER----------
QAIKSMTATAYQTQGGSENY------------LHV--------AITSTEIKP-------GDNLPVNFNVRGN--ANSLNQIKY----------
------FTYLILNK-------GKIFKVGRQPRRDGQN---
LVTMNLHITPDLIPSFRFVAYYQ-V----GNNE-IVADSVWVDV-KDTCMG----T----LVVKGA------
SSRDDRIQKPGAAMKIKLEGDPG-A--RVGLVAVDKAV----------------YVLND--KYKISQA-------------------------------
------K-----IWDTI-EKSDFGCTA--GSGQN------NLGVF---------EDAGLALTTST---
NLNTKQRSAAKCPQPANR-------------------RRRSSVLLLDSKASKAAQFQDQGLR-KCCEDGMHENP--
MGYTCEKRAKYIQEG---------DACKAAFLECCHYIKGIRDENQRES----------
ELFLARSDFEDELFGDDNIISRSDFPESWLWLTEE-LTGEPNNQGISSKTVPF--YLRDSITTWELLAVGLSPTKG-ICV-
A-EPYEITVMK-----------------------------------DFFIDLRLPYSVVKNEQVEIRAILYNY-ADE--DIYVRVEL----------------
----IYNPAFCSAST----------------EGQRYRQQFP--------IKALSSRAVPFVIV-----P-LEQ--G-----LHDVEVIASVR-------
--------GE---LASDGVRKKLKVVPEGER--KNIVTIIELDPSVKGVGGTQE--LTV-------------------IANKLDD---
KVPDT-EVETRISVL--GDPVAQ-IIE---NSIDGSKLNHL-
IITPSGCGEQNMITMTPSVIATYYLDATGQWENLG-VDR--RTEAIKQIMTGYAQQMVYK-K--ADHSYAAF-------
TNR---ASSSWLTAYVVKVLAMAS--------NM--VKDISHEIICGGVKWLILNRQQP-
DGVFKENAPVIHGEMLGGTKG--AEP-------------EASLTAFIVTALLESRSVCKEQINI-----LDSSINKA--
TDYLLKKYEK-LQ---RPYTTALTAYALAAAD-----RLNDDRV--LMAAST-------------------------GRNRWE----------------
-------------EYNARTHNIEGTSYALL-ALLKMKK--
FAEVGPVVRWLIDQ--------KYYGGTYGQTQ-----ATVMVFQALA---EYEIQMPTH------QDLNLDISIKLPE-----
REVPERYSINDRNAVQARTVETKLNE--------DFTVSAS-GDGKATMTILTVYNAQ---LREDANVCNKFHLDVS-------
--VENVELNLKQAKGGKAALRLKICTRYL------
GEVDSTMTIIDISMLTGFFPDAEDLKRLS---

NGVDRYISKFEIDNNMAQKGTVVIYLDKVSHSEDECLHFKIHKHFEVGFI-
QPGSVKVYSYYNLDEQCTKFYHPDKETGLLNKI-CHG------NICRCAEE---
---------------------------------TCSLLNQQK----------------------KIDLQLRIQKAC-
AQNVDYVYKTKLLRIEEKDGNDIYFMDVLEVIKGGTDRN----------------------
AQAKARQYVSQRKCQEALNLKLDNDYLIWGLSSDLW-------PMKDDISYLITKNTWIERWPNEDECQDE------------
---------EFQNLCDDFAQLSNTLTIF-GCPT--

>Naja_VenomFactor gi|2118405|pir||I51018 cobra venom factor precursor - monocled cobra

----

---MERMALYLVAALLIG---

------------------------------------FPGSSHGALYTLITPAVLRTDTEEQILVEAH------GDSTPKQLDIFVHDFP----------
RKQKTLFQTRVDMNPAGGMLVTPTIEI-----PAKEVSTDSRQNQYVVVQV-----------TGPQVRLEKVVLLSY-QS-
SFLFIQTDKGIYTPGS-PVLYRVFSMDH-NTS--KMNKT--------------VIVEFQ------------TPEGILVSS--------
NSVDLN-------FFWPY-------NLPDLVSLGTWRIVAKYE---H--------SPE-NYT-----------------------AYFDVRKYVLPS-
---------------FEVRLQ--PSEKFFYIDG---NENFHVSITARYL-YGEEV-EGVAFVLFGVKI--DDAKKSIPDSLTRIPI----------
---IDGDGKATLK--RDTFRS--
RFPNLNE-----LVGHTLYASVTVMTESGSDMVVTEQSGIHIVASPYQIHFTKT---PKYFKPGMPYELTVYVTN-
PDGSPAA-----H-VPVVSEA----------------------------------FHSMGTTLSD---------------------------------------
GTAKLILNIPL--NAQSLPITV----RTNHGDLPRER----------
QATKSMTAIAYQTQGGSGNY------------LHV---------AITSTEIKP--------GDNLPVNFNVKGN--ANSLKQIKY----------
------FTYLILNK-------GKIFKVGRQPRRDGQN--
LVTMNLHITPDLIPSFRFVAYYQ-V----GNNE-IVADSVWVDV-KDTCMG----T----LVVKG----------
DNLIQMPGAAMKIKLEGDPG-A--RVGLVAVDKAV----------------YVLND--KYKISQA------------------------------------
--K-----IWDTI-EKSDFGCTA---GSGQN------NLGVF---------EDAGLALTTST---
NLNTKQRSAAKCPQPANR----------------------RRRSSVLLLDSNASKAAEFQDQDLR-KCCEDVMHENP--
MGYTCEKRAKYIQEG---------DACKAAFLECCRYIKGVRDENQRES--------------
ELFLARDDNEDGFIADSDIISRSDFPKSWLWLTKD-LTEEPNSQGISSKTMSF--YLRDSITTWVVLAVSFTPTKG-ICV-
A-EPYEIRVMK-------------------------------------VFFIDLQMPYSVVKNEQVEIRAILHNY-VNE--DIYVRVEL---------------
------LYNPAFCSAST------------------KGQRYRQQFP--------IKALSSRAVPFVIV-----P-LEQ--G-----LHDVEIKASVQ----
-----------EA---LWSDGVRKKLKVVPEGVQ--KSIVTIVKLDPRAKGVGGTQL--EVI-----------------------KARKLDD---
RVPDT-EIETKIIQ--GDPVAQ-IIE---NSIDGSKLNHL-
IITPSGCGEQNMIRMAAPVIATYYLDTTEQWETLG-INR--RTEAVNQIVTGYAQQMVYK-K--ADHSYAAF-------
TNR---ASSSWLTAYVVKVFAMAA--------KM--VAGISHEIICGGVRWLILNRQQP-
DGAFKENAPVLSGTMQGGIQG--AEE--------------EVYLTAFILVALLESKTICNDYVNS-----LDSSIKKA--
TNYLLKKYEK-LQ---RPYTTALTAYALAAAD-----QLNDDRV--LMAAST--------------------------GRDHWE---------------
---------------EYNAHTHNIEGTSYALL-ALLKMKK---
FDQTGPIVRWLTDQ--------NFYGETYGQTQ-----ATVMAFQALA---EYEIQMPTH------KDLNLDITIELPD-----
REVPIRYRINYENALLARTVETKLNQ--------DITVTAS-GDGKATMTILTFYNAQ---LQEKANVCNKFHLNVS----------
---VENIHLN---AMGAKGALMLKICTRYL------
GEVDSTMTIIDISMLTGFLPDAEDLTRLS--

KGVDRYISRYEVDNNMAQKVAVIIYLNKVSHSEDECLHFKILKHFEVGFI-
QPGSVKVYSYYNLDEKCTKFYHPDKGTGLLNKI-CIG------NVCRCAGE--
--------------------------------TCSSLNHQE-----------------------RIDVPLQIEKAC-
ETNVDYVYKTKLLRIEEQDGNDIYVMDVLEVIKQGTDEN---------------------
PRAKTHQYISQRKCQEALNLKVNDDYLIWGSRSDLL-------PTKDKISYIITKNTWIERWPHEDECQEE-----------------
----EFQKLCDDFAQFSYTLTEF-GCPT---

>Human_C3 gi|119370332|sp|P01024.2|CO3_HUMAN RecName: Full=Complement C3; AltName: Full=C3 and PZP-like alpha-2-macroglobulin domain-containing protein 1; Contains: RecName: Full=Complement C3 beta chain; Contains: RecName: Full=Complement C3 alpha chain; Contains: RecName: Full=C3a anaphylatoxin; Contains: RecName: Full=Acylation stimulating protein; Short=ASP; AltName: Full=C3adesArg; Contains: RecName: Full=Complement C3b alpha' chain; Contains: RecName: Full=Complement C3c alpha' chain fragment 1; Contains: RecName: Full=Complement C3dg fragment; Contains: RecName: Full=Complement C3g fragment; Contains: RecName: Full=Complement C3d fragment; Contains: RecName: Full=Complement C3f fragment; Contains: RecName: Full=Complement C3c alpha' chain fragment 2; Flags: Precursor

----

-------------------------------------MGPTSGPSL--LLLL--

---------------------------LTH---
------------------------------------LPLALGSPMYSIITPNILRLESEETMVLEAH------DAQGDVPVTVTVHDFP-----------
GKKLVLSSEKTVLTPATNHMGNVTFTI----PANREFKSEKGRNKFVTVQA-------------TFGTQVVEKVVLVSL-QS-
GYLFIQTDKTIYTPGS-TVLYRIFTVNH-KLL--PVGRT--------------VMVNIE------------NPEGIPVKQ----
DSLSSQNQLG------VLPLSW-------DIPELVNMGQWKIRAYYE---N----------SPQQVFS--------------------
TEFEVKEYVLPS---------------FEVIVE--PTEKFYYIYN---EKGLEVTITARFL-YGKKV-EGTAFVIFGIQD--
GEQRISLPESLKRIPI---
EDGSGEVVLS--RKVLLDGVQNPRAED-----LVGKSLYVSATVILHSGSDMVQAERSGIPIVTSPYQIHFTKT---
PKYFKPGMPFDLMVFVTN-PDGSPAY-----R-VPVAVQGE-----------------------------DTVQSLTQGD----------------

--------------------GVAKLSINTHP--SQKPLSITV----RTKKQELSEAE-------
---QATRTMQALPYSTVGNSNNY------------LHL--------SVLRTELRP-------GETLNVNFLLRMDR-AHE-AKIRY-------
---------YTYLIMNK-------GRLLKAGRQVREPGQD---
LVVLPLSITTDFIPSFRLVAYYT-LIGASGQRE-VVADSVWVDV-KDSCVG----S----LVVKS-------
GQSEDRQPVPGQQMTLKIEGDHG-A--RVVLVAVDKGV----------------FVLNK--KNKLTQS---------------------------
---------K-----IWDVV-EKADIGCTP--GSGKD------YAGVF---------SDAGLTFTSSS-
--GQQTAQRAELQCPQPAAR-------------------RRR-SVQLTEKRMDKVGKYP-KELR-KCCEDGMRENP--
MRFSCQRRTRFISLG---------EACKKVFLDCCNYITELRR-QHARAS------------
HLGLARSNLDEDIIAEENIVSRSEFPESWLWNVED-L-KEPPKNGISTKLMNI--FLKDSITTWEILAVSMSDKKG-ICV-
A-DPFEVTVMQ----------------------------------DFFIDLRLPYSVVRNEQVEIRAVLYNYRQNQ--ELKVRVEL-----------
---------LHNPAFCSLAT----------------TKRRHQQTVT--------IPPKSSLSVPYVIV-----P-LKT--G-----LQEVEVKAAVY-
--------------HH---FISDGVRKSLKVVPEGIR-MNKTVAVRTLDPERLGREGVQK--EDI---------------------PPADLSD--
-QVPDT-ESETRILLQ---GTPVAQ-MTE---DAVDAERLKHL-
IVTPSGCGEQNMIGMTPTVIAVHYLDETEQWEKFG-LEK--RQGALELIKKGYTQQLAFR-Q--PSSAFAAF-------
VKR---APSTWLTAYVVKVFSLAV--------NL--I-AIDSQVLCGAVKWLILEKQKP-DGVFQEDAPVIHQEMIGGLRNN-
NEK-------------DMALTAFVLISLQEAKDICEEQVNS-----LPGSITKA--GDFLEANYMN-LQ---
RSYTVAIAGYALAQMG-----RLKGPLLNKFLTTAK------------------------DKNRWE----------------------------
DPGKQLYNVEATSYALL-ALLQLKD--
FDFVPPVVRWLNEQ--------RYYGGGYGSTQ-----ATFMVFQALA---QYQKDAPDH------QELNLDVSLQLPS-----
RSSKITHRIHWESASLLRSEETKENE--------GFTVTAE-GKGQGTLSVVTMYHAK----AKDQLTCNKFDLKVT----------
---IKPAPETEKRPQDAKNTMILEICTRYR------
GDQDATMSILDISMMTGFAPDTDDLKQLA---

NGVDRYISKYELDKAFSDRNTLIIYLDKVSHSEDDCLAFKVHQYFNVELI-
QPGAVKVYAYYNLEESCTRFYHPEKEDGKLNKL-CRD------ELCRCAEE---
--------------------------------NCFIQKSDD----------------------KVTLEERLDKAC-
EPGVDYVYKTRLVKVQLSNDFDEYIMAIEQTIKSGSDEV---------------------
QVGQQRTFISPIKCREALKLEEKKHYLMWGLSSDFW-------GEKPNLSYIIGKDTWVEHWPEEDECQDE-------------
--------ENQKQCQDLGAFTESMVVF-GCPN---

>Cavia_C3 gi|544053|sp|P12387.2|CO3_CAVPO RecName: Full=Complement C3; Contains: RecName: Full=Complement C3 beta chain; Contains: RecName: Full=Complement C3 alpha chain; Contains: RecName: Full=C3a anaphylatoxin; Contains: RecName: Full=Complement C3b alpha' chain; Contains: RecName: Full=Complement C3c alpha' chain fragment 1; Contains: RecName: Full=Complement C3dg fragment; Contains: RecName: Full=Complement C3g fragment; Contains:

RecName: Full=Complement C3d fragment; Contains: RecName: Full=Complement C3f fragment; Contains: RecName: Full=Complement C3c alpha' chain fragment 2; Flags: Precursor

----

-------------------------------------MGPAAGPSL--LLLL--

----------------------------LAS---
------------------------------------VSLALGDPMYSIITPNILRLENEETVVLEAH------EVQGDIPVTVTVHDFP-----------
AKKNVLSSEKTVLTSATGYLGTVTIKI----PASKEFKSDK-GRKLVVVQA-------------AFGGTQLEKVVLVSL-QS-
GYLFIQTDKTIYTPGS-TVLYRIFTVDS-DLL--PVGRT--------------IIVTIE------------TPDGIPIKR----DTLSSNNQHG--
----ILPLSW-------NIPELVNMGQWKIQAFYE---N----------SPKQVFS--------------------AEFEVKEYVLPS------------
---FEVLVE--PTEKFYYIDD---PKGLEVNIIARFL-YGKNV-DGTAFVIFGVQD--GDQRISLAQSLTRVVI------------------
---EDGSGEVVLS--
RQVLLDGVQPSRPEA-----LVGKSLYVSVTVILHSGSDMVEAERSGIPIVTSPYQIHFTKT---
PKYFKPAMPFEIMVLVTN-PDGSPAP-----H-VPVVTQG------------------------------SNVQSLTQAD------------------
------------------GVARLSINTPN--TRQPLSVTV----QTKKGGIPDAR-------
---QAINTMQALPYTTMYNSNNY------------LHL--------SMPRTELKP-------GETINVNFHLRSD--PNQEAKIRY------
----------YTYLIMNK-------GKLLKVGRQPREPGQA--
LVVLPMPITKELIPSFRLVAYYT-LIGASAQRE-VVADSVWADV-RDSCVG----T----LVVKGGSGKDGQDKRQQHL--
PRQQMTLRIEGNQG-A--RVGLVAVDKGV----------------FVLNK--KHKLTQS-----------------------------------K-----
IWDVV-EKADIGCTP---------------------------------------GSGKD------YAGVF---------TDAGLSFKSSK--
AGLQTAQREGLDCPKPAAR--------------------RRRSVQLMERRMDKAGKYKSKELR-RCCEDGMRENP--
MQFSCQRRARYVSLG---------EACVKAFLDCCTYMAQLRQQHRREQ-------------
NLGLARSDMDEDIIPEEDIISRSQFPESWLWTIEE-L-KEPERNGISTKTMNI--FLKDSITTWEILAVSLSDKKG-ICV-A-
DPFEVTVMQ---------------------------------DFFIDLRLPYSVVRNEQVEIRAVLYNYREAQ--SLKVRVEL---------------
-----LHNPAFCSLAT----------------AKKRHTQTVT--------IGPKSSVAVPYVLV-----P-LKI--G-----LQEVEVKAAVY-----
----------NY---FISDGVKKTLKVVPEGMR-VNKTVAIRTLNPEQLGQGGVQR--EEI--------------------PAADLSD---
QVPDT-DSETKILLQ---GTPVAQ-MAE---DAVDAERLKHL-
IITPSGCGEQNMIGMTPTVIAVHYLDQTEQWEKFG-LEK--RQEALNLINRGYTQQLAFK-Q--PNWAYAAF-------
KNR---ASSTWLTAYVVKVFSLAA--------NL--I-GIDSEVLCGAVKWLILEKQKP-DGVFQEDGPVIHQEMIGGVRTA-
QEA-------------DVSLTAFVLIALQEAKDICRAQVNN-----LEANINKA--GDYIESRYAD-VR---RPYTLAIAGYALALLE-
----RLNGATLQKFLNAAT-----------------------EKNRWE---------------------------EARQKLYSVEATSYALL-
ALLLLKD---FDAVPPVVRWLNEQ--------
RYYGRGYGSTQ-----ATFMVFQALA---QYQTDVPDH------KDLNMEVALQLPS-----
RSSPSKFRLVWEAGSLLRSEATKQNE--------GFKLTAK-GKGQGTLSVVAVYYAK----TKRKVVCKNFDLRVT---------

---LKPAPDTVKKPQEAKSTMILGICTRYL------
GDQDATMSILDISMMTGFIPDTDDLKLLA---

TGVDRYISKYEMNKDFS-KNTLIIYLDKVSHSEEECLSFKIHQFFNVGLI-
QPGSVKVYSYYNLDETCTQFYHPEKEDGMLNKL-CHK------DLCRCAEE--
-------------------------------------NCFIQLPE---------------------------KITLDERLEKAC-
EPGVDYVYKTKLLKMELSDDFDEYIMTIEQVIKSGSDEV---------------------
QAGKERRFISHIKCRDALHLKEGKHYLMWGLSSDLW-------GERPNMSYIIGKDTWVEAWPEAEECQDE-----------
----------ENQQQCQDLGTFTENMVVF-GCPN---

>Rattus_PreproC3 X52477_Rat pcRC201 mRNA for pre-pro-complement C3

---MGPTSGSQLLVLLLL---

---------------------------------LAS--
---SLLALGSPMYSIITPNVLRLESEETFILEAH------DAQGDVPVTVTVQDFL-----------
-KKQVLTSEKTVLTGATGHLNRVFIKI----PASKEFNADK-GHKYVTVVA-------------NFGATVVEKAVLVSF-QS-
GYLFIQTDKTIYTPGS-TVFYRIFTVDN-NLL--PVGKT--------------VVIVIE------------TPDGVPIKR----DILSSHNQYG-
-----ILPLSW-------NIPELVNMGQWKIRAFYE---H----------APKQTFS--------------------AEFEVKEYVLPS------------
---FEVLVE--PTEKFYYIHG---PKGLEVSITARFL-YGKNV-DGTAFVIFGVQD--EDKKISLALSLTRVLI--------------------
---EDGSGEAVLS--RKVLMDGVRPSSPEA-
----LVGKSLYVSVTVILHSGSDMVEAERSGIPIVTSPYQIHFTKT---PKFFKPAMPFDLMVFVTN-PDGSPAR-----R-
VPVVTQG------------------------------SDAQALTQDD----------------------------------GVAKLSVNTPN---------------
--NRQPLTITV----STKKEGIPDAR----------QATRTMQAQPYSTMHNSNNY-----------
-LHL--------SVSRVELKP-------GDNLNVNFHLRTD--AGQEAKIRY----------------YTYLVMNK-------
GKLLKAGRQVREPGQD---LVVLSLPITPEFIPSFRLVAYYT-LIGANGQRE-

VVADSVWVDV-KDSCVG----T----LVVKG-------DPRDNRQPAPGHQTTLRIEGNQG-A--RVGLVAVDKGV---------
-------FVLNK--KNKLTQS---------------------------------K-----IWDVV-EKADIGCTP---------------------------------
----GSGKN------YAGVF---------MDAGLTFKTNQ---GLQTDQREDPECAKPAAR-----------------
RRRSVQLMERRMDKAGQYTDKGLR-KCCEDGMRDIP--MPYSCQRRARLITQG---------
ESCLKAFMDCCNYITKLREQHRRDH------------VLGLARSDVDEDIIPEEDIISRSHFPESWLWTIEE-L-
KEPEKNGISTKVMNI--FLKDSITTWEILAVSLSDKKG-ICV-A-DPYEITVMQ-------------------------------
DFFIDLRLPYSVVRNEQVEIRAVLFNYREQE--KLKVRVEL-------------------LHNPAFCSMAT--------------
AKKRYYQTIE--------IPPKSSVAVPYVIV-----P-LKI--G-----LQEVEVKAAVF--------------NH---
FISDGVKKILKVVPEGMR-VNKTVAVRTLDPEHLNQGGVQR--EDV-----------------NAADLSD---QVPDT-
DSETRILLQ--GTPVAQ-MAE---DAVDGERLKHL-
IVTPSGCGEQNMIGMTPTVIAVHYLDQTEQWEKFG-LEK--RQEALELIKKGYTQQLAFK-Q--PISAYAAF-------
NNR---PPSTWLTAMWSRSFSLAA--------NL--I-AIDSQVLCGAVKWLILEKQKP-
DGVFQEDGPVIHQEMIGGFRNT-KEA------------DVSLTAFVLIALQEARDICEGQVNS-----LPGSINKA--
GEYLEASYLN-LQ---RPYTVAIAGYALALMN-----KLEEPYLTKFLNTAK----------------------DRNRWE---------------
-----------EPGQQLYNVEATSYALL-ALLLLKD---
FDSVPPVVRWLNDE--------RYYGGGYGSTQ-----ATFMVFQALA---QYRADVPDH------KDLNMDVSLHLPS-----
RSSPTVFRLLWESGSLLRSEETKQNE--------GFSLTAK-GKGQGTLSVVTVYHAK----VKGKTTCKKFDLRVT----------
---IKPAPETAKKPQDAKSSMILDICTRYL------
GDVDATMSILDISMMTGFIPDTNDLELLS--

SGVDRYISKYEMDKAFSNKNTLIIYLEKISHSEEDCLSFKVHQFFNVGLI-
QPGSVKVYSYYNLEESCTRFYHPEKDDGMLSKL-CHN------EMCRCAEE---
-------------------------------NCFMHQSQD---------------------QVSLNERLDKAC-
EPGVDYVYKTKLTTIELSDDFDEYIMTIEQVIKSGSDEV-------------------
QAGQERRFISHVKCRNALKLQKGKQYLMWGLSSDLW-------GEKPNTSYIIGKDTWVEHWPEAEERQDQ---------
----------KNQKQCEDLGAFTETMVVF-GCPN--

>Mus_C3 gi|341940525|sp|P01027.3|CO3_MOUSE RecName: Full=Complement C3; AltName: Full=HSE-MSF; Contains: RecName: Full=Complement C3 beta chain; Contains: RecName: Full=Complement C3 alpha chain; Contains: RecName: Full=C3a anaphylatoxin; Contains: RecName: Full=Acylation stimulating protein; Short=ASP; AltName: Full=C3adesArg; Contains: RecName: Full=Complement C3b alpha' chain; Contains: RecName: Full=Complement C3c alpha' chain fragment 1; Contains: RecName: Full=Complement C3dg fragment; Contains: RecName: Full=Complement C3g fragment; Contains: RecName: Full=Complement C3d fragment; Contains: RecName: Full=Complement C3f fragment; Contains: RecName: Full=Complement C3c alpha' chain fragment 2; Flags: Precursor

----

--MGPASGSQLLVLLLL---

-------------------------------LAS--
--SPLALGIPMYSIITPNVLRLESEETIVLEAH------DAQGDIPVTVTQDFL------------
KRQVLTSEKTVLTGASGHLRSVSIKI----PASKEFNSDKEGHKYVTVVA------------NFGETVVEKAVMVSF-QS-
GYLFIQTDKTIYTPGS-TVLYRIFTVDN-NLL--PVGKT-------------VVILIE------------TPDGIPVKR----DILSSNNQHG-
-----ILPLSW-------NIPELVNMGQWKIRAFYE---H----------APKQIFS---------------------AEFEVKEYVLPS-----------
---FEVRVE--PTETFYYIDD---PNGLEVSIIAKFL-YGKNV-DGTAFVIFGVQD--GDKKISLAHSLTRVVI------------------
---EDGVGDAVLT--
RKVLMEGVRPSNADA-----LVGKSLYVSVTVILHSGSDMVEAERSGIPIVTSPYQIHFTKT---
PKFFKPAMPFDLMVFVTN-PDGSPAS-----K-VLVVTQG-------------------------------SNAKALTQDD------------------
------------------GVAKLSINTPN---SRQPLTITV----RTKKDTLPESR---------
-QATKTMEAHPYSTMHNSNNY------------LHL--------SVSRMELKP-------GDNLNVNFHLRTD--PGHEAKIRY------
----------YTYLVMNK-------GKLLKAGRQVREPGQD---
LVVLSLPITPEFIPSFRLVAYYT-LIGASGQRE-VVADSVWVDV-KDSCIG----T----LVVKG-------DPRDNHL-
APGQQTTLRIEGNQG-A--RVGLVAVDKGV----------------FVLNK--KNKLTQS-------------------------------------K-----
IWDVV-EKADIGCTP---GSGKN------YAGVF---------MDAGLAFKTSQ---
GLQTEQRADLECTKPAAR---------------------RRRSVQLMERRMDKAGQYTDKGLR-KCCEDGMRDIP--
MRYSCQRRARLITQG---------ENCIKAFIDCCNHITKLREQHRRDH-------------
VLGLARSELEEDIIPEEDIISRSHFPQSWLWTIEE-L-KEPEKNGISTKVMNI--FLKDSITTWEILAVSLSDKKG-ICV-A-
DPYEIRVMQ----------------------------------DFFIDLRLPYSVVRNEQVEIRAVLFNYREQE--ELKVRVEL----------------
----LHNPAFCSMAT----------------AKNRYFQTIK--------IPPKSSVAVPYVIV-----P-LKI--G-----QQEVEVKAAVF-----
----------NH---FISDGVKKTLKVVPEGMR-INKTVAIHTLDPEKLGQGGVQK--VDV---------------------PAADLSD---
QVPDT-DSETRIILQ---GSPVVQ-MAE---DAVDGERLKHL-
IVTPAGCGEQNMIGMTPTVIAVHYLDQTEQWEKFG-IEK--RQEALELIKKGYTQQLAFK-Q--PSSAYAAF-------
NNR---PPSTWLTAYVVKVFSLAA--------NL--I-AIDSHVLCGAVKWLILEKQKP-
DGVFQEDGPVIHQEMIGGFRNA-KEA------------DVSLTAFVLIALQEARDICEGQVNS-----LPGSINKA--
GEYIEASYMN-LQ---RPYTVAIAGYALALMN-----KLEEPYLGKFLNTAK------------------------DRNRWE--------------
--------------EPDQQLYNVEATSYALL-ALLLLKD--
FDSVPPVVRWLNEQ--------RYYGGGYGSTQ-----ATFMVFQALA---QYQTDVPDH------KDLNMDVSFHLPS-----
RSSATTFRLLWENGNLLRSEETKQNE--------AFSLTAK-GKGRGTLSVVAVYHAK----LKSKVTCKKFDLRVS----------
---IRPAPETAKKPEEAKNTMFLEICTKYL------
GDVDATMSILDISMMTGFAPDTKDLELLA---

SGVDRYISKYEMNKAFSNKNTLIIYLEKISHTEEDCLTFKVHQYFNVGLI-
QPGSVKVYSYYNLEESCTRFYHPEKDDGMLSKL-CHS------EMCRCAEE---
---------------------------------NCFMQQSQE----------------------KINLNVRLDKAC-
EPGVDYVYKTELTNIELLDDFDEYTMTIQQVIKSGSDEV--------------------
QAGQQRKFISHIKCRNALKLQKGKKYLMWGLSSDLW-------GEKPNTSYIIGKDTWVEHWPEAEECQDQ-----------
----------KYQKQCEELGAFTESMVVY-GCPN---

>Cyprinus_C4_1 gi|9453863|dbj|BAB03284.1| complement C4-1 [Cyprinus carpio]

---MICPLC--

-----------------------FVV--
-----------------------------FVFLTVDAHRPTCLIVAPNVIHVGVEETVSVQLH------GATKETSMQLYFQEQI-----------
SRIIVSEKKSIILNKDNNYQAVVKLKV------DRSLYGSKKDEKYVQLAATSNDVFWN---TVGSARKAS---IYLST-KR-
GYIFIQTDKPIYNPGE-NVKYRIFTLDN-FML--PIKDE--------------IHVQII------------NCKGFIVYN-------HILISDQ----
--ILEKTI-------TIPEVEQEGKWKIIASYH---K----------YPESTTS--------------------LEFEVKEYVLPT---------------
FEVTIEA-PQPYHI-LKS----QSFPFNISARYT-YGKGV-NGIAYIRFGLID--EKGNKIYLPGTEKQ----------------ITV------
--EDGTAKTEVR--TEDLKT--ASENNTQGR--
-IEGYYLYIAVSVLEKASGNLEEAESSSVKIVTSPYVVDLSKT---KTYFTPGGVFSVLATATY-PDGKRVP-----N-
LKFTATVTIEGSPEHI----------------------LKNEGWGNDM-----------------------------------GDVAMPFQIPP---------
---QAKKLAVTV----FAEGRDQEVI-----------RSDKKMTAHAIKAE-GH-SY---------
---LSL--------EVEHQVLQP-------GEALKATLRDITPQ-SAAKPTF------------------IYFMVWSK-------
GQIVQIGRVPRTE---ITTVNVNVNVEMVPSFRLVAYYF---
TDSSPNEKIVADSVWVDV-KDICKG----K----VEID-----------PLPEIKPATKFEVTVRTDPK-A--KVALAAVDSAV------
----------YILN---KNKLGLQ-----------------------------------K-----MFEYM-NSYDLACSI----------------------------------
------GGGED------SSAVL---------QRAGLTFMCNC---DMETPTPLDHKCYKSKPE------------------

PRVRRTADYVKKFNNVVNSFKGSDRK--CCLDGVRPNK--MHTSCDERLKRVEAS-----K----SCRKAFKMCCEAAN--
--ERRRRETFEKRKTY-----LGRVADDTLDEIVINEDAIYLRKYFPQSWMWTVIQ-T-----DSTGI-IRHDA--
VAPDSITTWEIQAVGISPTKG-FCI-A-EPKPLRVFQ----------------------------------
DFFLSVNLPYSVKRNEQLQVKAVVYNY-KEE--SLKIIVKM--------------------DKKEGLCTAGGS------------------
DLKEEVV--------VPGNSAVAVYFTVV-----P-LVI--G-----NIPINVLAYAS----------------D---
TVNDRVEKELRVEGEGEI--ATIDREYNIDPKLRK--------LDI---------------------DIPVPAD---EIPGGVDSEISLSLK-----
---GGVMGE-SVN---NCLNLEGINKL-
INLPTGCAEQTMVKMSPAIHAMRYLDATNQWISLK-AER--RDEAQSMIQTGYNTVLTYK-K--VDGSYGAF-------
LRT---PSSIWLTAFIAKELTESR--------DI--I-SVKDSYIQESMSYLM-SKQKS-NGAWDDPNQLYDRGMKGGVGK--
AKD------------DVPLTAFILISMNHALKLYELGSDTE----LRVAMDRA--KSYLEGQLDA-LE---SPYSLAITAYALSLHD-
-QQSANAQRAHRKLKNIAN---------------------CDTTKCFWN--------------------
SSGGRGESAGKANAESVETTAYALL-SALSLRD--
KAYAKLTANWLTEQ--------RKYGGGFQSTQ-----DTVVALEALT---KYSMQNN--------DVDDLDLRVEMCL----
ENGKKQDLHLTKNNALTAEAVKV-RNVG-------KVSLNVQ-GTGRGTLAIVQTYRTM----
KKDESHCDLFHLSVTVKGELEYKIYNTNETFDDYYNY---------------------------------
EGDEDQKDEPMSSIEWFDLRSRRKRQISEEPKKESSVIYTVCVGMK------
KKNTIGMVMVEISLLSGLIPNAKDLEHNV---

--KGSERYIDHYEVHH---
--NKVFLYFAKI--TDTECLQFGAEQVVPMGLV-QPASAVIYDYYSPEKRCGIFYTAPHKSPMISKL-CNG------
DVCTCAEG--GCPRNKVTFS-------------------
--KDIKDKTRSRYACYSPGVDYAYVIKVLNSSTDHVFMYYTTLITDVLQTGLLDSG-------------------
IQKHATRQMILRLSCDE-LELKKEVHYLIMGQNDAIS----VLNNDGQHFRYVLNNDMWIEKMPEEKNCKAT---------
------------KNRAACHLLNEFIKQHPIT-RCDI---

>Triakis_C4 gi|34097968|dbj|BAC82347.1| complement C4 [Triakis scyllium]

--MFLLLLCL---

-----------------------FAA--
--------------------------------ASVTEQTLSFLIIGPNIIHTGVEESVSIQLH------DAEGTVNFQVYCWDLI-----------
KRKKCSETALFTLNPGNNYQENKTITVSQHKVKELSLWRRRQKYVYLVAESRQ---------ILQQRRMVP---IKLSN-
KM-GYIFIQTDQPIYTPNQ-IVMFRIFTLDH-YMR--PSNNQ-------------LRVSIY------------NSRNMQIMG-------
AVWMSEK------IKLLRT-------KIPDNEESGIWRIEAAFL---D---------SPMSKVS--------------------
AQFEVKEFVLPR---------------FDVKVQ--ADELFY-LVT---KDEFKFKIVAKHT-YGETV-EGRVYVRFGILH--
ESGNKSYIGGLEQE----------------LTM--
-MNGEVNSSLT--TQSLLE--KSNLISRNE---LVGWHLYMAVTTVESVSGEMEELELSNIKFVSSPYVIDLSKT---
RRYFTPMSSFATVATVTY-PDGTPAV-----N-VPVTIE----------------------------------GGQVRNTDEH---------------------
----------------GLVVFESEAPA---NASMLSMKV----MAGDGTLGKE-------
----FREEMVTAQVYQSP-SN-SY------------LHI--------RTPHKVPEA-------GQDIYIEFQAITEA-AGRSIDY--------------
-----YYYILINK-------GRVIASDRIHKAD--
PTKLRLQVTLDMVPTVRLLAYYY--TQAGERKE-IVANSVWIDV-KDECEG----Q----IEIK----------
EENKSYGPGGVYDLQFSTNDA-A--NVSFVAVDAAM-----------------YILNN--KNKLTPR----------------------------------
----K-----VFEQM-NSYDLGCFY--GGGVN------SVGVF---------KDAGLSFISDV----
DVASIRHEYSCKNEVRR---------------------QRRALNLQRQYAGKLNEYTDSRLR-KCYTDGLTKIL--
MPHSCEERTSRVKEL----------DCRLVFQKCCDYGV----ELRKNQSLKVD--------
SIARAAVEVDEEFFDETDVHVRSVFPHSWLWKTVE-V-----LSAGD-HKIRN--YMPDSITTWEIQAVGMFANKG-
FCV-A-EPKMIKVFR------------------------------------PFFISVKLPYSVKRNEQLDLTVILYNY-LHE--DLEVAVYM---------
-----------RKAEGLRTAAGS-----------------REKSRNVL--------VKANSAASVQFTTV-----P-LVV--G-----
NMPINVIAFSR---------------TH---TYSDAVLKHLKVLSEGVI--VTEESSIPINPKAKAS-------YEI---------------------
YEEEPSN---LVPFT-DNYLYIRAI---GRITGE-AVE---
NSLTAAGIEKL-IRVPTGCAEQTAMHMAPTVFAVEYLDQSDQWISLK-AER--KDEAIFHIETGYNKILGYK-K--
DDGSYGAW-------KTY---PSSTWLTAFIVKILSMAR--------SK--I-VINDSFIRESVAYLI-RGQKS-
SGAFEDPHPVIDRNMQGGVDG--TEK-------------DVSLTAFVTIALKHSLTAFPQNNIAEISR-VERSIGKA--
VEFLHKMFPY-VI---LPHTFAITAYAFAFVG--ADTDIVFEADRKLRAIAS--------------------YDTEQDIRYWK-------------
--------VDETLTPSGKQASAITVETTSYALL-QTLSRNG--
IDYAMPIVNWLTEQ--------QNYGGGFRSTQ-----DTVIALEALS---QYHIAFLKRE-----EEIDMQIHFNVLG-----
SPTENNIHLRRRNALTQKELKF-P-LGS------RIRIELS-GQGNGTLNFRKSYHLM----
EEPSSTCKTVHLSVTVKGQLEFRKPYDYAEADKQLNDS----------------------------------
KTLPANAPTGPLDWYDLRSRRKRDV---PDPTNRQTIYYEVCYWRD-
RNHEEIQFPSGMAIVDILLLSGLKPELVHLDQLK---

NGADQYIDHYEVKD-----GRVLLYLNTD-RMQKECIEFSAKQIVRMGLV-
QPATATLYDFYNPSVRCTLTYSAPKQNVMITKL-CDG------EVCMCAEG---

---------------------------------------ACPHINHVLS-----------------------
NEVNGSARIDFACYSPIVDYAYTVRVLNISSAGSFDNYQVSIADRLKMSNKDDD---------------------
VKIGEVRHFLSQRSCK--FQLKEGDEYLVMGQDGTTS-------DTSRKMQYLLDVKSWVEAIPPEDICELR----------------
------KYRSSCRNLRNPMTARVDH-GCP---

>Gallus_C4 gi|7512215|pir||T28153 complement C4 - chicken

--MGLL---

-------------------------LAA--
--VGPV---------------------------TGTVTAWAEGDR----------
GAGPCTLPVPFALTPHNNFNQLLQIEVTPVQAERCG----ALWGRGLLLEAHSSHL------PPPSTRSLS---VALGG-PR-
GHLIVQTDKPLYAPRQ-TVRFRVFSMDP-DLQ--PNPEP-------------VLVTIT------------NPLGARVRE------
VQRVPLDT------VLSDQL-------VLPDIALPGTWHIRAQLA---A----------SPNTNGS-----------------------
TAFEVRKYVLPG---------------FEVRIR--PERGFI-VLSDPDPAPLRIHLHVQFP-DGAPV-
WGRAQLRVGLRDARGQGGR-FLRGLEQQ-----------------SQV--
--------------------------------TEGHASLEVS--PVGVAK--AAGVALAD----LQGALLRLAVGVVESAGGELVERELS-
VPLVLSQWVLQLQKS---ARFFVPGAPYTLLVSTCA---NPPHAKGECLG-VTGVGTGAPAP-----------------------------
PLIELQADNS---------------------------------------GDIAVPINVPK---
GATRMELSV--MA-SQ----------------VTL--
--TEVALPVTADMAPYLHVVAFFL-
-----SGGN-VVAATWGGAV-RGGCDE----Q----VGVQLKVP------PRGTTLRPQDPLKVTVTTGTP-V--
TVAIGAIDTTV------------------LNLEP--RHRLNTA--K-----VEAAL-GSSDLGCSP---------
----------------------------------GGGPD------AVGIF---------GAAGLVLGLQG----GSPTVPAAPHCPPAPTR----------------
-----QRRSLELLKLLEEKAGPWRNNTVMWRCCRDGATALP--IRATCQQRGQRVTTA-----G----
GCRDAFLQCCEVAQ----NLRRKGQRGGL--------ARVMEAQLAEQLLDDDEDVPTRSFFPESWLWRRIH-V-------
AGT-ARLSV--LLPDSITTWEIQAVAIVPGHG-LCV-A-EPQRVTVTQ-------------------------------------
DVRVALWLPPSIRPLEQMQLQPLIHSR-LPR--SINVTVTL---------------------SAVEGVCAALDG--------------------
VPQMLE--------LPPGRAVAAPLTLV-----A-LHP--G-----DIPITITARGP-----------------W---
GLGDRVTRVLHVEPEGEL--HLEESTYILDADGGCEDWGTLGKLGT-WGRTLWCGVPTDKRSRSLKLPGDVPAE---
IVPDG-DFSMSIRVS---RVPGWALQGALGIG-----------DSL-
LRSPRGCGEQSLMSMAPTAAALRFLDESEGWGQLP-PGH--RQRGLRTLQQGFERVQSFR-K--SDGSYGAW-------
LHR---DSSTWLTALVLRVLALSR--------PY--L-PVAASGPAASLRWVL-GQQRP-
DGAFLEHRAVVHREMQGGVADPGPEA------------TVSLTAFVVVALHGARALLPPDSPELPL--LDKSLSRA--

STFLRGRVEQ-L----GTYGTAITSYALALVD--TAPPGPHPAVERLRGMAR-------------------SAHGGRATFWP--------
---------------------SGGPAATVEATGYALL-ALLQSRD---
IAGAARAARWLRQQ--------SNYGGGFHSTQ-----DTLVALEALAQMWLHWGRG---------NTMGLNLGLSWPG---
GARGRAGGTQVMLKPGLEPLEQELQVPLGS------PVTVQVE-GHGEGTLTVLRQFRLL----
SPPNATCQALHLEVAITGPILYHADEDYEDYEDYEEAEPKEGEEPTEGAV-------------------
PVEGAGPADDPAPLSPVSLWDARKRQRRSTHNPAHEVAFLVCFRRS-----
PGVALTGMAVVEITLLSGFSPHRADLDKLR---

---DVVDHWISHYELEG-
----NQLVLYLDEV-PPERQCLSFGATQDAAVGHM-QPAMAAIYDYYEPGQRCTVFYNAPQRSSTIATL-CSP------
KICECAQG--GCPQAQRRTQ------------------
----EVTADDRHDFACYSPRVDYALVVRVLSQSEIGAFVAFETEIKEVLLEGQDTA-------------------
VAPGERRRLLVRKSCP--LRLQLHNIYLVMGGSGRTR-------DPEGRPQFLLGPHSWLEEVPSPGRCKAT--------------
-------RLRGYCAQLQEFRTRLSQL-GCQL---

>Xenopus_C4 D78003_Xenopus laevis mRNA for fourth component of complement, complete cds

----

----------------------------MGPPPLLYAL--------VWAAVC---

--------------------------------MVA---
---KAQQPNFLVVAPRILHVGVKEVIGIQFEWPQGSLPPAGDIKVELSLRNQL-
----------SMIGCSGEEKLFLNKRNDYTILTNLMLTSQRLITCKLNERRSGRYVQLVVKSD--VL------GKQPKVVS---
IPVAY-QR-GYLFIQTDKSIYTPKE-TVHIRSFTLDH-VLR--PTEEQ--------------VTLSVF------------NAQGFQVRK------
VEKISKDS------VVADNL-------QIPDISTPGVWRISVHYT---D----------APETNFT---------------------
AEFEVKPYVLPN---------------FEVKII--PEVPYF-LMT---KDSFTFRVEARYV-YGEHV-AGVGYMRVGITA--
QNGKRYMLRGLEKQ----------------TTL---
--IDGDTTVTIR--MADIKE--KIQQDMGNL----LGTSLYFAASVVEKASGVLEEKEFTSVKFVSSPYKLDLSKT---

KRYFIPGTPAQIVVEVSH-IDGSPAA-----G-VSVSLSKSNA-----------------------------QLFEYKTDNN---------------------
----------------GVVAFHVNTAS---DEKKCDIRV----KANFDSG--------------
SETESITLLPYTSK-AS-SY------------LFL--------NVPNQVLDP-------GSSFKVTLKAIISN-KQNVEK--------------------
IYYMVLNK-------GQLLSLDSIRRTE--VNEMLITVKPSMIPSFRVIAYYY-
-----LGSE-IISNSVWVDV-ADVCEG----K----LELH----------ASKKILAPGEALKLDVRTEGT-A--TVSLSAVDTAV-------
---------YILNS--KNKLTPQ-------------------------------------K-----MFKAM-NAYDLGCSP---------------------------------
--------GGGKD------FINVF---------TDAGLAFVSSA-----GYTQINELGCRVHQ----------------------
RKKRAIDFQALTQQKAYSYTTTELQ-RCCQHGMMLPPGKMSRVCTKRAARVPDP----------TCRKAFLDCCEYAE---
-NLRKQLTLEKRKTQGF---GRTQNVDGSDEDFADESDIQIRSFFPETWLWRTVK-------VYNGL-FSEAV--
YMPDSITTWEIQAIGMSREKG-FCI-A-EPLKVKVFK-----------------------------------
DFHIYLRVPYSVKRFEQMELRPILYNY-NNK--DLEVKVYM---------------------EQAEDICSPGSG---------------
DSKPLIKEVT--------VGANSALPVPFVVV-----P-IGK--S-----NPVVSVVALGR----------------S---
FVSDGVKKAMKIVKEGAS--VFEEKSYIIDPADITRRS-----IDF----------------------DEEFPSN---MIPDG-DFRSSIKVT--
---MDSSMN-TIN---NSLGADGISKL-
IRVPYGCAEQTMISTSPGVYALRYLDHTEKWNLLS-PDR--KDEGLENMRQGYLRILQFK-K--ADGSYGAW-------
LHR---TSSTWLTAFVVKVLSLCR--------NY--I-DVNVEDIRLSAQYLA-TMQKD-TGAFQEKVSVIHQDMLGGSTT--
IDA-------------EVSITAYVTVSLYHSLDSLSEDNVA-----VKSKISKA--VDYLRGKLDT-IK---HPYSLALTTYALTLTS--
KDSVLKDKAYNKLMSHAQ--------------------GDPNKKELYFG------------------------------PKGTALAVETTSYVLL-
ITLLRGN--IKDAEKMYTWLSEQ--------
QNYGGGFKSTQ-----DTVMALEALS---EYWIRTFKQ------DDNTLEIEVNSLE-----
KQHSQKFKLRKEDNLQEELRSMGT-----------KFNIKVS-GKGKGILTVIKMYNIL----
QMQNTSCTELGLDVTVSDACEEAGMNEDDDY--
DYDEEMADEPREPIVWHDLRRRSRREATFTKEKEVKLLYEVCLWKK----
SSNVRLSGMAIVDITLLSGIEPVIDDLKKLA--

--ESSERYISHYEYQP-----
GRLLLYFDKV-PDTWDCVAFEAVQTVKVSLL-QPASAVIYDFYEPNIKCTVFYGAPSKPNFVSTL-CSG------
DVCQCAEG--LCPKKISSFK---------------------
---STETERQTFACYSPRVVFGYRVMVENIAEQDAFIVYTARILEVLQRNSDED---------------------
IKPEDTRLFYQPMSCK--MRLAKETEYLIMGQDGVTK-------NEHGHIRYILEKNFWVEELPGEQKCAAT--------------
-------RYRNFCTDARNFMDKYKEN-GCMV---

>Mus_C4_B gi|341940526|sp|P01029.3|CO4B_MOUSE RecName: Full=Complement C4-B; Contains: RecName: Full=Complement C4 beta chain; Contains: RecName: Full=Complement C4 alpha chain; Contains: RecName: Full=C4a anaphylatoxin; Contains: RecName: Full=Complement C4 gamma chain; Flags: Precursor

----

-------------------------------------MRLL--------WGLAWV---

------------------------------FSF---
--------------------------------------CASSLQKPRLLLFSPSVVNLGTPLSVGVQLL----DAPPGQEVKGSVFLRNPK-------
-----GGSCSPKKDFKLSSGDDFV-LLSLEVPLEDVRSCGLFDLRRAPHIQLVAQSP--WLRNTAFKATETQGVN---
LLFSS-RR-GHIFVQTDQPIYNPGQ-RVRYRVFALDQ-KMR--PSTDF-------------LTITVE------------NSHGLRVLK----
--KEIFTSTS------IFQDAF-------TIPDISEPGTWKISARFS---D----------GLESNRS---------------------
THFEVKKYVLPN---------------FEVKIT--PWKPYI-LMVPSNSDEIQLDIQARYI-YGKPV-QGVAYTRFALMD--
EQGKRTFLRGLETQ----------------AKL--
-VEGRTHISIS--KDQFQA--ALDKINIGVRD-LEGLRLYAATAVIESPGGEMEEAELTSWRFVSSAFSLDLSRT---
KRHLVPGAHFLLQALVQE-MSGSEAS-----N-VPVKVSATLVSGSDSQV---------------------LDIQQSTNGI------------
------------------------GQVSISFPIPP--TVTELRLLV----SAGSLY-------------
--PAIARLTVQAPPSR-GT-GF------------LSI-------EPLDPRSPSV-------GDTFILNLQPVGIP-APTFSH------------------
-YYYMIISR-------GQIMAMGREPRKT---
VTSVSVLVDHQLAPSFYFVAYFY------HQGH-PVANSLLINIQSRDCEG----K----LQLKV---------
DGAKEYRNADMMKLRIQTDSK-A--LVALGAVDMAL----------------YAVGGRSHKPLDMS----------------------------
--------K-----VFEVI-NSYNVGCGP---GGGDD------ALQVF---------
QDAGLAFSDGD----RLTQTREDLSCPKEKKS-------------------RQKRNVNFQKAVSEKLGQYSSPDAK-
RCCQDGMTKLP--MKRTCEQRAARVPQQ----------ACREPFLSCCKFAE----DLRRNQTRSQAHL------
ARNNHNMLQEEDLIDEDDILVRTSFPENWLWRVEP-V-----DSS---KLLTV--WLPDSMTTWEIHGVSLSKSKG-
LCV-A-KPTRVRVFR-----------------------------------KFHLHLRLPISIRRFEQFELRPVLYNY-LND--DVAVSVHV---------
-----------TPVEGLCLAGGG------------------MMAQQVT--------VPAGSARPVAFSVV-----P-TAA--A-----
NVPLKVVARGV-------------------FDLGDAVSKILQIEKEGAI--HREELVYNLDPLNNLGRT-----LEI---------------------
PGSSDPN---IVPDG-DFSSLVRVT---ASEPLETMGSE---
GALSPGGVASL-LRLPQGCAEQTMIYLAPTLTASNYLDRTEQWSKLS-PET--KDHAVDLIQKGYMRIQQFR-K--
NDGSFGAW-------LHR---DSSTWLTAFVLKILSLAQ--------EQ--V-GNSPEKLQETASWLL-AQQLG-
DGSFHDPCPVIHRAMQGGLVG--SDE-------------TVALTAFVVIALHHGLDVFQDDDAKQLKNRVEASITKA--
NSFLGQKASAGLL---GAHAAAITAYALTLTK--ASEDLRNVAHNSLMAMAE---------------------ETGEHLYWG------
LVLGSQDKVVLRPTAPRSPTEPVPQAPALWIETTAYALLHLLLREGK--
------------------GKMADKAASWLTHQ--------GSFHGAFRSTQ-----DTVVTLDALS---AYWIASHTT------
EEKALNVTLSSMG----RNGLKTHGLHLNNHQVKGLEEELKFSLGS------TISVKVE-GNSKGTLKILRTYNVL----
DMKNTTCQDLQIEVKVTGAVEYAWDANEDYEDYYD-MPAAD-----------------------------
DPSVPLQPVTPLQLFEGRRSRRRREAPKVVEEQESRVQYTVCIWRN-----
GKLGLSGMAIADITLLSGFHALRADLEKLT---

--SLSDRYVSHFETDG----
-PHVLLYFDSV-PTTRECVGFGASQEVVVGLV-QPSSAVLYDYYSPDHKCSVFYAAPTKSQLLATL-CSG------
DVCQCAEG---KCPRLLRSLE-------------------
RRVEDKDGYRMRFACYYPRVEYGFTVKVLREDGRAAFRLFESKITQVLHFRKDTM-------------------
ASIGQTRNFLSRASCR--LRLEPNKEYLIMGMDGETS-------DNKGDPQYLLDSNTWIEEMPSEQMCKST-------------
--------RHRAACFQLKDFLMEFSSR-GCQV--

>Mus_MHC_slp M21576_Mouse MHC sex-limited protein (Slp) mRNA, complete cds

----

----------------------------------MRLL--------WGLAWV--

-----------------------------FSF---
-------------------------------------CASSLQKPRLLLFSPSVVNLGTPLSVGVQLL----DAPPGQEVKGSVFLRNPK-------
-----GGSCSPKKDFKLSSGDDFV-LLSLEVPLEDVRSCGLFDLRRAPHIQLVAQSP--WLRNTAFKATETQGVN---
LLFSS-RR-GHIFVQTDQPIYNPGQ-RVRYRVFALDQ-KMR--PSTDF--------------LTITVE------------NSHGLSVLK----
--KEIFTSTP------ILQDTF-------IIPDISEPGTWKISARFS---D----------GLESNRS--------------------
THFEVKRYVLPN---------------FEVKIT--PWKPYI-LMVPSNSDEIQLDIQARYI-YGKPV-QGVAYTRFALMD--
EQGKRTFLQGLETQ----------------AKL---
-VEGRTHISIS--KDQFQA--ALDKINIGVRD-LEGLRLYAATAVIESPGGEMEEAELTSWRFVSSAFSLDLSRT---
KRHLVPGAHFLLQALVRE-ISGSEAS-----N-VIVKVSATLVSGSDSQV---------------------LNVQQSTNRI---------------
---------------------GQVSISFPIPP---TVTELRLLV----SAGSLY---------------
PTIARLTVQSPPSR-GT-GF------------LSI-------EPLDPRSPRV-------GDTFILNLQAVGIP-APTFSH-------------------
YYYMIISR-------GQIMAMSREARRT--
VTSVSVLVDHQLAPSFYFMAYFY------HQGH-PVANSLLINIQPRDCEG----K----LQLKV---------
VGAKEYHNGDMMKLQIQTDSK-A--LVALGAVDTAL----------------YAVGGWSHKPLDMS---------------------------
---------K-----VFEVI-NSYNLGCGP--------------------------------------GGGDD------ALQVF---------

QDAGLAFSDGD----RLTQTREDLSCPKEKKS------------------RQKRNVNFLKALSEKLGQYSSPDAK-
RCCQDGMTKLP--MKRTCEQRAARVPQQ----------ACREPFLSCCKFAE----DLRRNQTRSQAPL------
ARKVRDMV---NLIEEDDILVRTSFPENWLWRVEP-V-----DSS---KLLTV--RLPDSMTTWEIHGVSLSKSKG-LCV-A-
KPTRVRVFR---------------------------------KFHLHLRLPISVRRFEQLELRPVLYNY-LND--DKNVSVHV---------------
----TPVEGLCMAGGG------------------MMAQWVI--------VPAGSALPVAFSVV-----P-TAS--T-----NVPLKLVAKGT-
-----------------LDSSDSVSKILQIEKEGAI--HREEIVYNLDPLNNLGQM-----LEI--------------------PGSSDPN---
IVPEG-DFSTFVKVT--ASEPLETLGSE---GALSPGGVASL-
LRLPRSCAEQTMIYLAPTLTASNYLDRTKQWSKLS-PET--KDHAVHLIQKGHVRIQQFR-K--KDGSFGAW-------
LHR---DSSTWLTAFVLKILSLAQ--------EQ--V-GNSPEKLQETASWLL-AQQLG-DGSFHDPCPVIHRAMQGGLVG--
SNE------------TVALTAFVVIALHHGLNVFREGHAKQLKNRVEASITKA--NSFLGQKASAGLL---
GAHAAAITAYALTLTK--ASEDLRNVAHNSLMAMAE--------------------ETGENLYWG------
LVLGSQDKVVLRPAAPRSPTEPVPQAPALWIETTAYALLHLLLREGK---
-----------------GKMADKAASWLTHQ--------GNFHGAFRSTQ-----DTVVTLDALS---AYWIASHTT------
EEKALNVTLSSMG----RNGLKTHVLHLNNHQVKGLEEELKFSLGS------TISVKVE-GNSKGTLKILRTYNVL----
DMKNTTCQDLQIEVKVTDAVEYAWSAYEDYEDDYN-MPATD---------------------------
DPSVPLQPVTPLQLFEGRWSRRRREAPKVAEERESRVHYTVCIWHN-----
GKLGLSGMAIADITLLSGFHALRADLEKLT--

--SLSDRYVSHFETDG----
-PHVLLYFDSV-PTTRECVGFGASQEVVVGLV-QPASAVLYDYYSPDHKCSVFYAAPTKSQLLATL-CSG------
DVCQCAEG--KCPRLLRSLE------------------
RRVEDKDGYRMRFACYYHQVEYGFTVKVLREDGRAAFRLFESKITQVLHFRTDTM-------------------
ASIGQTRNFLSRASCR--LRLEPNKEYLIMGMDGETS-------DNKGDPQYLLDSNTWIEEMPSEQMCKST------------
--------RHRAACFQLKDFLMEFSSR-GCQV--

>Human_C4A K02403_Human complement component C4A mRNA, complete cds

----

---------------------------TRSAPRAASWLEDPREVRSVCLSAT--

---------------------------------FFT--
---LSLQKPRLLLFSPSVVHLGVPLSVGVQLQ----
DVPRGQVVKGSVFLRNPS----------RNNVPCSPKVDFTLSSERDFA-
LLSLQVPLKDAKSCGLHQLLRGPEVQLVAHSP--WLKDSLSRTTNIQGIN---LLFSS-RR-GHLFLQTDQPIYNPGQ-
RVRYRVFALDQ-KMR--PSTDT-------------ITVMVE-----------NSHGLRVRK------KEVYMPSS------IFQDDF-------
VIPDISEPGTWKISARFS---D----------GLESNSS------------------TQFEVKKYVLPN-------------FEVKIT--
PGKPYI-LTVPGHLDEMQLDIQARYI-YGKPV-QGVAYVRFGLLD--EDGKKTFFRGLESQ--------------TKL---------
---VNGQSHISLS--KAEFQD--ALEKLNMGITD-
LQGLRLYVAAAIIESPGGEMEEAELTSWYFVSSPFSLDLSKT---KRHLVPGAPFLLQALVRE-MSGSPAS-----G-
IPVKVSATVSSPGSVPEA-----------------QDIQQNTDGS-------------------------------GQVSIPIIPQ----------
---------------------------------TISELQLSV----SAGSPH-------------PAIARLTVAAPPSG-GP-GF-----------LSI--
-----ERPDSRPPRV-------GDTLNLNLRAVGS--GATFSH----------------YYYMILSR-------GQIVFMNREPKRT------
--LTSVSVFVDHHLAPSFYFVAFYY------HGDH-PVANSLRVDVQAGACEG--
--K----LELSV---------DGAKQYRNGESVKLHLETDSL-A--LVALGALDTAL--------------YAAGSKSHKPLNMG-------
-------------------------K-----VFEAM-NSYDLGCGP-----------------------------------GGGDS------ALQVF-------
--QAAGLAFSDGD----QWTLSRKRLSCPKEKTT----------------RKKRNVNFQKAINEKLGQYASPTAK-
RCCQDGVTRLP--MMRSCEQRAARVQQP----------DCREPFLSCCQFAE----SLRKKSRDKGQAG------
LQRALEILQEEDLIDEDDIPVRSFFPENWLWRVET-V--------DRFQILTL--WLPDSLTTWEIHGLSLSKTKG-LCV-A-
TPVQLRVFR-----------------------------EFHLHLRLPMSVRRFEQLELRPVLYNY-LDK--NLTVSVHV-------------
----SPVEGLCLAGGG---------------GLAQQVL--------VPAGSARPVAFSVV-----P-TAA--A-----AVSLKVVARGS---
------------FEFPVGDAVSKVLQIEKEGAI--HREELVYELNPLDHRGRT-----LEI-----------------PGNSDPN---
MIPDG-DFNSYVRVT---ASDPLDTLGSE---GALSPGGVASL-
LRLPRGCGEQTMIYLAPTLAASRYLDKTEQWSTLP-PET--KDHAVDLIQKGYMRIQQFR-K--ADGSYAAW-------
LSR---DSSTWLTAFVLKVLSLAQ--------EQ--V-GGSPEKLQETSNWLL-SQQQA-
DGSFQDPCPVLDRSMQGGLVG--NDE------------TVALTAFVTIALHHGLAVFQDEGAEPLKQRVEASISKA--
NSFLGEKASAGLL---GAHAAAITAYALSLTK--APVDLLGVAHNNLMAMAQ------------------ETGDNLYWG------
SVTGSQSNAVSPTPAPRNPSDPMPQAPALWIETTAYALLHLLLHEGK--
-----------------AEMADQASAWLTRQ--------GSFQGGFRSTQ-----DTVIALDALS---AYWIASHTT------
EERGLNVTLSSTG----RNGFKSHALQLNNRQIRGLEEELQFSLGS------KINVKVG-GNSKGTLKVLRTYNVL----
DMKNTTCQDLQIEVTVKGHVEYTMEANEDYE--YDELPAKD-------------------------
DPDAPLQPVTPLQLFEGRRNRRRREAPKVVEEQESRVHYTVCIWRN-----
GKVGLSGMAIADVTLLSGFHALRADLEKLT---

--SLSDRYVSHFETEG---
--PHVLLYFDSV-PTSRECVGFEAVQEVPVGLV-QPASATLYDYYNPERRCSVFYGAPSKSRLLATL-CSA------
EVCQCAEG--KCPRQRRALE----------------

-RGLQDEDGYRMKFACYYPRVEYGFQVKVLREDSRAAFRLFETKITQVLHFTKDVK------------------
AAANQMRNFLVRASCR--LRLEPGKEYLIMGLDGATY-------DLEGHPQYLLDSNSWIEEMPSERLCRST-------------
--------RQRAACAQLNDFLQEYGTQ-GCQV--

>Cyprinus_C5_2 gi|24785187|dbj|BAC23058.1| complement component C5-2 [Cyprinus carpio]

-----------------------------------MARL--------FFLCIL---

---------------------------HLF--
------------------------------------FCLTVQESVYLITAPKLLRLDALENVVVQLF------GYDQEITVHLYLKNTL----------
APVYKEYASQSLKLNAVNNYQASATLRI--------MPADFPKEDKFVYLQAIS----------SAFSAHEK---IPVTT-MN-
GFLFIQTDKPLYTPEQ-EVQVRVYSLNE-ELR--KSRRT--------------VTLTFV------------DPNGIKVEM----
IDVADIIGAK------PLLPPF-------KIPLKPTYGIWKIVATYA---D----------TFETTAT---------------------
AEFEVKEYVLPS---------------ILLHIE--PEANYVSEKN---FESFKLKISAKYA-RGTPVSPADIFLTFGYS---
TPEETVMIPETFAR----------------YML---
-ENGKEEVHLD--IKSALL--SMPDGPQSLSAMKENSFLRVRVLLQESTGGISQEAVLSNVKFVETPFTLNLFST---
PPFIKPGLPYAMRVLVKD-PLGKPVQ-----G-VSVKTRATITTADNRLDTLKFLGHL------------NTLTSTSQRD---------
---------------------------GIAYFTCNIPA--NVKQAEFTF----ETEDPSWS---
----------QAPLKLKAESYESV-NH-RY------------LYI-------DLPVSSAFEV-------GQYINVNVYFDFHD-NLSLKT--------
-----------FSFQILSR-------GKVVQFDTVKWSGGKN--
QNLNIKITPDMVPSARLVVYYV--LYGEEKAE-LVADSTWIDV-KAKCVN----N----LNVDIS------
VRNSEYVFKPKDKLELRVSTNSREE-SLVAFSAVDTAL----------------YNLKS--NDKDPLK-------------------------------
-----K-----VLQHV-ERSDLGCGR---GGGKN------NADVF---------ERVGLMFITNA--
-----NAKTSSAGGACSDL------------------ARPKRSADLAQMFEQKAKKYGKFY---SCCTFGIASSP--
TLETCQQRSERLSYP-----AK--TMCQQAFQECCGFAM----KQRQENK------------
DKFILSRSAVNFLLDAMPSQTRSYFPESWLWEEHT-------SKSGS-VSITR--NLPDSLTTWELKAVGVF-SEG-ICV---
SEDKIQVSQ--------------------------------DISVDVPLPYSMVRGEQIELRGSVYNQ-RLS--NTRFSVTL---------------
----TATEGVCVFRGAKQGK-----------HGKPHENKGL--------IEGLSVSSVTFYIM-----A-LEA--G-----THTLTFTLKTT-
-------------------WGTESVVKKLKVVPEGIR--KEIQSGGRIDPNGVFGTSMRT--LEF--------------------KNVIPPN---
IVPKS-TVNRILTVY--GDVLGE-LLS---IINNPEGLKQL-
TNLPRGSAEVELMGLLPIYYVYHYLEKTEKWGVLGKEAAASEKELRRKMQAGITSIMSFKMK--NEHAFSMWS-----
NKDK---SASTWVTALMVKTLADMQ--------EY--V-SLESQVLTNTINWLIKNCQND-
DGSFSEKSQSIPLKLMGAGADV-TEK------------TISLTSFVLIGIKNAMKVPDVNIQA-----FKTAIDRA--
AQYITSNSKK-IE---SLYATAIASYALTLAD--IHSISAVSLYEKIKKQAQ-----------------VDGNPATVRFWQ------------

-----ESKADRDLLKPSDVTAKTVETTVYVLL-NTLTRGE---
-STYAKPILNWLTQD--------QRYGRGVHSTQ-----DSILTLEALT---KYSIIA---------RQATLDMVVNIEY----
KTKGDISRIRLTQQMPVHKPIDVIKND--------DIIIKTAMSSGVTFASLRTVYYEM-----TENNENCHFDLSII-----------
---ISERDPNSYDPMLSSQRIVACAKYK--
PPENSNEKESMTVMEINLPTGVTPVQEDLDMYQ---

NGLESRISDYKITG-----DQVILQINSIPSEDLYCVGFRIQEEFEVGMT-RASVFRVYEFYAPENTCIKLYRKQSRRLLQL-
--CEG------DQCHCMAA--ECCNFKSTIN----
------------------PTINAERLKNDMCKDNMKYAFKVLIESSEAAGDFGTYKAKVKAVLKKERTDD--------------------
LNTDSEIDLVKKATCNSTNFEVGKQYLIMTAESMKI-----KVNRGYTYKFPLDSHAWVKWWPLDYDCRDA-----------
----------ACKKYASVLEGFEFDFLSS-GCKN---

>Mus_C5 gi|116608|sp|P06684.2|CO5_MOUSE RecName: Full=Complement C5; AltName: Full=Hemolytic complement; Contains: RecName: Full=Complement C5 beta chain; Contains: RecName: Full=Complement C5 alpha chain; Contains: RecName: Full=C5a anaphylatoxin; Contains: RecName: Full=Complement C5 alpha' chain; Flags: Precursor

----

-----------------------------------MGLW--------GILCLL--

---------------------------IFL--
----------------------------------DKTWGQEQTYVISAPKILRVGSSENVVIQVH------GYTEAFDATLSLKSYP-----------
DKKVTFSSGYVNLSPENKFQNAALLTL---QPNQVPREESPVSHVYLEVVS------------KHFSKSKK---IPITY-NN-
GILFIHTDKPVYTPDQ-SVKIRVYSLGD-DLK--PAKRE--------------TVLTFI------------DPEGSEVDI------
VEENDYTGI-----ISFPDF-------KIPSNPKYGVWTIKANYK---K----------DFTTTGT--------------------
AYFEIKEYVLPR---------------FSVSIE--LERTFIGYKN---FKNFEITVKARYF-
YNKVVPDAEVYAFFGLREDIKDEEKQMMHKATQA---------------AKL---

--VDGVAQISFD--SETAVK--ELSYNSLED---
LNNKYLYIAVTVTESSGGFSEEAEIPGVKYVLSPYTLNLVAT---PLFVKPGIPFSIKAQVKD-SLEQAVG-----G-
VPVTLMAQTVDVNQETSDLE------------------TKRSITHDTD------------------------------------GVAVFVLNLPS-------
--NVTVLKFEI----RTDDPELPEEN----------QASKEYEAVAYSSL-SQ-SY----------
--IYI------AWTENYKPMLV-------GEYLNIMVTPKSPY-IDKITH-------------------YNYLILSK-------
GKIVQYGTREKLFSST---YQNINIPVTQNMVPSARLLVYYI--VTGEQTAE-
LVADAVWINI-EEKCGN----Q----LQVHLS--------PDEYVYSPGQTVSLDMVTEAD-S--WVALSAVDRAV-------------
---YKVQG--NAKRAMQ------------------------------------R-----VFQALDEKSDLGCGA-----------------------------------
-----GGGHD------NADVF---------HLAGLTFLTNA-------NADDSHYRDDSCKE-----------------
ILRSKRNLHLLRQKIEEQAAKYKHSVPKKCCYDGARVN---FYETCEERVARVTIG-----PL----CIRAFNECCTIAN----
KIRKESPH-----------KPVQLGRIHIKTLLPVMKADIRSYFPESWLWEIHR-V--------PKRKQLQV--
TLPDSLTTWEIQGIGIS-DNG-ICV-A-DTLKAKVFK----------------------------------
EVFLEMNIPYSVVRGEQIQLKGTVYNY-MTS--GTKFCVKM--------------------SAVEGICTSGSSAASL-----------
HTSRPSRCVF------QRIEGSSSHLVTFTLL-----P-LEI--G-----LHSINFSLETS--------------------
FGKDILVKTLRVVPEGVK--RESYAGVILDPKGIRGIVNRR--KEF---------------------PYRIPLD---LVPKT-KVERILSVK-
--GLLVGE-FLS---TVLSKEGINIL-
THLPKGSAEAELMSIAPVFYVFHYLEAGNHWNIFYPDTLSKRQSLEKKIKQGVVSVMSYR-N--ADYSYSMWKGA---
-------SASTWLTAFALRVLGQVA--------KY--V-KQDENSICNSLLWLVEKCQLE-
NGSFKENSQYLPIKLQGTLPAEAQEK-------------TLYLTAFSVIGIRKAVDICPTMK-------IHTALDKA--
DSFLLENTLP-SK---STFTLAIVAYALSLGD--RTHPRFRLIVSALRKEAF------------------VKGDPPIYRYWR--------------
----DTLKRPDSSVPSSGTAGMVETTAYALL-ASLKLKD--
-MNYANPIIKWLSEE--------QRYGGGFYSTQ-----DTINAIEGLT---EYSLLL---------KQIHLDMDINVAY----
KHEGDFHKYKVTEKHFLGRPVEVSLND--------DLVVSTGYSSGLATVYVKTVVHKI-----SVSEEFCSFYLKID-----------
---TQDIEASSHFRLSDSGFKRIIACASYK-
PSKEESTSGSSHAVMDISLPTGIGANEEDLRALV--

EGVDQLLTDYQIKD-----GHVILQLNSIPSRDFLCVRFRIFELFQVGFL-
NPATFTVYEYHRPDKQCTMIYSISDTRLQKV---CEG------AACTCVEA--
-------------------------------DCAQLQAEVD----------------------
LAISADSRKEKACKPETAYAYKVRITSATEENVFVKYTATLLVTYKTGEAAD-----------------------
ENSEVTFIKKMSCTNANLVKGKQYLIMGKEVLQI-----KHNFSFKYIYPLDSSTWIEYWPTDTTCP----------------------
-SCQAFVENLNNFAEDLFLN-SCE--

>Human_C5 gi|166900096|sp|P01031.4|CO5_HUMAN RecName: Full=Complement C5; AltName: Full=C3 and PZP-like alpha-2-macroglobulin domain-containing protein 4; Contains: RecName: Full=Complement C5 beta chain; Contains: RecName: Full=Complement C5 alpha chain; Contains:

RecName: Full=C5a anaphylatoxin; Contains: RecName: Full=Complement C5 alpha' chain; Flags: Precursor

----

-----------------------------------MGLL--------GILCFL--

-------------------------IFL--
--------------------------------GKTWGQEQTYVISAPKIFRVGASENIVIQVY------GYTEAFDATISIKSYP-----------
DKKFSYSSGHVHLSSENKFQNSAILTI---QPKQLPGGQNPVSYVYLEVVS------------KHFSKSKR---MPITY-DN-
GFLFIHTDKPVYTPDQ-SVKVRVYSLND-DLK--PAKRE--------------TVLTFI------------DPEGSEVDM------
VEEIDHIGI-----ISFPDF-------KIPSNPRYGMWTIKAKYK---E----------DFSTTGT--------------------
AYFEVKEYVLPH---------------FSVSIE--PEYNFIGYKN---FKNFEITIKARYF-
YNKVVTEADVYITFGIREDLKDDQKEMMQTAMQN----------------TML--
---------------------------------------INGIAQVTFD--SETAVK--ELSYYSLED---
LNNKYLYIAVTVIESTGGFSEEAEIPGIKYVLSPYKLNLVAT---PLFLKPGIPYPIKVQVKD-SLDQLVG-----G-
VPVTLNAQTIDVNQETSDLD------------------PSKSVTRVDD-----------------------------------GVASFVLNLPS--------
--GVTVLEFNV----KTDAPDLPEEN----------QAREGYRAIAYSSL-SQ-SY---------
---LYI------DWTDNHKALLV-------GEHLNIIVTPKSPY-IDKITH-------------------YNYLILSK-------
GKIIHFGTREKFSDAS---YQSINIPVTQNMVPSSRLLVYYI--VTGEQTAE-
LVSDSVWLNI-EEKCGN----Q----LQVHLS--------PDADAYSPGQTVSLNMATGMD-S--WVALAAVDSAV----------
------YGVQR--GAKKPLE------------------------------------R-----VFQFL-EKSDLGCGA------------------------------------
----GGGLN------NANVF---------HLAGLTFLTNA-------NADDSQENDEPCKE-----------------ILRPRRT---
LQKKIEEIAAKYKHSVVKKCCYDGACVN---NDETCEQRAARISLG-----PR----CIKAFTECCVVAS----QLRANISH-----
------KDMQLGRLHMKTLLPVSKPEIRSYFPESWLWEVHL-V--------PRRKQLQF--ALPDSLTTWEIQGVGIS-NTG-
ICV-A-DTVKAKVFK---------------------------------DVFLEMNIPYSVVRGEQIQLKGTVYNY-RTS--GMQFCVKM----
----------------SAVEGICTSESPVIDH-----------QGTKSSKCVR------QKVEGSSSHLVTFTVL-----P-LEI--G-----
LHNINFSLETW--------------------FGKEILVKTLRVVPEGVK--RESYSGVTLDPRGIYGTISRR--KEF---------------------
PYRIPLD---LVPKT-EIKRILSVK---GLLVGE-ILS---
AVLSQEGINIL-THLPKGSAEAELMSVVPVFYVFHYLETGNHWNIFHSDPLIEKQKLKKKLKEGMLSIMSYR-N--
ADYSYSVWKGG----------SASTWLTAFALRVLGQVN--------KY--V-EQNQNSICNSLLWLVENYQLD-
NGSFKENSQYQPIKLQGTLPVEAREN-------------SLYLTAFTVIGIRKAFDICPLVK-------IDTALIKA--DNFLLENTLP-
AQ---STFTLAISAYALSLGD--KTHPQFRSIVSALKREAL------------------VKGNPPIYRFWK------------------
DNLQHKDSSVPNTGTARMVETTAYALL-TSLNLKD---
INYVNPVIKWLSEE--------QRYGGGFYSTQ-----DTINAIEGLT---EYSLLV---------KQLRLSMDIDVSY----
KHKGALHNYKMTDKNFLGRPVEVLLND--------DLIVSTGFGSGLATVHVTTVVHKT-----STSEEVCSFYLKID---------

--TQDIEASHYRGYGNSDYKRIVACASYK-
PSREESSSGSSHAVMDISLPTGISANEEDLKALV---

EGVDQLFTDYQIKD-----GHVILQLNSIPSSDFLCVRFRIFELFEVGFL-SPATFTVYEYHRPDKQCTMFYSTSNIKIQKV-
--CEG------AACKCVEA---DCGQMQEELD---
-------------------LTISAETRKQTACKPEIAYAYKVSITSITVENVFVKYKATLLDIYKTGEAVA----------------------
EKDSEITFIKKVTCTNAELVKGRQYLIMGKEALQI-----KYNFSFRYIYPLDSLTWIEYWPRDTTCS-----------------------
SCQAFLANLDEFAEDIFLN-GC---

>Lenthenteron_A2M D13567_Lethenteron camtschaticum mRNA for alpha-2-macroglobulin, complete cds

----

-------------------------------MGMAGHAFL--------LLLCAL---

--------------------------------AAV---
---VSADEQSSGHYLVFVPSELHALSSERLCVSLL----GVTG--
EVTFRATLHYKDSRDTSHVAQNHTVVGEVDAGVLLQPSGGDLHHCFSFTV---------PDVQGTTYANLVVRA----A-
GE---GLNFTKTHA---VVVRK-VK-DVVFVQTDKPVYKPGQ-SVKFRVVTLDE-NFA--TVLKTY-----------A--LIYIE-----
-------DPQRNRIAQ----WRNASGRA-G------IVQLEL-------DMPSEPPLGTYNVMVVEQSG-G----------DSVASHT--
---------------------FTVEEYVLPT---------------FEVSIQT-PS--YL-NYL---DKSVTLKVCGRYT-YGKPV-
HGAVNASVCIQG-QPRFWW----REECIIPVCNEFFM-------KVG---
-------------------------------KDGCAEWQVD--NAK-----PSNASC------HTTHVLKVVAVLEEEGTGMKMKQ-
KAEKNFETDITRISFVDM---PSWYRHGLPIVGKVKVER-PDGSPVP-----H-KLVSLIVKQGNAAP------------------------
-PGSQHHTGAD-------------------------------------GTFTFTIDTGD-----FN---
RSDTIFLEA----TDPEFNSTAHPSVT-------YQQGYSTISAFFSP-SD-SF------------LQL------DRVAHTLECGS-------
SVPLRLLLVLKENR-SSAGDGHAGGVVHPV----------INVLVMSR-------GNILHTESFTMDN---------------------------

------------------VYLGKAHSHHFQLKVKHSFAPTARLLAWMV-F-----NNE-TVADTLALPV-HKCFPN----K----VSVG-
---------FEHEQELPGAMSTLSIRASPG-S--LCALRVVDKSV----------------LLLKP--EAQLSAE-----------------------------
--------S-----IFQRL-QVQDLTYYDYGIERWDCSPEHYRRRKRSYGVWMD---------------DFQNS------VHRLF---------
KGMGLVVLTDT-------TVKRSVDCHQLMHH---------------------RHRIAYSMETVASPGLRFSDRGN--------------
ISPV--GQGGAMFKTVKETVREYFPETWIWDLYP-V-----
SESGL-EQVAV--KVPDSITEWKASAFC-SSPAG-FGL-S-EVSSLRVFT----------------------------------
PFFVEPVLPYSVVRGETFPLAISVHNY-LHS--CLKIEVTL--------------------L-DSEHFAVA-----------------
AGGPRGAASC--------VCPDRKGLFSFLIE-----P-LAL--G-----TVNVSVRAAAV-HSDEPCGNEVVVVPEE---
GAVDTVVRSVIVEPEGIP--KELAYSSLLCPKSPAS--ER---FVF---------------------NLPLPAN---VIVGS-A-RAYATIA---
---GDIMGS-ALQ---N------LDKL-
LTLPTGCGEQNMVKFAPNIYIQEYLQNSGQLT----DAV--RDKALNFLRVGYQRQLTYK-R--DDHSYSAFG-----
KSDD---DGNTWLTAFVLKSFVRAS--------KH--I-AVSEDHITGPFSWLV-EHQNASTGCFISVGRLFNNAMKGG-----
VSD-------------DVSLTAYVTAALLESNIS--GPVVE---R-ALGCLRPL---------VLE-SS---NAHLLALASYAFSLSA---
DGATAQALRDALQSRAV----------------TK---G--GLTHWQEANKK------
DDKDEEDEEGGFNFRRSYGGTTSAAVETTAYALL-SRLAVPGQLA-----ASA---
---------------TSSNIGVVQWLSKQ--------RNAYGGFSSTQ-----DTVVGLQALS---AFAALI-
DGDGGGGGSGGIKAVVLDSAH----TLLREVSID--STNALILHQVQL-PPPVPAPMVS-SCTVEAT-
GQGCALFQVSLKYNEP---PKSS---KPKFSLSVETNPAN---
---------CS-KAARKGFQ-INVEVS---YHGERGESNMALVEVKMISGYSAVKSSLKELQ-------------------------------------
----

---------------------TFYDFVKKVEVDA-----SRVVIYLDKV-DKKPIAIKLSVTQDIAVDNL-
QPATVRVYDYYATEDAATSMYSA-PCTH-SQ---

>Gallus_Ovostatin gi|1171932|sp|P20740.3|OVOS_CHICK RecName: Full=Ovostatin; AltName: Full=Ovomacroglobulin; Flags: Precursor

----

-------------------------MHCFLGREILSFFCLTVRKMWLKFILAIL--

---------------------------------------LLH---
--AAAGKEPEPQYVLMVPAVLQSDSPSQVCLQFF----NLNQ--
TISVRVVLEYDT--------INTTIFEKNTTTSNG-------LQCLNFMI-------PPVTSVSLAFISFTAKG----T------
TFDLKERRS---VMIWN-ME-SFVFVQTDKPIYKPGQ-SVMFRVVALDF-NFK--PVQEM------------YPLIAVQ--------
----DPQNNRIFQ----WQNVTSEI-N------IVQIEF-------PLTEEPILGNYKIIVTKK---S----------GERTSHS---------------
--------FLVEEYVLPK---------------FDVTVTA-PG--SL-TVM---DSELTVKICAVYT-YGQPV-
EGKVQLSVCRDFDSYGRCKKSPVCQSFTK--------------DLD---
--------------------------TDGCLSHILS---SKVFE--LNRI--------GYKRNLDVKAIVTEKEQVCNLTA-
TQSISITQVMSSLQFENV---DHHYRRGIPYFGQIKLVD-KDNSPIS-----N-KVIQLFVNNK----------------------------
NTHNFTTDIN------------------------------------GIAPFSIDTSKI--
FDPELSLKA----LYKTSDQCHSEGWIEPS----YPDASLSVQRLYSW-TS-SF------------VRI------EPLWKDMSCGQ-----
--KRMITVYYILNTEG-YEHINIVN-----------------FYYVGMAK-------GKIVLTGEIKVNIQAD-----------------------------
-----------------QNGTFMIPLVVNEKMAPALRLLVYML-H----PAKE-LVADSVRFSI-EKCFKN----K----VQLQ----------
FSEKQMLTTSNVSLVIEAAAN-S--FCAVRAVDKSM----------------LLLKS--ETELSAE-------------------------------------
T-----IYNLH-PIQDLQGYIFNGLNLEDDPQDPCVSSDDIFHKGLYYRPLTS--------GLGPD------VYQFL---------
RDMGMKFFTNS-------KIRQPTVCTRETVR---------------------------PPSYFLNAGFTASTHH--------------------------
--VKLSAEVAREERGKRHILETIREFFPETWIWDIIL-I-----NSTGK-ASVSY-
-TIPDTITEWKASAFCVEELAG-FGM-S-VPATLTAFQ----------------------------------
PFFVDLTLPYSIIHGEDFLVRANVFNY-LNH--CIKINVLL---------------------LESLDYQA-----------------
KLISPEDDGC--------VCAKIRKSYVWNIF-----P-KGT--G-----DVLFSITAETN--DDEACEEEALRNIRI---
DYRDTQIRALLVEPEGIR--REETQNFLICMKDDVIS------QDV--------------------AIDLPTN---VVEGS-P-RPSFSVV-
---GDIMGT-AIQ---N------VHQL-
LQMPFGNGEQNMVLFAPNIYVLDYLDKTRQLS----EDV--KSKTIGYLVSGYQKQLSYK-H--PDGSYSTFG-----IRDK-
--EGNTWLTAFVYKSFAEAS--------RF--I-YIDDNVQAQTLIWLA-TKQKT-DGCFQSTGILVNNAMKGG-----VEN---
----------ELSLSAYITIALLEAGHSMSHTVIR---N-AFYCLETA---SE-----KN-IT---DIYTQALVAYAFCLAG---
KAEICESFLRELQKSAK----------------EV---D--GSKYWEQN-QR------SAPEKSHL-----LDHV----QSTDVEITSYVLL-
ALLYK----PNRS--QED---LTKASAIVQWIIRQ--------
QNSYGGFASMQ-----DTVVALQALA---AYGAAT-YNS-----VTQNV-IKINSKN----TFEKVFTVN--NENRLLLQQTPL-
PQVPG------KYSLTVN-GTGCVLIQTALRYNIH---LPEG---AFGFSLSVQTSNAS---
------------------------------------CPRDQP-GKFD-IVLISS---YTGKRSSSNMVIIDVKMLSGFVPVKSSLDQLI------------
----

---DDHTVMQVEYKK-----NHVLLYLGNILQKRRKEVTFSVEQDFVVTHP-
KPAPVQIYDYYETEEYAVAEYMS-LCRG-VVEE-MG--

>Rattus_Alpha1Inhibitor gi|112893|sp|P14046.1|A1I3_RAT RecName: Full=Alpha-1-inhibitor 3; AltName: Full=Alpha-1-inhibitor 3 variant II; Short=Alpha-1-inhibitor III; Flags: Precursor

----
------------------------MKKDREAQLCLFSAL--------LAFLPF---
-----------------------------------ASL---LNGNSKYMVLVPSQLYTETPEKICLHLY----HLNE--TVTVTASLISQR--------
GTRKLFDELVVDKDL-------FHCVSFTI--------PRLPSSEEEESLDINI----E-GA---KHKFSERRV---VLVKN-KE-
SVVFVQTDKPMYKPGQ-SVKFRVVSMDK-NLH--PLNELF-----------P--LAYIE------------DPKMNRIMQ----
WQDVKTEN-G------LKQLSF-------SLSAEPIQGPYKIVILKQ---S----------GVKEEHS-----------------------
FTVMEFVLPR---------------FGVDVKV-PN--AI-SVY---DEIINVTACATYT-YGKPV-
PGHVKISLCHGNPTFSSETKSGCKEEDS----------------RLD--NNGCSTQEVN--ITE-FQ--LKENYL------KMHQAFHVNATVTEEGTGSEFSG-
SGRIEVERTRNKFLFLKA---DSHFRHGIPFFVKVRLVD-IKGDPIP-----N-EQVLIKARDAG---------------------------
YTNATTTDQH------------------------------------GLAKFSIDTNG-----I---
SDYSLNIKV----YHKEESSCIHSSCTAER----HAEAHHTAYAVYSL-SK-SY------------IYL------DTEAGVLPCNQ-------
IHTVQAHFILKGQV-LGVLQQIV-----------------FHYLVMAQ-------GSILQTGNHTHQVEPG---ESQVQGNFALEIPVEFSMVPVAKMLIYTI-L----PDGE-VIADSVKFQV-EKCLRN----K----VHLS--------
--FSPSQSLPASQTHMRVTASPQ-S--LCGLRAVDQSV----------------LLQKP--EAELSPS-----------------------------------L-----IYDLP-GMQDSNFIASSNDPFEDEDYCLMYQPI---------------------AREKD------VYRYV---------
RETGLMAFTNL-------KIKLPTYCNTDYDM-----------------------VPLAVPAVALDSSTDRGMYE-------------
SLPVVAVK--S---PLPQEPPRKDPPPKDPVIETIRNYFPETWIWDLVT-V-----NSSGV-TELEM--TVPDTITEWKAGALCLSNDTG-LGL-S-SVASFQAFQ---------------------------------
PFFVELTMPYSVIRGEAFTLKATVLNY-LPT--SLPMAVLL-------------------E-ASPDFTAV----------------
PVENNQDSYC--------LGANGRHTSSWLVT-----P-KSL--G-----NVNFSVSAEAR-QSPGPCGSEVATVPET---
GRKDTVVKVLIVEPEGIK--KEHTFSSLLCASDAE---LS---ETL--------------------SLLLPPT---VVKDS-A-RAHFSVM---GDILSS-AIK---N------TQNL-
IQMPYGCGEQNMVLFAPNIYVLKYLNETQQLT----EKI--KSKALGYLRAGYQRELNYK-H--KDGSYSAFG-----
DHNGQG-QGNTWLTAFVLKSFAQAR--------AF--I-FIDESHITDAFTWLS-KQQKD-SGCFRSSGSLLNNAMKGG-----VDD-------------EITLSAYITMALLESSLPDTDPVVS---K-ALSCLESS---WENIEQGGN-GS---
FVYTKALMAYAFALAG---NQEKRNEILKSLDKEAI----------------KE---D--NSIHWERP-QK------PTKSEGYL----

YTPQA----SSAEVEMSAYVVL-ARLTAQPA-PS----PED--
LALSMGTIKWLTKQ--------QNSYGGFSSTQ-----DTVVALDALS---KYGAAT-FSK-----SQKTPSVTVQSSG----
SFSQKFQVD--KSNRLLLQQVSL-PYIPG------NYTVSVS-GEGCVYAQTTLRYNVP---LEKQ---QPAFALKVQTVPLT--
--CNNPKGQNSFQ-ISLEIS---
YMGSRPASNMVIADVKMLSGFIPLKPTVKKLE---

RLGHVSRTEVTT-----NNVLLYLDQV-TNQTLSFSFIIQQDIPVKNL-QPAIVKVYDYYETDEVAFAEYSS-PCSS-D--D-
QNV---

>Human_PZP X54380_Human mRNA for pregnancy zone protein

------------------------MRKDRL----LHLCL--------VLLLIL--

------------------------------LSA--
-------------------------------------SDSNSTEPQYMVLVPSLLHTEAPKKGCVLLS----HLNE--TVTVSASLESGR--------
ENRSLFTDLVAEKDL-------FHCVSFTL--------PRISASSEVAFLSIQI----K-GP---TQDFRKRNT---VLVLN-TQ-
SLVFVQTDKPMYKPGQ-TVRFRVVSVDE-NFR--PRNELI-----------P--LIYLE------------NPRRNRIAQ----
WQSLKLEA-G------INQLSF-------PLSSEPIQGSYRVVVQTE---S----------GGRIQHP----------------------
FTVEEFVLPK---------------FEVKVQV-PK--II-SIM---DEKVNITVCGEYT-YGKPV-PGLATVSLC----RKLSRV----
LNCDKQEVCEEFSQ-------QLN--
SNGCITQQVH--TKM-LQ--ITNTGF------EMK--LRVEARIREEGTDLEVTA-NRISEITNIVSKLKFVKV---
DSHFRQGIPFFAQVLLVD-GKGVPIP-----N-KLFFISVNDAN---------------------------YYSNATTNEQ-----------------
------------------GLAQFSINTTS-----I--SVNKLFVRV----
FTVHPNLCFHYSWVAED----HQGAQHTANRVFSL-SG-SY------------IHL------EPVAGTLPCGH-------

TETITAHYTLNRQA-MGELSELS-----------------FHYLIMAK-------GVIVRSGTHTLPVESG-----------------------------
--------------DMKGSFALSFPVESDVAPIARMFIFAI-L----PDGE-VVGDSEKFEI-ENCLAN----K----VDLS---------
FSPAQSPPASHAHLQVAAAPQ-S--LCALRAVDQSV----------------LLMKP--EAELSVS--------------------------------
---S-----VYNLL-TVKDLTN-FPDNVDQQEEEQGH--CPRPFFIHNGAIYVPLS--------SNEAD------IYSFL---------
KGMGLKVFTNS-------KIRKPKSCSVIPSV----------------------SAGAVGQGYYGAGLGVVERPY----------------------
---VPQL---GTYNVIPLNNEQSSGPVPETVRSYFPETWIWELVA-V-----
NSSGV-AEVGV--TVPDTITEWKAGAFCLSEDAG-LGI-S-STASLRAFQ---------------------------------
PFFVELTMPYSVIRGEVFTLKATVLNY-LPK--CIRVSVQL-------------------K-ASPAFLAS----------------
QNTKGEESYC--------ICGSERQTLSWTVT-----P-KTL--G-----NVNFSVSAEAM-QSLELCGNEVVEVPEI---
KRKDTVIKTLLVEAEGIE--QEKTFSSMTCASGAN---VS---EQL--------------------SLKLPSN---VVKES-A-RASFSVL--
---GDILGS-AMQ---N------IQNL-
LQMPYGCGEQNMVLFAPNIYVLNYLNETQQLT----QEI--KAKAVGYLITGYQRQLNYK-H--QDGSYSTFG-----
ERYGRN-QGNTWLTAFVLKTFAQAR--------SY--I-FIDEAHITQSLTWLS-QMQKD-NGCFRSSGSLLNNAIKGG-----
VED------------EATLSAYVTIALLEIPLPVTNPIVR---N-ALFCLESA---WNVAKEGTH-GS---HVYTKALLAYAFSLLG--
-KQNQNREILNSLDKEAV----------------KE---D--NLVHWERP-QR------PKAPVGHL----YQTQA----
PSAEVEMTSYVLL-AYLTAQPA-PT----SGD--
LTSATNIVKWIMKQ--------QNAQGGFSSTQ-----DTVVALHALS---RYGAAT-FTR-----TEKTAQVTVQDSQ----
TFSTNFQVD--NNNLLLLQQISL-PELPG------EYVITVT-GERCVYLQTSMKYNIL---PEKE---DSPFALKVQTVPQT----
--CDGHKAHTSFQ-ISLTIS---
YTGNRPASNMVIVDVKMVSGFIPLKPTVKMLE--

RSSSVSRTEVSN-----NHVLIYVEQV-TNQTLSFSFMVLQDIPVGDL-KPAIVKVYDYYETDESVVAEYIA-PCST-
DTEH-GNV--

>Cavia_AlphaMacroglobulinPre gi|81871982|sp|Q60486|Q60486_CAVPO Alpha-macroglobulin precursor

----

-----------------------MGNKKL----LHPNP--------VLLLLI--

-------------------------------LLA---
--------------------------------------ADASISGKPQYMVLVPSLLHSGTPEKICLLLT----QLNE--TVTVKASLDTIR--------
ENGSLFMNMVAEKDL-------FQCVAFTV--------PQSPYPEAVMFLTVEV----E-GP---THGFRSRKT---VLVKS-KD-
SLVFVQTDKPIYKPGQ-TVKARVVSLDE-NFR--PLNELF-----------P--LIFIQ-----------DPKGNRVMQ----
WQNLKLER-G------LTQLSF-------PLSSEPLLGSYSVVVHKE---S---------GGRMHHS-----------------------
FTVEEFVLPK---------------FEVQVSM-PK--KI-TIL---EQEFTVSVCGRYT-YGKPV-PGNITMSIC----RNYNNP-
SACLSEESRAFCKKYNQ-------QLN--
SQGCFIQQVK--TND-FQ--LRRKEY------EMR--LRVEAKIREEGTGVQLTG-TGFSEITATITKLSFVKV---
DSYVRPGVPFFGQVRLVD-GKNVPMP-----H-KMITITASEAN---------------------------YHSNATTDEN---------------
---------------------GLVQFSINTTN-----M---IGTSLNIQV----
KHKDSTNCYDYQWLLEA----NEGASHTANAVFSL-SR-SF------------VHL------EPQLGKLPCHQ-------
TQTFKAHYILKGQE-LKELV--------------------FYYVIMAK-------GGIVQSGTYVLSVEQG--------------------------------
------------NTKGHFSVSVPVESDLAPVARVLIYAI-L----PSGE-IIADSAKYNV-ENCLDN----K----VNLS----------
FSEGQSLPASKTHLRVTASPQ-S--LCALRAVDQSV----------------LLRKP--EAVLSAS------------------------------------
S-----VYALL-PVKDLTG-FPGLLGQQEENDGE-CVSLYNTYIDGILYSPEPN-------INEKD------MYGFL---------
KDMGLKVFTNT-------KIQKPQLCAHVQKF-----------------------EVPTMAYSYSESSSFRSGPRR-----------------------
--VPAVGIAATYSEPPKETVRTYSPETWIWDLKV-T-----DSSGV-
AEVEV--TVPDTITEWKAGAFCLSNDTG-LGL-S-PTASLRAFQ----------------------------------
PFFVELTMPYSVIRGEAFTLKATVLNY-LPD--CIRISVHL--------------------E-ASPKFLAE-----------------
PKAKEQESYC--------VCGNERQTVSWVVT-----P-KSL--G-----NVNFTVSAEAL-ESSELCGNEKTVVPTY---
GKKDTIIKPLLVEPEGIE--KEETWTSLIRVSDTT---VS---EKL---------------------HLELPSN---VIQDS-A-RATVSIL------
---GDILGS-AMQ---N------IQNL-
LQMPYGCGEQNMVLFAPNIYVLDYLNETQQLT----PDI--KSKAISYLSTGYQRQLNYK-H--RDGSYSTFG-----
ENYRGG-QGNTWLTAFVLKTFSQAR--------KY--I-FIDEAHITQALSWLS-QKQKD-NGCFWSSGSLLNNAIKGG----
-VED-------------EISLSAYITIALLEMSLPDTHPVVR---N-ALFCLESA---WKSAKEGTH-GS---
HVYTKALLAYAFALAG---NQERKKEILKSLEDEGV----------------KE---D--NSLHWARP-QK------PKVSEGFL----
FKSQA----PSAEVEMTSYVLL-AYLTARPA-PT----PED--
LTSATDIVNWVTKQ--------QNSHGGYSSTQ-----DTVVALHALS---KYAAAT-FTR-----TEKAAQVTIKSSG----
TFSTNFEVN--HNNRLLLQQVSL-PTVSD------SYTITVT-GEGNVYLQTSLKYNVP---SEKG---TFPFALEAETVPQA---
---CDGPKAHTSFQ-ISLNVS---
YIGSRPVSNMAIVDVKMVSGFIPLKPTVKNLE--

---KSEHISRTEVSN-

----NHVLIYLDKV-SNQTLSLSFFVVQDIEVRDL-KPAIIKVYDYYETNEFAIAEYHA-PCSK-D--P-GNA-------------------
----

>Human_A2MG gi|308153640|sp|P01023.3|A2MG_HUMAN RecName: Full=Alpha-2-macroglobulin; Short=Alpha-2-M; AltName: Full=C3 and PZP-like alpha-2-macroglobulin domain-containing protein 5; Flags: Precursor

----

------------------------MGKNKL----LHPSL--------VLLLLV--

-------------------------------LLP--
--------------------------------------TDASVSGKPQYMVLVPSLLHTETTEKGCVLLS----YLNE--TVTVSASLESVR--------
GNRSLFTDLEAENDV-------LHCVAFAV--------PKSSSNEEVMFLTVQV----K-GP---TQEFKKRTT---VMVKN-ED-
SLVFVQTDKSIYKPGQ-TVKFRVVSMDE-NFH--PLNELI-----------P--LVYIQ-----------DPKGNRIAQ----
WQSFQLEG-G------LKQFSF-------PLSSEPFQGSYKVVVQKK---S----------GGRTEHP-----------------------
FTVEEFVLPK---------------FEVQVTV-PK--II-TIL---EEEMNVSVCGLYT-YGKPV-PGHVTVSIC----RKYSDA-
SDCHGEDSQAFCEKFSG-------QLN--
SHGCFYQQVK--TKV-FQ--LKRKEY------EMK--LHTEAQIQEEGTVVELTG-RQSSEITRTITKLSFVKV---
DSHFRQGIPFFGQVRLVD-GKGVPIP-----N-KVIFIRGNEAN---------------------------YYSNATTDEH------------------
------------------GLVQFSINTTNV---MGTSLTVRV----
NYKDRSPCYGYQWVSEE----HEEAHHTAYLVFSP-SK-SF------------VHL------EPMSHELPCGH-------
TQTVQAHYILNGGT-LLGLKKLS-----------------FYYLIMAK-------GGIVRTGTHGLLVKQE------------------------------
---------------DMKGHFSISIPVKSDIAPVARLLIYAV-L----PTGD-VIGDSAKYDV-ENCLAN----K----VDLS----------
FSPSQSLPASHAHLRVTAAPQ-S--VCALRAVDQSV----------------LLMKP--DAELSAS--
--S-----VYNLL-PEKDLTG-FPGPLNDQDNED---CINRHNVYINGITYTPVSS-------TNEKD------MYSFL---------
EDMGLKAFTNS-------KIRKPKMCPQLQQY--------------------EMHGPEGLRVGFYESDVMGRGHAR----------------
--LVHVEEPHTETVRKYFPETWIWDLVV-V-----NSAGV-
AEVGV--TVPDTITEWKAGAFCLSEDAG-LGI-S-STASLRAFQ----------------------------------
PFFVELTMPYSVIRGEAFTLKATVLNY-LPK--CIRVSVQL--------------------E-ASPAFLAV-----------------
PVEKEQAPHC--------ICANGRQTVSWAVT-----P-KSL--G-----NVNFTVSAEAL-ESQELCGTEVPSVPEH---
GRKDTVIKPLLVEPEGLE--KETTFNSLLCPSGGE---VS---EEL--------------------SLKLPPN---VVEES-A-RASVSVL---
---GDILGS-AMQ---N------TQNL-
LQMPYGCGEQNMVLFAPNIYVLDYLNETQQLT----PEI--KSKAIGYLNTGYQRQLNYK-H--YDGSYSTFG-----

ERYGRN-QGNTWLTAFVLKTFAQAR--------AY--I-FIDEAHITQALIWLS-QRQKD-NGCFRSSGSLLNNAIKGG-----
VED-------------EVTLSAYITIALLEIPLTVTHPVVR---N-ALFCLESA---WKTAQEGDH-GS---HVYTKALLAYAFALAG-
--NQDKRKEVLKSLNEEAV----------------KK---D--NSVHWERP-QK------PKAPVGHF----YEPQA----
PSAEVEMTSYVLL-AYLTAQPA-PT----SED--
LTSATNIVKWITKQ--------QNAQGGFSSTQ-----DTVVALHALS---KYGAAT-FTR-----TGKAAQVTIQSSG----
TFSSKFQVD--NNNRLLLQQVSL-PELPG------EYSMKVT-GEGCVYLQTSLKYNIL---PEKE---EFPFALGVQTLPQT---
--CDEPKAHTSFQ-ISLSVS---
YTGSRSASNMAIVDVKMVSGFIPLKPTVKMLE--

RSNHVSRTEVSS-----NHVLIYLDKV-SNQTLSLFFTVLQDVPVRDL-KPAIVKVYDYYETDEFAIAEYNA-PCSK-D--L-
GNA--

>Rattus_A2MG gi|119370261|sp|P06238.2|A2MG_RAT RecName: Full=Alpha-2-macroglobulin; Short=Alpha-2-M; Flags: Precursor

----

-------------------------MGKHRLRSLALLPLL--------LRLLLL--

----------------------------------LLP--
--TDASAPQKPIYMVMVPSLLHAGTPEKACFLFS----HLNE--TVAVRVSLESVR----
----GNQSLFTDLVVDKDL-------FHCTSFTV--------PQSSSDE-VMFFTVQV----K-GA---THEFRRRST---VLVKK-KE-
SLVFAQTDKPIYKPGQ-TVRFRVVSLDE-SFH--PLNELI-----------P--LLYIQ------------DPKNNRIAQ----
WQNFNLEG-G------LKQLSF-------PLSSEPTQGSYKVVIRTE---S----------GRTVEHP-----------------------
FSVEEFVLPK---------------FEVRVTV-PE--TI-TIL---EEEMNVSVCGIYT-YGKPV-PGRVTVNIC----RKYSNP-
SNCFGEESVAFCEKLSQ-------QLD---

GRGCFSQLVK--TKS-FQ--LKRQEY------EMQ--LDVHAKIQEEGTGVEETG-KGLTKITRTITKLSFVNV---
DSHFRQGIPFVGQVLLVD-GRGTPIP-----Y-ETIFIGADEAN--------------------------LYINTTTDKH-------------------
----------------GLARFSINTDD-----I---MGTSLTVRA----
KYKDSNACYGFRWLTEE----NVEAWHTAYAVFSP-SR-SF------------LHL------ESLPDKLRCDQ-------
TLEVQAHYILNGEA-MQELKELV-----------------FYYLMMAK-------GGIVRAGTHVLPLKQG--------------------------
-------------------QMRGHFSILISMETDLAPVARLVLYAI-L----PNGE-VVGDTAKYEI-ENCLAN----K----VDLV--------
--FRPNSGLPATRALLSVMASPQ-S--LCGLRAVDQSV----------------LLMKP--ETELSAS--------------------------------
----L-----IYDLL-PVKDLTG-FPQGADQREEDTNG-CVKQNDTYINGILYSPVQN-------TNEED------MYGFL---------
KDMGLKVFTNS-------NIRKPKVCERLRDN-------------------------KGIPAAYHLVSQSHMDAFL------------------------
--ESSESPTETRRSYFPETWIWDLVV-V-----DSAGV-AEVEV--
TVPDTITEWKAGAFCLSNDTG-LGL-S-PVVQFQAFQ----------------------------------
PFFVELTMPYSVIRGEAFTLKATVLNY-LPT--CIRVAVQL--------------------E-ASPDFLAA-----------------
PEEKEQRSHC--------ICMNQRHTASWAVI-----P-KSL--G-----NVNFTVSAEAL-NSKELCGNEVPVVPEQ---
GKKDTIIKSLLVEPEGLE--NEVTFNSLLCPMGAE---VS---ELI---------------------ALKLPSD---VVEES-A-RASVTVL----
--GDILGS-AMQ---N------TQDL-
LKMPYGCGEQNMVLFAPNIYVLDYLNETQQLT----QEI--KTKAIAYLNTGYQRQLNYK-H--RDGSYSTFG-----
DKPGRN-HANTWLTAFVLKSFAQAR--------KY--I-FIDEVHITQALLWLS-QQQKD-NGCFRSSGSLLNNAMKGG---
--VED-------------EVTLSAYITIALLEMSLPVTHPVVR---N-ALFCLDTA---WKSARGGAG-GS---
HVYTKALLAYAFALAG---NQDTKKEILKSLDEEAV----------------KE---E--DSVHWTRP-QK------PSVSVALW----
YQPQA----PSAEVEMTAYVLL-AYLTTEPA-PT----QED---
LTAAMLIVKWLTKQ--------QNSHGGFSSTQ-----DTVVALHALS---KYGSAT-FTR-----AKKAAQVTIHSSG----
TFSTKFQVN--NNNQLLLQRVTL-PTVPG------DYTVKVT-GEGCVYLQTSLKYSVL---PREE---EFPFTVVVQTLPGT--
--CEDPKAHTSFQ-ISLNIS---
YTGSRSESNMAIADVKMVSGFIPLKPTVKMLE---

RSVHVSRTEVSN-----NHVLIYLDKV-SNQTVNLSFTVQQDIPIRDL-KPAVVKVYDYYEKDEFAVAKYSA-PCST-D--Y-
GNA--

>Rattus_Alpha1Macroglobulin M77183_R.norvegicus alpha-1-macroglobulin mRNA, complete cds

----

-----------------------MRRNQL----PIPVF--------LLLLLL--

------------------------------LPR--
-------------------------------------DATAATGKPRYVVLVPSELYAGVPEKVCVHLN----HLNE--TVTLNVTLEYGV-------
-QYSNLLIDQAVDKDS-------SYCSSFTI--------SRPLSPS--ALIAVEI----K-GP---THHFIKKKS---MWITK-AE-
SPVFVQTDKPIYKPGQ-TVKFRVVSVDI-SFR--PVNETF-----------P--VVYIE------------NPKRNRIFQ----
WQNVDLPG-G------LHQLSF-------PLSVEPALGIYKVVVQKD---S----------GKKIEHS-----------------------
FEVKEYVLPK---------------FEVQVKM-PK--TM-AFL---EEELVVTACGLYT-YGKPV-PGLVTMKVC----
RKYTQSYSNCHGQHSKSICEEFSK-------QAD--
---------EKGCFRQVVK--TKV-FQ--PRQKGY------DMK--IEVEAKIKEDGTGIELTG-TGSCEIANTLSKLKFTKA---
NTFYRPGLPFFGQVLLVD-EKGQPIP-----N-KNLTVQVNSVR--------------------------SQFTFTTDEH------------------
------------------GLANILIDTTN-----F--TFSFMGIRV----
IYKQNNICFDNWWVDEY----HTQADHSAARIFSP-SR-SY------------IQL------ELVLGTLACGQ-------
TQEIRIHFLLNEDA-LKDAKDLT-----------------FYYLIKAR-------GSIFNSGSHVLPLEQG----------------------------------
------------KVKGVVSFPIRVEPGMAPVAKLIVYTI-L----PNEE-LIADVQKFDI-EKCFAN----T----VNLS----------
FPSAQSLPASDTHLTVKATPL-S--LCALTAVDQSV----------------LLLKP--EAKLSPQ-------------------------------------
S-----IYNLL-PQKAEQGAYLGPLPYKGGEN---CIKAEDITHNGIVYTPKQD-------LNDND------AYSVF---------
QSIGLKIFTNT-------RVHKPRYCPMYQAY--------------------PPLPYVGEPQALAMSAI-PGAGYR--------------
SSNIRTSS---MMMMGASEVAQEVEVRETVRKYFPETWIWDMVP-
L-----DLSGD-GELPV--KVPDTITEWKASAFCLSGTTG-LGL-S-STISHKVFQ-----------------------------------
PFFLELTLPYSVVRGEAFILKATVLNY-MPH--CIRIHVSL--------------------E-MSPDFLAV-----------------
PVGSHEDSHC--------ICGNERKTVSWAVT-----P-KSL--G-----EVNFTATAEAL-QSPELCGNKVAEVPAL---
VQKDTVVKPVIVEPEGIE--KEQTYNTLLCPQDAE---LQ---ENW----------------------TLDLPAN---VVEGS-A-
RATQSVL---GDILGS-AMQ---N------LQNL-
LQMPYGCGEQNMVLFVPNIYVLEYLNETQQLT----EAI--KSKAISYLISGYQRQLNYQ-H--SDGSYSTFG-----
DRGMRHSQGNTWLTAFVLKAFAQAQ--------SY--I-YIEKTHITNAFNWLS-MKQRE-
NGCFQQSGSLLNNAMKGG-----VDD-------------EVTLSAYITIALLEMPLPVTHSVVR---N-ALFCLETA---WASISN-
SQ-ES---HVYTKALLAYAFALAG---NRAKRSEVLESLNKDAV----------------NE---E--ESVHWQRP-KN------
VEENVREMRSFSYKPRA----PSAEVEMTAYVLL-AYLTSASSRPTRDLSSSD--
--------------LTTASKIVKWISKQ--------QNSHGGFSSTQ-----DTVVALQALS---KYGAAT-FTK-----
SNKEVSVTIESSG----TVSGTLHVN--NGNRLLLQEVRL-ADLPG------NYITKVS-GSGCVYLQTSLKYNIL---PEAEG--
EAPFTLKVNTLPLN--FDKAEHHRKFQ-IHINVS--
-YIGERPNSNMVIVDVKMVSGFIPVKPSVKKLQ---

DQSNIQRTEVNT-----NHVLIYIEKL-TNQTMGFSFAVEQDIPVKNL-KPAPVKVYDYYETDEFAIEEYSA-PFSS-DSEQ-GNA--
----

>Mus_A2M gi|338817897|sp|Q61838.3|A2M_MOUSE RecName: Full=Alpha-2-macroglobulin; Short=Alpha-2-M; AltName: Full=Pregnancy zone protein; Contains: RecName: Full=Alpha-2-macroglobulin 165 kDa subunit; Contains: RecName: Full=Alpha-2-macroglobulin 35 kDa subunit; Flags: Precursor

----

-------------------------MRRNQL----PTPAF--------LLLFLL--

--------------------------------LPR--
--------------------------------------DATTATAKPQYVVLVPSEVYSGVPEKACVSLN----HVNE--TVMLSLTLEYAM--------QQTKLLTDQAVDKDS-------FYCSPFTI--------SGSPLPY--TFITVEI----K-GP---TQRFIKKKS---IQIIK-AE-SPVFVQTDKPIYKPGQ-IVKFRVVSVDI-SFR--PLNETF-----------P--VVYIE------------TPKRNRIFQ----WQNIHLAG-G------LHQLSF-------PLSVEPALGIYKVVVQKD---S----------GKKIEHS-----------------------FEVKEYVLPK---------------FEVIIKM-QK--TM-AFL---EEELPITACGVYT-YGKPV-PGLVTLRVC----RKYSRYRSTCHNQNSMSICEEFSQ-------QAD---DKGCFRQVVK--TKV-FQ--LRQKGH------DMK--IEVEAKIKEEGTGIELTG-IGSCEIANALSKLKFTKV---NTNYRPGLPFSGQVLLVD-EKGKPIP-----N-KNITSVVSPLG---------------------------YLSIFTTDEH------------------------------------GLANISIDTSN-----F--TAPFLRVVV----TYKQNHVCYDNWWLDEF----HTQADHSATLVFSP-SQ-SY------------IQL------ELVFGTLACGQ-------TQEIRIHYLLNEDI-MKNEKDLT-----------------FYYLIKAR-------GSIFNLGSHVLSLEQG---NMKGVFSLPIQVEPGMAPEAQLLIYAI-L----PNEE-LVADAQNFEI-EKCFAN----K----VNLS----------FPSAQSLPASDTHLKVKAAPL-S--LCALTAVDQSV----------------LLLKP--EAKLSPQ------------------------------------S-----IYNLL-PGKTVQGAFFGVPVYKDHEN---CISGEDITHNGIVYTPKHS-------LGDND------AHSIF---------QSVGINIFTNS-------KIHKPRFCQEFQHY--------------------PAMGGVA-PQALAVAASGPGSSFR--------------AMGVP--MMGLDYSDEINQVVEVRETVRKYFPETWIWDLVP-L-----DVSGD-GELAV--KVPDTITEWKASAFCLSGTTG-LGL-S-STISLQAFQ----------------------------------PFFLELTLPYSVVRGEAFTLKATVLNY-MSH--CIQIRVDL--------------------E-ISPDFLAV-----------------PVGGHENSHC--------ICGNERKTVSWAVT-----P-KSL--G-----EVNFTATAEAL-

QSPELCGNKLTEVPAL---VHKDTVVKSVIVEPEGIE--KEQTYNTLLCPQDTE---LQ---DNW--------------------
SLELPPN---VVEGS-A-RATHSVL--GDILGS-AMQ---N------
LQNL-LQMPYGCGEQNMVLFVPNIYVLNYLNETQQLT----EAI--KSKAINYLISGYQRQLNYQ-H--SDGSYSTFG-----
NHGGGNTPGNTWLTAFVLKAFAQAQ--------SH--I-FIEKTHITNAFNWLS-MKQKE-
NGCFQQSGYLLNNAMKGG-----VDD-------------EVTLSAYITIALLEMPLPVTHSAVR---N-ALFCLETA---WASISQ-
SQ-ES---HVYTKALLAYAFALAG---NKAKRSELLESLNKDAV----------------KE---E--DSLHWQRP----------
GDVQKVKALSFYQPRA----PSAEVEMTAYVLL-AYLTSESSRPTRDLSSSD--
------------LSTASKIVKWISKQ--------QNSHGGFSSTQ-----DTVVALQALS---KYGAAT-FTR-----SQKEVLVTIESSG--
--TFSKTFHVN--SGNRLLLQEVRL-PDLPG------NYVTKGS-GSGCVYLQTSLKYNIL---PVADG--
KAPFALQVNTLPLN---FDKAGDHRTFQ-IRINVS-
--YTGERPSSNMVIVDVKMVSGFIPMKPSVKKLQ--

DQPNIQRTEVNT-----NHVLIYIEKL-TNQTLGFSFAVEQDIPVKNL-KPAPIKVYDYYETDEFTVEEYSA-PFSD-GSEQ-
GNA---

>Ciona_Tep gi|19032251|emb|CAD24311.1| alpha-2-macroglobulin homologue [Ciona intestinalis]

---------------------------------MNLRWRPACSLGTIYLLAT--

---------------------------------LSS---
--LATASNVYNIYFPKHIRPGFNISFTAAII----DNPN--TVQIHTAFRSMD--------
------NSFHVDSTDSVNSGSSSRISMNGL--------PIHYSGSHGFELNITG----TDLVTGAQLFFNSSTD---FQFQA-KS-
ISILIQTDKAIYQPGH-TVKFRAIALKP-DLK--PLQGN--------------ISYTFK------------DPRGNVVML----EPEVPLNH-
G------VAGGQF-------SLTKDAVAGMWKVEFMA----E----------GFKESLS-----------------------VEVKRYKLPK-------

--------FKVEVKA-PS--YI-HPQ---STGLTIKLDAKYT-FGKGV-
QGTGLLEVVGGYQYPVYHGFGGRFAPRPPTQNKITRRYPN--
---------------------------------FDGTVELLIT--NDEIRE--ELGWNG------ASESIITVTGSVTEALTREAFND-
TQRIDAKTTNVKVETLVK---PLTIKPGLKYSAYIQITE-VDGKPLPEDDRLA-
NNLLLNIEYRYPRGEPEPGTNTTVSTWYAYRWE-----ETRVFVIPPS-----------------------------------
GIVKVTIDAPSD--TFTSINFRP----YTNATMSQ------------
RWALQWTAERADSP-SN-SY------------LQI--------TTEENSVVP-------GNMATVTIRTTEAV-SE----------------------
-FTILIISR-------GEILSERKFQTLSGVP---
ENSHLFEFSVEYDMIPGVQVLASYV-R----DDGE-IVADYIKLTV-TAELEN----Q----VSIT----------
SSSTNIDAGEDVSIRVQTSSSGA--YVGARAIDQSV----------------LLLKS--GNDVSQE------------------------------------
-R-----IVTDL-NKYSVTQELNHMWRWWWWYPTP--------------------------SGASD------ASDVF---------
RKAGILVFTDA-------LVYQKPEASIYPFR---------------------------PIAFSLNGGFAERNII-------------------------------
---ATAAVDTSTPATPTRTRTLFPETWLWDEQI-S-----GADGS-ATFNT--
TAPDTITSWIFSAFSVSDQHG-LGV-S-EQHKVTVFR---------------------------------
NFFITLNLPVRVIRGELIIVQAIVFNY-LST--EVDAVLTL----------------------TESNKFVLLRPGNNS----------
AAVGFSRRIT--------IPASGSVSVKFPIR-----M-GTL--G-----EIPITMTAISE-------------------
IASDALTRKVFVQPEGIT--QCTSGSVLFQRMDASAPPDV---ESL---------------------NIQIPAG---IVPGS-E-
KVKLLVY---GDILGS-TMN---N------LGSL-
LRTPSGCGEQNMLGFAPDVFVTLYLHSAGKLD----AAT--RAKAFKHFQTGYSNELNYK-H--RDGSFSAFG-----
EGDA---SGSTWLTAFAAKCFMFAR--------ELRPT-LVSASVIDQALTFLI-NQQNT-TGTFREPGRVSHKAMQGG----
-VDS-------------PITMTAYVLITLKETNYAVKNRA-------VQEAAENA--RIYLENHLTSISD---NKYALAIVTYALHVA---
-GSSRANEALLALEALAT---------------------VQGGFKFWHDN------------
SESPDSYSSRWRPYYYNPPTNDIEMSAYALL-TYVRRND--
-----LNAGIPVMKWLASK--------RSSLGGYSGTQ-----DTVIAIQALS---KVAGLLVGNT---------QNLQISASH---
SNDPFTASYNINRENSIVFNSVNV-PAVDG------TVQVTAT-GVGVAVAQISVCYNTP-----NQPYEIEPFQCTNT-----
--VVSTALKKAKVNWCCSLR-----
PGDNATGMFLMEVNLPSGYTVNIDNE--

---RTRNPSAKLVEIDG---
--NGVNVVYYDELAPGRSVCADIELLNLGNVGGS-KARKVAASDYYQPKERVEALYQV-DEAPVVCDS-
CSTEDIAVCSVCADCVGCPGPAFTQWSEWSDCAFCGRSTSFRTRECRSPFSDNLAGHVCGGVDRESRRCVATFPC
PDTFDGLWFNMPRNFPSSNSVPFYAHQCRMERGSRQIREQIPGIALSGSQYLTCNNYDVNPNNNYTFSILVKPNR
FRSSGPTTIFSYGMEHNYARAHLEKVWWRSELRFKVRSDTGMREVRGVSSNLLRTDQWNHIVVAVPSGDGDDIR
MFVNGNAVGSTKSFTTRYFGKHGRNRFFLGQNTRGNAWARGYFQGGLAAVGTWRSVLTDQQITALYEAYRPAIE
SSDPLSVKLLRHFAVQQLLLCFQSPATIEDLYSRSAAPVTCPTAPISPLMPFLPIL--

>Ornithodoros_A2MPre gi|22901939|gb|AAN10129.1| alpha-2-macroglobulin precursor splice variant 1 [Ornithodoros moubata]

----------------------------------MARNP--------AIICLL--

--------------------------ISC--
---------------------------------LLASSHLAQSGYIFTAPKVLRSHRPAVFRLTLT----DVGVDGQVSVRLLNYNNE---------
-----SLVLAHQVYDIKQGELEQTLNFDV----------PEYSGSSAKIEVSG----T-FG---EYMFSGKKE---IDFQK-SK-
DHILIQTDKALYKPGQ-KVQLRVLPVTS-DLT--PVTDA-------------VAEIYVS------------SPSEARIAQ----
WKNVTFER-G------IVQFDF-------KLTEEPELGLWQIVVEL----P----------SQTVRQH-----------------------
FEVNEYVLPK---------------FEVTVTP-PS--YL-LAN---AKEIVWKICATYT-FGQPV-
EGTLLANVTYEKYHWETESFPYVEHEGP--
-----------------------INGCFDFVVN---TTALR--FNENY-------QVYKRLQLFAQVNETGTGITLNK-
TSYISRTSNPLELKFIAEEHGNNYFKPLMPYYGTLLVKK-PDGVPLP-----G-ELIQLCLLTQSEIIKTLWWRTDRRL------
-------SCKNYTSDAL------------------------------------GLVKFTLPPLQT--
TVVTVSVEA----TAVNYEAEKYDTYGTRIN---QPKTPSTCKRG-TR-QQ-QL------------CPN------QPSKDPLSCTS----
---NYKVQLLYTADPDA-DYL----------------------FHYQIVSR-------
GQILTDGTIPGKFTASEAVPAVVDDSYLQEEVRNESLPSNVAENVSN----------------
VGSLEIELAPDSRYVPLAKLLVFYV-R----PDGE-VIADSQEFEV-EKCLKN----N----VTFR----------
FGSESVQPATSAAIHLTGSPL-S--FCGVGVVDKSV----------------HLLKD--DNQLTKA------------------------------------
-K-----VYDLL-KRLDINRYTWPRQSSYDYCRRKSSSTGPRYARRVIWPGPRTSN------VEYVD------SITAF---------
DESGVIVLSDL-------TLETRPCREAIYDR----------PPSAFAAPASGPAGLFGPPAPSPPRMSIPSPVRP-----------------
--VAESFD---AALPGAPG-PAGVPAKSAVEVRTYFPETWLWELKE-L-----
DEHGN-LDFKE--KIPHTVTEWVGSAVCINNQDG-IGV-S-DPARIKAFQ----------------------------------
PFFASFSLPYSVIRGEVFPVTLSVFNY-LDK--CLPVELTL----------------------AESEDFEF------------------LEERTKTLC--
------VCGSK-VVEKFSVK-----P-KTI--G-----EVNVTVFAVGS-ENSEVCGDK--PVEKV---VAKDAVTQPLLVEAEGFP-
-KEETRSVFICSQ--GEAGEK---PQF--------------------ELALPED---VVEGS-A-RAYVAVS-------------------------------
-------------------------------GDIMGP-AIK---N------LDSL-VQVPTGCGEQNMVKFTPNVYVLDYLKATGKQD----
ADI--EKKAVENLKTGYQRQQKYR-H--SDGSYSAFG-----TNDR---QGSLFLTAFVIRSFKQAE--------RY--I-
PIDEKMLQQSVNWVL-NKQIPVNGCFNNVGRVLSSGLKGKV---NESN-------------PGPLTAYVLAALLEGGL--------
---A-HSNLTEGA----LHCIDAQK-NP---SPHNLALSVYATALAG---QDVSAK--LEALESLAV----------------HD---G--
ALTHWRNG-------------------------G----ASADVETAAYAVL-TYVKLGG--------QEN--------------------------------------
-----------------------LNKAQPIIKWMATK--------RNSRGGFSSTQ-----DTVLGLQALS---AFSSQL-SKD-----PV-
DLAVKVSGEQ-----VEESYDLK--EDSKLVLQQRKV-VNLPN------TLTGETT-GTGCALISTTLKYNVH---TAPT---

```
SQGFELAVT---PV-----------------------------------------------------------------------LD-PT-CTSAL-LRVCTK---
FDGE-QPSNMAVVELKLVSGYTISDDDIKEIY-------------------------------------------------------------------------
---------------------------------------------------------------------------------------------------------------------------
---------------------------------------------------------------------------------------------------------------------------
---------------------------------------------------------------------------------------------------------------------------
---------------------------------------------------------------------------------------------------------------------------
---------------------------------------------------------------------------------------------------------------------------
---------------------------------------------------------------------------------------------------------------------------
---------------------------------------------------------------------------------------------------------------------------
---------------------------------------------------------------------------------------------------------------------------
----------------------------------------------------------------------------------------------------
QKADVALKRHEIDR-----NQVNFYFEEV-TSEEKCFEVTVHKEFEVVDA-KPATVKVYDYYQLENAKSVSYSL-TGCE-
SPQ---------------------------------------------------------------------------------------------------------------------
---------------------------------------------------------------------------------------------------------------------------
---------------------------------------------------------------------------------------------------------------------------
----------------------------------------

>Limulus_A2M gi|2073373|dbj|BAA19844.1|

---------------------------------------------------------------------------------------------------------------------------
---------------------------------------------------------------------------------------------------------------------------
---------------------------------------------------------------------------------------------------------------------------
---------------------------------------------------------------------------------------------------------------------------
------------------------MEEIKWQKMSTL--------LFLLLL--------------------------------------------------------------
---------------------------------------------------------------------------------------------------------------------------
---------------------------------------------------------------------------------------------------------------------------
---------------------------------------------------------------------------------------------------------------------------
---------------------------------------------------------------------------------------------------------------------------
---------------------------------------------------------------------------------------------------------------------------
---------------------------------------------------------------------------------------------------------------------------
---------------------------------------------------------------------------------------------------------------------------
---------------------------------------------------------------------------------------------------------------------------
-----------------------------FTH----------------------------------------------------------------------------------------
---------------------------------------DVYSKSGFILTAPKSLTPGKSNILNLHLF----DIKT--NGFLRIGVKDQD---------
---DGNVVAETEVSFNKDNPSSSIQLTI---------PSGVEVKRPKLYANG----S-YSSPSSNDFFFEKD---INMHK-DK-
LIVFVQTDKPLYKPGQ-TVKVRILPTTP-DLK--LVPKE------------TIGSFQIE------------NPDGIVLGY----WPMLSFAE-
G------IAQFEL-------ALPDEPTYGMWRIKGNI----E----------DTEIYEN----------------------FEVKEYVLPK------------
---FEVKITP-PS--YL-LTN---ADSITWKICAQYT-YGQPV-EGTFVAETNVVKYNWEKEGVPVIHKEGL-----------------
----------------------------------------------------------------------------------IDGCLDVTVN---SSALG--FNEQR----
---LSYRAVNMFAEVTEKGTGIKMNA-TDSIYRTSNPLNIMYLEPTSGKGYLKPGLPFYGKLKVEK-PDGTPAP-----G-
EQIELCRFADRERWNRKRWLEEKIR-------------ACKEFTSDEA----------------------------------GIIKFTVPPQTP-----
-----------------------------------------DITSFRFKA----KALQYGKKDGDNKLN------QPQHSFTVSSWYSP-SG-SH--
----------LQL------EPITEEIECGK-------PLTVKFKYTTGEEK-KQK---------------------FYYQIMAR-------
NFIVDTGSFEHEFLLSEDKSGLTDETYLPIDVTALSLNPPNEPEWENNVIVPPH---------
IGETSLTLIPSFEMNPSAKILVFYV-R----EDGE-TVADSTKITV-KKCLRN----K----VGLK----------
FGEEKVLPGASSTLQLTASPY-S--ICGIGAVDKSV----------------HILSS--DNRITEE----------------------------------E-
```

----VFNKL-GGHDYYWPKQATSDYKYCEDYKFKQTEGEHEGSFSSGFTS---------TNYLD------SITAF---------
DEAGLVVISDM-------ELETRPCKPSGFED-----------------GGRPCPQYDVAFAAPQAANRIGGGGEA----------------
---GGFGGGIRKKTNKPVVEIRTYFPETWLWELQN-I-----
GATGE-LSLKR--DIPHTITEWVGSAICISEETG-LGV-S-EAATVKGFQ----------------------------------
PFFVSFTLPYSVIRGEKVPIIVTVFNY-LSE--CLPIKLSL----------------------EQSDKFEM-----------------
QNDTNSYTSC--------VCGGKSDTTRWMIK-----P-RSL--G-----QVNLTVYGASL-PNEAICGNQ--DYSTV---
TARDAATRQLLVEPEGFP--KEDTWSTFACPKDQNGKFTA---TS----------------------DLLLPED---LVEDS-A-
RGYVSIT---GDLMGP-AIK---N------LDHL-
VRLPTGCGEQNMVKFVPNIFVLDYLTATGSIT----DSI--KEKALNNMRKGYARQQNYR-H--PDGSYSAFG-----
NRDK---QGNLFLTAFVYRSFAQAE--------RF--I-LINKNKLNETENWIL-NRQRS-NGCFRKIGKLFNSALKGGI-
SSNDET-------------PAPLTAYVLISLLEAGY-----------K-NETVIDQG----ISCLEALS-NP---STYSLALFAYATSLA----
GHPSAKDYLAKLEERAI----------------TE---G--GKTFWKSP------------------SSGRYYWG----NSIGVEIAGYAVL-
TLLQHGG--------ASN--LAKVTPIIRWLAKQ--------
QNYRGGFYSTQ-----DTVIALQAMS---KFATII-YKD-----EL-DLEVGVESSG-----FEKKIMLT--KDNSILMQTFRL-
QTVPS------PVDFEAT-GSGCGLVQTSLRYNVN---TPPP---RKGFHLEVT---VK---
-----------------------------------RGLYRDCINAH-IATCVK---YDGKGGVSNMAVLEMKMVSGWIPDEESIKNIV------
----

---DREELNLRRYEVDG-----NQLNLYFSEL-TDQNLCFNFWLEQDIEVQET-
KPATIRLYDYYELEQEVVTSYSI-DENC-EK---------------LPPLP---

>Daphnia DappuP313404 pep:novel scaffold:Dappu1:scaffold_9:154585:163074:-1
gene:DappuG313404 transcript:DappuT313404 description:""

-----------------MRVGSEKDPTRDACDVIMLLRL--------LLAVAF--

--FTC--
---TLAQNDGTYTIVAPKVLRPSLDYHVSISLH----GGAP--
LTNLVVAIEGQQ-------------DGGGQVRNAQSATVEPNSTQVIKF--------QIGELGPGKYNLTARG------SG---
GLTFVNTTE---LEYSE-KS-HSVFIQTDKAIYKPGH-LVQFRVIVVNP-QLK--PSVVG-------------SLDVYMT------------
DGKGNRIKQ----WNRVFTKQ-G------VFASEL-------QLSDQPVLGDWNITAVV----S----------GQSFSKH------------
-----------FQVAEYILPK---------------FQVTIDL-PT--YL-TFN---ESKMVATVKAKYT-YGKPV-
KGNVTIAAYPQYRVSYIQPFFTEPVRKTVQ---
------------------------IDGTVDVDFN--LFKELK--LVD---------DFERDIRFDVTVIEGLTERKQNM-
SSLLTLYKYKYKMELIKT---SDSFKPGLKYTAFLKLAY-QDNTPIQ-------DANGVVIVKHGFSHNQDEY-----------------
--NRTEYPVPRN-----------------------------------GILELNFYPPVDE--
NVYTLGIET----QYQD-----------------LVEWFSTINRAQSP-SN-SF------------IQV--------ILKTENPKV-------
NEEIAIEVNSTAPL-DS-----------------------YIYEVMGR-------GNLVVARTVQAGNQRS--------------------------------
------------------HTFRFQATAAMAPVARVVVYYV-R----ADGE-VVADALNFDV-DGTFQN----F----VDIQ----------
VTPDSVEPGKAVDIVVKAKPN-S--YVGVLGVDQSV----------------LLLKT--GNDISRQ----------------------------------
--D-----VLDEV-KSYDS--TRRPD------FESWL---------PEVGGPKMCEE-------
LRLAAPTLSVDVSF--
---------------------SKKVEEPIALRQHFPETFLWLDITNL-----GTDGT-ARFVK--EAPDTITSWVITAFSLDTFHG-LGV-
IEQPAKMQVFR----------------------------------PFFIQLNLPYSVIRGEVVAIQAVVFNY-MNK--EITAELTF-------------
---------ENIGDFQFIDNGLEDNEISS-----EAIFRKKSVR--------IPAQDGTPVSFLIR-----P-TTL--G-----
NIDLRLTAKAA-------------------TAGDAIVKKLLVKAEGET--IYRNKAYLLDLRSIRNYN-----KNV--------------------
SVTIPFN---AVPGS-A-AVELSAI--VDIMGP-SIN---N------
LNTL-LRMPFGCGEQNMLLFVPNIVVTEYLKNIGQLT----DAI--SSKALGFMETGYQKELTYK-R--DDGSFSAFG-----
KSDA---AGSTWLTAFVARSFRQAQ--------PY--I-TIEDHVIEDSLKWLS-ANQAP-NGSFPEVGKVSHTDMQGG-----
SGK-------------GVPLTAYVLLAFLENK-A-----GL---R-YGPSMQKA--AEFLVKELPS-IT---DPYALSLVTYALHLA----
EVEERDAAFDMLQAKAN----------------TT---D-EEFRFWSKP-----------KSEKDKSN-PWSSLT----
TSVDVEMTAYALL-TFLQ-----------RGL---VIEALPIMKWMVAN-
-------RNSNGGFSSTQ-----DTVIGLYALA---KLAEKI-TVP-----NT-NINVKIKHDT-----GAETFSLN--
RENAMVLQKFKL-PPKTS------HVEISAV-GSGFSIIQVSTSYNLN---VTGE---WPLFTLDPQ---LF----------------------
--KNANQNRMQLT-ICSSFV------GEE-
SNMAVMEISLPSGYVMDEDSLPSLR--

---AIKDVKKVETKEGG-----
TGISLYFDKM-TRNTVCPTVQAYRVFKVAEQ-RKVPVVMYDYYDSSRRARVFYEP-VPAN-VCDI-CQS------
NDCKN-QC---SSYPGWSGDE--EGSKGWG--
---------TLDGRSNSRPTSGQQSLLPTLLCFVLSGLTSALLLI---

>Daphnia_2 DappuP320144 pep:novel scaffold:Dappu1:scaffold_33:1019904:1026535:1 gene:DappuG320144 transcript:DappuT320144 description:""

-----------------MIMLIALMINEWCKRNFVVTASAVVRPDTVYRVHIVVL----PGAP--DLVFKALITKGN-------------
GQHVASGSSYTIDAGTSSNLLLKI--------PASILFAGDYRLKLDS----FDPLHPQQPILIQESP---LVFHS-DF-
LSIIVQSNRKIFANGM-TVRFRIILTQM-DLK--PYTDP-------------ITVFIL-----------DPQGFVIRR----WPSRNPTN-
G------VVSLTY-------QLPSNPSVGSWTIRVEAM---Q---------QVHEHQ-----------------------FGVEHYYIPF--------
------FEVMPSA-PA--YV-LDS---DETYTVEVTTSVH-TQFVA-NGNLTVHVYARP--------VNSTANDY---------------
QLV---VEELFPW------------------------------------NYEFTYDVDLGQVKS--
AMGSNS------LVGWVIRVTTVLHSYFMGEARDG-FIETRVIGAQLKFKFSGAK--TAVFKPGMPFEGHVYVMY-
DDDQALTSDKLKG-ATITLRPVVTSTNGQTKTL------------------
PEIVVPAKGEYLSHQQQENANKYSKEYDNWMEHQIEDVKFNQFRTTGVYHFRF---------------------SVPK---------
-------------------DATSMRISA----TYKDG------D-GD------KATAELQAVPFYSA-KE-MY-----------VHV--------
ETSTAYGQL-------GENVVIHLRSNFGF-QV----------------------YSYVVVSK-------GLVIHGATETHPHPTK-----------
------------------------------------LV-TFSVPVSSEMAPTFKLVAMIV-S----PVGE-LVADSVTIPV-QSFNRY----K----
MNVT----------TVQMRDHSKETVQLVTRTRPG-A--FVGVSILRTVN---------------YVFQA--DNELTPS--------------
--------------------R-----VLKAL-YKLE-PFTKSVH--GVTWTD----RE---------GLKAERTEYFKGA-NPGAD------
TKRTF---------DLAGLLLFSDA-------RI-SQYP---
DIANCDQSNGYEPCL-----AA-GCFHKDRR--CDGKAD----CTDGSDEDDS---------
KLQVLERKTEWNFATCAGADFYDWLDGDWAWFDVP-T-----TDDGIEFQDQ---EVAADDEIWYINAFSFHHELG-
FAV-LDEVIVYDGSP---------------------------------PFYVLAESPNSVRRGETVSVRLMAINN-LKE--EVMMLIVL------
--------------EASDDYLFVET---GKDGEVEHYRPKQVGGEHHHMVA--------MKPESYQEVYLPIA-----PQVEQ--G---
--MITVKIQTHTQ--------------------IRQQVFQIDLEIMPEGAT--ISRHTSLLLDLKNRAHVLRFLDIPVE-----------------
-ESPIIPYSKF-RRYVFGS-P-RASITLC--GDVFGPVFPS------
TPITTDSL-LGRSLRGTEATLFNLATTLWSLHYLRLTNQLE----SAV--LYSGLNDMNVQMAELMRLY-S--
HDGSFRAHT-----NS-----NPSVWVTAWVIRVLGQSQ--FQDWE-NY--F-FVDRRLLGTSVQWIL-SHQAND-
GNFEDPDEYF-FPLALRSALP-NNA---------TR--KGSLTAYVLIGLMKCRESLEGSLRV---T-TAIAELRA--
INYLERAISH-LA---DPSEIAIVTYALSIA----NSPTKEAAFYLLHNIKR--------------------EGEGMVYWSREPVP------
SNQILYENQRPFLQPRLPMHQDAVAVEATSYALL-VYLARDG-I---
-----------GDLQERVVTWLNTM--------RMVDGGFVSIY-----DSIVAMEALT---EYAYRA-RLR-----
DITDMSVVVEASA----SPDATELMKINSDTLATVHRMDI-PNVWG------HVNVIGR-GAGQAVLQLKVQYGID-

WEELRDNPKRRYFDLYVE---
ESYSHFRNKSHITVDACVKWM---ATDEMRTSGPATLEVEMPSGYELIQSDANELV--

------------------IR---NNYTFIRDVFIGR-----GKIIWMFDRV-
GEERLCIKYPIHRWFPVANMTLYRTAIIYESHIPEHFEMQVFNATPLYVLDICEVCGS---
--YQCPYCPYFSASLPT---FSSPFLIV-
CSLVAIALAYFY-RPHSFL---

>Daphnia_3 DappuP320145 pep:novel scaffold:Dappu1:scaffold_33:1029241:1036715:1
gene:DappuG320145 transcript:DappuT320145 description:""

--MFVL----PDAE--GLIVKALITKDG-----------------GQHIASAIE-
TIDSGVSHNLLLKI--------PGSL-TDGQYQLKLDG----YDLLQPQKSVFSKRGH---LEFHP-DF-
LSILVQTNRKIYKNEM-KVRFRAIITQLVNLK--PYSDP--------------VTVYIL------------NPEGFVMRR----
WPSRYPTT-G------VITLLY-------QLPTFPSEGKWTIRVEAM---S----------QIHDHH------------------------
IIVERFYYRF---------------FDVIPSA-PA--YV-LDS---DESYTAAVTTSFH-PGRVA-SGNLTLQVYARP--------
VNSSLADF----------------RFV--RQDFHQW--
-TYDFTYNVQLEEVKR--SVGVSS------LTGWVIRVAMTLYHHFEGESRNG-FIETRIIRAQLKFRFGGSK--
TAVFKPGMPYEGHVYLMY-DDDEAVSPEKLAG-ATLTIRPVATTSNGQLKTL-----------------------
PEIVVPRQGEYHRSSDDDHLNYYGNKYDVWMDRQMEDAEYKTFRQFGIHHFRF---------------------------TVPK----------
--------------------------DATSLKITA----YYKDD------E-GD------QVSVDQQAVAFYSL-GN-FF---------------VHV---------
STSTDEGLL-------GHFATIHLRSNFAF-QD-----------------------------YSYVISSK-------GLVVHGATETLAHATK----------------

------------------------------------SL-TFSVPVSTDMAPTFKLVAMMV-S----PLGD-LVADSVTIPV-QSINRY----K----
TNLT----------MVQTKDHSKLTVQVVTRTLPG-A--FTGVSLIRSAN----------------YMFQA--DNEITYS-----------------
-------------------R-----MIRAL-YQLE-PFSRSVS--GITWTD----RE---------GSKADKSQYFKSA-NPGAD------TKRTF--
-------NLAGLLVFTDA-------RI-SQYP---
DTANCNKANGYEPCM-----AV-GCFHKDQR--CDGKSD----CTDGSDEDDC---------
PLTKDDRMDFRMFRRNRFHDFYDWLDGDWAWFDIP-V-----TDDGFDFNDR---
SVALTNEVWFLNAFTFHRELG-FAV-VDDVVEYEGSL-----------------------------------
PFYVSVESPSFIRRGETVGIRLLFINN-LQV--DTMGLIIL--------------------EASDDYCFVET---
EAHGEVAHYHPSLVCAEKHHVVT--------VKGGTTTEVFMPIS-----PRIEQ--G-----SITVKIQVLSH-------------------
IFEQNLEIGLEVLPEGAT--ISRHTSLLLDLKNRAHLLRFLDIPVE------------------ESPIIPYSKF-RRYVFGS-P-RASITLC-
--GDVFGPVFPS------TPITTDSM-
LSRSLRGTEANLYNLATTSWSLHYLRLTNQLV----SDV--LYSGLNDMNVQMAELMRLY-H--HDGSFRAHT-----NS-
----NPSVWVTAWVIRILGQSQ--FQDWE-NH--Y-YVDQQLLTNSVKWIL-QFQRRD-GSFGETENYI-SPLDRRLLPR-
YNS---------TF--KGGHTAHVLLSLCATSESLDGAVKV---A-TASAKVRA--VRYLERILDI-LT---DQYELAIVTYALQIT--
--SSPAQEAAFGRLHILRR---------------------EEEGMVYWSREPVP------
SNKILYENQRPFLQPRLPMHQDAVAVEATSYALL-VYLARDG-I---
----------GDLQERVVSWLNTM--------RMVDGGFVSIY-----DSIVAMEALT---EYAYRA-RLR-----
DITDMSVVVEASA----SPDATELMKINSDTLATVHRMDI-PNVWG------HVNVIGR-GAGQAVLQLKVQYGID-
WEDLRDTPKRRYFDLYVE---
ETYSHFRNKSHITVDACVKWL---AMDEAPVSGSTTLEVELPSGYRIVQSDADQVL---------------------------------------
----

-------------IH---KNYTFLRDVFVSD-----EKIVWTFDKV-
GPERLCFNYLIRRWFPVANGTMYRSAIVYESHSPEHFEMQVFNATPLYVLDICEVCGS----------------------------------
--YQCPYCPYFSAA-PVVVSFNP--
IIVFCSLLISANICLNWSTS---

>Daphnia_4 DappuP61510 pep:novel scaffold:Dappu1:scaffold_101:194522:195044:-1 gene:DappuG61510 transcript:DappuT61510 description:""

------------------------LSGL-VRLPMGCGEQNMILFTPNIYVTKYLEATNQLE----PSF--
KTKAVNFMKSGYQRELTYR-H--DDGSYSAFG-----KSDE---NGSLWLTAFVVKSFAASR--------RY--I-
HIDDNELQTSVHWLQ-SKQLE-NGCFPVIGTVLHRDLK--

--VPSLF-----------------

---PPYSKQETDF--

>Daphnia_5 DappuP229736 pep:novel scaffold:Dappu1:scaffold_262:35838:47396:1 gene:DappuG229736 transcript:DappuT229736 description:""

--------------------------MKPFFGAASSLL--------LLVLLD---

------------------------------STAAQQRQQQQQQQSPPTQSTRINFQDPIR--------DNNQRPNLFNNNIDPF------
SRRPFGVGD---NT------------------------------------NAIFE-----------------EP------------
TYFIVASRIIRPSQIYRVAVSIL----SSPI--TLSVRASILRN---------------GVDIGSATQ-ECKPGIPETLLIKI--------PSTT-
VPGQYRLRVEG----NTDRALGGTAFLNETL---LNFSQ-RS-MTIFIQTEKPVYHQSQ-IVRFRTIPINT-ELR--AFDDA---
-----------VDVYMI------------DPRGFVVRR----WLSRQSNF-G------AVSLDY-------ALSDQPTFGEWTIRVTAQ---G-
---------QTEEEH------------------------FHVEEYYQTR---------------FEVNVTM-PA--FY-FDS---DDFIHGTVMANYT-
SGAPV-RGNLTLKATFKP--------IRATPLIP----------------DRP--
GSIRDSVIERNLTFKE--------------------------------FRGFYTFKYP--MAELQR--MVP--K------
LDGIELRITAIVGDRYREDYVEG-FSTARIYNSSLKLNFLGGS--PQVIKPAMPFTCFLAVSF-HDGSILPAERFRY-
DRLEIRPTVQFRSSASRTL-------------------EVREAKALPE---------------------------------
QPGIWEFTIDLRSELAQTTGSNVGTGDKRVKDLVN----------------------------EISALKLIA----NYKDG------Y-GE----
--RATAEMLAITHYSI-GD-KH------------IKV--------LTSTRSPKV-------GEYLILHVRTNFYA-ET----------------------
FNYIVVSK-------GIVLVTGSQSS---SP---SVTTLSIPLSAEMAPVATVVIYHV-
A----KYGE-VVADSLTIPV-NGISRN----N----FTLT----------LNPMKDKTGDTVEVVVLGDPG-S--YVGISAIDKGF-----
-----------FNMQA--GNELSYA-----------------------------------E-----VITKM-TSFD-ESLNGTL--RHYWTS----RE------
---G-DAETVVNYPAS-TFGID------ANRTF---------EYASLVVFTDA-------FV-VRRP--------------------------------------
--------------------------D--ACNITLGFLACM-----SG-SCYRSERR--CDGQYD----CDDRSDEAGCPSN--------
IRRELAQYRLKRINRLLRMYE---NSWLWKDIN-I-----GPHGHSIFSV---QIPEIPTHWVVSAFSVSPKNG-FGL-
VRSSREFAGYR---------------------------------PFYMNVEMPTNCRQGEQVGIRITLFNY-ATI--EADVVVTL-----------
---------ADSPDYKFVHV---EEFGEVKSYEARTSRGEKQHLTW--------IPAQGSQVVYIPIV-----P-TKL--G-----
EIEVTIQAKSL-------------------IRKDQVVRRIRVEADGVP--QFRHTSVMLDLSNRAWFLQYVYVNVT----------------
--ETPIIPYEKD-RYYVFGS-N-RARVSVV---GDVVGPAFPN-----
-MPVNATSL-LTLPMDCAEQNMFSFAANLYTVKYMRLTTQRK----REI--DRQAFYHLNIGYQRQLSFQ-H--
RDGAFSYFRADWDYSS------KSVWLTAFCARILAEAN--FNEWE-NY--L-YIDPGVIAKAVDWMI-QFQSPE-
GAFYEVAPRF-ADRKMNSTTSWGFN----DPIR-YR--NISLTAHVVIALTTIRD-LPGELGP---R-VAVSRSRA--
ISWLDRNLNL-LEKFGDPYEVAIVAYAMMLA----KSTSAEAAFGLLQQKAR--------------------
EDGGYKYWGREPVP------LPAQRLENQRPFLLPRLPNAFDAANVETTAYALL-TYVGRQE-L--------------------------
---FVEPIVKWINTQ--------RLTDGGWASTQ-----DTIIATQALI---EYTVRH-RIR-
----EVTSLTLHVEATS----NPQLQRTMYITENNLATPQFMDI-PNAWG------TVKIQAR-GAGYAIAQLSLQYNVD-
VNRFVTPPAVRAFSVVPR--LSFSG-
RNNSHINYDICSSWT---NQRESNQSGMAVLDVAVPTGYYMQQQVLDEYI--

------LS---RKVRNLRRAKFLD-----HKVVFYFDAL-
DGDDTCVKFTFERWHPVANMTRYLPVRVYDYYAPERFNETVVQTYELYVMDICQVCGS--------------------------
---YQCTYCPTYNHA---
TRGLMTSSLTMIAALLISLVFTAIYQR--

>Daphnia_6 DappuP320142 pep:novel scaffold:Dappu1:scaffold_33:1001195:1007497:1 gene:DappuG320142 transcript:DappuT320142 description:""

--MRQI--------
PASV-TNGRYRLKLEG----YDTLHPQKAVFIKESQ---LDFHS-DF-LSIVVQTNRKVFRNGM-KVRFRVILTQL-NLK--
PYTDP-------------ITVFIV-----------NPQGFIMRR----WPSRNPRN-G------VATMSY-------
DVPSYPTMGNWTIRVEAM---Q---------QVHEHQ----------------------FVVERYYIPF--------------FEVIPSA-PA--
YV-LDS---DDKYTAGVTTSFH-RARVA-NGNTTVQVHARP--------INTTLRAY---------------RLV---------------------
-----------------------SEEHPPWIVIHLARLC-----------------------WLQTHEFSYEVDLNDVRS--AMGTKS------
LAGWVIRVTTSVHNYFMGETRRS-FIETRVIRAQLKFKFSGAK--TAVFKPGMPFEGHVYVMY-DDDQALSPEKLAA-
ATLTIRPVVTTSNGQLKTL------------------
PEVIVPATGEYLSNPKGASKKKYGADFKQWMERQAEDAEFGQFRRTGVYHFRF--------------------SVPK---------
------------------DANAIKITA----TYKDG------Q-GD------KATAEMRAVAFYSA-NE-MY-----------VHV-------
GTSTEQGRL-------GENAVIHLRSNFGF-QV---------------------YSYVVASK-------GLVIHGATETHPHPTK---------
-----------------------------------LV-TFSVPVSSEMAPTFKLVAMIV-S----PVGE-LVADSVTIPV-QSFNRY----K----
VNVT---------TVQVRDHSKETVQLVTRTRPG-A--FVGVSLLRTIN---------------YIFQA--DNALTPS-----------------
-----------------R-----VLKAL-YKLE-PFTKSVH--GVTWTD----RE--------GLKAERTEYFKGA-NPGAD------TKRTF---

------DLAGLLLFSDA-------RI-SQYP---
DIANCDQSNGYEPCL-----AA-GCFHKDRR--CDGKAD----CTDGSDEDDC---------
PPAVDDKMNFRIFRRSRYHDFYDWLDGDWAWYDIP-T-----TDDGIEFHDR---GIAATDESWYINAFSFHPEFG-
FSM-MDEVVAYDGSP---------------------------------PFYFSVESPSAIRRGETLGVRLMAVNN-LKE--EVMTLIVL----
----------------EASDDYLFVET---GDDGEVEHYRPKLVSGERQHMVA--------IKAQSYREVYLPIS-----PQVEQ--G-----
TITIKIQTISQ-------------------IRRQTFQIDLDILPEGAT--VTRYTSLLLDLKNRAHVLRYLDIPVE-----------------
ESPVIPYSKF-RRYVFGS-P-RASVTLC--GDVFGPVFPS------
TPISTDSL-LSRSLHGTKANLYNLASTLWSLHYLRLTNQLK----SRV--LYRGLNTMNIQMAELMRLY-N--
YDGSFIAQK-----AS-----DPSIW--AWVIRVLGQSQ--FQDWE-NH--F-FVDRRLLGTSVQWIL-KNQNRD-
GTFEEPDDYP-FPLEIQSV---RNA---------TR--KVALTAHVLIGLHHCSESLEGPLKV---L-TATAKARS--
VNYLERAVSR-LT---DPYEVAIVTYALTLA----NSPAKESAFNGLHKIKR--------------------ESDGMVYWSREPVP----
--SNQILYENQRPFLQPRLPMHQDAVAVEATSYALL-VYLARDG-I---
-------------GDLQERVVSWLNTM--------RMVDGGFVSIY-----DSIVAMEALT---EYAYRA-RLR-----
DITDMSVVVEASA-----SKMGKTMRINSNSLANVHRMNI-PNVWG------HVNIIGR-GAGQAVLQLKVQYGID-
WEELNDVPKRRYFDLSVD---
ETYSHFRNKSHVTVEACIRWL---ATDEAKTSGPTTLEVETPSGYGFVQSDANELV---------------------------------------
----

------------IK---NNYTFIRDVFIGR-----SKIIWVFDRV-
GEERLCIKYHVHRWFPVANMTLYREAVVYESHMPEHFEMIIFNATPLYVLDICEVCGS---------------------------------
---YQCPYCPYFSGS-
PVSAKLSSPLLVIFGSLLANAFVHYLVHRHQFRPTL--

>Daphnia_7 DappuP320143 pep:novel scaffold:Dappu1:scaffold_33:1010121:1016691:1
gene:DappuG320143 transcript:DappuT320143 description:""

---MCILML---

--NFFVTASSILRPDTLYRVHVVVL----PGSP--DLVFKALITKRS----------------
GQHVASASSDSVDAGTSTHLLLKV--------PASI-SDGDYRLKLEG----YDVQHPQQAVLIKESP---LVFRS-DF-
LSIIVQLNRKIFANAM-TVRFRIILTQM-DLK--PYTDP-------------ITVFIL-----------DPQGFIVRR----WPSRNPTN-
G------VVSLTY-------QLPPNPSVGSWTIRVEAM---Q---------QVHEHQ-----------------------FGVEHYYIPF--------
-------FEVMPSA-PA--YV-LDS---DETYTVEVTTSVH-TQFVA-NGNLTVHVYARP--------VNSTADDY---------------
QLV--VEELFPW---NHEFTYDVNLGDVKS--
AIRSRS------LVGWVIRVKTVLHSYFMGEARDG-FIETRVIGAQLKFKFSGAK--TAVFKPGMPYEGHVYVMY-
DDDQALSSEKLAG-ATITLRPFVTSTNGQMKTL------------------
PEITVPAKGEYLSQKQDSNKMDHNNEFDNWMEHQIEDVKFNQFRATGVYNFRF-------------------------SVPK--------
-------------------------DAKSMRISA----TYKDG------D-GD------KATAELQAVPFYSA-KE-MY------------AHV--------
ETSTAYGQL-------GENVIIHLRSNFGF-QV---------------------YSYVVVSK-------GLVIHGATETHPHPTK------------
---LV-TFSVPVSSEMAPTFKLVAMIV-S----PVGE-LVADSVTIPV-QSFNRY----K----
VNLT----------TVQVRDHSKETVQLVSRTRPG-A--YFGYSLLRTLN---------------YIFQA--DNELTPS------------------
-------------------R-----VLKAL-YKLE-PFTKSVH--RVTWTD----RE---------GLKAERTEYFKGA-NPGAD------TKRTF----
-----DLAGLLIFSDA-------RI-SQYP---ETTNCDQSNGYELCL--
---AA-GCFHKDRR--CDGKAD----CTDGSDEDDCKSFTFQ---
SPREDDKMEFRLMRWSRNLDFYDWLDGDWAWFDVP-T-----TDDGIEFQDQ---
EVAADDDSWYINAFSFHPEFG-FAM-LDEVVVYDGSP---
PFYVLAESPSAVHRGETLGVRLMAINN-LKE--EVMTLIVL--------------------EASDDYLFVET---
GDDGEVEHYRPKLVGGEHHHMVA--------MKPESYREVYLPIA-----PQVEQ--G-----SITVKIQIHTQ------------------
--IRRQIFELDLEIMPEGAT--ISRHTSLLLDLKNRAHVLRFLDIPVE-----------------ESPIIPYSKF-RRYVYGS-P-
RASITLC---GDVFGPVFPS------TPITTNSM-
LSRSLRGTEATLFNLATTLWSLHYLRLTNQLV----SDV--LYSGLNAMNVQMAELMRLY-S--HDGSFRAHT-----NS---
--NPSVWVTAWVIRVLGQSQ--FQDWE-NH--F-FVDRRLLGTSVQWIL-SHQAND-GSFEDPDEYF-FPLELRKASS-
NNS---------TR--KGSLTAYVLIGLMKCRESLEGSLRV---T-TAIAELRA--INYLERAISH-LA---DPSEIAIVTYALSIA----
NSPTKEAAFYLLHNIKR---------------------EGEGMVYWSREPVP------
SNKILYENQRPFLQPRLPMHQDAVAVEATSYALL-VYLARDG-I---
----------GDLQERVVSWLNTM--------RMVDGGFVSIY-----DSIVAMEALT---EYAYRA-RLR-----
DITDMSVVVEASA----SPDATELMKINSDTLATVHRMDI-PNVWG------HVNVIGR-GAGQAVLQLKVQYGID-
WEELRDNPKRRYFDLYVE---
ESYSHFRNKSHITVDACVKWM---ATDEMRTSGPATLEVEMPSGYELIQSDANELV---

---------------IR---NNYTFIRDVFIGR-----GKIIWMFDRV-
GEERLCIKYPIHRWFPVANMTLYRSAIIYESHMPEHFEMQVFNATPLYVLDICEVCGS---

--YQCPYCPYFSGS-
PVSAKLSSPLLVIFGSLLANAFVHYL-HRHQFRPL--

>Strigamia SMAR009298-PA pep:novel scaffold:Smar1:JH431880:45283:54968:1 gene:SMAR009298 transcript:SMAR009298-RA description:""

---------------------------------MLLLG--------FTLCFL---

--------------------------LST---
--------------------------------SLAREDDQRPMYTITAPNSISSRSFYTVEVFIH----HSHR--PVNVTVELRKPM--------
EGEGHYYSFAPLSSMIRP----GDGKSFRF--------KTKLLKSDSYTLHVTG----N-LG----FKFDETMI---IPVNP-KK-
ATVLIQTDKGLYQAGQ-T---G------VFSSSM-------
DLSDNPVLGIWKFEVLNE---E----------TVTTVQT----------------------FIVQEYILPT---------------FEVSLRTQPT--FI-
TYN---QSDVLVIVDAKYT-HGQAV-KGKVSLHIRDEYCTYSFVELCDAKMYKSE---------------FKN---------------------
--IVDEASISVN--FVRELE--LPKHIYG-----
FGFRNFKIEAIVVEQLTQIARNA-STVITMHSEAIKVELVP----AGSFKPGLNFTQLIKVVY-HDDTPLL-----N-
GDGDVNVTVQFSSAHEDS--------------------IYYSLPMTKH-----------------------------------GILRLEVAISK-----------
---------------------------------------KATSLTFQA----KYK------------------GITGYASVPRSASS-GNYEY------------
MKL--------TQLTPVAIV-------DKVMQLEIQSTRNL-ISR---------------------LILLVIAR-------
GDINYVKTYPPADQLS--QIVQFRIHETMAPVMTVLVYYV-S----GCGN-
LVVDAISIEV-EGLFRT----P----VSIK----------LKHNTTEPGKDIQLEIKTRPK-S--FLGILAVDQSV----------------LLLSK-
-GNDITEQKEIIEKVPYSLGKNGMPDNSKSDPGAGKSEFSGMPDK-----ILDDM-
AQYEMSRSYFYNPLIADKNSAFPHPW----------------------SSATT------PLQVF---------RDGGLVIKTNF-------
LEDVDNLAVPAAIG------------------------GMTIDKAIVDNALELQDDAP--------------TEDNFRSG--------------------
---DSEGHIRKSFPETFLWIDAE-S-----SSSGK-YTLHK--
KAPDSTTSYIVTAIAINEKTG-LGI-A-YPTKMTIFK--------------------------------
KFFSTLSLPLSIQRGEFVSIQLVTFNY-YDH--PVKGTVTL-------------------H-NEHSDFAFVVNKKEI----------
AETSKHALVN--------IPAKDGVVSTFLIS-----P-KKL--G-----YIELSVSSTSK-------------------
YGSDNVVRKLLVKAEGNE--VTVNKPYLIDLSGMVQQFS----TDI--------------------PVAYPNT---VVSNS-Q-
TLSVSVT---GNILGA-SIQ---N------LNKL-
ISLPQGCGEQSMVNFAPDVVIATLFKQVGKFP----INL--QKKVIGFLEKGYQTQLTYH-R--TDGAFSAFG-----NTDV-
--TGSTW---

>Strigamia_2 SMAR006270-PA pep:novel scaffold:Smar1:JH431683:16032:34051:1 gene:SMAR006270 transcript:SMAR006270-RA description:""

MAVGTMELLFFDTFSHESNEELNLDLVQFPDPVYINEVRIIPLGTRVQADFPGGVRLGATNPSQFELEFFVNDLSKT
GASTFEGLGSLEYKQNVDIQLTFKKKIPTDGLVLRGWYTTITIAIYGSISKIKERTSPPPPPPPQATQKIRSTEKEAIDAT
PHTDWDRDSPSETDSIIQSTAEQVAFADPHTPNQEHGHYPADHPKPTYPERSTYPEHKSYDYDFPSGRSWSSTVTE
HQTSNTNKLDNFPKERNVYGRSSYSRDNYEEKKPNWDHKDRDRDSRDPRTQQREKERTRDGREYREIDRVEFPLV
QEPENEPAWEKLAESVEKDSPTERTRYPEREEELSPEVAAEREFRERTIKRDAGKGEPLRPDSKRPRTPPLSSSPKRR
PRTPPLPSEGDQHRARGPCSPPPPKDEMEPGEVADDKNLFDPLTPDDSPDLTVDNLSDGEIHEGETNDGGYSEFIY
DEILSDEEFMSDGEERNLDLPELYYEDSWNYISSFNPYQCELGSLLYFSDPSLTHYEIEKKKMGCPPDEIAMETAQKLL
KTLEYFDDKEHSEKWVEAMELLPSMLVPGLAILKQSAELKGLVDVLLDWLCEGLNFEKAIEQSQAVYKVRHIKAGIR
LAIALSNCDYEMTHQLLEKNVQKKLLDLYGKEYMALSIKLLIVQALDASTRTEEGMKYFLGLSENQFAGPEDKSDYQ
RLLNFVLGKQSSRAAVAVSALLKKIHVFEIISKFCASVEKIIDNSAVFEISPNKPESAKDCDNELSNAISADNDIDNFSIN
YLNDDEIEDIISYLIEINKMLGHAQQFIQDDGRDEASEETPHQLGLQLVYHLQVLQYVDMLLDFHSKDSTKKELDDP
EALTVLQPLYTMVFLPIGRQVVVDVLSIEHNLNALVPFLELTGDEQNEIKIRKSASMGYATELLLVITHYSENVSLLKN
FGEKLLNICTQESSSKLQEIVSWFSVMKRIPSYSADAAAPLCTIIKNLIEEVLTFPGELITSVRILKDLCIPPSTNLKPEEQ
REELKYRYAIIQFIANDGLSNFITILQKICDAYQYPYPQNYAVIGPQWGLLLALIKPIVSLMKHSLCYLIYCRNAEYKDL
MAVPIILRVYSFLNILPITSSFYPVAQGIQSEIIEMVLAYTQPIMTISDSDEALNKSLWSQMLQEVLKFATTLPSNFLSG
VTILSELLPLPLPIQTRESLTNEDILQATNARKLWSAHLHSMSNEIKDIIKKLLGTSLPLLQQQLKRISVQLADLGASCSL
VVTRAVFDIVLENLEDNEPEDLENKECFSNSHTYRLLNMLDCLANQPCIKIALIQLLRANTKLDEKYAQLLPKLTSLLN
TASDKTVHLQIQECILSIFQSLCDYEIILGSENSLSPLDQLGNALPAKDQMVAICLALLDHVSNSSHSFSTTLLAFRNLI
MILETDLGFYCLKLNFDKKPEILHSILNRCSTSFNKDNVDSVAALNICLDFCRLLYTDDSAQGVPRTLFFTISELGFYIE
WKVNEAKEEIPIHPLIKIEKILLEFVKDDDALDVQSAENVSSLILQLNSEETKVERDLLEPNFLQPDSLSAQFQARAIYVI
NETDDDRFSPTYWLLQTSADDSDQEVELVSCDLESMAERFCPGLDLKTELGKESITKDSLLPLKCKKKGSNSQGTTL
GKRKYQPLINPGQKERSGKKPFIAPMRGRGFSRSGIHPSRANDPFRSRPPNTSRPPSMHVDDFVALETFHPNSVPT
GPTNKRPSKETGRGRGRGNFEGNRAFGNARGRFLQSQQGGSYNSRRGDDKSGRGSNEWINRNVSRGNSGNVS
QWNARNNQLLSGKNTAKVNANLQRNFPRGGGHLEPRTDVRTTPGNARLQETYGRFQRTRGGNNSGNWSGSRS
KERTDGRFQAKMVRNLIYVYILSVVSSLTLVHSTDPLYTISAPNTFRPNSPYQLIITNH----QAPG--PIDVVVELKANE-

-------------NNETTPLVGQAKIEVGETKRIDL--------QVGSWSSGMYTLTVNG------SG---GIEFSKTTT---LSYSS-KT-
FSAFIQTDKAVYKPGQ-LVHFRIITTDI-ILK--PVDLK------------ESSIYIV-----------DSQGNRVKQ----WSNVTFEK-
G------VFEESL-------QL--
-----------YT-YGKPV-KGKVLLNVTDYYCKWPCSPYNMKPFSTTTS---
--IDGTADIQVN--LVRDLA--LPDWM-------
RHDRRFDVFAVVTEELTGRKQNG-SGDITMYDSQYKLSFSE----SGNFKPGLKYTITLQVSL-QDGSPIL-----
DDGEISINYFSSWNSPG---------------------KTLNYTIPKN----------------------------------GEIDVEIIPPE-----------
-------------------------------------STENLRFET----SYK-----------------GARTTSYVSKAQSL-SE-RY-----------LQI----
----TLLSQKPKI-------GDEVEVLVDSTKELNDN---------------------LIVQVIGR-------GKILHHESIPPSKAKS---------
--------------------------------------QTLKFKITSEMAPKIRVIVYYA-T----SCGE-VVADALDFSV-EGIFKT----K----
VNIH----------ANPNSTKPGSPVDISVQTEPQ-S--YVALLAVDQSV---------------LLLKS--GNDITQR----------------
-----------------E-----ILDDL-QSYEIGRQSYYSYRDGNYMIRPWR------------------------PPSPS------TMQLF---------
SNVGLVFFTNG--------LFAHIPSYGGYGGYGGYGGGGMYDGELYEMDAMPRPAVHFAPQAFASGNRGPPAAPP-
----

PRPPPPPPQSRPISDTLMEPTRVRRHFPETFLWTNAT-A-----GADGM-ISITA--NAPDTITSFFITAFAMNENTG-LGL-
SKSPEKLQVFR--------------------------------PFFVALNLPYSIVRGEAVALQALVFNY-LKE--DVDAEITL--------------
------ENGQNALDFVELENTVDEDK------SQKKLIKTIR--------AKAGEGTSVSFLVA-----A-KKL--E-----
YIDINVVAKSK-------------------VAADAIVRKLLVKAEGKK--MYSNKAFLVDLRNQSSFE-----AKV-------------------
-SIDIPAG---AVSES-E-KIEVSAI--SDVMGS-TIN---N------
IDQL-LRMPYGCGEQNMLNFVPNIVITEYLTKTKQLN----DEI--KNKALKFMESGYQRELTYK-R--TDNSFSAFG-----
NSDK---NGSVWLTAFVVKSFVQAK--------NY--I-TIDDTVVSSSLTWLA-QQQAK-NGSFSEVGEVFHKAMQGG---
--SGK------------GLSLTAYVLSAFLESKGIELLPGTS---S-VEEVVKSS--LEYLEKELHN-LK---SDYDLVITTYVLHLA----
DSKKKDEAFEKMNKVAK--------------------TEKDVKFWSVP-----------LPVENSSVPYYNRP----
ASVDVEMTAYAML-TYVQRGL--IPEAIPIMRWLISK--
------RNSNGGFESTQ-----DTVMGIQALA---KFAASL-TPP-----AGSKLDISVSYDS-----NKTDFAIT--
KETALILHREQL-PRTTR------DLTISAS-GNGVGVVQVSWSYNVL---TTED---RPAFSIHVN---------------------------
--ATGENEELIIN-SCAKYI----
YKVNGESNMAVMEIEFPSGYVADLDHLPSIN--

EEKKIKRVETKNGD-----TSIVVYFDKI--GKEVCTSARGHRAFKVAKI-KPALVSVYDYYDPIKRGEQFYNA-PATS-
VCEL-CET------PECKKKQC---GTE------------

>Strigamia_3 SMAR006272-PA pep:novel scaffold:Smar1:JH431683:43422:50346:-1
gene:SMAR006272 transcript:SMAR006272-RA description:""

---------------------------MSINKLFAF--------YLTICL---

--------------------------CIG---
-----------------------------------YVAANGYYTIVAPKVLRPALDYHVSVSVH----NVLQ--PVRMAITLSGRS----------
---DAGDLVSSEQHVFLQSEQNQVIGFTVISEGKSRIGMWGTGNYSMKVTG------SG---GINFFNETE---LTYEH-
KN-YMVFIQTDKAIYKPGQ-PVLIRVIVVSP-SLR--PTGTE-------------RLDIFVT------------DGDGNRVKQ----
WNRVFTQR-G------VFTTEM-------PLSDEPVLGDWNITVNILVINKLKFYAKIEEIDQKYQKS---------------------
FTVAEYVLPS---------------FDVRIDL-PN--YA-TFT---ESDIVATITAKYT-YGKPV-
KGKAVIMVTPLVRSPQIRTYYEDPLRKTVE--
---------------------IDGKVDVPFN--LQQELN--IKD---------DYHRMVRFEVIVTESVTERRENA-
TGVIAMFKYKEKIELIKH---SETFKPGLKFSAFVKVAD-NDDIPVN-------DSTNPLIIRYGYGHDDSLY------------------
QTKQFKIPTN--------------------------------GSIELEIYPPLAD--
NIQRLIIIA----KYKS-----------------IEQYFPPIRRAESP-SN-TY------------IQA--------
VLMTEKAKVVFKWFAGGEEVLIHVNSTAPMPSFNVVAGLGALLKKIAKCCDSVALQIYQIVLGR-------
GDIVFADKVEANGQKT---IAVKFVATNNMSPRTRFIIYYT-T----SSGE-
VVADGLSFEV-EGVFRNFVIKLFQKIVDLK----------ANKHGSQPGDTINFQVTTQPN-S--FVGLLAVDQSV-----------
-----LLLKS--GNDVTQE----------------------------------E-----IIHQL-ELFDTGKQPKSHLDLIYSSIWF------------------
--------PGSAT------ASEVF---------KDAGVMVMSNA-------LVYEEYNFIMPRGG------------------------
GELRPGVEKGKPVGDYYIP---
KEAEVGFRDPLDGSIIPIMRQHFPETWIWSNAT-A-----GQDGR-AVFTR--EAPDTITSWVISAFSLDMFTG-LAV-
SPSPLRVTIFR--------------------------------PFFISLNLPYAVIRNEAIAIQAVIFNY-MKETIEVCATVTL----------------
-----ENTNQFDFVTVEDVVNE--------VEVVNSKTKK--------VESGTPATVYFMIV-----P-KEL--G-----
YIDVKVTARSSTRSQ---------------VVSDSLKRKLLVKPEGVP--QFVNKAYLIDLRSSSLFN-----ASV-------------------
-NVSIPKT---AVSGS-E-RVEISTI---ADIMGP-TVD---N------
FDNL-LQLPFGCGEQNMIRFVPNIVVIDYLSSIQHLT----PIV--KSVALTNMETGYQRQLTYK-R--EDGSFSAFG-----
NSDQ---SGSTWLTAFVMRAFSQAK--------YF--I-AIDEQVVNGSLYWLI-AQQLE-NGSFPEVGVVSNKAIQGG-----
SGK-------------GLALTAYVLLAFVENK-A-----ER---I-FSSQMTKA--LRLLEDQIES-IE---DSYSLAIVSYTLHVI----
NSGKKDAAFRQLQSKS-----------------IS---A-GEFRYWKKN-----------ASAETAET---IKLA----TPIDIEMTAYALM-
SYVL-----------RND--LSGSILIMKWLITQ--------
RNVNGGFQSTQ-----DTVVGIQALT---MLAKRI-VDS-----QI-YIDVMFQYDN-----EQKNVHLD--
KDNSMILMKEEI-PSTVK------MVNITAT-GRGFAIVQVSYSYNIM---VSKE---NPSFQVNPF---VD-------------------
--RSSTKDRLQLN-VCAAYA---E-NGAT-
SNMAVMEVTLPSGFVIDRDSLPALH---

--RVDEVKRVDIKDRD-----
TTVVVYFDKL-DNKLVCPTIKAYRTYRVAKQ-KATAVYVYDYYDQAKAARYFFQA-PQAT-LCDL-CEG------
DECNSAKC--RK----------------E-----------
TEERQAAENENSGSLKMADA---

>Strigamia_4 SMAR005770-PA pep:novel scaffold:Smar1:JH431640:61740:71194:1 gene:SMAR005770 transcript:SMAR005770-RA description:""

---------------------------MERPRTSIAIYL--------LVIYSF--

-----------------------------VGV---
--KALNYVVSAPNVFRLNVPETVGISLS----NHPLNQPVTVEVLIKSYP------------
--HKTNIMARRLVVESGKPQITTLTF-PESAISEIQKTDEVLFVYLVVTA-----------PLGIFPEKE---
IILPVRKYSGYAFLQTDKPLYTPKD-IVKIRILNLNE-KLI--PNDKP--------------VLLEIK------------NPNASRVLY-----
EKFNPDDKG------IIEYVF-------KFPIFPVHGLWSASIKYG---Y----------QLTGIKT---------------------
VHFEVKEYELPT---------------FSVKIEP-PK--VI-LKN---QKLIETRITANYV-YGKPV-
VGLLNIKFGIKTVEGIVVEIGSLYLQPL---
-------------------KEGERWISIN--VERDIK--QKNIVWFPE---
VDGSNLLIEAEVIEKATGNRETAIDYSTIFSYSPFVVSFPRS---SLDFKPGVPYQVQADVKF-VNGKEAP-----K-
VSVSIISAVSKKGGQVFRHLP-----------------RPLASLTNEL------------------------------------GRVMFELGTGI----------
--EDQELEITV----TTEEEGLSVEN----------QATNVLNVIKFKSP-DTTSY------------
VWI------AAPPEEMIFTV-------GRTFQTQVTVFPAA-GDIK---------------------LHYMVVSR-------
GMLLLQGESASERNAV--VHPIQFSVTADMSPSARVIVYII------KNNQ-
IIADSMKIDV-HQTCKY----NNGDDLKLS---------ATATGFYKPGQNVKIEITGERD-S--FVGLLAVDEAV---------------
-YLLNK--EGLLTRK-------------------------------------T-----MFDEM-ERHDLGCGP---
GGGQD------TEAVF---------RKTGFLILSNA-------AINEHKRIDGECDA----------------

NHKRNKRSLSTLPKPVKVEHFYTGLAAV--CCKLGRLTGP--DGMDCTQRAAVIYAA-----
INDTENCSQAFLDCCETEV----
TYKASYMAVARSAMSNKANDANLEDSELIDEFEVYVLQNLRHLFPETWIFHTEKITNKECSKNNKNSCKLTYSTNFP
HSITTWRVQALAVSQTNGSLCI-A-EPIRLTVFK----------------------------------
SVFIQMQLPYSAVRMEQILVPVTIFNY-GDE--ELPVTVYM---------------------YGVEGICMGAGA---------------
GERSESQKIT--------IPKNSATTVTFPIM-----P-LEV--A-----SYPLRVVALSW--------------------
SESDAVEKILRVVPEGIP--VQKPLSIMLDPSGTIRQRKKRNAIDG-----IEETFDEEHNRQSLVIDMPLPKE---YIPGT-
E-KCLVSTM--GDFLGQAVVT------
SIQGLEARFFHLPTGCGEQTMIKLAPLVYSVLYLKRTGRLT----AEG--EKNGYSLMQQGYSFMLNYR-K--
ADGSFAVYQHV----------ASSTWLTALVAKVLCQAS--------LF--I-DIPKEVTCNALDWTL-RKQRD-
TGAFFADFGVYHTEMIGG-----VQS-------------EATLTAYVLISIMECSKC----------DSADKRIAALRAISYLEHHVGF-
LN---HPYSLAVVTYALTLA----KSPKASSANAKLKALAI-------------------FNEAGDTRFWDVN--------------
HAEFQGNKPWIYVNRPNALAVETTSYALL-TQLLFDD---
--IAYSHPIINWLNQQ--------RNDQGAFASTQ-----DTIMALQAMA---EYSYRA---------KLPALNMVCNVSS---
QTSRRQRSLIMTNENSLVLQKLEL-PAGG-------KIHVDVE-GKGIATMSLSLHYNIE-----
SSKKDQCKFDLKIQTTEIEDIIMPQMPKPEFDGFDLLPEMVVRTITSSEDQKRKFGYKVVVQKEQEDDEYEYEYDNE
ND------------------LAHESRHIVKLEICVRHL------GDKPAGMSILDIGIFTGYKPVKDDLVKLL---------------------------
----

------------------------------KDATISQYEPSD-----TSVVLYIDTVPHDEDLCIKLRTMQEISVGKV-
QPTTVKIYDYYEPDKSCQKFYTPDGKSSMLSPI-CEG------RQCRCVEG--
--------------------------------QCPPCFPFSDLKKEK-----------------
NSQEQRNKLMRLVCDKHNFVWNGTLIMKSQSDGFVYYDFLVTEILKEGLQTKE--------------------
EVIDEIITFTASSACACPAMEVNKVYIIMGRDGFRY----KDEHSNWKYKYTFDQKTSVHEMHLLRNIENEK------------
--------EREMQKTFLWLYRRMIERGLGCDQ---

>Strigamia_5 SMAR002371-PA pep:novel scaffold:Smar1:JH431114:22302:29439:-1
gene:SMAR002371 transcript:SMAR002371-RA description:""

----------------------------------MVAIF--------AWLALM---

-----------------------------PVVLCQQLPPQNIPMPPQAGPPFPFGQGVYGPNMGPNFFGMPI-----------------------
--WLRRE-----------------DP-----------THMILASKVVRPGSVYRVIVHIL----
RSER--PVGVRASVSRD---------------GVEVGSANK-MLKVGIAQELLIKV--------PPTS-VHGRYKLRVEG----
NANGVTGGTVFANETN---LEFSS-RF-LTVLIQTNRPVYNRLQ-IVRFRIILITT-DLK--PYTDP--------------VEVSIL-----
-------DPNGFVMRR----WFSAYSNV-G------TVTLHF-------TLPSDPVQGYWTILVTAQ---N----------QEEKKL-------
-----------------ILVEKYWPAR---------------FEVNVNL-PT--FL-LDS---DRFIRGSIQANYTYMYSPV-
MGNATVQLSVQP--------NRYTYNVK----------------PEK---TVVMEEYVPW---
------------------------------FNGTYDFFFS--VDRLLH--SVP--Q------LDGTLVKVEAFVFDKFMEMQNSG-
FAMASITNSSISLRFLGSS--AKVFKPGMPFETLVAVSY-HDLVPLPLRKIGN-SRLEVVTTATMRSGSRVQL------------
-------APYNERIYDS------------------------------------GVFKIKF----------------------TPPF-----------------------------
DATMLAIRA----IYSDI------E-GD------RVTAETVAVTYFSP-DR-RY------------MHV--------YTSTNNPKI-------
GEYIIFHVRTNFYV-KS-----------------------FHYLILAK-------DIIVTSGEEIMETVTQ--------------------------------------
----------KIKTFAVPVSGEMAPSCRIVVYHV-A----KDRE-IISDSLTVPI-DAVSRY----E----IKME----------
LNPYKDKTGETIELRVSGDSG-A--FFGVSVMDAET----------------FKLQG--GNELSPA----------------------------------
--L-----VSEMM-HTFD-NDYKRLN--KMLWTS----RE---------G-EPDKRAYYQTT-NYGVD------NNRTF---------
NMAGLVVFTDA-------NI-TQLP---D--LCFKELGLFSCM-----
DG-NCYRSDQK--CNGKWE----CPTGFDEADCGVD-------IAAINLKDFRLTRKNHMDRFYDPDYGDWAWISFN-L-
----APEGRERFPIG--PVPRRPAKWILNAISMSREHG-LAI-ISQPYQFEGSR---------------------------------
PFFITVEMPSICTRGEQIGIRVQVFNL-QPF--ETYVMLEL--------------------PSSPHYRFVHV---
ESEGMVKSYNPRTSSGDHQHLIW--------VHAHKSVTVHFPIV-----P-THI--G-----SFNVSIEAFTH--------------------
VGRDRVMKQLTVEPDGMQ--QILHTSMILDLKNQPLVIKFLDVNVT------------------ETPIVPYQRW-LRYNFES-P-
RAYVSLV---GDVVGPAFLK------TPVNTQVL-
LRRPDRSAEMQTFNFAANLWTLHYLRLTNQLQ----MPK--AKEVFKHLNVYYAWIMAYM-K--PDGSFVM----
WRGFE------PSVWLTQFVVRTFQQAR--FPDWE-FY--L-YIDPNIIKQSVSWLL-GHQTYE-GSFYETTLYP-
LDRKMDPRIK-RFK----DVSD-YHLGNVSLTAQVLITLCEVND-LPGQLRM---Q-VANARLKA--AQYLERVLTS-LE---
DPYEVAITTYALTRA----SSVDAELGFNILDGMKR---------------------EQEGMVYWSREEAP------
LVGIITENQRPYIQPRLPQKFDAMNVEATSYALL-VYVLKGG-I---
---------GMIQEEIVRWLNTM--------RMSDGGFISTQ-----DTIIAMQALT---EYTFRE-RLR-----DITDMDVSLESSA--
--SPGLSRVLHIDVNNLAQLQRMEI-PNVWG------HVQVFGR-GSGLAILQMDVQYSID-YVPLLIKPPVKAFELTVQ-
--TQYSG-RNASTLHIYSCQRWT---
YVEESPVSGAAVLELEIPTGYYQYQPILEKIV---

--ENENRGLRKNIRRIKITE-
----RKL-----HL-DTRFTCLNLNVQRWFPVANLTQYLRARIYDYYAPERFNETDFKHFNLYVLSICQVCGS--------------

----
YQCPYCPHFSFG---HVAHIS-YLIFIVCLMFSVLRLI--

>Strigamia_6 SMAR006274-PA pep:novel scaffold:Smar1:JH431683:59546:66419:1 gene:SMAR006274 transcript:SMAR006274-RA description:""

--------------------------------TYFIVVSEVLRPGHIYRVAVHLL----DAPL--PVTVKVSIHRD--------------
GAEVLQTSR-ICQAYVPELLKIKV--------PISL-IRDQYKLRVEG----NIKGVFGGLAFFNETF---LHFSQ-QS-
ITILIQTDRPVYKQGQ-TV----------------------------------DVYLL------------DAKWSIVKR----WMSRQNNQ-G------
PISLSY-------ELPQQLNTGQWTIRVVAY---G----------QVEEKQ-----------------------FLVKEYYKPR--------------
FTVAVKM-AS--FFSINV---DNFIHGCIEANYT-SGAAV-TGNVSLHIAVHP--------PRIGYSNI----------------KSD------
---------------------------------------PFIEQFIPE---------------------------------FSGVHCFKIG--MWSLAN--LAS--K----
--LDGMQVTVAATVGDKVTLRNIEG-FSCTIVTNGDVKLNFLGSK--PQVFKAAAPFKTYVVASY-
NDNSPLPSYRLKR-HRFELYITVVYQNGSKQKL------------------DTRLIKMLNK---------------------------------
YIGVWPVEIDLRSEI------------SIDQTKLK----------------------------DIKYLQLEA----TYRDN---------------------------
--YEKPILSK-------GLILTRGTETI---IS------
---SIRTFAVTIQPNMAPSSTIIIYYI-G----SGGE-VIADSITFPV-NSFIDN----E------
VS----------LTSRKTKITNNIEITVKGKAG-S--SISLSALPREL----------------YLMQA--GNEISRH-------------------------
----------L-----VLEQM-SSFN-DNKNKTLIHKHTWLS----RE---------G-FPNRPVQFPSY-SYAPD------ANRTF---------
EFLSLLVFTDA-------NL-TAHE--D--NCDKMQEMKACL-----
TQ-GCYNKDKE--CDGTYD----CADWSDERNCAVK--------EDYSLQNWRIHRQSHTEKTFD---DSWLWNDVI-I-----
GFKGFTSFNV---TVPDKSEQWMISAFSMSKNRG-LRL-LDNPIQYDSQS---------------------------------
PFYLNVEMPSICKLGEQLAIRATVFNN-KNT--EIEVAVIL--------------------ASSKHYKFFSV--
INDTNGHGEKRGSTENSEHQHIIM--------IKPKDSVITHIPIK-----M-VKI--G-----KINVTVMAQTD-------------------
HEKYTVTKVLKVEADGIP--QYRHTSTLLDLNNRAYLISYFDVNLT------------------DTPVIPYRKE-RYYVYSS-N-
IAHFSVF---GDVVGPALMP------LPVNSSVL-
LRQHDDSAEQNLFNFAINLYMMKYLRDINQRN----LAV--EKKMFHYMNLVYAQQLSYR-T--EDGAFKPFR--WK-
SN------TSVWLTAFCARIFQEAN--SKDWE-NF--L-YIDENVVTDAIAWIL-KFQTPY-GAFFESS--V-YGDRFNQTCC-
FHLNEDMDPPR-YR--NVSLTAHVVLALHSVRE-MRGELGI---N-AAKAKNLA--INYLERMLRT-
IQDNKDPYDLALLAYTLTVV----KSQVADEALNILVGKGR---------------------EIEGMMYWGIEEIP------

PPKYNIASNKPYLQSRLPARYDSRNIESTAYTLM-VFVAHQA-L--
-----------MQEPMVQWLNFQ--------RLTTGGWGSAQ-----NTLVAYQALL---EYSSRL-PAR-----DTSDILLSVDSSS--
--STGFEKIFNVNGNNLLKLQTVKI-PNAWG------TVRLVVK-GTGLAVAQLSVQYNVD-
VPEFQTQPPVPAFSVVVD--ATYEG-
YNSSHLRLQSCQSWL---PTNESEISGMAVLELEIPTGYVVDQENLISYV--

-LS---GKVNKLTEARFYE-----RKIIFYFKHL-
DSTNTCVNIVAERLYPVANLTSIMGVKVYDLFAPERFNELMYETRTLQSLTICDVCGS----------------------------------
--FECPNCFVEVSH---
ANPFPNCRFLVLASIMLALFYA---

>Strigamia_7 SMAR008374-PA pep:novel scaffold:Smar1:JH431836:34693:46592:-1
gene:SMAR008374 transcript:SMAR008374-RA description:""

-----------------------------MYCAILMYK--------WFLLIW--

-------------------------------FHLHLTNGRANIDMHP--
--SHIIVASKTIRPGTIYQIVVNIL----HMEK--PIHVRASISRE-------
--------RVQIVQAKR-LMKKGESNKLLLKI--------PQTT-IPGDYQLNVYG----SMTGGTGGNLFQSSKE---LSFSP-KF-
LTILIQSNRAVYNADQ-TIRFRTVIMTT-EMK--PYADP-------------IDINIL-----------NPKGYVMKN----
YGSVYLNV-G------VGSFTY-------QLPLDPNRGWWTIQVVAQ---T----------QVEEKR-----------------------
IYVEKYYMQR---------------TEVYIQT-PD--TV-MDT---DEAITGTMFGWYL-NNAPV-LGNATIRVYVTP--------
PQQFYKQA----------------ESL---HALQDYVEQ-----------------------------------
FNGNYELNIK--LADLEN--LVS--P------LAGSVVTIEGSLTDQRLELFCVG-YSKTRIIDGNVRMKFLGSH--
PLFFKSGMPFKTYLVISY-ADEAPLSESKLQD-STLHISRLVTHANGGSTRL------------------VEIHDKFPND-----------

----------------------GLYTLQF--------------------VVPE--------------------------NTIRMSIKA----VYKDN--------
EE------MTTADLLLFPYYSL-DT-RS-----------LFV--------YTSTKNALV-------GEYLIIHVRSNFYM-KH----------------
------FFYLVVSK-------GIIIHSGVETVDALRM--
PIKTIAIPVSSEMPPTCNVVVYHV-T----RDGV-VISDSMRVPI-NPINKA----K----FTFE---------
LNNRKDKTGNTLEYCILTEPG-D--QIAVSAHDFDT---------------SCVNC--GQEITKA--------------------------------
-S-----VLESL-HSFDADHWRTSKILRASRDH----------------KSDQLFFYDSS-GFGGN-----DEDTF---------
TQTGLEIITDA-------II-ASSS--M--LCGNDSSYISCQ-----DG-
SCFLATKV--CDGEID----CRNGFDESNCSSP------ISEDFNIYEFRISRFSHFDQFYDMADGDWAWFDYN--------
SNGFDAFNQ---RVPNRGSKWIIYAFHSNKDTG-FSI-LQDSLLYDGTK-------------------------------
QFCISVEMPTLCRRGEQIGIRVELFNF-GET--ELFVTVVL-------------------MASDSYQFVHV---
EEKGQVRSYDARTSSGEHQHLVW--

--VLIYGHTIHQIN---

>Strigamia_8 SMAR006270-PA pep:novel scaffold:Smar1:JH431683:16032:34051:1
gene:SMAR006270 transcript:SMAR006270-RA description:""

MAVGTMELLFFDTFSHESNEELNLDLVQFPDPVYINEVRIIPLGTRVQADFPGGVRLGATNPSQFELEFFVNDLSKT
GASTFEGLGSLEYKQNVDIQLTFKKKIPTDGLVLRGWYTTITIAIYGSISKIKERTSPPPPPPPQATQKIRSTEKEAIDAT
PHTDWDRDSPSETDSIIQSTAEQVAFADPHTPNQEHGHYPADHPKPTYPERSTYPEHKSYDYDFPSGRSWSSTVTE
HQTSNTNKLDNFPKERNVYGRSSYSRDNYEEKKPNWDHKDRDRDSRDPRTQQREKERTRDGREYREIDRVEFPLV
QEPENEPAWEKLAESVEKDSPTERTRYPEREEELSPEVAAEREFRERTIKRDAGKGEPLRPDSKRPRTPPLSSSPKRR
PRTPPLPSEGDQHRARGPCSPPPPKDEMEPGEVADDKNLFDPLTPDDSPDLTVDNLSDGEIHEGETNDGGYSEFIY
DEILSDEEFMSDGEERNLDLPELYYEDSWNYISSFNPYQCELGSLLYFSDPSLTHYEIEKKKMGCPPDEIAMETAQKLL
KTLEYFDDKEHSEKWVEAMELLPSMLVPGLAILKQSAELKGLVDVLLDWLCEGLNFEKAIEQSQAVYKVRHIKAGIR
LAIALSNCDYEMTHQLLEKNVQKKLLDLYGKEYMALSIKLLIVQALDASTRTEEGMKYFLGLSENQFAGPEDKSDYQ
RLLNFVLGKQSSRAAVAVSALLKKIHVFEIISKFCASVEKIIDNSAVFEISPNKPESAKDCDNELSNAISADNDIDNFSIN
YLNDDEIEDIISYLIEINKMLGHAQQFIQDDGRDEASEETPHQLGLQLVYHLQVLQYVDMLLDFHSKDSTKKELDDP

EALTVLQPLYTMVFLPIGRQVVVDVLSIEHNLNALVPFLELTGDEQNEIKIRKSASMGYATELLLVITHYSENVSLLKN
FGEKLLNICTQESSSKLQEIVSWFSVMKRIPSYSADAAAPLCTIIKNLIEEVLTFPGELITSVRILKDLCIPPSTNLKPEEQ
REELKYRYAIIQFIANDGLSNFITILQKICDAYQYPYPQNYAVIGPQWGLLLALIKPIVSLMKHSLCYLIYCRNAEYKDL
MAVPIILRVYSFLNILPITSSFYPVAQGIQSEIIEMVLAYTQPIMTISDSDEALNKSLWSQMLQEVLKFATTLPSNFLSG
VTILSELLPLPLPIQTRESLTNEDILQATNARKLWSAHLHSMSNEIKDIIKKLLGTSLPLLQQQLKRISVQLADLGASCSL
VVTRAVFDIVLENLEDNEPEDLENKECFSNSHTYRLLNMLDCLANQPCIKIALIQLLRANTKLDEKYAQLLPKLTSLLN
TASDKTVHLQIQECILSIFQSLCDYEIILGSENSLSPLDQLGNALPAKDQMVAICLALLDHVSNSSHSFSTTLLAFRNLI
MILETDLGFYCLKLNFDKKPEILHSILNRCSTSFNKDNVDSVAALNICLDFCRLLYTDDSAQGVPRTLFFTISELGFYIE
WKVNEAKEEIPIHPLIKIEKILLEFVKDDDALDVQSAENVSSLILQLNSEETKVERDLLEPNFLQPDSLSAQFQARAIYVI
NETDDDRFSPTYWLLQTSADDSDQEVELVSCDLESMAERFCPGLDLKTELGKESITKDSLLPLKCKKKGSNSQGTTL
GKRKYQPLINPGQKERSGKKPFIAPMRGRGFSRSGIHPSRANDPFRSRPPNTSRPPSMHVDDFVALETFHPNSVPT
GPTNKRPSKETGRGRGRGNFEGNRAFGNARGRFLQSQQGGSYNSRRGDDKSGRGSNEWINRNVSRGNSGNVS
QWNARNNQLLSGKNTAKVNANLQRNFPRGGGHLEPRTDVRTTPGNARLQETYGRFQRTRGGNNSGNWSGSRS
KERTDGRFQAKMVRNLIYVYILSVVSSLTLVHSTDPLYTISAPNTFRPNSPYQLIITNH----QAPG--PIDVVVELKANE-
--------------NNETTPLVGQAKIEVGETKRIDL--------QVGSWSSGMYTLTVNG------SG---GIEFSKTTT---LSYSS-KT-
FSAFIQTDKAVYKPGQ-LVHFRIITTDI-ILK--PVDLK------------ESSIYIV-----------DSQGNRVKQ---WSNVTFEK-
G------VFEESL-------QL--
------------YT-YGKPV-KGKVLLNVTDYYCKWPCSPYNMKPFSTTTS---
--IDGTADIQVN--LVRDLA--LPDWM-------
RHDRRFDVFAVVTEELTGRKQNG-SGDITMYDSQYKLSFSE----SGNFKPGLKYTITLQVSL-QDGSPIL-----
DDGEISINYFSSWNSPG----------------------KTLNYTIPKN-----------------------------------GEIDVEIIPPE-----------
--------------------------------------STENLRFET----SYK-----------------GARTTSYVSKAQSL-SE-RY-----------LQI----
----TLLSQKPKI-------GDEVEVLVDSTKELNDN----------------------LIVQVIGR-------GKILHHESIPPSKAKS----------
---------------------------------------QTLKFKITSEMAPKIRVIVYYA-T----SCGE-VVADALDFSV-EGIFKT----K----
VNIH----------ANPNSTKPGSPVDISVQTEPQ-S--YVALLAVDQSV----------------LLLKS--GNDITQR------------------
------------------E-----ILDDL-QSYEIGRQSYYSYRDGNYMIRPWR------------------------PPSPS------TMQLF---------
SNVGLVFFTNG--------LFAHIPSYGGYGGYGGYGGGGMYDGELYEMDAMPRPAVHFAPQAFASGNRGPPAAPP-
----
PRPPPPPPQSRPISDTLMEPTRVRRHFPETFLWTNAT-A-----GADGM-ISITA--NAPDTITSFFITAFAMNENTG-LGL-
SKSPEKLQVFR---------------------------------PFFVALNLPYSIVRGEAVALQALVFNY-LKE--DVDAEITL---------------
------ENGQNALDFVELENTVDEDK------SQKKLIKTIR--------AKAGEGTSVSFLVA-----A-KKL--E-----
YIDINVVAKSK--------------------VAADAIVRKLLVKAEGKK--MYSNKAFLVDLRNQSSFE-----AKV-------------------
-SIDIPAG---AVSES-E-KIEVSAI--SDVMGS-TIN---N------
IDQL-LRMPYGCGEQNMLNFVPNIVITEYLTKTKQLN----DEI--KNKALKFMESGYQRELTYK-R--TDNSFSAFG-----
NSDK---NGSVWLTAFVVKSFVQAK--------NY--I-TIDDTVVSSSLTWLA-QQQAK-NGSFSEVGEVFHKAMQGG---
--SGK-------------GLSLTAYVLSAFLESKGIELLPGTS---S-VEEVVKSS--LEYLEKELHN-LK---SDYDLVITTYVLHLA----
DSKKKDEAFEKMNKVAK---------------------TEKDVKFWSVP------------LPVENSSVPYYNRP----
ASVDVEMTAYAML-TYVQRGL--IPEAIPIMRWLISK--
------RNSNGGFESTQ-----DTVMGIQALA---KFAASL-TPP-----AGSKLDISVSYDS-----NKTDFAIT--
KETALILHREQL-PRTTR------DLTISAS-GNGVGVVQVSWSYNVL---TTED---RPAFSIHVN----------------------------
--ATGENEELIIN-SCAKYI----
YKVNGESNMAVMEIEFPSGYVADLDHLPSIN--

EEKKIKRVETKNGD-----TSIVVYFDKI--GKEVCTSARGHRAFKVAKI-KPALVSVYDYYDPIKRGEQFYNA-PATS-
VCEL-CET------PECKKKQC--GTE---------------

>Strigamia_9 SMAR006272-PA pep:novel scaffold:Smar1:JH431683:43422:50346:-1 gene:SMAR006272 transcript:SMAR006272-RA description:""

------------------------------MSINKLFAF--------YLTICL--

----------------------------CIG---
-------------------------------------YVAANGYYTIVAPKVLRPALDYHVSVSVH----NVLQ--PVRMAITLSGRS-----------
---DAGDLVSSEQHVFLQSEQNQVIGFTVISEGKSRIGMWGTGNYSMKVTG------SG---GINFFNETE---LTYEH-
KN-YMVFIQTDKAIYKPGQ-PVLIRVIVVSP-SLR--PTGTE------------RLDIFVT-----------DGDGNRVKQ----
WNRVFTQR-G------VFTTEM-------PLSDEPVLGDWNITVNILVINKLKFYAKIEEIDQKYQKS----------------------
FTVAEYVLPS---------------FDVRIDL-PN--YA-TFT---ESDIVATITAKYT-YGKPV-
KGKAVIMVTPLVRSPQIRTYYEDPLRKTVE---
----------------------IDGKVDVPFN--LQQELN--IKD---------DYHRMVRFEVIVTESVTERRENA-
TGVIAMFKYKEKIELIKH---SETFKPGLKFSAFVKVAD-NDDIPVN-------DSTNPLIIRYGYGHDDSLY------------------
QTKQFKIPTN-----------------------------------GSIELEIYPPLAD--
NIQRLIIIA----KYKS-----------------IEQYFPPIRRAESP-SN-TY-----------IQA--------
VLMTEKAKVVFKWFAGGEEVLIHVNSTAPMPSFNVVAGLGALLKKIAKCCDSVALQIYQIVLGR-------
GDIVFADKVEANGQKT---IAVKFVATNNMSPRTRFIIYYT-T----SSGE-
VVADGLSFEV-EGVFRNFVIKLFQKIVDLK----------ANKHGSQPGDTINFQVTTQPN-S--FVGLLAVDQSV-----------
-----LLLKS--GNDVTQE-----------------------------------E-----IIHQL-ELFDTGKQPKSHLDLIYSSIWF------------------
--------PGSAT------ASEVF---------KDAGVMVMSNA-------LVYEEYNFIMPRGG------------------------
GELRPGVEKGKPVGDYYIP--
KEAEVGFRDPLDGSIIPIMRQHFPETWIWSNAT-A-----GQDGR-AVFTR--EAPDTITSWVISAFSLDMFTG-LAV-

SPSPLRVTIFR--PFFISLNLPYAVIRNEAIAIQAVIFNY-MKETIEVCATVTL---------------------
-----ENTNQFDFVTVEDVVNE--------VEVVNSKTKK--------VESGTPATVYFMIV-----P-KEL--G-----
YIDVKVTARSSTRSQ--------------------VVSDSLKRKLLVKPEGVP--QFVNKAYLIDLRSSSLFN-----ASV-------------------------
-NVSIPKT---AVSGS-E-RVEISTI---ADIMGP-TVD---N------
FDNL-LQLPFGCGEQNMIRFVPNIVVIDYLSSIQHLT----PIV--KSVALTNMETGYQRQLTYK-R--EDGSFSAFG-----
NSDQ---SGSTWLTAFVMRAFSQAK--------YF--I-AIDEQVVNGSLYWLI-AQQLE-NGSFPEVGVVSNKAIQGG-----
SGK----------------GLALTAYVLLAFVENK-A-----ER---I-FSSQMTKA--LRLLEDQIES-IE---DSYSLAIVSYTLHVI----
NSGKKDAAFRQLQSKS---------------------IS---A-GEFRYWKKN-----------ASAETAET---IKLA----TPIDIEMTAYALM-
SYVL-------------RND---LSGSILIMKWLITQ--------
RNVNGGFQSTQ-----DTVVGIQALT---MLAKRI-VDS-----QI-YIDVMFQYDN-----EQKNVHLD--
KDNSMILMKEEI-PSTVK------MVNITAT-GRGFAIVQVSYSYNIM---VSKE---NPSFQVNPF---VD-------------------------
--RSSTKDRLQLN-VCAAYA---E-NGAT-
SNMAVMEVTLPSGFVIDRDSLPALH---

---RVDEVKRVDIKDRD-----
TTVVVYFDKL-DNKLVCPTIKAYRTYRVAKQ-KATAVYVYDYYDQAKAARYFFQA-PQAT-LCDL-CEG------
DECNSAKC--RK--------------------E-----------
TEERQAAENENSGSLKMADA--

>Metaseiulus gi_PIPE_391330844_PIPE_ref_PIPE_XP_003739862.1_PIPE_ PREDICTED: ovostatin-like [Metaseiulus occidentalis]

-----------------------------------M--

------------------------VFNIKKDIGRCERDGGGILEIMMKSALIAGIVGLFV---
----

--CRA--
---STQSSANFSAFLLMPKTIYQGDRNFFVLELQ----NQKV--

DGSARISIVDQN--------NSTIQYYQRTFSIPKNEGPTFNLDISDLVL--------SGVDQGSIYSTVILEG----T-FG---
STVIKENNT---IKIQT-DD-PLLLIQTDKKIYKPGQ-KVFLRVMPLSA-DLR--PRNGGM----------I--SVSVN-----------
DPLNNVVAN----WADVNFTD-M------VMQFDF-------QLISEPALGAWFINVRYE------------------------------------
-----------ETSLPSPGPSNRHLLGVRVHFAEVRGHN-HSTPYL-LMD---AETATWKVCAKYT-FGKPV-NGDVKINTT--
--LIRYPW------------EQLRGIPENYVEGRL---
------TDGCYNLTLE--TSA-LN--LTNPSF------EYRQ-VRVIANVFEDGTKLQMNA-TNQLSRTSMPLRLAFQPDSS-
DKYFRPYMNTYGTLRVSR-PDDTPAV-----G-EQIELCLNAKSELYQYHYQRTDVRL------------MCKNYTSDSQ-----
-----------------------------GIVRYTLPPHK------P---SVVSFSVEA----
SSTRYPTKYYPESQHSVMIR-QPRSSTFYRSFFSP-SQ-QY-----------VQF-----EPLSNEKLPCDK-------
PVQLTAYYTFREQD-KNLL--------------------FRYVVMGR-------SRNLLTKEVKVTAPQE-----------------
VAAQSDQSEMLIESLPNTFSDMAASSDKKIGRLHIEIDPVSTQLPHLTVALFYI------NGTE-VVADSIRVPR-
KVCSPN----K----VDLE---------FSENVIQPGANVTMNLEASPN-S--QCGIQIVDRSV---------------RLLSG--
SDNNPFL-----------------------------------R-----YRDDL-PNREIGQWEHPQPDTYGY------------------------------
CQSSG------GQSDF---------VDSESIFLIFL-------VPYQRMYIASAAVA---------------------
LKVGQAPEAFEGAAFVAPSASR-------------IGNDF--
GGVTATATQSVVDVRDYFPELWLFDISA-T-----DGTGK-LSRAL--TAPHQVTTWNADAACIHKTDG-LGV-S-
QSKEIKTFQ---------------------------------AFFADVTLPYSVKRLEVLPIIVSAYNY-LPQ--CMPVSITI-------------------
DPIPGLE-------------------PRTPLKTEAC--------VCSDSS-PFQYELRVAVTNE-TTI--G-----DIDVTVTVMNS--
DNMAICKDKPDAKSV---PSRDKLTKPLKVVPEGFM--VEKIDSFLLCPQTGIGK------ANF--------------------
DLVAPEN---VVRGS-A-RALFSVS--GDIMGQ-STS---N-----
LDDL-IVLPTGCGEQTMAKFSSNLAILNYLNATGKLD----SST--EKRFRTNLNRGYQSELTFR-A--QDGSYSVWG-----
GSWG---QPSSFLTAMVYQGFKRAH--------NF--I-FIDDDGLSTTYNYLL-GTQDRVSGCFNKIGSIYSWALRSSE-SA-
SDT------------RGSYTAYVLAALHGSL-----------T-NKTRIEKA----LLCVKAQK-NL---NQHALALSAYTAALYK---
DNDLANKYLSDLKTFEN---------------NK----LPNQRFYHPSDSY--------------------------APQSIETSAYAIL-
AQLTLSGADA--IQEVQPIVRWLVKK-------
QNRNGGFSSSQ-----DTAIGLQALS---KFAELT-SKD-----QK-SMDNKIHAKG---QNFEQNYAIN--
DKNAIFWDTRIL-NTIPN------NLIVTSE-GSGCVVATSQLRYNVP---SVPG---ETGFLFNVT---VT---------------------
---KASTKRNVVLE-LCASYD----
KTGGQPSSMAIIELTPFSGYDVVKPSLEAIL--

---FRNEIKRYDADG----
-HPIILYFDEF-TASPRCVTLELEQAFAVENL-QPAIATVYDYYDKSNKKSVEYKRGDLPTEKDNE-----------------------
----

>Metaseiulus_2 gi_PIPE_391331531_PIPE_ref_PIPE_XP_003740198.1_PIPE_ PREDICTED: CD109 antigen [Metaseiulus occidentalis]

-------------------------------------MAISRAL--------L----F--

----------------------------V---
--------------------------------LLAASARCQRASVYTIVAPSVLRPNVIYNVAASVG----DVPN--PVDIKLSILGND---------
EKGQFNYISRSAQVAP-----RETELISF--------EIGDWNPGNYSLKAEG------TG---SIQFSNETS---LGFAH-KS-
YSVFIQTDKAVYKPGQ-KVHFRMIITDP-FLQ--PSVTG-------------AVNAWVT------------DGKGNRIHS----
WERQFTTR-G------VLGNEF-------ELSESPVLGDWALHCEV----S----------NQKFSKS----------------------
FQVAEYVLPT---------------FQVRVEL-PK--YA-TYN---ESDVVATVKAAYT-YGKPV-
KGLVTLTVTPRTRYQQIRPRPYEQFQT-----------------KVP---
---------------------------IDGEVNIPLT--ITRDLQ--LKTD--------FFQREIEFFALVEEELTSRKYNS-
TSYMPIFDKAVRIQLIKT---SDTFKPGLKYTLFLKVSY-QDDTPVQ-----D-PE-NQIILRYGYNYEEEQW-----------------
--TEQKHWIPQS-----------------------------------GIIRLELVPPNEP--
TTVVLGMHA----EYKG-----------------QIFYLDSILPAKSP-SN-SF------------IQA-------RLMSPENPRV-------
NDRLEFEVNSTYPL-DH----------------------ITYEIMGR-------GDIVSASTITLRNETT------------------------------------
-------------FRFVVVASFKLAPKARLVISSIA-----KNKE-IVTDSVNFDV-SGILRT----P----VDIK----------
ASVSQTKPGSRVEVTVSTTPS-S--YVGILGVDQSV----------------LLLRT--GNDLSEA------------------------------------
Q-----IVNEL-ESYDAGKKAKVWPTYYRRRKRRSLWY----------------------PGSYT------AGEQF---------
RDAGMIVLTNG-------FVFDQDE-VEGRDN-----------------------VIRLDTNTLTNQPLPPSGLP-----------------------
--EAPDVEPGRIRLRSRFPETWMWTNTT-T-----STDGR-
AVISE--IIPDTITSWIVTAFAMDQTTG-LGI-APTAAKVTTFR---------------------------------
PFFVTVSLPYAVKRDESIAIQCVVFNY-GNK--AIEADVFI-------------------D-NSKKDFIFTSAST-----------KPDR--
KKIT--------IQPQNGSPVSFLIT-----P-TRL--G-----YIDIKITASHG--------------------FGGDSVLKKLLVQPEGST--
QYFNKAILLERRNSQAAPSQ---YNV--------------------SVSFPKN---AVPGS-E-RVYLTAV-----------------------------
---------------------------------GDILGP-SVN---N------LKDL-LKMPHGCGEQNMLDLVPNIVLLDYLKASKRLT----
PQL--EAQAQKNIEHGYQKQLTYK-H--DDGSFSAFG-----NVDK---SGSSWLTAFVVKSLAHAR--------EY--I-
RVDELVLKNATEWLM-EQHLP-DGSFKEPGQVIYKPMQSG-----AGS-------------GAALAAYIVIALSEAR-V-----RN-
--E-YPDKIRQS--EEFLLRELRT-TA---DPYVTALITYALHIS----NNPNKDQAFFKLQSLAS----------------RE---D--
QLIYWKDP-----------------ST-DLFFKR----HFKDIEMTAYALL-SLIS----------RGD--------------------------------------
--------------------TGQAIPVMQWLISQ--------QNSNGGFTSTQ-----DTIIAIEALS---RIAATV-ASP-----TI-
SIDATIKFGQI---GGSRTLRID--SRNALVLQKLEL-PSDLK------WVEIEAT-GFGTAVVQVSWEYNLM---VTSE---
EPTFYLNPQ---LD---KTSTESYMQLS-VCTHYR---

G-EGNA-TNMAVMEVGLPSGYVFDYDTLSSIH---

RTNDVRRVESNDGD-----TNVVIYFDRV-TKNELCVTVPAHREYAVAHQ-KPAPVKIYDYYNLANSARIFYHP-KKAR-
ICDI-CVE------DECSD-SC---NDVEAT--------
FER-E-----------TADGDLAEPDASATLAAPSITAVAILAIMLLLLQNAR---

>Metaseiulus_3 gi_PIPE_391330201_PIPE_ref_PIPE_XP_003739552.1_PIPE_ PREDICTED: C3 and PZP-like alpha-2-macroglobulin domain-containing protein 8-like [Metaseiulus occidentalis]

----------------------MEIRRTILFTDLMEKC--------SNRLVP---

-----------------------------------MCEVSWKEVKHFDSSPDVR--
------------------------------DRFGT-----------------RESSRCKTHKIILRTYLVVASRYVRPGQIYRIYVQLF----
GTENDSPINIIAAIQRD---------------GVEVAHASE-QVIPGMDTDVLMQV--------PTTS-VQGRYKLRVEG----
NVNGVLGGTAFVNETV---LEFSQ-RS-MTIFVQTDRPLYMQGQ-IVKFRALPLTT-QLR--AFSDA-------------
VDVYML------------SPNRTIMRR----WLSRRTNL-G------AVSLEY-------PLSEQPHYGDWTIQVVAQ---G----------
QVEEHQ------------------------FRVEEYYQTR---------------FEVNVTM-PS--YF-VMS---QEFVEGSVMANFT-
SGAAV-NGNLTLRASVEP--------VKATYDIQ---------------YQF--
VRSIEQHFRW---------------------------------FEGYMTFKFA--VRDLLM--LSP--R------
LEGMKIVIKAEVGERFLDLIEFG-ESATVVFNSTLKLAFLGNS--PQVYKPGMPFKAYVALSY-HDNSPIEKGTLVG-
QR--IDEYLRISVNGARGS-------------------GNVVPVPVPG---------------------------------QKNMWEIAFDTGAPA--
-----------GGAEISP----------------------------PI--IRIEA----SFFDDF-----L-G-------RTGTELLLYPEFSP-SG-YH---------
---IQV--------TTSTKNPKV-------GEYIVLHVRANYFV-ER----------------------FSYLVVSK-------GMVLLSGYEVMQ-
-SS---SIRTMAVSLSSEMAPSATIVVFDVLK----REGR-IIADSLTFPV-
DAISKD----K----FTVQ----------LNNRKDKTGDNIEVAIYGPPG-T--YVALSGLQRAL----------------YEMHA--

GTQLSYS-----------------------------------E-----VIEKM-NSFI-P-MNGTL--KHETLS----HT---------G-
DASLFVNFPSS-SYGLD------ANRTF---------SYAGLVVFTDA-------SV-TRVR---
---------------------E--ECNITAGYLGCY-----DG-SCYPASRR--CDGFRD----CSDGSDETACPE---------
NRIDIKKFRMFRTNRYLLQYT---NAWLWKDIN-I-----GSLGRYIFTV---PVPDVPTQWMISAFGMTEANG-FGI-
VSKPISFSSAK---------------------------------PFYMTVEMPGRIRLGEQIGVRITVFNY-RHY--ETEVLITL---------------
-----ANSPDYKFVHV---DPFGWVNSYRPKTSFGEHQHLVF--------VKAGKSFEVYMPIV-----P-VRL--G-----
EINVTILAKTQ--------------------IAKDQATKTVYVEADGIP--QERHISMLLDLSQGAYLLKYLDTNIT------------------
ETPILPFRED-RLYIFGS-N-KATVSIV--GDVVGPAFPT------
MPVNASSL-IEKPAFCGEQNMFNFAANMWTLFYLRLTGQYR----QGD--QKEAFTYLNLLYQRQLQFQ-N--
DDGSFTVFR--WD-RR------PSVWLTAFALRTFQKAT--FQEWE-HY--I-YIDPTVIRRGVAWLL-STQSDE-
GSFHEFSIYP-YDRKMNSYSE-GHF----ERLK-YT--NVSLTAHVLITLCEVKD-IGGDVGA---R-MATAKGSA--
RRYLEQKLNI-IRNWEDPFELAIVAYALTIA----NSDDAEDAFKYLDDRKM--------------------
EAAGMIYWGRVRVD------PPRDKYENQRPYRQPRLPALYESSNVEGTAYALL-TFVKRNA-V-------------------------
---IQKEIVEWLNCQ--------RLHDGGWSSTQ-----DTVIAMQALT---EYTIQN-
RLS-----DVVDLTVTVDAPS----TPGFTRELRINKENLSKRQALTI-PNAFG------GVLIKVQ-
GSGLAILQMHVQYNIDTWKHLVTQPPAKAFDLDIR--
---------QYAFG-RNNSHITFTSCQRWT---RLEESMASGMAVLEFNLPSGYYVQQQRLDSYV---------------------------
----

-----------------------RY---ARVRNLREARYQE-----KKLEMYFDYL-
DDEPICVDVTISRWYPVANMSRYISIKVYDYYAPERFNESMFEMYHLYAMSICHVCGS--------------------------------
---YQCPYCPVFSDA---
ARLRVGLLIYAVVVLT--VLRAINLIR---

>Metaseiulus_4 gi_PIPE_391337209_PIPE_ref_PIPE_XP_003742963.1_PIPE_ PREDICTED: CD109 antigen-like [Metaseiulus occidentalis]

-------------------------------MFRVQAF--------LISLSL---

----------------------------FSVPCSSQEDPMTLWQRERDNRYSFNSLDESGNVT--
----------------------------------GPSND-----------------QV------------KFLVLASNTVRPGQIFRVHVHLI----KSPY--
ALVIRSSLSCD---------------GAEVAAALE-ELDAGMSTVMLLQV--------PESS-KSGRYIFRAQG----
NTGGALGGTAFWEERE---IYFRP-QF-LTILIQTSQLVYNIEQ-KIGARIVLLTT-ELK--PYEDP--------------
IDVYLLVVLVFHKSPVPQDSRGIIMKR----WTSRYPYL-G------VVSISF-------TLPEEYEAGWWTIRAQVL---N--------
--QVEEKR-------------------------ILLERWYTER---------------FDVYVSM-PP--YA-LTS---DEYFEGDISANFT-AVAPT-
YGNLTVRVILKP--------FSASTKSR----------------KPI---ALLDKFIEDYVHD---------
--------------------------FRGAHHFRYS--MRTLES--LALPYR------LSDCEVEVHASVGERYIDVIVDG-
FSKARIVNDSISVYILGDK--PLTFKPGMEFVFYVQVSY-NNHVKLPEDRLRD-ANVSVT-LSATGDYGRREM----------
---------RYRDHILDSN-------------------------------------GVAKIRD----------------------MVPE------------------------------
KTDRVIIRAVFTL---DD--------SR------TASAEEIMVAFHRP-DN-RQ------------LQI--------STSTTDGVA-------
GEYAILHVRSNFNL-EY------------------------FYYLTISK-------GTVLQHGKKTVLGLDH-------------------------------------
-------------TATTFALPLSPEMSPSLRVVVYFV-D----ESKDELISDSISIPV-RGINRG----S----FRIW----------
INKQQDRRGDLVEVVANYTTE-A--IVCSTSLDTDL----------------IANQG--RNDITPT------------------------------------
-G-----VVRAL-HRME-TNGTSLV--GGVARD----PD---------G-GSDELFFYATP-NSAVD------SNSTF---------
TSAGLVIFTNL-------YVAQVLD--T--SCNTTGKLACKT-----
GL-HCYLPIER--CDGYRQ----CEDGSDESGCEDI------LADDEDSFIFHVYRRNRFNNFFDATGSMFGYHDIF-M-----
GNLRQIFTKT---QVPKAATLYHVNAIAVSKQFG-FQV-LEAPVLHDSTR-----------------------------------
PFFMTTEAPGEAVLGEQIGLRVFLFNY-QHF--EIQAELIL--------------------HDSDDYRFVHV---
ESFGVVNAYKPRTSRGNHQHVVF--------VSPWQHIIIHVPVV-----A-SRV--G-----EVTFKLTGRTQ-------------------
-VANEEAEITINFLPEGVP--LQMHLAMMFDMRAISKNLKYFDFNIT------------------DTPIIPFDESHRRYTFDS-P-
SARISVI--GDVVGASSAD------
IPVALGEFGFATGVQSGEHALFDFGYHLAVLHYLRLTNQME----TDT--SRKLFEHLNQVLVDQTGYI-R---
DGAFTMFK-----DR------PSVWLTALAAKIYRQAI--FPDWEFRL--L-YIDTALVENSVRFLL-
RHQDPHAGFFYEDNGAL-HNRRHRAFML-DDQ----RQLRNFH--NVTLTAHVLITLSKVADVLTGSLLG---E-
LVIAQKAA--VGYLEEQLPR-LRT--SSYHLAISTYALKLA----GSIQAETGWMLLQTLRR---------------------
ETEGMVYWSSVDIG------PATVVYEAQRPFYYANMPAEDDALGVETTSYALM-LMMEFGSNE----------------------
---DESQVVRWLNWM--------RYTDFGFVGTQVRGENDTLLAMEALT---
KYSFRT-HVR-----DITDLKVKVQASA----NPGKVLELIVNKENLAQRKFVQVKPPVYG------QAEIHAE-
GAGLAIVQMDVTYNVD-RDYLLVPPAQDAFDLRVE---
---------TLFWG-RNKSHIEVRTCAKWI---LTRVSNFSGAAVVEVIVPTGYFHHRPIIDRYV---------------------------------
----

-------------------KY---GDQPRLKRAFIAP-----
RSAVFMFDTIPAYDWTCFKFRMERWFPVANATRYLKAKVYEYNAPENYKEKIFETMDIWVQNICDVCGS----------

----
YQCPYCPFFSGC---PTNVVIPLYVTVASLLFIMFQETMILRTD--

>Metaseiulus_5 gi_PIPE_391339074_PIPE_ref_PIPE_XP_003743878.1_PIPE_ PREDICTED: CD109 antigen-like [Metaseiulus occidentalis]

-------------------------------------MAFRFQNPRVVGTERICVV----PEPEKYP---RYPWVLDR-------------
GLDLLTALQ------------TL--------PATL-SPGRYRLLVEG----SMNDYNGGVAFKNETR---LILLQ-RS-
LTIVVQTDRPLYRQGQ-TVRFRVIPITT-EFD--GYQEA-------------LDVGML-----------NPGNIEMKR----
WLSRRTTG-G------WLALEY-------ALSEVTQFGRWTIRVKAN---G---------YTHEEH-----------------------
FYVKEYHTPP--------------FEVRVFS-PS--FI-VSS---DQFLRGTVHANYS-SGAPV-SGNISVVAVMEA--------
VHNEDSRE---------------SRG--YEMSLDS--------------------------------
FYGIWDFEFP--IGDLLR--STN--N------VQRFKLVVKAKVMDVRTSLVRKG-YSESLIFKKSYTLRFEGPA--
KQVFKPGLPFRARIELRY-QDGTPVEQEWFRG-RPVSHYLRASVFVTGLAAQ------------------PKLQHPRIFD--------
----
LGNTRWEIQIDATPNWSNSSRPIFLEVCSQCLALSQILHTFSRPGSGQWFGDLYLYRPLNLWLRPGVQYHSLRH----
ALRDSMAHLFDEYFG------VERSRLVLLPEFSE-NS-HH------------LSI--------STSTRNAKV-------
GEYIIFHVRSNYYV-ES----------------------FQYLIMAK-------GVIIKSSVESM---TT-------------------------------------
---------SIRTFAVTLTKQMAPKALIIVYDL-T----ADDK-LLADSLEFSV-DAIAST----E----MSVR---------TD-
VKDRSGANIELTVGGAVG-S--FVGLSSQPLDV---------------LSSFI--HHPISNK----------------------------------K----
-VDSAL-EFTDNNVPHTTQ--------------------------------------MD------THSTF---------QASGLALFSDA-------YV-
GRRP--R--VCNASSGYASCF-----DG-SCYRASKS--
CNGVDD----CADGADEFSCHHK--------HAVDWEKFRRTRRNLVSSDFQ---GTWLWKDIS-I-----STSGRNTVSI---
PTPTTPMPWALSAIGISPFRG-VYT-LPNPEMVTSPR--------------------------------
FFFMTVDSPEIARLGELFSVRVALFNL-GQT--KLEVLVTL-------------------
ANSLDYKFVYVNALERDNNDRSLQPITMFGEHQHLVF--------LWPQRPVVLLFPIV-----G-IRP--G-----
NTNLTVAAKTQ-------------------TSKTSISKTIRLQPEGFT--WSSWASMLVDIPRGAYIMRKFDTGVS---------------
--ALQEPPLALAAGQSPGDH-N-TAVITVA--GSVFGPPFPV---
---SPLTVETV-FGLPGGTAEANMFSFAANLLSLQLIQSNKNL-----VRK--HKQVFNHLFITYQKQLSFQ-R--
ENGGFAMFTHGEV--------PASVWLTAFTARYFHKANRLFPEWK-NF--V-HIDHKVLERSLAFLL-ATQAK-

SGAFHDHDGYFTYDRKMALKGA-DDS---------HKVANVSLTAFVVITLYELRN-LDGPLTI---K-IPPAIELA--
QNYLELSLRY-IDKDDDPYQLAITAYALRLI----GSSDAEYAFTLLDRKYI--------------------
QDGDLRFWSKRRYPSEPGGMPPFSLHPSQTPQVGPLARSDLHALDVETTSYALL-AYLHGKA--------------------------
--ILVKPIVEWINHQ--------RNNEGGWASTQ-----DSIVAMEALL---
EYEKRTFGSDAEE--RELSMTLTLEAPC----LPGISKQFHVSFANRFQVQRFRI-PNAWG------SIMVRAR-
GVGVSLLQMHLKSQLR----SPAPPVSSSLSLNVK---
-----VFSSGRNHSRLHFRSCQSWI---AERESLISGLVVLEAQLPTGYQVDRDTLLHYI--------------------------------------
----

-----------------HTATSRNLRHAHADQ-----SKATFYFDYV-------------SYSTGNRASPSLLLVKRGK----------------------
----

>Tetranychus tetur08g02040.1 pep:novel supercontig:GCA_000239435.1:HE587308:885859:897819:1 gene:tetur08g02040 transcript:tetur08g02040.1 description:""

------------------------------------MFLP--------IFTVII---

-------------------------FSV--
---------------------------------DKGLAQNPTYTILAPEKIRPNSDYFVSVSTH----HVYN--PVNVTLTISGYD--------
NSTKSFNEISQTIELSG-----EMTRLARF--------GVGPWGIGNYKLTTIG------TG---GLTFTNETK---LVYDG-KS-
FSVFIQTDKAIYKPGQ-KVLFRAIIVRP-NLA--LADTG------------ANYVYIE------------DAKSNRIKQ----
WDRVFTTH-G------VLSFDF-------QLSENPVLGDWTINVEI----D----------RKIFKKT-----------------------
FTVAEYVLPT---------------FSVDVTL-PN--YV-TYN---KSDLIANVKATYT-YGKPV-
TGEVTLTVQPRIRYGSLTTRPLEQYQV----------------KAK---

-----------------------LDGSVDIPVD--VVRDVK--LRKD--------FYDREIEFFALVEESLTGRKYNK-
TAILKVYDKDINIELIKT---APYYKPGLKVICYLKVAY-RDDTPVE-----D--NGPPVKIMYGYGYDEDAY-----------------
-NSIIEEVPNK-----------------------------------GIIKFEFTAVNN---
SHSVIGMKA----EYKG-----------------QVYNLNSFEMSLSP-SS-NY------------IQV-------AIPDGYQAKI-------
DETLRLSVTTTEPI-ES-----------------------FVAIVVSK-------GKIVSTETFYFKSGKQ--------------------------------------
-----------HVASLSITHSMAPKARIIVYYVRT----ANSE-IVADSVAIDV-GGIFRT----N----VEVS----------
ASTEKAKPGEKVDIKVKTNSN-A--YVAVLGLDESV----------------LLLKS--GNDITRD------------------------------------
D-----VTNEL-ATYDSNYYNPNSDDSFSYFNRNYF-------------------------NSLSA------AANIF---------
HNAGLIILSNG-------IVFDHSIQLHYRSS-------FPVSLSESADSMSAFDEAPNPVGSRFYTKSLSSSTAP------------
VKVRKYFPETWLWANQT-----SGEQILRYGFSIPGLAPSP----AGAVSGKSIVLDDKLL---
DYRPRLAKIVLPDATESRIVLRKNFPETWLWDHTY-S-----RSDGL-ATFSS--NIPDTITSWSVTAFAVDKRTG-LGV-
VEKPTKVTVFR---------------------------------PFFVKLNLPYSIIRGETVAIQVLVFNY-FTK--PREAQVVL---------------
-----H-NDNKEFNFTVASNEVDTYL------NDATRSQFVQ--------VAAESAASVTFLIS-----T-NRV--G-----
SINLKVTALTD--------------------NAGDTVIKQLLVKPEGKT--QYLNRAVLLDFSKAGGSSSN---HLV-------------------
--PLWIPNN---AVPGS-K-GLSVSVI---GDVLGT-SLN---N------
LDDL-LKIPYGCGEQNMLNLVPNIVVLQYLKTAGRLT----LDI--QSKALHHMELGYQRELTYK-R--SDGSFSAFG-----
ESDK---NGSTWLTAFVLKSFYQAK--------EY--I-TIDDKVLAEATDFLL-KQQKS-DGSFPEHGEIHHKPMQGGA----
AGS-------------QGALSAYVTIALLQNNYF-----KQ---Q-YPKHFDRS--EKYLYEQLRE-AK---TSYETNIIAYALHLM----
KSATIEPALAK--SLIK----------------MK---QDGDVSYWSDEDET------EKTNFTNKQS-SHFFLP----
KSTDIEATAYGLL-SLVN-----------HNQ---TDQAVTVMKWLISR-
-------QNAQGGFSSTQ-----DTVIALQALS---SIASHL-TSS-----TQ-NIDVTFKFGNVTEKQTSRTFQIR--
DNNAQVLQKFEM--TDARGETIPDHVEIVTQ-GTGVAVVQVSWRYNLA---VSAE---EPAFFLNPI---LG--------------
---KASTDNFLQLN-LCTYYK---A--GLA-
TNMAVMEVELPSGYSADVDALQSIK---

--SQGNGIKRIESHNGD-----
TNVVIYFDRL-TREELCLTVPAHRTQKVANN-KPVPVTLYDYYNRHESARIMYEP-KMTT-TCSI-CSS------SDC---QC-
---NNEGENAIKE--ASF-----------------------
DGSSGFIASTNLAIVLLLQLISFVCII--

>Tetranychus_2 tetur04g03640.1 pep:novel
supercontig:GCA_000239435.1:HE587304:2006927:2008000:1 gene:tetur04g03640
transcript:tetur04g03640.1 description:""

---MWK---
------------------------------GEW---

--IGF---
-----------FGYFVL--LIS-

----------VLHEF---

--------------LNFLRQSIT--IIDSYAVQHDPRNLQHRQKRSIKREN-----LFDSIDPDKGEQKTRIDLSPRTQTAAIVPDS-
E-ECVVSAI---ADSLGPSVITTLSN------INHL-
IIKPTGCGEQNVIRMAPTLFTLDYLNATGRLT----ATQ--RETGLKYLKAGYENQMMFR-K--LDGSFSTFE-----KR-----
PSSLWLTAFVTRILCKAA--------PFLGD-SLDPEVILTAVDYLVDHQESSPSGSWKEYHPVIHKSALGG-----LTG-------
------VIPITAFTYTTH---
--------------------------------------TTSAY---
---NLTDINLICNISS---GRFRKSIEFH--EDNAQVMRIFEI-
NNECD------YVDMITR-GTGLGSVRVKYK---

>Tetranychus_3 tetur04g03660.1 pep:novel supercontig:GCA_000239435.1:HE587304:2008952:2016911:-1 gene:tetur04g03660 transcript:tetur04g03660.1 description:""

-------------------------------------MAPKIFRLYTQENVVVL------------
PLTEEAFLLEIKANDQVLFTKYFPKSPKNPFSVPIELGANVSTGVAKLSV--------INRDGDENSSQIIITG----------------
------------DT-GFGFIQTDKPIYTPKE-KVRIRLMRLDD-DLK--PMADK-------------VKLSIK-----------NPHKIIMDE--
--VVLESSKDNY------FINHEF---NIPPHFLQDPVKNKWTIIMAYG---P----------EFQVTSN--------------------
ATFEVREYILPL---------------FQVTLKS-LQ--FI-TPS---TSIVNGSVKATYL-TGKPV-
TGTVRFKFKIRDSTNRSIGIGQTDELEL--
--------------------VKGETSYKFS--TNEFSE--SGVEVSM-----
IIGSVLVVEATVIEAATRQKVKHIDSSTYFVSSPYKVSFESS---FRVFRPEHPIRLIAEIYD-VHNQPVV-----G-
IPVRLSVFSSRM-------------------------VTKDVISDDL------------------------------------GRVTVDYDTTT---------------
------------------------------------QDKKIIFEV----RTRDAKLKE------------DEQSFSRLLVEPSN-QTSAA------------LTL--
------VEKNDRFKV-------GDKYTNSIIFEGST-FIFSQ--------------------AYYITVVR-------GRIDKLERIPDNDNFG------
--FTIEPHMAPSFRLIAISY------RYDR-VVSDSLLINVDPPECSL----N----
LTYT-------NSKGATNDIEPGENGTLIVSANQSENRRQISIVGVDEAV----------------YLLRS--ANTLTRH---------------
---------------------------GLRKMFNSKDKGCGP--GGGID------PTDVL---------
LNSGLVIAGIPSNSIGSSCVHMNDKSSKRRPIRASPRIYITSSSSSMRKFASSPTYSSHSASPVSSSKQYSTNAFINQCC
RLGKIKPNDKAKAMSCEERRDILLR-----SVKNVNCASAFLDCCLNSIIPPLLRTSVFDVSSGESKGQ---
DNTEAGVTLGEEDAIEESTLIRQDFRETWLFDLVT-L-----DESQTSVKYPV--TVPHTITSWRLNAMSLSAKDG-LCL-
MDKPLRLISNK----------------------------------ELHIRVDLPYSIVVNEQVEMLVTIFNN-GPT--RKKVNLFM------------
--------YGVDGVCSEADA---------------GQKTERRLVT--------VEPGMLHTEGFALS-----P-IRT--G-----
EFKIQVDALAH--------------------GTSDVVIKTLHVVPQGIT--IIDSYAVQLDPRNLQHRQKRSIKREN-----
LFDSIDPDKGEQKTRIDLIPRTQTSAIVPDS-E-ECVVSAI---
ADSLGPSVITTLSN------INHL-IIKPTGCGEQNVIRMAPTLFTLDYLNATGRLT----VTQ--
RETGLKYLKAGYENQMMFR-K--LDGSFSTFE-----KR-----PSSLWLTAFVTRILCKAA--------PFLGD-
SLDPEVILTAVDYLVDHQESSPSGSWKEYHPVIHKSALGG-----LTG-------------VIPITAFTYTTLRSCENFTYPRILA---
KRRDKSLKLA--ESYLCGKLTSELAKG-DPYHLALLAYSLSTTTCLQSAERRKSIISRLREIGV-------------------

YSSGENKLFWN-----------------------------TTTPIETTGYVLL-ALLNLRETK--
----------------------PDEIKAIINYLESQ--------RSYTGAFDATQ-----DTIVALEALS---TYAKSAY--------
NLTDINLICNISS---GRFRKSIEFH--EDNAQVMRTFEI-NNECD------YVDMITR-GTGLGSVRVKYKYNVL-----
EAPEKLCGFELDVNVTQAIDSPEQIILSDSDIQLDDLFNITMLAEIGVLNDR-------
LVQVESVVDSTLTDNNDSSSSKTGPSYTANEKAESNLKLSGASKVTNKLTVCAKRF------
DSVDSGMVILEVGILSGFVPDENDLVALK--

---KNNPFISSYEKTA-----
RSVIFYLEDISSKQQYCLNFKIYQENKVANL-QSAMVKIYDYYKKGQGCSQLYHSTRRTDYLKKI-CNS------
SICHCTAR--
WVCPLNDNLNEVGEIGSVNIIRAKDKFARLLCSGQYGFVWLGRFVKHTELDSLVLLNFKIGQSIYGDGQEGQ--------
----
DRIFTMDRSCFEPNMLRTTFTETFLLMGKSRNTTTLHLDSRSILYDLENIGSKDPKIHYTIKRVIDDFKRTRRQLTC-----
----

>Tetranychus_4 tetur04g01020.1 pep:novel
supercontig:GCA_000239435.1:HE587304:621246:636316:-1 gene:tetur04g01020
transcript:tetur04g01020.1 description:""

--------------------MKYLDTNLT-----------------DTPIIPLRND-RRYIFGS-T-KARISIV--------------------------------
------------------------GDVVGPVFPS------MPLTSENL-LRKPYGCAEQNMFNFAANMYTLLYLRLTGQKS----ADL-
-EKQAFHYLNIQYQRQLSYQ-N--EDGSFSAFRFGEK---------PSVWLTAFVVRIFHKAT--FQEWE-SY--L-
YIDRKIIEKATQWLL-THQTSE-GAFYEIGLQP-YDRKMNSSSK-IAS----DSVR-YR--NISLTAHVLISLSQLHG-
LSGHLSP---L-ISNAKHTA--VQYLEKMLAV-VKSHDDPYELAIVTYALTLA----NSADAESAFNELDNRMR------------
--------EVSGMRYWSKLPLP------GPKTTIENNRPYLLPRRPHEYDALNIETTAYGLL-THIIKTA-V--------------------
---IQREIVEWLNTN--------RLHNGGWASTQ-----DSIIALQALV---EYSIQS-
RIR-----DITDIEVTIEAPS----SPEFSERIEINSQNLARLQSFEI-PKAYG------PVIIRAQ-GSGLAIIQLDVQYNVD-
WPQFQIPPPIRAFTLDVK--LRSFG-
RNNSHINYRACSQWI---LTEESTSSGMAVLDLTIPTGYIIQQQDLDAYV---

-KS---GKVRNLKEAKFSE-----RKALFYFDYL-
DSNPICVTFTTQRWYPVANMSRFIPARVYDYYAPERYEEQMVSTYDLYRLSICHVCGS----------------------------
----
YQCPYCPIFSGAQGVTKINLQMMLLSFIILIITTRQLNQNLILVT--

>Ixodes ISCW003923-RA alpha-macroglobulin, putative|protein_coding|DS716413:7286-62557:-1|gene:ISCW003923

----------------------------------MLYFIRHISNSLDGHATIQLL----APSG--QVLSETSIKSAG-----------------------------
GFPQTVSL--------NVPKELPVGSSLAAIG----K-FG---RYSFDKRLS---VKVADGSAGLIIIIQTDKPVYRAGE-
VVRLRILSIDH-RKL--PLA---

----------KDA---IKGCHEFTLN--GRD-LL-
-LANEDL------NRRP-LILTAEVTEEAT--AP-----G-
ESVNICFLAVNVTMREVSK------------------GCRDFKSGPD-----------------------------------GLVRFSVPGLR-----S--
--SPSSLRVTA----KAEGGSLN-------------PPTTTVLLQPWRSL-SQ-RY-----------
-LRV------LAPGQKLKCGV-------STPVRFHYTKLKNH-SSVA--------------------FNYQVFSG-------
RRVVEQGRFEPRVSQDDTDDRIPVESGLQSGGFITA----------------------------ELSLEPKPEWGLSSKARLLLYYA-H-
---PKGE-LIGDSRRLQV-EPCTSN----P---

--WQAGALCLHPTRG-VGL-S--NAALNALQ------------------
--------------PFFAQLTLPAVAKRGELVPVTATLFSY-LQA--CVPVKLSL-------------------N-ADSACMKL-------------
----LGEPQKRDC--------LCGNESIVLRVQVR-----P-LCL--G-----AINITLHALGLAENKGLC-DEGKPLETR---
QARDSLRKSLLVTAEGQP--QEKSTAKYICLNGSGEGPQK---HEF--------------------PLELPRN---VVSGS-S-
RGVFTLS---GNVGSPVSVD---R------LDKL-
VRLPTGCGEQNLALLAPNVFVLDYLNSSGERG----HPL--ESKLKENIAKGYQRQLNYR-H--AEGGYSAFG-----
SQDP---EPSLWLTAFAVRTFGRSR--------RF--M-PIDEAELSGSIRWIL-SNQYD-NGCFPSVGRVLNSRLKGGL-E--
GHS-------------LAPLTAYVLISLLEAG------------A-EIPQVSRVGAVRCLLQELQQ-QQ-Q-DSHTMALAAYAFSLF----
EDSKASELLASLRDEAQPGNGESIFFFFFLCFLRT---E--ATLHWHKNVS---------------------------
TAVAVETASYVVL-ASLKLGG--------RAA--
AKSVLPVVRWVLEQ--------RNHDGGFVSTQ-----DTVVALQVLA---EYAKAT-SSS-----DT-RLQIRVSSEG-----
LGSTLTVN--RENALLKQELFL-PVVPT------VLHIEAT-GTGCAFIQATVKYNVP---AAEE---TKSFGFWVTALHAN----
--CRPQVKVNLFS-FCSYLV------
SWAMSGMVIVQVKMQSGYRFEKVGASSWC--

LCLEEQALKRHEVDQ-----DQVNLYFEEL-SNRKSCALLELVQDYVVENA-VPATVTLQDYYEP-------------------------
----

>Ixodes_2 ISCW023777-RA alpha-macroglobulin, putative|protein_coding|DS970697:293496-343163:-1|gene:ISCW023777

---ESTFLPF--------
KVPKHIDSQAKIEVNG-----TFG---SYVFGDKKE---IDFQK-SK-TNVLVQTDKALYKPGQ-KVQLRVLPINN-ELK--
PVTDV-------------QATIYVT------------SPGDVRIAQ----WNNVTFEK-G------IVQRDF-------
KLSEEPELGLWQIVVELP--------------SLTVRQH-----------------------FEVNEYVLPK---------------FEVTVKP-PS--YV-
LAD---AKEITWKICAHYT-FGQPV-DGTLKVNVTYERYSWEKDDYPRVSHEGP---
--INGCFDMTVN---TTALR--FNENY-------
EIYKRLHLITSVNETGTGITMNK-TSYVSRSFNPLELTFLEGEHGKNYFKPAMPFYGSLLVKK-PDGVPVG-----G-
ERIQLCLLSQSEIIKPLWWRTDRRL-------------SCKNYTSDEN-----------------------------------GLVKFTIPPMKT-----
---SVVTISVEA----VAVNYETVKYDTYGVKIN---QPKSTLYLQAWYSS-SN-NF--
----------IQV------EPSKVPVSCTS-------RHPIRVRYTAEADK-EIQ---------------------FHFQVMSR-------
GKILKDAVVPVTFKADQAVVTEVDETYLVEEERNETLPSNVAEEQAA----------------
TGSLEYELTPDFNYAPQVKVLVFYV-R----PDGE-VIADAEQFEV-EKCLQN--

-----------------------------NESGMVVMSDL-------TLETRPCRKAIYDR----------
PPYALAAPASPPPPPAFANRVSLVGGAPAVATLD--
---SLPEAIAVNREIPAKSAVEVRNYFPETWLWDLKE-L-----DENGQ-LSFKE--KIPHTITEWVGSTVCINSQDG-VGV-
S-DPAKIKAFQ---------------------------------PFFASFSLPYSVVRGELVPVKVSVFNY-LEK--CLPIGLTL----------------
-----AESEDYTI------------------QDSASTTLC--------VCGSKSLTHKFLVR-----P-KTI--G-----EVNFTVSAAGS-
NSDTVCGDQKVEKV-----VARDAVTRPLIIEAEGFP--KEETKSVFVCPKDVTEGDGK---NEF--------------------
DLVLPDD---LVEGS-A-RAYVSVT---GDIMGP-AVQ---N------
LDSL-VRVPTGCGEQNMIKFTPNVYVLDYLKATGKHE----EDI--EKKAVENLKTGYQRQMKYR-H--PDGSYSAFG---
--TSDS---TGSMFLTSFVVKSFKQAE--------KY--V-PIDAANLKESI-----KKGSS---
----
---------------ASADIETAAYAVL-TYVKLGD--------KEN--
LGKAQPIVRWMATQ--------RNSRGGFSSTQ-----DTVLGLQALS---AFATHV-SKD-----PV-DISVKVAGAD-----
VDESYDLK--EPTKLVIQEKKV-TNLPN------KLAVETT-GSGCALISTTLKYNVH---TPPK---SEGFELTVT---------------

--PTQEPNKCNTADVKVCLR-------------

>Ixodes_3 ISCW020822-RA thioester-containing protein, putative|protein_coding|DS837598:3653-100934:1|gene:ISCW020822

-------------------------------MDATRLL--------LLTSAF--

----------------------------L---
-------------------------------PLVAP---QSSGIYTIVAPRKLRPNLKYHVSTSLS----QSASSPPVDLRVTLSGPS---------
DNGSSNRITKQVQLHDPFGLLREQRW-SS--------QVGDWGPGKYKLSASG------SG---GLDFFNETE---LTYEH-KS-
YSVFVQTDKAVYKPGQ-KVLFRVIVMDP-YLL--PTVTG-------------AMNVHVT------------DAKGNRIHQ----
WDRVLTQK-G------IYSSEL-------QLSDQPVLGDWAIHVDI----L----------GQKYSKN----------------------
FTVAEYVLPT---------------FEVRVKL-PA--YA-TYN---KSEVVATVSATYT-YGKPV-
KGTVTLTVAPRTRYHQLRPRPYEQYQT-----------------KAE---
------------------------IDGSVDIPVA--VVRDLS--LKTD--------FFRRDIEFFALVEERLTGRKYNS-
TSYLTLHDKEVKVELVKT---SETFKPGLKYTCFLKVAY-QDDTPVH-----D-AV-NQLTLYQGFNFNEDLW--------------
-----KTSRHWVPAN-----------------------------------GVVRLELFPPNDN--
ATVVLGLRA----EFRG-----------------QTHYLEGIYPARSP-TR-SF------------LQA--------WVTTEDPMI-------
GDLVEVEVNSTQPL-DH----------------------LVYEVMGR-------GDIVFAQTLPASGVRT------------------------------
------------------YRFSFSTSFRMAPRARVLVYYYVR-----KDGE-LVADAVNFDL-GGILRT----P----VQVQ----------
SNLAETKPGGQVDILVSTRPN-A--YVGLLGVDQSV----------------LLLKK--GNDLSQE---------------------------------

--Q-----VIEEL-ESFDSGKQARVWPPWYRRR-RRSLWW----------------------PGSTT------AHDLF--------
KDSGMVVLTNG-------LVYESDDGLFARKQ----------------------VIRLDTDVLTNPVLPPSDLP----------------------
---EAPPPVPGRIRLRQQYPETWLWSNVT-A-----SHDGR-
VVISS--TVPDTITSWVISAFALDSLTG-LGI-APSQAKVTVFR--------------------------------
PFFVTASLPYSILRGESVAIQCVVFNY-NNK--PVQARVTL-------------------E-NAKSEFVFTSLSNDVGGEQ------
SKDRRSKEVT--------VPAQDGVAVSFLIT-----P-TKL--G-----YIDIHVSATSS-------------------
LAGDSILKKLLVKPEGSK--QHFNRAVLVDRRNPSAPPTS---TNI--------------------SIPIPKN---AVPGS-E-RISVSAV-
--GDLLGP-HVN---N------LDQL-
LVMPHGCGEQNMLDFVPNVVVLDYLRRANRLS----PAV--RGKALRNLEDGYQRQLTYK-R--DDNSFSAFG-----
NTDR---SGSTWLTAFVLKSFVQAV--------PY--T-SVDPAVLENATRWLV-ERQKP-DGSFEEPGEVIYKPMQSG-----
AGS------------GAALTAYVLIALLENK-V-----GF---Q-HALRFAASAAEEFLLKELRT-QS---DPYVVAVVTYALHLS----
GHRARDGAFQKLLSLAT---------------RE---D--DMVFWKDPGV-------APVNTTDKQS-DFFFKA----
HFKDVEMTAYALL-TLME-----------RGD--
VSAAIPVMRWLVSK--------QNSNGGYSSTQ-----DTVIGIQALA---RLAASV-VSQ-----TI-AVDASVKYGD----
GRKRTLKIH--SGNALVLQRIEL-PSDLK------YVEIESS-GFGVAIIQVSWSFNLA---VSSE---APAFFLNPL---LD-------
---KTSTESYLQLS-VCTHYR---G-EGEA-
SNMAVMEVGLPSGYLFDFDTLSSIH--

--RTKEVRRVESQDSD-----
TNVVIYFDRI-GREELCVTVPAHREHKVANQ-KPVPVKVYDYYDLARSARMFYSP-YKTT-LCDV-CDG------VECGN-
DC---NTVKGTKAGT--DQLER-E----------T--
----EPDAAADTRA---SLAAVLALSLTAVLFVRWW--

>Ixodes_4 ISCW003089-RA conserved hypothetical protein|protein_coding|DS687147:36344-71042:-1|gene:ISCW003089

---MRQV--------
PQSA-KPGKYKFRVEG----NVNGVLGGTGFIQERD---VEFQP-QF-LTILIQTNQFVYNYDQ-SIKARIVLLTT-ELK--
PYTEP-------------VDVWMLLLLQ--------DSRGIVMKR----WVSQYPYL-G------FVKINF-------
ELPHDFAVGFWTLKVVAL---S----------QVEEKK------------------------ILLERWYTER---------------YDVYVTI-PP--
FV-LDS---DEYYEGDVSANFT-TVNPV-YGNATIRLY--
---FQAGYQFKFP--MSDLKD--LAAPHS------
LDKCEIEVKAAVGERFLDIIVHG-YARSRIINSSLSLKILGVK--PLVFKPGMTFTIYVAVTY-HDLVKLPEEKLKE-
SNITLSFTGQGGGGGL---------------------NDIETPPNEK------------------------------------
GMATVEITPPEGTEKIVIRVNTNQYIVPSYKVKNCHSSC-----------------------CIHFIALQA----KYRDK--------ED------
VVETEALAVAQLSP-GK-KF------------IQI--------STSTSNGVA-------GEYAIFHVRTNFYI-KF-----------------------
FHYLVISK-------GTVLQAGVQKAYGLLQ--
TITSFSVPLSPEMAPAIKVMVYQI-S----GNGD-LTADAVIVPV-RGINRG----N----FYLW----------
KNLKQDRSNNLIELVPTYTTE-T--IAGLNGLDSDL---------------VAVQG--KNDLTPT-----------------------------------
-S-----VIESF-YRME-DHWGSHI--RAIWRD----RD---------G-KPDEAEYFVTP-SFAPD------VNKTF---------
IFSGLIVATNL-------NVTTLPS---MFRFCTPA---------------
YCFERMFR----FLVP----FMLSLDEQTFYDGEQD---DLEDKPLFEFFLYRTNRFNNFFDATGSNFAWHHVK-T-----
GNLKDIYTPC---IVPKPPTNYMFNAIAVSRKYG-FAI-IDKPIYHNSMR----------------------------------
PFYMTYEAPSTAVIGEQIGIRVVLFNY-QSY--LIQAELRV--------------------LGSDDYRFVQV---
GPLGRVGAYNPVTTKGEIQHIVY--------IQPYQHIIIHVPIV-----S-VKI--G-----DVEVNLVATTQ-------------------
VAREEATISITFLPDGVP--LRMHTSLLMDLRAQAYNLKFLDLNVT------------------EDPIIPFESQYRRYLFGS-P-
AAHVSVI---GDVVGTPLEG-------
NVEPEDFGFSAATKSGEHAMFGFAYNVLRLTYLRLTDQLT----RDI--AKPIFEKLNKAYVYQSSYF-K---NGAFTMFK-
----RQ------PSVWLTAFSIRMYELAL--FPDWE-NF--I-FIDPRLISQGVTFLL-SHQQPN-GAFYETTKHP-
WNRRMNEKVLGRDG----RYLN--R--NVTLTAQVVLALSKV--TLTGDIRQ---Q-ATTARAAA--VKYLERQLAQ-LK---
DAYAISITTYALLEA----GSNEAEYGYNLLESIKR---------------------DAEGAIYWSSVPIP------
PPVIISQSQRPFLMPRFPHPDDSLAVEATSYALI-IYLKQGG-L--
---------FQDQIVRWLNRM--------RTTDYGFIGTQAR---DTIAALEALT---QYSFRT-HVR-----GITEMKVTVESSS----
NPGFPSTLQISKNDLARRKVFDVQPNVWG------HCDVLAK-GAGLSVIQMDVSYNVD-RDFLLVPPAYEAFELTLQ-
---PHFSG-RNRSHINIKSCVRWT---
LTNMSEASGAAVLELAIPTGYLNYRPTLDAYV---

--KS---
RVVPRLRMAKVAP-----RTAVFMFEYL-
TNEWSCVDFLIQRWYPVANSTRYLKAKVYEYNTPENYKETIWENYDLFVLSICEVCGS---------------------------------
---YQCPYCPYFSSA---SS----------

ISLSWVLMFSTALLCQELYRKVTQ---

>Ixodes_5 ISCW020822-RA thioester-containing protein, putative|protein_coding|DS837598:3653-100934:1|gene:ISCW020822

---MDATRLL--------LLTSAF---L---PLVAP---QSSGIYTIVAPRKLRPNLKYHVSTSLS----QSASSPPVDLRVTLSGPS---------DNGSSNRITKQVQLHDPFGLLREQRW-SS--------QVGDWGPGKYKLSASG------SG---GLDFFNETE---LTYEH-KS-YSVFVQTDKAVYKPGQ-KVLFRVIVMDP-YLL--PTVTG-------------AMNVHVT------------DAKGNRIHQ----WDRVLTQK-G------IYSSEL-------QLSDQPVLGDWAIHVDI----L----------GQKYSKN----------------------FTVAEYVLPT---------------FEVRVKL-PA--YA-TYN---KSEVVATVSATYT-YGKPV-KGTVTLTVAPRTRYHQLRPRPYEQYQT-----------------KAE---IDGSVDIPVA--VVRDLS--LKTD--------FFRRDIEFFALVEERLTGRKYNS-TSYLTLHDKEVKVELVKT---SETFKPGLKYTCFLKVAY-QDDTPVH-----D-AV-NQLTLYQGFNFNEDLW-------------------KTSRHWVPAN------------------------------------GVVRLELFPPNDN---ATVVLGLRA----EFRG-----------------QTHYLEGIYPARSP-TR-SF------------LQA--------WVTTEDPMI-------GDLVEVEVNSTQPL-DH-----------------------LVYEVMGR-------GDIVFAQTLPASGVRT---YRFSFSTSFRMAPRARVLVYYVR-----KDGE-LVADAVNFDL-GGILRT----P----VQVQ----------SNLAETKPGGQVDILVSTRPN-A--YVGLLGVDQSV----------------LLLKK--GNDLSQE-----------------------------------Q-----VIEEL-ESFDSGKQARVWPPWYRRR-RRSLWW-----------------------PGSTT------AHDLF---------KDSGMVVLTNG-------LVYESDDGLFARKQ-----------------------VIRLDTDVLTNPVLPPSDLP---EAPPPVPGRIRLRQQYPETWLWSNVT-A-----SHDGR-VVISS--TVPDTITSWVISAFALDSLTG-LGI-APSQAKVTVFR---------------------------------PFFVTASLPYSILRGESVAIQCVVFNY-NNK--PVQARVTL--------------------E-NAKSEFVFTSLSNDVGGEQ------SKDRRSKEVT--------VPAQDGVAVSFLIT-----P-TKL--G-----YIDIHVSATSS--------------------LAGDSILKKLLVKPEGSK--QHFNRAVLVDRRNPSAPPTS---TNI--------------------SIPIPKN---AVPGS-E-RISVSAV--GDLLGP-HVN---N------LDQL-LVMPHGCGEQNMLDFVPNVVVLDYLRRANRLS----PAV--RGKALRNLEDGYQRQLTYK-R--DDNSFSAFG-----NTDR---SGSTWLTAFVLKSFVQAV--------PY--T-SVDPAVLENATRWLV-ERQKP-DGSFEEPGEVIYKPMQSG-----AGS-------------GAALTAYVLIALLENK-V-----GF---Q-HALRFAASAAEEFLLKELRT-QS---DPYVVAVVTYALHLS----GHRARDGAFQKLLSLAT----------------RE---D--DMVFWKDPGV-------APVNTTDKQS-DFFFKA----

HFKDVEMTAYALL-TLME-----------RGD--
VSAAIPVMRWLVSK--------QNSNGGYSSTQ-----DTVIGIQALA---RLAASV-VSQ-----TI-AVDASVKYGD----
GRKRTLKIH--SGNALVLQRIEL-PSDLK------YVEIESS-GFGVAIIQVSWSFNLA---VSSE---APAFFLNPL---LD--------
--KTSTESYLQLS-VCTHYR---G-EGEA-
SNMAVMEVGLPSGYLFDFDTLSSIH--

---RTKEVRRVESQDSD-----
TNVVIYFDRI-GREELCVTVPAHREHKVANQ-KPVPVKVYDYYDLARSARMFYSP-YKTT-LCDV-CDG------VECGN-
DC--NTVKGTKAGT--DQLER-E-----------T--
----EPDAAADTRA---SLAAVLALSLTAVLFVRWW---

>Mesobuthus MMa28752

MEISKIHTYPASNGQVGSSHVLQKPASPR---

---------------------------------------YGRPCTKMVPQIGCVAYLMCFF--------LNT-VL--------------------------------------
----

--FRVDC-VRLYRVE--DENSDTVL------ENSLQP-----------------
--GKYAH-----------------QP------------
LYLVVASKIVRPGQVYRVAMTIV----SSSH--PLRVRASLQRE---------------GEEVASAKE-NMESGDSKLLLMEV-----
---SSSV-RSGRYSLRVEG----NINGVLGGTGFYNETE---LEFSP-NF-LTILIQTNKLVYNYEQ-PIRIRAILLTT-ELK--
PYADP--------------VNIYLI------------DSKGFIMKR----WVSIYPHL-G------FVSVHF-------
MLPRDFPHGWWTVRFVVL---G----------QIEEKK-----------------------ILLERWFSHR---------------FDVNVIL-PA--
FI-LDS---EEYLEGSIMANHT-NVALV-QGNATIRAYVRP--------IKKYNQD------------------DL-------------------------
----------------WQKDLFLQENVIQ---------------------------------FYGVYDFSFS--MRSLQR--LAGSTP------
LDQCEVEVQASVGERFYDAVVTG-YARAHIINNSLNLYFLGSQ--PQIFKPGMTFSLRIAVSY-HDFVPLSEERLSS-
SSLIIEPMALIKTGGRRKL-------------------REILEHFDSS-----------------------------------GILEITI----------------------
DTPQ----------------------------DAEKIEIRA----RYEDR--------EG------ETQTSITLLAHYSQ-QW-RN------------LQI--

------TSSTKLGQA-------GEYAVFHVRSDFHL-KS---------------------FNYLVISK-------GIILYTGTEEVTGLIK---------
-----------------------------------SITTFTITISPEMAPSLTIVAYHI-T----NDGE-VIADSMILPI-NAINRV----P----
FKLD----------INVKQDRTGNIIELIPTLTTE-A--RVGTSGIDLDT---------------ISVQG--YNDLTPS--------------------
-------------SFPQPTLPQII-RTLFFTHFKTVV--LNYWRQCINLRNIIYSSSVYIG-KIEKVDLVKFN-
ILAHQIMEWILTRHLL---------YLSSLIIFTNL-------KV-TTYP---
-N--TCHESLE-SLCR-----NG-DCYPNDKK--CDGYKD----CSDGTDEAECESKA-----ENE---
LLHFFLYRKNRQNAFMDATASDFAWHDVL-L-----GHQNNDFVEC---EVPKGPSTYRLNAIGMSKVHG-LVI-
LEDQPLHDSAR------------------------------PFFIMVESPEVAIIGEQIGIKVVLFNF-QRF--EIKAEVIL---------------
---APSDDYKFVQV---EAFGIVSSYSPRLTSGEHQHLVY--------IKPYDQVIVHVPVV-----A-TRK--G-----
IVEVVIIGRTQ------------------VAKDVVTIPIEILPDGAP--VTVHTSFLLDMRSEAYNIKFLNLTVT----------------
EDPIIPLEEMYRRYLFGS-A-SARVSLI---GDVVGAPLPE---D--
SFVDVTDIGHSYPVKSGDHVMFNFAYNLYTLIYLRLTNQMQ----API--TKKILEYLNQAYVFANVYF-K---
DDAFTMFM-----KE------PSVWLTAYCIRIFHLAQ--YPDWE-NN--L-YIDPEIISKAMRYVL-RQQSPQ-
GSFYESTIHP-WNGRMDPRSK-NPL----DHIQ-YR--NISLTAHVLITLSEVSD-LTGDLRI---E-VTSARKRA--
ANYLETQLPG-LN---HPYEISIVTYALMLA----GSADAQVGFSKLENIKL-------------------SAEGLVYWSPEKIP------
PPQVIYQNQRPFLQPRLPQKHDALAVEATAYALM-VYINYGG-L---
-------------YQEQIVKWLNTM--------RMFDAGFISTQ-----DTIVALQALI---EYSFRT-HIR-----EITNMKLTVESSS----
NAGNIETIFVTQKNLAKKTEIE---

>Mesobuthus_2 MMa43260

---------------------MNEVNV--

----------------------IKINKGLSVIRRKMFQGVVLLV--------VLAATS--

--MAELTADSYMEMSGNEIYR---
---ERIKQ-----------------EP------------TYFVLASKVVRPGQIFRVVVIIY----RSTN--
PMTVRASIHRD---------------GVELASSSQ-ECKLKTPETLLLKI--------PPTS-TVGLYKLRLEG----
NVNGVLGGTAFANETY---LEFSQ-RS-MTIFIQTDKPIYQQGQ-IVRFRAIPIAT-NLK--AFSDA-------------
VDVYML------------DPYGTVMKR----WLSRQTNL-G------AVSLEY-------PLSEQPVYGNWSIRVVAQ---G----------
QIEDKS------------------------FCVEEYYQTP---------------YEVNVTV-SP--FC-LNN---DKYIYGRVEANYT-SGVAV-
TGNLTIKIFIVG--------YGS----Q----------------KSH---FV-TEKHYKY-------------
---------------------FDGVQEFKIP--MNELKW--KIQ--Y------LDKSTVTVEAHVGEHYLNRIEMG-
FAQTIVFNSDIELKFLGSS--PQVFKPAMPFKAYLAVSF-HDGSALPSWRLSN-RQLEIKTVVTFKNNKIKNL------------
-------NTHQEQMLPT-------------------------------------QPGIWQVNLDLHTQL-------------NGTNLN---------------------
---------DVYAVDLEA----LYQDD------S-GD------AAKANLLIYASYSP-TN-HR------------IHV--------STSTKYPKV-----
--GEYIIFHVRADYYV-QK-----------------------FYYVILSK-------GMILFAGYEDM---IS---------------------------------------
------------TIKTFAITISAEMSPSATIVVYDI-A----REGI-VVCDSLIFPV-DGISRN----N----FSVT----------
LNEKKDKTLNTVEVIIKGKPG-T--YIGLSAVDRVL----------------YSMHT--GNELTHA---------------------------------------
Q-----VLGKM-NTFD-EAMNGTL--SHMWHS----SI---------G-SPGKIVHFPSS-SYGID------TNRTF---------
EFAGLIVFTDA-------NI-TKRP---E--YCDQSQGYRTCM-----
DGISCYYLQNQ--CDGKND----CDDGSDESGCPSQ--------NILDLEKFRLKRFNRIQRLYD---SSWLWQDIN-I-----
GPLGHYIFTV---PVPSLSTHWIISAFGMNSVHG-FGV-LSEIVEISGLR------------------------------------
PFYMTVEMPSKSIQGEQIGIRISIFNY-MYS--ECEVVVSL--------------------GTSSDYKFIHV---
EPFGVVHSFSPRTSVGEHQHLVF--------VKPGKSTVVYMPIV-----P-TRL--G-----DINVTVMAKTQ-------------------
-VAKDMITKTIHVEPNGVP--QYRHTSFLLDLSQGAYLIKYLDTNLT------------------ANPIIPFRQD-RLYVFGS-N-
RATVSIV---GDVIGPIFPT------IPVNATYL-
LKKPFWCGEQNMFNFAANLYMIRYLRLIGQRK----PYI--EKKAFHFLNIGYQRQLSYQ-N--DDGSFSLFK--WK-SK--
----GSVWPTAFCARIFYNAA--FQEWE-NF--L-YIDPSVITKAISWLI-KHQSPD-GAFYETTMFP-YDRKMNLTSD-
WKH----DNVK-LR--NISLTAHVLITLTEVKD-LGGEIGI---K-ASLARSKA--QTYLEKKLSI-
LNNFNDPYELAIVAYALTVS----NSIDGEEAFNKLDSRMR---------------------ETSGLKYWARESIL------
PLKTTIENNRPYILPRVAQKYDASNVETTAYGLL-VHVARQA-V---
------------IQREIVEWLNTQ--------RMSDGGWASTQ-----DTIIATQALI---QYSIQS-RLR-----DVTDVTITIEVPS----
TTGFVKQVHISEDNLAQLQTLEV--

---NLF-------------------------------------

>Mesobuthus_3 MMa54913

-----------------------MSVLISIIGIMILGI--------IIVILH--

---------------------------ISA---
------------N-----------------SILAQSNTGIQELNTYAVVAPVKLRPNFHYHVSVSIY----DVLS--DVDITVSVEGLT-------
--E-----AVSKTLILHA-----RETEIVEL--------EIGDWKPGIYNLSVSG------HG---GANFHNYTE---LIYEH-KS-
YSVFIQTDKAVYKPGQ-LVQFRAIVVDP-YLV--PSVSG------------TIDMHVR-----------DSQMNRIHQ----
WRDIPINR-G------IAAADF-------LLADEPNLGDWTVHVDV----K----------GQNFSKP----------------------
FTVAEYSLPT---------------FEVHIEL-PN--YA-TYN---ESDIVVTVKAMYT-YGKPV-
KGEVTLTVAPRTRYNYLSVRPYESTQV-----------------KTK---
-----------------------IDGYIDIQMN--LLQDLQ--LKTD--------FFEREIEFFALVEEEITSRKYNK-
TNTMWIYDKDVKVQLIKT---SDTFKPGLKYTAFLKVTH-QDGTLVT-----G-GN-RQLKLTYGYTHIERDW------------
------KSTYYTLPEN-----------------------------------GLLKLEFYPLHE--
ELNILYMRA----EFRG----------------QTHYIDGIDSAVSP-SN-NF------------IQV--------TPLTKTVRT-------
GSNIEFEVNATEPM-SH----------------------LTYQVMGR-------GSMVSARTIPVPNEKV------------------------------
------------------YRFSFRVSSQMAPKARVIVYYIRN----SNKE-IVADAVNFEV-EGVFRT----Q----VNVR----------
SNLIDVQPETTVDIRIETRPS-A--FIGILAMDKES----------------ILLKS--GNDISQN-----------------------------------D-
----VIKEL-ETYDGGKEHKYWPPWNQKRKKRSLWW----------------------LGSTS------AAEIF---------
DDSGVRVLTDG-------LIYRFPKENRFREN----------------------VIYIDDRTLNVPVQPPSTLP-------------------------
---DAPTVK-EHIVLRKTYPDTWLWMNAT-A-----GSDGV-AVISH-
-SVPKALTTWVISAFSMSSVHG-LGI-ENFPAEIKVSR---------------------------------
QFFIRMSLPYTVLKGESVAIQVVIFNY-NSR--SVKAEVTM-------------------V-NSKNEYEFTIAGGDKTEYQ------
NKKERSKYVT--------IPPNEGTPVSFLIT-----P-KKI--G-----YIDIRVVANTD-------------------
RASDAITKRLHVKLEGST--QYFNKAIFLDLASSGSEPIK---NNI--------------------SIPIPYN---SINGS-Q-RITISGI-----
--GDLLGV-SVN---N------MDHL-
LRLPYGCGEQNMLNFVSNVIITNYLTQSRRLT----NSI--RERAIRFLRFGYQRQLTYK-R--QDGSFSIFG-----QKDR---
SGSVWLTALVIKSFHSAR--------TL--I-DIDENVIENALKWLV-KNQNS-DGSFDEPGEIHHRPMQ--------EN---------
----KDALTAYVLISLLESG-A-----ES---N-YSRQIELA--ELYLTNAIYK-VR---DPYSVNIISYALHLL----
GNQIASDAFTKMSGLSK----------------KS---DKGDMIYWTNS-E-------ENINVTDKQS-DYFYLQ----
QSQDIEMTAYALL-VYTK-----------KSI---SDG-LPVLKWLISS-----
---QNEIGGFSSTQ-----DTIVALQALS---EMSSRM-ISS-----TT-SINAKFNYGD----ERSKNIRIN--DQNFMTLQQIDL-

PTDAH------YTEIEAT-GIGTGIIQVSWAFNLA---VSGE---TPAFFLNAL---LD--
---------------------------------KTSTESYLQLS-ICTHYK---E---GV-SNMAVMEVGLPSGYLADTDALPSIL----------------
----

---------------------------------------QIPRVKKVEANQGD-----TNVVIYFDRL-TREESCVTVPAHRTHKVAHQ-
RPVPVKVYDYYNLVKTARMFYHP-HKAT-LCDI-CDG------DDCRE-GC--
--------------------------------KKMEKKDYSK--LQG----------------------DETDKGSILTFNIMLIAGLTALTLTV------------
----

>Parasteatoda aug3.g11592.t1

------------------------------MILRWIFIL--------AWIPTL--

-----------------------------LCLIQDSYEESISP---
---------------QRIKH-----------------EP------------TYFVTASKTVRPGQVYSISVAVF----KSSS--PISVRASVQRD--------
-------GVELASATQ-ECRSGIPETLLLKI--------PPTS-LLGSYKLKVEG----DVHGVLSGTAFYNETE---LQYSQ-KS-
MTIFIQTDKPIYMQDQ-TVFFRAIPVTT-DMK--AFSDA--------------VDVYVL------------DPRGTIMQR----
WLSRQTNL-G------AVSLQY-------QISKQPRFGNWTIRVVAQ---G----------QVEEKS-----------------------
FFIEEYYQTP---------------FEVNVTL-PA--FI-MEN---EQVVRGLVTANYT-SGSPV-FGNLTVKARIES--------
LHTQYKDF--
--GQLPETKLFVNNVAVSF------------------------YDGSSLPAWRVES-
QKLELKTVVFFRTGAHQEL-------------------ETRHEQMSPV---------------------------------QFGIWEINIDLISVF-----
--------GTLSALN----------------------------DVHSMSIEA----KYVDHS-------GE------EVRTSLLAYASYTP-SN-RH-----
-------MQV--------STSTRNAKV-------GEYIIFHVRADYYV-EQ-----------------------FSYLILSK-------
GIVLFSGVEEMPA--SIKTFA--
----FTVT----------LNNKKDKTGETIEVIVEGQPG-T--YVGLSAVDHVL----------------HRMRT--GSDLSPA-------------
-----------------------E-----VMEKM-NAFD-EHRNGTL--SFSWQS----RT---------G-KSNRFVSFPTA-SYGID------

AYKIF---------EYAGLVVFTDA---
---HIPKQK-------------------------

---GDFIGSAFPH------
MPVNTSTL-LNKPDWCGEQNMFNFAANLYTLLYLRLSGQRQ----LEV--EKEAFKHLNVGYQRQLSYQ-K--
ADGSFSPFR--WD-TP------SSVWLTAFCARIFHKAT--FQEWE-NF--L-FIDPKVIGKAVSWLL-DHQSYE-
GAFFETSLNP-LDRKMNLTSG-RLK----DPVR-YR--NISLTAHVLITLAEMND-LTGDIGN---K-VARAKRSA--
QRYLEKMLHT-VKESKEPYEIAIVAYALTLV----NSVDGEVAFNFLDSKMK---------------------EVAGTRYWSKDIVL--
----PTLARVENNRPYVMPRLPHKYDSVNVETTAYALL-VHVKRQA-V---
------------------IQREIVQWLNTQ--------RSTANGWASTQ-----DSIIAMQALM---EYAIQS-RVR-----
DVMDVTVTVESPS----TVGFSKQLQIDEDNYSELQTVEI-PNAYG------AVIVKAQ-GSGLALVQLSVQYNVE-
WKHLMTPPPIPAFNLDVA--IYFWG-
RNNSHITYQSCQNWN---LLSESRMSGMAVLEVDLPTGYIIEQHELHGYV---

----RS---NRVRNLREGRFEE-----GKITFYFEYL-
DITPTCVKFTVQRWFPVANMTRYLPVKVYDYYAPERYNETMMDMYNLFVMSVCQVCGS---------------------------
---YQCPYCPVFSHS---
ITMQWNIFLTYSCFLL----SFYFLLKEVP---

>Parasteatoda_2 aug3.g14443.t1

---MT-----P---

-----------------SMK---S------INKL-LYMPTGCGEQNLITIIPHIIILDYLSQSNRLT----SDK--
KDQLISDLRLGYQRQLTYK-R--SDGSFSTFG-----ERDR---SGSTWLTAYALRALSLAN--------KY--I-
FIDSEVLNKALEWMI-SKQAP-DGSFDEPGEVHHKALQGG-----TEK-------------GAALTAYILMALFEAK-A-----QS--
-K-YNRQIALG--QNYIERELLS-SS---SPYVISMISYALHLL----GSSSKDRAFQLLLNMAE----------------RR---D--
DMMFWDNK-E-------AQVNMTDKQS-DYWFLA----ASIDIETAAYAIR-TFAL-----------KSD--------------------------
----------------------------------PTGSVPVLKWLISK--------QNKNGGFSSTQ-----DTVVALHALS---EISP-L-IPA-----FS-
SLNVKFSY-P----EGQKTLQIS----NALKMQEVEI-PSSTP------FVEIEVS-GSGVGIVQVSWSFNLA---VSGE---
APQFFLNAL---LD---KTSTASYLQLS-ICTHQR---
ERRNDT-SNMAVMEVTLPSGYIADVDALPSIL---

QIPKVKRVESQLHD-----TNVIIYFDRM-DAEESCVTVPAHRIHKVARQ-RRAPVKVYDFYIQARSARMFYRP-HRTD-
LCDI-CDD------DDCGD-NC--FKTTT---SS--
EES---------------------SFASSAHLLQSYSIFISLLSIFFLKAMM---

>Parasteatoda_3 aug3.g19014.t1

-------------------------------MNSLSFIV--------LICLLI---

--------------------------IPC---
-------------------------------------LKAQTFYLTTPSTLRLDSDETIAIAVE-------GKDGALVNVYIQDFP-----------
GKIKNITQTVLEVWPGRPEIFKINLNPAAFPADFFKTNPTSEKYVSLIVNS------------QQFQKEIQ---IPITN-KA-
GYVFIQTDKPIYTPKQKTAHIRIIPLNE-DAL--PSNKP--------------FKLQIR------------
NPQNVVVEETFFDRKSRRSQKV-------FISHIY-------KFPTFPVLGEWAATVNYG---H----------ELQQNST------------
---------VHFELQEYVLPT---------------FTVDLKA-PE--VI-LES---DETITASVQAKFV-YGKKV-
NGLVTFRLGVKQMMAARQVFFAVIGPKEL--
--------------------------DDGSYTLRLD--TKNLAH--HKDIGWFPE---
IEGSHLVVEATVVDNASGNKEVATDSRGIFAKSPFSISFKRC---LQDFKPGLVSVFEADINF-VDGRPAV-----G-
VPTILRAVAKSNKDNSKELKV-----------------TQTNAVSDDE------------------------------------GKVSFELQPEI---------
---HHETVSITI----ETDDPRYVGN-----------QAVGHFEQHKFKST-NE-AY----------
--VAI-------ERSSSQKLKV-------DDMFVKVVHFEPGE-IQNTYYTVDDMFVKVVHFEPGEIQNTYYTVSAK-------
GKIILQKPFQQGQVKV---QRVEFVITQDMVPNFRLTVFAH------YKDE-
LLVDSLNIDV-ENTCNK----N----AEVS--------IVPEFDVKEPGNNGKIVIRGTKA-T--LVGLLGVDEAV----------------
YALSK--KDLLTRS------------------------------------K-----VFKEL-AKHDLGCGP---
GGGID------SDKVL---------ANAGLIMASNV-----HNPSSTTSSCVAIKRR----------------------------
KREIGSEILSDYSGLAKECCSLGMSHDS--QNRDCETRTNIVVRQ----
LKGEHSNCSRAFLECCQKAKERGFTEMKAMKAGMHLARMGAIGIDEPNFVSLDEEEFFEKGLMVRSDFRETWFA
EDII-I-----GPDNQ-EVFDV--SLPHSITTWVIQAVSVSPNYG-ICV-A-EPQKIVSTK----------------------------------
KVFLQLNLPYSVVRNEQTEVQATLFNY-GTK--KLAAVVYM--------------------YGVKDVCSGANE---------------
GEKSERKRLI--------VDPQSAMTTTFPVI-----P-LKE--G-----MFNLKVVALSP--------------------
EGSDVILKTLNVIPEGYL--VEVDIPIKLDPTNQQRRAKRHITNDL-----FSDSIDTSQNLQVTAIKLPVPKD---FVPGT-
E-SCTITAL--GDQFGPTVETAINN------PDAL-
LTKPRGCGEQNMMFLAPTLYTMQYLRSVGKIT----PEI--EEKGYAFIRDGYGNQLSYR-K--DDGSYSAWN-----GK--
---PTSTWLTAFVMKVFCQAN--------KM--V-HIDEDVICSGVKWLV-NQQQP-DGSFVENNPVYHIEMTGG-----
VQG-------------SIPMTAFVLIALEEC---

--ECDIGRTEVN---

>Parasteatoda_4 aug3.g2288.t1

-----MHSF---------SRIIQS--

------------------------------------DCL--IIFFF--------ICTYVI--
----

--------------------------------YSTSQETRIFTAGEWDGTSDENV------YLSLSG--
-------------------------------RNFND-----------------DP------------RYLVIASNIVRPGQVYRVCVSIL----SSSN--
PLIVRASLHRD---------------GEQIVSATE-TVNPQHVSTLLMQV--------PFNA-IPGAYLFKVEG----
HLEGDLGGSGFSNETL---VDFSE-RF-LTILIQTNQLVYNLEQ-MMYIRIILLTT-ELK--PFTEP--------------IDVYIV------
------DSRGVIMKR----WVSIYPLL-G------VVNLEF-------ELPYDYPEGWWTIKAVAL---G----------QTEETR----------
--------------VLLERWFSHR---------------FDVNVGT-DA--FV-LNS---DEYIEGQILANYT-SVATV-TGNITLRTYLRP---
-----LGRYRDL------------------GL---NFKDRYVEEYVDL---------------------------------
---FHGTYDFSIS--MAQLRD--MAMPVP------LEHCEIEIQADVYERFYDFKVSA-YGRLRVVNSSVSLRFLGVN--
PQVFKPGMPFKTHLAISY-NDLVPFPEDKLET-ARVVVNPIVITRN-GRREL-------------------NQQILSFNSD----------
-------------------------GVVEVAI----------------------DTPK----------------------------NSERIIIRA----YYEDDL-----
EVGS------RARTELTVLAMHSR-KD-RY------------LQI--------STSTKQAKA-------GEYAVLHVWSNFYL-KS----------
-------------FHYLVVAK-------GIIVQTGTEKVMGMLH---
SITTFSVPVSPEMAPVFHVVVYHI-T----ADGE-VLTDSVIIPI-DALNKH----D----FKLV----------
VNMKQERSGNYIELIPFLTSE-S--NIGIWGFDSDH----------------VSTHG--RQQLTVT------------------------------------
--S-----INEAM-YEFEPDHLK-FH--RAFWRN----RD---------G-SPEKISYYTTH-NSARD------TKNSF---------
ENSNLLVFTNL-------IV-SYLP---Y--FCNTSTDLSSCK-----SG-
QCYPTNKK--CDGIRD----CEDGTDEGSCFQMQ-----LNEDKDLFEFYLYRRNRQGAFYDATAGNFAWKNKI-I-----
GEDQEEYIGT---TAPKGPSAYALNAVGMNKMYG-LHI-LPESIIFDSTK----------------------------------
PFFIIAEGPEEAVIGEQIGIRVSVMNY-QFI--EIKAEIIL-------------------MASDDYRFVQV---
EPMGIVSSYNPRTGSGELQHLIY--------VKPVSHAFVHIPIV-----A-TKI--G-----EIQVTIIGRTQ-------------------
VAKDVAEMTITISPDGVP--VHRHTSLLLDMRNEAYLIRYLDVNVT------------------EDPIIPYEETYRYYIFDS-P-
KASVSII---GDVVGAPFPE---
DPRSPIGLKALGMADAVKSGDFIMFDFAYTLLTLHYLRITNQLK----SQT--MRGMLEYLNKAYVYQSVFY-K---
NGAYTMFK-----DE-----QPSLWLTALCVRMFHLAQ--YPDWE-NY--L-YIDPDMLSRSVEYIL-RYQTRE-
GSFYEVYPQQ-WNRKMNVLSS-ND-----PYIN-YQ--NISLTAHVLITLTAVAD-LSGDIRV---D-ISNAKNSA--

VRYLERRLPQ-LD---DPYQVAIVTYALLEA----GSVESEIGFNKLDRLKR---------------------EKEGMVYWSPDDIV-----
-SSEILYQNQRPFILPRLPSKYDSVAVEATAYALL-VYVRYNG-I--
----------ITDQIVKWLNTM--------RTTDEGFISSQ-----DTIVATEALI---EYSFRT-HVR-----DITSMKVSVESSS----
NAGKIHQMSIMVDNLAESRRIEVAPRVWG------HAEIIAK-GSGLSVLQLDVGYNVD-RDFLLIQPPVPSFDLTVK---
--GYYHG-RNKSHMNIESCAKWT---
YTKESETSGVAVMEIALPTGYLIHKPDMDTYV---

---FS---
RKVPRLKRGRVYP-----KSAVFMFDYL-
DTSWLCVNFTVQRWYPVANLTRYLKAKVYDYYTPERYKETIYEDFDLFVLNICEVCGS----------------------------------
---YQCPYCPYFSFSA---ER----------
INMQWILLLTLASLVIIINNKRLSY---

>Parasteatoda_5 aug3.g25461.t2

---------------------------------MFKLFLRLQCLLLWMTMCF--

---AKRGFILTAPKLIDAGSTEYFTFTAF----DVPPGGVVSLKLLNYYTN----------
------TLLAESEVNVLNNMNMWVEMEV---------PPIPAVSKAKLQVQG-------NFSDGYRIKAEKE---VHIRH-SS-
VLTFVQTDKPIYKPGQ-IVKFRILPMDN-QLR--PLEPN------------TVGDIWIE------------DPSGIRVAQ----
WHKIEFKE-G------MVQLEL-------PLSNEPPLGLWNIKTVIN--------------EVTTAQR----------------------
FEVEKYVLPK---------------FEVKIKP-PS--II-MAD---AETIPVEICAWYT-FGKKV-
DGKLKTRVSYKRMSWDSKQIPSVEQSGL---
------------------------ISGCHNITVSTYSLQMHKGQ-----------YHRRALEFFAEVIENGTEIVVNE-
SITKNVMKSPLTIEFLGTED-ADYFKPGIPFYGQVQVRK-ADGEAAP-----S-

EKITICVYDRNMQRSHSGWSPYDREEDSN---------RCHRYTTNKK-------------------------------------
GRFRFSIAPQPS--SITKLTLVA----EAVKHESQHYEGMSYLKKIE-
KPQATLTLNPWYSP-SR-NY------------LQI------QSTPDEMECDK-------MHRVKVRYTGQKGA-SIK-------------------
----FYHKVISR-------GRIVQQGSHQRTFYSK---------
DESVNRRYDFALLTAGSNSRSYYPLRPPTVSNETSMPELGEFDLSFDPRATMSPVARLLIYYV-R----DDGE-
VVADSRKFRI-TKCLLN----K----VNLH----------FRHEQQYPSTEASLLLSATPS-S--VCGIGLVDKSV----------------
LLLKQ--EKQFNIE--------------------------------------K-----IFEIM-
KGFDVDKSTKPLQVSDDYCSKKADKPESLPDPPVFFDQEGRRRSDRRVRYLSRLESYHADTIMAF---------
EEAGMVAITDF-------ELEMRPCQNREPIAFELSEMNEKGIQMLARRNGRPRMRAYAGQSMPIRAELQVPSLKS---
--SDPQPEAPSVEEIRNYFPETWLWEMQT-V-
----GGSGE-TAMRR--KLPHTITQWTGKAVCVHPKTG-LGI-S-DDARITAFQ----------------------------------
PFFVGFRLPYSIVRGEILPLVVTVFNY-LTE--CLPIKLSL----------------------ESSEEYKI-----------------LSESGSHKVC---
-----VCGGQSAYHKFVLK-----P-TAL--G-----MVNVTVYGYSLEDDDEVCGNEITARL-----
SARDAVSKQLKVEAEGIR--KEEVTNYFICPKNTNGSYA----TEI---------------------DLIPPDD---VVEGS-A-RAFISIS--
--ADLMAP-ALN---S------LQEL-
VNLPVGCGEQNMILFTPNIFVLDYLTATDRKT----QDI--ENECIKNMKIGYQRELNYR-H--KDGSYSAFG-----ERDK-
--EGSLWLTSFVIRSFGQAT--------RF--I-DIDRQDIQVSVNWVL-ENQFE-NGCFQPIGTLHHKDMKGGL-TEGEHS-
------------LSPLTAYVLIAMIESGMV------R---N-DQGEAKNA----VKCLHEDP-NP---NLYALSLYAYACALMR---
QNETAIKYLKLLDDRAF----------------DV---G--GLRHWSSE------------------GKGLK--S----KSISVEIAGYYIL-
ARIELEN--------DES--TESVLPVVRWIAKQ--------
RNSKGGFISTQ-----DTIVALQALA---RYASIQ-TKG-----TV-DLGLAVEADN-----LVKGFKLN--EENKLVTQKMKI-
PEVPT------VVDLQAV-GEGCALVQFTFRYNVE---RAVG---SDAFDLNVD---VV---
---------------------------------------RMGSSECNLPR-IGICMR---YKIPDQTSNMVVVYVKMASGFTPDEESLNKLH-----
----

--SEINISLKRYEVEE-----NVVNLYFDQL-TNEQMCFTFYVRNELEVEDI-
KPATITIQDYYTPELQVTKEYSI-PSAC-SVPG-LPE------LPRLPPLV---
---------------------------ISSNDEISTS--ETDFDLF-----------
TSTNNPETVEVNTIPTTLEDGNDESAVTENIDNTTMTDFNNHTDGETTTFDISVE------------------
ESVTNQTPTDENGTMAENGTMAENGTMAESLNSLDVELADDSFSLLMNDNTSRLSNFIDVDHELDFPEGIEGV---
----
IPVTVVPPKLTSEKSTINCPICSERFPENFEEIYCGSAFVLKVRLVDANSVRIIQDVSPLLAKPITVKKFAALHYDKGCEC
PLLQRRGSFLLIMGDNDTLWVKEKDQEIHLKKITHVIATSSRTAEPNKIRNAKIACADDP

>Parasteatoda_6 aug3.g2883.t3

-----------------------------MLPEFVSFL--------LFIITS---

--------------------------VSCQSSYDMYPQFPGEFRL---
--------------------NKNKN-----------------EP------------TYFVMASKMVRPGQVYRVTVSVF----HTMF--
PITVRASIQRD---------------GVELATALQ-EVKQDMPETLLLKV--------PTTS-VQGSYNLRVEG----
NVNGVLGGTAFINETR---LTFSQ-RS-MTIFIQTDRPVYKQGQ-TVRFRALPITT-DLK--AFPDA-------------
VDLVML------------DPRRTIVRR----WLSRQTNL-V------TIAKDFEPEWTMAQLNISFFPNTHPIDTCHS---G---------
-QVEEHN-----------------------FLVEEYYQTR---------------FEVNVTM-PS--FF-TDT---EPFVYGTVEANYT-SGVPV-
IGNLTLVASIES--------LKS----T---------------SKR--VASIQQYWRR------------
----------------------FEGKTDFKFD--MREFSE--LVP--Q------LDKSKIVVTAHVGERYLDLIEKG-
FAEAIVFSSRIKIDFLGDD--PQIFKPGMPYKVFVAAYY-QDGSGIPPERLLK-QRMQITPIIEFYNGGSRRL--------------
-----STRFEKMSTL---------------------------------HPGVWEVGIDLENEF-------------ISKEDMK-------------------------
----NIKLMVLEG----YFFDDL-----T-GE------QAKAHLKVYASYSV-SN-RH------------LRI--------STSTRQPKV-------
GEYIIFHVRANYFV-ES----------------------FSYVVVSK-------GIILSAGQETM---TS--------------------------------------
----------SIKTFAISLSPQMAPTSTVVIYSV-A----REGE-VILDSLTFPV-DGIKRN----N----FTVT----------
LNNLKDKSLDTVEVVVFGRPS-S--YVGLAAIDKAL----------------YDMKG--GNEFSHV---------------------------------
---E-----VLRKM-SLFD-EGFNSTL--THVWLS----KE---------G-NLDSVVHYPAS-SYGID------AYSTL---------
QYAGLVVFTDA-------NV-TRRY---D--YCNESLGYLTCL-----
DG-SCYRYDKN--CDGFKD----CPDNTDESGCPEK--------DELRLKDFKMNRINRIQRLYD---NAWLWHDFN-I-----
GPLGYYIFNV---PVPEIPTRWMVSAFGMNNVDG-FGI-
QDKAIEVCSFKIASIXXXXXXXXXXXXXXXXXXXANSPDYKFVHVESFGRVDSYAPRTTSGEQQHLVWIKPGK-AVE--
VYMPIVPTRLXXXXXXXXXXXXXXXXXXANSPDYKFVHV---
ESFGRVDSYAPRTTSGEQQHLVWIKPGKAVEIKPGKAVEVYMPIV-----P-TRL--G-----DIDVTIMTKSQ-------------
------VAKDIITRRIHVEADGIP--QYRHTTVQLDLSQGAYLIKYLDLNIT------------------ETPIVPYRQE-RLYMFGS-N-
RAEVSVVGDVTGPAFPTMPVNASTLLRKPFWCGEQTMFNFAANLYNLLYLRLTGQRQPDIEKQAFKYLNMGDVT
GPAFPT------MPVNASTL-LRKPFWCGEQTMFNFAANLYNLLYLRLTGQRQ----PDI--
EKQAFKYLNMGYQRQLSYQ-L--SDGSFIPFR--WN-SR------PSVWLTAFCARIFHKAT--FQEWE-HF--L-
FIDPDVISRAVNWLL-DHQTPE-GAFYETASTL-FDRKMDSVLD-SSY----GGPR-YR--NISLTAHVLITLSELKD-
LQGDVGS---R-ISTARRQA--QKYLERALHL-VRDLPDPYELAISTYALTMA----NSPDANEALNFLDSRMR------------
---------EESGMRYWGRERVK------GQTVKIENTRPYIISRLPDKYDASSIEATGYGLL-VHVERNA-V--------------------
---IQREIVEWLNSQ--------RLTVGGWASTQ-----DSIVAMQALI---
EHAIQS-RIR-----DVVDVTVTIEVPS----VPGFVKQLHIGDDNLSKQQKFEI-DNAWG------VVLVRAQ-
GSGVALVQLSIQYNVD-WPHLQTPPPVEAFALQVR---
---------GYYYG-RNSSHIEISSCQRWT---YLEESEKSGMAVLEVSIPTGYVIQQQDLDTYV------------------------------
----

-------------------HS---GRVRNLREARYDE-----RKVSFYFNYL-
DQHSTCVSFKIQRWYPVANMTRWLPVRVYDYYAPERFNETMFDVYNLFVLSICQVCGS-----------------------------
---YQCPYCPIFSWS--------
SIVVSSPIILLASIFSWFILYRNMSI--

>Parasteatoda_7 aug3.g3268.t2

---------------------------------MNYTL--------LLCVIC---

--------------------------MIL--
------------------------------CVSFAQDSPSQDGYIFTSPRTLVIESDNELHLRRF----GKLDAGVLKVQVTYSDYS--------
---SQNDTLAAEKEFEIPAGETDSFLKLQL--------SKFENYVYNGRLIIEG-----TFG---EYKISGNDS---VYFSTPKT-
EITVIQTDKPLYKPGQ-TVKFRVLLLQK-DLK--PFGNEK-----------NNIEIHVE------------DPKATRLFQ----
FKDINLGS-G------LVQNEF-------PLSDEPVKGTWKIYAKRN---N----------QIIESTS-----------------------
FEVKEYVLPK---------------FDVSIKF-PS--FV-LVD---AETIPIEICAKYT-YGKPV-
TGTLNLNCSLEKYSYSREKLPVLQETFK---
--------------------LDGCYNYTLN--VSRIQY--GNS---------FRYKRIMVAANVVEDGTGVDRNE-
TQYLQRQQSPLSISFSNAQ--RQFYKPGLPYNGEIKVNQ-PDDTPAA-----N-VPIELCATITKERVLAEWWATKKLK---
----------YCKNYTSDKD-----------------------------------GIIQYTVLAQNM--
-DVVSIFLEA----KSVTFPSNGPHNTLN------VPSAAIYLDPYYSP-SE-SF------------IQL------QPVQQPVSCDS-------
KLKLKLLFTSKESD-NFK----------------------FYYQITKQ-------
SDVVSTNILETDFSVENDVSSRYESDEKILKGEETQLLPILQSSSSNAESDCPASKETKYLPPVGEVDFEIDVDAALSPV
FYVLVYYV-R----GDRE-TVADSQRIEV-QKCFKN----K----VKFA----------FGDEVQQPGMKTSVQITASPN-S--
LCGIKTIDKSI----------------SLLSN--NDQLTKE---
GYIDDSCRFVLIGLYSGRASSSILIPPGSGGT-----------------NSYDD------SLSAF---------QSSGFLVISDL-------
ILFTRPCMKGEGYG------SGGYGDGGMVNTVFQAEALSFDSAAGPAAMGLSAARRP--------------------------------
---AAAPFAAEAKIGLSATKSVSEVRTFFPETWLFEMEM-T-----GPDGT-YISKE--

KLPDTITQWIGSAVCISSEDG-LGI-S-NTTSIDAFQ-------------------------------------
AFFIDYTLPASVIRGEEFVLVVSIFSY-ADG--ALPQTTFK---
-----CFEVK-----G------G-----AILIVIHVSLK--
------------HLHSC---SSSSSFV--RNVLGK-AMD---N------
LDRL-VALPTGCGEQNMVKFTPNYLVLDFLKDVGMLS----EEI--ERRAKKNLMKG--

--------------------------------------NVLGKAMDNLD-----------------R--
--LVALP----
--TGCG---
---EQNMVK-FTPNYLVLDFLK-----

--DVGMLSEEIER-----RAK---------------------KNLMKGRN------------------
----

>Parasteatoda_8 aug3.g3269.t2

--------------------------------------MHCLFIFVAFFSFLAICF---

--SQDQDGYIFMSPRTLRIGANNEVIFRRF----GSLNAGTLKVQVFNKIDY----------
SSDNETIAFSKKFEISEGETEAILNVRF--------DLQDVHVFSGRIQING-----TFG---DQKISGSDT---VALHKPTR-
SIVIIQTDKPLYKPGE-TVQFRVLRVNK-DLK--PFKES------------NKTIAYVE-----------DPKGTRLFQ----YTNLDLEK-
G------LVQNKF-------QLTEDPVLGSWSIKVYTG---D----------KFNQLTN----------------------FDVKEYVLPK---------
------FEVTIKF-PS--FV-LAN---AKTIPITVCAEYT-YGEPA-SGTLNLNASLKRYIYERDVDEAKIPTKRITTE--------------

--IKGCYTYNLN--VKEIES--DTSY--------
YNSRSIVVAATVTEDGTGVERSA-SQNLNRDTEPLKLSFIDG---LDYYKPGLPYNGKLKVTN-PDGTIVS-----G-
EPIEICATINKRRILADWWATKTVQ------------TCRNYTSSSQ----------------------------------GLISYTILPQNV------
--DVISISLEA----KALNYDPATNKDVGNEPNQLSQPTTSKFLNPYFSP-SG-SF--
----------IQI------QPIPQPVACAS-------SQTLKVLFTSQESA-TYE----------------------FYYQVVYQ-------
STVVIDSSIQTSFSPDDDVSAKYEKQNKVINGPEVQLDPPKESIPQSSEDNCPEAQDSKYLPPIGEVSIDIDVNTIMSP
SIRVLVYYV-K----ESGE-VVADSMEIDV-ESCFEN----K----VQFA----------FGDKKQQPGAETTVSITAGSN-S--
LCGIKAIDKSV----------------LLLNN--NEQLTKE-----------------------------------R-----IFQML-
SRFDDNDYYSTNPCYQNPIQPGLQNAAVKSLSIAYPPGGNS--------YSYQD------SLSAF---------STSGYLVISNL-----
--YIFTRPCENAPRFGPGGAAAVAGPDGRAIKIALPMSAPGGALQSISEVRSNFPETWLF------------------------------
------------ELDMTKRKGILTLLSVSKVRCNFPETWLFELDMTKRRPSSSAGPAGPLQSISEVRSNFPETWLFELDM-T-
----NEDGT-FQKTV--TLPDTITDWVGSAVCINTADG-LGI-S-NTTTITGFQ--------------------------------
AFFIDYTLPVSVIRGEEFTMVVSISSY-ANA--SLPVTVTL---------------------EDPQGFKV-----------------
TDNSIDGDIC--------VHPQSSVTLPVTLT-----A-TKV--G-----KVQITVSAVSA-ESSDVCGSSPTSND-----
YARDAIRKPIEVKPEGFP--NENVINTLFCPSENEDNSFS---TSV--------------------SLKLPKD---VVPDS-T-RAYVDIT-
--GNVLGP-AIN---N------LENL-
VQLPTGCGEQNMVKFTPNYLVLDYLTDIGQVT----DDI--KNRAIRNLVAGYQRELAYR-H--PDGSFSAFG-----
TTDD---EGSMFLTAFVLRSFYEAK--------RY--I-YIDDNLITDMQKWIV-SKQDT-TGCFPNIGRIIDIDLQGGI--
PDENA-------------SGSITAYCLASLLITNY-----------N-NKTVVNDA----FSCLNDVP-PT---NPYSQFLYAYAYSLAG---
YKQDAEDLIEKARQKIN----------------RT---E--GMESFIIV------------------NG-----T----KAIEIETDAYAVL-TTLAVG-
--GS--------------------ASDALPYVRYLTSN--------LNPNGGFTNTQ-----
DTCVGLSALA---KYASVV-YED-----PL-NLMVKTTG-G-----LTKSVTLN--DQNKLLVQRFKV-TDISK------SIKIKAK-
GTGCGLIQTAILFNTK---TAPE---KKKFSISVR---GD--
-----CS-DPNCNTAK-IVTLVS---YLPPGQVAGMSIVQIGMITGFSPVRQSLEDLK----N----------------------------------
----

---DKN------------------------
---------------------NKILRIDVED-----NTVIVYFSEI-SNAVENFSFEVNQVVKVRKA-
QPGTAKVYDYYANENSASTSYKI-NSAN--

>Parasteatoda_9 aug3.g3270.t2

---------------------------------MKYLLYFGLIIAALLSDIA---

---------------GKQQDISTNCVDDDNDNGNIQDGSNNNPSGYIFTSPRSLKNGGNNELNLRRF----
GNLDGGNLQVQVFYRNDY----------NSENETVAASEDFEIPEGEEESILNLYL--------KISDDNYFNGRIQING-----
SFG---DYEISGSDS---VSFSKGKK-SIIIIQTDKPLYKPGE-TVKFRVLKVNK-FLK--PTEKN------------NSAEVYVE-------
-----DTKGTRLFQ----FENLDLQK-G------LVQRDF-------PLSDEPVLGRWSIKVKNG--------------DSEQSTN----------
-------------FEVKEYVLPK---------------FEVKIKF-PS--FV-LAN---AEIIP---
---LDGCYTYDLN--VTKIQS--
DQSY--------YNYRRIEVAAKVQEEGTGVEKTD-TQYLYRQTSPLSLSFIDS---LNYYKPGLPYNGKLKVSN-
PDNSVAA-----E-EPIEICVTINKKRILADWWATKTIK-------------VCRNYTSSEE------------------------------------
GLISYTILPQNV---DVISMNLDA----KSLKYAPDDTGGPNVLS----
QPQTSSYLSPFFSP-SS-SF------------IQI------QPIHQPVECGS-------KQTLKLLFTAKEDA-DFK----------------------
FYYQVSRQ-------
SKIVLEDYVETHFSSEKDVSSQYEDEDKIIDGDEVQLDPPKESVPKSSVDKCPEAQESRYLPPLGEINIAIDADITLSPTI
YVLVYYV-R----DDGE-VVADSMKIDI-ENCFKN----K----VDFA----------FGYDAQQPGAETPVTIKAGAN-S--
LCGIKAVDKSV----------------LLLDN--SDQLTKE------------------------------------K-----VFQMV-
ANFDSNQYYPSNPCYRSKVQPGLENAAVQKLFIPFPPVGSS--------SNYED------SLSAF---------QTSGLLVISDL-----
--YVFTRPCETGFRGG-------PGGRPILLQSKGNLHRKAFATTPGIIQADIISNVRIG--
--SGTSSLQSISDVRNNFPETWLFDLEM-T-----NEDGV-FEKDF--
KLPDTITKWVGSAVCVNSEDG-LGI-S-NTTSITAFQ----------------------------------AFFIDYTLPISVIRGEEF--
VKELCPY-TEG--ALPITVTL----------------------EDPEGFEV-----------------VGDSINGDIC--------VHPDSSVNLPITLK-
----A-TKV--G-----KVQIIVKAETA-QSSQVCGDSPTSDS-----YARDAIMKPIEVEAEGFF--
NDNVINTLFCPSDNDDNSFE---TSI---------------------TLSLPND---VVPDS-S-RAYMDFT-------------------------------
----------------------------------GNVLGP-AIN---N------LNNL-VSLPTGCGEQNMVKFTPNYLVLDYLQDIGMLT----
DDI--KNKAIRNLNTGYQRELRYR-H--NDGSFSAFG-----ETDR---EGSMFLTAFVLRSFYEAK--------RY--I-
YIDDNLIKQMQEWIV-SKQST-NGCFPNIGRIVDIGLQGGV--RDENS-------------SGSITAYVLASLLIANY-----------
N-NKTIINDA----FSCLKDYP-PT---NPYGEFLYAYAYSLDQ---NTPETKNFIDEARQKIN----------------RT---E--
GMESFIIV------------------NG-----T----KAIEIETVAYAVL-TTLGIG---GT----
-----------------ASEALPYVRYLTTN--------LNPNGGFSSTQ-----DTCIGLKALA---EFAKGV-YKD-----PL-DLSVTVSG-
G-----LVKNIKIS--DDNKLLVQRFKV-DDIST------TININAE-GTGCGLIQTDLRYNTR---VAPE---KKKFSLGVM---
GQ--CS-DSKCNTAT-IEVLTS---
YLPEGQVAGMSIVEVKLVTGFSPVKQSLENIS----
NKVEKISFDVNQVVEVKNAQPGTAKVYDYYANGIAQNEFKLSEEPSLGTWSINVSVANEFHTTEFAVDEFDPPTFE
VKLNLPSFILSTEENILIEVCAKYKYGLPVTGMLNFTAFSLHSLYAHYGLPKKPVPTMDIYTQIHGCYQVDLYVPLIEPG
NVYTYSKIQISAQVIEDGTGAKMEVKQYLERTTMPLILEFFDSLEYYKPGMPYNGKLKVLKPDNTPAENETIEICTWI
DKERSIAVWYAMREIEICSNFTSNLKGVILYTFLPQNLEDVSITLKAKSLAYLPQNSSDTTYRASSLEQPAATKHLKAYY
SPTNSYIQIQAFHQPVKCGTKQTLKVFYTADESSTDFQFFYQIIYQETVMLEDSVTESYKPQADIFKKFKNSNAVIKGE
EFQIEPSSAEIGNQSNAEFLNSSLYTPTKEIIADSMKIAVDKCFKNKVDFFFGDKKKEPGSDTDIYIKASPNSFCGIKAID

QSDTIVNSGDQITPDKLYQLRTSLFNDYYRPSNPCYSDIKQPGLDNNAFKNMTRPPIGKYGTPNYVDSFAAFQDSS
VLVISDLFLVTRPCKRNKKYPASYYDDEYINEDYFGQTPAVEYTYGDYGTEDYETSGSESDERMYSDTQIISGARTNP
ETWLFEMDLTGPDGAFITKKQLPDTITDWVGRAICLNLKDGLGISNDTYISSYKSFSIDYTLPFSIIRGEELTLVVTVLN
YESGALPVAITVDNPGFTKISSNKEVCVDPEGKTTFPIVLKASELGEVNVTVRAETTSTHGICGGSSISNTNAKDTVTK
PIQIMAEGYPVEKVISILAYPSISAKNVFQTKVTFTTPKNVVPGSDTGYVDFVGDILGPAINNLQNLVSLPTQGGELN
MINFVPSYLVLDYLTEIGLLTEDIKNRATNNLNTAYQREMKYRHDDGSFSTFGDADEEGSMFLTAFVLRSFYEAQDY
ILIEDSILRQMQDWIVSKQLDNGCFPNTGQIIDFDVQDGLTYGNSTGSTTAYVLASLVITEFDNISVINKAFKCLKENY
PADPHTQLMYSYTEALINKLKEAQMHMDDARRHLNKTGAAAYFAVANGTKSKEIEATAYAVLSTLEMGKDPTAA
FPYVNFLTKTLSPYGGFISIQDTCVGLEALTKFLEYVYGDSISLQIETTGGLQETVSLNDDNRFSIQRFKVSDTSSKINIL
ARGSGYGLLQAVHRYNTKVPPESTTFSIEVSGKCLDKNCKNGIIDVSVAYLPTNLQSGMSILEINLVTGFVPDTDSLYK
VKSNEDSKILKIDVEN-----NSVIFYFEEI-TNDKQNFSFQIQQIVEVNNR-KPGLAKVYAYYAKDNAASTSYTV-E-------
----

>Parasteatoda_10 aug3.g3271.t1

------------------------------------MTKPERER---YSTLNFPL------
--DDFESASNEGKLKING-----EFG---SYAFNGIDE---VKFDKPDD-SIVIIQTDKPLYQPGQ-T------------------------------
---G------IVQFEF-------TLSEEPVLGYWRIEVKIA-------------DSSYSTE--------
---------------FHVSEYGTFK-----------------------ED--

--------------TKYLSRTTHPLELEFFDG---LEYYKPGLPYNGKLKVSN-PDKTPAP-----N-
EPIEICAIIDKQRILAVWFAKRKIE-------------ICSNFTSNAR------------------------------------GIIAYTILPQNV----------
--DDVSISLEA----KSLKYHDSSSENRNLSLE---QPRTSKFLDPFYSP-SG-SF----------
--IQI------QPVHDPLPCGV-------KQTFKLLFTADGNS-EFK----------------------FYYQVIHE-------
ENIVVHNTTTKTFSMEKDVSSKYEMASKIIDGMEMQLDPPSSVVGHSAINDFPSV---
RYLPYIGEVDIDVEVDQNLSPQFFILVYYV-K----DNRE-IVADSIQVQV-DKCFKN----K----VNFS----------
FADKQKQPGLSTEIKISASPN-S--LCGIRAVDKSL----------------LLLDS--NDQLTKD------------------------------------
K-----VFDLRNDLFNT--YYFASSPCSDKRQPGLSNDAFTNITRPPAGVYGS--------SNYVD------SIAAF---------
QNPKLLVISDL-------FIATRPCKKNNGYD---------------FSVFDEFDDAEYITAEYATGEYLSGEFAS---------------------

---YEYDSGEAATQSVSDVRTDFPETWLFELEM-T-----GSDGI-
FKAKE--KLPDSITQWIGRAVCLNTEDG-LGI-S-DDTNITAFQ----------------------------------
AFFIDYTLPLSAIRGEEFDVIVSLSNY-VDG--ALPVTVTL----------------------DHPTGFTV-----------------
VNGSINENMC--------IQPKGKTSIHLTLK-----G-TQV--G-----KVNITVRAETT-SKSSVCGNSTVSKA-----
YARDAIRKPLEIEAEGFS--EEKVINSLICPADSSSNVYK---TSV---------------------ILKTPSD---LIPDS-A-RAYIDFD----
--GNILGP-AIN---N------LDNL-
VSLPTGCGEQNMVKFTPNYLVLDYLKHIGKLT----EDI--KTKVIRNLNTGYQRELTYR-H--GDGSFSAFG-----TSDE---
EGSMFLTAFVLRSFYESK--------KY--I-YIDDDVLKQMEDWIV-SKQRN-DGCFPNYGEIVHIDIEGGL--KEKKS-------
------NGSITAYVLTSLVISNS-----------T-NSTAINKA----FNCLQQNP-PT---NPYSQLLYAYAEALMD---
KKKVAENHINEARQRIS----------------RN---E--GVDSFIIV------------------NG-----T----KNIVIEADAYAVL-
STLAVGK--------
KSSEVVAYVRYLTNNFNPYGGFSSTQFSFIEGVDSFIIVNGTKNIVIEADAYAVLSTLAVGKKSSEVVAYVRYLTNN----
----FNPYGGFSSTQ-----DTCVGLHALS---QFAKLV-YKD-----PL-DLQIQTSG-G-----LKQMIKIN--
DKNKLLVQRYKV-SDISK------GINITAK-GSGCGL--

KSGMSIVEIKLITGFVPVKESINKVKLSNDNKVLRVDVENNKVILY---

--LDE--
VKLSNDNKVLRVDVEN-----NKVILYLDEV-SNKVQVINFQVEQLVKVTNA-KPGTAKVYDYYANENSSSTSYTI-
NGTS-TDVN-SS--

>Parasteatoda_11 aug3.g9168.t1

-------------------------------MLGVIWAL--------SALLAF---

-----------------------------TEG---
---------------Q-----------------SSTTVAP---LSSAVYTVIAPAKLRPNIPFHVSASVH----NLPA--PLDLKITIEGPA--------
-DNGQYNTIGKQITLNS-----GETQILNF--------EIGEWSSGNYSLIVEG------DG---GLKFRNQTV---LTYEH-KS-
YSVFIQTDKAIYKPGQ-LVQFRVLVVNP-HLL--PSVTG------------AINMYIT-----------DASGNRIKQ----
WGKVFTTK-G------IASAEL-------LLSDQPVLGDWTINVDI----L---------GQMFKKT---------------------
FTVAEYVLPN--------------FEVQIAL-PP--YV-TYN---KPDFVATVSAKYT-YGKPV-KGHVKLLVKPSLRFGYL-
ANENKPAVT----------------EAE--
IDGSVDIPIN--LARDLG--LKED--------TMALEIDVIAEVEEYLTKRKYNA-TNILKVYDRDIKVEMVKS---
SESFKPGLKYTAHLKVCY-QDDTPVV-----V-TNGDQIVLKYGYTYDERQW------------------DSRRIAVPNN---------
-------------------------GLLSVDFYPPVNT--NASSLSMNA----EFRG---------
--------FDYNLDNVEAAMSP-SN-SF------------IQV--------LLRTENPTV-------DKEVELEVNATEPL-SQ---------------
-------LVYEVLGR-------GDIVLAGAIDVPNVKT---
YRFSIPVTHKMAPKARIVVFYVRP----ENNE-IVADAVNFDV-SGTFRT----P----VSIN----------
TNVKETKPGAPVNVTVATKPN-A--LVGLLGIDQSV---------------LLLKS--GNDITQN---------------------------------
--D-----VITEL-ETYDGGKKRYQYGPYYRK--KRSLWW----------------------PGSAT------AHDVF---------
DDSGVVVLTNG-------LVYRYIQLIMYRSY-----------------------SGAIEDELNAVDTM----LY---------------------------
---DHGNGKGSKPRVRKHFPETWIWDLLP-A-----GNDGK-LSLVK--
NIPDTITSWVISAFAMDPVNG-LGI-APDTAKVTVFR---------------------------------
PFFVKLNLPYSVVRGESLYLEAIVFNY-NKK--PMKAVVTL--------------------E-NKNDDFEFTVAGNEIGE--------
AYDKMSKSVE--------VPAEDGVSVMFLIT-----P-KSL--G-----YIDIKVSAVAN-------------------
NAGDAVVRKLLVKPEGAP--QYFNKAILVDLRDTNSKKFE---ETV--------------------EISIPEE---AVPGS-G-
KVSLSAI--GDLLGP-TVN---N------LDKL-
LRMPQGCGEQNMLNFVPNIVITKYLKRVNRLT----PTI--QKKSLNFMESGYQRELTYR-R--NDGSFSAFG-----
NNDK---NGSTWLTAFVVRSFHQAK--------EF--M-EIDDGVMTSALEWLS-RQQKS-DGSFDEPGEIHHKAMQGG-
----SGS-------------KSALTAYVLLAFLENQ-A-----QK---T-FAQEMDKA--TKYLLRELKN-SQ---DPYFVSIVTYAFHLS--
--EHEEKDTALQKLLSLST----------------RG---V--DTIHWQKP---------------KDNS-MPYYEP----
QSQDVEMTAYALL-TYSV-----------RGD--
VAGALPILRWMISQ--------QNEYGGYSSTQ-----DTVVGIQALA---SLGVRL-ASA-----SI-SINISYNFND-----
MQKTLSIN--SDNAMVLQNVIL-PSDVR------SVALQAE-GFGVGLVQVTWSYNVA---VTDV---TPAFSLKPV---LG--
---KASTDDYMELD-ICTRYK---E-SG-A-
SNMAVMEVGLPSGFEADTEILPMIK--

--NLDKIKRVETNEGD-----
TNVVIYFDRI-DNEEVCVHVPAFRSHKVANP-KPVPVKVYDYYDLSKSARMFYEP-KSVT-LCEV-CEG------EDCGS-
KC--NELT--------------------------------
DGVNASSTPIYNLFLIALAAMFTFQIARANY--

Alignment of Dscams, for figure 7

>Daphnia DappuP127336

IENLWCGTRYQLYVRAYNCPMLYFVVEYLSPATWYNLRVTAHNNAGTVAEYEFAT

>Daphnia_2 DappuP331991

GALQVYASDGNPAVLPCPVEQAWAVEVTHWCFARHRLNMTCTVLAYPPAKIRWRITTGGDWFRIGQPAGDDA
GRYRCQAANSQGLRARLEPRQPAELTCDSGMYQCQASAELFNEQMVYPGYLQCSLASNSPPSIAWTVDSYLNISS
SEVEDGGLYCCSIIYSQEQHCARLNVGQDYYLDCPFAGYPVQSISWRDTDAKNGTLRISTMDNGEYTCLVKGSGSA
KKALFIEIRPPVIVQDSGRTQVTCSISSGDLPIKVSWTKDGSLLIFKKLRGEHGIYTCTARNLAKPPKWIIENYPASIPCQ
VHGMPTPIVRWFRDGSLRFSKMAKNDEGLFQCRAGNNIGNAVSKTIRIVRIHTSGAIFPTGLRCEASGLNISRVEAD
GGMYVCGSDATDIRLIVQELPRAPSRSAELQWLDEDPLLEYLHPFTVYEVQVNAINSAGIGPPSTHEEVPSGPVRHV
QAESKSSRSALVSWGRLVTFTVTLRPAIVYAVTVRALNRMGAGPESPSSSPADAIQLTWGFRIFYLDGLLCGTQYEL
QVQARNCRINSFQVEKFIEDETYHLKLTASSDSGHHASYTVRR

>Daphnia_3 DappuP43826

QSYETQATGGGVAVLRCPPAIKNDIQVTAWCRTQHRLHLPCLAQGYPVPEIRWESTASFGVLVLENVQPEDGATY
RCTAANSVGLRVQLRPGHTAQMECDQGIYQCQSSVRLFNEQTLVPGTLRCSAVGNPAPDISWLLDGHLNISSAQV
EDGGEYTCVAKNGQATHSSRLNVEADVSIKCPVAGYPIMEIAWLRSGGKTGVLIIKSVDKGVYTCTVRDRQGARRD
FTLDVVPPRMLKQGIRTRLLCGVSQGDKPLDFSWTKDGSALTFTNLSAVHGRYECAARNAAGPPFWSVEGQPLTV
HCQADGYPKPNVTWSWNGTLIIRSARKSLQGRYTCRAVNRIGQDLTATISIFRDSLSRLAVKRDLNCRVDGLTIAAT
QVDAGIYICGQVERDFLLTVLSRPPPPSRRVALSWSAPNPLIAYLQPSTSYQARVVAETSAGRGPPSTYGEPPGTCPS
DLRALPVSSSSIRLTWSRLIGYHLGLRKATKFLIVVRAFNRYGEGPLSPSAGPSQSIEVTWNYRLHYLDNLRCGTRYQ
VYLTSHNCPIRSFGVDFVQPSTAYNLKVRAKTSGGVAVEYDVVT

>Daphnia_4 DappuP48415

PPFEVTVENGGTAVFRCPDSVRDYVTVTSWCRVLVQPNGPLCNFGHDQNGARHRHRLIGGTLIIASVVPEDHGRY
VCSVNNSMGLHVRIIEAAQGQQQIDEGMYQCQATVQLFTRQILHPGSLKCLASGIPPPHFTWTLDAHLNISHVRV
EDGGNYKCVAENGRVEHSASLHIGKRLELPCPVAGYPIEGITWLPLNGRNGSLTIDPLDAGLYSCEARGQNGARQS
LQLNIAPPRIVMVNSRVQVSCVIEEGDPPFRIRWFRDDSILTIDQVTVNHGNYSCRASNAAGPPFWVNQGHSVEL
KCHVHGVPPPQVVWSFNQSLVISPVDVADAGFYQCEASNGVGNNINALMALVHLNQDYMAVRRGLKCSIRGLS
VTNAAADEGLYECGEDGDAVFLQVQDVPQPPGRRIQLEWKPPNPIQEFLQPATVYQVYMTASNSLGQSQPSTDE
EAPEGPPLQLGATSVTSNGFTLSWGLIQSYLVTLRPNTNYMAYVQAVNNQGTGPASPEEAPSQSLQITWGYRIFYI
GDLNCGTEYEFLLISSSCPIQQFVFRFLIPARDYAVRVTTTNAAGAQKDYTIRT

>Strigamia SMAR000414-PA

QPFDVYVYNGNTAVIRCPSFLTEYVEVLSWCQARHKLTIPCVSNGHPVPEEKWRYKIVQGALIIIDVQQYDAGKFIC
ESTNGAGLFATITPGHKTTFNCDKGMYQCHASARLFEEKTLRPKSILCEATGTPTPDISWTLDSWLNITSLKVENGG
VYTCHAKNGSVEYSTRIYVGDTVWLNCPIYGYPFDTLTWLPAHLRNGTLRLENVDEGRYICSVRNNNGARGYVDIN

IVPPKIVRQGSRARLQCVVSDGDTPMTIKWLKDGSILTINNVSSRHGKYTCSAKNGAAPPHWVIKGSGVIIDCMAE
GFPKPTIQWMKNGSLRIPYLSEHNEGYYFCHASNGIGDGLSKAMYLFIFKNNNKSFSISLICEATGLFISKAKKDSNTY
VCGSDETNFHVTILDVPDPPNGSNLVKWQIPSPITHFLKPYAFYDIRLAAANSIGYSNFSTEEQAPSGPPLNVEIEPV
DKQSLRITWGVIRGYRIGLDMYTQYMIIVQAYNGKGNGPPTPSVAPTTTVYIIWGYKVFYLENLACGAVYNLYMESI
NCPIKNFTIEYLTPATFYHVRVTAFSNGGTVADYITAT

>Strigamia_2 SMAR004329-PA

QPYDVYVYDENTAVMRCPTFLMSYVNVTAWCQTYNRLTLPCEVQANPTANTSWRHIQVSGSLIIGNVKSSDNGIY
RCTSYNSVGLAVATEPGKQTSIVCVRGVYQCCGSSAIFSTVVKNPGKLKCAAKGNPLPSFSWFLDTELTIKKSKTEDG
GLYKCVAENGSSFYWSRINIGANAILNCPVYGYPITDVQW--------------------------------------
VAPKLVQEGSRGRLQCVVVEGDTPLSIHWEKDGKSLSIESVLSIHGNYTCVA-----
PPKWIVEGADAYFHCKAEGYPVPKITWMFNGTLILKSVSKVKEGRYACLASNGVGHNLNMMVLLFTQKIQRLNN
NIDLVCSANGLIIRKTSRDSTVYICGKATIEMHLNIQEPPEVPNRSAFLTWEKPSQILHYLQPATSYSFRVAAVNAIGIS
KFSTDMDVPTGTVQNVEVEAIDGNTFRITWGIIRGYNIGLHKYTMYNVVIQVYNDKGVGPLTPSQAPSKSIYVLWG
YKVIYLNNLACGVNYHIYMEAFISQILHYLVQYLQPATSYSFRIAAVNAIGKFSNEITQT

>Strigamia_3 SMAR005038-PA

QAFDVHVYDGNTAVMRCPKFLHSFVNVTAWCQVYHRLTLSCVANGHPTPKYSWRVKILSGSLLIENSRVSDGGKY
MCIATNEINLNAVVTPNRAISLNCYAGIYQCSNAAVLFDQQEFQPGLLECVAEGSPIPQFKWSLDSAVKINSLKVED
GGIYRCIAESGSVEHFARIIIDGIVQIDCPVYGYPIKMIKW-------------------------------
GEIELFVVPPKIIREGARARIQCVVREGDTPLQINWFKDGNSLTIESVSSVHGNYTCVAGNSAAPLKWIAEGIRISIDC
LANGFPEPTVTWKRNGTLTFQNIQRYDDGFYVCLASNNIQQYLSKVISVFVEASIEIRTRKSIPCEATGLRIKWVDKD
STDFICGEAQLVAHLIVQENPNAPNQSATLRWTLNSNITRYLKPATVYNFRIIAESDIGNSSISTDETIPEGVPESIEVE
SIDANTLRITWGAIRGYNVGLLMYTQYDIIVQAYNSKGKGPISPSSAPSKSIYVLWGYKVMYLDDLMCGATYQLYM
EAFNCPVKSFSAEYLNPATWYILRLSVEGSGKTVAEYKFAT

>Strigamia_4 SMAR005057-PA

QIYDIHVYDKNTAIMRCPKFVQPYVNITAWCQVLHRLTLPCVASGHPTPDYIWRIEVGSGLLVIEKTQDTDSGRYM
CMASNEVNLFALISPNQEVFLNCNAGIYQCFGSAVIFNKQVLYPKFLECTSNGNPTPQFSWSLDSSLKISNVNVQD
GGVYRCIAKTGFVEHLARISIDSVVQIDCPVYGYPMNKVQWMPIHIRNGSLIIHNVDKGKYICLVTNDENVKGEIELL
VVPPKI----------------------------
NSLSIESVSSVHGNYTCIAGNNAAPLRWIIKGQILSVDCQAEGFPDPVITWKKNGTLMFKNIQRSDDGYYVCLASN
DNQESLSKFISI---

>Strigamia_5 SMAR005058-PA

QIYDIHVYDQNTAIMRCPKFVQPYVNITAWCQVLHRLTLPCVASGHPTPDYIWRIEVGSGLLVIEKTQNTDSGRYM
CMASNEVNLTALITPNEGVFLNCYAGIYQCFGSAVIFDEQILYPKLLECTSRGNPTPQFFWSLDSSLKISNVNIEDGG
VYRCIAKTGFVEHSARISIDGIVQIDCPVYGYPMNKIQWMPIHIRNGSLIIRDVDKGKYTCIVTNEDGVKGEIELLVVA
PKI---
PLRWIIEGQILSVDCQGEGFPDPTITWKKNGTLIFKNIQQSDDGYYVCLASNSNQQTLSKFISIFLQKSYEIFANKGLP
CEATGLHISWVDQDSTQFVCGENQINVTLVVQENPDAPNQIVKLNWTMGSEVTRYLKPATTYNFRVMAENIIGN
SSFSTDETVPNGVPENIDVESIDANTLRVTWGAIRGYNVGLHMYTQYDVIVQAYNGKGNGPRSPSNAPSKSIYVL
WGYKVVYLDNLICGATYQLYMEAFNCPVKTFSVEYLNPATWYVLRLSVDSSSKTLAEYKFAT

>Strigamia_6 SMAR005060-PA

QAFDVHVYDGNTAVMRCPKFLQRYVNVTAWCQVTHRLTLPCIANGHPPPTYLWNAKTMSGLLLIENVDESNSGT
YICTATNELKLKVIITPNQALSLKCLSGIYQCFASAVIFKAQVLNPGLLKCIAKGNPQPKLHWTLDSSLRISNVQVEDG
GIYRCIAKTGSVFYEARIDIDGTVKINCPVYGYPINSIQWLPQHIRNGTLQIKHIDSGKYLCSAIGNNEISGEVELKIVPP
KLIREGARARIQCVVREGESPIKISWFKDGNSLTIQSVSSAHGNYTCIASNQAAPLRWIVEGEKISLHCAAEGYPKPLI
TWKKNGTLEFESIRKSDGGLYICLANN-
VQNTISQVVSIFTRMNIDIRVKKGIPCEALGLRIDWVDRDSTEFICDESKQTINLLVQENPDIPNETVELTWKLPSEVT
RYLSPATTYNFRLMAENEIGNGSFSTEETVPSGPPENVDVESLDANTLRITWGSIRGYNIGLQMFTQYDVIVQAFNT
KGEGPESPSSAPSRSIYVLWGYKVVYLENLLCGATYQVYMEAFNCPVKTFSAEYLSPATWYTLRLSVESSSKILAEYRF
AT

>Strigamia_7 SMAR005787-PA

QRYEIHVYDGNTAVLKCPTHLAKYIDVIQWCRAKHRLTLPCVAQGYPVPNAIWRVQQTKSALIIENTQPADSATYV
CAVSNGIGLAVNVEPGKPAVFRCDRGMYQCQATAELFGERTLQPGSLQCISMGIPTPRITWSLDSVLNISDVQNPD
GGQYKCTARNGMAEHAARLNVGTNIKIPCPVVGHPITSITWLPVNLRNGTLVIEEVDSGTYTCLARNNQGAKSDV
DVAVVPPKILREGARARLQCVVSEGDLPLAIKWLKDGSVLSIDPLTPGHGNYTCYASNTAAPPKWIVEGNNVLLQC
AAEGVPEPTVTWLKNRSLYIEKASNHHRGHYVCQAANGIGRDLSTVVYLVERMQRNLTVQKGLKCVARGLIVVEA
DQDSTNFSCGSDHKRIGVIVIDVPAKINRSVRVKWNMPSTIKKYLLPAVSYSFRAIAVNDVGSSEASMAEEAPSGSP
QDVEVEALDTQSIRVTWGKLRGYKIGLKNFTKYSVIVQAYNGVGRGPASPRESPSDSILVSWGYKVYYFDELQCGTK
YRIFVSAYNCPIQNISLEF---------------------------

>Strigamia_8 SMAR005788-PA

MYVCVASNIIGLRAHIQPGKSTTIHCDAGMYQCQATAELFRDIVAQPGSLQCIASGSPAPLIDWLLESTLNILQAKVE
DGGLYICFAINGRVQHQARLNIGKDALLNCPFYGYPVDSIVWLPIDLRNGTLKLREVDNGNYTCIVSNKDGAKGDIY
IDVVPVKILTEGMRARLQCVVAEGDPPITLKWMKDNSMLTITNVTPRHGNYTCIAANSVSPPKWILNGQSASFHC
RAEGFPIPTIKWFRNGTLHIPSARTQHRGFFFV----------
KRVMEWAERKKRDVSGRVGLQCTIIGLTILNIIKDALSYVCGQDECAFRLKVLEPPFHPNGSVLAQWTEPSPITQYLK
PATKYEFRILAVNQLGRSEPGMEDEAPLVPPSMVKVDVIDEKTLRVSWGNINAYYVGLKTYTQYTVTVTAVNSGGI
GPSSPSKPPSQSIHLTWGFKVLYYENLYCGTIYQTYIIVHNCPIQHFVIEYLHKASEYQLRITAFNAAGSRAEYRVTT

>Strigamia_9 SMAR007303-PA

QPFEPHVYDGSTAAIKCSGIVTKYVIVDTWCIAKHRLTLPCVGQGHPSPIIKWNMKTTLLALIIRNVQDSYSGLYLCV
AENVAGLEIVVNTGGSQGLVCQSGIYQCHATAQ-------
HSGNLECKASSNPIATITWYLDSVLSLNKLTVENGGTYSCQAINGIDTYSARINVGDSFIINCPFYGYPISNIEWLPIMF
HNGTLIVSNVDDGKYSCIVSNIQSAVGTVEMVVIAPKILREGMRARLQCVVSEGDSPITIEWLKDNSSLSIDSVTSVH
GNYTCVVSNRAVLPKWVVKGSTALMPCIATGFPKPKTTWYKNGSLEIHNVNLKHAGYYFCLSNNTIGS-
ISAMALLIHKKLQNNTVRKGFKCVAHGFAIKKNRRHSTSFICGTENA--
NIQPTQTPDPPNRTVVIGWQTPMPVNKFLKPSANYNVRIIAGNDIGMSLPSMPDQAPAGPPLNVNAIATDSNSLK
VSWGIIKGYNIGLHKFTQYSVVVQAFNDGGKGPLSPTKPPSQSIHLTWGFKVNYLDGLHCGTKYQLYMVAYNCPIK
YFVVEYLSPATWYNLRIITHSSAGAAAEYTFAT

>Strigamia_10 SMAR008780-PA

LPCAAQGYPIPFYNWSIRQISGTIIITRAQVSDTATYVCVANNSVGLSAYIKPGKPANFTCDKGMYQCQATAELFHD
QTVQPGSLQCIVVGNPPPQISWTVHGSLNISHIRTEDGGEYKCSARNGGVEHSAKLNVGNLMYIKCPVSGYPIESIT
WLPVNHRNGTLMITNVDSGKYSCVARNSQGARRDLEVKVAPPKILSEGMRAQISCAVRQGEQPISIHWLKNGSIL
TIGHVARRHGNYTCTVSNAASPPQWITEGSRLQIDCSATGFPRPSITWKKNGTLSFVQATEADRGYYLCQASNGIG
AGLSKVVFFFDIKSKNQTVKKNLSCQATGMIIPQAERDTATYHCGQDEAKIHLIVQEAPDAPKRAMQITWTEPNLI
QRYLLPASIYNLRVLAENDIGLSQSSTSEEAPTGPPQKVKVDAVDSNTLKVSWGNIQGYYVGLLKFMRYGIVVQSFN
RLGPGPKSPDKPPAQSLHVGWGYKVLY---

>Strigamia_12 SMAR000616-PA

NGSLVIIHVTKESEGHFLCQASNGIGSGLSKVVSIFDARNVNQTVRKSLKCEAGGLVVHKTERDASTFSCGHDEKLID
LTVQEQPASPSRSVHLVWMSASPVLRHLLPGASYQMWVIAENALGKSKTSCLQEVPGGPPVGVEAKAVGSSTIKIT
WGELHGYYVGLLKFTKYSVVVQAYNKVGGGPDSPSEPPSQSIHVTWGYKLLYLNNLHCGSRYQVYLTAYNCRVLYF
VVQYLYPNTAYSVQITVHNDAGAVAEYDFIT

>Strigamia_13 SMAR009342-PA

QAFDVHVYDGNTAAMRCPKFLLRYVNVTAWCQVAHRLTLPCVANGNPPPKYIWRVKLVSGLLIIENAQISDSGNY
ICTADNEINLKVTTSPNQPMSISCFAGIYQCYGTSVLFEEKNYELGLLQCSAKGNPTPQFEWKLDSTFSVSNVKTDD
GGVYFCYANTGRVYHAARINVGDKMQLECPVYGHSIKSITW-------------------------------------
IPPKVIREGARARIQCVVREGQSPIKIIWLMNGNSLTIENVSSVHGNYTCVAGNRATPPSWVIEGRSLLLHCQAEGF
PLPIITWEKNGTLLFQNVQRSDEGLYICIANNGIQEPLSHVVSVFKQPNIELKVKKGISCEAKGLHISWVDRDSTVFTC
GVSVLTVTLLVQEAPDAPNQSIQLSWTMTSGITSYLKPATVYSFRVMAENQIGNSSFSTDENVPEGFPESVDVESID
ANTLRITWGAIRGYNIGLHMYTQYDIIVQAYNSKGIGPASPSSPPSKSIYVLWGYKVLYLDNLVCGSTYQLYMEAFN
CPVSSFSADY--------------------------

>Strigamia_14 SMAR009349-PA

QAFDVHVYDGNTAAMRCPKFLLRYVNVTAWCQVAHRLTLPCVANGNPPPKYIWRVKLVSGLLIIENAQISDSGNY
ICTADNEINLKVTTSPNQPMSISCLAGIYQCYGTSILFEEKTYELEILQCTAKGNPTPQFEWKLDSTFGISNIKTEDGGV
YFCYANTGRVYHAARINVGDVMQLECPVYGHPFKSIIWLPYNRRNGSLIIQNVDNGKYKCTTTGTNGVTEDIELIVV
PPKVIQEGARARIQCVVREGQSPIKIVWLKNGNSLTIENVSSVHGNYTCVAGNRAAPPSWVIKSNSLLLHCQAQGF
PLPIITWEKNGTLIFKNVQRSDEGLYICIANNGIQEPLSHVVSVFKQAELELKVQKGIPCVANGLHISWVDRDSTVFA
CGASVMTVTLLVQEAPDAPNQTIQLSWTVASRITSYLKAATVYSFRVMAENHVGNSSFSTDESVPEGFPESVDVES
IDANTLRITWGAIRGYNIGLHMFTQYDIIVQAYNAKGIGPASPSSPPSKSIYVLWGYKVLYLDNLVCGSTYQLYMEAF
NCPVSSFSADYLNPATWYILRLTVESGNKIVADYRFAT

>Strigamia_15 SMAR009501-PA

LEIVVNTGGSQGLVCQSGIYQCHATAQVFEERILHPGNLECKVSSNPVATITWYLDSVLSLNKLTVENGGTYSCQAI
NGIDTYSARINVGDSFIINCPFYGYPISNIEWLPVMFHNGTLIVSNVDDGKYSCIVSNIQSAVGTVEMVVIAPKILREG
MRARLQCVVSEGDSPITIQWLKDNNSLSIESVSSVHGNYTCVVSNRAVLPKWVVKGSSALMPCITTGFPKPKTTWY
KNGSLEIHNVNLKHAGYYFCLSNNTIGS-

ISAMALLIHKKLQNNTVQKGFKCVAHGFAIKKTERHSTSFICGTENATFNLLVLETPDPPNRTVVIGWQTPMPVNKF
LKPSANYNVRIIAGNDIGMSLPSMPDQAPAGPPLNVNAIATDSNSLKVSS---------
LHTFTQYSVVVQAFNDGGKGPLSPTKPPSQSIHLTWGFKINYLDGLHCGTKYQLYMVAYNCPIKYFVVEYLSPATW
YNLRIITHSSAGAAAEYTFAT

>Strigamia_16 SMAR009834-PA

QLYEVQVYDGNTAVMRCPSFVKDYVSVMFWCRTLHRLTLPCAAQGFPLPSYSWRILLMGGSLMLTSALILDAGTYI
CEVSNSMGLSAYIYPGQSATLTCDRGMYQCQGTAELFSDKVVQTGSLECAVSGSPTPQVVWTLDSLVNISQVGVA
DGGEYVCTASNGSVRHVGRINVGTDLVVRCYVSGFPIDSVHWLPFTIRNGTLLIQNVDEGQYTCAAKAGR-
DRKQTNISVAPPKILREGTRARIQCVLSEGDLPIAISWLKDSSSMSIERVFSVHGNYSCKAGNRAAPPRWLTQGGST
YLSCQVDGFPKPTVTWMKEGDLQVLGAEEADKGYYLCKASNGIGAGLSEVVYLFQTKTRNVTAKMGLVCEAYGLV
IEKVEKDSGVYPCGEDESHVQLIVQDVSDAPSRKIRLAWTAPSPINLYLEPAKSYHMRLYAKNEIGTSKASTNIEAPG
GPPLEVRVEAVDSTCLNVYWGKLTGYKIGLKKFTKYRVVVAAVNQMGDGPFSPSRSPSQGLSVSWGYKVLYLDDL
WCGTRYQLYIVAWNCPILYFVVEYLAPATKYVLRVTAHNSAGTIGVYEFVT

>Strigamia_17 SMAR009857-PA

QVYESQVYDGNTAVMRCPSFVADYVAVTKWCLTTHRLTVPCAAQGYPIPSYSWRVQVIGGMLLIRDAQNGDAA
VYVCVVNNSVGLDVIIEPGKAAVMKCDAGMYQCQGTAELFQDMVMHPGSLMCIASGIPPSLVTWALDSHLNLTS
TRSEDGGDYSCIAQNGSTRHTSRLNIGDRLMLKCPVAGYPIASIFWLPVDFRNGTLIVQNVDTGKYTCSVSSPDGAR
RDVEVSVFNAEILPEGTRARLQCVVSEGDVPLSIRWTKDSSILTINNVTPRQGRYTCTVSNEAAPPQWVVQGHSVR
LDCQADGFPKPIITWMKNGSLRLMSADENDRGQYFCLASNGIGAGLSKRVHLFDVKYRHQTIKRGLICQVRGLRIR
HVERDTATFSCGRDDTSIQLLILENPDMPSRSIKLSWTQPSPIINYLLPAHDYHFRVFAENEVGLSDPSTSEEVPSGPP
QDVKVQATGATSLLVTWGELLGYNVGLRKFAKYGVVVRAFNRVGHGTNSPALPPSQSLQVSWGFKVIYLFNLECG
TRYQMYVTARNCPILYFVVHHLQPATWYRLRITAHNSAGSVAEYDFAT

>Strigamia_18 SMAR009858-PA

QYYELQVYDENTGVLRCPSFVEDYVSVASWCKTLHRLTLPCAAQGFPTPSYRWRVRQVLSLLVLDKVHSEDAGVYR
CLVFNSMGLSVHVSPGKECRMSCDKGMYQCQGAAEIYPEKPMRSGTIKCVAFGNPPPEISWQVDSHLNLTRARL
EDGGEYRCIAKNGKREIAGRIQIGKSITIKCRVTGHPLDSISWLPTDHRNGTLHIHQVDQGIYTCIAQNRQGSRNWL
GLFVVAPTIVRDGMRARLQCVVTQGDLPIVITWLKDESTLTFQSVSPRHGRYRCIASSEVGMPKWVIQRGQVTLDC
QAEGYPEAIITWKKNGSLLLMAVTEADEGYYMCHASNGVGPGLSKVVYVFEEKKRNHTAKKGMECRATGMTILN
ANRDSGTYSCGHDQMITRLTVLESPEAPSRLVKLLWSAPSPIDKYLLPGTAYSFRVMAANELGIGKPSTTEEAPAGF
PQDVQVTALSSDSLKVTCGEILGYVVSLQKYTKYSVIVQAYNRMGRGPATPSKPPSQMLQVEWGYKVLYLDNLLC
GTRYHLNLAAFNCPVLYFVVEYLRPETWYAVRVTAHNEAGTIARFDVAT

>Strigamia_19 SMAR010628-PA

PVYDLQVYDGSSAVLRCSSLMTSVVNVTAWCQVRHRFTLPCAAQGFPLPEYTWRFTQISSILIIKSVMVSDTGIYVC
RAENNVDLKATLIPGSSLKLQCHKGMYQCQASTALFQEHTLQIGSIKCSFKGNPIPGVTWRLDTHRYMVELQTDD
GGLYTCEAANGKTSHWARLNIGGNVYLNCAYYGYPIDKILWLPVNLRNGTLVISNVDSGRYTCVASNKDGASQSLD
LAVVAPNILYAGVIARISCVVYQGDAPIQLLWLKDGSILTIPEVKPIHGDYTCVAKNLAAQAYWMVEGGSVHLHCLA
GGHPQPLITWLKNGTLYISHVDDQHNGYYFCEAQNGIGQGLSKVVRLFTNPLQRRIIQKGLECDPKGLSVDAALRD
SGSYVCGSADAIFEVTVQEPPDSPNRTLTLMWVAPNPLKRYLQPASDYEFRLFAINDRGASEASMAEEAPSGAPEE
VEVEAADGTTVNVFWGNIRGYYVGLQKFIQYAIVVQAFNGEGRGPLTPSKPPSYSINVTWGFK---
LYDLQCGTNYHLYVIAVNCPILNFVTEYLMPATWYVLRMSAFNNAGSVVEYTFAT

>Strigamia_20 SMAR010629-PA

QHYEIQVHDGNTAVFKCPKAMEGFVTVVSWCKTRNRLTLPCVTQGIPVPVTTWRLQQTSGALIISDTRLADSSAYI
CVANNTGGLSVKVQPGSSVTFTCDRGMYQCQGAAELFAEKIEYPGSLPCVATGSPPPHFKWTLDTSLNITDLVVED
GGLYSCHAINGSTEHGARLHIGERSIINCPSYGYPIEKYSWLPDNIRNGTLVISEVDSGRYTCIIRNND-
ARGDVEIVVVPPKILREGMRARLQCVVSEGDLPLSIKWVKDGNSLSIESVTSKHGNYTCIAHNVAGPPRWRIQGRE
VLLNCQADGFPKPKIKWMKNGSLHIKHSTETHRGQYFCIANNGVGGDLSKAVTVFHSKYQAQSAILGLVCSAKGL
QIMAVERDSGSYLCGADETTIELTVKESPEPPNQKAFLSWTAPSPIIRYLRPATTYNFRVLAENDIGVSQASTEEEAP
GGPPQAVNVEALDSQTLKVTWGVLRGYQVGLRKYSPYHIVVAAYNNKGRGPLSPSKPPSQTIYITWGYKVFYLESL
ACGTKYHLYLVAVSCPITALSVEYLLPATWYKLRMTANSSAGSTANYDFAT

>Strigamia_21 SMAR010632-PA

---MGV------------NAGFY--------
FEEKLQQPGSLRCVARGVPLPQITWFLDSILNVTHMRVEDGGVYKCESQSGVVQHFARINIGHDVRINCAMYGYPI
ESVDWIPLDLRNGTLLIGSVDSGRYRCSVRNKQGGTGEVEVVVVSPKILREGTQARVMCALIEGDPPVKFQWLKDS
SILSIGPAFPVHGNYTCVASNVASPPRWTLEGRSTVIHCQAEGFPPPSITWMKNGSLLVKHATESHRGYYFCAATN
GIGTGLSRGVFLIETKMQSFTVVEGARCEAKGLTVTNAQKDTRIFVCGGDMANIQIIVQGIPDAPNRSVELAWNPP
SRITKYLTPSVGYHFRMFSENTVGLSPPSMEDE---

>Strigamia_22 SMAR010689-PA

QMYEVQVYNGNTAVLKCPSFVTDYVKVMSWCRTIHRLTLPCAAQGYPAPTYSWRIKQIGGSLLIQNARIADGKTY
VCVVSSNVGLSAYIQPGSSAVLNCQRGMYQCQGTAELFQERVLSPGSLRCVASGNPAPQMMWMLDSYLNLTGV
RTQDGGDYTCLASNGNSSHTSRLNIGFDVTLKCRVYGHPLESLSWLPVNRRNGTLAIQNIDGGKYACVVRGVTGV
RKDMEITVVPPKILYEGMRAQVTCAVRQGDLPMAIHWMKDGSILTINDVRHRHGRYTCIANNAAAPPRWLVEG
ASARMDCQADGYPEPSITWTKNGSLILSSIREVDRGYYSCQANSKVGEGLSKVFFLFEVRSRNQTAKRGLQCNAKG
LIVTRSERDSGVYSCGRDSTVIHLTIQEPPEAPARSVNLAWLQPSQVAKYLLPATKYAFRVMAENEVGVSEPSTAEE
APSAPPTHILIEATQPQCLKVSWGEILGYNVGLLPFTKYDIVVQAYNRVGPGPLSPSRAPSQSVHVTWGYKLLFLNN
LWCGSRYQLYMSAANCAILYFIIEYLESRVSYNVRITAHNSAGQTEVYDFAT

>Strigamia_23 SMAR000686-PA

-----------------------------CQTYNRLSLPCEVQAHPSAEIKWHIQV-
SGSLIITDVGQYDYGTYRCTSINSVGLSVTFDYGKSTSFSCDKGIYQCCEATVLFESAVKTPGKLKCSADGNPLPTISW
FLDSDLTIDFIKTENGGLYKCVADNGKDFHWSRINVGDTAILNCPIYGYPIIEVRWLPNDFRNDSLIIHNIDRGIYNCF
VEGSDGLQGNIQLNVVAPKLVQEGSRGRLQCVVLEGDTPLKIEWQKDDNSLNIESVSSAHGNYTCIASNKAAPPK
WILEGNDAYFHCRAEGYPNPTITWMFNGTLIIKDVSKEKEGRYMCIASNGVGLSLSTTVFLFDRKEVKLNAQVGLIC
AAKGLIIEEVTRDSNKFECGTDSMNFIINVQEQPEAPNRSIFLSWSKSSPITHYLQPATSYTFRVAAVNTIGISKFSTDA
DVPTGTVQNIDVEAIDGNTLRITWGAIRGYNIGLRKYTMYTVVIQAYNDKGVGPLSPSIPPSKSIYVLWGYKVLY-----
----

>Strigamia_24 SMAR010692-PA

QIYEVQVYNGNTAVLKCPSFVTDYVKVVSWCRTVHRLTLPCAAQGHPPPSYSWRIQQIGGTLLIRNARIADSETYVC
VVSSNVGLTVYVVQPGGSVTLTCQRGMYQCQGTAELFKEHVLSPGSLRCIATGNPPPKMMWVLDSYLNLTGIRTED
GGEYSCVAVNGNATHAARINVGEDVFAKCRVYGHPLESITWLPINRRNDTLLILNVDSGKYMCVVRGSGGVRSTLE

ITVVPPKVLYEGMRAQVTCAVRQGDLPLTIKWLKDGSSLSIESVASEHGNYTCVASNMAAPPRWMLEGGSARMD
CEADGYPEPAITWMKNSSLLLTGVKESDQGYYLCQAANNIGEGLSKIFFIFDVRSQNQSAKRGLKCNAKGLIVTRSE
RDSGFYVCGRDTTTIHLAVQEPPEAPARSVHLIWSHASPVLKYLLPATKYSFRVMAENDVGVSEPSTAEEAPSAAPI
HIHIEATQPQCLIVSWGEILGYNVGLSPFTKYDVVVQAYNKIGSGPISPSRPPSQSIHVTWGYKVLYIDNLWCGSRY
QLYISAANCSILYFIVEYLEPRVSYNLRVTAHNSAGQTQVYDFTT

>Strigamia_25 SMAR010787-PA

QLYEVQVYDGNTGVLRCPSFVTDYVSVTSWCRTYHRLTLPCAAQGHPPPSYSWRLNNLGGSLLIQSGRIEDSGLYT
CVVNNSIGLTARLEPGRSATLRCDMGMYQCHATAQIFGPETIQPGSLMCIASGNPQPVMSWTLDGFLNITYTKPE
DGGDYKCVATNGKAQHSARLNVGGDSMIRCPYYGYPIDSVWWLPVNRRNGTIIIQNVDPGKYTCVIRNPQRARR
DVEVVVVPPMIFVEGMRTRATCYVSKGDLPITISWLKDESILTIDNVTHVHGNFTCVARNSVASPRWLVEGHRVVL
NCRAQGSPNPIITWMKNGSLVMSSATEADDGEYMCQASNGVGAGLSRVVRLFVTKIKNYTVKRGISCEVRGLVV
RKAERDNAVFSCGSNEAFIYLVVQQVPESPSRAVDLTWLPPNPILRYLKPDHSYHLRVRAENSVGLGAASTQEEAP
GGPPRHVQVVAVDSQTLKVTWGEILGYNIGLKKFTRYVLVVQAFNRHGNGPSS-------------------WIIFGA-----
LYMTAYSCPILYFIVEYLEPKSRYELRITAHNNAGTTVHYEFTT

>Strigamia_26 SMAR010788-PA

QLYEVQVYDGNTGVLRCPSFVTDYVSVTSWCRTYHRLTLPCAAQGHPPPSYSWRVKNLGGSLIIQSGRIEDSGLYTC
VVNNSIGLTARVDPGRSSTLRCDMGMYQCHATAQIFDPETIQPGSLMCVASGNPQPVMSWTLDGFLNITYTKPE
DGGDYKCVATNGKVEHSARLNVGGDSMIRCPYYGYPIDSVWWLPVNRRNGTIIIQNVDPGKYTCVIRNPQRARR
DVEVVVVPPVIFVEGMRTRATCYVSKGDLPITILWLKDDSILTIDNVTHAHGNFTCVAQNSVASPRWLVEGHPVVL
NCRAEGSPFPIITWMKNGSLVMSSATETNDGEYMCQASNGVGAGLSRVVRLFVEKNKNYTVKKGMSCQVRGLV
VRKAERDNAVFSCGSSEAFVYLTVQQVPESPSRSADMSWLPPNPITRYLKPDHSYHFRIRAENSVGLGTPSTQEEAP
GGPPRHVQVAPVDSQTLKVTWGEILGYNIGLKKFSRYVLVVQAFNRHGDGPSSPSKPASQSVKLSWGYKVVYLDH
LWCGTRYQLYMTAYSCSILYFIVEYLKPKNNYELRMTAHNNAGTTALYDFTT

>Strigamia_27 SMAR010961-PA

QAFDVHVYDGNTAVMRCLKFLHSFVNVTAWCQVYHRLT---------------
RVKILSDSLLIENSRVPDGGKYMCIATNEIN--------KTFECSCYA-----------
FDQQEFQPGLLECVAEGSPIPQFKWSLDSAVKINSLRVEDGGIYRCIAESGSVEHFARIIIRRNSPNRFSVYGYPIKMI
KWLPFYIRNGSLIIENI-----------------------
IPPKIIRERARARIQCVVREGDTPLQIDWFKDGNSLTIESVSSVHGNYTCVAGNSAACSR-------------------------------
----------VYRKLHTNRLKFLDKFYVMI--

>Strigamia_28 SMAR010994-PA

--MPS----------

NHSFLIEGVRKMDEGYYLCQATNGIGSGVSKPVFLFSVKQKTQAVRKGLHCDVSGLMIMHAERDAVTYSCGQDEA
NIQLLVQEPPGPPSRVIKLGWTPGSTIATYLLPGLTYHFRVMAENNLGRSEPTTPEEAPGGPPLAIDVKPTVSASLRV
TWGEILGYYVGLRKFTKYSVVVQAYNRMGSGPYSPSEAPSQSLQVGWGYKLFYLPNLFCGSRYQFYITAFNCAILYY
VVEYLVPGTWYTLRVMAHNDAGTLTLLDFAT

>Strigamia_29 SMAR011182-PA

QPFEPHVYDGSTAAIKCSGTVSKYVIVDTWCIAKHRLTLPCVGQGHPSPIIKWNMKTTLLALIIRNVQDSYSGLYLCV
AENVAGLEIVVNTGGSQGLVC--
GTETCHATAQVFEEKILQPGNLECKASSNPIATITWYLDSVLSLNKLTVENGGTYSCQASNGIDTYSARINVGDTFIIT
CPFYGYPYNNMRLLPIMLHNGTLIVGNVDDGKYSCVVSNIQSAVGTVEMVVLAPKILREGMRARLQCVVSEGDSP
ITIEWLKDNSSLSIESVSSVHGNYTCVVSNRAVLPKWVIQGSTALMPCLSTGFPKPTTTWYKNGSLEIHNVNLKHAG
YYFCLSNNTIGS-
ISAMALLIHKKLQNNTVQKGFKCVAHGFAIKKTERHSTSFICGTENATFNLLVLETPDPPNRTVVIGWQTPMPVNKF
LKPSANYNVRVIAGNDIGMSLPSMPDQAPAGPPLNVNAIATDSNSLKVSWGIIKGYNVGLHKFTQYSVVVQAFND
GGKGPLSPTKPPSQSIHLTWGFEINYLDGLHCGTKYQLYMVAYNCSIKYFVVEYLSPATWYNLRIITHSSAGAAAEYT
FAT

>Strigamia_30 SMAR011319-PA

LSAAFDYGKSASFTCDKGIYQCCGSTALFRSVVKTPGKLKCISGGNPLPAFSWFLDSELSIASIKTENGGLYKCVATNG
KDFYWSRINIGDNAILNCPVFGYPISDLKWLPNEIHNDSLVIQNIDSGIYSCFAKGSDGVEGNVQLEVVAPKLVQEG
SRGRLQCVVVEGDLPLKMQWQKDGNSLSIESVSSTHGNYTCIASNEAAPPKWILEINDAYFHCTATGYPTPAITW
MFNGTLIIKNVSKDKEGRYICIASNGVGQGLSTTVLLFNKKDNTLNAEVGLICTPNGLNIVEVTRDSNKFECGSDSMF
FFLNVQEPPEAPNRSIFLSWSKSSQITHFLQPATSYTFRVAAVNAIGTSKLSTDGDVPTGAVLNIDVEAIDGNTLRIT
WGAIRGYNIGLRKYTMYTVVIQAYNDKGVGPLSPSISPSKSIYVLWGYKVLY---

>Strigamia_31 SMAR011548-PA

QSFDVHVYDGNTAIMRCPKFLLSYVNVTAWCQVTHRLSLPCVANGNPAPKYIWRRKLMSGLLVIENARTSDMGT
YICTADNNINLKVMMSPNQAISISCLVGVFQCY-----
FEAQTYEPGMLQCTAKGNPTPQFQWKLDSSLEISSLKVEDGGVYFCFAKTGQVFHAARINVGESVQLECPVYGYPI
HEIKWLPFNRRNNSLIIYNVDNGKYTCKITGNNGIKGSVELTVVPPKVIREGARARIQCVIREGQSPIKITWLKDGNSL
TIEYVSSTHGNYTCVASNRAA---

>Strigamia_32 SMAR011561-PA

QAYDAQVYDENTAVLKCPSFVADYVTVTSWCRTLHWLSLACAAQGHPAPSYSWRLHQVHGLLIIDHVQPEDAGT
FVCSASNSLGLSVHIEPGRMATFTCDSGMYQCQSTAELFKSHTLNPGSLRCVAAGIPVPKVIWNVDSHVNISRVQV
EDGGLFACTASNGNTTHTAPIAVGEDLRMRCPVSGHPIDSIYWLPVNHRNGTILVQSVDAGKYTCVASNNQGARR
DFEVAVVPPKITYEGVRASVFCSSSQGDLPLNIKWYKDNSTLVIELVKAHHGNYTCSASNAAAPPRWHVEGSAVQI
ECQAEGHPPPLISWFKQGSLRISSALEYDEGHYMCKATNNVGAGLSKVVFLIDVKLKNESVKMGLRCNVHGLKIM
NVVRDSTMYKCGEDEASVLLIVQELPEAPSRAVHISWSRPIPVTGYLKPATKYRFRVFATNELGMSESSTGEEAPSG
PPKDVRVEAMNSQTLRITWGEILGYHVGLKKFTKYSIVVQAFNDHGIGPNSPSSSPSQSLHIQWGYKVLYLQSLTCG
TRYQVYVTPVNCPILYFVVEYLAPAEAYQLRVTAHSSAGNFAQYDFMT

>Strigamia_33 SMAR011562-PA

TKLFMEVGENVGTFDLC-------
FRIISWCRTVNHLTIPCAVQGLPIPSHSWRIVKINGLLVIQNTQVSDAGIYLCKVSNIIGLSAYIEPGQQIRFTCDHGM
YQCQGSAQFISSEPLQPGKLQCTAVGSPVPQISWYLNSILSIISVDLQDGGYYKCVGQNGLDQVVDRLNIGKDLQLI
CPYLGYPVTSITWLPTDRRNGILRIEEVDEGDYTCFVRNSNESQQTRRVYVVPPVILQVGSRTRLQCVVTEGDLPITIT
WLKDGSVVTFSSITIDHGNYTCIASNKVAPPKWIVQQGSVTLDCAAEGSPLPTITWKQNGSLHIRDVSKKDERYFLC
EVTNGIGPPL------
FELKSQNKTSKRGMDCRAYGLVITYLDKDGGVYECGKDVAQIHVLIREPPEAPSRNVHLHWNPPYPIKRYLLPGTGY
SFRILAENDLGYGQASTAEEEPSGAPRNVRAESIGSGTLLVTWGELLGYNIGLEKYSNYSIKVQAYNRAGHGPASPSE
SPSQRLNLSWGYKTFYLSNLQCGKHYQVFLTSYNCPIRNFMIQYLQPSTQYTVLVTVHNDAGTDATYNVFT

>Strigamia_34 SMAR000919-PA

QPYDVYVYDENTAVMRCPTFLMSYVNVTAWCQTYNRLTLPCEVQANPTANTSWHIQV-
SGSLIIENVKSSDNGIYRCISYNSVGLAVATEPGKQTSIVCDRGVYQCCGSSAIFSTVVKNPGKLKCAAKGNPLPSFL
WFLDTELTIKKSKTEDGGLYKCVAENGSSFYWTRINIGANAILNCPVYGYPIKDVQWLPQDMRNDSLTISDVDSGIY
ICSVQ-------------
IAPKLVQEGSRGRLQCVVVEGDTPLSIHWDKDGKSLSIESVLSIHGNYTCVASNKAAPPKWIVEGEDAYFHCKADGY
PVPKVSWMFNGTLILKSVSKVKEGRYVCLASNGVGRNLNMIVLLFTKKSQQFNENIDLVCAANG--------
DSTKYICGNDTIELELNVQEPPEVPKLVCTINNCAIDVPC--
LQPATSYSFRVAAVNAIGISKFSTDMDVPTGTVQNVEVEAIDGNTFRITWGIIRGYNIGLHKYTMYNVVIQVYNDK
GVGPLTPSQAPSKSIYVLWGYKVIYLNNLACGVNYHIYMEAFNCTVQTFQVEYLIPATWYDLRVTAITNGDIPAEYK
FAT

>Strigamia_35 SMAR011671-PA

QYFEVQVYDGNTAVLKCPSFVRDYVMVTSWCETLHRLTLPCAAQGYPVPSHRWRVRHMGGFLLLERVEADDAG
VYRCVVSNSVGLTVHVQPGKPATFTCDRGMYQCQGTAQLFTSKTVQQGSLRCVASGHPPPRFTWTLDSFVNVTN
VRVDDGGNYRCMAANGEVNYIGRLNVGSNMTINCHVSGHPIESIKWLPYNRRNGSLVIRNLDQGIFTCVARGPG
GVRRQVNVKVVPPLIVQEGIRARLTCVVTQGDLPITITWVKDGSSLTFSGIKSHQGAYSCIAKNPIAPPRWLMQQES
FLFDCKADGHPVPIITWKKNGSLLLQNVDERDEGYYLCEASNGVGAGLSKVVYLFDHKSANRSLRRGLACLARGLIIP
KVDRDAAQYTCGKDETHISMLVEEQPEVPSRTAVIAWSAPNPLLFFLYPGWSYTFRVLAENGVGLSAPSTTEEAPD
GSPVHMEAKALGPEAVKITWGEILGYYIGLRKFTKYSVLVQAFNGMGSGPHSPSGAPSTSLAVTWGYKVFYLPDLH
CGTRYQTQVAAFNCPVRHFVIQYLISGIMYHLHITAHNDAGTIAEFEFAT

>Strigamia_36 SMAR011672-PA

KFYEVQVYDGNTAVLRCPSFVVDYVSVTSWCRTVHRLSLPCAGQGYPIPVYSWRIRPAGGNLLLDNVRPSDSATYV
CFVNNTVGLSASIQPGKPASFQCDRGMYQCQASMELFSEKIVQAGSLECIASASPAPLVTWTLDSILNVSRVQVQD
GGEYACAAKNGKIVHSARINVGEDVVIKCPYAGHPIESIQWLPIDHRNGTLLVRSVDAGTYTCLVRSPDGATQDLEV
MVVPPTILHIGMRVRVVCVVSQGDVPMTITWLKDGHVLTISKAHSEHGNYTCIAHNDAAPPRWLTEGGRVLIDC
QADGFPVPRVTWMKNGSLLVTEARPDDRGRYSCRASNGVGTGLSHVIVLFESKTRNMTIRQGLVCSAIGLSILESS
GDTDTFRCGTDETLIYLTITERPSPPARFARITWTTSSPIHQYLQPDASYQCRVWAMNAAGPSQPSTTEEAPGGPP
RNVQVEPTDARAVKVSWGRIRGYYVGLRQFTKYSFIVQAYNQAGTSSRSPNQAPSQSLHVSWGYKVVYLSSLECG
TRYQMYIQAMNCPILYFVVQYLVPATWYALRVTAHNNAGTVAEFHFAT

>Strigamia_37 SMAR011726-PA

QVYDVHVYDGNTGVLKCPSVLSDFVRVISW--------
IPCVAHGNPAPKFKWRAYQTASSLVLVDPQVSDGGIYICEAQNSVALSAKIEPKQAAIFKCSRGMYQCQASGELFS
DKVLQPGSLQCVAVGSPPPSVTWTLDSTFNVSNTRTEDGGLYRCIVKNGIVEYMARVNIGNDIWVDCPMYGYPID
NITWLPFDLRNGTIKITNVDSGRYTCIVNNKHGAKEDKQLVVVPPKIFHLGMRAHLTCAVSEGDLPVRFQWLKDGS
VLSIGSVEAKHGNYTCIASNDAGPPWWVIQGGSILINCSADGFPKPVISWTKNGSLWIKQALIEHKGRYFCQATNGI
GGGLSTPINIFDIKYRNQTVRKGVPCEARGLHVMAADRDTAVFTCGRDEANINIIVQEPPDFPSRDLILEWITPSRIT
KYLSPATSYHFRVLAENMLGTSALGMAEEAPAGPPESAKVEAVNPQTLKVSWGKLRGYNIGLQKFTQYGVAVQAY
NDAGKGPLSPSKPPSQSIHVTWGFKIFYLTNLECGTRYHLYLFAFNCPIQYFVVEYLIPAMRYDLRMTAHNTAGSVA
AYKFAT

>Strigamia_38 SMAR011727-PD

QPYDIQVYDKTTAVLKCPSFLEEHVRVTMWCHVKHSLTLPCVAQGHSAPKAEWRVQQTSGALIIHRAQTSDSGTY
VCDVSNEAGLTVSILPGKSATFRCDKGMYQCQGAGQLFDDNTLESGSLACVATGSPAPVITWFVDSVLNVTETRW
EDGGTYRCLANNGTVEHIARLNIGEDVRLNCFYYGYPVTSINWLPFNLRNGTVVIRKVDSGKYTCTVVNDKGAQEH
VEMAVVPPKIVREGMRARLQCVASEGDAPLYLRWTKDGSILSISHVTPRHGNYTCIATNEAAPPRWTLEGHSLQLD
CQANGFPEPKVTWMKNGSLLFIRAREYHAGHYFCKASNGIGDGLSTAVHVFDVKFVNQSLKRGLECPPNGLTVEE
ATRDTGIYNCGTDDTNLQITVFEPPDAPSRNVHVTWSHPTRIIKYLRPATHYQLRMFAENELGLSSPGTKEEAPAGA
PVDVEADAVDANTVVVRWGKLRGYTITLEKFTKYSLTISAFNDQGQGPKSPSEPPSQSIYITWGYKVFFLSGLKCGS
HYQMYMTAFNCPVSTFKVEYLTPGTAYLLRVTALNGAGSVATYDFIT

>Strigamia_39 SMAR012077-PA

---SAFS-----------------------

VAPKILREGMRARLQCVVSEGDAPITIEWLKDNSSLSIDSVSSVHGNYTCVVSNRAVLPKWVIKGGSTVMPCVTTG
FPKPTTIWYK--

>Strigamia_40 SMAR001994-PA

CTAKHRLTLSCVGQGHPPPAIKWRMKTTPLALIIRVVKETDSGVYVCLAENIAGMEITLNATEKQNLVCKSGIYQCH
AIAQVFEEKILNPGHLECEATSNPIAKITWSLDSELILKKVTVENGGVYRCAATNGSDSFGARVNIGENFIVTCPYFGY
PVSSLKWLPIMFHNGSLSVSNVHASDCISILSNRTF---------
VAPKILREGMRARLQCVVSEGDSPITIQWLKDNNSLSIESVSSVHGNYTCVVSNRAVLPKWVVKGRPAVMSCLTTG
FPKPITTWYKNGSLKIDHVNLQHAGYYFCLSNNTIGS-
ISAMALLIHKKFQNNTVRKNFKCVAHGFAINKAERDSTTFVCGMENATFNLLKL--
PMRRNRTVVVGWSEPSPINKYLKPSIDYHVRVIAANDIGLGLPSMPD---

>Strigamia_41 SMAR012080-PA

---MKLK---------------------

HKKVQNDRVKNGFKCIAHGFAIKQAGRDSTNFICGSENATFNLLVLEKPDAPNRTVVIGWTAPNPINKYLKPSANY

HVRIMAGNDIGLGLPSMPDQAPAGPPLNVNAIATDSNSLKVSWGIVKGYYVGLHKFTQYSVVVQAFNDGGKGPL
SPTKPPSQSIHLTWGFKVTYLDGLHCGTKYQLYMIAYN--------------------------------------

>Strigamia_42 SMAR012325-PA

QPFEPHVYDGSTAAIKCSGTVSKYVIVDTWCTAKHRLTLSCVGQGHPPPAIKWRMKTTPLALIIRVVKETDSGVYVC
LAENIAGMEITLNATEKQNLVCKSGIYQCHAIAQVFEEKILNPGHLECEATSNPIAKITWSLDSELILKKVTVENGGVY
RCAATNGSDSFGARVNIGDNFTMTCPYFGYPVSSIKWLPIMFHNGSLSVSNVDDGKYSCIVSNIQGAVGTVDILVV
APKILREGMRARLQCVVSEGDSPITIQWLKDNSSLSIDSVTSVHGNYTCVVSNRAVLPKWIVKGSPAEMTCLTTGFP
KPTTTCSS---

>Strigamia_43 SMAR012326-PA

---------------------------------------VHAIA---GGKRYLIT---ITKKYFIS--
FEEKILNPGHLECEATSNPIAKITWSLDSELILKKVTVENGGVYRCAATNGSDSFGARVNIGDNFTMTCPYFGYPVSS
TKWLPIMFHNGSLSVSNVDDGKYSCIVSNIQGAVGTVDILVIAPKILREGMRARLQCVVSEGDSPITIQWLKDNNSL
SIESVSSVHGNYTCVVSNRAV--

>Strigamia_45 SMAR013455-PA

PPKWVIEGKTLFLHCQAEGFPLPIITWEKNGTLVFKNIQRLDEGLYICIANNDIQEPLSHVVSVFELPNLEIKAKKGITC
QASGLHISWVDRDSTEFSCGKSVMKANLIVQEEPDAP--

>Strigamia_46 SMAR014023-PA

PPKVIREGARARIQCVIREGQSPIKITWLKDGNSLTIENVSSAHGNYTCVASNRAA---------------------------------------
----

>Strigamia_47 SMAR015611-PB

QQYEAHVYDGNTAVLKCPQFIGSHVVISAWCQTTNTLTLPCIAQGFPVPELEWQFSQLKISMTIQQIETSDAGIYVC
VAKNSVGLSALVEPNKMATLKCESGMYQCQDVAQVFEEKILEPGKIECVVEGNPLPSINWFIDSVLEISNLKTADGG
IYSCHFKNGAVKHASRINVGTSVQIDCPFYGYPINQVKWIPFEPRNGSLIIAKLDDGKYLCLVNNGKKEKQDVEMVV
MPPKIIREGMRARLQCVISEGDTPIKIEWLKDNSSLSIENVAAEHGNYTCIVSNHAGPPKWIQTGEEVKLPCATEGF
PVPLVTWTKNGTLHIFSVQEKDKGFYFCEAQNGIGE-
LGAMLQIMIKQYQALTPHKNLSCKAYGLKIHHINHHSATYQCGSDEASFHLSVKDVPKPPNRTVSVSVSWSLQSPTKR
HLLPSQKYHIRVLAENEVGFSEPSVADEAPDGTPNDITVMAIDTNTLKISWGIIRGYYVGLVKFTHYSIVVQAFNDG

GKGPMSPSKAPSQSIHITWGYKVFYLENLRCGTQYQLYMLAVNCLIKYFVIEYLKPATWYDLRMIAHSNAGAVAEF
TFAT

>Strigamia_48 SMAR002919-PA

---LYVSPGSNQLQ--

GAIRGYNIGLNMYTQYDIVVQAYNAKGLGPMSPNSPPSRSIYVLWGYKIQYLENLICGATYQLYMEAFN------------
----

>Strigamia_49 SMAR015640-PA

--APKI---------------------------
SILKIEHIQISDGNYTCVAINSAAPPKWVIEGKTLFLHCQAEGFPLPIISWEKNGTLTFLNIQRSDEGLYICIANNGIQEP
LSHVVSVFAQPKLEIKAKKSIVCEATGLHISWVDRDSTEFSCGKSVMKANLIVQEEPDAPNQTAELSWTLASAVNRY
LKPATIYSFRVLAENQIGNSSFSTEESVPEGPPENVDVESMDANTLRVTWGAIRGYNIG-------------------------------
----

>Strigamia_50 SMAR003721-PA

QIYEAQVYDGNTAVMKCPSFVADYVSVTSWCRTVHQLTLPCTAQAYPVPTYSWRMVRAGGNLLIRNVQLKDAG
VYICLVQNPVGLTAYMDSNQAATFRCNRGMYQCQAAAELSSEETTYPGSLKCVASGSPLPVISWTLDGHLNITEVS
LEDGGTYACVATNGVVISSTRINVGTTLIVHCPYAGYPIDSLLWLPVSHLNGTLKVLSVDAGIYTCIARNKQGARREM
EVMVVPPKILHEGAHARIICAVVEGDLPITFAWLKDGSVLTINRVTSRHGNYTCSAQNPAGPPRWMVQGSLVVID
CQASGFPVPRIEWLKNGSLVLGGARVEHEGRYLCRATNGVGNGLSKMITVFDVHFRNETVQKGLHCMVRGLSISR
SHRDTALFTCGNDESRIQLVIIEPPDAPARSLKLSWTRPNQITHFLKPATNYHCRVLAVNEAGSSEPSTLEEAPSGTPL
SVRAEATGADVVKVTWGNILGYYIGLRHFTKYGVVVQAYNHMGTSPRSPSLPPSQSFQVMWGYKVVYLNHLECG
NRYQIYIAAVNCPILYFVVEYLELGMWFNLRVMAHNSAGTMAEYEFAT

>Strigamia_51 SMAR004310-PA

QPYQIFVTKGNTGVIRCPQFLQEFVEVTAWCKMRHKLSIPCIAEGHPVPNVQWRILLTLEGMLIKNVNSNDRKIFV
CVASNSIGLKVSMIPGQTLSFACHKGIYQCQAKAELFMEQTLKPNSLQCSASGTPMPEISWSLDSFLNISSIKTDQG
GVYKCTAKNGQLEHMSRINVGENVYLNCPVVGYPINSLIWLPFDLQNKTLVISKADAGRYVCIVSNKQGAESSVLLS
IVPPKILSEGVRAQLHCVVSEGDLPLTIIWTKDESILSISNVTAFHGNYTCIAANNAAPPRWQIMHDSISVPCRANGF
PPPEIKWTKDGTLQINEAKLEDQGFYFCSANNQIGKGLSRVIYIFENEIVELKVKINLFCEVFGLSILEVSKDSGEYYCG
SAKTIIKLIVQEKPLKPNRSVEVFW-
KPDLIYKYLQPATAYEFRIAAENEVGSSNYSLNEEAPEGPPQQVEVEATDRDSLRISWGPIRGYYIGLKIYTKYSIVVM
AYNNGGSGPTTPRSPPSHSVYVVWGYKVFYLQNLTCGATYFIYVEAYNCLLQYFTVKYLESGNVYNLRLTAHTKGEK
VADYQVVT

>Strigamia_52 SMAR004313-PA

QPYEAHVYDGNTAILKCPPFIGSHVIVSAWCETTNTLTLPCIVQGFPVPELEWRIFKSENSIKIKQIQTN--
GIYVCEAKNSIGLSVRIEPNKVVNLKCESGMYQCHDTAQVFEEKIMEPGKLECEAIANPRPSAKWFIDSVLEIPNIKSE

DGGVYTCQFVNGVTKHSNRINVGRSVQINCPYYGYPIKQIKWLPTDPRNGSLWITEMDDGVYTCVVKNENTATQ
DVEIVVISPKILREGMRARLQCVVSEGDTPLKIQWLKDNSSLSIENVAAEHGNYTCFVSNRAAPPKWIQSGADLQLP
CATEGFPKPIVTWTKNGTLHIFVVKKEDKGFYFCEAKNGIGR-
LGGTVKVVLMKVQNITSHKGLQCETTGLTILNTNHDSNAYQCGEDEAVFHLYVKDVPIAPNRTMTITWSLQS---
QYLLPSQKYEVRVLAENEVGLSDPSIAEEAPGAAPSDISVIAIDTNTLKVSWGIIRGYYIGLLKYSQYSVVIQAFNDGG
KGPLSPTKAPSQSVHITWGYKVLFLDNLNCGTKYQLYMIAVNCPIKYFVLEYLKPATWYNLRMIAHSNAGAVAEYLF
AT

>Metaseiulus gi_PIPE_391326293_PIPE_ref_PIPE_XP_003737652.1_PIPE_

-----------------PG-----------
CRTRHALSLTCVAQAFPTPVYRWRVLQADGSLYFVKTKIQDSGVYVCVVNNTAGLQAHMTPGRSATLTCERGVY
QCQATSEIFKERIIDPGSLHCIASGTPLPQVTWTLDSHVNITDVRVSDGGYYACIARSGEKVHEARINIGETVRIQCPY
SGYPISDIFWLPTNRRNGTLVLTTSDEGLYRCTARNKDGDSGTLRVKVVKPVLIQEGMKVVVTCSVADGDAPFEVT
WLRNGATLTIEKASPRHGDYTCTARNAFAPPRWSIEGRSATLDCQAEGYPHPQLRWEKNGSLTIQDVTKSDAGFY
LCQATNDVGPGLSSVISLFKTKFHTNMVRKDLTCEVKGLTLSRVGRDSALYSCGTDDTNIQLIVQEPPDPPSRHVRL
TWEPPSLITQYLRPSSSYDLVVIAENGVGESQPSTEGEVPEGPPEQINVEATSSTSLRVSWGEILGYYVGLRKWTRYS
VSVQAYNKKGPGPRSPSRPPSSKIKVTWGFKVRYLEGLQCGTKYQVYLVGVRCPMRAYVINYLNPATWYEIKVTAS
NDAGQVHIYAVAT

>Metaseiulus_2 gi_PIPE_391334253_PIPE_ref_PIPE_XP_003741520.1_PIPE_

RSFTAKVFEGNTGVLRCSDADRMYIQVKTWCVAQHSLTLSCIAQAVPPPTYQWRISQHDGTLYIHQVTTEDAGTY
QCNANNTMGLSAMITPGGSVYLNCHKGVYQCQANSILFEEITTGPGSLKCIASGRPLPQVTWTLDSFVNISSVRPQ
DGGMYHCTARSGEVSHSQRLNVSETMVLICPAGGWPIDTITWLPYNHRNGTLMVHDVDEGRYTCTAKNSQGAS
NGVFIRVVKPVLLHQGQRYNVLCTVSKGDSPIHIRWYKDESTLIFNELQPHHGNYTCEARNDAGPPSWRVEGTTAF
LDCQADGFPQPKIRWTRNGTLIIQSAKNSDSGYYLCQAANGVSPVLSKVVRLFRWKFRAETAQKGMQCEAFGIVIT
DTDRDSALFSCGSDDTNIQLILQEPPDPPSRKIRLSWTAPSVIVKYLLPATMYVVRVLAVNAIGVSEVGTHNEAPEGP
PLNVRLEAVGPHSVRVAWGELRGFYVGLRRNTEYTMVVQAFNDKGSGPTSVPESPKSSITLAWIGYNVYLGGLEC
GQKYFLYMRAENCPIESFQIKYLVPSMPYLLLVRATNSAGTEAQYDFVT

>Metaseiulus_3 gi_PIPE_391337343_PIPE_ref_PIPE_XP_003743029.1_PIPE_

HKFSASVFDGNSALLRCPANVRDFVRVSSWCTLHNVLTLPCISQGHPSPKTTWRHHILDGLMVISAATPGDSGRYV
CVVNNSAGLIARVEPGRTANMSCDRGMYQCQGTTQLFRELTLKPGSLKCSASGNPLPQITWTLDSYVNITAVRVE
DGGVYSCIAKNGRAEHSARLQVGEAVRIHCPAAGYPLKSIHWLPQNHRNGTLVIADVDDGQYTCVVTGEHNVSK
QLKLTVIPPIVLTQGKRAGAACIVSDGDLPIRISWFKDGSFLSFTSVDQLHGNYTCVATNPAAPPKWLTEGEPIVFDC
QASGHPLPNIRWKKNGSLSIREVSREDAGPYMCQAVNGVGPGISKVINLFERKFQALTVRRGLSCRAIGIHVGSAD
RDSSLFSCGRDDTNFQVVVQEAPEKPSRSATLTWQHPSPILRYLKPKTEYEIRLKAENALGMSEASTDEEAPSSVPR
NIKISSTGSSSLHVAWASVQGYYVGLKKSTKYVVVVQAFNSKGAGPVSPPPAASSSVHLAWGYIVFYVRNLSCGRA
YTFYILAHNCTISTFVLHYLVPATWYTLSVAAHSEAGTETEYPFST

>Metaseiulus_4 gi_PIPE_391343779_PIPE_ref_PIPE_XP_003746183.1_PIPE_

QYFEVQVYDGNTAVLRCPSFVRDYIVVTGWCVTKHLLTLPCAAQAFPAPKYAWRYLLKGGSLLLQKVRSSDAGKYV
CIVSNPVGLRVALVPGKSAVLNCDGGMYQCQGTAQLFKSVTLKVATLKCSATGSPAPSLNWFLDSYVNISRAQVE
DSGMWTCEATNGSVLFSDRVNVGKDVVLHCRVTGYPIESIVWLPLHHRNGTLIIFSVDAGQYSCFARGNDNATAS
LFMIVVPPLILYEGMRTRVYCNIARGDQPISIKWLKNGLALVIDSLNPAHGNYTCMASNDAAPPHWVREGHSIHID

CMATGHPMPRIIWQRNGTLRISIVRYQDRGPYLCQAANGVGSGLSTVVQLIEHAVHNNTVKLNLNCPVRGLIVHK
VVRDSGTFTCGNDEMQVRLYVQEPPEPVSRSVNVVWREPSPLIYYLSPDTIYYFRISAVNEIGVGAPSTEEEVPAGP
PTNVYVQAVGPNSLRVSWGKIRGYYVGLRKFTKYSILLQAYNSIGAGPRSPQLAPTNSIKVSWGYQVIYLMGLYCGT
QYHLYLVAHNCPISHLTIEYLKPETWYSVRVTATNDAGTVAEFPVRT

>Tetranychus tetur07g00600.1

MTFQANVYDGNIGLFKCPNFLRDHVKVSSWCQVHNSITLPCVAHGFPVPHYRWDLLTGTGILIINNVSSSDSGRYI
CVANNSAGLSVQLELGSQLRLHCDIGCYSCESTISSFSELITSPGSLKCVASGNPLPTVTWTLDSYVNITSLRSEDGGLY
QCDAHNGSAHHSQMIYLGQQVKIRCPVSGHPITRIIWLPINDRNGSLIIKEADEGFYSCKASNDQGDEKRFYIQVRK
PSILTEKMRVIITCNVLTGDPPISVDWLKDNSSLVFRDVKQKHGSYTCLATNQVGPPKWIIEGERVTFDCQADGFP
MPIIRWKINGSLTIKETDKPDSAYYLCEAENGIGSVLSKVVSLFKSTFQVMRVKIGISCEAFGLITPVSTRDSGSFLCGQ
SEMTSRVLVEEPPDPPSKSISLRYSLPSPVTKYLKPLTNYDIRMFAVNNIGKSEPSTDEEAPLTSPVSVRAEAISSTSILV
KWGSVTGYYIGLRKSTSYIFTVQAFNTKGAGPQSAPLSPASTVTISWGFKVFLIRSLHCGTKYTIYMVSFNCPINNFIV
QYLQPATWYNLLIAAQSDAGTEAEYFFAT

>Tetranychus_2 tetur17g02480.1

PHIVMEVMDGNTAVFKCPNFIKDYLIISAWCRARHKLTLPCVAQGYPVPHYNWRMTRLDGIVFIQNVKIQDSGIYV
CQVNNTIGLSAKLVPGSSLFLNCHRGIYQCHASSQIFKSQTLDPGSLKCVAAGNPLPQITWTLDSYVNISDVRVEDG
GEYKCKADNSSIEHGQRVNIGDLLLINCPVAGYPIESITWLPDNHRNGSLEIHQVDEGFYACLARNKQNSESSLYISV
VRPTILREGQRASVICTAASGDYPIHVKWLKDNSILLFESLALEHGNYTCIATNEAGPPRWIIEGRSISIDCQAEGYPIP
RIRWTKNGSLAIHNAQKSDAGYYLCQTNNNIGAGLSKVIKLFETKFNVVTVTKGLQCKSIGILIRDADRDSALFTCGK
DETNIQLLLQEPPDPPGRSATVSWSPPSPIIDYLHPLTHYQLRMYATNAIGRSEPSTDEEAPGGPPLHIKAIPMSSSS
VRVTWGPIMGYYVGLTRATKYSITVQAFNSKGSGPASPPSTPSNSITITWTGYIVYVEGLSCGSSYQFYIVAFDCPIDK
MEIMYLRPATSYELKIIATNEAGTTVSHTFKT

>Tetranychus_3 tetur17g02500.1

FDEAVLEEGSLNCVAYGNPLPQVTWLLDSYVNITSVLAQDGGLYECTASNDTVRHSRRINIEESLIINCPIGGYPIETIF
WLPYNHRNGTLIINDVDQGKYTCGARNKEGAKGSFYVSVVRPFILQSGQRYNALCSVTRGDPPIEIRWLKDGSHLT
FESLGPEHGNYTCIATNSAGPPRWHIEDQLAIIDCQADGFPVPQTHWSRNGSLAIHNAQKSDAGYYLCQTSNNIGS
GLSKVIKLFETKFNVHTVRKGVQCKAIGILIRSADRDSALFTCGKDETNVQLLLQEPPDAPGRSATITWSPPSPIMEYL
HPVTHYQLRMFATNAIGRSEASTDEEAPGGPPLHIKAIPISSSSIRVTWGVIKGYYVGLTRATKYFVTVQAFNSKGSG
PASPPVSPSYSITISWTGYIIHFEGLSCGTSYQFYIIAFNCPMNKIEIMYLRPATNYDLKITATNEAGIQAAYSFKT

>Tetranychus_4 tetur19g01590.1

----------------KDFVGQFVYYL--DSRVRFCHFHMNK-AFVSVDHGQVGV-------
----

FTEKDVKPGSLKCMSTGNPLPQITWKLDSFVNISSVSVEIGGTYSCTASSSTIENVAPLRVHRSLSVKCPVGGYPIVNI
FWLPNNHRNGTLVVSDIDEGYYSCTASNDEGSSNGFHLTIVAPKVIQMGQRLSISCTVIKGDPPFHIEWLKDGSTILF
KAIKGEHGNYTCLATNAVGPPQWIYEGDQVWSNCQASGFPMPSIIWKRSGALFIDDIQKSDAGLYLCQASNGVG
TELSKVIRIFTSKFRSETFTKLLSCEAYGLAILDVDRDSALYSCGKDETRIQLIVRESPDPPSRSVKVNWAPSSPIQRYLV
PLTNYTICLTAVNEIGQSEPSTDEEVPNKAPDHLKGYALSSRSIKVAWGKINGYYVGLERKTKYEIVVQAFNSKGPGIL
SPPKPPFTSITLEWGFIIRALHGLFCGTFYEITIRAFNCTIKHFSIKYLHPQTWYHLKSTAYSDAGTDAVYSLMT

>Tetranychus_5 tetur19g02050.1

--------------MFCPSLTNAAIYT-----------TLLIS----------VHSLTESDLPLISPPLSSLPLISSISSSSLISSPSSSP--------------
-
VTTTATFDELIVDPGSLKCEATGSPMPTITWKQDSYVNISSISVPDGGFYTCVAANASASHSARIHVGSTVIVNCPYS
GYPIDSIFWLPSNRRNGSLVIHDVDDGSYTCQASNGIKSAQKINIIIAGPMIIQEGMRARLVCTVIQGDPPFVFQWF
KNDSDLTFARVSPRHGNYTCIVSNSVAPPYWKVEGQNIMIDCLAEGSPQPIITWERNGSLLIKTVEPDDGGLYLCQA
SNSIGPGLSKVVTLFVTKFRSETVRLSLSCDARGLRIESARRDSALFTCGKDETNIRLIVQEPPESPSREAKLTWSQSSEI
NRFLRPATTYVCSVKSENDVGLSKSSTEEQAPEGPPLNLKAKPNDSRSVKVTWGKLKGYYIGLKPFTSYDIIVQVYNS
MGTGPRSPSKEPSESISVTWGYKVIYLDNLLCGTLYKLFMTDLTCPITKMLIEYLTPKSTYSIRITAQTRGSTMAEYDV
RL

>Tetranychus_6 tetur21g02250.1

------------------------------------VNLISQGYSVNDVRQ------------
SSKCPNCSNDTNSSNNFHKFWVKMRPGSTVIIDCDKGVYQCSDYVEIFYSQSLNPGSLKCSSTGSPLPKIRWYHYS
WLNITSIDPEDGGLYTCEAFNGIARSSASIQVQESLTIHCPYSGYPIESITWFVASRGNGTLTLVDADEGWFKCEVRN
KEGASGSLYIDIEKPSILREGMRTTVVCSIGLGEPPFRITWLKDGSSLKITDIRRKHGNYTCKAASARAAPKWILKGSK
VLMNCQAEGIPPPVYQWKKNGSLAIIDSTKADEGDYVCEVTNNKGPSLTSISRLFTKSYELVKVEAGIICDPLGVTLD
SPLVDSGTFICGKAEKTVHLIVQGPPRSPSRDITIAWEAPSPILDYLLPETIYRIRVLAQNTFGRSEPSTEEEAPGEAPN
NVRVESISSQTLKIIWRKIEGYHIGLKRNTKYGLVIRPYNKKGPGVTSPPGEIHRSLTVEIGYIVNYLEDLRCGTKYTVSI
SAFNCPILSFVVQYLIPATWYDLLMIANSEPGTEAQYLFAT

>Tetranychus_7 tetur07g02520.1

RSLTIHVHDGGTTAFTCSEWSSQLMSVTSWCRVICKLTVTCIASGYPLPSYKWRYIQVNQFLVIKNLTQPDMGTIGC
WVNNSLGLRVNLSISQVTLINCEQGYYQCHGIVQLFNSIILSEGQLVCKVEGYPMPRIIWTLDSKLKVESIILLDAGLY
GCVAENKKIVHSNRVNVGTQLTVHCPVVGYPIDRLLWLPYNSNDGSLEIRNQDQGKYTCRAFQGNYSESTFLLSIV
KPVILHEGQRASISCTVATGDYPMVITWSKDGSSLLFESLDVTHGNYTCRSTNDAGAPLWTLEGNKVIIHCQANGY
PVPRIRWSRNGSLIIHETVSSDAGYYLCQAHNSVGPGLSKVVNLFTEHNLHNYSKIGLTCIAHGLHISSSKSDNGLFKC
GTDELTIKLTIQEPPSQPSRWISISWRTQSPITNHLRPGQRYTLVVIAENALGRSNSSTEEEPASSSPLNLSLSPLSSTSI
RVTWGQILGYYIGLKQLTKYSIIVHSFNSKGSSPATLPSTPSTSIHLTWGYHIYHFQGLECATLYHFYLVAYNCPIEDFII
KYHAIGSISKIQISVHNKLGTKVEYIFSP

>Tetranychus_8 tetur08g01100.1

------------MLLLLLFLLTHIYLTT---CLPDN-------------------------------
FTEQIVERKSLKCSAKGNPLPQITWKQDSYVNISSTKTDDGGFYSCKASNGSLQHTARINVAQNIQITCPYSGYPIKS
VTWLPQNHRNGTFVINNVDAGQYQCAVTDEDGAKRDLFINVVSPIVLVSGRRAGVACIVSAGDLPIRITWLKDGSF
LTFDKVSRSHGNYTCLAENPASPPSWIVEGNSLTLDCQADGSPRPIIRWKKNGSLTIREVAKEDAGHYMCQAMNG
IGPGASMVVKVFEKKFETHTVKLGIRCVAIGFKIRTAERDSALFTCGKDNSNFQIVVQESPDCPSRSFALTWSHPSPIL
SYLEPQTTYLVTLSAQNGLGTSSECTDGEAPTGAPRDIKVVPISSTSLQVNWGPILGYYVGLRKLTRYLIIVQAYNKKG
AGPPSPPEAPFNSALLSWGYIVYHIKNLSCGKRYQFYIVPFNCPILNFNVQYLSPATWYNVFITAHNEAGTGAEYTFG
T

>Tetranychus_9 tetur09g01130.1

ENYSIQSSDGNAGLIPCPPMVKDFVKVIHWCTVKNRLTVGCLASADPVPVYYWTLQSHGPILFLRSVQIQDSGVYV
CLVNNSLGLTVSMRPGEPASIDCDKGMYQCFSTLHLFKSQLLEEGSLKCSANGSPLPQIKWSLYSFLNVSSITREEGG
LYSCIAFNGQSVYSAPISITGRLSIDCPFSGHPIGTITWLPISRRNGTLQLVDVDQGWYRCEVSNGQEAQGSLYVHIE
RPVILTEGMRATVVCSILSGDPPLTFTWLKEGSSFRIKSVKRSHGNYTCVVSSSNIPPRWIIKGRNIVIDCQTDGYPQP
THQWKKNGSISIVGITKEDDGVYICESSNNVGPPLSATTRLFNKNFELIEANKGLSCLASGVILGSVDTDSAFFTCGA
DEKNIQLVVRGPPEPPSRAVALSWLPTSPLLGYLMPETLYRFRVIAQNALGRSKPSTEEEAPNEAPTNVRAEALSSRS
LRIYWRKIDGFHIGLKRSTKYGIIVRPYNKKGLGVPSPPGEVHRSLTIKIGFIVNYLENLRCGTKYSISITPYNCPIISFVIQ
YLIPATWYDLLMIANSEPGTEAQYLFAT

>Tetranychus_10 tetur11g03400.1

-------------MLSSKVLAFHYILMLTY--
SLLSPSNVTVSPDSLRCAAKGNPLPQIMWTTHSFINISRVSVEEGGRYECTAINGSESAQSWINVSRPIYINCPYSVYR
LSTLSWLPINRRNGTLVVHNVDEGEYKCLVEDKDGAERRVSVRVVAPTILQEGMRASVTCTITTGDVPIDIQWFKN
GSTLIFKPLKQDHGIYSCVASNEASPPQWKLEGQSLVIDCQAIGKPEPRVLWKKNGSLYFIEITPEDSGSYMCEASNG
IGTPLSAVVKVFTERFQSIKAQKGLGCVSHGFLIRSSSRDSGLYTCGSDERKIELIIQDEPDAPSRSFQIQWNEPNQIKK
YLSPKTSYHIRVRAENDYGKSGWSTDEEVPDNPPSHLQVSATGSTSIRVSWNTVTGYYIGLEKLTKYTIVVQAYNNK
GAGPLSKPSSPSHNIELAWYFVRYKLIGLDCGSPYHLSLLAYNCLIDYFSVQYLEPACWYNIWISARNGAGTEAEYKIA
T

>Tetranychus_11 tetur12g03540.1

--AISCL-----
---------YHAN-------------CQSKY--------------------------------LPLSD-NETLIIKSPDQAHSDCTVHYYFQAPNSIA---
SAPVILEEGSRSKILCTVIKGDPPFTIKWLKDGVALVFSRVKLNNGNYTCLVSNGVARPRWTLEGKDVLIHCQADGF
PDPVIQWKRNNTLEIKSTDKSDAGYYMCSASNGIGPGINTIIELFKEEFKVETVRKGVRCEALGIRVGAADRDSSLFT
CGTDKTNIQAIVQEKPDPPSRDISLSWSPPSLILHYLWPDTSYHLRILAENVLGTSEPGTELEVPSEYPSDVRAEPFGS
KSIKVKWNRIKGYYVGLTPLTRYIIIVKAFNRKGAGPASSPKAPHNSISIKWGYRLYIFGDLSCGRKYEIYVTGYNCPIK
DFKIQYLIPDASYRIRIVARSDSGTQAEYNFLT

>Tetranychus_12 tetur16g02990.1

SLLEPRVYDGNIAVFRCPDHLKDTVRVKSWCEVKNDKTLPCIASGYPLVKYEWRYSTTDGLLIITEPKSSDSNEYRCIS
SNNLGLTLTTSPGSSLSLNCHEGFYICQQTIQLFTDQLLSPGSLICSARGRPLPQVTWTLDSYVNVSSIRTEDSGLYQCI
ASNGKVTHSRRINVGDNVWFYCPVYGYPYGEVSW--------------------------------------
VGPRIMEEGMRNVAFCTVSSGDGPIYIDWYKNGSTITFQSLTTQHGNYTCLASNSAASPTWIKEGAKVILDCQAAG
FPEPQIRWKKNGSLFISSIGSSDSGKYMCQATNGVNPALSKVINVFDSTFVSKIVKKGLSCNASGLAIGVVDRDSALF
TCGSAEKTFRLIVQEPPETPSDSVLLTWSEPSPIIEYLKATTNYEIRVIAKNALGSSEPSTLEKEPVSPPQDVRITPRGSS
VLKVSWPRITGYHIALERLTRYEVKVQGFNMAGDGPFSAPKAPPMTIDLSWGYLVFYIYSAQCGTRYQIYVLGINCP
ILYFDIQYLSPATWYNLLMKAVNEAGTDAEYVFAT

>Tetranychus_13 tetur16g03020.1

PKPEIKVDDGSDVTLHCDSSFSNQFKVSAWCQVQDLLTINCPIIGFPRPDIRWSHLYPESIMFIPRLTFRDSGKYQCS
ANNSFGIRVAITSGSDVTFNCDNGIYQCHSSITLFPEQIKITGSLKCSAFGSPIPTIKWRIDSFLNISSLTVQDAGTYECI
AENSSVSHLSRIDIGQTYQLYCPYTGYPVDEVYFLPFEERLGKINILSVDAGFYKCIVSSLNGYESNLYLHVAPPILLEEG
MRISIMCSVISGDPPITIAWLKNGSSLIIENLNKTDGNYTCSASSLHSKPHFTLTEMPVLFNCQADGVPSPVIRWKFN
GSLHIRTVSLDDEGTYLCEASNGLGKSISVSAFLIRPVTSKSIVKRSLICQASGLTIHSATRDSALYICGSAKTIVKVIVQE

PSDPPSNSLTVTWTVHSPILGF-----------------------------------
TASVPLDSKSVRISWGVIEGFYIGLKKSTKYAIIVQAFNKEGEGPYSPPKAPSGSASLTWGFILSFLSNLLCGTKYSARM
CAFNCPIDYFEVQYMLPGTWYDLLVTAKNEAGTEAKYLVAT

>Tetranychus_14 tetur16g03946.1

SLLEPRVYDGNIAVFRCPDHLKDTVRVKSWCEVKNDKTLPCIASGYPLVKYEWRYSTTDGLLIITEPKSSDSNEYRCIS
SNNLGLTLTTSPGSSLSLNCHEGFYICQQTIQLFTDQLLSPGSLICSARGRPLPQVTWTLDSYVNVSSIRTEDSGLYQCI
ASNGKVTHSRRINVGDNVWFYCPVYGYPYGEVSW--------------------------------------
VGPRIMEEGMRNVAFCTVSSGDGPIYIDWYKNGSTITFQSLTTQHGNYTCLASNSAASPTWIKEGAKVILDCQAAG
FPEPQIRWKKNGSLFISSIGSSDSGKYMCQATNGVNPALSKVINVFDSTFVSKIVKKGLSCNASGLAIGVVDRDSALF
TCGSAEKTFRLIVQEPPETPSDSVLLTWSEPSPIIEYLKATTNYEIRVIAKNALGSSEPSTLEKEPVSPPQDVRITPRGSS
VLKVSWPRITGYHIALERLTRYEVKVQGFNMAGDGPFSAPKAPPMTIDLSWGYLVFYIYSAQCGTRYQIYVLGINCP
ILYFDIQYLSPATWYNLLMKAVNEAGTDAEYVFAT

>Ixodes ISCW001673-RA

QLHVLTTRVVNMLVLKARSLSVHPLAATPKCQTRHRLTLFSGTCHCLTSKRRWRFSVSGGTLSIRHAAVADSGRYLC
VANNSLALSAVVVPGGSATFSCDSGMYQCQGAARLFSEQSAEPGSLQCSATGSPVPRITWSLDSFVNISAARTEDG
GLYACAASNGSVEHAARLNVGGPLRLDCHYAGHPVDRISWLPSSKRNGSLVISEVDNGTYTCHVSGPLGTSGTVTV
NVVRPTILQAGMRARLGCTVISGDPPFEFDWRKDGSDLTFASLGPRHGNYSCVVSNAAARPLWVIEGRDARMDC
RADGYPVPTITWERNGSLLVKNTREQSAGRYLCQATNGIGSGLSKLVHLFDIKFRSEAVQRGLRCEAQGLEISSVER
DAALFTCGGDDLNIKLIVQ---
GLVSMKTTLLWSGESKATSFLRPAVAYRCQVRAENEVGIGEPSTGIEVPGGPPLEVKATAVDSQTVRVTWGELKGY
YVGLRKFSPYVVLVQAFNAAGPGPRSPSQAPSESIRVTWGYRIWYLTLVPRNARVEVWVTASSCVVLLAIFFFLGAD
GSLALRRIEAADAGTCNVRNKLA

>Ixodes_2 ISCW022831-RA

-----------------P--------------------
LACVAQGYPAPSYRWRVRVFRGFLLIQSTVRQDAGTYVCAANNSAGLKVSVSPGKTVVFNCDRGMYQCQGAAQ
LFPDQLLKPGSLKCSVTGNPLPQISWFRYSFVNVSSLATEHGGVYSCRAENASVEHTARLSVGSDARMQCAASGYP
ITLISWLRPSSRNGSLHIVHVDQGWYECAVSNKKGAVGSMFLRVAKPVILQEGMRTTVVCSVLSGEPPVEIDWLKD
SSSLTMDNVTRRHGNYSCKATSGIASPRWMKQGQRTVFDCEADGNPLPVHRWKKNGSLVIVEVTPKDQGHYLC
EASNGVGPALSVAAYLFHEEFETKTVRSKISCEVFGVFIPSVGRDSGVFVCGKKDRTIQLIVQGPPDIPSRSATLFWT
QPSPLLGYLVPGTAYILRVVAENAVGKSGPSTEEEAPSGSPYEIRITATSSKTVHVRWGKLKGFHVGLRRFTQYAVVV
QAFNNKGAGPLSPPSAPSSTLELEWGYVVHYLTNLICGNRYQVTITAFNCPITFFVVQYLAPGTWYDVMVSAYNDA
GTEVEYRLAT

>Ixodes_3 ISCW022837-RA

PPRWKVAGENVVVDCQAEGFPPPRIWWEKNGSLMVREAERNDTGFYLCQASNGVGSGISKVIELFKNAFNSKTL
RKGIKCEVVGLEIHAVDRDSALFSCGQDETRTQLIVQEPPGAPSRSMSVSWDQPSPISAYLRPVTTYIVRIRAENSLG
PGEFSTDEEAPEGPPLNVQATAVSSSSVKVTWGLLKGYYVGLARSTKYTVLVQAFNDKGSGPPS-------------
GYYIYIFQDLQCGSSYQFYVASYNCPIKSFLVQYLAPGKWYILQVTAHSEAGTEQEFTFST

>Ixodes_4 ISCW023871-RA

YKYEPRVYDGNTAVLKCPSYIRQYTLVDAWCRTRHRLT---------------
RMQSSNGVLSIRAVNVHDGGRYVCIARNTVGLSAEVSPGLPAVFNCDAGMYQCQAAAQLFAPQVVKRGSLLCRA
RGSPAPELTWAIDSLLNISEARHEDSGEYSCMARNATEAHSARLEVGTELALRCPYGGFPVDSLTW------------------
----
VAPVIVEEGTRSRLMCSVSKGDPPLRFRWLKNGSIIKFARVRFVDGSYVCFVSNDAASPRWKTEGASVFLHCASDG
FPSPAITWKKNGSLLVREVEESDQGFYLCEAQNGIGPGISKLVFLFEVKHRSFLLKKGPQCLAAG-----AER-------------
----

>Ixodes_5 ISCW023928-RA

HPYKPQVFDGNTAVFRCPSFVKDFVDFVSWCKTKHKLTIACAVQGFPVPSYSWRTAQVDGSLFLSTPVVRDAGKY
FCVVNNSVGLKAELQPGHPVTFNCDAGVYQCQASAELFAEQTSLPGSLRCSASGSPLPQVTWTLDSFVNVTELRVE
DGGEYVCRATNGEAKHAARVNVGRSFQTVCPVAGFPIHSVVWLPTNHR-STLTVHNVDEGEYSCVA-
RSGNARGNTFVHVVPPVISNQGMNVKMLCSVVQGDPPISLRWMHGASVLTIKGVSMRDGNYTCEASNAALPP
MWTTEGETVVLDCAADGFPVPRIAWKRNGSLVVQDAEISDSGFYLCEAHNGIGAGLSRVVSLFSTKFSSQNVKRG
LRCDASGLTISSTERDGALYTCGSDETNVQLLVQEPPSAPSRTVEIMWSPSS---

>Ixodes_6 ISCW001732-RA

--------------MRTPPRLRQAI-------------TGCVGPCRTSRARGW----------
VRRSLLPDAESFGDSSGQATAKSVRVRDGKSVSLVCPPRLRQA------FSQPCRLSSSSECVRTGCVGPCRT----
SLLPDAESFGDSSGQATASSAS-------
EEFAGKSVSLVCPLYVAAWASVSWIPFNHRDGSLSISNVDDGSYVCRFTDSRNHTGNVLLKVEPPVIMQVGMRIK
VFCTVVRGDAPFLFTWLKDGSMLSADSLQLEHGNYTCVVKNQAAPPKWEVEGRNVQLQCSANGTPQPTITWMI
NGTLSIHQLEPEESGYYLCKASNGFGEDLSKLVFLFDVKFRAHAVKRGLACTATGLVVSTETVDSGIYSCGSDETSMR
LLVQEVPGAPGNSLLLSWAEPSAITRYLRPARVFSLRVKAENGVGWGRFSTEEDSPASPPVNITARPTGPNSIKISW
GHLKGYYISLRLSMSYSVTIQAFTSKGAGPMSPPSPPTSSVTLGWEYVLHQVRDLECGTTYQFYMTAHNCELLDFSV
RLLTPGATYHVHVVAKSTAGTEAQYEFAT

>Ixodes_7 ISCW016847-RA

LPCAAQGSPPPQYRWRTSQVDGVLVVRKATLRDAGKFTCVANNSAGLTATIQPGQTAIIKCDKGMYQCQAGFELF
RPETVHPGSLKCSASGNPLPQITWSLDSYLNFSVVQVEDGGDYRCTANNGTVLHTARINVTPKLHFS-
KVKSYQFVEVTILFRNNL-
STQLLTRQDLGTSEKFRLAFFSCPALSEMPAVKPTILREGQRSSVMCTVISGDLPINITWFKDDSTLLFKSLRLDYGNY
TCVAANEAGPPQWIIEGRSAVIDCEADGFPMPRIRWTKNGSLAINDAKEEDAGFFLCQASNGIGQGLSKRWSRFK
SKFSAEMIRKGLKCDATGILIRQTDRDSALFTCGHDDTNIQLIVQDPPSAP---
----YLHAEATSFTSVSIKW---------------------------------P---

>Ixodes_8 ISCW017407-RA

SFVNISAVRAEDGGLYRCSAGNGVSEHAARVNIGEVLSITCPVGGHPIDSITWLPYNHRNGTLLVQDVDEGLYSCTA
RNKDGAQNSVSVRVVRPAILHQGQRFNVLCTVSKGDSPIHIAWYKDDSTLIFDKLVPEHGNYTCEARNQAGPPRW
RIEGGTAIIDCQADGFPVPRVRWTKNGSLAVHNSDEKDAGFFLCQASNGISPVLSKVVKLFKSKFKAESVQRGLKCE
AFGITIRGADRDSALFTCGTDDTNMQLILQEPPDSPSRHVKLSWVTPSPVVKY---
---------------------------------DSGKYGKTS--

>Ixodes_9 ISCW017597-RA

---------------------------------------LPCLAQGNPPPTQTW-------
ALVLHGARTQDGGKYACVVNNSAGLSARLEPGRTANLSCDRGMYQCQGTAQLFSEQEVRPGSLKCSASGNPLPQ
VTWTLDSETSAAALKLLEGFSF-
LFLRDGGANFAIYCFLGRPVTLHCPVAGHPIQSIAWLPQNHRNGTLVISDVDSGWYSCVAQDPDGAKGQLALDVI
PPVVLTEGKRAGAACIVSDGDLPISVEWRKDGSFLSFAAVRQSHGNYTCVASNPAAPPRWRQEGQAVVFDCQAE
GFPVPVIRWKKNGSLSIREADRKDGGQYMCQAINGVGPGISTVVRLFERKFQALTVRRGLTCRAVGVHIPTADRDS
SLFSCGRDDTNFQVVVQEPPDKPSRAATLVWAPPS---

>Ixodes_10 ISCW017602-RA

QKFTANVYDGNSALLRCPPAVKDYVRVTSWCQVRNVLTLPCLAQGNPPPTQTWRLTLLEGALVLHGARTQDEGK
YACVVNNSAGLSAHLEPGRTANLSCDRGMYQCQGTAQLFSEQEVRPGSLKCSASGNPLPQVTWTLDSYVNLTSV
RVEDGGRYACVARNGAAQHSARLNVGRPVTLHCPVAGHPIRSIAWLPQNHRNGTLVISDVDSGWYSCVAQDPD
GAKRQVALDVIPPVVLTEGKRAGAACIVSDGDLPISVEWRKDGSFLSFAAVRQSHGNYTCVASNPAAPPRWRQEG
QAVVFDCQADGFPVPVIRWKKNGSLSIREADRKDGGQYMCQAINGVGPGISTVVRL----------------------------------
----

>Ixodes_11 ISCW020407-RA

QQYEIRVYDMNTGVLRCPNYVREYVIVTSWCQTKDTLTIPCEAQGHPQPMYRWRLVLVGGTLVLRRATVQDSGT
YVCVVSNGAGLEVEMRPGETVTLNCDSGLYQCQGHAYVFEESVVRRESLRCAATGTPLPQITWSVYSFVNFTKVRL
EDGGTYRCEAANGQDSYSARLNVGRKLLLHCPYSGYPISKVIWLPSSKRNGTLALETVDEGRYSCIVRNDQDATNQ
LNLRVVPPSIPQLGSRASVTCSVPEGDAPIRLSWLRDGSTLVFKSLREEHAVYTCLASNEAAPPRWRLEGERVVLDC
QADGTPEPRVRWKKNGSLVIQEIETSDAGGYMCEASNGVGLPLYTVVQVVRQRFLSHMTGKGLRCDASGFTILLA
EKDTGAIKCGHDEQITHLSIQDRPDEPSRSVTVLWKSPS--

>Ixodes_12 ISCW022820-RA

LRASVKPGHSLRLNCDRGMYQCQAASEVFPESIHKPGSLRCTATGNPLPQVTWDLDSYVNISSVRTEDGGIYRCRA
SNGLASHMARVNVGGNLVVHCPVGGYPLTAIRWLPSGHRNGTLVVSEVDEGTYECVAENGRGARRALHVHVVG
PKVLEEGMRSVVVCVVIDGDPPVFIGWLKDGSSLTFHSVGPKHGNYTCVARNPAAPPYWRKQGESVLIDCQADG
VPHPQIRWKKNGSLVLREIGLNDAGEYMCQATNNVKPSLSEVIKLFKTQFSSQNVRKGIRCEAYGILVKATDRDSSL
FTCGRDETNFQIVVQEKPDSPSQSVAMAWMQPLPLTSYLNPVTTYNFRVLAENSLGHGNPSTKEEAPSNPPTEIQI

EPTSSKSIKIKWSPVKGYYLGLRRNTEYSIRLQAFNSAGSGPASPPSPPATSVHLVWGYVLHYLEQLRCGTRYQLYME
AFNCRISFFVAQYLAPGTWYNLLMTAHNDAGTDA------

>Ixodes_13 ISCW022826-RA

FTKKLVDLGSLRCVASGNPLPRVTWALDSYVNVTSSTRDDGGLYRCEASNGSAWHDDRIDVGTTLVYHCPFTGHP
APKVTWLPHNERNGTIIVVDVDEGVYTCKAATPKLAKEDLLVKIKKTVLLAEGMQVVITCSVRSGDTPIKIWWLKD
GSNLVFNEVGRAHGRYTCVAENDGGPPKWKIEGSRVTFDCQADGHPAPLIRWKINGSLIINDVEPKDAGKYLCEA
TNGIGVGLSTVVRLFKVSYQALRVNKGLVCEAFGLRFAAAERDSGLYTCGKDETNIKLLVQETPDSPSRRITLRWNA
PSDIIGYLQPITSYHLRVLSVNQLGRSDPSTDEEVPSKPPEELVVVPVTSQILKASWGRIRGYYVGLKRSTKYSVIVQAF
NGKGAGPPS-----YLGFVLYW------
LEELNCGTRYHFYVVAFNCPIRFFAVQYLQSGSWYHLLVSASNDAGTEAQFVFAT

>Mesobuthus MMa08972

--------------------------------------MRI--------------------
LPLGRRNGTLVIHHVDDGQYQCRATNDQGASRKVYIKVVPPKIAEEGMRASVSCSVPIGDPPISISWLKDNSTLVFK
SLTQEHGTYTCVATNDAAAPKWKIEGHSLLLDCMAVGRPEPRVVWKRNGSLYFREIEQEDDGKYMCEASNGVGS
ALSTVVKLFKQKFQASVIQRGLKCEAFGLRIISTRRDTALYTCGQDETSIQLTVLDKPDPPGRSISLKWNKPNPITRYLI
PLVRYNFRLSTENRLGRSGFSTDGEV------
KVHATPIGSSRLHVSWGLLKGYHVGLKRATKYSIVVQAFNDRGAGPLSPPQTPSSTIHLTWGFIINWLTGLKCGTKY
QFFITSYN---------------------------------------

>Mesobuthus_2 MMa43828

--FKSRKSHPG------

---AIVHL--

GPIKGYYVGLYKFTRYSVLVQAYNSMGAGPRSPSKPPSQSLHISWGYKILYLDGLECGASYHLYVVALTCPITRMMIE
YLNPETWYVIRVTAYNAAGMMAEYDVIT

>Mesobuthus_3 MMa47028

QYYEVQVYDGNTAVLTCPSFVKDYVTVTSWCQTTHRLTLPCAAQGFPVPRYSWRMTQLSGSLIIRQVNVEDEGKY
VCLISNSAGLSAHVQPGKTAKFNCDSGMYQCQGTAQLFSEETLQPGSLKCTASGNPLPQITWTLDSYVNITSVRVE
DGGVYECHAKNGTISHSSRLNVGESLVLRCHVAGYPIETITWMPINRRNGTLVIRDVDDGSYKCKASNRQGASGTV
RVHVVKPVILQEGMRSRLVCTVIQGDPPFVIHWRKDRSDLTFASVSPRHGNYTCIVSNAAAPPRWNVEGQNVRID
CLADGFPTPVITWERNGSLLIKNVEEDDGGYYLCQATNGIASGLSKVVFLFETKFRSETVKKNMQCSASGITIMSAE
RDSALFTCGRDDMNIRLIVQ--FSEAMSS--

>Mesobuthus_4 MMa54582

QRYNPNVYDGNTAVLRCPSFVREYVSVDSWCKTRHHLTLPCVAQSYPAPTYKWRIWISHGVLTIKKTKMEDSGKF
VCIVKNSAGLTAYIQPGRQATFNCDKGMYQCQGTAEL--

EKISKPTTLKCSASGNPLPTLRWLLDSYINISDVRVEDGGEYQCEASNGSIGHKARLNVGEDLILRCPYGGYPIESITW
LPLNPRKGTFILRNVDEGEYTCVVRNAVGASGTTYISVVSPVIAVEGSRAKLICSTTTGDPPIKYQWLHDGSIIIFRRVT
SKHGYYTCLASNSAASPKWIIEGNTVLIDCSATGFPIPTITWRKNGSLQIREMEEDDAGYYLCQANNGIGGGLTVVIK
LFEINYRSHTVRTSLKCTVKGLTIKSTSRDTGLYTCGTDDTNIQLVIQDVPDAPSRTVSLQWEVASPITGNLTPVTSYTI
RVITENVLGRSDPSTEEEAPAGPPIGILVHSTGAQSLKVTWGKIKGYYVGLRPFTKYAVVVQAYNKIGPGPRSPPTSP
TNSVEVQWEYILHYLEGLHCGTTYHLYMTATNCPIHHFTVQYLLPEREYNLMVTAYTDAGAQEEYEFRT

>Mesobuthus_5 MMa12454

------MHN---

IRPVIVHMGQRLSITCTVIMGDPPITIQWLRDNSTLLFEAVKPNHANYSCLAKNEAGPPRWKIEGRSITIDCQADGF
PPPRLSWRRNGSLTILEVDTDDSGYYLCQASNGIESGLSKVIKLFKTKFHAEIAKKGLKCEAFGLILYTAGRDSALFTCG
RDETNIQLLIQEPPDSPSRTAHISWSPPSRLTKYLHPRTKYIFKLIAVNALGQSEQPTDEEAPGGPPLSIHATPLSASTIK
VSWGRIKGYYIGLKRYTKYSIRVQAFNSKGTGPPSLPKTPSSTITVKW--

>Mesobuthus_6 MMa26231

---------------MEKGGL---------------------------------------
LPVNHREGTLTIENVDQGDYSCVAKNREEAKGSVYIFVVAPMIANEGMKIKLMCSVLEGDPPVRVVWMKNNSVL
VFRKVAFRHGNYTCVASNVAAPPRWVIENHQVTIDCLADGHPKPRMYWRINGTLLIQEVTKLDSGFYLCHANNGI
GAGLSKVIYLFETKFGTHTVAKGLKCEALGLTISQTGRDSALYTCGSDETNIQLVVQ-----------ANWKDS------
LTPSTTYNIKILAENNLGMSDPSTEEEVPGGPPLDVKVQATGSQSLK---
SVIQGYYIGLKRLTKYIVIVQAYNNVGAGPRSPPTSPTFSISVQWDYILHYLKGLKCGTRYRLHMTASNCPIQYFSVLY
LAPGREYVLRVTAHSEAGTEAEYIVHT

>Mesobuthus_7 MMa27504

QYYEVKVYDGNTAVIRCPSFVKDYVTVIGWCKTRHRLTIPCAAQGLPIPKYRWRFEQVGGSLIIKDLRPDDDSKYVC
VMRNNVGLVATIEPGKSTIFNCDEGMYQCQATSELFTEQTYQPGSLKCIASGSPKPEISWTLDSHVNISNVRVEDG
GYYRCKATNGTILHEAKLNVMETLVVICRVAGHPIDSIHWLPFNHRNGTMEIIQLDTGIYTCIARNQQGAQTLAIN
VVPPLILHEGMRTRVYCNIASGDLPISVTWLKDGVALAIEKLNPKHGNYTCVASNAAALPHWIIEGNTVMLNCKAD
GFPTPQIVWKRNGTLRITDSLGEDAGYYLCQATNGIGAGLSTVIYL------------------------------------GDKD--------------
----

>Mesobuthus_8 MMa27661

LPCAAQGFPIPSIRWRFVQLDGTLVLHQATVKDSGKYVCVVNNSFGLSATILPGKSATINCDKGMYQCQGTTELFP
AQTIQPGSLKCVATGNPLPQIIWKLDSYVNITSVQTEDGGEYQCTANNGEVSHKSKVNVGESLNVRCPVGGYPLET
IHWLPYNHRNGTLEVHHVDEGPYTCVARNREGAQSTVLVRVVKPVILREGQRSSVLCTVGSGDLPIKIRWFKDGST
LLFESLGLDHGNYTCIASNEAGPPRWVIESKNVVIDCQAEGFPQPRVRWTKNGSLAIHDVKEIDSGYYLCQASNGIG
QGLSKVLKLFKSKFTAEMVKKGLKCESYGVLIRQADRDSALFTCGRDDTNIQLIIQ----------------------------------
VAAH---------TAEMVKKGHNTRLKCESYGDKPITVTW---

>Mesobuthus_9 MMa36242

VKVNIDPGESATLTCDQGMYQCQAIAQLFGERIVHPGSFRCMASGHPLPEITWYVNSMINLTDVTVNDSGLYECV
AQSGRANHVARLNVGHNLYLECPVTGYPLDSIIWLPLARRNGTLLVKEVDQGFYTCVVRS-
QGQKRSVAVIIKPPKIVREGTLVVLTCAINQGDPPFDLQWFKNGSILVFSEVKRHHGNYTCLAKNAGGPPHWLIEG
QSITLDCKAEGLPPPTIIWKKNGSLGIREAESANSGYYLCQANNNVGAAISKLVTIFETEHTVETFRLHLLCSASGLIKS
KVAGDSGLYACGVSEATFQVIVQEAPASPGRSLTISWQEPSAITGYLQPATSYSLRVLAENAIGKSNLSTEEEAPGGP
PLRIKASPTGPNSIKVSWGNITGYYIGLRRSTKYSVSVQAFNGKGTGPRSPPTAPTSSITLSWECVLHHITSLDCGTRY
QFYMVCHNCPIREFTLKLLTPGTWYMLQVTAYSSAGTEAQYEFQT

>Mesobuthus_10 MMa41956

CATKHLLSLTCVAQAFPLPTYRWGLLVASGSLYFQQTNIRDSGIYICIVNNSAGLKAEIQPDKSVTLTCDKGIYQCQA
ATELFIDRTTQPGSLQCVATGTPLPQITWTLDSHVNISSVRVEDGGHYVCTAANGDLRHTGRLNVGENMVVQCRV
AGYPISHITWLPTNRRNGTLLVEDVDEGSYSCMAQNAEGAEGGMKIHVEKPLIQKEGMKIRVVCTVASGDPPFTIT
WLKDNSSLTIENATPKHGRYTCVVKNEAAPARWRIQGRSVTLDCQAEGYPQPQIRWEKNGSLTIQDVARDDAGY
YLCQATNGIGSELSSVISLFETKFQTQMVKKKMTCSVTG-----------------------LIIE---NDP------------------------------
----

>Mesobuthus_11 MMa41957

------------------------------------MSV-------------------------R--
----
DSALYTCGQDDTNIQLIVQEIPDPPSRDVKISWEPPSLITQYLHPATVYELYLVAENAIGRSGLGTEEEIPEGPPSMVR
AESTSSQSLRISWGKIMGYYVGLKKYSYYTIVVQAFNSKGAGPRSPSLPPSQSIKVSWGHKVVYALRLNRERRYEFW
VTASTTPEKIWTFRGILDEDAGNYTCRVQNVYGDEIGFSLNV

>Mesobuthus_12 MMa43724

YHEQTVHPRSMKCIAVGNPLPQITWTLDSYLNITSVDVDDSGVYTCTATNGQKSHSQRLNVGKTFIISCPFGGYPYS
SIIWLPVGQHNGTLVIADVDQGRYSCEVSNDQRAERTFPLIVSSPKILHEGMRTAVTCIVVAGDGPLKIKWMKDNS
TLTIKKLAYVHGNYTCVATNDLAPPRWLLQGKSAMINCQASGVPQPHVRWKVNGSLNIRSVEESDGGLYVCEAN
NGVGNGISSIIRFFETKFLEKNFKKGIECNADGIIISSVERDSAMLTCGEDTANIQLTVQDVPDPPSSSIRISWSQDSPI
TEYLNPQTTYSVSIRALNALGKSVASTLEEPPRKAPGSVKVVAHSSQVLNVSWDLIQGYYVGLARFTDYSIIVQAYNG
RGAGPAS-------------GYVLHFVHDLDCGTRYYFYLLAFNCPITFFVVQYLSPGTWYTLLMTSHNEAGADAEYVFAT

>Parasteatoda aug3.g10045.t1

MHDGNTAVLKCPGFVKDYVSVEAWCRTKHRLTLPCAGQAFPLPNYRWRREIVGGTLILRNAEIRDSGRYICALTSS
VNLSVSVYPGESVTFNCDHGLYQCQGIAQLFKNMTVNPGSLKCSASGNPLPQITWSLDSYVNISRANVEHSGEFTC
SAKNGEIQHSRWIRIGELMKIRCPAGGYPIHRISWLPDNHRNGTLLVRQVDEGRYTCTLRNKEGAKGSVYITVVRPV
IVQMGQRLSMTCTVIKGDPPITLKWLRDESTLLFDP-----SN----------

PPRWKTEGRSVMIDCEADGFPPPRLTWRKNGSLTILDVAEIDAGYYLCQASNGIGSGLSKVISLFTNKFHAEIVKKG
MSCQA--
LLIESVGRDSALFTCGKDETNIQLIIQEKPDIPSRSLDLSWSPPSRLLKYLIPNTRYSIQISAVNALGESENSTEEEAPGGP
PLSAKALPLSSTSVKVLWGQVKGYYVGLQRSTKYLIRIQAFNSKGAGPPSPPKPPSSSVTLKWTTGFIVIENLHCGMR
YEFYVT--DCPITHFEIKYLTPNTWYQLQLTSHNEAGTEAEYIFTT

>Parasteatoda_2 aug3.g1203.t1

QYYEVQVYDGNTAVLTCPSFVKDYVTVTSW--------
LPCAAQGFPVPRYSWRMAQLSGSLIIRQVTLEDSGKYVCLISNSAGLTAHIQPGRSARFNCDSGMYQCQGTAQLFS
EETLQPGSLKCVASGNPLPQITWTLDSYVNITSVRIEDGGEYECHARSGIVSHAAKLNVGESLMLRCHVAGYPIDTIT
WLPINRRNGTLIIQDVDDGTYKCKAWNKQGAYGTVRVHVVRPVILQEGMRSRLVCTVIQGDPPFVISWKRDESDL
TFSSVTPRHGNYTCIVSNAAAPPRWHVEGRNVRIDCLADGFPAPVITWERNGSLLIKNAQDDDAGYYLCQASNGI
ASGLSKVVFLFDTKFRSETARKGLKCESTGITIKSAERDSALFTCGQDDMNIRLIVQEPPEPPSRSAKISWSSPSAIIRYL
RPARAYSCRVRAENSVGLGEPSTAEEVPGGPPLDVKAEAVDSQSVRVTWGPIKGYYIGLQKFTSYSVLVQAYNSM
GAGPRSPGEAPSQSLHISWGYKVLFLEGLECGVKYQLYVVPLTCPITRMSIEYLAAETWYVVKLTAYNAAGTTAEYD
VIT

>Parasteatoda_3 aug3.g14097.t2

GEDLVMRCPYGGHPIKGIRWLPLNHRGGSLTIQSVDEGQYSCIVRNNEGSTSSTHVSVVSPVIVDEGSRTKLMCVV
TKGDPPLRFQWLKNGSIVTFKKVSSSDGHYTCVVSNIASPPQWTVEGNTVWMDCAAVGFPAPNILWKKNGTLVL
SEVEESDSGSYLCQASNGIGAGLTKAIFITKSSNNQYAIKEAIKLIVQGLIVSSAETDSGIYTCGADESVIKLIVQGVPDA
PSRSISLQWEVLSHITGSLTPVALYYIRVIAENSLGKSKPSTDEEAPAGAPMEVHVHSTGAQSMKVIWGELKGYYVG
LQPFTEYDIVIKAYNSAGAGPDSPPTSPFSSIEFKWEYMLHYLGGLRCGTRYHLYMTASNCPIKHFEIQYLSPDREYE
VMVTAHSDAGTQAEYSFRT

>Parasteatoda_4 aug3.g14551.t1

---VPVVKV----------------------------NSVLLEL------
--PQS--------------
GYELHYLEGLECGVKYQLYVVPLTCPITRMSIEYLAAETWYVVKLTAYNAAGTTAEYDVIT

>Parasteatoda_5 aug3.g14610.t1

QRFDPQVYDGNTAVLRCPTFVREYVSVDSWCQTRHRLTLPCVAQGNPPPTYRWRIWVNQGTLNIKKVQDTDGG
KYQCIVRNSIGLIVTMRPGQEATFNCDRGMYQCQGTSELFPEHTAHSGSLKCVTTGNPLPRITWHLDGVLNITEAR
VEDSGEYRCQGENGSTFHAARLNIGEDLTIRCPYGGYPIKSIKWLPLSHRGGTLTIQNVDRGEYSCTVKNTEGASGS
TYVSVVPPVIADEETRNKLMCVVIQGDPPMRFHWLKNGSIVTFKKVVSSDGQYTCVATNVASPPQWTVEGQRIW
IDCAAVGFPTPSIIWKKNGTLVLSDVEENDGGSYLCQASNGIAAGLSKIITLFKEPFQTKTVTEELRCIASGMETLDSN
GSADECTI----------
DPSRPDSPSRSVTLQWEVTSHITGSLSPVTLYFIRIVAQNALGQSDPSTAEEAPSGTPREVQVHSTGAQSMKVSWG
YIRGYYIGLQPFTKYDIVMKAFNSAGTGPKSPPTSPKTSIEIKWEYTLHYLSNLKCGTKYHLYMTASNCPIKHFVVQYL
APERDYVIMVIAHSDAGTQAEYSFRT

>Parasteatoda_6 aug3.g15517.t4

QGYQVQVYDGNTAVLRCPSIVRDYVIVTKWCLTKHILSLTCVAQAYPLPGYKWGVILLSGSLYFQQVNVRDSGTYV
CVVNNSAGLSAHLHPEKPVTLKCDRGVYQCQASAELFSEKTLRPGSFQCAASGTPLPQVTWSLDSHVNISSVRIED
GGMYSCVARNGEIRHSARLNVGEVTSIQCRVAGYPIDEIMWLPTNRRNGTLLIEQVDEGSYVCSARAADSVDGSLK
ITVEKPVILKEGMKIYGMCAVSAGDPPFHISWLKDGSSMTIDSATTRHGHYTCVVKNDVAPPRWRMEGRSVSLHC
QADGFPQPQIRWEKNGSLTIQDITEEDGGYYLCQATNGIGPGLSSVVTLFEAKFRTQMVKKGLSCSVSGLRVTSVG
RDSALYTCGEDDTNIQLIVQEQPDAPSRVVRLSWEPPSLITQYLRPATGYDVSLIAENVIGASGQGTEEEVPEGPPLS
VKAEAASSDALKISWGNLKGFYVGLNPFSHYSIVVQAFNLKGAGPRSPSVPPSQTIKVTWGYKVFYLQNLQCGTKY
YIYVAALGCGIKSFVVRYLSPGTWYSIRVTAHNVAGTVAEYEMAT

>Parasteatoda_7 aug3.g16920.t1

QRYEIRLFDGNMAVLRCPSFVSDYVKVTSWCHTENEITLPCIAQGYPAPTYRWSVSQEGGVLIFHKAQLSDSGRYV
CSATNNLGLRITIIPGTTATFNCDAGIYQCQAAARLFQEKTMRPGSLTCIASGHPEPSIKWTLDSSVNFTSVDVTDSG
LYTCEAVNGYASYSRRLNVGERFQAICPFGGYPYESIVWLPINQRNGTLVISGMDEGTYSCEVSG-
GQVSRSIRIIITKPKVLHEGMRTAVTCVVVAGDGPLSTRWLKDGSTITIKYLKNKHGNYTCISGNDVSPPRWVIEGRS
AIIDCQADGVPQPHVRWKVNGSLNFRSVETTDAGFYLCEANNGVDSGLSTVVRFFYSKFMVLGARRGIECIAKGLV
IEKAERDSALFECGEDTMNIQVTIKDIPDAPSRSIKLTWDRPAPINQYLKPKTRYFFKVKCLNSIGESQYGTLEEPPRLP
PHSIKATAISSTSMNISWSRIDGFYVGLNRNVEYSFILQSFNKRGASPPSRPLPPSTSIKITWGFILHHLHDLLCGTKYY
CFLVATN------------------------------------

>Parasteatoda_8 aug3.g17018.t2

QPYKIQVFDGNTAVMRCPSFMKDYVIVTSWCVTRNRLTLPCAAQGYPVPRYSWRITELNGTLLIRKANIQDGGKY
VCIVNNTNGLQVRALPGKTAIFTCDRGMYQCQDAAQLFEPSTVEPGSLKCIASGNPLPQVTWTLDSFVNISSLRVE
DGGVYRCLATNGSLSHSAKINVGEVLNVRCPVAGYPVEEVYWIPFNHRNGTLIIREIDGGEYRCIARNSQGGERTLN
IRVTPPVIIEEGARSKLLCSVTKGDPPITIQWLKDGKAMLFTRVELRHGNYTCIARNLAAPPRWTTEGSDVILNCVAD
GFPKPQILWKRNGSLSIRNVEKTDEGFYMCQASNGIGSGISTVITLFKEEFLAETVRRGVKCQAFGVHIVSADRDSSL
FTCGKDEMNIQIIVQERPDKPSRTISISWSPPSPILSYLLPATTYHFRVMAENSFGKSDFSTDLEVPGGPPLNVQADA
VGSHTLRVTWGRLTGYYVGLKRLTTYSVIVQAFNRRGAGPPSPPTNPHTSIQIEWGFRLYSFEDLQCGTRYHFYMT
AFNCAITFFVIQYLSPGTWYRLHITAHNDAGTQAEYSFST

>Parasteatoda_9 aug3.g17489.t1

QRFNPVVHNGNTAVLKCPSSLKDIVFVESWCLTRNKLTLPCIAEGNPAPSYRWRIRINQGTLHIEKTANSDCGKYQC
IVRNTIGLVVQLSPGQEATFSCDKGMYQCQSTAELFPENIVHPGSLQCIATGNPTPQVRWYFESFLNASDIHLKDSG
EYQCHVTNGSLFHSSRVNVGEDLVIRCPYGGYPIKEIIWLPMNHRTGSLSIHSVDEGEYTCIVADEKGAKSTIHISVVP
PVLVDEGIRTKLMCVVTKGDPPIFIQWLKNDSILAFNKVSSSDGFYTCVASNMAAPPTWTIEGNTIWLDCGADGFP
APTILWKKNGTLVLSDLDESDSGSYLCQANNGIGSGLSKIIYLVKDPYKTKIINEYLICTANGLHITNATRDNGTYSCGV
DEAVTKMIVQGTPAPPSRSVYLYWDVSSHITGSLNPATAYFIRVIVQNSVGKSKPSTEEEVPSGPPLEVHIQSTGAHS
MRILWGILKGYYVGLKPFTEYDIFIKAFNSIGAGPDSP---

>Parasteatoda_10 aug3.g18884.t1

QRYDIHLFDGNMALLRCPSFVKDYVKVTSWCHAENAITLSCIAQGHPVPIYRWSVRQEGGVLIFNKVRISDAGRYS
CHVSNSVGLRVALLPGKTALFNCDSGMYQCQAAARLFPEKTVRPGSLLCISSGNPAPQIKWTLDSYVNITSTDVTDS

GVYTCTAFNGSTSHSRRLNVGGTFRVTCPFGGYPFDSITWLPANQRNGTLVINEIDDGRYSCEVRAQQGVSRTFRF
SITGPKVLHEGMRTAVTCIVVAGDGPLSTTWLKDGSTLTLKNLAYRHGNYTCIARNDVAPPRWIIEGRPARLDCQA
DGVPLPHVRWKVNGSLNFQSVDTADSGYYLCEANNGVGSGLSTVVRLFVSKYAMMTARKGMECIAVGLVIDKVE
KDSALFTCGDDSKNIQLTIQDIPDAPSRSVRVSWSKPSPILQYLKPKNRYFFRVKCENSLGESQFGTLEEPPRRTPHGI
KAFPLSSRSINITWS-IDGYYIGLSPLTDYNVIVQAYNGRGAGPPS-------------
GYILHHLHELQCGTSYSFYLVAFNCPITFFVIQYLSPGTWYTLFMTARNDAGTDSEYLFAT

>Parasteatoda_11 aug3.g20363.t1

QSFETQLFDGNMAVLHCPSYISDYVKVTSWCHMENSVTLACVAQGSPVPSYRWSIRQENGILIFESIKYSHGGKYS
CFVSNTMGLALNIIPGNEATFNCDSGMYQCQASAQLFPEKIVRPGSLMCVATGNPEPQFKWSMDSYVNITAVDIT
DSGLYTCEAFNGSVIHSRRLNVGEKFSILCPFGGYPFDSISWLPINQKNGTVQISEMDDGIYSCEVTYSQGVSRSFIVV
ITKPHVLHEGMRTAVACIVLSGDPPITIKWLKDGSTLTINTLSYKHGNYTCMATNKVGPPRWILQGQPAKIDCQAD
GVPQPHVRWKVNGSLNFRSVEKSDEGAYLCEANNGVGTGLSTVVNLFHTKFFVLTERRGLTCISYGLVIEKVERDTA
TFTCGEDSMNFQLTIQDVPDAPSRTVRLTWNRPSPILHYLSPKTRYFFRVKCENSLGESQYGTLEEPPRNAPLGVKAI
PISSTSINISWRNVDGFYVGLNRYTEYSVIVQPFNSRGAGPPSAPGIPSKTIKLSWGYILYNFQDLQCGTKYFCYIVAF
NCPIRFFIVQYLIPGTWYSLLMTARNDAGTDAEYVFAT

>Parasteatoda_12 aug3.g2126.t2

QKFVPQVYDGNTAVLRCPAFVREYVTIDSWCRARHRLTIPCVATAQPAPEYRWRTKITSGTLQISNVRLSDGGKYQ
CIISNGIGLRVTIIPGETAVLNCDKGMYQCQGTAQIFPDTTVHAGSLKCVSAGNFVPRVIWTLYSYLNISDTRVEDSG
VYRCDVSNGAVWHAAKLNVDQTLVLNCPVGGYPIKSIVWLPLDHRAGVLEIQDVDEGEYTCIAKSPDDASGSTFVS
VVAPVIANEGERAKIICSVTKGDPPIRFKWLQNGSIIQFRRIKASDGAYTCIATNAATPPTWLSEGSSAWMNCSAEG
FPTPSVIWRKNGSLVLWDVELSDKGFYLCQANNGIGAALSKVIFLFEETYQSRAIKEGLKCTAQGLVIVSSSRESGMY
ACGYDESTFQLVVQSVPDPPSRTATIRWDVPSHITGSLQPVTTYQLRIIAENSIGKSKPSTQVEAPSGPSRDLTVHPT
GDQSLKVSWGQIQGYHVGLQPFTKYEIIVKAYNVAGAGPQTPPTSPFNSINIKWEYTLHYLENAKCGTKYHMYLTA
SNCPILHFNIQYLTPDREYLLMVTAHSEAGTQGEYKFRT

>Parasteatoda_13 aug3.g23711.t1

QPYEVHVSDGSTAVLLCPPAVRSFIKVMLWCQVHHRLILPCVGQGYPQPSYKWRILHKDGVLIIQQAAIQDTGVYT
CELNNSVGLSAKIDPGKTATLSCDAAVYQCQGSAELFSEQAVYPGSLKCSASGNPLPQVTWLVDSYVNISSITVEDG
GSYQCLAQNKLIEHSKRLNVGQIITVHCPVSGYPISKVYWLPHNHRNGTLVIEDLDSGKYHCVAENNRSARREVSLN
IAPPVVLSEGKRATVTCVVSSGDLPIRISWMKDDSTLSFSSVSQIHGNYTCIASNPVSPPKWKTEGQAAMFDCQAT
GYPDPVIRWKKNGSLIIKESTKEDSGHYMCQATNDVGSGLSTVVFLFKEKFKALTLTRGLACRAMGLKISSVNRDSS
LFTCGRDDTNFQIVVQEPPDRPSRSASITWSAPSVIAKYLIPHMTYHVRVKAENAIGSGDFSTEGEAPSGPPRDLRA
VATNSRTIHVTWSPIDGYYVGLSKNTKYRVIVQAFNNKGAGPPSPPAAPASSIHLSWGYILYQFYGLQCGMQYQFY
LIAFNCAINFFVIQYLTPANHYLLLITANNDAGTEAEYQFAT

>Parasteatoda_14 aug3.g23720.t1

QNFEARVYDGNIGVLRCPSHVRDYVKVTSWCQVRNTLTIPCVAQGHPAPAYRWKHHLINGSLILRRVTPQDGGLY
VCVAKCDAGIKITAWPGSTVTINCDKGMYQCQGSAELFEELIVKPGSLRCSAVGNPLPQITWYSYSFINITRVEVSD
GGQYECRAINGVDSFSSIVHVGDILIVHCPVSGYPIQSIRWLPLGRRNGTLLIQGLDAGKYRCQVENDEGASVDVFL
GVVPPSIPQEGMRASVSCSVPTGDAPIRISWLKDGSTLIFKSLRQEHGTYTCVAYNDAAPPRWKVEGGTVTLDCTA
DGRPEPRVIWKKNGSLVIRDIEQDDDGRYMCEASNGVGTPLSTVVKLFKQKFVAQTVRTGLKCEAFGLTIHVVERD
NGLYTCGQDEKHNQLTVQERPDAPGRKAVLRWTKPTPIVKYLQPVSQYSFRLIAENVVGRSLPSTESEVPGAPPRN

VHAVATDSTTLHVSWGSIRGYYIGLRSNTKYIIVVQAFNDKGAGPLSPPMPPASSIGLKWGYVLHWFTGLNCGGEY
QFYITAFNCPILFFVVQYLTPSTWYSIMVTAHNSVGTEVVYETST

>Parasteatoda_15 aug3.g25152.t1

QEYQAHVYDGNTAVFKCPDFIKEFLTVTSWCLIKNTLTIPCTAQGYPVPQYTWRMLQVNGSLFIERARVQDTGKYI
CIVNNSIGLSVSLHPGRSATLNCDQGMYQCQSGAQLFKRQTIQPGSLRCVASGNPIPRVTWSLDSFVNVSSARSED
GGIYECRAQNGSVSHQERVDVGDSLFYQCPASGFPEPVITWLPFNERNGSFYIKDVDSGTYVCSAVNKQKAQREL
HIRVKRPVILAEGMHVIITCSVLTGDPPITIRWLKDGSSLVFSSVGRSHGNYTCLAENAAGPPRWRVEGESVTFDCQ
AEGYPQPLIRWKKNGSLSIKDLSVDDRGYYLCEAANSVGADLSTAVKLFKTPFRVHRVSKDVQCEAFGLTVISPDRD
SSLFSCGKDDTNIQVLVEEVPDPPSRGVTLSWSVPSPVTKYLYPTTTYQFKIQAENALGRSPFSTDEEVPSEPPLHVE
AIALSTHSIRVMWGKVRGYYVGLRRATEYSITVQAFNNRGAGPPSPPPSPTSAVVLSWGYKLFYLRSLNCGSRYQF
HIVAYNCPISFFVVQYLEPATWYNLLMSAHNEAGTEAEYVFAT

>Parasteatoda_16 aug3.g25153.t2

-----------------MAFLNKTKKV-----------VPFQA-------------------------------NVCEIKKIIQ--------------CERR----
ERQEELRREELERHE---EELERQHQLEM----------------RLELLHQN--
NGTLIIREVDEGKYTCIAENKDGSQKDVFVQVVGPKILEEGMRSLATCAVLSGDPPITIQWLKDGAFLRFTAVGPHH
GNYTCVAKNPAAPPFWRSQGESLTVDCQANGFPVPQVRWKKNGSLVITEVELADAGLYMCQATNGISPSLSAVV
KLFRVKFQSSTVRKGLICSAFGVTIISADRDSALFSCGRDETNFQVVVQERPDSPSRTVTLTWVQPLPLTRYLLPVTTY
NMRIIAENLLGQSKSSTKEEAPAAPPSDIRAEPLSSTSIKVSWGSITGYYLGLRRFTQYVIRLQAYNKVGSGPHSPPGT
PATSAKLEWGYILNYLQPLRCGTRYQVQISCFNCPISYFEVKYLTPGTWYNLMISAQNDAGTDAEYVFAT

>Parasteatoda_17 aug3.g25671.t1

QHYEIRLFDGNMGVLRCPSFASDYVRVTSWCHTEDFITLPCIAQGYPIPTYRWSVSQEGGILIFHKVVPSDTGHYAC
HVTNVMGYRVVIAPGKTATFNCDEGIYQCQASAVLFPEKTVQPGSLTCIASGHPEPQIKWTLDSSVNFTSVDVADS
GVYSCEASNGTESYSKRLNVGESFYGMCPFGGYPYESISWLPINQRNGTLLISETDDGTYSCEVTGTGQVSRSFRIVI
TKPKVLHEGMRTAVTCIVVAGDGPLSTRWMKDESTITIKSLTHKHGNYTCLATNDVGPPRWILQGRSARIDCQAD
GVPQPHVRWKVNGSLNFRNVEATDSGYYLCEASNGVGVGLSTVVRLFQSKFMVLSVRKGIECTVNGLAVERAER
DSSLFMCGDDTMNVQVTIKDLPDAPSRSLRLTWDRPSPITQYLKPKTRYFFRVKCMNPIGESQFGTLEEPPRSPPRY
VKALATSSKSLNVSWSNIDGFYVGLNRHSEYNVIVQSFNKRGAGPPSKPLPPYNSIKINWGFVLYYLHDLLCGTKYYC
FLVATNCPIRFYVIQYLIPGTWYSLLMTSRNDAGTDAEYVFAT

>Parasteatoda_18 aug3.g25674.t1

QKYEIRLSDGNMALLRCPSFVSDYVKVASWCHAENIVTLSCLAQGHPIPTYRWTVRQEGGVLVFHKVVSADGGTYI
CEVSNSVGLRVSLFPGKTANFNCDSGIYQCQASSRL-------------------
APQMRWFLDSYLNFTSVDVADGGLYHCEAFNGSVKHSKRLNVSSVFTVNCPFGGFPFDKISWLPINQRNGTMVIE
GVDDGRYSCEVTTGQGVSRTFRISVTGPKVLHEGMLTAVTCIVDSGDGPLATRWLKDGTTLTIRNLEYKHGNYTCE
STNDVAPPRWILEGRPAKINCQADGVPQPHIRWKKNGSLNFQSIEPSDEGYYLCEANNGVDVGLSTVVKLFQFKFK
VIGTRRGIECSCYGIIIEKAERDSALFTCGEDSMNIQLTVQDIPDAPSRNVRLTWSKPSPIVQYLKPKTRYFFRVKCQN
SFGESQFGSLEEPPRHPPEDLKALVLSSRTINVTWS-
IEGFYVGLNRFTEYSIIVQPFNSRGAGPPSPPGVPSTTIKVSWGYILRHLKDLQCGSTYYLSLVAYNCPVTFFIIQYLTP
GAWYSLLMTARNDAGTDAEYVFAT

>Parasteatoda_19 aug3.g26388.t1

QKYEIRLSDGNMALLRCPSFVSDYVKVTSWCHTENTVTLSCLAQGHPTPTYRWTVRQEGGVLVFHKVVPADGGTY
ICEVSNSVGIHVNIYPGKTANFNCDSGVYQCQASAKLFPEKILRPGSLVCVASGNPAPQIKWYLDSYLNFTSIDVSDS
GLYQCEAFNGSIQHSKRLNVESRFAVYCPFGGFPFDNIVWLPVNQRNGTLVIEGVDDGRYSCEVASSQGISRSFRIL
VTGPKVLHEGMLTAVTCIVDAGDGPLTTRWLKDGSTLTIKKLEHKHGNYTCASTNDVAPPRWILEGRPATISCQAD
GEPHPHIRWKKNGSLNFPSVEASDEGYYLCEANNGVGPGLSTVVKLFQFKFKVLGARRGIECSSYGMIIEKAERDSA
LFTCGEDSLNIQLTVQDIPDAPSRNLRLTWNKPSPILQYLKPKTRYFFRVKCQNSLGESQFGTLEEPPRHPPENIKATV
VSSRSINVTWN-
IEGFYIGLNRFTEYSIVVQSFNSRGAGPPSPPGVPATTIKVSWGYVLHHLKDLQCGSTYYIYLVAFNCPITFFIIQYLNP
GTWYSILMTARNDAGTDAEYVFAT

>Parasteatoda_20 aug3.g27791.t2

QRYEIYLVDGNMALLRCPSYVKEYIRVTAWCHTENIITLFCIAQGHPVPKYQWSAKQEDGVLKFSNVKLSDAGFYSC
QVTNSIGLKVSIIPGKDGLFNCDSGMYQCQATARLFPEKTVRPGSLLCITSGNPTPQVKWTLDSYVNITSADVMDS
GLYSCVSFNGRSVHSRRLNVGGTFKVFCPFGGYPYNDIIWIANNQRNGTLLISEMDGGLYSCEVRSSQSVSRTFRISI
NAPKILYEGKRTAVTCIIVEGDGPISTTWLKDGSTLTFKNLTYNHGNYTCVARNEVAPPRWIIKGRPVKIECQADGVP
LPHVRWKMNGSLNFQNVEILDSGYYMCEASNGVGSGITAVVYLFHTKYAMMTVRRGMDCEVVGLVIEKVEKDS
ALFSCGDDSKNIQLTIQDIPDAPSRSVRLSWSKPSPIIQYLKPKNRYFFRVKCENSLGESQFGTLEEPPKRAPYGVKVS
AVSSRSINVTWS-
VDGFYIGLSPLTDYSVIVQSYNSRGAGPPSKPESPTKSIKISWGYILCHLHDLQCGTSYSFYLIAFNCPITVFVIQYLIPGT
WYTLLITARNDAGTDSEYIFAT

>Parasteatoda_21 aug3.g2782.t3

---GN----
FRWRVWLVKGTLNFRKVLRTDAGKYQCIVRNSIGLQVSLLLGQEATFTCDRGMYQCQGTVELFPEQSVHPGSLKC
VTTGNPPPKVTWYVDSFLNISDTRVEDGGEYKCQVSNGSTFHSSRLNVGEELLIRCPYGGYPIKSIRWLKDTER----
SIPILD----SAIQKSI--SRGALGYKAAPP--
VDEEARTKLVCIVSKGDPPLRFHWLKNGSIMAFKRITSADGHYSCVASNVASPPRWTVEGNTVWMDCSAVGFPS
PSVLWKKNGSLVISDAEESDAGNYLCQANNGIGAGLSKIVTLFKDSFQSRSTREGLKCDSSGLHIVNATRDSGKYSC
GMDESIIQFIVQGFPDAPSRSVSLFWEITSHITGSLNPITLYFVRVIAENSVGQSLPSTEEEAPSGSPKDVQVQSTGAQ
SIKVTWGRIKGYYVGLQPFTKYEIIVKAYNSAGTGPKSPPTSPSSSITVHWEYSLHYLKGLRCGTRYQLYMTASN------
----

>Parasteatoda_22 aug3.g2979.t1

QRFNPQVYDGNTAVLRCPTFVKEYVTVDSWCQTRHRLTLSCVAQGNPPPAYRWRVWLVQGTLNFRKVLRTDAG
KYQCIVRNSIGLQASLLPGQEGTFTCDRGMYQCQGTAELFPEQTAHPGSLKCTSTGNPPPRITWYLDSFLNISDARV
DDGGEYKCQVSNGSTFHAARLNVGEDLIVRCPYGGYPIKGVRWLPLNHRGGTLTIQSVDEGEYSCVVRSMDGATG
ATFVSVVSPVIVDEGARTKLVCIVTKGDPPLRFHWLKNGSIVAFKRVTSADGMYTCVATNVASPPQWTVEGNTVW
MDCASVGFPAPSVLWKKNGTLVISDAEENDAGAYLCQANNGIGAGLSKIISLFKDSFQNRATKEGIKCDATGRNTA
Q----------------------
SFPDAPSRSVSLIWDVASHITGSLTPVTLYFVRVLSENAIGQSKPSTEEEAPSGTPKDVQVHSTGAQSIKVTWGHIKG
YYIGLQPFTMYDIVVKAYNSAGSGPKTPPTSPSSTISIRWEYNIHYIHNLKCGTRYQLYMTASNCPIKHFMVQYLAPD
RDYDVMVIAHSDAGTQGEYAIRT

>Parasteatoda_23 aug3.g3156.t1

QHYVVEVYDGSTAVLRCPGFMEEYVSVISWCQTRNRLTLPCVAHGFPLPSYRWRISQLEGTLIIQQATVADSGHYV
CLTNNSLGLSTSMSPGRPWIINCDRGMYQCQSAVELFEEQTIQPGSLKCVASGNPLPQIVWRLDSFVNVSNVKTE
DGGDYRCVANNGEVSHESRVNVGDVLVLRCPVAGYPLENIHWLPYNHRNGTLEVHHVDQGPYVCVATNKAGAQ
STVIVRVVKPVILREGQRASVLCTVSSGDLPFKIRWFKDGSTLLFESLALHHGNYTCVAENDAGPPRWVIEGKNIVV
DCQTEGFPQPRVRWTKNGSLSITDATESDAGYYLCQASNGIGQGLSKVVRLFLSKFTAEMVHKNLKCEAIGLLFRQ
YSKKTSHVICGRDDGNITVIPENTKKYI-----------------
SFRFMAKSLDQLAKNLSEDQFRLSSQVPLLLQKRVYPYDYICHPDKFAETNRLNEVSITFRDVCMKTYELDPTWYYT
TPGLSLELLVESGIRGGINPY---DANNLYGWTQSHSVGYILLYYRNLQLYL-----------------SIDLNTTLRT

>Parasteatoda_24 aug3.g4548.t1

QDFDIHLFDGNTAILRCPSYVNDYVRVRSWCHTENRITLPCIAQGFPAPSYRWPVSQEGGILVFHKVTSTDSGRYVC
HVTNVIGLRVSVSPGKTATFNCDQGMYQCQASSQLFPEKTVRSGSLTCEATGHPQPTIRWTLDSSINFTSVDLTDS
GIYTCQARNGESSYSNRLNVGETFKVNCPFGGYPYESIAWLPDSQKNGTLLIKEMDDGVYTCEVRGSAQASRSFRV
VITKPQVLHEGMRTAFTCIVLAGDSPMTIQWLKDGSTLTIKSLAKKHGNYTCLATNDISPPKWILKGRAAMIDCQA
DGVPQPHIRWKVNGSLNFRGIEKSDSGYYLCEASNGVGSILSTVVRLILPKFLIMSIKKGLDCQATGLTIDKTERDSSL
FICGEDSMNIQVTIQDLPDAPSRSIRLTWDRPSPITEYLKPKTKYYFRVKCSNSIGESQFGTLEEPPRLPPIFVTSKATSS
KSVNVTWSNYDGFYIGLNRNTDYSFIVQAFNQRGAGPPSRPLPPTNSIKIIWGLTIYHLHDLLCGTKYYCFIVASNCPI
KFYIIQYLIPGTWYSILMTSKNDAGTDAEYVFAT

>Parasteatoda_25 aug3.g5120.t1

QSYEIRLFDGNMALIHCPNSVSDYVRTVSWCYTQNSVTLSCLAQGYPVPRYRWSIKLFGGVLIFHKPLPIDSGLYVC
HVSNTMGLQVTVTPGRTATFNCDSGMYQCQSSARLFPEMTVRSGSLTCIATGNPEPRIRWLLDSYVNFTSVDVKD
SGLYTCEAINGQVMHSKRLNVGAGYAMTCPFGGYPFDVIKWLPLNQRNGTFLITEVDKGLYSCEVSSSNEVVQTFR
LDIAGPKVLHEGMRTVANCIVLGGDGPLTTRWLKDGSILTFKNLTHMHGNYTCVVTNDVAPPRWILRNKPARIDC
QADGAPLPHVRWKMNGSLNIRSVQGSDAGFYLCEANNGVGSGLSAIVKLFVTKFLVITVRRGIECSPEGFSIEKSER
DSALMTCGEDSINIQITVQDIPDAPSRSVRLTWKKPSPVTRYLRPNTRYFFRVKCENMLGESQFGTLEEPPSNSPQF
VKAEPISSKSINVTWS-
MDGFYVGLNRNSEYSIVVQAYNSRGAGPPSRPSAPSRTLKLSWGYILHYLHDLQCGTSYSFYLVAFNCPISVFIIQYLS
PGSWYSMLITAKNDAGTDAEYIFAT

>Parasteatoda_26 aug3.g5125.t1

QNYEIRLFDGNMAILRCPSSVSDYVRVVSWCHTENILTLSCIAQGHPVPRYRWSARQDGGVLVFHKTVSSDSGRYV
CHVSNSMGLKVSIVPGRTATFHCDSGMYQCQSYSHLFIEKTVRPGSLTCTATGNPEPHIRWKLDSYVNFTSIDIIDSG
AYSCEAINGRAQHSKRLNVGASFEMICPFGGYPYDTISWLPINQRNGTFQIVDVDNGQYSCEVSSNQGASRSFRIS
VSGPKILHEGMRTVATCIVLGGDGPLAAKWLKDGSILTFKNLTHRHGNYSCVVSNDVAPPKWIIQGRPSQIDCQAD
GVPQPHVRWKMNGSLNFRSVEASDAGYYLCEANNGVGSPLSAIVRLFLTKFLVISARRGIECSPEGLTIEKAERDSAL
ITCGEDSINIQVTVQDIPDAPSRSVRLTWKRPFPITRYLKPNTRYFFRVKCENALGESQFGTMEEPPSVSPQYVKATA
ITSKLINVTWS-
IDGFYVGLNRYTEYTVVVQAYNGRGAGPPSPPNAPSKTLKLSWGYILHHLHDLQCGTTYSFYLVAFNCPINFFIIQYL
SPGSWYSMLMTAKNDAGTDAEYIFAT

>Parasteatoda_27 aug3.g5556.t2

QRYAVEMHDGNTAVLKCPGFVKDYVIVEAWCRTKHRLTLPCAGQAFPLPNYRWRREMVGGTLILRNAEIRDSGR
YICVLTSSGNLSVSVYPGEVVTFNCDHGLYQCQGAAQLFENMTVNPGSLKCSASGNPLPQITWSLDSYVNISRAGV

EHSGEFTCAAKNGEVEHSSWVRVGEMMKIRCPAGGYPIHRISWLPDNHRNGTLLVRQVDEGRYTCTLRNKEGAK
GSVYITVVRPVIVQMGQRLSISCTVIKGDPPITLKWLRDESTLLFESIKPEHGNYTCVASNEAGPPRWKTEGRSIMID
CEADGFPPPRLTWRKNGTLTILDVGEHDAGYYLCQASNGIGSGLSKVISLFTNKFHAEIVKKGLSCQAYGLLIDLVDR
DSALFTCGKDETNIQLIIQEKPDIPSRSVDISWSPPSRLLKYLTPDTRYFILISAVNALGESEST-----
PGGPPLAVKTLPLSSTAVKVLWGQIKGYYVGLQRNTKYLVRVQAFNNKGAGPPSPPKPPSTSVTLKWTKGFIVLINL
QCGMRYEFYIT--NCPITHFEIKYLTPNTFYQLQLTSHNEAGTEAEYIFTT

>Parasteatoda_28 aug3.g6282.t1

QRYEIHLNDDNMGLLQCPSFVKDYVRITSWCHSENVITLSCIAQGIPVPVYRWTVKQEDGVLKFNKVKPSDAGQYS
CQVTNTVGLRVNIFPGKSGLFNCDSGMYQCQASARLFPEKTVRPGSLLCISNGNPTPQVKWTLDSYVNITSADVTD
SGVYTCTVFNGSTSHSRRLNVGGSFRMSCPFGGYPYDEIAWLPTHQRNGTVLITDIDGGLYSCEIRSPQGVSRTFRIS
ITGPRILHEGMRTAVTCIIIAGDGPLSTTWFKDSSTLTLKNLSYRHGNYTCVARNDVS-----------------------------
NGSLNFQSLDTLDAGHYLCEASNGVGSGLTAVVRLFHSKYMMMTIRRGMECEVSGLVIEKVEKDSALFTCGDDSK
NIQLTIQDIPDAPSRSVRVSWSKPSPIIQYLKPKNRYFFRVKCENSLGESQFGTMEEPPRKAPHNVKALALSSRSINVT
WS-
IDGYYVGLSAMTDYSVIVQGFNSKGAGPPSKPDTPSKTIKISWGYILHYQRDLQCGTSYSFYLITFNCAIKFFVVQYLIP
GTWYTLFITARNDAGTDSEYVFAT

>Parasteatoda_29 aug3.g661.t1

QNYEIHLVNGNMALLRCPSYVTDYVKVTSWCHTENEITLSCIAQGFPPPTYRWSVSQEGGVLIFHKAQRNDSGLYK
CHAVNGVGIHASLVPGATSTLNCDAGMYQCQASARLFPEKTIRPGSLTCIATGHPEPTIKWILDSSINFTSVDVTDS
GLYTCEAVNGSVSYAKRLNVGEGFQSLCPFGGYPYDSIIWLPINQRNGTFLISGMDEGIYSCEVTSGLSVSRSFSVMV
TKPKILQEGMRIAVTCVVIAGDGPLSTRWFKDGSTLTIKYLTHKHGNYTCIAKNDVAPPRWVLEGQSAIINCQADG
VPQPYVRWKVNGSLSFRNIESTDAGFYLCEANNGVAVGLSKVVRLFNSKFQFVSAKKGIECFAMGLIVEKTQRDSA
LFECGEDVMNIKVTIEDIPDAPSRSIRLTWDKPSPINQYLKPKTKYFFRVKCLNLIGESQFGTLEEPPRVPPRLVKAIGT
SSRSVNVSWAHVEGFYVGLNRKEEYHFIVQSFNKKGASPPSRPLPPSSSIKVVWGFILRHLYDLLCGTKYHCSLVATN
CFIRFFIIQYLTPGTWYSLLITARNDAGTDAEYVFAT

>Parasteatoda_30 aug3.g7222.t1

VKYEIRTYDGNTAVLKCPSSVKDFVPVVSWCICKDEMNLFCIAQGYPVPTYSWRLQVLRSTVQIHKASIVDSATYICV
ANNSAGLRVSIYPGISVTFTCDKGMYQCQGSAQLFSDHILRPGSLHCLVTGNPLPQVSWRLYSFINISQITTEHGGIY
SCHARNGAISHTARISVGDEVNVDCALSGHPIRSVRWLSGGQRNGTLSIAQADQGWYHCEVIGNQAATGSIFIRV
ERPVILMEGMRTMVVCTVLAGESPINILWLKDSSSLTIPSVSRYHGNYTCLVASGTASPKWVKKNQKVVFACQAEG
IPEPIHRWKYNGSLVIRDVEKQDSGLYLCEASNGVGAALAEVIKLFNKSFEVKTVKEHLTCDAHGLSIPAVLRDSGLFS
CGREEKSIQLIVQAPPETPSRQITFSWTAPSPITGYLNPGTTYFFRVHAENILGRSNPSTEEEAPSISPQDVRAIPISST
MLKVTWASLLGYYVGLRRNTKYAITVQGYNSKGAGPAAPPKPPEFSIELHWGYVIHYVKNLKCGTKYHFTITAFNCP
ILYFEIRYLTPGTWYDLLMTAHNDAGTEAEYVFST

>Parasteatoda_31 aug3.g7225.t1

EVYEISVQDQNTALIRCPSSVSSYVQVSAWCQVTNSLTLPCVAHGHPSPQYYWKSTIQDGTLVIHKVSATDSGKYIC
FANNSVGLKVDVVTGEEVSLNCDSGLYQCQAHVQLFPDQIHMPGSLHCSASGSPIPQILWFLDSYVNITDLAVSDG
GLYGCEAQNGLLSHSARIDVGTTVSIRCPVSGYPIDRTYILPYGDRRGVVRIHNVDEGIYRCIATNGRGAERPLVLKVT
APLILEEGMRSSIICSVIAGDPPISLTWYRNSSSLIINHVSRHHGNYTCFASNSAAPPVWVIKGMSVTFDCQAEGQPR
PVVRWKFNGSLTITVAQKGDAGYYMCETSNDVGEPLRYSVRLIQTSTQVVHVRKSFSCSASGVIIKSSQRDSDVFTC

GEDKTTVRLVVQEPPDPPSNSISLSWTPGNPITSYLRPVTTYHIYVNARNGIGQSLPSTNAEAPRTPPRHVKAIPLDS
SSIRISWSYVDGYYVGLKRGTQYSIVVQAFNSQGAGPPSPPDAPATSVNLNWGFILTYLENLQCGTSYQIVLTAYNC
PISHLVIQYLSPGTWHDLLVTAHSDAGTDAEYRFAT

>Parasteatoda_32 aug3.g7229.t2

QFYETQVYDGNTAVLKCPSFVKEYVSVMSWCQTKHRISLPCAAEGYPVPSIHWRYVQLGGTLIIRRAEPEDSGKYIC
YVNNSVALKVSVTPGESIIFNC---
DLLQVAPIVREDKGMYQCFVTNGMSQGSAELNL--------------------
LPQNRRNGTLIINTVDEGSYRCIAENKDGAFRDVEVKVVAPVILKEGNRVIITCSILDGDTPINLNWYKDNSVLYMKR
VTYENGNYTCVASNRADPPQWKIQGQNVIIDCQADGFPQPRIWWEKNGSLMIRESEQNDAGYYLCQATNGIGS
GLSKVIELFKGKFVAQTLKKGLFCEAFGISIHSVDRDSALFTCGQDDTNLQLIVQEAPDSPSRTITIRWAAPSPIVSYLR
PVTSYHVRVMAENGIGWSEPSTAEEAPEGVPTAVHAVASSSNTIHVSWGNIKGYYVGLRRATKYSIIVQAFNAKGS
GPPCPPVSPTSTVQLKWGKILKXFQDLYCGSHYQFYIVAYNCPVNFFIVQYLTPATWYTILMTAHSEAGTEAEYVFA
T

>Parasteatoda_33 aug3.g9149.t1

QPYAVDVYDGNTAVLKCPGLVQDYVTVTSWCETRHRLTVPCASQGFPIPTYEWRFLQLDGTLLLREAKIEDSGHYL
CKVHNSAGLSVQMIPGKSATFNCDKGMIQCQGTAQLFNDQTLEPGTLKCVAQGNPLPQITWTLDSYVNITSVRPE
DGGLYKCVATNGEASHSDRLNVGETLRLQCPVGGHPIENIKW--------------------------------------
VKPFILQQGQRFSVLCTITEGDQPIRIQWIKDGSSLVFESLAPEHGNYTCVASNGAGPPRWRIETRSAVVDCQADGF
PIPRIRWTKNGSLVIHSVEEGDDGHYLCQATNGIGQGLSKVIHLFKSKFRAESVRKGLICEAIGLTIRRADRDSALYTC
GQDDTNIQLIMQEPPDAPSRSAKIGWAAPSQISQYLLPITFYHFRLYAENALGRSEPSTDQEAPGGPPQKLKARAV
GAHIVKVSWGIIKGYYVGLKKFTEYAVIVQAFNAKGAGPPSPPKSPHSSIGLSWQGYVLYLYDLRCGSRYQFYIAAFN
CPLKSFVVRYLSPGTWYRLQVTAHNEAGTDAEYTFVT

>Parasteatoda_34 aug3.g9642.t1

QVYEIQLFDGNIAVLHCPSYVSDYVKVASWCQVENSITLPCISQGHPVPNHRWLVRQENGILIFEKIKSSHAGKYVC
YVSNTMGITATIFPGKNATFNCDAGMYQCQASAQLFPEKIVRSGSLMCVATGNPVPQVKWSMDSYVNITSLDITD
SGLYSCEALNGRTVHSRRLNVGEEFSVLCPFGGYPFDSIIWLPVNQRNGTLKISEMDDGIYSCEVLYNQGAIRTFSIVI
TKPNILHEGMRTAVTCIVVAGDPPITTRWLKDGSTLTLNTLAFKHGEYACMATNDVGPPRWILQGHSAKIDCQAD
GVPQPHVRWKVNGSLNFRNVEKSDEGSYLCEANNGVGTGLSTVVRFFETKFSTLTVRRGLNCISSGLIIEKAERDSA
SFTCGEDSMNFQLTVQDVPDAPSRSVRLTWNRPSPILRYLNPKTRYFFRVKCENALGDSQYGTLEEPPREATIGVKA
IPVSSRAVNVSWGSVDGFYVGLNRNTEYIIIVQPFNSRGAGPPSAPGMPSKTVKLSWGYILYKFQDLQCGTKYYCYV
IAFNCPIRFFIVQYLIPGTWYTLLMTARNDAGTDAEYIFAT

>Parasteatoda_35 aug3.g9655.t3

RRYKVQVYDGNTAVLRCPSFVHDYITFM-
WCQTRHRLTLACAATAFPPPAYRWRITQLDGSLILQFVSVQDSGRYVCSVNNTAGLSVYVTPGRSATFNCDKGMY
QCQSSAELFANEAIRPGSLRCTASGNPLPQVTWFLDSYVNITDTHVEDGGEYSCSASNASVRHTARLDIGHTIVVKC
PISGFPIENVFWLPINHRLGRLVVQDIDEGFYSCEAIGPDNAGGKMHMSVVAPTIAKESTKTKVMCSVSQGDPPIQI
MWRKNGSILMFRRISSKHGNYTCFASNMAAPPRWKVEGKTVVMHCSSDGYPSPRIYWKKNGSLALQEVTEADG
GHYLCQATNGIGAGLSKVILLFETKFVSHTVTKGLKCEAEGLIIKSATRDSALYTCGNDQTNIQLVVQEPPSPPSRTM
TLAWSPTSPVMKYLLPSSTYKFRVIAENALGVSEPSTEEEVPGGPPLEVNVQPTGSQSLKVTW---------

FKCLQSSHIGLKALTT--------------------
XYILHYLEDLRCGTGYRLYMTATNCPILYYSIHLLTPSREYTIRVSAHSDAGTEAEYRFMT

>Drosophila_1 gi_PIPE_8072216_PIPE_gb_PIPE_AF260530.1_PIPE_

QYYEADVNKGNSAVIKCPSFVADFVEVVSWCRTKHRLTILCPAQGYPAPSFRWRVKQVSGTLIIKDAVVEDSGKYL
CVVNNSVGLSAKIDPGRPAVFTCDKGMYQCEASAELFQEETMEPGFLKCVAGGNPTPEISWELDSYLNITSVHAN
DGGLYKCIAKSGVAEHSAKLNVGETLIVTCPVAGYPIDSIVWLPINRKNGTLIIENVDQATYTCVAKNQEGARGSLEV
QVVPPQVADVGDIASANCVVPKGDLPLEIRWSLNSSLLNIDSLNAFHGVYKCIATNPAGPPRWILEGSDAKVECKA
DGFPKPQVTWKKEGTLHVDNIQKTNEGYYLCEAINGIGSGLSAVIMIFTEKLRNQTARRGLQCEAKGLSIKRTERDS
ALFTCGSDDASINMIVQEVPEMPGRSVQLSWAQPSPLDRYLSPATTYNIRIVAENAIGTSQSSTAEEAPSGKPQNIK
VEPVNQTTMRVTWGEILGYYVGLRVYTQYSVVIQAFNKIGAGPLSPSQPPSQTIRVGWTYKVVYIEGLLCGSRYQV
YATGFNCPMSHFVVESLEPATWYNLRITAHNSAGTVAEYDFAT

>Drosophila_2 Dscam2-PN FBgn0265296

QAYKVDVEVGCTAILRCPTFVKELVRVVSWCRSMHRLTLLCVAQGCPSPEYSWRVRLLGPILAIEAVTGEDSGVYKC
TAGNVGGIQVEISPGGTAEFRCNRGMYQCQATAELFIEQTLQPGSLKCSAAGNPTPQISWTLDSHVNISHVMVED
GGEYACIAENGRVQHAARLNIGETLNLKCPVAGYPIEEIHWLPDDIRDGSLTISPVDSGVYTCWARNKQGARRSGE
VTVVPPKLLNMGDRASLTCSVVKGDLPLTINWRKDGSILVIENLGSDHGNYSCVVRNSAAPPRWIVENRHIMLHC
QAQGVPTPSIVWKKNGSLLLQHVKEDREGFYLCQANNGIGTGIGKVIQLFSSTSRSVMVKKGLQCAVSGLQIRTVD
ADSGPYFCGNDQQLVQLQVQEPPLPPSRSVNIKWQPKGDVTKYLKPATRYAFRVIAEGSAGRSAPSTEPQRPAGP
PLSLSARPLSSTELLISWGDIQGYNVGLAKFARYTVVVQAFNQVGPGPLSPSRPPSQSLQVSWGYKLIFLKGLMCGS
TYQIHLSAQNCPLLYFVLQYLQPSTLYQLRMEAHNVAGSQAEFNFVT

>Drosophila_3 Dscam3-PE FBgn0261046

RQFHVHVENGNSALIKCPEYVRPYVRVASWCLVTNTLNLPCNIQGNPFPIFTWRVILSRTLLLIKNADERDAGKWIC
QASNQFGVSVHILPGGTANFNCDRGVYQCQAMAELFIEQNVRPGSLKCSASGSPPPQFAWLLDSHLNISHVRPDD
GGLYKCVASNGSVQHSARLNVGEDIIVHCPFAGYPVEQIRWLTTSNHGGQLVIKNVDQGIYTCIVRSRAGARRDM
QLNVSPPVILQEGGRAQITCAVSSGDMPIYFSWKKDDSLLVFKDISARHGKYTCYASNAAAAPRWRYEGNTISINCE
AEGYPIPTITWFKNHSLLLNLATDNDEGYYMCQATNEIGAGLKKTIRIFEQSARNISSRRNLDCHAKGLTIGHSDRDS
GVYRCGRAEQIIFLAVQERPDTPSRTVKLSWRRPSPVLSYLTPATTFLIRMQAINEIERSAYTTQEEAPTEAPSNVQV
QTGGESELIVTWGELIGYTVNLRKYSRYAVTIRAMNSFGSGPWSPEAAPSQSLKISWGYKILYISGLKCGNQYIIKMS
AHNCSIHHFSIEHFLPAKWYQLRISATNDAGTTEHYHFST

>Drosophila_4 Dscam4-PJ FBgn0263219

QKYAVQVHDGNTAVLKCPSYMSEFVLVTAWCRTVNRLTLPCIAQGHPVPTYRWITIVSAGLLKITKARLEDSGKYLC
WVNNTAGLTAHLQPDKDAQFQCDPGMYQCQSTAELFSEQTLQPGSLKCVATGNPLPQFTWSLDSHVNISNVKE
EDGGEYTCTAQNGKVSHSAKVNIGSDLIVKCPVAGYPIDKIHWLPINRRNGTLIIEQLDAGTYTCMAQNKQKSRRN
VEIQVVPPKILREGMRAAISCQILEGDLPVSFRWERNGASLVIEHISSDHGNYTCIASNVAGPPKWILEGADVLLHCQ
SSGYPTPTITWKKNGTIFFKKISKESQGHFLCEAKNNIGSGVSKVIFLFQTKTKQISVAKGVQCNVQGLGISHTYRDT
GIYICGQDEMSIQLIVQEVPEQPSRSLQLTWSQPSPIEEYLRPAKAYHIRMSAENKLGASEFSTLEEVPSGPPLAVRA
EPKSSTEIFVTWGILLGYYVGLNKFTQYHVIVQAYTSQGSGPPSPSSPPSTSIYITWGYKVFYLTNLWCGTRYQLYITA
YNCGILYFMIESLVPGTKYQLKVTAHNNAGTTAIYNFTT

>Aplysia.Californica

TNYEVGLNDGGTAVLKCPLYVTDYVTVIGWCITRNVLTLPCVAQAYPTPAFSWRLAQKGGNLLVRNATVYDSGDYI
CTGVNSHGLSVLIEPGHIAQYNCDQGMYQCQGAAQLFREQFVRQGTLHRRFSGNPIPQVSWSQDSYVNISRVEV
ALGGEYTCYASSGQDRRLNKVNVSKDLSLRCYVSGYPLESIHWLPINHRNGTLVIRHVDSGKHTCVARNREEMDRS
MHIAVEPPVIRKQGDRILVTCAIISGDLPITIQWETDDTPFCPSTERCRNGNYTCYVSNAAAPPRWVVEHESVQLDC
QADGTPAPQVVWKKNGTLVIRQAREEDHGYYLCHASNQVGFDISKVIYLFNEPRRNYTVMKGMECQAVGLTLNP
TSRDSGFYTCGNGVLVNYLIVHEPPEPPSRSVMVSWSKPSPIIGYLLPSFAYHIRVLANNSVGYSEPSTEEEAPTGPP
DSVTVQAIGSQALKVIWEKILGYYIGLKKFTQYTVHVKAYNVKGISPASPSQPPSESIKVAWGYKILYANNLMCGTQ
YKFTVHAFNCPIRFFSVKYLFPATWYIMEVTAHSDAGTASQLKFAT

Alignment supporting figure 8, Argonautes

>Drosophila DrosAGO1

GREGRPIVLR ANHFGYVHHY DINIPVFDGR NNLYTRDPLP FRVTIKVSLF NLEEALEGAI

LALDVVPVGR SFFSSPLGGG REVWFGFHQS VRPSQWKMMD VSATAFYKAQ PVIDFMCEVL

LTDSQRVKFT KEIKGLKIEI TRKYRVCNVT RRPAQMQSFT VAKYFLDKYR MKLRYPHLPC

LQVGQEHKYL PLEVCNIVAG QKKLTDMQTS TMIKATARSA PDREREIDSY VQEFGLTISN

SMMEVRGRVL PPPKLQYFAP QRTVREDALR NFTQQLQKIS NDAGMQLVVV VLPYAEVKRV

GDTVLGTQCV QAKNVTSPQT LSNLCLKINV KLGGINSILV PSIRPPVIFL GADVTHPPAG

DNKKPSIAAV VGSMDAHPSR YAATVRVQEI IQELSSMVRE LLIMFYKPHR IILYRDGVSE

GQFPHVLQHE LTAIREACIT FIVVQKRHHT RLFCAEKKEQ SGKSGNIPAG TTVDVGITHP

FDFYLCSHQG IQGTSRPSHY HVLWDDNHFD SDELQCLTYQ LCHTYVRCTR SVSIPAPAYY

AHLVAFRARY HL

>Drosophila DrosAGO2

GTIGKPGQVG INYLSVAYHY DVKILAYDGK ASCYSVDKLP YTIEIKIDLK SLTTYMNDAM

QCVEVVRVGR SFFKMSLDDG YEALVGLYQA FMLGD-RPFD ISHKSFPISM PMIEYLERFS

NLDYSRRFLE PFLRGINVVY TRVYRVNGLS RAPASSETFT IASYFHSR-N YPLKFPQLHC

LNVGSSIKLL PIELCSIEEG QRKDGATQVA NMIKYAATST NVRKRKIDPT ISRFGIRIAN

DFIVVSTRVL SPPQVEYPRS GRKMNYTQLN DFGNLIISQG KAVNIDLAIV IIPYDTIKQK

AELQHGTQCI KQFTVCNNQT IGNILLKINS KLNGINHKIK DDPRLNTMYI GADVTHPSPD

QREIPSVVGV AASHDPYGAS YNMQYRLQEE IEDMFSITLE HLRVYKYPDH IIYYRDGVSD

GQFPKIKNEE LRCIKQACIC CVIVVKRHHT RFFPSGDVTT SNKFNNVDPG TVVDRTIVHP

MQFFMVSHQA IQGTAKPTRY NVIENTGNLD IDLLQQLTYN LCHMFPRCNR SVSYPAPAYL

AHLVAARGRV YL

>Parasteatoda aug3.g12796.t1

GGLGRKINLI SNLFRNIYRY EVKIAVYDGK SLIYTSSPLQ FKVYIKIQLR VLRELFRRAL

VALETIPIGS NFFRTNQLVG KVTLFGYHQS LRLGQWNIMD TSATTFFTKQ PLVEYIAKLL

LRNDQLQLLQ SKLKNLKIET YLKHSIFKIT EKNAWQIQFT VHEYFREKYN INLAFPHLPC

VQVKPKSQYL PIELCYIPEG QNELSDDEKR EMIKFTADNP ASRFHKIDEY LNNFDIVVER

DPVRLSGRAM QAPNLNYLA- ERHCRFNDLK NFSDQLQRIG RAAGLQLVVV VIPYRNVKQI

AEVKLGTQCI DHNNACNPSL LGNLCLKINA KTGGINHVLT QGEIPPVMII GADVSHAGVT

DKSGISVAAV AGSLDMILSR FAVEYRLQEI ILELKDMVKA LLEVFYKPEK IIFYRDGVSD

SYFQEVKEEE VNAVRRACIT FVVVQKRHHV RFRPEDFRDG ARPEGNVPPG TVADTEIVHP

RDFYLCSHLG LRGTSRPAHY TVLEDDSEIS ADDLQKLTYY LCYTSLRHSK STSAPVPVTY

AELTAKRTLL WL

>Daphnia DappuP311791

GTLGRPIKLS ANHFPILYHY DVEVPVFDLK KNIYTARRIP FIISLQIDVG ALASYCQGPI

QALDIAMLGS CLLSKPLGGG VEVWFGHFQS LRLG-WKPFD ATQRAFLRSG LVHDIMADMF

LDDRDYGDFH KKIATLKVSY NATVGCNGI- KGAANTEKFT VQEYFEKKLN TKLKYPHLPC

VWVGSREKLV PMELCSIAEG QRKLTDFQTS AMIKVAATPA DVRKRKIDQY AQHFGISVDT

QMAKIQGRVL PTPKLVYIT- --RCGDREID FFISALTKAG REMGMQIIFV IINYEIVKRV

GDLDLKTQCI QQKNVPDPST MANICLKLNA KLGGINNLIS RDFRPQVIIM GADVTHPGAD

DSGKPSIAAV VGSVDPRASQ YCCEIRIQEY IEDMENMVYN LLRKFNKPQR IIFYRDGVSE

GQFAKVLEWE LSAIRKACVT FIVVQKRHHT RLFPEDQRDE CGRGKNVPPG TIVDNTIVHP

QDFFLVSHQG IQGTSRPTHY HVLWDDSKFQ ANDIQMLTYY MCYLFTRCTR SVSYPAPCYY

SHLVAFRGRQ YY

>Daphnia DappuP305022

GREGRPIMLR ANHFGYIHHY DISIPVFDGR SNLYTRDPLP FHVAIKVSLY ALEEALEGAI

LALDVVPVGR SFFSSPLGGG REVWFGFHQS VRPSQWKMMD VSATAFYKSQ PVIEFMCEVL

LTDSQRVKFT KEIKGLKIEI TRKYRVCNVT RRPAQMQSFT VAKYFLDKYK MKLRYPHLPC

LQVGQEHKYL PLEVCNIVAG QKKLTDMQTS TMIKATARSA PDREREIDPY VQEFGLTISN

SMMEVRGRVL PPPKLQYFAP QRTVREDALR NFTQQLQKIS NDAGMQLVCV VLPYAEVKRV

GDTVLGTQCV QAKNVTSPQT LSNLCLKINV KLGGVNSILV PTIRPPVIFL GADITHPPAG

DNKKPSIAAV VGSMDAHPSR YAATVRVQEV IQELSAMVRE LLILFYKPNR IIMYRDGASE

GQFSTVLQHE LTAIREACIT FIVVQKRHHT RLFCADKKEQ SGKSGNIPAG TTVDAGITHP

FDFYLCSHQG IQGTSRPSHY HVLWDDNHFD ADELQCLTYQ LCHTYVRCTR SVSIPAPAYY

AHLVAFRARY HL

>Strigamia SMAR010593

GKRGRPIILS ANHFARIYHY DIEICVFDGE KNLYSNHPIP FTVAIKVSLK DVQAFLKGAI

QALDIVSIGR SLYPDPLGGG AEMWRGYYQS LRPGKWKDLD VSNRSFYKAQ PALEFAVDFF

LPPRRWDSFS KEIKGVRIEV TRKYRLIGIG K-PASEHRFT TEKYFAFVYN YKLRHAILPT

LQAQPSAKFL PMEVCIILAG QKKLTDMQTA TMIRQTALPA PERRLEIDSC VKGFGVSVVN

EMMRVTGRVL PPPLLQLIHP DCRTRPDQID WFCKELIKTG REIGVQLVMV VLPYAIF--V

ADIVIDGALL -----AAPEK RVHVNLVNEA KKRLISRSL- -----PVIIL GADVNHPAAG

ESRRPSIAAI VGSMDSNAQR YSAKICVQEV ITNLKDKVRE LLVEFYKPVR IIFYRDGVGE

GQFYEVLSNE LVAIRAACIT FLVVQKRHHT RLFCEDNKDA IGRSKNIPPG TVVDTMIIHP

NDFFLCSHQG IQGTSRPAHY HVLWDDNDFH ADDLHQLTYE LCHTYVRCPR SVSIPAPAYY

AHLAAFRARK HL

>Strigamia SMAR007228

---------- ---------- ----PVFDGR KNLYTRDTLL FRVAVKVSLY ALEEALEGAI

QALDVVPVGR SFFSSPLGGG REVWFGFHQS VRPSQWKMMD FSATAFYKAQ PVIEFMCEVL

LTDSQRVKFT KEIKGLKIEI TRKYRVCNVT RRPAQMQSFT VAKYFLDKYR MKLRFPHLPC

LQVGQEHKYL PLEVCNIVAG QKKLTDMQTS TMIKATARSA PDREKEIDPY VQEFGLSISN

TMMEVRGRVL PPPKLQYFAP QRTCQEQALR NFTSQLQKIS NDAGMQLVVV VLPYAEVKRV

GDTVLGTQCV QAKNVTSPQT LSNLCLKINV KLGGVNSILV PSIRPPVIFL GADVTHPPAG

DNKKPSIAAV VGSMDAHPSR YAATVRVQEI IQELSAMVRE LLIQFYKPNR IIFYRDGVSE

GQFQAVLQHE LLAVREACIT FIVVQKRHHT RLFCSDKKEQ IGKSGNIPAG TTVDIGITHP

FDFYLCSHAG IQGTSRPSHY HVLWDDNHFS ADELQCLTYQ LCHTYVRCTR SVSIPAPAYY

AHLVAFRARY HL

>Strigamia SMAR015088

GKMGKPVDLV VNYFKAHNQY QVDFSLFDGG AILFTTIKLH -EVKIKKQLS SRDPI---VL

QMLNIQKLIR GEYYDLQGHK IELIPGFVTS IRQHETSLLD LAFKSIRQDT -VLAIIRN--

---------- ---------- -RTYRVAEIA IELIPGFVTA FKSIRQDTRN CRISDAGQPL

LMIKPSAKYL IPEFC-LMTG YMKSDFRTMK AISDHMRTTP TDRINRMKEE MKERGFQIRN

NLVTLKGRQF PPEGIVLTRR D----ERIVS DFLTTFKTVC RPMGMQLVFV ILPYDCVKKI

CCVDIGSQVA LGTTLSIMSI ATKIAIQMNC KIGGAAWTIF NPFQ-KIMVV GIDTYKDSAQ

--RAKAVCAT VCTMNNMFTK YFSQVSFQEM SDAFVVQLSN ALRTFTLPTV LMFYRDGVGD

GQLPLVREWE IKQIREAIIL FVIVNKRINT RFF------- -KNSTNPTAG TVVDTVVTRP

YDFFLISQSV TQGTVAPTMY NVILDTTGLA PDRIQLLTYK MTHLYFNWQG TIRVPAPVQY

AHKFAFLVGQ SL

>Metaseiulus gi|391334350|ref|XP_003741568.1|

GSNGRPIQLV SNLYPTVYRY EVTIPAYDGR SILYMMRPLP FEICLKLSLR TFN-----VV

QALDIIPVGR SFFSRTMRGC RELWFGYFCS IRPGQWKPMN ISATLFHEKL PLVDYVVKFL

LKASEHEVLK RELQGVKIKV TRRYTVAGIS KKGAADYTFS VADYFENHYG IHLRYPALQC

VETLTKDGYI PMEVCETIEG QKIVNSDQTA VLIRKTAMTP KKRFESIAAH LREFGLQVDL

KPVQVKARVL NSPTLEICHG SGRC-----A EFAKSLERLG RNLGMVMTLV IMGYEVIKQL

SENDESTQCI RGSNICNNNF ITNVLMKMNA KLGGINNGL- -KEVPPFIVF GADVSHPGPT

NDIQPSIAAV VGSLDSTPSQ YHTVTSFQEY IANLKVMIKD CLRAFYKPMT LLFYRDGVSE

GQFEAVRVFE ILQIRMACLT FIVVQKRHHV RFKPLNDAD- -SRHENIPAG TVVEDTVTHP

FDYFLCSHAG IQGTSKPAHY RVLHDDANRS ADELQTISYS LCHVYGRCSR SVSIPAPVYY

AHLAAARAKD HF

>Metaseiulus gi|391346806|ref|XP_003747659.1|

GTEGRPITLR ANHFGYLHHY DINIPVFDGR KNMYTRDDLP FRVAIKVSLY GLEEALEGAI

QAIDVVPVGR SFFSSPLGGG REVWFGFHQS VRPSQWKMMD VSATAFYKAQ PVIDFMCEIL

LTDSQRVKFT KEIKGLKIEI TRKYRVCNVT RRPAQLQSFT VAKYFLDKYK MKLRYPHFPC

LQVGQEHKYL PLEVCNVVAG QKKLTDLQTS TMIKATARSA PDREREIDPY VQEFGLSIAH

TMMEVRGRVL PPPKLQYFAP QRGCREDCLR SFTQQLQKIS SDAGMQLVVV VLPYAEVKRV

GDTVLGTQCV QAKNVTSPQT LSNLCLKINV KLGGINSILL PSIRPPVIFF GCDVTHPPAG

DTKKPSIAAV VGSMDAHPSR YAATVRVQEI VEDLSSMVRE LLIQFYKPNR IIFYRDGVSE

GQFHQVLQHE LIAIRTACIT LIVVQKRHHT RLFCADRKEQ MGKSGNIPAG TTVDVGITHP

FDFYLCSHAG IQGTSRPSHY HVLWDDNQFT ADELQCLTYQ LCHTYVRCTR SVSIPAPAYY

AHLVAFRARY HL

>Tetranychus tetur20g02910.1

GTEGRPLLLR ANHYGFLHHY DVTIPVFDGR KNMYTSDDLP FRVAIKVNLD HLERALEGAI

QALDVVPVGR SFFSTPLGGG REVWFGFHQS VRPSHWRMTD VSATAFYKAQ PVIDFACEVL

FTDSQRVKFT KEIKGLKIEI TRKYRVCNVT RRPSQLQSFT VAKYFFEKYK MKLRFPHLPC

LQVGQEHKYL PLEVCNIVAG QKKLTDMQTS TMIKATARSA PDREREIDPY IKEFGLSISN

TMMEVKGRVL PPPKLQYFAP QRTCREDALR TFIGQLQKIS NDAGMQLIVV VLPYAEVKRV

GDTVLGTQCV QAKNVTSPQT LSNLCLKINV KLGGINSILV PSVRPPVIFL GADVTHPPAG

DNKKPSIAAV VGSMDAHPSR YSATVRVQEI IQDLASMVKE LMIQFYKPVR IIFYRDGVSE

GQFPAVLNTE LLALRKACIT FIAVQKRHHT RLFCADKREQ VGRSGNIPAG TTVDVGITHP

FDFYLCSHAG IQGTSRPSHY HVLWDDNHFS ADELQCLTYQ LCHTYVRCTR SVSIPAPAYY

AHLVAFRARY HL

>Tetranychus tetur02g10560.1

DKSGRKIKLM TNHFVSIYHY DFSWVGYDGE ANVYLPYKLQ FSVTFKISLA LVKEYYSGAI

QALHIIALGR LAMFPILSNW MELALGHRKS LRFSEIGLTD RASAAFLKSG NGLDFVKSII

PNPAQIEALR RAFANVKIST DKKYIVKDIA EIAANKDTFT VAGFFQQKYG IILQYPNLPC

LIAINGSR-I PIEICKIVQN QRQLDAQEKA KMISRTATEP DERFTCLDED LKDFKLDINV

KPIGVEGIVL NAPRLAYFCA S----QKEVE SFCTAFTKKA SEMKMAFALC FLPYNLTKRI

ADTVLNTQGV TLHSVHDVQT IANILAKVNK KLGGVNVILK ETIKPKIMII GADVTHPGTG

DELQSSVAAS VSTYDSNHAM FYPSVRVQEV IKDFKSMVQE HLENFFFPDT ILYLRDGVSD

GQYRQILNEE VIHLKNPFVT ACVISKRILT RTRPIDPEDA VMK--NVPPG TTIDRIITHP

FDYFQYGHKG IKGTSRPCHY YMLHDDNNLT IDEMSKISYY LCHIFERSTS STSAPAPVMH

AHNLAFKVRQ WI

>Tetranychus tetur02g10570.1

EKFGRKIKLM TNHFVQIYHY DFSWVGYDGE ANVYLPYKLQ FSVTFKISLA LVKEYYYGAI

QALHIIALGR LAMFPILSNW MELALGHRKS LRFSEIGLTD RASAAFLKSG NGLDFVKSII

PNPAHIRALK SAFANAKIST GKKYVVKDIA EKAANRDTFT VADYFRKKYK IVLKYPNLPC

LITRNNSR-I PIEVCKIEPN QRPLSAQEKA EMIKLTATEP DERFTCLNED LGDFKLGIHV

SPIGVEGIVL NAPRLSYFCA S----QQEVE SFCAAFTKKA SEMKMAFALC IFPYNLTKRI

ADTKLNTQGV TLESVCSAQT ITNTLAKVNK KLGGINVILK ETIKPKIMII GADVTHPGTG

DELQSSVAAS VSTYDANHTM FYPSVRVQEV IKDFKSMVQE HLKNFSFPDI ILYLRDGVSD

GEYRRILNEE VIHLKNPFVT ACVISKRILT RTRPIDPEDA VMK--NVPPG TTIDRIITHP

FDYFQYGHKG IKGTSRPCHY YMLHDDNNLT IEEMSKITYY LCHIFERSTS STSAPAPVMH

AHNLAFKVRQ WI

>Tetranychus tetur02g10580.1

EKFGRKIKLM TNHFVQIYHY DFSWVGYDGK ANVYLPYELE FIVNFNISLA SIEEYYAGAI

QALHIIPLGR LAMFPILSNW MELALGHRKS LRFSEIGLTD RASAAFLKSG NGLDFVKSII

PSPVHMEALR RAFAHVKIST GKKYVVKDIA ERAANRDTFT VADYFREKYK IVLEYPNLPC

LITSNNSK-I PIEVCKIEPN QRSLSAQEKA EMIKLTATEP DERFTCLDED LKDFMLDINV

SPIGVEGIVL NAPRLAYFCA S----QQEGI PFCKAFTEKA YEMGMAFALC IFPYNLTKRI

ADTKLNTQGV TLESVCSAQT IANILAKVNK KLGGVNVILK ESIKPKIMII GADVTHPGTG

DELQSSVAAS VSTYDANHTM FYPSVRVQEV IKDFKSMVQE HLKNFFFPDT ILYLRDGVSD

GQYRQILNEE VIHLKNPFVT AVVISKRILT RTRPIDPEDA VMK--NVPPG TTIDRIITHP

FDYFQYGHKG IKGTSRPCHY YMLHDDNNLT IDEMSKISYY LCHIFERSTS STSAPAPVMH

AHNLAFKVRQ WI

>Tetranychus tetur09g00620.1

ASTNKKIELV TNHYCVVYHY DVKIPVFDGK QNLYTVRPIP YRIKIRVDLE ALSDYVNDAV

RALDIIPLGN CIFPKSIGCG KQVISGHYQS IRPTASGLSD KSSTTFYNAT DLVDFIRNLL

TIENNLLFIE KELQGIKLEV TMKFKVRKLT RTSVKETYFS VYDFFLEKYK IKISYLHLPC

VVVGNAKRYL PLEVCQVVKN QKALSQKERI SFIKSSSQDC DRRFEVITNY LNEFSLDLSE

KPIQVDGLCL DRPKIYYLS- --KLTEDVVK RFISLLISFG NDKGAKLIMF IITYHEIKQS

GDVEFGTQCV VDSNVKGFFI LANIFLKMNA KLGGINNVIG VDTTLATIIM GADVTHPT--

DKMSYSIAAC VASLDENQTQ YAASVRVQEI ILDLPDMVYE LLNTYVLPER LIFYRDGVSD

GQFSKVMEKE IERMPEAFVT FIVVQKRHHT RLKPLNPDDS EGKAKNVPSG TLVTQKIVST

SDFFLCSHSS SLGTSKPSHY YVLVDDNNFD SGVLYSLTFS LCYIYAKCTR VISIPAPVQY

AHLAAFRARQ HL

>Tetranychus tetur09g03140.1

TNFARRIQLM TNHFISIYHY DFSWVGYDGS KNVYTPYKLK FSVAFKIPLS SLDEYYAGTI

QALEVISLGR YAMFPVLSSW MELALGHRKS LRLSEIGLTD RAATAFRKSG SAIDFLNWVL

PGTHQIEAIR RAFLCVKVVT DRNYKIKGVT YNAANVETFS VAEYFQRKYG KTLRYPNLPC

LRAGK---MI PIELCEIVAN QTRLSPDEQA EMIKLTATPP NARFTCLNDT LEQFGLNIDI

KPIGVTGFVL AAPKLIFFCA S----PEDGI NFCKMFVTKA CEMKMAFALC FFPYNLTKRV

ADVKLCTQGV ASKNVCSPQL IANILAKVNK KLGGVNVILK QDDKPKIMII GADVTHPGEA

DELHSSVAAS VSTYDKDHTM FYPSVRVQET IEDFQAMIQE HLQNFKLPST ILYLRDGVSE

GQYSQIVTKE VNHLLRPYVT AVIISKRIQT RTRPVNPNEG VGKHGNVPPG TTIDSIITHP

FDYFQYGHEG IQGTSRPCHY YLVHDDNNLS TEEMSQVSFH LCHLFERCTR STSAPAPVMH

AHNLAYRARQ WI

>Tetranychus tetur04g01190.1

GNEGKRITLR SNFFNTVHQY DIQFYAFDSD RILLTNKPLE YQIRIKVTLS F---NFNGVL

QMLDIIVYGS KIFFPSKPTP FELAFGYHQS TQFCQSGPMD RASAVLYREG PLLDRIFELF

LDSKSMDQL- KEFIGLRVET ESRHSIRSFT RTNSRETSFT VYNYFVRKYK IHLKYPNLPC

VEYGQ---YI PIELCKLLPD QRKLRDDLIK ILTHATSQRP SGRAAGTNAA LAEFGITLNP

NMVEVPGRIL PAPRLRYMS- -RKITDDIIR EFCKNLQRVG SMVGVQLIVC MIPYTEIKSV

GDRTLGTQII LDKHFFDTAY LSNLLLKINS KIGGINLSLA DVSKPLTMVV GVDVNHPSPG

-ENSPSIAAV VGSLNPELSN YHTNIVINEE TVDVRGMIKP ILEAFKYPDN IVVYRDGVSE

GQFAYVLAYE VFPLRDMLLT YMIVQKRHNA RFFPMKKEDS T-RSENILPG CVIDTTVCHY

FDFYLCSQNS FLGTARPGKY TVLWDENNLT ADELQKVTYY LCYLFARSTK SIADPAPARY

AHHAAARGKV HL

>Ixodes ISCW015916

-------MVL ANHFGNVYHY D---PAFDGR KNLYTRRKLP FSVKIHVNLD ALHAVYNNVI

QALDIIPVGR SIFMLPIGGG QEVWFGYYTS VRPAQWKPMD RSATSFYEEQ TPLEFMVKF-

LNDSHINTLD EELKLLKVEA MRKYRVIKIT RVSVVKLEFS VAEYFRKKYP RFAHYPQLPC

IMVGSATRYI PLEACRIPKG QRKLAPDMTK EMIKRTAQPP ALRFAKIQPY LSEFGIKIST

EPTQLKGRVL EAPTIVMMNT PR-LRRDELE NFTRLFQQTG GKLGMQLVIV VLGYADIKQT

AETSLGTQCI LEQNFCKPQL MVNLCQKINA KMGGINNGLL LAQKPPVIII GADVSHPAPG

DRIRPSIAAC VGSLDSIPSK YRASIRVQEM IKDLSGMVIE LLKAFRKPEH IIFYRDGVSE

GQFAEVRDLE LQAIRDACVT FIVVQKRHHT RFMPTNDRDG VGKARNVPPG TTVDTVVTHP

FDFFLCSHYG IQGTSKPAHY YVVHDDYNFS SDDLQKLSYY LCHTYARCAR SVSIPAPVYY

AHLAAFRAKE HI

>Ixodes ISCW021130

---------- ----GQLYHY DVEIPAFDGR KSMYFRTKLP YSVAIHLSMS AINKVYEGVL

QAIHCIPVGR SFFKKPLGGG KEVWFGFTPT VHLCQWKPMN VTATTFYKTG PLINFIGQVL

LDIWKIKKLN GILRNIKVRV TPKPKVLAVT TSPATKIEFT VAAYFKKKYG -PLRYPNLPC

IQCGTKEKYF PVEVCEIPEN FTKLSGQETS QMIKMTAIPP AERFQKISSV ASSFGINIDV

KPLELEGRVL DPPQIVFFSC Q--LTVDLLD RFLRKFQDVA TKLGMKFLLI ILNHDAIKLI

CERDLGTQCC MEKNVPLPAL LVNLCHKVNA KCGGDANTI- -SKRPPVIIL GADVNHPAPG

RSNHPSYAAL VGSLDSCPSK YHASVRIQEI IKDLKGMVKE ALRAYYKPRK IIFYRDGVSE

GQFAEVLNHE LPALRQACIV FILVQKRHST RFMPKYQQDG VGRFNNVPPG TTVDRIVTHP

FDFFLCSHAG IQGTSRPTHY YVLHDDVGFQ ADELQSLTFY LCHTYARCPR SVSIPAPAYY

AHWVAFRANQ HA

>Ixodes ISCW013378

GTEGRPILLR ANHFGYLHHY DVTIPVFDGR KNMYTRDDIP FRVAIKVSLY ALEEVLEGAV

QALDVVPVGR SFFSSPLGGG REVWFGFHQS VRPSQWKMMD VSATAFYKAQ PVTEFMCEVL

LTDSQRVKFT KEIKGLKIEI TRKYRVCNVT RRPAQLQSFT VAKYFLDKYK MKLRYPHLPC

LQVGQEHKYL PLEVCNIVAG QKKLTDMQTS TMIKATARSA PDREREIDPY VQEFGLSISN

TMMEVRGRIL PPPKLQYFAP QRTCREDALR NFTQQLQKIS NDAGMQLVVV VLPYAEVKRV

GDTVLGTQCV QAKNVTSPQT LSNLCLKINV KLGGINSILV PSIRPPVIFL GADVTHPPAG

DNKKPSIAAV VGSMDAHPSR YAATVRVQEI IQDLASMVKE LLIQFYKPNR IIFYRDGVSE

GQFQQVLHHE LLAVREACIT FVVVQKRHHT RLFCSDKKEQ IGKSGNIPAG TTVDLGITHP

FDFYLCSHAG IQGTSRPSHY HVLWDDNQFS ADELQCLTYQ LCHTYVRCTR SVSIPAPAYY

AHLVAFRARY HL

>Ixodes ISCW011768

GKLGRPIHLT ANHFGNVYHY DVEIPAFDGR KNLYTRRELK FIVKIQVNLD ALHAVFDNVL

QAVDIVPVGR SFFKPPLGGG REVWFGYYTS VRPAQWKPMD MSATAFYEPI PVMTFMCRIF

LRDFQSVRLN KELKGLRIKV TRRYKVVRIT KESAKKLYFS VADYFQSKYG -RLSYPNLPC

VQSGSSTHYL PLEVCEIIEG QKKLDENQIS EMIKRTAQPP AKRFNEIEPY LREFGIKIST

DPTQLRGRVL DPPSLVFLS- -RFPQKHDLD NFVKLLLRVG QELGMEIMVI VLAYAEIKQV

AETDLGTQCI MDNNVCNAAL VTNLCQKLNA KMGGTNNSLL AQEKPPVIII GADVTHPAPG

DKLRPSIAAC VGSLDSIPSK FHASIRIQEI IKDLKDMMKD MLKAFYKPER IIFYRDGVSE

GQFLEVRNRE VSAIRLACLT FIVVQKRHHT RFMPSSDREG VGKCRNVPPG TTVDSVVTHP

FDFFLCSHFG IQGTSKPSHY YVVWDDSNFT ADDLQKLSYY LCHTYARCAR SVSIPAPVYY

AHLAAYRAKN HV

>Mesobuthus MMa10623

GQEGRKIRII TNYFGLIYHY DVTIAVFDGK KNLFTRNPMP YHVTLKIDLE PLREIFQGAI

MALDTIPIGR SFFSIPLGGG REIWFGYHQS VRVSQWKPLD ISATTFYKSG PLLKFVEEVL

LTDCQIRTLR KELKSLKITV NKKYTINNVT RDPASYLYFS VADYFRKKYR -ELQYPNLPC

LNVGNS-KYL PLEVCEVLPG QKKLDDKKVA EMIKHTARPP QTRFGDIERL TKDFDISINR

RPLQLYGRVL MPPSLMYLAS SNICREADME NFARMICEAG RKFGMQLIMV VIPYAEVKEI

AEIRMGTQCI KDVNLCNAQL ICNLCQKINA KLGGINNSLS PTIKPPVIII GADCNHPSAH

EKIRSSVAAL VGSLDNYPSR YAATVNVQEI IENMKEMMKD MLLAFYRPER IIFYRDGLSE

GEFAKAFNKE LKLIRQACIT FIIVGKRHHT RFIPENPENG IGKHKNIPPG TVVDTEIIHP

FDFYICSHTG IQGTSRPAHY TVLWDDNNFT ADELQTLSYY LCHTYVRCTR SISIPCPVMY

AHLAAYRAKQ HL

>Mesobuthus MMa15079

GGDKRKIELI ANYFGLIFHY DIDIPAFDGE KNLYSNKPLP YEINLKISFE PLNQLIEGGV

MALETIPIGR SFYTKPISGG LEIWFGHYQC VQVTKRKLMD FSTTAFYRNC NVIDFMLDIL

ITPAQQNILL KQLKNVNVQV TRKHRIIGIT PDSANETFFS IAEYFRSQYE -ELKYPNLPC

LDVSTRDRYL PMEVCEIVEG QKKLSEKQTA EMIQLTAQPP VERFKEIDIF SREFGIRIIP

HPVRLVGKVL NPPVITYFSP SQKCNRSTLE NFAKMLISEG RNVGLQLIII VLPYCEVKTV

AETDLGTQCV KDANTCSPQL ICNICQKINA KMGGVNNTLL LEENLDVMII GADCCHPSPG

EDNEYSIAAL VGNLDHFSGR YKASVRAQGI IIEMKDMVKE LLIVYRKPEK VIFYRDGVSE

GEFDKVLNYE LRQIRDAFVT FIVVRKRHHT RFNVLNRSDG VGKYVNIPPG TVIDSDIIYP

GDFFLCSQMA VQGTSQPAHY TVINDDNKFS LDEIQSLTYN LCHTSVRSTT SITVPSPVMY

ASLAASRVKN YV

>Mesobuthus MMa13679

TNDERKIKLI TNYYGIIYHY DIDIPAFDGE KNLYTSKPLP YEVNLKISLE SLYQLLEGAV

MAIETIPIGR SFYTKPISGG LEIWFGHYQC VQITKGRLMD SSAKSFYKNC SVIDFMLDIL

ITPAQLDTLI KKLKNVNIQV TRKYRISSIT PNSANESFFS ISDYFRSQYE -ELKYPNLPC

LDVSTRHNYL PMEVCEIVEG QKKLSEKQTA EMIRFTAQSP VDRFEEIDIF SREFGIRINP

HPVKLIGKVL NPPDIRYFSP SQKCNRSTLE NFARMLIRQG RNVGLQLIVI VLPYCEVKTV

AETDLGTQCI KDINICSPQL ICNICQKINA KMGGVNNTLL LEEDLDIMII GADCCHPSPG

EDNEYSMAAL VGNLDNFSGR YKASMRAQEI IIEIKEMMKE LLIVYRIPEK VIFYRDGVSE

GDFDKLLDYE LQQIRDAFIT FIVVRKRHHT RFNVMNRSDG VGKNANVPPG TVVDTHIVYP

GDFFLCSQMG SQGTSQPAHY TVINDDNNFR LEEIQSLTYN LCHTSARSTT SISVPSSVMY

ADLAAARAMD YL

>Mesobuthus MMa36176

GKRGRKINLI ANYFGEVFHY DVEIPAYDGE KNLYSRKPLP YEIRIKISLE PLHQLMEGGI

MAIETIPVGR SFFRKPLGGG REIWFGHHQN MQICNWKPMD RSATTFYKSC PLIDFMMEIL

LTDANRRVLK KELKNLKVQV TRKYRIFDIT KENASNTFFS VSDYFGAEYR -RLMYPNLPC

IDVGSDKRYL PMEVCEIVQG QKKLTDKQTS EMIRFTARPP AERFREIAPF SREFGIRISP

NPLNFVGRML DPPNIMYFSS SRFCNQNSLE DFAVMLRNGG RNVGVQLVVI VLPYSEIKNI

AETELGTQCI KDVNTCNPQL ICNLCQKINA KMGGINNSLV PADKPPVIII GADCTHPAPG

DKIGFSIAAA VGSLDGYPSR FKASVRVQDM VIDLKDIVKD LLMAFYKPEK IIFYRDGVSE

GEYKKVLDFE LKAVRKACIT FIVCGKRHHT RFTPADRREG VGKHGNIPPG TTVDTDVVHP

FDFYLCSHTG IQGTSRPAHY TVLWDDSSFS ADELQTLTYY LCHTYVRCTR SISLPCPVMY

ADLAAFRAKQ YL

>Mesobuthus MMa10549

GSAGTKINLI ANYFGEVYHY DVEIPAFDGE KNLFTRNKLP YEVKIQISLE PLHQLLEGGV

MAIETIPVGR SFFHKPLSGG REIWFGHHQS MRIANWKPMD MSATTFYKSG PVIDYMLDVL

LTDFQRRSLA KELKGLRIQV TRKYRISDLT RENANNTFFS VSEYFKAEYR -RLAYPNLPC

LDVGTQNKYL PMEVCDIVEG QKKLSEKQTA EMIKYTARPP AERFREIHPF AKEFGIRINS

KPLQFGGRVL DPPNVLYFST ARWCNEEALE NFAKMLYNGG RNVGMQLAVI VLPYTEIKNV

AETELGTQCV KDSNTCNPQL ICNLCQKINA KMGGVNNSLV PTEKPPVIII GADCTHPAPG

DKVKPSIAAV VGSLDGFPSR YKASVRVQEI IIDLKDMVKE LLRAFYKPEK IIFYRDGVSE

GEFNKVVNDE LKQVRQACIT FIVVGKRHHT RFAPVDRREG VGKHANIPPG TTVDTDVVHP

FDFFMCSHTG IQGTSRPAHY TVLWDDNNFS ADDLQTLTYY LCHTYVRCTR SISIPCPVMY

AHLAAYRAKQ YL

>Mesobuthus MMa13534

GQEGRKINII SNYFGVIYHY DVTIAVFDGK KNMFTRYPMP YHIILKIYLE PLQEIFQDAV

VALDTIPIGR SFFSTPLGGG REIWFGYHQS ICISQWKPLD ISATAFYKSG SVLRFVEEVL

LKDYEIRKLR KELKYLKITV NKKYTINSIT RDSANHVHFS VADYFHKQYR -ELKYPNLPC

LKVGNS--YL PLEVCDICPA QTKLDDKQIA EMIKHTARPP QTRFRDIEHL TREFGISISR

HPLQLNGRVL MPPTIMYLAS SNICRESDME NFARMIYETG RKFGMQLIMI VIPYAEIKEV

AEIRMGTQCV RDINLCNAQL ICNLCQKINA KLGGVNNTLS PTIKPPVIII GADCNHPSPN

EKIKSSVAAL VGSLDNHPSR YAATINVQEI IENMKDMMKD MLLAFYRPEK IIFYRDGLSE

GEFIKAFNKE LKLIRQACIT FIIVGKRHHT RFIPENLENG VGKHRNIPPG TVVDTEITHP

FDFFICSHTG IQGTSRPAHY TVLWDDNKFT ADELQTLSYY LCHTYVRCTR SISIPCPIMY

AHLAAYRAKQ YL

>Parasteatoda aug3.g8695.t1

GTEGRPIMLR ANHFGFLHHY DVTIPVFDGR KNMYTRDDIP FRVAIKVSLY ALEEALEGVI

QALDVVPVGR SFFSSPLGGG REVWFGFHQS VRPSQWKMMD VSATAFYKAQ PVTDFLCEVL

LTDSQRVKFT KEIKGLKIEI TRKYRVCNVT RRPAQLQSFT VAKYFQDKYK MKLRYPHFPC

LQVGQEHKYL PLEVCNIVAG QKKLTDMQTS TMIKATARSA PDREREIDPY VHEFGLSISN

TMMEVRGRIL PPPKLQYFAP QRTCREDALR NFTQQLQKIS NDAGMQLVVV VLPYAEVKRV

GDTVLGTQCV QSKNVTSPQT LSNLCLKINV KLGGINSILV PNIRPPVIFL GADVTHPPAG

DNKKPSIAAV VGSMDGHPSR YAATVRVQEI IQDLASMVKE LLIQFYKPNR IIFYRDGVSE

GQFHQVLTHE LLAVREACIT FIVVQKRHHT RLFCSDKKEQ IGKSGNIPAG TTVDVGITHP

FDFYLCSHAG IQGTSRPSHY HVLWDDNQFT ADELQCLTYQ LCHTYVRCTR SVSIPAPAYY

AHLVAFRARY HL

>Parasteatoda aug3.g11588.t1

GTVGRPIQLI TNYFGVVYHY DIEIPVFDGV KNVFTSKLLP YEVVVQIFLE ALQNSSESVI

MALNSIQVGR SFFYLNLGEG LEIWFGCHQS VHSTEKGAAN LAAKAFHKEG PVLGYINDIL

MKPYEMKNVN DALKGIRVEV TRRFIVEGIS KKPASDMNLT VAKYFEMRYG -RLRYPFLPC

LFMRTSNKYI PLEHCKVMKG QGKLSPALGA KMIKQTAINP DRRFQSIGEK MRNYNLLLDL

RNIRVNGRVL NFPTLAYFA- --ECNGKMID TFEQYFKAAG NKVGLAFAII ILSYDEIKFI

ADYQLRNQCI DSNVLINDQI ATNLCLKLNA KLGGVNHILR NKLR-PVMIL GADAVHSPRG

-ARCPSIAAV VGSMDAFPSK YKIACRVQEL ILEMRAMVAN LLKAFYHPDK IIFFRDGVSE

GQFQKTYEYE VSEIQKACIT FIVVQKRHQT RFIPANTRDG VGRHGNIPPG TTVDRDIVHP

FDYFLNSHEG IQGTSKPAHY TVLHDDNKFS PDELQELSYH LCYTYNKCNR SISIPAPVKY

ADLACYRAKK FA

>Parasteatoda aug3.g12795.t1

GILGRRIRLV SNLYGKIFHY DVKIAVYDGK YNLYAPSPLQ FLVLMNINLS VLHELYQGAL

VALETVPIGR NFYHPDLSGG IQIWFGYHQS TRLGQWNLTN TSATTFFNKQ NLIEYVGKSY

LSESAIERLA GELKNVKIET GARRCIIDVT MQSARSLKFS VLQYFRDTHN INLAYPHLPC

VQVKPKERYL PLELCIIPEG EKELSIEDKR KMIQYTADPP VKKFNKIDKY LNNFGVKVED

KAISVNGRVL EAPTLNYLAP EKWCGYRDLE RFSQELKIIG KNAGLQLTVV LIPYNNVKQV

AEIELGTQCI DSNNACNASV LSNLCIKINA KMGGINHILT QREIPPVIIM GADVCHAGIT

DKTGVSVASL TASLDMLLSR FAVICRLQEN ILELKDMVKT LLKAFYKPEK IIFFRDGVSD

TQFQDIKKKE VNAIREACIT FITVQKRHHV RFRPEDFNDG VGREGNVPPG TVVDTGIVHP

SDFFLCSHQG LKGTSKPAHY TVLEDDNKFT ADDLQKLTYC SCNISFRCSK TLSIPVPVHY

ADLAAYRTRS RL

>Parasteatoda aug3.g12797.t1

GTLGRPIRLV CNLFNDVYHY DVKIAVYDGE SNMYTPSPLA FKIQIKISLS VLHELFRNAV

MALETIPIGR NFFHKDLSGG IEIWFGYHQS LRLGQWKIMN TSATTFFSKQ PLIEFMAKSL

IGNHELKKLR SEVKNLKIET YVTHCIIDVS HKNARDITFS IDQYFLERYN KKLDFPFLPC

VQVKPKNKYL PIEVCYIPEG QKELSDNDKR EMIKFTADNP ENRFRKIDEY LKNFGIGVER

DPVRLSGRTM QAPNLNYLA- ERHCRFNDLK NFSDQLQRIG GAAGLQLVVV VIPYRNVKQI

AEVELGTQCI DHNNACNPSL LGNLCLKINA KTGGINHVLT QGEIPPVMII GADVSHAGVT

DKSGISVAAV AGSLDMILSR FAVTCKLQET IINLKDMVKS LLITFHWPEK IIFYRDGVSD

GHFSEVLEKE LTAIRGACIT FVVVQKRHHV RFRPEDHRDG ARPEGNVPPG TVVDTEIVHP

RDFYLCSHLG LKGTSRPAHY TVLADDSDFS AEDFQKLTYY SCNISFRCSK SISIPVPVAY

ADLAAYRTRL RL

>Parasteatoda aug3.g12799.t1

RPPGRVIRLI SNCFGSVYHY DVDIAAYDGR KNLYTKRPLN FVVNIQISLD SLQALFEGAV

MAVETIPVGR SFFYPPLGGG REIWFGYHQS LRLGQWKPMD ITATTFYQKG PVLNYIAEFL

LRDADIRRIS KELKNMRIEV NRKYRILTLT RERADRLEFT VANYFRRQYN RRLRFPHLPC

LQVNPAAKYL PIEVCDMVEG QKKLEERQNA EMIKFTARPP KARFDEIDQY LQEFGMRVYN

EPLRLEGRVL GPPSVRYFGG SGFCSHEHLS KFAKLLVNIA GESGIQLAVI AVPYGEIKQA

AETVLGTQCV KDDNVCNPPL VSNLCQKINA KMGGINNSLT PGETPPVLII GADVTHPSPS

KDIKPSIAAA VGSLDSHPSR YAVTVRAQEI ILELKSMVKD LLSAFYKPEK IIFYRDGVSE

GQFEHVMRHE VTAIREACIT FVVVQKRHHT RFMPQDARDG VGRMKNIPPG TTVDNTVVHP

FDFFLCSHFG LQGTSRPCHY TVLADDNNFS ADDLHKLTYY LCHTYVRCTK SISSPAPVMY

AHLAAFRTRQ YL

Alignment supporting figure 8, Dicers

>Drosophila Dros_Dcr1

RDFQVELLAT AYERNTIICL GHRSSKEFIA LKLLQE---- HGRVSVYLSC EVEPCSIYTM

LTHLTDLRVW QEQP---MQI PFDHCWTDYH VSILRPEGFL YLLETREELI VLEDCHYQRI

RPRILGLAGP LHSLATLEQS VLCQIETSRP HEYIVLLVVL HEMGPWCPKV RRLLQTLRCF

LCALIYCNQN TARVLFELLF LRCQYTTDQE EVLKRFRMHD CNVLIGTSVL EEGIDVPKCN

LVVRWDPPTT YRSYVQCKGR ARAAYHVILV EQMLLSKCSA CLAAYDLGSA IALVNKYCAR

LPSDTFTKLT AYTLRLPINS PLIVGLPMAR RLAALQACVE LHRIGELDDP GTTKRRQYYY

KRIASEF-CD CRPPCYLYFI QLTLQCPIPE EQNTRGRKIY PPEDAQQGFG ILTTKRIPFS

IFTRSGEVKV LTSEQIVCIN GFLNYTFTNV LRLQPFDPQR FQDAVVMPWY FPGDNYRTFK

HYYLVKYQPL LDVLNFLTPR YVNRKGVALP TSCTVHPFPA SLWRTAVCLP CILYRINGLL

LADDIRDRQP -DLVGHPGIL QALTANDGIN LERLETIGDS FLKYAITTYL EGKLSHLRSK

QVANLNLYRL GRRKRLGEYM IATKFEPHDN WLPPCYYVPK ELEKALIPDK SIADCVEALI

GAYLIECGPR GALLFMAWLG Y-GAWPTPRS PLEESLGYKF RDRSYLLQAM THASYTPNRL

TDCYQRLEFL GDAVLDYLIT RHLYPGALTD LRSALVNNTI FASLAVRHGF HKFFRHLSPG

LNDVIDRFVR IQQECDDAEV EVPKALGDVF ESIAGAIFLD SNMSLDVVWH VYSNMMSPEP

KSPIRELLEL EPETAKFFRG IGRNYRIAKC TAAKCAL

>Drosophila Dros_Dcr2

RGYQLRLVDH LTKSNGIVYL PTGSGKTFVA ILVLKRPIES GGKRALFMCN TVLARQQAMA

VRRCTNFKVG FYVGDDWTRG MWSDEIKKNQ VLVGTAQVFL DMVTQTYSVV IIDECHFREF

MPRVVGLTGV LIKLKELEIT YRGNIITTKP TEVMVFLYQM KEYGIYAPKV QRFLMSLKVS

ICCLVFVERR TCKCIYGLLV LTPQFMVGQK SAIQQFRDGN ANLMICSSVL EEGIDVQACN

HVFILDPVKT FNMYVQSKGR ARTAKFVLFT HNDIAEYLQD DIDPFLPNNA LAILHRYCQT

IPTDAFGFVI PISINMPVNC MLIYSDPMAK ISAAFKACKV LYSLGELNEN KADKSKDRTY

KTECPLEFYD ALPICYAYEI FLEPQFESCE YTEH----MY LNLQTPRNYA ILLRNKLPMP

LFSNQGKLHV QNSEQLELLH QFHGMVFRDI LKIPAPRPED FEGKIVTQWY EKNQQDKTYY

EFTMSKYKFM IEVLTF---- YVHNRGKFNA KSCFNFNFPG DLWLKLIFLP SILNRMYFLL

HAEALRVKPL LILQKTVSFL AAITAADVFD MERLEILGDS FLKLSATLYL EGTLTEVKSK

LVSNRNLLFC LIDADIPKTL NTIQFTPRYT WLPPGISLPH ---NVLIPNK VIADTLEALL

GVIVKNYGLQ HAFKMLEYFK ----IDKPLT QLEKNLGYTF KDRRYLLQAL THPSYPTNRI

TGSYQELEFI GDAILDFLIS AYIFPGALTD LRSALVNNTT LACICVRHRL HFFILAENAK

LSEIISKFVN FQEELDMTEV DVPKALGDVL EALIAAVYLD C-RDLQRTWE VIFNLFEPEP

INHIRQLVEH KHAKPVFY-G FGSNKDQAKL SAAKHAL

>Daphnia DappuP309030

RDYQIELFQA ALDENIIVYL PTGSGKTFIA ALLIKEPLDS GGKRTVFLVP TVLAIQQAAY

LRRHTYLKVK EFYGDIWEKD RWNIEFDSNH VLVMTAQIFV DILNHAFNLL VFDECHMKQV

LPKILGLTAA LFRIQQLSET MGCVVRITKP CETILIDYLL STIGPYGKQV CNGLNFII--

VCTPYFAKYS DRLLLFVQPF LKPLFTMGQN EIMQYFREGL CNLLVATSVL EEGIDIPDCN

LIIRFDRIKT YCDYVQTKGR ARSAFYCILV EQQLLSTGSQ IIPPYTLPSS ISLINRYCGQ

LSSDPDVSLA PCHLFLPLNS PLVIGDVMAK RAVALKASIK LHELKELDDP QLNKENIAVY

NRRLPVCFSN CRPPCFVYAI DFTLTKPCLD IT-----KLY FPFAVDTKLA ILTSKVIPFP

VVTRAGEFQV LDQSQLGKLE RFHQFVFQDV LFLFIFDSSR LIDSVIVPSY FPNNEFKTYA

SYFQLKYQQL LQVNFLVNRV ASTREETPM- --CHVHPLAG SIWKQVVWLP CILHRLDRML

VAEELRDSGN SGMLSNFGIL EALTTHEGFD MERSETIGDS ILKLVISIYV EGRLSLMRMR

QINNKHLFKL GAKKDIGEFT VAQRFELMAN FLPPGFKTPT ------VLMK NVADCMEALI

GVYLTTTGIK GAIKLMDWMG FNEMNGFPIL PSEKRLRYTF KNKALLIEAL THASYIPNRI

TNCYQRLEFL GDAVLDYLVT RYYYPAILTD LRSAMVNNET FAVMAVQNRF HLYLKHLSLS

LNVILDRFVR SQEHQSLASI DVPKVLGDIF ESVAGAIFVD SGMSLDAVWK SYLPFLHDAP

ISALRVLHER YPNALKFFKG AGGNSKTAKS AAAKYAL

>Daphnia DappuP308316

---------- -------MYY SRWT------ ---------- ---------- ----------

---------- ---------- ---EELGKSQ VLVMTYSIFL EVIHKNFNLL ILDDCHLKGV

LPRILGLTAS IVNITILETK LKSVISTTRP KELICYIQII QNFGPWGPKV LRLLDILRIY

LCGMIFTERR TAKLLYHLLY LSPLYTVDQE DVMKRFRSRD CNILVATSVL EDGIDVPACH

LVIRYDLPQS YRAYVHSKAR ARAAHYILMV EQILLSKSNC IQAPYSLQNA ISIINRYCAK

LPSDCFTRLT PCSLQLPINS PLVLSQPMAK RSAALEACRL LHQKKELDDP GTTKRRQYYY

KQVADPF-IN GIPPCYLYAI TMVLSEPIPE EQNTRGRKIY KPETSIQSLG ILNSKPLSFP

IFTRSGEVEV VTEGQLKQIS DFQRYIFRNV LSLYVFDPRL FSDAVVMKWY FPDNNYPTFE

KYYRHKYQPL LDVLNFLTPR YVNRKGVMLP TSCSIHPFSA SLWRQAVCLP CILYRLNGLL

LAEQLRDEQQ -GLESHPGIL QAFTANDGIN LERLETIGDS FLKYAITTYL EGELSHLRSR

QVSNLHLYQL GKKKLFGGCM VATKFNPHEN WLPPGYVIPD ALEEALIPDK SIADCVEALI

GAYLRACGPR GALLFMSWLG Y-GYWTPPIS PLEEKICYKF NDRSYLLQAF SHASYYLNRL

TDCYQRLEFL GDAVLDYLIT RYLYPGALTD LRSALVNNTT FAVLAERYEF HRYFKHLSPS

LNQIMDKFIK AQE---EAEV EVPKVLGDVF ESVAGAIYLD SHMSLNAVWR VYYNMMKKEP

KSPIRELLEL ETDRVKFFKG IGRNYRIAKC TAAKYAL

>Daphnia DappuP329028

RNYQLELYEH ARRENTIVYL PTGSGKTMIA VLLIRDPLAQ DGQRVVFLVP TVLAKQQAAY

IRRHTCLEVG EYYGDLWKGD RWAKELEKNN VLVMTAQLFV NAVNHGFALL VIDECHMKHS

LPRVLGLTAA LFLISKLKTS MNSIIRTTKP IEIICIKILT SIFGPYGPHV TRLMDILRQF

LCSIVFVERR SANVLYHILY LNPLFTMGQS KIIRQFRDGT CNLLVATSVL EEGVDVRACN

LVIRFDGIKT FCDYVQSKGR ARSAFYILMV EQSLMDPSQK IIPPYTLLSS ISLLNWYCAT

LPSDGSAPLA PCTLLLPLNC PLLIGRVMAK QEVSLQACIR LHQLGELDDF EHSQGSDFYL

RGVARLL--- ---PFYLYLL TYEVVKDSAQ PENQ-----F DPNRAQRRMG FLSSQKLPFG

LYSPAGQINV LTLDDVKLCH RFQQFLFEKI LQLVVDYFKV YGDAVVTAHH FPNPAYATYA

NYFRDRHQPL LEVFNFLTPK YVNRKEAADE GDCEVHRFPA SMWRQAVWIP SIFYRLNSLL

LADELRDDGP MD-NCPKGVL RALTANAGYD LERLEVIGDS FLKLAASIRV EGKMTHLRML

QVCNRNLFKL GKIKSIPRFV VATKFVAKEN WLPPCYFPNN TDDDCMISKK SVADSVEALF

GLYLTLHGIK GALKVMRWMG SSSFGGTPPE PLERSLNFRF GNRFYLLQAF SHASYHHNRM

TSCYQRLEFL GDAVFDYLIT KYLYPGALSD MRSALVNNVT FAVLAVRNGF HRYLKHLVPD

VHQAIDRFVQ QQEDDPMAEV EVPKVLGDIF ESLVGAVFLD SDMSLDAVWR VFYPLIRQEP

KSPIRTIYEK YPGKVQFYKG VGKNSRIAKN TAAKYAL

>Strigamia SMAR009083

REYQVELLEK AIKKNIVVCL GTGSGKTFIA VMLIREPFKN GGKRTFFLAK TVLVAQQATV

IRQHTGLQVG HYTGDAWDLD VWKREFEKNE VLVMTTQILV NILNMAFNLL IFDECHIVQV

MPKVMGLTAS VLNIKSLEIR MHCVIETTKP KELFILMFAV QELGLWCHKV RRLFETLLKY

FKAIIFVERR TAWVINRFIF ITSEFVMGQA EKILKFRLKE CNVLVATRVV EEGMDIPTCN

LVIRFDEPKD FRAYVQSKGR ARASNYIMLV EDALQEICDM DIPPYTMFSC IQLVNRYCAL

LPKDKFTRLS PCILWLPVNS SVIKGDIMAK KSVAMTACIL LHKCGELTNP GSLKRRQLYR

KQVAPQFKVC PRPPCYMYLF RIKLSEWT-- -KDKIGGTMY DSEKCCHNFG IITSCALPFP

IFTRHGKHWV FENLELEQLA RFHRNFFIQF LRIFQFEEGK FADSVVTPLY FPNEQFKTFT

QYYKKS-QPL IAVLNML-PI KVEK------ TACSVLPIRA SHFNQGTMIP SILYRLNDLF

LAHEFLYKIE NGIQFSTAIL NALTANDCFD LERLEFLGDS FLKYVTTLYL EGRLTSGRGT

LVSNSHLYRL AKSVGLSELV KTEIFSPASH WLPPGFTMKR ESEYRLIADK SVADGVEAIL

GAYLLYSGPK AALRFLVYLG --RPYNFPTT ALEEGIGYVF RDKSFLVQAF THPSYYNNQV

TDCYQRLEFL GDAILDYLIT WHLFPGKLTD MRSALVNNVT FAKLAIQSHF NYHLNARSPS

LFNAITKFLA LAEECSDEEV EVPKALGDIF ESVAGAIYLD SGCSLDAVWK VYFKMMEQIP

SSRSLRVVSR HTVQAKPFRG VGPNARQAKI NVAKVIL

>Strigamia SMAR007746

REYQVELLDA ARKHNLIMCL GTIPNKVFVS LMLVRERKRE KLKWTVVVFT CGVVHRYANM

IRDHIDLNVA EFTR---EHL SVENNLTDNN VLVMTAQIFD HFLQTDKNLI ILGDSHYEKI

ALRILGLSAN IMQMNCLERT VNCRVETNQS NEIVVILHIL SSLGAWCPKI LRLLDILIQF

LCGLVFVDKR TACIMHMLLF LVPNFLVGQE EVLRRFRLHE CNLLVTTSVL QEGIDIPKCN

LVVRFDLPRD YKAYVQCKGR ARASHFVLLT EEVLLSRCDE LLPVYSMSTA ISLVNKYSAK

LPSDTFTRLT PAVLFLPINS PIVVGPVMAK MAAALNMCKV LHKAGELDDP GTTKRRQYYY

KSTAKAF-VN CHPANYVYVI NMKLTCPIPE EQNTRGRRIY APEETERCFG IVTSKPIPFP

VFTRSGEVTV LTNDQLLRLS FFHNYTFSKV LRLFEFVLPN YEDSVVMPWY FPDDEFETFE

NYYFKKYQPL LDVLNLLTPR YVNRKGVALP TSCTIHPFPA SLWRKAVCLP CVLYRANSLL

VAEQLRDKQV -SLSSHPGIL QGLTANDGIN LERLETIGDS FLKHAITAYL EGKLSYLRSK

QVSNLNLYRL GKKKGLGECM IATKFEPNDN WLPPGYFVPK ELEQALIPDK SIADCVEALI

GAYLTSCGPR GALLFMSWLG F-GFLKAPPS PLEEKIGYCF RDRSYLLQSF THASYHYNRL

TDCYQRLEFL GDAVLDYLIT RHLYPGALTD LRSALVNNTI FATLAVKHDF HKYFKAICPG

LFVVIDKFVQ MQKECEEAEI EVPKALGDVF ESVAGAIYLD SNMSLDTVWR VYYNMMKSEP

KSPIRELLEM EPETAKFFRG LGRNYRIAKC TAAKHAL

>Metaseiulus gi|391328911|ref|XP_003738926.1|

RHFQLELFEL AVKQNVIICL GTGTGKTLVS ALLIKERLEN GGRRSVFLAP TVLVQQQTAY

LKRHLNASVA SFVGDDWTGT QWWQEFDQHN VLVMTPTILN NILNANFNLL IFDECHYVQI

MPRILGLTAS VLNIEELECK LGANLVTTRP REIIV---ML AAVGPFGEKA WKLMNVLEAF

LCGIVFTVER TVFALCSWIF LRCDFVIGQE RVLQDFRKRQ LNLLIATSVI EEGVDIPACN

LVLRYDPPKN MRSYVQSRGR ARGSMYLLFA EFQLLRYCRS HLSNITPFAA RQILQWYCDS

LGSDRYTLQK PCGIHLPRQS PLVYGAWEAR TSAAINVLSR LYSLGELTEP ATQTNLILCP

RRMSSSLQPT SVPPVDLILY KFSLRIDKLN DPDHLDSRYM DPADEPICMG LLVKIALPFP

VYTRAGTEMV LHESQYKLCA EFNRYLFDEV LRI-VSEIPL SEDSVVNIEA ----------

---------- ---LEMIFPR HEDRLGNVSL RTCKVEKLSA PVWNKATSVP SIIYRLNRLL

VADELR-NVP TDLENCDGIL KALTITDIVD SERLEVIGDS FLKIAVTLHF EGELTTRRCR

IISNMSLLQH AVPKGIHEMV ECVKFRVRVN FVPPGAQERA LLSGRCYKSK MCADSVEALI

GVYLEKCGPV GALEYLRWLD FLDRFELPLP EPQETLGYIF QNELLVLEAI THKSYRYNRL

TRSYERLEFL GDAVIDYLIS KYIYPGQLTD VRASLVSNNT FAAIIVDNKL HTVMQHHSPL

LLKLTNRFVE YREDLEDLEV DIPKPLGDLM ESLMGAVFLD SGKDLDVVWA VLSK-----P

RSSAQ----- ---------- ---------- -------

>Metaseiulus gi|391332351|ref|XP_003740599.1|

RSYQIELFKS AKERNTIVCL GTGTGKTYIS VLLLKHP-GS GGKRSIFLAP TVLVEQQGAV

LGRHLSVKVG IYVGDLWSRE RWVNELKAHG VLVMTPAILY NAACHGFSLI VMDECHYVSI

MPRVLGLTGS VINIRELEAT LFSTASSTKP KEMIVLSSCY EECGAFCPKM HRLLEVLAAF

LCGIVFVKLR IAYVLYQWLF LRCAFIVGQR RTIAAFRDGV YNLLLATNVI EEGMDVPACN

LVVRFDAPRD ARSYTQSKGR ARASLYVVLC ERIVTTFCDE FLEPFSAKSA MSILCWYCQT

IVHDQYSNSL PMSLTMPLLC PVITNEDRAK AYLALEMCRK LHEKRELTET RHKTRQYLNR

RAIAPPLEIL EENECHLYVF KNSVFEKSSS RSTGAKSPAD GP-QGSTYLG ILVRNRDEFP

IFTESSEQLI MSQSKKATFA RFNRYIFSVV LNIFIFDKEL YEDAVVEVLF FPFAPDLNFL

EYFGGKQQPI LELLEMLFPS AESS-----D RGVVVHKMKA NLWRRAVCLP SILYRLNRFH

VCETLR--VY PDLNDCEGFV RILSSVDELD IGRYRLVGES FLQMVIGFSL EKELRSLRSY

KISTRNLLVR AYERNITEIL DNVKFIAKEN FLPPGYRTLE -KEEKLYRAR VAADAVKAIT

GLAVDKCGPS GALRVMRWLG FVDAFALNLD SLESRLRYRF EHKALVLEAI THHSYRGDSL

TQSNVQLCSI GAVVIEYLIS KFIASYNLFE LRASICSAVN HAHIVAKNGL HKNLLYTNQS

LFTVIRNYID CLDESDDTDD RVPLALAEML MALVGAVFID SGKNLEIVWS VYAELCGEQP

ISPIRELFAR FPGSYFEFSQ LANSKAEGRI SIAKKFL

>Metaseiulus gi|391341486|ref|XP_003745061.1|

AEFQVELLAE AKRGGNKVIF ISVLDKPFFI VNFTKEPSAS IDKLLVVCLS ST-MLSTFQW

LDDYTDLTCV C---DADEDE GLEKRLSNSS VILTVPTAIS TLSEFSLCTV LIDRCEIAGS

ALRVVGLTNA LSGIQNLEKY WKAQATVPKP EEKVLLAYVF LTLGPWCDKL TNLMDAILLY

LCGLIIVRHR TALVVNRWLF VKSNFIIGQE EVLQKFRYRD YNLLITTTAT EDTLELPHCN

LVVRFDPPES YKSYIVCKAK AKAARFFIML EGLLLERSSD LERCYTLSNS IAIINRYCLR

LPSDTLTKLA PCVLRLPINS PLIVGEPMAK QDVSLRALER LHKRGEIDDP GTTKRRQYYH

KSLAPPF-QG TLD--FLYNI SMKLLCAIPE EQNSRARTIY DPADSPRGFG FVSGTRLTFP

IYTRSGEVMV LTEEQRKKAE FFHAYVFRDV LRLFTFNREI FSDAVVMPWY FPATPFKTFD

DYYRHKYQPL LDVLNLLTPR YVNRKGVSLP TSCLIHPFPA SLWRKVVCLP CILYRMNHLL

LADSIRDD-- -DDSESCGLL QALTANDGIN LERLETVGDS FLKYAITNYL EGKLSFLRSK

QISNVNLYRL GKELGIGSLM VATKFEPSDN WLAPGFCIPE GLEKAVIPDK SIADCLEALI

GAYLVSCGRC NTLKCMTWFG FASLFKKIPP PLEERIGYKF KNKGFLLQAF THASYHYNTL

TDCYQRLEFL GDAVLDYLIT RYLYPGALTD LRSALVNNTF FAALAVKYEF HKYFLNLSPR

LFKLIHRFVS TKKECEEAEI EVPKALGDIF ESVAGAIYLD SGMSLDTTWN VYLAMMKPEP

KSPIRELLEL EPQTAKFFIG IGRNKRIAKC TAAKRAL

>Metaseiulus gi|391345092|ref|XP_003746827.1|

RDYQIELLLK AVERNIIICL GTGTGKTFVS VLLIKE--DG GNRRSVFLAP TVLVKQQAAV

IKIHVDAEVG CYFGDNWQES EWRREIEKNN VLVMVPQVFS NILNQNFNLM VFDECHYVQI

MPRILGLTAS VLNIRGLCSR LDSTLATTKP REMIVMKTML IEVGPHGPKL RRLLNIFETF

LCCVVFARER TVFALWCVLF IRPGFVMGEG STLENFRTGV HNVLVATSVI EEGVDVPQCN

LIVRMNGDMN FRSYVQGRGR ARASIYAILV EQIITRYCDP DLPTITAQSA RSVLQWYCDT

LGNDRFQVMR PCSILLPKAS KLVYGKWLSR VSAALATCKR LYELDELTET IQQKSSIKCE

RAMSRELDSG KLPGASIYVF RNRLEIPLND DHSHM--RTF RPEEEALCFG LLTFASGCFP

IFTRNGREIV VTRELWELCT KFHAYLLKRV CRL---EKQM FSINAVVSRK ----------

---------- ---LDFCRDV YKVKTKFKEQ TSFEVEFLSA PQWSKARTIL TCTHRLNRLL

IAEHYR---I KEVENSPGIL TALTAQDVVN SERFEVLGDS FLKIAVTLHL EGTLTAERCR

LISNMNLLKL AVPKKIHEAV ESRIFSARES FRVPGMISRE ------YKSK ICADSVEAMI

GVYLERSGPI GALEFLRYLG FLSRFDLSYD PVESILGYRF KNRLYLLEAI THQSYN-NRV

THSYERLEFL GDAVIDYLVS CFIFPGQLTS VRSALVCNNQ FAQIVTKAGL SRCLLHRSPL

LFRMMTSYTE AVEETEDADV DAPKPFGDLM ESLIGAVFID SNQDFSRTWD VFRGLMGDRP

RNPIAVLEEM FPSGCEYFSA VSRKKKIAKL LLAKIAL

>Metaseiulus gi|391348645|ref|XP_003748555.1|

RSYQIELFEF AKKHNTIVCL GTGTGKTFIS VLLVKHP--- IGKRTVFLAP SVLVEQQSAV

LAQHMTVKVA SYVGDRWSSE NWNREFNENG VLVMTPEVCR IAIDHGFNLL VLDECHYVKI

MPRILGLTAS VVNIRDLEAC LDSVAKTTKP TEIIVLASTY HTCGPYCPRM TRLLEVIRRF

LSAVVFVKER VAYVLYNWMF IRCSFIVGQN KVMKNFRNGE YNLMFATSVI EEGMDVPACN

LIIRFDPPMD VRSYTQSKGR ARASLYVVLS EQLVMKCCDP DLPPYTAKSA ISIIYGYCQR

FMRLKYPNLF PCTLSMPIVC PVISNRARAK SYVALEMCKR LHQCGELADK AGEKKRYVLP

RKRASILELK EEGTYHLYVI RTEVVSWTAK EQNWRGDPVF DPVKYPTWLA IVLRSKSNFP

IFTRSGEECV FDDELKSLCA RFNRYVFSIV LDVFEFRDED YDDAVVELLY YQFDESLSFA

EYGFRRTQPV LEVLRMLKPR SSDQQEKKQK YLVAVHKIKA SLWRKLTSIP TIVHRLNRLL

LAEDVRDMAQ PDLENCLGLM KALTCEEIFD LERLELLGDS FLQIVSTFAV ISELNVLRQY

EVNNSHLLYR AHEKGLTQVL EAQPFRASYN FLPFGHRTAP -FEQKLFKAK IGGDVVEAMA

GVYLEKCGPV GALRFFKWIG FLDAFKLDFS AAERQVGYKF KSRGLALQAI THHSCREHLL

TDDMDKLAFV GEAVIDYLLT LYIYPGQLTD LRSALINKQV TAYAVVRAEL HTVLLHTSNK

LFSAIKKYLS NFE------- ---------- -TVIDDRFYC LS-------- ----------

---------- ---------- ---------- -------

>Metaseiulus gi|391348710|ref|XP_003748587.1|

RSYQVELFEY AKKHNTIVCL GTGTGKTFIS VLLIKYP--- VGKRTIFLAP NVLVEQQSAV

LACHMTVKVG TYVGDRWTSE HWNKELQEHG VLVMTPEVCR IAVDHGFNLL VLDECHYVKI

MPRILGLTAS VVNVRDLEAC LFSTARTTKP VEIIVLAKTY VSCGAFCPKM KTLLQIIKQF

LSAVVFVQER VAYVLYIWMF VNCSFIVGQK KVMRDFRGGI YNLMFATSVI EEGMDVPACN

LIVRFDPPMD VRSYTQSKGR ARASLYVLLS EQLVMHCCDP DLPPYTAQSA ISVIHSYCQQ

FMRMKYPNVF PCTLTMPIVC PLVSNATRAK MFVALEMCKR LHACGELSDK SHQKKRYILP

RKQAAILELR EENSYELYVI KTDVIAFASN LQNWRGEDLF DPSKYPTWLA IIMRSGSSFS

IFTRSGEERV LDDKLKKLCT RFNRYAFHVV LETFVFKEED YEDAVVELLY YGFNKSMSFA

DYALRKTQPV LTVLQMLYSR FENQQERRQN YVCAVHSIKA SLWRKICSIP SILYRLNRLL

TAETIRDVSQ PDLRNCLGIM KALTSQEVFD LERLELLGDS FLQIVATFSV ISELNVLRQY

EVNNRHLLCR AHDKKLTSVL EAYPFKATHN FLPLGCRPIP -HEEKLFKAK VGGDVVEALA

GTYLEQCGPI GALRFFRWIG FLDAFKLDFS AAEKQIDFTF KSKGLVLQAI THHSCRDHLL

TEDYNRLAFI GEAVADYLLT LYIYPGQLSD LRSALINNQV TAYAVVRAEL HTVLLYTNSK

LFSAIRKYLE NIDETDDAEA EVPRPLARIL QSILGAVFID SGKSFESVWR ILVRIMGHEP

IPPTTELCKK FPGTVFSYSQ IAENKKIAKT TLAKRYL

>Tetranychus tetur07g00990.1

RPYQLEALDK ALSNNTILCL PTGSGKTFIA TIVLKEPYNE NGKRAFFLVP NQLVQQQAKA

IENDTTFNVG RFSGDDWTKA YWDLIFMKYQ VLVMTRKIFF DIIASGHAVI IFDEAHYVRI

MIRIIGLTAS LINIKEIGNT YCADVFSSNA NEIIWLEHIF STFGPWANKM LRLLQFLYEF

ISAIIFVERR DAYVLSSWLF IKCDFTVGQE TVITRFREKT LNILVSTSVL EEGLDINHCN

CIVRYDKPTN YRAYVQSKGR ARSAYFIFFA ERKLRFLSLQ T----PLYEA KTRVIFYCDH

LPADGYTVKM PCKLTLPKIS PYIFGRKCAE ASAYFEAAKE LWAHQELDDP GTKKKRRQY-

----PIIFSD KLKPCWLYKV SAK------- ---------N EKQEETSRLG LISFYPLPML

IYSSKENLTI LKEQQLENIR HFQEFIFSGC LKI------- --GALISKNY FVNESYATFE

DYYKTRYQPL LCCTKTFFPC QPDRKAMKLA NSCSYEYFSY EYLDIIYTIP AISSVINQYL

NASAIRTPIY TDKLSLFKIV EAFTADSKIN LERLEIIGDS FLKYIVSALL EARITYLKMT

LVSNFYLLHL GRSKGLAQYI NDKTFLPRLN WLPPFYSSAN ------ISDK SVADMMEALL

GAHLICLGEK AAISFLSWIG -----SFNFD SLEQKLGYTF KRKDLLIEAL THPSWSRSHF

-NSYQRLEFL GDAILDYLIT SYIFPGEVTI IRQALVNNDY FATISLVNGF DKFIFHLSPT

LYKEINTFRE QLTEMETLT- EPPKALGDIF ESIAGAIYLD SGNSLSTVWH IYCKFFCNEP

KSPILRLFEA FP----FLLE DAMNKRTEKH YLVLSAL

>Ixodes ISCW000889

KRFEVELFEG AKEANTIVCL GTGTGKTFIA VMLIKEPFEE GGKRTFFLAP TVLVAQQQKA

IQAHTSLRVG GYIGDNWDAA RWHKEFVNSQ VLVMTPEIFK IILHHAFNLL ILDECHYREI

MPRVLGLTAS VINMHGLEMA MRSRVLT--- ---------- -------PKL KKLLEVLRVF

LCGIVFVKER VARVLCAWLF VRPHFVVGQC KVLEQFRRQE CNLLVATSVV EEGMDVPKCS

LVVRFDFPPD YRSYVQSKGR ARASLYLMMV ESLLMNKCDE MLPPYTMTSA IGLVNRYCVK

LPSDIFTRLQ PCTVYLPMTS PLIQGQPMAK MAAALETCKR LHQMGELDDP GTKKRRRVYS

KRVCRLFREA PLATFRLHVL TTRLERLASD LQNWRKERLV DPEDSPLWFG MLLREDMPFP

IFTRSGEELV LTKEQCERLR HFHRFVCDEV MRVFQFQRHS YEDAVVVPQY ----------

---------- ---------- ---------- ---------- ---------- ----------

---------- ---------- -SWTA----- ---------S FAEYLSGAY- ----------

---------- ---------- -------KAE CLRPDYVAPK ------VPDK SVADSVEALI

GAYLLVCGPV GALKVMKWMG F-WGFPPPQT ALERALGYTF VDRSFLLQAV THASYYRNRL

TDCYQRLEFL GDAVIDYLVT RYLYPGQLTD LRSSLVNNTF FASLVVKYGL HGCFKHCNPS

LFSAIGRFVN YQAECQEPEV EVPKALGDLF ESLMGAVFLD CGMSLDRVWR IIYRMFGQEP

VPPVKELTE- RFRDARFFSC VAKNKKLAKM ALAKKCL

>Ixodes ISCW000890

---EVELLDT AKGQNSIVFL --SSGKTFMV VMLVKEDVES GGKRVLVLVP SASGTRHQKM

MEDYTDLKSA FFSA--DNRT DFPKQFAEHH VLVLTPEAFA HHFVEADNLV VFDDCHYGPV

LMRVLGLAPP LAVILELEDG LQSTACTSRP RETVVLCYTL VTLGPWCAKL RHLLRILKEY

LCGIVFVRQR TAYILSLWLF ITPNFLVGQE EVLQKFRARE CNLLVATSVV EEGIEVPRCN

LVVRFDPPEN LRAYMLSKGK AKASRYFVLV EQILLERSDS LKPPYSMSTA IALVNRYCAK

LPSDTFTRLT PCSLHLPINS PLIVGDPMAK QAVALKTCEE LHKMGELDDP GTTKRRQYYD

KK--PLLDVD HTSP--RYVN RKGVTLPTSS EQTKRAKR-- ---ESLQQKQ IL----VPFP

VFTRSGEVTV LTSQQVGDLR NFHRYTFTNV LRLFVFKEED YLDTVVMPWY FPDAGFETFD

AYYREKYQPL LDVLNLLTPR YVNRKGVTLP TSCLVHPFPA SLWRKAVCLP CVLYRMNSLL

LAEQLRDERP -DLSSHSGLL QALTANDGIN LERLETVGDS FLKYAVTAHL EGKLSHLRSR

QISNLNLYRL GRARRLGGLM VATKFEPSDN WLPPGYVVPP GLERALIPDK SIADCVEALI

GAYLVSCGPR ATLLFMSWLG SAPLYRTLES PLEARIGYRF RDRAYLLQAF THASYHYNRL

TDCYQRLEFL GDAVLDYLIT RHLYPGTLTD LRSALVNNTF FASLAVKYDF HRYFKNVSPG

LFAVIQRFVE TKCECEEAEV EVPKALGDIF ESVAGAIFLD SNMSLDTVWR VYYAMMKPEP

KSPIRELLEL EPQTAKFFVG VGRNKRIAKC TAAKRAL

>Parasteatoda aug3.g914.t1

REYQIEILEE AMRKNTIACL GTGTGKTFIA VLLIREPFEE GGKRIFFLVP TVLVNQQKLV

ISKHTDLKVK GFFGDSWSID DWRKEFIASE VLVMTAEIFR IILDHAFQLL IFDECHYRQA

MPRIFGLSAS LLNLKELEAT MLCGITTTDP DEYVVLRETL KVLGPWCDKL KRLLQIFVAA

ISSIIFVKLR TAYVLCEWLF LKPDFIVGQR KKVRAFRNED CNVLVATCVL EEGMDIRQCN

VVVRFDLPPE FRSYVQSKGR ARASIYVLMV EKLLLSRCDN IIAPYTMTSA VSLINRYCSN

LPSDMATKLV PCELKMPINS PLIISEPMAK MSAALKVCQA LHDIGELNDP GTNRRRQLYD

KHV-PIFLQN ARPPCYLHVI NMTLINPLPN VLNPRGRPII DPQQTSRSLG LLCSSKLPFP

VFTKCGKIMI LSAEEIQRIE EFHRFLFSDA LRLNVFDENL FRDAVLLRSY FDPKTGETFK

YFYKTEYQPL VETL--WKPI YLSPKEVFED SSCFRHPFPA SFLFQVLCLP TALFRLNGLL

LAEEIR-EIK EDLSLKYCLL QALTAGDEFD LERLEMIGDS FLKYVMSIKA EGKLSLFRSR

LIQNLNLYQK AKKKGLGEYM TTTTFSCSST WLPPCYTIEE QIDKKSVSDK SVADSVEALI

GAYLLTSGPK GALKFMSWLG FDNWPPTPSN PIEKKIGYTF KNKAYLLQAF THQSYHYNDI

TDCYQRLEFL GDAVLDYLIT RQLYPGKLTD LRSALVNNIF FASLAVAFSY HEFLKIASPN

LFKLMSHYIE LLEECFELEV EVPKALGDVF ESVAGAIYLD SGMSLDAVWN VYFPMIKPSP

KSPIRELYEM EP-KLMFYDG VGPNKKVAKR SAAKRAL

>Parasteatoda aug3.g8634.t1

REYQVELLDS ARKKNTIICL GTGTGKTFIA VMLIKESLSN GGKRTIFLAP TVLVIQQAKF

VRDCTDLRVG QYFGEKWPKE KWLKEVETHQ VLAMTPDLFC VILYHGFNLI IFDECHYCKI

MPRILGMSAS LINIKQLEAV LKSHVETSKP KEFIVLSYVI SSLGLWCPKM KRLLEVLKPY

LCCLIFVRQR AAYLLSEWLF LSPSYIIGQE EVLQKFRTKL YNIVVATSVI EEGMDIPKCN

LVIRFDRPEN FRAYVQSKGR ARVSHYIMMV EELLLMKCDN LIEPYTLSTA IALVNRYCAK

LPSDTFTRLT PCVLKLPINS PLIEGQWMAK MAVALKTCEE LHKAGELDDP GTTKRRLYYF

KRVADAL-KN SAPPCYLYVF DMKLTCPIPE EQNTRGRKIH DPSDTARGFG ILTGKIIPFP

VFTRSGEVTV LEEEKLDALA FFHRYTFSDV LRLFKFITDD YQDAVVMPWY FPDNEYDTFA

AYYQKKYQPL LDVLNLLTPR YVNRKGVTLP TSCTVHPFPA YLWRKTVCLP CILYRLNSLL

LAEQLRDDNP -DLSKHPGIL QALTANDGIN LERLETVGDS FLKYAITVYL EGKLSYLRSK

QISNYNLYKL GKRKGLGELM VASKFEPYDN WLPPNYVVPK GLEEALIPDK SIADCVEALI

GAYLVSCGSR GALLFMSWLG Y-GFLTPPPS PIEDKIDYSF NDKSYLLQAF THASYRYNTL

TDCYQRLEFL GDAVLDYLIT RHLYPGTLTD LRSALVNNTF FASLAVKYDF HKFFKSISPA

LFNVINKFVQ LKEECEEMEV EVPKALGDIF ESVAGAIYLD SNMSLDTVWR VYYTMMKPEP

KSPIRELLEL EPQTAKF--- ---------- -------
