# Supplementary material for: Comparative Genomics Reveals the Origins and Diversity of Arthropod Immune Systems
Source: Mol Biol Evol. 2015 Apr 22;32(8):2111–29. doi: 10.1093/molbev/msv093 (PMC4833078; doi:10.1093/molbev/msv093)
Supplement: Supplementary Data [file supp_msv093_SUPPLEMENTAL_TablesFigures_Rev1_March15.pdf]

Comparative genomics reveals the origins and diversity of arthropod immune systems

Supplementary Tables and Figures

William J. Palmer* and Francis M. Jiggins

Department of Genetics, University of Cambridge, Downing Street, Cambridge CB2 3EH UK

* corresponding author; w.palmer@gen.cam.ac.uk

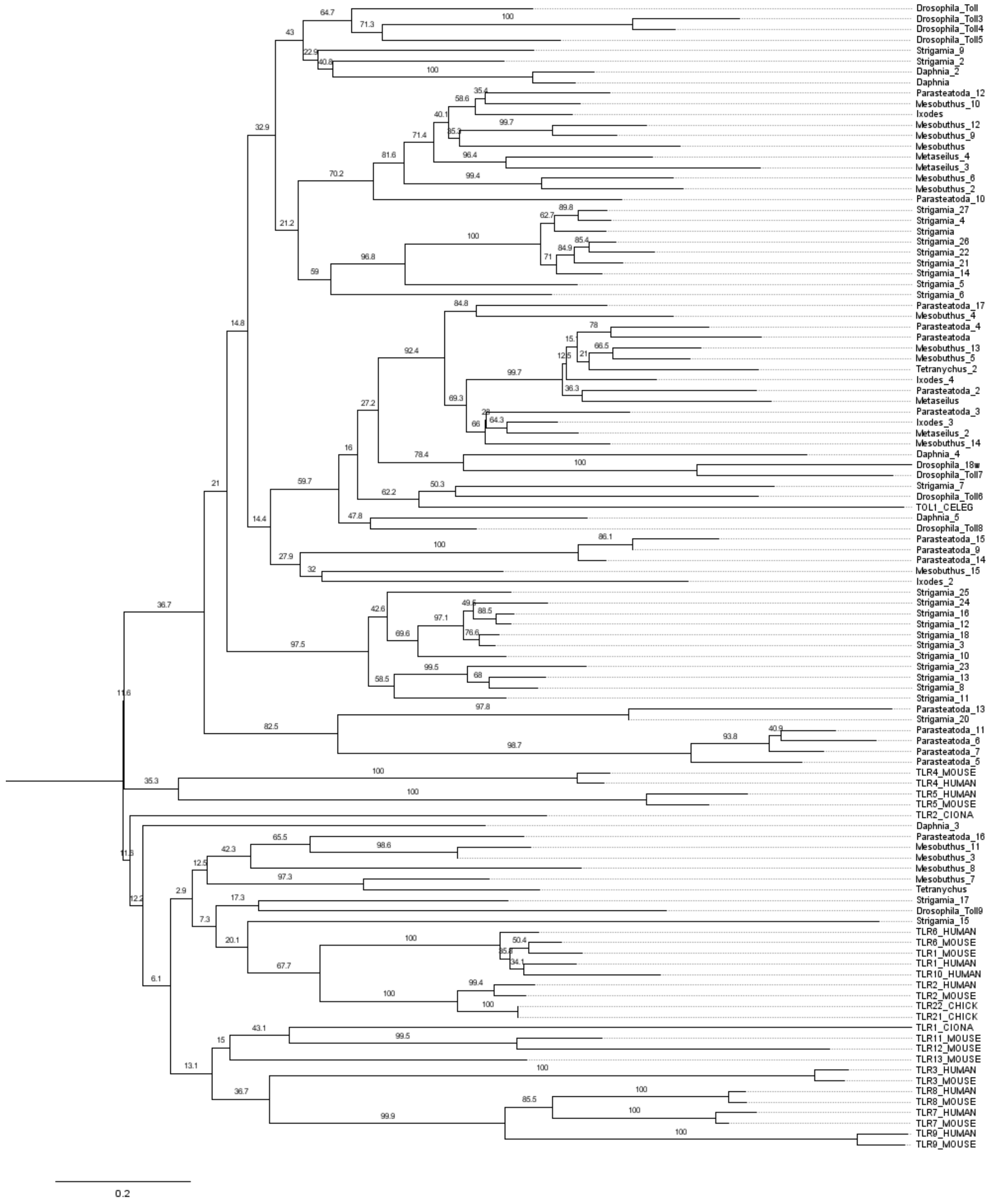

Supplementary Figure 1 – Phylogenetic tree of TLRs from seven species of arthropods, four chordates (human, mouse, chicken and *Ciona*), and the nematode *Caenorhabditis*. The tree was reconstructed by maximum likelihood from the TIR domains and is midpoint rooted. Node labels are bootstrap support from 1000 replicates. Scale bar is substitutions per site.

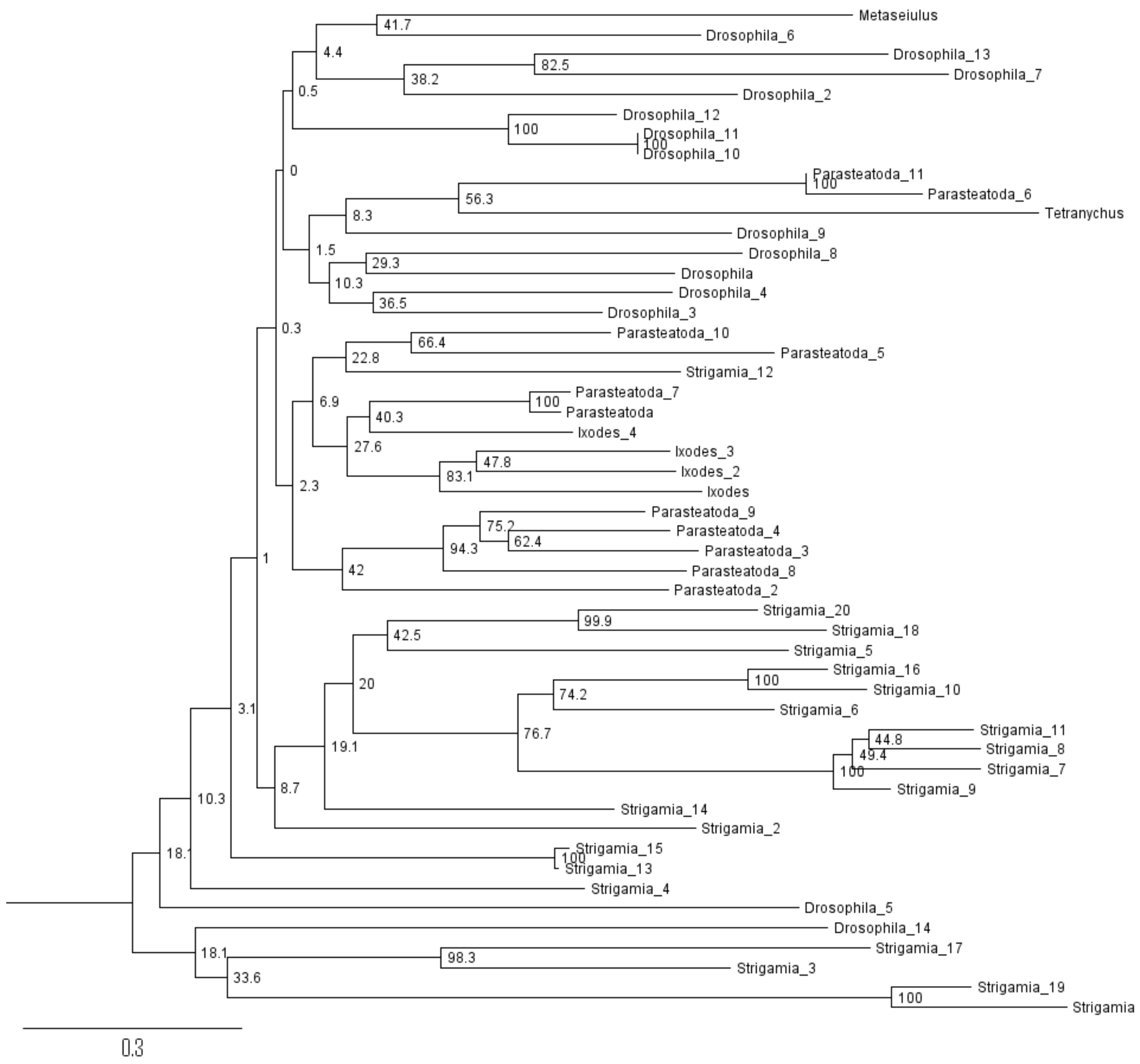

Supplementary Figure 2 – PGRP tree was reconstructed by maximum likelihood tree from the PGRP domain sequences and is midpoint rooted. Node labels are bootstrap support from 1000 replicates. Scale bar is substitutions per site.

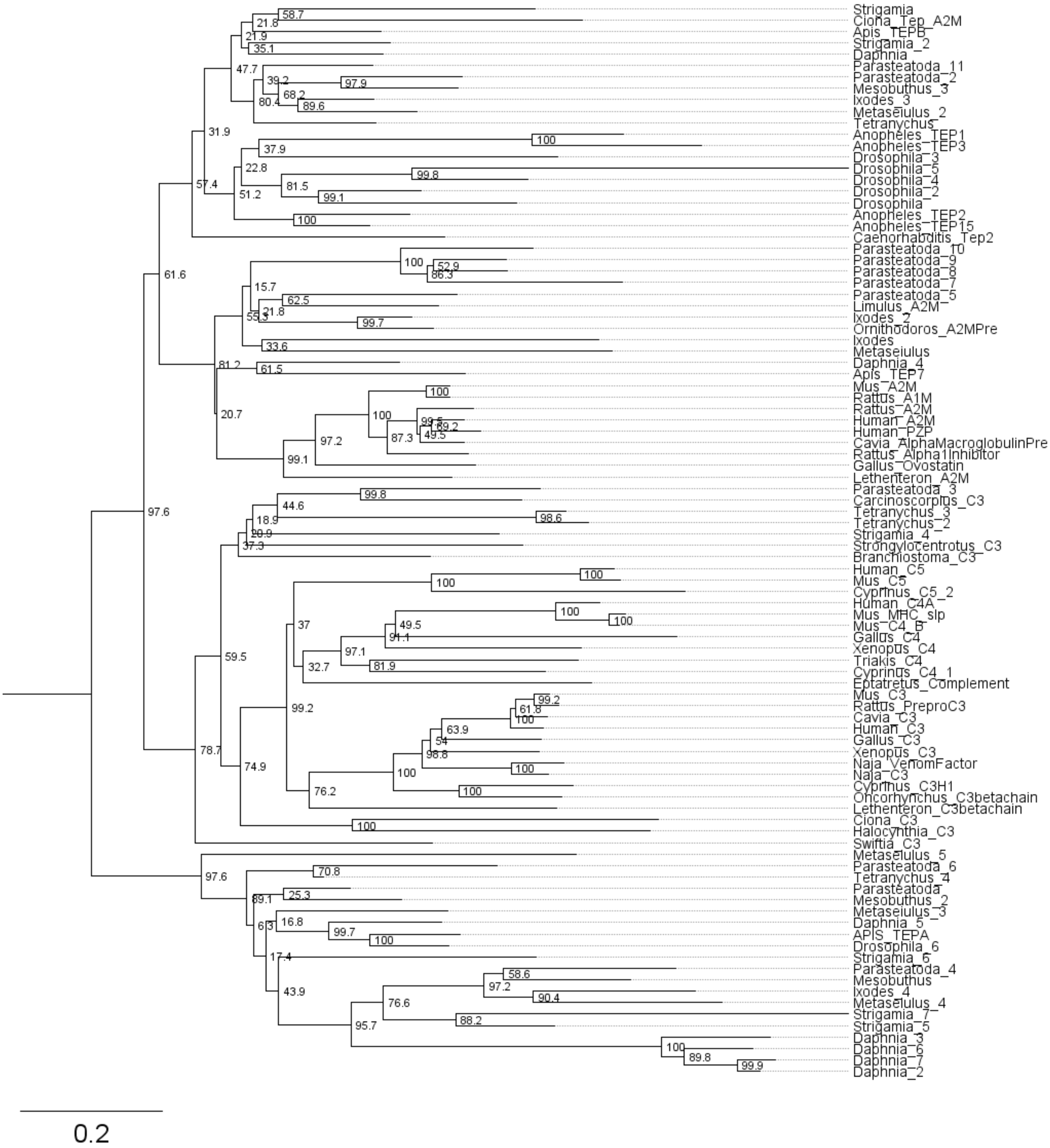

Supplementary Figure 3 - Gene tree of the thioester-containing protein (TEP) family. Sequences include arthropod TEPs, macroglobulin complement related proteins (MCRs), the vertebrate C3, C4 and C5 complement factors, and alpha-2 macroglobulins. In addition to the chelicerate sequences we annotated, we included two arthropod sequences from horseshoe crabs (*Limulus* and *Carcinoscorpius*) and a sequence from the tick *Ornithodoros*. The tree is midpoint rooted and was reconstructed by maximum likelihood. Node labels are bootstrap support from 1000 replicates. Scale bar is substitutions per site.

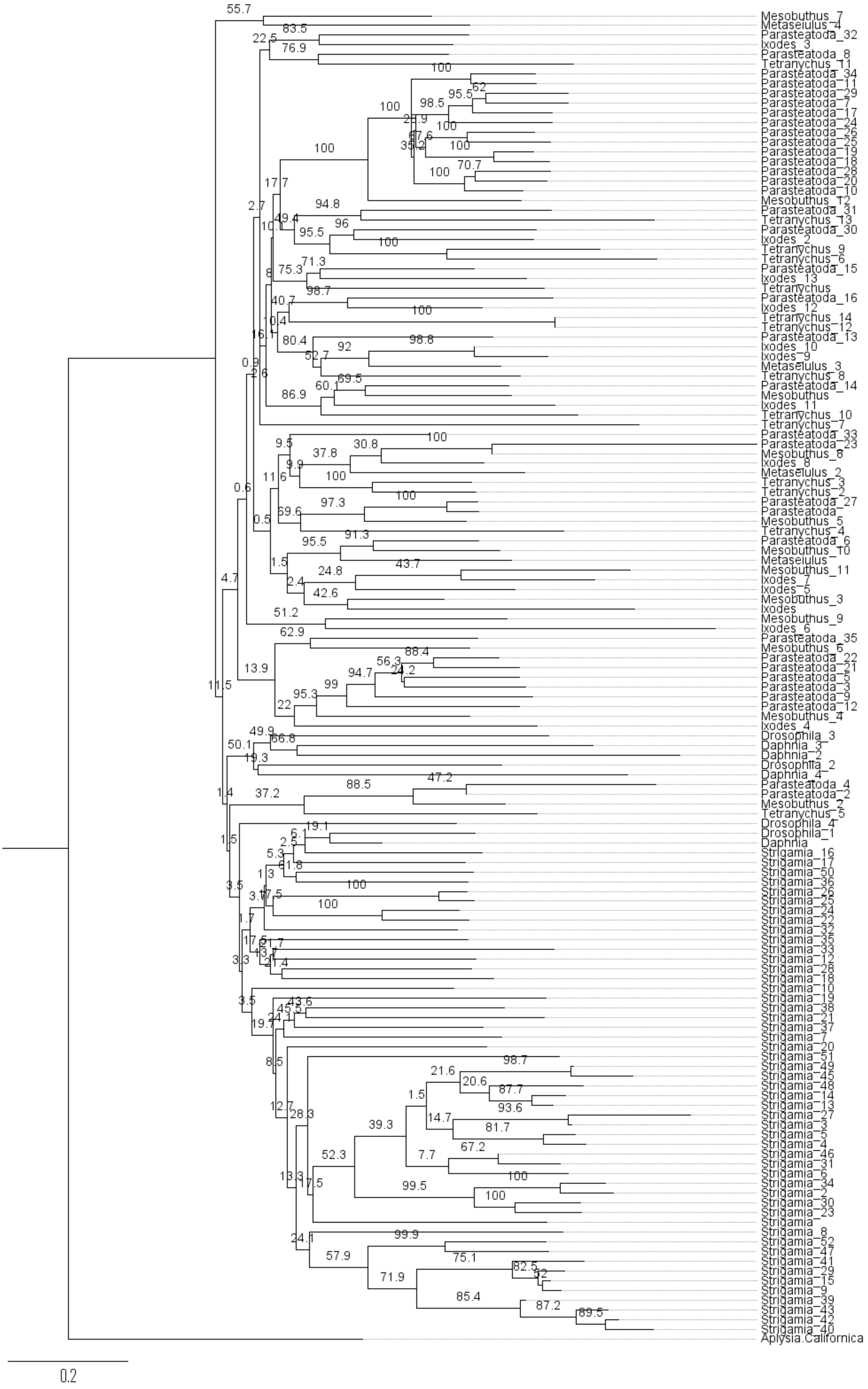

Supplementary Figure 4 - The diversity of Dscam in the arthropods. The tree is reconstructed by maximum likelihood based on complete Dscam amino acid sequence alignment and is rooted using Dscam from the mollusc *Aplysia californica*. Node labels are bootstrap support from 1000 replicates. Scale bar is substitutions per site.

Supplementary table 1 –Protein domains and features considered essential to the immune function of different protein families. Identifiers are for the NCBI conserved domain database (CDD).

Gene Class	Domain	Identifier
PGRP	PGRP	cd06583
BGRP/GNBP	GH16_beta_GRP	cd02179
FREP	Fibrinogen-related domain	cl00085
C-type lectin	CLECT	cd00037
TEP	A2M_2	cl10467
TLR	TIR_2 Superfamily	cl17458
	Leucine Rich Repeat Region	cl19302,cl19073,cl15307,cl19480, cl19479,cl02423
fadd	Death/Death Fadd	cd01670/cd08306
	DED_FADD	cd08336
Tube	Death_Tube	cd08308
Dorsal	RHD-n_Dorsal_Dif	cd07887
	IPT_NFkappaB	cd01177
myd88	Death_MyD88	cd08312
	TIR	smart00255
pelle	Death_Pelle	cd08307
	PTKc	cd00192
RELISH	RHD_n relish	cl08275
	IPT_NFkappaB	cd01177
	ANK	cd00204
PPO	Hemocyanin_M	cl02853
	Tyrosinase	cl02830
DUOX	dual_peroxidase_like	cd09820
	NOX_Duox_like_FAD_NADP	cd06186
Lysozyme	LYZ1	cd00119
	Lysozyme like superfamily	cl00222
AMP	Attacin_C	cl04254
	Attacin_N	cl04252
	Cecropin	cl02832
	Defensin2	cl03093

	Defensin associated knot	cl11589
Argonaute (RNAi)	Piwi_ago-like[b]	cl00628
Dicer (RNAi)[c]	dsRNA_bind	cl04028
	Dicer_dimer	pfam03368
	PAZ	smart00949
	RIBOc	cd00593
	DEXDc	cd00046

[b] PIWI-like genes were identified on the basis of a phylogeny and removed from the analysis

[c]Dicers were not required to have all the domains

Supplementary table 2 - Predicted amidase activity and cellular location of all PGRPs identified in this study. The cysteine and two histidine residues (His1, His2 and Cys) are those shown in Figure 3 of Reiser et al, 2004. The letters refer to the amino acid residue or alignment gap found at these sites.

TreeID	Gene ID	Trans membrane helix	Secretory Pathway	His1	His2	Cys	Predicted amidase activity
Strigamia	SMAR001446-PA	N	Y	H	G	-	NO
Strigamia 2	SMAR007557-PA	N	Y	H	H	C	YES
Strigamia 3	SMAR007558-PA	N	Y	T	E	-	NO
Strigamia 4	SMAR007559-PA	N	Y	H	H	C	YES
Strigamia 5	SMAR007560-PA	N	N	H	H	C	YES
Strigamia 6	SMAR007561-PA	Y	N	H	H	C	YES
Strigamia 7	SMAR007562-PA	N	N	H	H	C	YES
Strigamia 8	SMAR007563-PA	N	N	H	H	C	YES
Strigamia 9	SMAR007564-PA	N	Y	H	-	-	NO
Strigamia 10	SMAR007565-PA	N	N	H	H	C	YES
Strigamia 11	SMAR007566-PA	N	N	H	H	C	YES
Strigamia 12	SMAR009813-PA	N	Y	H	H	C	YES
Strigamia 13	SMAR010214-PA	N	Y	S	H	S	NO
Strigamia 14	SMAR013411-PA	N	Y	H	H	C	YES
Strigamia 15	SMAR014273-PA	Y	N	S	H	S	NO
Strigamia 16	SMAR014565-PA	N	N	H	H	C	YES
Strigamia 17	SMAR014870-PA	N	Y	S	A	-	NO
Strigamia 18	SMAR014909-PA	N	Y	H	H	C	YES
Strigamia 19	SMAR015150-PA	N	Y	H	Y	-	NO
Strigamia 20	SMAR015602-PA	Y	Y	H	H	-	NO
Metaseiulus	gi|391339223|ref| XP_003743951.1|	N	Y	H	H	C	YES
Tetranychus	tetur14g01760.1	N	Y	D	-	-	NO
Ixodes	ISCW024689-RA	N	N	H	-	-	NO
Ixodes 2	ISCW004389-RA	N	N	H	Q	C	NO
Ixodes 3	ISCW024175-RA	N	N	-	Q	C	NO
Ixodes 4	ISCW022212-RA	N	Y	H	H	C	YES
Parasteatoda	aug3.g1176.t1	N	Y	H	H	C	YES
Parasteatoda 2	aug3.g20166.t1	N	Y	S	A	S	NO

Parasteatoda 3	aug3.g20281.t1	N	Y	L	H	S	NO
Parasteatoda 4	aug3.g21135.t1	N	Y	M	H	S	NO
Parasteatoda 5	aug3.g21165.t1	N	N	H	H	C	YES
Parasteatoda 6	aug3.g21166.t1	N	Y	H	H	C	YES
Parasteatoda 7	aug3.g1177.t1	N	Y	H	H	C	YES
Parasteatoda 8	aug3.g12678.t1	N	Y	A	H	S	NO
Parasteatoda 9	aug3.g12679.t1	N	Y	L	H	S	NO
Parasteatoda 10	aug3.g18552.t1	N	Y	K	H	C	NO
Parasteatoda 11	aug3.g18554.t1	N	N	H	Y	-	NO

Supplementary table 3 - Presence/Absence of thioester motif GCGEQ (GCAEQ in some vertebrates)

GeneID	Tree Name	GCGEQ	TE Motif
AF291654	Anopheles_TEP1	GCGEQ	Y
AGAP008364-RA|AGAP008364	Anopheles_TEP15	GCGEQ	Y
AGAP008366-RA|AGAP008366	Anopheles_TEP2	GCGEQ	Y
AGAP010816-RA|AGAP010816	Anopheles_TEP3	ASGAP	N
GB12605|GB12605-RA	Apis_TEP7	GCGEQ	Y
TEPB|GB11563|GB11563-RA	APIS_TEPA	DSAEQ	N
TEPA|GB18789|GB18789-RA	Apis_TEPB	GCGEQ	Y
gi|13928544|dbj|BAB47146.1|	Branchiostoma_C3	GCGEQ	Y
gi|74959685|sp|O46015|O46015_CAEEL	Caenorhabditis_Tep2	GCGEQ	Y
AF517564_Carcinoscorpius	Carcinoscorpius_C3	GCGEQ	Y
gi|81871982|sp|Q60486|Q60486_CAVPO	Cavia_AlphaMacroglobulinPre	GCGEQ	Y
gi|544053|sp|P12387.2|CO3_CAVPO	Cavia_C3	GCGEQ	Y
gi|18074013|emb|CAC85959.1|	Ciona_C3	GCGEQ	Y
gi|19032251|emb|CAD24311.1|	Ciona_Tep_A2M	GCGEQ	Y
AB016210_Cyprinus	Cyprinus_C3H1	GCGEQ	Y
gi|9453863|dbj|BAB03284.1|	Cyprinus_C4_1	GCAEQ	Y
gi|24785187|dbj|BAC23058.1|	Cyprinus_C5_2	GSAEV	N
DappuP313404	Daphnia	GCGEQ	Y
DappuP320144	Daphnia_2	RGTEA	N
DappuP320145	Daphnia_3	RGTEA	N
DappuP61510	Daphnia_4	GCGEQ	Y
DappuP229736	Daphnia_5	DCAEQ	N
DappuP320142	Daphnia_6	HGTKA	N
DappuP320143	Daphnia_7	RGTEA	N
FBgn0041183|FBtr0080811|TepI	Drosophila	GCGEQ	Y
FBgn0041182|FBtr0079510|TepII	Drosophila_2	GCGEQ	Y
FBgn0041181|FBtr0079477|TepIII	Drosophila_3	GCGEQ	Y
FBgn0041180|FBtr0331329|TepIV	Drosophila_4	GCGEQ	Y
FBgn0032808|FBtr0331331|CG13079|Tep5	Drosophila_5	GTGEL	N

FBgn0020240|FBtr0079543|Mcr|Tep6	Drosophila_6	ESGEQ	N
Z11595_Eptatretus	Eptatretus_Complement	GCGEQ	Y
gi|82136251|sp|Q90633|Q90633_CHICK	Gallus_C3	GCGEQ	Y
gi|7512215|pir||T28153	Gallus_C4	GCGEQ	Y
gi|1171932|sp|P20740.3|OVOS_CHICK	Gallus_Ovostatin	GNGEQ	Y
AB006964_Halocynthia	Halocynthia_C3	GCGEQ	Y
gi|308153640|sp|P01023.3|A2MG_HUMAN	Human_A2M	GCGEQ	Y
gi|119370332|sp|P01024.2|CO3_HUMAN	Human_C3	GCGEQ	Y
K02403_Human	Human_C4A	GCGEQ	Y
gi|166900096|sp|P01031.4|CO5_HUMAN	Human_C5	GSAEA	N
X54380_Human	Human_PZP	GCGEQ	Y
ISCW003923-RA	Ixodes	GCGEQ	Y
ISCW023777-RA	Ixodes_2	GCGEQ	Y
ISCW020822-RA	Ixodes_3	GCGEQ	Y
ISCW003089-RA	Ixodes_4	KSGEH	N
D13567_Lethenteron	Lethenteron_A2M	GCGEQ	Y
gi|1352101|sp|Q00685.1|CO3_LAMJA	Lethenteron_C3betachain	GCGEQ	Y
gi|2073373|dbj|BAA19844.1|	Limulus_A2M	GCGEQ	Y
MMa28752	Mesobuthus	KSGDH	N
MMa43260	Mesobuthus_2	WCGEQ	N
MMa54913	Mesobuthus_3	GCGEQ	Y
gi|391330844|ref|XP_003739862.1|	Metaseiulus	GCGEQ	Y
gi|391331531|ref|XP_003740198.1|	Metaseiulus_2	GCGEQ	Y
gi|391330201|ref|XP_003739552.1|	Metaseiulus_3	FCGEQ	N
gi|391337209|ref|XP_003742963.1|	Metaseiulus_4	QSGEH	N
gi|391339074|ref|XP_003743878.1|	Metaseiulus_5	GTAEA	N
gi|338817897|sp|Q61838.3|A2M_MOUSE	Mus_A2M	GCGEQ	Y
gi|341940525|sp|P01027.3|CO3_MOUSE	Mus_C3	GCGEQ	Y
gi|341940526|sp|P01029.3|CO4B_MOUSE	Mus_C4_B	GCAEQ	Y
gi|116608|sp|P06684.2|CO5_MOUSE	Mus_C5	GSAEA	N
M21576_Mouse	Mus_MHC_slp	SCAEQ	N
gi|399269|sp|Q01833.1|CO3_NAJNA	Naja_C3	GCGEQ	Y
gi|2118405|pir||I51018	Naja_VenomFactor	GCGEQ	Y

gi|1352103|sp|P98093.1|CO3_ONCMY	Oncorhynchus_C3betachain	GCGEQ	Y
gi|22901939|gb|AAN10129.1|	Ornithodoros_A2MPre	GCGEQ	Y
aug3.g11592.t1	Parasteatoda	WCGEQ	N
aug3.g3271.t1	Parasteatoda_10	GCGEQ	Y
aug3.g9168.t1	Parasteatoda_11	GCGEQ	Y
aug3.g14443.t1	Parasteatoda_2	GCGEQ	Y
aug3.g19014.t1	Parasteatoda_3	GCGEQ	Y
aug3.g2288.t1	Parasteatoda_4	KSGDF	N
aug3.g25461.t2	Parasteatoda_5	GCGEQ	Y
aug3.g2883.t3	Parasteatoda_6	WCGEQ	N
aug3.g3268.t2	Parasteatoda_7	GCGEQ	Y
aug3.g3269.t2	Parasteatoda_8	GCGEQ	Y
aug3.g3270.t2	Parasteatoda_9	GCGEQ	Y
gi|119370261|sp|P06238.2|A2MG_RAT	Rattus_A1M	GCGEQ	Y
gi|112893|sp|P14046.1|A1I3_RAT	Rattus_A2M	GCGEQ	Y
M77183_R.norvegicus	Rattus_Alpha1Inhibitor	GCGEQ	Y
X52477_Rat	Rattus_PreproC3	GCGEQ	Y
SMAR009298-PA	Strigamia	GCGEQ	Y
SMAR006270-PA	Strigamia_2	GCGEQ	Y
SMAR005770-PA	Strigamia_4	GCGEQ	Y
SMAR002371-PA	Strigamia_5	RSAEM	N
SMAR006274-PA	Strigamia_6	DSAEQ	N
SMAR008374-PA	Strigamia_7	-----	N
AF025526_Strongylocentrotus	Strongylocentrotus_C3	GCGEQ	Y
gi|27357203|gb|AAN86548.1|	Swiftia_C3	GCGEQ	Y
tetur08g02040.1	Tetranychus	GCGEQ	Y
tetur04g03640.1	Tetranychus_2	GCGEQ	Y
tetur04g03660.1	Tetranychus_3	GCGEQ	Y
tetur04g01020.1	Tetranychus_4	GCAEQ	?
gi|34097968|dbj|BAC82347.1|	Triakis_C4	GCAEQ	Y
gi|758425|gb|AAB60608.1|	Xenopus_C3	GCGEQ	Y
D78003_Xenopus	Xenopus_C4	GCAEQ	Y

Supplementary table 4 - *Drosophila melanogaster* reference gene set compiled from Immunodb [10], IIID [52], Flybase [53] and [54]

	SYMBOL	NAME	Flybase ID
**PGRP**			
FBgn0030310|FBtr0073509|PGRP-SA	PGRP-SA	Peptidoglycan recognition protein SA	CG11709
FBgn0030695|FBtr0074183|PGRP-LE	PGRP-LE	Peptidoglycan recognition protein LE	CG8995
FBgn0033327|FBtr0088708|PGRP-SC1b	PGRP-SC1b	PGRP-SC1b	CG8577
FBgn0035806|FBtr0076807|PGRP-SD	PGRP-SD	PGRP-SD	CG7496
FBgn0035975|FBtr0076435|PGRP-LA	PGRP-LA	Peptidoglycan recognition protein LA	CG32042
FBgn0035976|FBtr0089491|PGRP-LC	PGRP-LC	Peptidoglycan recognition protein LC	CG4432
FBgn0035977|FBtr0076439|PGRP-LF	PGRP-LF	Peptidoglycan recognition protein LF	CG4437
FBgn0037906|FBtr0306097|PGRP-LB	PGRP-LB	Peptidoglycan recognition protein LB	CG14704
FBgn0043575|FBtr0088709|PGRP-SC2	PGRP-SC2	PGRP-SC2	CG14745
FBgn0043576|FBtr0088707|PGRP-SC1a	PGRP-SC1a	PGRP-SC1a	CG14746
FBgn0043577|FBtr0333627|PGRP-SB2	PGRP-SB2	PGRP-SB2	CG9697
FBgn0043578|FBtr0075348|PGRP-SB1	PGRP-SB1	PGRP-SB1	CG9681
FBgn0260458|FBtr0114577|PGRP-LD	PGRP-LD	Peptidoglycan recognition protein LD	CG33717
**GNBP**			
FBgn0040323|FBtr0075050	GNBP1	Gram-negative bacteria binding protein 1	CG6895
FBgn0040322|FBtr0075094	GNBP2	Gram-negative bacteria binding protein 2	CG4144
FBgn0040321|FBtr0076510	GNBP3	Gram-negative bacteria binding protein 3	CG5008
**FREP**			
FBgn0051832|FBtr0080726	CG31832	-	CG31832
FBgn0050281|FBtr0071760	CG30281	-	CG30281
FBgn0050280|FBtr0300928	CG30280	-	CG30280
FBgn0038365|FBtr0307203	CG9593	-	CG9593
FBgn0036929|FBtr0074904	CG7668	-	CG7668
FBgn0035452|FBtr0273204	CG10359	-	CG10359
FBgn0034160|FBtr0087033	CG5550	-	CG5550

FBgn0033312|FBtr0310502	CG8642	-	CG8642
FBgn0031804|FBtr0079280	CG9500	-	CG9500
FBgn0030880|FBtr0074495	CG6788	-	CG6788
FBgn0030163|FBtr0071429	CG1791	-	CG1791
FBgn0030164|FBtr0071408	CG1889	-	CG1889
FBgn0003326|FBtr0087855	sca	scabrous	CG17579

TEP

FBgn0041183|FBtr0080811	Tep1	Thioester-containing protein 1	CG18096
FBgn0041182|FBtr0079510	Tep2	Thioester-containing protein 2	CG7052
FBgn0041181|FBtr0079477	Tep3	Thioester-containing protein 3	CG7068
FBgn0041180|FBtr0331329	Tep4	Thioester-containing protein 4	CG10363
FBgn0032808|FBtr0331331	Tep5	Thioester-containing protein 5	CG13079
FBgn0020240|FBtr0079543	Mcr	Macroglobulin complement-related	CG7586

DSCAM

DSCAM1	Dscam1	Down syndrome cell adhesion molecule 1	CG17800
DSCAM2	Dscam2	Down syndrome cell adhesion molecule 2	CG42256
DSCAM3	Dscam3	Down syndrome cell adhesion molecule 3	CG31190
DSCAM4	Dscam4	Down syndrome cell adhesion molecule 4	CG42330

Nimrods

FBgn0027594	drpr	Draper	CG2086
FBgn0243514	eater	Eater	CG6124
FBgn0261514|FBtr0304720	NimA	Nimrod A	CG42282
FBgn0259896|FBtr0310075	NimC1	Nimrod C1	CG8942
FBgn0028939|FBtr0080589	NimC2	Nimrod C2	CG18146
FBgn0001967|FBtr0290243	NimC3	Nimrod C3	CG16880
FBgn0260011|FBtr0080592	NimC4	Nimrod C4	CG16876
FBgn0027929|FBtr0310074	NimB1	Nimrod B1	CG33119

FBgn0028543|FBtr0080618	NimB2	Nimrod B2	CG31839
FBgn0028542|FBtr0080617	NimB4	Nimrod B4	CG33115
FBgn0028936|FBtr0080586	NimB5	Nimrod B5	CG16873

SPZ

SPZ	spz	spatzle	CG6134
SPZ2	spz2	spatzle 2	CG42576
SPZ3	spz3	spatzle 3	CG7104
SPZ4	spz4	spatzle 4	CG14928
SPZ5	spz5	spatzle 5	CG9972
SPZ6	spz6	spatzle 6	CG9196

TLR

FBgn0036978|FBtr0078240	Toll-9	Toll-9	CG5528
FBgn0036494|FBtr0075614	Toll-6	Toll-6	CG7250
FBgn0034476|FBtr0086336	Toll-7	Toll-7	CG8595
FBgn0032095|FBtr0079768	Toll-4	Toll-4	CG18241
FBgn0029114|FBtr0075607	Tollo	Tollo	CG6890
FBgn0026760|FBtr0080502	Tehao	Tehao	CG7121
FBgn0015770|FBtr0081585	MstProx	MstProx	CG1149
FBgn0004364|FBtr0086309	18w	18 wheeler	CG8896
FBgn0262473|FBtr0330154	Tl	Toll	CG5490

TOLLPATH

MyD88	Myd88	Myd88	CG2078
Tube	tub	tube	CG10520
Pelle	pll	pelle	CG5974
Dorsal	dl	dorsal	CG6667
Cactus	cact	cactus	CG5848
Dif	Dif	Dorsal-related immunity factor	CG6794

IMD PATH

IMD	imd	immune deficiency	CG5576
TAK1	Tak1	TGF-beta activated kinase 1	CG18492
IKKg (Kenny)	key	kenny	CG16910
IKKb (Ird5)	ird5	immune response deficient 5	CG4201
FADD	Fadd	Fas-associated death domain ortholog	CG12297
DREDD	Dredd	Death related ced-3/Nedd2-like protein	CG7486
Relish	Rel	Relish	CG11992

JAKSTAT

Dome(less)	dome	domeless	CG14226
Hop	hop	hopscotch	CG1594
Stat92	Stat92E	Signal-transducer and activator of transcription protein at 92E	CG4257

JNK

Hemipterous	hep	hemipterous	CG4353
Bsk (JNK)	bsk	basket	CG5680

PPO

FBgn0261362|FBtr0302291 | PPO1	proPO-A1	prophenol oxidase A1	CG42639
FBgn0033367|FBtr0088663 | PPO2	proPO45	prophenoloxidase 45	CG8193
FBgn0261363|FBtr0302290 | PPO3	proPO59	prophenoloxidase 59	CG42640

DUOX

FBgn0031464	DUOX	Dual Oxidase	CG3131

Lysozyme

Lysozyme	-	LYS1	CG8492
Lysozyme	LysB	LYS8	CG1179
Lysozyme	LysC	LYS5	CG9111
Lysozyme	LysD	LYS7	CG9118
Lysozyme	LysE	LYS9	CG1180
Lysozyme	LysP	LYS6	CG9116
Lysozyme	LysS	LYS4	CG1165
Lysozyme	LysX	LYS2	CG9120
Lysozyme	-	LYS10	CG16756
Lysozyme	-	LYS11	CG16799
Lysozyme	-	LYS12	CG11159
Lysozyme	-	LYS13	CG30062
Lysozyme	-	LYS3	CG7798

Antimicrobial Petptides

Immunodb:AMP1	AttA	Attacin	CG10146
Immunodb:AMP2	AttB	Attacin	CG18372
Immunodb:AMP3	AttC	Attacin	CG4740
Immunodb:AMP4	AttD	Attacin	CG7629
Immunodb:AMP5	Anp	Cecropin	CG1361
Immunodb:AMP6	CecA1	Cecropin	CG1365
Immunodb:AMP7	CecA2	Cecropin	CG1367
Immunodb:AMP8	CecB	Cecropin	CG1878
Immunodb:AMP9	CecC	Cecropin	CG1373
Immunodb:AMP10	Def	Defensin	CG1385
Immunodb:AMP11	Dpt	Diptericin	CG12763
Immunodb:AMP12	DptB	Diptericin	CG10794
Immunodb:AMP13	Dro	Drosocin	CG10816
Immunodb:AMP14	Dro2	Drosomycin	CG32279
Immunodb:AMP15	Dro3	Drosomycin	CG32283
Immunodb:AMP16	Dro4	Drosomycin	CG32282

Immunodb:AMP17	Dro5	Drosomycin	CG10812
Immunodb:AMP18	Dro6	Drosomycin	CG32268
Immunodb:AMP19	Drs	Drosomycin	CG10810
Immunodb:AMP20	Drs-like	Drosomycin	CG32274
Immunodb:AMP21	Mtk	Metchnikowin	CG8175

Supplementary table 5 – A complete list* of all putative immunity genes identified in 7 Arthropod protein sets as identified in this study. *Clip domain serine proteases and their inhibitors may be found in supplementary table 6.

	GeneID	*Drosophila* top blast hit (Name)	*Drosophila* top blast hit (FlyBase ID)
Daphnia pulex			
**<u>Recognition and Related</u>**			
BGRP/GNBP	DappuP332783	GNBP1	CG6895
	DappuP28775	GNBP2	CG4144
Tep and MCR like	DappuP313404	Tep3	CG7068
	DappuP320144	Mcr	CG7586
	DappuP320145	Mcr	CG7586
	DappuP61510	Tep2	CG7052
	DappuP229736	Mcr	CG7586
	DappuP320142	Mcr	CG7586
	DappuP320143	Mcr	CG7586
Fibrinogen Related	DappuP233432	Nrx-IV	CG6827
	DappuP233433	axo	CG42664
	DappuP206258	CG8642	CG8642
	DappuP221139	CG41520	CG41520
	DappuP235623	CG45105	CG31291
	DappuP313952	Nrx-IV	CG6827
	DappuP314880	CG17574	CG17574
	DappuP100997	AdamTS-A	CG14869
	DappuP316232	Nrx-IV	CG6827
	DappuP316370	Nrx-IV	CG6827
	DappuP316536	Nrx-IV	CG6827
	DappuP223872	LanB2	CG3322
	DappuP101703	Mtor	CG8274
	DappuP195987	CG41520	CG41520
	DappuP303744	Nrx-IV	CG6827
	DappuP303745	Nrx-IV	CG6827
	DappuP303746	Nrx-IV	CG6827
	DappuP103274	CG11873	CG11873

	GeneID	*Drosophila* top blast hit (Name)	*Drosophila* top blast hit (FlyBase ID)
	DappuP103449	CG31832	CG31832
	DappuP103450	CG41520	CG41520
	DappuP244609	Nrx-IV	CG6827
	DappuP225479	Khc	CG7765
	DappuP246540	prtp	CG1837
	DappuP248859	CenB1A	CG6742
	DappuP106904	lva	CG6450
	DappuP323209	brp	CG42344
	DappuP307466	Nrx-IV	CG6827
	DappuP252960	tectonic	CG42731
	DappuP253847	CG42255	CG42255
	DappuP309407	AdamTS-A	CG14869
	DappuP257184	Nrx-IV	CG6827
	DappuP257243	Nrx-IV	CG6827
	DappuP330534	sca	CG17579
	DappuP265459	AdamTS-A	CG14869
	DappuP118473	Nrx-IV	CG6827
	DappuP120497	CG31076	CG31076
Dscam	DappuP127336	Dscam1	CG17800
	DappuP331991	Dscam3	CG31190
	DappuP43826	Dscam2	CG42256
	DappuP48415	Dscam2	CG42256
Nimrod	DappuP47122	drpr	CG2086
Spatzle	DappuP47496	spz3	CG7104
	DappuP309408	spz5	CG9972
	DappuP317626	spz6	CG9196
	DappuP317627	spz6	CG9196
Toll and TLR	DappuP190084	Tl	CG5490
	DappuP97929	Tl	CG5490
	DappuP314608	Toll-9	CG5528
	DappuP128614	Tollo	CG6890
	DappuP65779	Tollo	CG6890

	GeneID	*Drosophila* top blast hit (Name)	*Drosophila* top blast hit (FlyBase ID)
**Signalling**			
Imd pathway	DappuP313869	imd	CG5576
	DappuP310110	Fadd	CG12297
	DappuP225308	Dredd	CG7486
	DappuP301643	Tak1	CG18492
	DappuP317376	ird5	CG4201
	DappuP303763	key	CG16910
	DappuP329057	Rel	CG11992
	DappuP65051	Rel	CG11992
	DappuP300285	Rel	CG11992
	DappuP237874	Rel	CG11992
Toll Pathway	DappuP305569	Myd88	CG2078
	DappuP314397	pll	CG5974
	DappuP331814	pll	CG5974
	DappuP52849	dl	CG6667
	DappuP62213	cact	CG5848
	DappuP65048	cact	CG5848
Jnk Pathway			
	DappuP127867	bsk	CG5680
	DappuP187828	bsk	CG5680
	DappuP32176	hep	CG4353
JAK/STAT Pathway			
	DappuP100606	dome	CG14226
	DappuP311837	hop	CG1594
	DappuP97641	Stat92E	CG4257
**Effectors**			
ProPhenol Oxidases	DappuP326755	proPO59	CG2952
Dual Oxidases	DappuP320872	Duox	CG3131

	GeneID	*Drosophila* top blast hit (Name)	*Drosophila* top blast hit (FlyBase ID)
	DappuP329115	Duox	CG3131
Lysozymes	DappuP300634	-	-
RNAi Ago2	DappuP311791	Argonaute-1	CG6671
RNAi Dcr2	DappuP309030	Dicer-1	CG4792
	DappuP329028	Dicer-2	CG6493

Strigamia maritima

Recognition and Related

PGRPs	SMAR001446-PA	Peptidoglycan recognition protein SA	CG11709
	SMAR007557-PA	PGRP-SB1	CG9681
	SMAR007558-PA	Peptidoglycan recognition protein SA	CG11709
	SMAR007559-PA	Peptidoglycan recognition protein SA	CG11709
	SMAR007560-PA	Peptidoglycan recognition protein SA	CG11709
	SMAR007561-PA	PGRP-SC2	CG14745
	SMAR007562-PA	PGRP-SC1b	CG8577
	SMAR007563-PA	PGRP-SC1b	CG8577
	SMAR007564-PA	PGRP-SC1b	CG8577
	SMAR007565-PA	PGRP-SC2	CG14745
	SMAR007566-PA	PGRP-SC1b	CG8577
	SMAR009813-PA	Peptidoglycan recognition protein LF	CG4437
	SMAR010214-PA	PGRP-SC2	CG14745
	SMAR013411-PA	PGRP-SC2	CG14745
	SMAR014273-PA	PGRP-SC2	CG14745
	SMAR014565-PA	Peptidoglycan recognition protein	CG4437

	GeneID	*Drosophila* top blast hit (Name)	*Drosophila* top blast hit (FlyBase ID)
		LF	
	SMAR014870-PA	Peptidoglycan recognition protein LF	CG4437
	SMAR014909-PA	PGRP-SB1	CG9681
	SMAR015150-PA	Peptidoglycan recognition protein SA	CG11709
	SMAR015602-PA	PGRP-SC2	CG14745
BGRP/GNBPs	SMAR001813-PA	Gram-negative bacteria binding protein 3	CG5008
TEPs and MCRs	SMAR009298-PA	Thioester-containing protein 1	CG18096
	SMAR006270-PA	Thioester-containing protein 2	CG7052
	SMAR006272-PA	Thioester-containing protein 2	CG7052
	SMAR005770-PA	Thioester-containing protein 2	CG7052
	SMAR002371-PA	Macroglobulin complement-related	CG7586
	SMAR006274-PA	Macroglobulin complement-related	CG7586
	SMAR008374-PA	Macroglobulin complement-related	CG7586
Fibrinogen Related	SMAR001380-PA	scabrous	CG17579
	SMAR000567-PA	scabrous	CG17579
	SMAR003278-PA	-	CG41520
	SMAR003523-PA	-	CG41520
	SMAR003660-PA	-	CG30280
	SMAR003974-PA	NFAT homolog	CG11172
	SMAR006481-PA	-	CG41520
	SMAR007089-PA	-	CG41520
	SMAR009371-PA	-	CG41520
	SMAR009547-PA	-	CG41520
	SMAR010882-PA	-	CG41520
	SMAR011186-PA	Vinculin	CG3299
	SMAR012847-PA	-	CG41520
	SMAR012852-PA	-	CG41520

	GeneID	*Drosophila* top blast hit (Name)	*Drosophila* top blast hit (FlyBase ID)
	SMAR012853-PA	-	CG41520
	SMAR012855-PA	-	CG41520
	SMAR013431-PA	-	CG41520
	SMAR015487-PA	-	CG41520
	SMAR015779-PA	-	CG41520
Dscam	SMAR000414-PA	Down syndrome cell adhesion molecule 1	CG17800
	SMAR004329-PA	Down syndrome cell adhesion molecule 4	CG42330
	SMAR005038-PA	Down syndrome cell adhesion molecule 1	CG17800
	SMAR005057-PA	Down syndrome cell adhesion molecule 4	CG42330
	SMAR005058-PA	Down syndrome cell adhesion molecule 1	CG17800
	SMAR005060-PA	Down syndrome cell adhesion molecule 1	CG17800
	SMAR005787-PA	Down syndrome cell adhesion molecule 1	CG17800
	SMAR005788-PA	Down syndrome cell adhesion molecule 1	CG17800
	SMAR007303-PA	Down syndrome cell adhesion molecule 1	CG17800
	SMAR008780-PA	Down syndrome cell adhesion molecule 1	CG17800
	SMAR000616-PA	Down syndrome cell adhesion molecule 4	CG42330
	SMAR009342-PA	Down syndrome cell adhesion molecule 4	CG42330
	SMAR009349-PA	Down syndrome cell adhesion molecule 4	CG42330
	SMAR009501-PA	Down syndrome cell adhesion molecule 4	CG42330
	SMAR009834-PA	Down syndrome cell adhesion molecule 4	CG42330

GeneID	*Drosophila* top blast hit (Name)	*Drosophila* top blast hit (FlyBase ID)
SMAR009857-PA	Down syndrome cell adhesion molecule 1	CG17800
SMAR009858-PA	Down syndrome cell adhesion molecule 4	CG42330
SMAR010628-PA	Down syndrome cell adhesion molecule 1	CG17800
SMAR010629-PA	Down syndrome cell adhesion molecule 4	CG42330
SMAR010632-PA	Down syndrome cell adhesion molecule 4	CG42330
SMAR010689-PA	Down syndrome cell adhesion molecule 4	CG42330
SMAR000686-PA	Down syndrome cell adhesion molecule 1	CG17800
SMAR010692-PA	Down syndrome cell adhesion molecule 1	CG17800
SMAR010787-PA	Down syndrome cell adhesion molecule 1	CG17800
SMAR010788-PA	Down syndrome cell adhesion molecule 4	CG42330
SMAR010961-PA	Down syndrome cell adhesion molecule 4	CG42330
SMAR010994-PA	Down syndrome cell adhesion molecule 4	CG42330
SMAR011182-PA	Down syndrome cell adhesion molecule 1	CG17800
SMAR011319-PA	Down syndrome cell adhesion molecule 1	CG17800
SMAR011548-PA	Down syndrome cell adhesion molecule 1	CG17800
SMAR011561-PA	Down syndrome cell adhesion molecule 1	CG17800
SMAR011562-PA	Down syndrome cell adhesion molecule 4	CG42330
SMAR000919-PA	Down syndrome cell adhesion	CG17800

GeneID	*Drosophila* top blast hit (Name)	*Drosophila* top blast hit (FlyBase ID)
	molecule 1	
SMAR011671-PA	Down syndrome cell adhesion molecule 4	CG42330
SMAR011672-PA	Down syndrome cell adhesion molecule 1	CG17800
SMAR011726-PA	Down syndrome cell adhesion molecule 1	CG17800
SMAR011727-PD	Down syndrome cell adhesion molecule 1	CG17800
SMAR012077-PA	Down syndrome cell adhesion molecule 4	CG42330
SMAR001994-PA	Down syndrome cell adhesion molecule 4	CG42330
SMAR012080-PA	Down syndrome cell adhesion molecule 1	CG17800
SMAR012325-PA	Down syndrome cell adhesion molecule 1	CG17800
SMAR012326-PA	Down syndrome cell adhesion molecule 2	CG42256
SMAR013455-PA	Down syndrome cell adhesion molecule 2	CG42256
SMAR014023-PA	Down syndrome cell adhesion molecule 3	CG31190
SMAR015611-PB	Down syndrome cell adhesion molecule 1	CG17800
SMAR002919-PA	Down syndrome cell adhesion molecule 4	CG42330
SMAR015640-PA	Down syndrome cell adhesion molecule 1	CG17800
SMAR003721-PA	Down syndrome cell adhesion molecule 1	CG17800
SMAR004310-PA	Down syndrome cell adhesion molecule 1	CG17800
SMAR004313-PA	Down syndrome cell adhesion molecule 1	CG17800

	GeneID	*Drosophila* top blast hit (Name)	*Drosophila* top blast hit (FlyBase ID)
Nimrods	SMAR004984-PA	draper	CG2086
Spatzles	SMAR009848-PA	spatzle 3	CG7104
	SMAR012695-PA	spatzle 6	CG9196
Toll and TLR	SMAR012279-PA	Toll	CG5490
	SMAR011407-PA	Toll	CG5490
	SMAR011269-PA	Tollo	CG6890
	SMAR010912-PA	Toll	CG5490
	SMAR014647-PA	Toll	CG5490
	SMAR008553-PA	Toll	CG5490
	SMAR008376-PA	Toll-6	CG7250
	SMAR006691-PA	Tollo	CG6890
	SMAR005841-PA	Tollo	CG6890
	SMAR014575-PA	Tollo	CG6890
	SMAR005346-PA	Tollo	CG6890
	SMAR005007-PA	Tollo	CG6890
	SMAR005008-PA	Tollo	CG6890
	SMAR005010-PA	Toll	CG5490
	SMAR004813-PA	-	CG14762
	SMAR004578-PA	Tollo	CG6890
	SMAR004491-PA	Toll-9	CG5528
	SMAR003805-PA	Tollo	CG6890
	SMAR015400-PA	Toll	CG5490
	SMAR002710-PA	Tollo	CG6890
	SMAR002611-PA	Toll	CG5490
	SMAR002366-PA	Toll	CG5490
	SMAR002249-PA	Tollo	CG6890
	SMAR002076-PA	Tollo	CG6890
	SMAR001938-PA	Tollo	CG6890
	SMAR001266-PA	Toll	CG5490
	SMAR000970-PA	Toll	CG5490

Signalling

	GeneID	*Drosophila* top blast hit (Name)	*Drosophila* top blast hit (FlyBase ID)
Imd Pathway	SMAR000319-PA	immune deficiency	CG5576
	SMAR007362-PA	Fas-associated death domain ortholog	CG12297
	SMAR008273-PA	TGF-beta activated kinase 1	CG18492
	SMAR004900-PA	TGF-beta activated kinase 1	CG18492
	SMAR009684-PA	TGF-beta activated kinase 1	CG18492
	SMAR002690-PA	immune response deficient 5	CG4201
	SMAR012259-PA	kenny	CG16910
	SMAR011690-PA	Relish	CG11992
Toll Pathway	SMAR008506-PA	Myd88	CG2078
	SMAR014285-PA	pelle	CG5974
	SMAR003060-PA	cactus	CG5848
	SMAR014442-PA	dorsal	CG6667
JNK Pathway			
	SMAR006177-PA	hemipterous	CG4353
	SMAR008084-PA	basket	CG5680
JAK/STAT Pathway			
	SMAR011060-PA	domeless	CG14226
	SMAR011063-PA	domeless	CG14226
	SMAR001417-PA	hopscotch	CG1594
	SMAR014120-PA	Signal-transducer and activator of transcription protein at 92E	CG4257
**<u>Effectors</u>**			
ProPhenol Oxidases	SMAR009245-PA	prophenoloxidase 45	CG8193
Dual Oxidases	SMAR006405-PA	Dual oxidase	CG3131
Lysozymes	SMAR001436-PA	Lysozyme C	CG9111
	SMAR006863-PA	Lysozyme S	CG1165
	SMAR011592-PA	Lysozyme P	CG9116

	GeneID	*Drosophila* top blast hit (Name)	*Drosophila* top blast hit (FlyBase ID)
	SMAR012085-PA	-	CG8492
	SMAR012086-PA	-	CG8492
RNAi Ago2	SMAR010593-PA	Argonaute-1	CG6671
	SMAR015088-PA	aubergine	CG6137
RNAi Dcr2	SMAR009083-PA	Dicer-1	CG4792
	SMAR007746-PA	Dicer-1	CG4792
Metaseiulus occidentalis			
**Recognition and Related**			
PGRP	gi|391339223|ref|XP_003743951.1|	Peptidoglycan recognition protein LF	CG4437
TEP and MCR like	gi|391330844|ref|XP_003739862.1|	Thioester-containing protein 4	CG10363
	gi|391331531|ref|XP_003740198.1|	Thioester-containing protein 3	CG7068
	gi|391330201|ref|XP_003739552.1|	Macroglobulin complement-related	CG7586
	gi|391337209|ref|XP_003742963.1|	Macroglobulin complement-related	CG7586
	gi|391339074|ref|XP_003743878.1|	Macroglobulin complement-related	CG7586
Fibrinogen Related	gi|391335595|ref|XP_003742175.1|	-	CG31832
Dscams	gi|391326293|ref|XP_003737652.1|	Down syndrome cell adhesion molecule 1	CG17800
	gi|391334253|ref|XP_003741520.1|	Down syndrome cell adhesion molecule 1	CG17800
	gi|391337343|ref|XP_003743029.1|	Down syndrome cell adhesion molecule 4	CG42330
	gi|391343779|ref|XP_003746183.1|	Down syndrome cell adhesion molecule 4	CG42330
Spatzles	gi|391337754|ref|XP_003743230.1|	spatzle 3	CG7104
Tolls and TLRs	gi|391327659|ref|XP_003738314.1|	Tollo	CG6890
	gi|391335441|ref|XP_003742102.1|	Tollo	CG6890
	gi|391337512|ref|XP_003743111.1|	Toll	CG5490
	gi|391342046|ref|XP_003745335.1|	Toll	CG5490

	GeneID	*Drosophila* top blast hit (Name)	*Drosophila* top blast hit (FlyBase ID)
**Signalling**			
Imd Pathway	gi|391344683|ref|XP_003746625.1|	TGF-beta activated kinase 1	CG18492
	gi|391327264|ref|XP_003738123.1|	immune response deficient 5	CG4201
	gi|391327266|ref|XP_003738124.1|	immune response deficient 5	CG4201
Toll Pathway	gi|391346209|ref|XP_003747371.1|	Myd88	CG2078
	gi|391348537|ref|XP_003748503.1|	pelle	CG5974
	gi|391333133|ref|XP_003740976.1|	cactus	CG5848
	gi|391334748|ref|XP_003741763.1|	dorsal	CG6667
JNK Pathway	gi|391341374|ref|XP_003745005.1|	hemipterous	CG4353
	gi|391337438|ref|XP_003743075.1|	basket	CG5680
	gi|391329708|ref|XP_003739310.1|	basket	CG5680
	gi|391327502|ref|XP_003738237.1|	basket	CG5680
JAK/STAT Pathway	gi|391339487|ref|XP_003744080.1|	domeless	CG14226
	gi|391326368|ref|XP_003737689.1|	Signal-transducer and activator of transcription protein at 92E	CG4257
	gi|391326370|ref|XP_003737690.1|	Signal-transducer and activator of transcription protein at 92E	CG4257
	gi|391326336|ref|XP_003737673.1|	Signal-transducer and activator of transcription protein at 92E	CG4257
	gi|391326334|ref|XP_003737672.1|	Signal-transducer and activator of transcription protein at 92E	CG4257
	gi|391334191|ref|XP_003741491.1|	Signal-transducer and activator of transcription protein at 92E	CG4257
**Effectors**			
Dual Oxidases	gi|391347609|ref|XP_003748052.1|	Dual oxidase	CG3131
Lysozymes	gi|391338518|ref|XP_003743605.1|	Lysozyme S	CG1165
	gi|391339068|ref|XP_003743875.1|	-	CG16799
	gi|391346777|ref|XP_003747645.1|	-	CG16799
	gi|391347082|ref|XP_003747794.1|	-	CG16799

	GeneID	*Drosophila* top blast hit (Name)	*Drosophila* top blast hit (FlyBase ID)
	gi|391347161|ref|XP_003747833.1|	-	CG16799
RNAi Ago2	gi|391334350|ref|XP_003741568.1|	Argonaute 1	CG6671
RNAi Dcr2	gi|391328911|ref|XP_003738926.1|	Dicer 1	CG4792
	gi|391332351|ref|XP_003740599.1|	Dicer 1	CG4792
	gi|391345092|ref|XP_003746827.1|	Dicer 2	CG6493
	gi|391348645|ref|XP_003748555.1|	Dicer 1	CG4792
	gi|391348710|ref|XP_003748587.1|	Dicer 1	CG4792
Tetranychus urticae			
**Recognition and Related**			
PGRPs	tetur14g01760.1	PGRP-LF	CG4437
TEP and MCR like	tetur08g02040.1	Tep2	CG7052
	tetur04g03640.1	Tep2	CG7052
	tetur04g03660.1	Tep2	CG7052
	tetur04g01020.1	Mcr	CG7586
Fibrinogen Related	tetur16g01240.1	CG41520	CG41520
	tetur18g01690.1	CG41520	CG41520
Dscam	tetur07g00600.1	Dscam4	CG42330
	tetur17g02480.1	Dscam4	CG42330
	tetur17g02500.1	Dscam1	CG17800
	tetur19g01590.1	Dscam4	CG42330
	tetur19g02050.1	Dscam4	CG42330
	tetur21g02250.1	Dscam4	CG42330
	tetur07g02520.1	Dscam4	CG42330
	tetur08g01100.1	Dscam4	CG42330
	tetur09g01130.1	Dscam1	CG17800
	tetur11g03400.1	Dscam4	CG42330
	tetur12g03540.1	Dscam4	CG42330
	tetur16g02990.1	Dscam2	CG42256
	tetur16g03020.1	Dscam4	CG42330
	tetur16g03946.1	Dscam2	CG42256
Spatzles	tetur04g00590.1	spz5	CG9972
	tetur10g05070.1	spz4	CG14928

	GeneID	*Drosophila* top blast hit (Name)	*Drosophila* top blast hit (FlyBase ID)
	tetur11g00320.1	spz6	CG9196
	tetur11g06410.1	spz5	CG9972
	tetur20g01990.1	spz5	CG9972
Toll and TLRs	tetur36g00940.1	Toll-9	CG5528
	tetur09g04990.1	Tollo	CG6890
**Signalling**			
Imd Pathway	tetur07g04360.1	Tak1	CG18492
	tetur07g00650.1	Rel	CG11992
Toll Pathway	tetur11g01630.1	Myd88	CG2078
	tetur01g00840.1	pll	CG5974
	tetur03g06520.1	pll	CG5974
	tetur14g01540.1	cact	CG5848
	tetur11g04270.1	dl	CG6667
JNK Pathway	tetur02g11770.1	hep	CG4353
	tetur07g07260.1	bsk	CG5680
JAK/STAT Pathway	tetur05g02920.1	dome	CG14226
	tetur02g05790.1	hop	CG1594
	tetur36g00600.1	Stat92E	CG4257
	tetur17g02010.1	Stat92E	CG4257
RNAi Ago2	tetur02g10560.1	AGO1	CG6671
	tetur02g10570.1	AGO1	CG6671
	tetur02g10580.1	AGO1	CG6671
	tetur09g00620.1	AGO1	CG6671
	tetur09g03140.1	AGO1	CG6671
	tetur04g01190.1	AGO1	CG6671
RNAi Dcr2	tetur07g00990.1	Dcr-2	CG6493
**Effectors**			
Dual Oxidases	tetur08g07420.1	Duox	CG3131
	tetur18g01610.1	Duox	CG3131

	GeneID	*Drosophila* top blast hit (Name)	*Drosophila* top blast hit (FlyBase ID)
Lysozymes	tetur09g04040.1	-	CG8492
	tetur21g01120.1	-	CG16799
	tetur21g02460.1	-	CG16799

Ixodes scapularis

Recognition and Related

PGRPs	ISCW024689-RA	PGRP-LB	CG14704
	ISCW004389-RA	PGRP-LB	CG14704
	ISCW024175-RA	PGRP-LF	CG4437
	ISCW022212-RA	PGRP-SC1b	CG8577
TEP and MCR like	ISCW003923-RA	Tep4	CG10363
	ISCW023777-RA	Tep2	CG7052
	ISCW020822-RA	Tep2	CG7052
	ISCW003089-RA	Mcr	CG7586
Fibrinogen Related	ISCW022063-RA	CG8642	CG8642
	ISCW024644-RA	sca	CG17579
	ISCW002664-RA	CG41520	CG41520
	ISCW013746-RA	CG41520	CG41520
	ISCW003711-RA	CG30281	CG30281
	ISCW024400-RA	sca	CG17579
	ISCW010128-RA	CG1791	CG1791
	ISCW012248-RA	CG41520	CG41520
	ISCW024182-RA	CG30281	CG30281
	ISCW024309-RA	sca	CG17579
	ISCW004981-RA	CG30281	CG30281
	ISCW009412-RA	CG41520	CG41520
	ISCW024554-RA	sca	CG17579
	ISCW024686-RA	CG41520	CG41520
	ISCW024835-RA	sca	CG17579
	ISCW000158-RA	CG41520	CG41520
	ISCW024445-RA	CG9500	CG9500
	ISCW024504-RA	CG5550	CG5550

	GeneID	*Drosophila* top blast hit (Name)	*Drosophila* top blast hit (FlyBase ID)
	ISCW024801-RA	CG9593	CG9593
	ISCW024486-RA	CG9593	CG9593
	ISCW024548-RA	CG9593	CG9593
	ISCW024814-RA	sca	CG17579
	ISCW001478-RA	sca	CG17579
	ISCW008812-RA	CG30281	CG30281
	ISCW024125-RA	CG9500	CG9500
	ISCW024256-RA	CG31832	CG31832
Dscams	ISCW001673-RA	Dscam1	CG17800
	ISCW022831-RA	Dscam4	CG42330
	ISCW022837-RA	Dscam4	CG42330
	ISCW023871-RA	Dscam1	CG17800
	ISCW023928-RA	Dscam1	CG17800
	ISCW001732-RA	Dscam4	CG42330
	ISCW016847-RA	Dscam1	CG17800
	ISCW017407-RA	Dscam1	CG17800
	ISCW017597-RA	Dscam4	CG42330
	ISCW017602-RA	Dscam4	CG42330
	ISCW020407-RA	Dscam4	CG42330
	ISCW022820-RA	Dscam4	CG42330
	ISCW022826-RA	Dscam4	CG42330
Nimrods	ISCW020553-RA	drpr	CG2086
Spatzles	ISCW018887-RA	spz3	CG7104
	ISCW002691-RA	spz6	CG9196
Toll and TLR	ISCW018193-RA	Tl	CG5490
	ISCW007724-RA	Tl	CG5490
	ISCW022740-RA	Tollo	CG6890
	ISCW020989-RA	Tollo	CG6890
**Signalling**			
Imd Pathway	ISCW023496-RA	Tak1	CG18492
	ISCW002130-RA	ird5	CG4201
	ISCW011555-RA	key	CG16910

	GeneID	*Drosophila* top blast hit (Name)	*Drosophila* top blast hit (FlyBase ID)
	ISCW018935-RA	Rel	CG11992
Toll Pathway	ISCW008802-RA	Myd88	CG2078
	ISCW001463-RA	pll	CG5974
	ISCW007160-RA	pll	CG5974
	ISCW019520-RA	cact	CG5848
	ISCW000140-RA	dl	CG6667
JNK Pathway	ISCW006910-RA	hep	CG4353
	ISCW020577-RA	bsk	CG5680
JAK/STAT Pathway	ISCW001458-RA	dome	CG14226
	ISCW013495-RA	dome	CG14226
	ISCW016158-RA	hop	CG1594
	ISCW005692-RA	Stat92E	CG4257
**Effectors**			
Dual Oxidases	ISCW007865-RA	Duox	CG3131
Lysozyme	ISCW001645-RA	LysP	CG9116
	ISCW001646-RA	LysP	CG9116
	ISCW017129-RA	-	CG8492
	ISCW018431-RA	-	CG8492
RNAi Ago2	ISCW015916-RA	AGO1	CG6671
	ISCW021130-RA	AGO1	CG6671
	ISCW011768-RA	AGO1	CG6671
RNAi Dcr2	ISCW000889-RA	Dcr-1	CG4792
AMP Defensin_2	ISCW005926-RA	Def	CG1385
	ISCW005927-RA	Osi5	CG15590
	ISCW005928-RA	Drs	CG10810
	ISCW016747-RA	Pk92B	CG4720
	ISCW024381-RA	Drsl5	CG10812

Mesobuthus martensii

Recognition and Related

	GeneID	*Drosophila* top blast hit (Name)	*Drosophila* top blast hit (FlyBase ID)
TEP and MCR like	MMa28752	Mcr	CG7586
	MMa43260	Mcr	CG7586
	MMa54913	Tep2	CG7052
Fibrinogen Related	MMa13045	CG30280	CG30280
	MMa33084	CG30280	CG30280
	MMa21157	CG41520	CG41520
	MMa22385	CG41520	CG41520
	MMa13471	CG41520	CG41520
	MMa51082	CG41520	CG41520
	MMa08459	CG41520	CG41520
	MMa10841	sca	CG17579
	MMa14599	CG41520	CG41520
	MMa38459	CG41520	CG41520
	MMa28819	sca	CG17579
	MMa24044	CG41520	CG41520
	MMa22389	CG30280	CG30280
	MMa10830	CG41520	CG41520
	MMa13044	CG41520	CG41520
	MMa33083	CG10359	CG10359
	MMa14600	CG41520	CG41520
	MMa22390	CG41520	CG41520
	MMa13043	CG41520	CG41520
	MMa13624	CG41520	CG41520
	MMa22394	CG41520	CG41520
	MMa38435	CG41520	CG41520
	MMa31589	CG30280	CG30280
	MMa03337	CG41520	CG41520
Dscams	MMa08972	Dscam4	CG42330
	MMa43828	Dscam1	CG17800
	MMa47028	Dscam4	CG42330
	MMa54582	Dscam1	CG17800
	MMa12454	Dscam1	CG17800

	GeneID	*Drosophila* top blast hit (Name)	*Drosophila* top blast hit (FlyBase ID)
	MMa26231	Dscam1	CG17800
	MMa27504	Dscam4	CG42330
	MMa27661	Dscam4	CG42330
	MMa36242	Dscam1	CG17800
	MMa41956	Dscam4	CG42330
	MMa41957	Dscam4	CG42330
	MMa43724	Dscam4	CG42330
Spatzles	MMa30204	spz3	CG7104
	MMa17304	spz6	CG9196
	MMa21952	spz4	CG14928
	MMa25818	spz3	CG7104
	MMa26169	spz5	CG9972
Toll and TLR	MMa46071	Tl	CG5490
	MMa34077	CG7896	CG7896
	MMa52435	Toll-6	CG7250
	MMa37636	Tollo	CG6890
	MMa37517	Tl	CG5490
	MMa02436	Tehao	CG7121
	MMa51810	Toll-9	CG5528
	MMa42574	Tl	CG5490
	MMa11502	Tl	CG5490
	MMa34079	Toll-9	CG5528
	MMa22782	Tl	CG5490
	MMa07778	Tollo	CG6890
	MMa13223	Toll-6	CG7250
	MMa40477	Tollo	CG6890
**Signalling**			
Imd Pathway	MMa32087 (fadd)	CG30349	CG30349
	MMa02027 (fadd)	CG1638	CG1638
	MMa30295 (fadd)	CG15082	CG15082
	MMa44238	Dredd	CG7486
	MMa52113	Tak1	CG18492

	GeneID	*Drosophila* top blast hit (Name)	*Drosophila* top blast hit (FlyBase ID)
	MMa20866	ird5	CG4201
	MMa26000	Rel	CG11992
Toll Pathway	MMa19524	Myd88	CG2078
	MMa20752	tub	CG10520
	MMa29082	cact	CG5848
	MMa29443	cact	CG5848
	MMa35756	dl	CG6667
JNK Pathway	MMa38230	hep	CG4353
	MMa30726	bsk	CG5680
	MMa26791	bsk	CG5680
JAK/STAT Pathway	MMa17359	Stat92E	CG4257
	MMa12827	Stat92E	CG4257
**Effectors**			
ProPhenolOxidases	MMa13066	proPO59	CG2952
	MMa33538	proPO45	CG8193
	MMa36925	proPO45	CG8193
	MMa43766	proPO45	CG8193
	MMa43767	proPO45	CG8193
	MMa45521	proPO45	CG8193
	MMa54611	proPO59	CG2952
	MMa54612	proPO45	CG8193
Dual Oxidases	MMa56943	Duox	CG3131
	MMa00235	Duox	CG3131
Lysozymes	MMa08231	LysP	CG9116
	MMa11065	-	CG8492
	MMa18052	LysP	CG9116
	Mma33840	LysP	CG9116

	GeneID	*Drosophila* top blast hit (Name)	*Drosophila* top blast hit (FlyBase ID)
	Mma38927	-	CG8492
	Mma40083	-	CG16799
	Mma42986	-	CG8492
	Mma42987	-	CG16756
	Mma50853	-	CG8492
RNAi Ago2	MMa10623	AGO1	CG6671
	MMa15079	AGO1	CG6671
	MMa13679	AGO1	CG6671
	MMa36176	AGO1	CG6671
	MMa10549	AGO1	CG6671
	MMa13534	AGO1	CG6671
AMP defensin_2	MMa09284	-	CG9698
	MMa09285	-	CG11655
	MMa36626	mgl	CG42611
	MMa39355	-	CG4324
	MMa39356	-	CG4324
Parasteatoda tepidariorum			
**Recognition and Related**			
PGRPs	aug3.g1176.t1	PGRP-SC2	CG14745
	aug3.g20166.t1	PGRP-SC1b	CG8577
	aug3.g20281.t1	PGRP-SB1	CG9681
	aug3.g21135.t1	PGRP-SC2	CG14745
	aug3.g21165.t1	PGRP-SC2	CG14745
	aug3.g21166.t1	PGRP-LB	CG14704
	aug3.g1177.t1	PGRP-SC2	CG14745
	aug3.g12678.t1	PGRP-SC2	CG14745
	aug3.g12679.t1	PGRP-SC2	CG14745
	aug3.g18552.t1	PGRP-SB1	CG9681
	aug3.g18554.t1	PGRP-LB	CG14704
Tep and MCR like	aug3.g11592.t1	Mcr	CG7586

	GeneID	*Drosophila* top blast hit (Name)	*Drosophila* top blast hit (FlyBase ID)
	aug3.g14443.t1	Tep3	CG7068
	aug3.g19014.t1	Tep2	CG7052
	aug3.g2288.t1	Mcr	CG7586
	aug3.g25461.t2	Tep2	CG7052
	aug3.g2883.t3	Mcr	CG7586
	aug3.g3268.t2	Tep4	CG10363
	aug3.g3269.t2	Tep2	CG7052
	aug3.g3270.t2	Tep2	CG7052
	aug3.g3271.t1	Tep4	CG10363
	aug3.g9168.t1	Tep2	CG7052
Fibrinogen related	aug3.g248.t1	sca	CG17579
	aug3.g11460.t1	CG1889	CG1889
	aug3.g12207.t1	CG41520	CG41520
	aug3.g12735.t1	CG41520	CG41520
	aug3.g13841.t2	axo	CG43225
	aug3.g14668.t1	CG41520	CG41520
	aug3.g14670.t1	CG41520	CG41520
	aug3.g14671.t2	CG41520	CG41520
	aug3.g16622.t1	sca	CG17579
	aug3.g18262.t1	CG41520	CG41520
	aug3.g18310.t1	CG30281	CG30281
	aug3.g20703.t1	CG41520	CG41520
	aug3.g21239.t1	CG41520	CG41520
	aug3.g2672.t1	CG41520	CG41520
	aug3.g353.t1	CG41520	CG41520
	aug3.g355.t3	CG41520	CG41520
	aug3.g356.t1	CG41520	CG41520
	aug3.g6232.t1	CG41520	CG41520
	aug3.g6421.t1	CG41520	CG41520
	aug3.g943.t1	sca	CG17579
Dscam	aug3.g10045.t1	Dscam1	CG17800
	aug3.g1203.t1	Dscam4	CG42330

GeneID	*Drosophila* top blast hit (Name)	*Drosophila* top blast hit (FlyBase ID)
aug3.g14097.t2	Dscam4	CG42330
aug3.g14551.t1	Dscam4	CG42330
aug3.g14610.t1	Dscam4	CG42330
aug3.g15517.t4	Dscam1	CG17800
aug3.g16920.t1	Dscam1	CG17800
aug3.g17018.t2	Dscam4	CG42330
aug3.g17489.t1	Dscam4	CG42330
aug3.g18884.t1	Dscam4	CG42330
aug3.g20363.t1	Dscam4	CG42330
aug3.g2126.t2	Dscam1	CG17800
aug3.g23711.t1	Dscam4	CG42330
aug3.g23720.t1	Dscam1	CG17800
aug3.g25152.t1	Dscam4	CG42330
aug3.g25153.t2	Dscam4	CG42330
aug3.g25671.t1	Dscam1	CG17800
aug3.g25674.t1	Dscam1	CG17800
aug3.g26388.t1	Dscam1	CG17800
aug3.g27791.t2	Dscam4	CG42330
aug3.g2782.t3	Dscam4	CG42330
aug3.g2979.t1	Dscam1	CG17800
aug3.g3156.t1	Dscam4	CG42330
aug3.g4548.t1	Dscam1	CG17800
aug3.g5120.t1	Dscam1	CG17800
aug3.g5125.t2	Dscam1	CG17800
aug3.g5556.t2	Dscam4	CG42330
aug3.g6282.t1	Dscam4	CG42330
aug3.g661.t1	Dscam1	CG17800
aug3.g7222.t1	Dscam1	CG17800
aug3.g7225.t1	Dscam4	CG42330
aug3.g7229.t2	Dscam4	CG42330
aug3.g9149.t1	Dscam1	CG17800
aug3.g9642.t1	Dscam4	CG42330

	GeneID	*Drosophila* top blast hit (Name)	*Drosophila* top blast hit (FlyBase ID)
	aug3.g9655.t3	Dscam4	CG42330
Nimrods	aug3.g23881.t1	drpr	CG2086
	aug3.g23882.t1	drpr	CG2086
	aug3.g6804.t1	drpr	CG2086
Spatzles	aug3.g13823.t1	spz3	CG7104
	aug3.g15385.t1	spz6	CG9196
	aug3.g22009.t1	spz5	CG9972
	aug3.g4804.t1	spz4	CG14928
	aug3.g7738.t1	spz	CG6134
	aug3.g8985.t1	spz5	CG9972
	aug3.g984.t1	spz3	CG7104
Toll and TLR	aug3.g494.t1	Toll-6	CG7250
	aug3.g1692.t1	Tollo	CG6890
	aug3.g2549.t1	Tollo	CG6890
	aug3.g6063.t1	Tollo	CG6890
	aug3.g11043.t2	Tl	CG5490
	aug3.g12127.t1	Tl	CG5490
	aug3.g12128.t3	Tl	CG5490
	aug3.g13589.t1	Tollo	CG6890
	aug3.g17600.t3	Tl	CG5490
	aug3.g18079.t1	Tl	CG5490
	aug3.g18263.t1	Tl	CG5490
	aug3.g19333.t1	Tl	CG5490
	aug3.g20004.t2	Tl	CG5490
	aug3.g20240.t1	Tl	CG5490
	aug3.g20941.t1	Tollo	CG6890
	aug3.g25912.t1	Toll-6	CG7250
**Signalling**			
Imd Pathway	aug3.g20760.t1	Fadd	CG12297
	aug3.g22957.t1	Fadd	CG12297
	aug3.g21200.t1	Dredd	CG7486
	aug3.g3694.t1	Tak1	CG18492

	GeneID	*Drosophila* top blast hit (Name)	*Drosophila* top blast hit (FlyBase ID)
	aug3.g14578.t1	ird5	CG4201
	aug3.g9911.t1	key	CG16910
	aug3.g21479.t1	Rel	CG11992
	aug3.g5506.t2	Rel	CG11992
	aug3.g5507.t1	Rel	CG11992
Toll Pathway	aug3.g17480.t1	Myd88	CG2078
	aug3.g3068.t1	pll	CG5974
	aug3.g26918.t1	pll	CG5974
	aug3.g18414.t1	cact	CG5848
	aug3.g11642.t1	dl	CG6667
	aug3.g16812.t1	dl	CG6667
	aug3.g5492.t1	dl	CG6667
JNK Pathway	aug3.g3148.t1	hep	CG4353
	aug3.g13874.t1	bsk	CG5680
	aug3.g4516.t2	bsk	CG5680
JAK/STAT Pathway	aug3.g1187.t1	dome	CG14226
	aug3.g4975.t2	dome	CG14226
	aug3.g4567.t1	Stat92E	CG4257
	aug3.g22960.t1	Stat92E	CG4257
**Effectors**			
ProPhenol Oxidases	aug3.g14602.t1	proPO45	CG8193
	aug3.g11546.t1	proPO59	CG2952
	aug3.g4195.t1	proPO59	CG2952
	aug3.g4196.t1	proPO45	CG8193
	aug3.g4197.t1	proPO45	CG8193
	aug3.g14600.t1	proPO45	CG8193
	aug3.g14601.t1	proPO45	CG8193

	GeneID	*Drosophila* top blast hit (Name)	*Drosophila* top blast hit (FlyBase ID)
	aug3.g14603.t1	proPO59	CG2952
	aug3.g18082.t1	proPO59	CG2952
	aug3.g26249.t1	proPO45	CG8193
	aug3.g26250.t1	proPO45	CG8193
	aug3.g4193.t1	proPO45	CG8193
	aug3.g4194.t1	proPO45	CG8193
Dual Oxidases	aug3.g4371.t1	Duox	CG3131
	aug3.g7524.t1	Duox	CG3131
	aug3.g27392.t3	Duox	CG3131
Lysozymes	aug3.g13279.t1	LysX	CG9120
	aug3.g18600.t1	-	CG8492
	aug3.g24582.t1	LysP	CG9116
RNAi Ago2	aug3.g11588.t1	AGO1	CG6671
	aug3.g12795.t1	AGO1	CG6671
	aug3.g12797.t1	AGO1	CG6671
	aug3.g12799.t1	AGO1	CG6671
RNAi Dcr2	aug3.g914.t1	Dcr-1	CG4792

Supplementary Table 6 - Clip Domain Serine Proteases and Serine Protease Inhibitors identified in this study.

	Taxon	GeneID
**Clip Domain Serine Proteases**	Daphnia	DappuP305928
	Daphnia	DappuP226573
	Daphnia	DappuP269840
	Strigamia	SMAR012578
	Strigamia	SMAR011880
	Strigamia	SMAR009761
	Strigamia	SMAR014508
	Metaseiulus	gi|391332259
	Metaseiulus	gi|391334724
	Metaseiulus	gi|391339096

	**Taxon**	**GeneID**
	Metaseiulus	gi|391344904
	Metaseiulus	gi|391337480
	Tetranychus	tetur09g00280.1
	Tetranychus	tetur13g03390.1
	Tetranychus	tetur14g03480.1
	Ixodes	ISCW002695
	Ixodes	ISCW002697
	Ixodes	ISCW018541
	Ixodes	ISCW009167
	Mesobuthus	MMa27185
	Mesobuthus	MMa14655
	Mesobuthus	MMa31060
	Mesobuthus	MMa33514
	Mesobuthus	MMa31634
	Mesobuthus	MMa33966
	Mesobuthus	MMa31651
	Parasteatoda	aug3.g7646.t1
	Parasteatoda	aug3.g8570.t1
	Parasteatoda	aug3.g8879.t1
	Parasteatoda	aug3.g15441.t1
	Parasteatoda	aug3.g15442.t1
	Parasteatoda	aug3.g17419.t1
	Parasteatoda	aug3.g26401.t1
	Parasteatoda	aug3.g26402.t1
**Serine Protease Inhibitors (Serpins)**	Daphnia	DappuP189444
	Daphnia	DappuP51792
	Daphnia	DappuP53805
	Daphnia	DappuP56449

Taxon	GeneID
Daphnia	DappuP63029
Daphnia	DappuP64475
Strigamia	SMAR000663
Strigamia	SMAR001099
Strigamia	SMAR003045
Strigamia	SMAR003048
Strigamia	SMAR003087
Strigamia	SMAR004057
Strigamia	SMAR004060
Strigamia	SMAR004061
Strigamia	SMAR005784
Strigamia	SMAR013388
Strigamia	SMAR015154
Metaseiulus	gi|391325485
Metaseiulus	gi|391326191
Metaseiulus	gi|391326226
Metaseiulus	gi|391331479
Metaseiulus	gi|391331488
Metaseiulus	gi|391331490
Metaseiulus	gi|391331828
Metaseiulus	gi|391331839
Metaseiulus	gi|391339863
Metaseiulus	gi|391340672
Metaseiulus	gi|391341243
Metaseiulus	gi|391342181
Metaseiulus	gi|391342183
Metaseiulus	gi|391345696
Metaseiulus	gi|391345845
Metaseiulus	gi|391347554
Tetranychus	tetur01g02240.1
Tetranychus	tetur01g02260.1
Tetranychus	tetur02g03240.1

Taxon	GeneID
Tetranychus	tetur05g01350.1
Tetranychus	tetur08g03770.1
Tetranychus	tetur08g04280.1
Tetranychus	tetur08g04290.1
Tetranychus	tetur10g04760.1
Tetranychus	tetur10g04770.1
Tetranychus	tetur10g04780.1
Tetranychus	tetur10g04790.1
Tetranychus	tetur10g04810.1
Tetranychus	tetur10g04820.1
Tetranychus	tetur128g00010.1
Tetranychus	tetur128g00020.1
Tetranychus	tetur128g00030.1
Tetranychus	tetur128g00050.1
Tetranychus	tetur128g00060.1
Tetranychus	tetur18g03300.1
Tetranychus	tetur20g01980.1
Tetranychus	tetur30g02180.1
Tetranychus	tetur35g00950.1
Tetranychus	tetur57g00050.1
Ixodes	ISCW000427
Ixodes	ISCW004156
Ixodes	ISCW004225
Ixodes	ISCW005088
Ixodes	ISCW006061
Ixodes	ISCW006062
Ixodes	ISCW009616
Ixodes	ISCW010066
Ixodes	ISCW010422
Ixodes	ISCW011013
Ixodes	ISCW011016
Ixodes	ISCW011017

Taxon	GeneID
Ixodes	ISCW013840
Ixodes	ISCW014100
Ixodes	ISCW014257
Ixodes	ISCW014651
Ixodes	ISCW014652
Ixodes	ISCW014779
Ixodes	ISCW015198
Ixodes	ISCW015204
Ixodes	ISCW015349
Ixodes	ISCW016489
Ixodes	ISCW018607
Ixodes	ISCW020581
Ixodes	ISCW021415
Ixodes	ISCW021416
Ixodes	ISCW021417
Ixodes	ISCW023617
Ixodes	ISCW023618
Ixodes	ISCW023619
Ixodes	ISCW023620
Ixodes	ISCW023621
Ixodes	ISCW023622
Ixodes	ISCW023623
Ixodes	ISCW023624
Ixodes	ISCW024013
Ixodes	ISCW024046
Ixodes	ISCW024109
Ixodes	ISCW024342
Ixodes	ISCW024387
Ixodes	ISCW024435
Ixodes	ISCW024495
Ixodes	ISCW024500
Ixodes	ISCW024738

Taxon	GeneID
Mesobuthus	MMa00531
Mesobuthus	MMa02864
Mesobuthus	MMa05993
Mesobuthus	MMa12086
Mesobuthus	MMa12087
Mesobuthus	MMa12745
Mesobuthus	MMa18919
Mesobuthus	MMa18921
Mesobuthus	MMa22061
Mesobuthus	MMa25988
Mesobuthus	MMa26099
Mesobuthus	MMa31248
Mesobuthus	MMa32542
Mesobuthus	MMa32544
Mesobuthus	MMa32545
Mesobuthus	MMa32547
Mesobuthus	MMa33063
Mesobuthus	MMa34746
Mesobuthus	MMa34747
Mesobuthus	MMa34748
Mesobuthus	MMa39750
Mesobuthus	MMa40492
Mesobuthus	MMa43935
Mesobuthus	MMa46416
Mesobuthus	MMa49367
Mesobuthus	MMa52968
Mesobuthus	MMa52970
Mesobuthus	MMa52971
Mesobuthus	MMa52997
Mesobuthus	MMa53423
Parasteatoda	aug3.g1060.t1
Parasteatoda	aug3.g11037.t1

Taxon	GeneID
Parasteatoda	aug3.g13559.t1
Parasteatoda	aug3.g13560.t1
Parasteatoda	aug3.g18534.t1
Parasteatoda	aug3.g18605.t1
Parasteatoda	aug3.g18606.t1
Parasteatoda	aug3.g18607.t1
Parasteatoda	aug3.g18608.t1
Parasteatoda	aug3.g18609.t1
Parasteatoda	aug3.g20374.t1
Parasteatoda	aug3.g20412.t1
Parasteatoda	aug3.g20413.t1
Parasteatoda	aug3.g20414.t1
Parasteatoda	aug3.g20807.t1
Parasteatoda	aug3.g20808.t1
Parasteatoda	aug3.g22979.t1
Parasteatoda	aug3.g23172.t1
Parasteatoda	aug3.g24368.t1
Parasteatoda	aug3.g24654.t1
Parasteatoda	aug3.g26180.t1
Parasteatoda	aug3.g26893.t1
Parasteatoda	aug3.g26894.t1
Parasteatoda	aug3.g26895.t1
Parasteatoda	aug3.g26896.t1
Parasteatoda	aug3.g26897.t1
Parasteatoda	aug3.g26898.t1
Parasteatoda	aug3.g6709.t1
Parasteatoda	aug3.g9433.t1
Parasteatoda	aug3.g9434.t1
Parasteatoda	aug3.g9435.t1
Parasteatoda	aug3.g9436.t1
Parasteatoda	aug3.g9437.t1

Supplementary table 7 – Coordinates of leucine rich repeat regions (LRRs) and cysteine rich regions (LRRCTs) within TLRs as predicted by LRR finder (Offord et al, 2010).

Clade	GeneID	Start	Stop	Length	E-value	Feature
**Drosophila 1-8 Like**	DappuP190084	10	33	24	0	LRR
**mccTLR clade**	DappuP190084	34	57	24	0	LRR
	DappuP190084	58	81	24	0.01	LRR
	DappuP190084	82	105	24	0	LRR
	DappuP190084	106	460	355	0	Potential LRRCT
	DappuP190084					
	DappuP97929	171	194	24	0.01	LRR
	DappuP97929	195	218	24	0	LRR
	DappuP97929	219	242	24	0	LRR
	DappuP97929	243	266	24	0.02	LRR
	DappuP97929	267	290	24	0	LRR
	DappuP97929	291	314	24	0	LRR
	DappuP97929	315	339	25	0.02	LRR
	DappuP97929	340	365	26	0	LRR
	DappuP97929	366	389	24	0	LRR
	DappuP97929	390	413	24	0	LRR
	DappuP97929	414	437	24	0	LRR
	DappuP97929	438	461	24	0	LRR
	DappuP97929	462	494	33	0	LRR
	DappuP97929	495	517	23	0	LRR
	DappuP97929	518	543	26	0	LRR
	DappuP97929	544	711	168	0.04	Potential LRRCT
	DappuP97929	712	743	32	0	LRR
	DappuP97929	744	1100	357	0	Potential LRRCT
	DappuP97929					
	DappuP128614	108	161	54	0.12	InLRR
	DappuP128614	162	185	24	0	LRR
	DappuP128614	186	214	29	0	LRR
	DappuP128614	215	238	24	0	LRR
	DappuP128614	239	262	24	0	LRR

Clade	GeneID	Start	Stop	Length	E-value	Feature
	DappuP128614	263	286	24	0	LRR
	DappuP128614	287	310	24	0	LRR
	DappuP128614	311	334	24	0	LRR
	DappuP128614	335	358	24	0	LRR
	DappuP128614	359	382	24	0	LRR
	DappuP128614	383	406	24	0	LRR
	DappuP128614	407	430	24	0.02	LRR
	DappuP128614	431	453	23	0	LRR
	DappuP128614	454	476	23	0	LRR
	DappuP128614	477	500	24	0	LRR
	DappuP128614	501	524	24	0	LRR
	DappuP128614	525	547	23	0	LRR
	DappuP128614	548	569	22	0	LRR
	DappuP128614	570	593	24	0.01	LRR
	DappuP128614	594	615	22	0.01	LRR
	DappuP128614	616	639	24	0.03	LRR
	DappuP128614	640	803	164	0.01	Potential LRRCT
	DappuP128614	804	827	24	0.01	LRR
	DappuP128614	828	851	24	0	LRR
	DappuP128614	852	875	24	0	LRR
	DappuP128614	876	899	24	0	LRR
	DappuP128614	900	1339	440	0	Potential LRRCT
	DappuP128614					
	DappuP65779	135	188	54	0.02	LRR
	DappuP65779	189	212	24	0	LRR
	DappuP65779	213	241	29	0	LRR
	DappuP65779	242	265	24	0	LRR
	DappuP65779	266	289	24	0	LRR
	DappuP65779	290	314	25	0.01	LRR
	DappuP65779	315	338	24	0.01	LRR
	DappuP65779	339	364	26	0	LRR
	DappuP65779	365	388	24	0	LRR

Clade	GeneID	Start	Stop	Length	E-value	Feature
	DappuP65779	389	412	24	0	LRR
	DappuP65779	413	436	24	0	LRR
	DappuP65779	437	460	24	0.02	LRR
	DappuP65779	461	483	23	0	LRR
	DappuP65779	484	507	24	0	LRR
	DappuP65779	508	531	24	0.02	LRR
	DappuP65779	532	578	47	0	LRR
	DappuP65779	579	600	22	0	LRR
	DappuP65779	601	625	25	0	LRR
	DappuP65779	626	647	22	0	LRR
	DappuP65779	648	671	24	0	LRR
	DappuP65779	672	700	29	0	LRR
	DappuP65779	701	700	0	0.06	Potential LRRCT
	DappuP65779	827	874	48	0.08	InLRR
	DappuP65779	875	898	24	0	LRR
	DappuP65779	899	922	24	0	LRR
	DappuP65779	923	947	25	0	LRR
	DappuP65779	948	1312	365	0.1	Potential LRRCT
	DappuP65779					
	SMAR012279-PA	188	210	23	0.03	LRR
	SMAR012279-PA	211	234	24	0.09	InLRR
	SMAR012279-PA	235	258	24	0	LRR
	SMAR012279-PA	259	282	24	0	LRR
	SMAR012279-PA	283	306	24	0.08	InLRR
	SMAR012279-PA	307	330	24	0.03	LRR
	SMAR012279-PA	331	354	24	0	LRR
	SMAR012279-PA	355	476	122	0	Potential LRRCT
	SMAR012279-PA	477	504	28	0	LRR
	SMAR012279-PA	505	611	107	0	Potential LRRCT
	SMAR012279-PA	612	635	24	0	LRR
	SMAR012279-PA	636	657	22	0	LRR
	SMAR012279-PA	658	680	23	0	LRR

Clade	GeneID	Start	Stop	Length	E-value	Feature
	SMAR012279-PA	681	949	269	0.17	Potential LRRCT
	SMAR012279-PA					
	SMAR011407-PA	162	185	24	0	LRR
	SMAR011407-PA	186	209	24	0	LRR
	SMAR011407-PA	210	232	23	0.03	LRR
	SMAR011407-PA	233	256	24	0	LRR
	SMAR011407-PA	257	280	24	0	LRR
	SMAR011407-PA	281	304	24	0	LRR
	SMAR011407-PA	305	328	24	0.01	LRR
	SMAR011407-PA	329	352	24	0	LRR
	SMAR011407-PA	353	376	24	0.02	LRR
	SMAR011407-PA	377	400	24	0	LRR
	SMAR011407-PA	401	424	24	0	LRR
	SMAR011407-PA	425	449	25	0	LRR
	SMAR011407-PA	450	473	24	0.01	LRR
	SMAR011407-PA	474	494	21	0	LRR
	SMAR011407-PA	495	518	24	0	LRR
	SMAR011407-PA	519	675	157	0	Potential LRRCT
	SMAR011407-PA	676	699	24	0	LRR
	SMAR011407-PA	700	721	22	0	LRR
	SMAR011407-PA	722	1034	313	0	Potential LRRCT
	SMAR011407-PA					
	SMAR011269-PA	182	209	28	0.01	LRR
	SMAR011269-PA	210	232	23	0	LRR
	SMAR011269-PA	233	256	24	0.02	LRR
	SMAR011269-PA	257	280	24	0	LRR
	SMAR011269-PA	281	304	24	0	LRR
	SMAR011269-PA	305	330	26	0.02	LRR
	SMAR011269-PA	331	354	24	0.01	LRR
	SMAR011269-PA	355	394	40	0.01	LRR
	SMAR011269-PA	395	416	22	0	LRR
	SMAR011269-PA	417	440	24	0.16	InLRR

Clade	GeneID	Start	Stop	Length	E-value	Feature
	SMAR011269-PA	441	464	24	0	LRR
	SMAR011269-PA	465	488	24	0.19	InLRR
	SMAR011269-PA	489	531	43	0	LRR
	SMAR011269-PA	532	578	47	0.04	LRR
	SMAR011269-PA	579	602	24	0.11	InLRR
	SMAR011269-PA	603	779	177	0	Potential LRRCT
	SMAR011269-PA	780	803	24	0	LRR
	SMAR011269-PA	804	827	24	0	LRR
	SMAR011269-PA	828	850	23	0	LRR
	SMAR011269-PA	851	1099	249	0.14	Potential LRRCT
	SMAR011269-PA					
	SMAR010912-PA	188	233	46	0	LRR
	SMAR010912-PA	234	257	24	0	LRR
	SMAR010912-PA	258	281	24	0	LRR
	SMAR010912-PA	282	305	24	0.13	InLRR
	SMAR010912-PA	306	328	23	0	LRR
	SMAR010912-PA	329	352	24	0	LRR
	SMAR010912-PA	353	399	47	0.02	LRR
	SMAR010912-PA	400	446	47	0	LRR
	SMAR010912-PA	447	603	157	0	Potential LRRCT
	SMAR010912-PA	604	627	24	0.01	LRR
	SMAR010912-PA	628	649	22	0	LRR
	SMAR010912-PA	650	672	23	0	LRR
	SMAR010912-PA	673	940	268	0.03	Potential LRRCT
	SMAR010912-PA					
	SMAR014647-PA	159	182	24	0	LRR
	SMAR014647-PA	183	206	24	0	LRR
	SMAR014647-PA	207	230	24	0	LRR
	SMAR014647-PA	231	254	24	0.01	LRR
	SMAR014647-PA	255	277	23	0	LRR
	SMAR014647-PA	278	296	19	0	LRR
	SMAR014647-PA	297	320	24	0	LRR

Clade	GeneID	Start	Stop	Length	E-value	Feature
	SMAR014647-PA	321	366	46	0.04	LRR
	SMAR014647-PA	367	554	188	0.01	Potential LRRCT
	SMAR014647-PA	555	576	22	0	LRR
	SMAR014647-PA	577	872	296	0.01	Potential LRRCT
	SMAR014647-PA					
	SMAR008553-PA	131	154	24	0.13	lnLRR
	SMAR008553-PA	155	178	24	0	LRR
	SMAR008553-PA	179	202	24	0	LRR
	SMAR008553-PA	203	226	24	0	LRR
	SMAR008553-PA	227	250	24	0	LRR
	SMAR008553-PA	251	274	24	0.01	LRR
	SMAR008553-PA	275	298	24	0	LRR
	SMAR008553-PA	299	322	24	0	LRR
	SMAR008553-PA	323	344	22	0	LRR
	SMAR008553-PA	345	364	20	0	LRR
	SMAR008553-PA	365	387	23	0.02	LRR
	SMAR008553-PA	388	409	22	0.02	LRR
	SMAR008553-PA	410	453	44	0	LRR
	SMAR008553-PA	454	477	24	0	LRR
	SMAR008553-PA	478	501	24	0	LRR
	SMAR008553-PA	502	525	24	0	LRR
	SMAR008553-PA	526	684	159	0.14	Potential LRRCT
	SMAR008553-PA	685	708	24	0	LRR
	SMAR008553-PA	709	732	24	0	LRR
	SMAR008553-PA	733	754	22	0	LRR
	SMAR008553-PA	755	778	24	0	LRR
	SMAR008553-PA	779	805	27	0	LRR
	SMAR008553-PA	806	855	50	0	LRR
	SMAR008553-PA	856	1111	256	0.11	Potential LRRCT
	SMAR008553-PA	1112	1133	22	0.01	LRR
	SMAR008553-PA	1134	1154	21	0	LRR
	SMAR008553-PA	1155	1491	337	0.14	Potential LRRCT

Clade	GeneID	Start	Stop	Length	E-value	Feature
	SMAR008553-PA					
	SMAR008376-PA	110	163	54	0.01	LRR
	SMAR008376-PA	164	187	24	0	LRR
	SMAR008376-PA	188	218	31	0	LRR
	SMAR008376-PA	219	242	24	0	LRR
	SMAR008376-PA	243	266	24	0	LRR
	SMAR008376-PA	267	290	24	0	LRR
	SMAR008376-PA	291	314	24	0	LRR
	SMAR008376-PA	315	340	26	0	LRR
	SMAR008376-PA	341	364	24	0	LRR
	SMAR008376-PA	365	388	24	0	LRR
	SMAR008376-PA	389	412	24	0	LRR
	SMAR008376-PA	413	436	24	0	LRR
	SMAR008376-PA	437	459	23	0.01	LRR
	SMAR008376-PA	460	483	24	0	LRR
	SMAR008376-PA	484	507	24	0	LRR
	SMAR008376-PA	508	531	24	0	LRR
	SMAR008376-PA	532	554	23	0.05	InLRR
	SMAR008376-PA	555	576	22	0	LRR
	SMAR008376-PA	577	601	25	0	LRR
	SMAR008376-PA	602	623	22	0	LRR
	SMAR008376-PA	624	647	24	0.03	LRR
	SMAR008376-PA	648	676	29	0.01	LRR
	SMAR008376-PA	677	700	24	0.02	Potential LRRCT
	SMAR008376-PA	801	824	24	0.02	LRR
	SMAR008376-PA	825	848	24	0.01	LRR
	SMAR008376-PA	849	872	24	0	LRR
	SMAR008376-PA	873	896	24	0	LRR
	SMAR008376-PA	897	920	24	0	LRR
	SMAR008376-PA	921	1188	268	0.09	Potential LRRCT
	SMAR008376-PA					
	SMAR006691-PA	84	133	50	0.08	InLRR

Clade	GeneID	Start	Stop	Length	E-value	Feature
	SMAR006691-PA	134	157	24	0	LRR
	SMAR006691-PA	158	181	24	0	LRR
	SMAR006691-PA	182	205	24	0	LRR
	SMAR006691-PA	206	227	22	0	LRR
	SMAR006691-PA	228	251	24	0	LRR
	SMAR006691-PA	252	296	45	0	LRR
	SMAR006691-PA	297	339	43	0.03	LRR
	SMAR006691-PA	340	412	73	0	LRR
	SMAR006691-PA	413	567	155	0	Potential LRRCT
	SMAR006691-PA	568	591	24	0.01	LRR
	SMAR006691-PA	592	615	24	0	LRR
	SMAR006691-PA	616	903	288	0	Potential LRRCT
	SMAR006691-PA					
	SMAR005841-PA	181	203	23	0.1	lnLRR
	SMAR005841-PA	204	251	48	0	LRR
	SMAR005841-PA	252	275	24	0	LRR
	SMAR005841-PA	276	299	24	0	LRR
	SMAR005841-PA	300	323	24	0.01	LRR
	SMAR005841-PA	324	345	22	0.01	LRR
	SMAR005841-PA	346	370	25	0.08	lnLRR
	SMAR005841-PA	371	542	172	0.01	Potential LRRCT
	SMAR005841-PA	543	574	32	0.06	lnLRR
	SMAR005841-PA	575	598	24	0	LRR
	SMAR005841-PA	599	622	24	0	LRR
	SMAR005841-PA	623	646	24	0	LRR
	SMAR005841-PA	647	669	23	0	LRR
	SMAR005841-PA	670	691	22	0	LRR
	SMAR005841-PA	692	713	22	0	LRR
	SMAR005841-PA	714	736	23	0	LRR
	SMAR005841-PA	737	966	230	0.01	Potential LRRCT
	SMAR005841-PA	967	990	24	0.14	lnLRR
	SMAR005841-PA	991	1014	24	0	LRR

Clade	GeneID	Start	Stop	Length	E-value	Feature
	SMAR005841-PA	1015	1038	24	0	LRR
	SMAR005841-PA	1039	1062	24	0.07	InLRR
	SMAR005841-PA	1063	1086	24	0	LRR
	SMAR005841-PA	1087	1110	24	0	LRR
	SMAR005841-PA	1111	1133	23	0	LRR
	SMAR005841-PA	1134	1311	178	0	Potential LRRCT
	SMAR005841-PA	1312	1361	50	0.13	InLRR
	SMAR005841-PA	1362	1383	22	0	LRR
	SMAR005841-PA	1384	1420	37	0	LRR
	SMAR005841-PA	1421	1442	22	0	LRR
	SMAR005841-PA	1443	1753	311	0	Potential LRRCT
	SMAR005841-PA					
	SMAR014575-PA	180	207	28	0.01	LRR
	SMAR014575-PA	208	230	23	0	LRR
	SMAR014575-PA	231	254	24	0.03	LRR
	SMAR014575-PA	255	278	24	0.05	InLRR
	SMAR014575-PA	279	302	24	0	LRR
	SMAR014575-PA	303	330	28	0.02	LRR
	SMAR014575-PA	331	354	24	0	LRR
	SMAR014575-PA	355	378	24	0	LRR
	SMAR014575-PA	379	402	24	0	LRR
	SMAR014575-PA	403	424	22	0	LRR
	SMAR014575-PA	425	449	25	0	LRR
	SMAR014575-PA	450	473	24	0	LRR
	SMAR014575-PA	474	497	24	0.01	LRR
	SMAR014575-PA	498	540	43	0.01	LRR
	SMAR014575-PA	541	611	71	0.1	InLRR
	SMAR014575-PA	612	773	162	0	Potential LRRCT
	SMAR014575-PA	774	797	24	0.01	LRR
	SMAR014575-PA	798	821	24	0	LRR
	SMAR014575-PA	822	845	24	0	LRR
	SMAR014575-PA	846	868	23	0	LRR

Clade	GeneID	Start	Stop	Length	E-value	Feature
	SMAR014575-PA	869	1130	262	0	Potential LRRCT
	SMAR014575-PA					
	SMAR005346-PA	127	176	50	0.05	lnLRR
	SMAR005346-PA	177	226	50	0	LRR
	SMAR005346-PA	227	250	24	0	LRR
	SMAR005346-PA	251	274	24	0	LRR
	SMAR005346-PA	275	298	24	0	LRR
	SMAR005346-PA	299	324	26	0	LRR
	SMAR005346-PA	325	348	24	0	LRR
	SMAR005346-PA	349	372	24	0.19	lnLRR
	SMAR005346-PA	373	396	24	0.01	LRR
	SMAR005346-PA	397	418	22	0	LRR
	SMAR005346-PA	419	442	24	0	LRR
	SMAR005346-PA	443	466	24	0	LRR
	SMAR005346-PA	467	490	24	0	LRR
	SMAR005346-PA	491	533	43	0	LRR
	SMAR005346-PA	534	557	24	0	LRR
	SMAR005346-PA	558	634	77	0.17	lnLRR
	SMAR005346-PA	635	685	51	0.06	Potential LRRCT
	SMAR005346-PA	686	761	76	0.03	Potential LRRCT
	SMAR005346-PA	762	785	24	0.01	LRR
	SMAR005346-PA	786	809	24	0	LRR
	SMAR005346-PA	810	1090	281	0	Potential LRRCT
	SMAR005346-PA					
	SMAR005007-PA	108	185	78	0.01	LRR
	SMAR005007-PA	186	213	28	0.08	lnLRR
	SMAR005007-PA	214	236	23	0	LRR
	SMAR005007-PA	237	260	24	0	LRR
	SMAR005007-PA	261	284	24	0.01	LRR
	SMAR005007-PA	285	334	50	0	LRR
	SMAR005007-PA	335	358	24	0	LRR
	SMAR005007-PA	359	382	24	0	LRR

Clade	GeneID	Start	Stop	Length	E-value	Feature
	SMAR005007-PA	383	427	45	0	LRR
	SMAR005007-PA	428	473	46	0	LRR
	SMAR005007-PA	474	521	48	0	LRR
	SMAR005007-PA	522	564	43	0.12	InLRR
	SMAR005007-PA	565	635	71	0	LRR
	SMAR005007-PA	636	821	186	0	Potential LRRCT
	SMAR005007-PA	822	845	24	0	LRR
	SMAR005007-PA	846	869	24	0	LRR
	SMAR005007-PA	870	1156	287	0	Potential LRRCT
	SMAR005007-PA					
	SMAR005008-PA	196	219	24	0	LRR
	SMAR005008-PA	220	243	24	0.01	LRR
	SMAR005008-PA	244	267	24	0	LRR
	SMAR005008-PA	268	289	22	0	LRR
	SMAR005008-PA	290	313	24	0.05	InLRR
	SMAR005008-PA	314	379	66	0	LRR
	SMAR005008-PA	380	401	22	0.02	LRR
	SMAR005008-PA	402	426	25	0	LRR
	SMAR005008-PA	427	472	46	0.1	InLRR
	SMAR005008-PA	473	626	154	0	Potential LRRCT
	SMAR005008-PA	627	650	24	0	LRR
	SMAR005008-PA	651	674	24	0	LRR
	SMAR005008-PA	675	958	284	0	Potential LRRCT
	SMAR005008-PA					
	SMAR005010-PA	171	190	20	0	LRR
	SMAR005010-PA	191	216	26	0	LRR
	SMAR005010-PA	217	240	24	0.03	LRR
	SMAR005010-PA	241	264	24	0	LRR
	SMAR005010-PA	265	288	24	0	LRR
	SMAR005010-PA	289	312	24	0.01	LRR
	SMAR005010-PA	313	335	23	0.01	LRR
	SMAR005010-PA	336	359	24	0.01	LRR

Clade	GeneID	Start	Stop	Length	E-value	Feature
	SMAR005010-PA	360	406	47	0	LRR
	SMAR005010-PA	407	611	205	0.01	Potential LRRCT
	SMAR005010-PA	612	635	24	0	LRR
	SMAR005010-PA	636	657	22	0	LRR
	SMAR005010-PA	658	680	23	0	LRR
	SMAR005010-PA	681	947	267	0.01	Potential LRRCT
	SMAR005010-PA					
	SMAR004813-PA	95	118	24	0	LRR
	SMAR004813-PA	119	142	24	0.01	LRR
	SMAR004813-PA	143	194	52	0	LRR
	SMAR004813-PA	195	218	24	0	LRR
	SMAR004813-PA	219	244	26	0	LRR
	SMAR004813-PA	245	270	26	0	LRR
	SMAR004813-PA	271	292	22	0	LRR
	SMAR004813-PA	293	317	25	0.02	LRR
	SMAR004813-PA	318	349	32	0	LRR
	SMAR004813-PA	350	374	25	0	LRR
	SMAR004813-PA	375	398	24	0	Potential LRRCT
	SMAR004813-PA	399	420	22	0	LRR
	SMAR004813-PA	421	444	24	0	LRR
	SMAR004813-PA	445	716	272	0	Potential LRRCT
	SMAR004813-PA					
	SMAR004578-PA	161	235	75	0.02	LRR
	SMAR004578-PA	236	263	28	0	LRR
	SMAR004578-PA	264	298	35	0	LRR
	SMAR004578-PA	299	322	24	0	LRR
	SMAR004578-PA	323	346	24	0	LRR
	SMAR004578-PA	347	371	25	0	LRR
	SMAR004578-PA	372	419	48	0	LRR
	SMAR004578-PA	420	462	43	0	LRR
	SMAR004578-PA	463	487	25	0.01	LRR
	SMAR004578-PA	488	509	22	0.06	lnLRR

Clade	GeneID	Start	Stop	Length	E-value	Feature
	SMAR004578-PA	510	533	24	0.06	InLRR
	SMAR004578-PA	534	718	185	0	Potential LRRCT
	SMAR004578-PA	719	742	24	0	LRR
	SMAR004578-PA	743	766	24	0	LRR
	SMAR004578-PA	767	1052	286	0	Potential LRRCT
	SMAR004578-PA					
	SMAR003805-PA	184	211	28	0	LRR
	SMAR003805-PA	212	234	23	0.06	InLRR
	SMAR003805-PA	235	258	24	0	LRR
	SMAR003805-PA	259	282	24	0	LRR
	SMAR003805-PA	283	306	24	0	LRR
	SMAR003805-PA	307	332	26	0.02	LRR
	SMAR003805-PA	333	356	24	0	LRR
	SMAR003805-PA	357	380	24	0	LRR
	SMAR003805-PA	381	412	32	0	LRR
	SMAR003805-PA	413	460	48	0	LRR
	SMAR003805-PA	461	503	43	0.09	InLRR
	SMAR003805-PA	504	574	71	0.02	LRR
	SMAR003805-PA	575	735	161	0	Potential LRRCT
	SMAR003805-PA	736	759	24	0.05	InLRR
	SMAR003805-PA	760	783	24	0	LRR
	SMAR003805-PA	784	807	24	0	LRR
	SMAR003805-PA	808	830	23	0	LRR
	SMAR003805-PA	831	1093	263	0.15	Potential LRRCT
	SMAR003805-PA					
	SMAR015400-PA	155	178	24	0	LRR
	SMAR015400-PA	179	221	43	0	LRR
	SMAR015400-PA	222	324	103	0.02	Potential LRRCT
	SMAR015400-PA	325	348	24	0	LRR
	SMAR015400-PA	349	370	22	0	LRR
	SMAR015400-PA	371	603	233	0.02	Potential LRRCT
	SMAR015400-PA					

Clade	GeneID	Start	Stop	Length	E-value	Feature
	SMAR002710-PA	49	119	71	0.06	InLRR
	SMAR002710-PA	120	296	177	0	Potential LRRCT
	SMAR002710-PA	297	320	24	0	LRR
	SMAR002710-PA	321	344	24	0	LRR
	SMAR002710-PA	345	537	193	0	LRR
	SMAR002710-PA					
	SMAR002611-PA	180	202	23	0	LRR
	SMAR002611-PA	203	226	24	0.04	LRR
	SMAR002611-PA	227	250	24	0.01	LRR
	SMAR002611-PA	251	274	24	0	LRR
	SMAR002611-PA	275	297	23	0	LRR
	SMAR002611-PA	298	478	181	0	Potential LRRCT
	SMAR002611-PA	479	502	24	0.06	InLRR
	SMAR002611-PA	503	524	22	0	LRR
	SMAR002611-PA	525	854	330	0	Potential LRRCT
	SMAR002611-PA					
	SMAR002366-PA	138	293	156	0.12	Potential LRRCT
	SMAR002366-PA	294	319	26	0	LRR
	SMAR002366-PA	320	341	22	0	LRR
	SMAR002366-PA	342	365	24	0	LRR
	SMAR002366-PA	366	631	266	0	Potential LRRCT
	SMAR002366-PA					
	SMAR002249-PA	26	49	24	0	LRR
	SMAR002249-PA	50	72	23	0	LRR
	SMAR002249-PA	73	336	264	0.01	Potential LRRCT
	SMAR002249-PA					
	SMAR002076-PA	24	47	24	0	LRR
	SMAR002076-PA	48	79	32	0	LRR
	SMAR002076-PA	80	200	121	0	Potential LRRCT
	SMAR002076-PA	201	224	24	0	LRR
	SMAR002076-PA	225	271	47	0	LRR
	SMAR002076-PA	272	534	263	0.12	Potential LRRCT

Clade	GeneID	Start	Stop	Length	E-value	Feature
	SMAR002076-PA					
	SMAR001938-PA	91	114	24	0.02	LRR
	SMAR001938-PA	115	138	24	0	LRR
	SMAR001938-PA	139	162	24	0	LRR
	SMAR001938-PA	163	184	22	0	LRR
	SMAR001938-PA	185	425	241	0.03	Potential LRRCT
	SMAR001938-PA					
	SMAR001266-PA	180	202	23	0	LRR
	SMAR001266-PA	203	226	24	0.01	LRR
	SMAR001266-PA	227	250	24	0	LRR
	SMAR001266-PA	251	274	24	0	LRR
	SMAR001266-PA	275	298	24	0	LRR
	SMAR001266-PA	299	321	23	0	LRR
	SMAR001266-PA	322	345	24	0	LRR
	SMAR001266-PA	346	468	123	0	Potential LRRCT
	SMAR001266-PA	469	494	26	0	LRR
	SMAR001266-PA	495	600	106	0.08	Potential LRRCT
	SMAR001266-PA	601	624	24	0	LRR
	SMAR001266-PA	625	646	22	0	LRR
	SMAR001266-PA	647	669	23	0	LRR
	SMAR001266-PA	670	952	283	0.01	Potential LRRCT
	SMAR001266-PA					
	SMAR000970-PA	180	202	23	0	LRR
	SMAR000970-PA	203	226	24	0.08	InLRR
	SMAR000970-PA	227	250	24	0	LRR
	SMAR000970-PA	251	280	30	0	LRR
	SMAR000970-PA	281	333	53	0	LRR
	SMAR000970-PA	334	440	107	0.09	Potential LRRCT
	SMAR000970-PA	441	465	25	0.1	InLRR
	SMAR000970-PA	466	488	23	0	LRR
	SMAR000970-PA	489	511	23	0	LRR
	SMAR000970-PA	512	784	273	0	Potential LRRCT

Clade	GeneID	Start	Stop	Length	E-value	Feature
	SMAR000970-PA					
	gi_PIPE_391327659_PIPE_ref_PIPE_XP_003738314.1_PIPE_	122	175	54	0.02	LRR
	gi_PIPE_391327659_PIPE_ref_PIPE_XP_003738314.1_PIPE_	176	199	24	0	LRR
	gi_PIPE_391327659_PIPE_ref_PIPE_XP_003738314.1_PIPE_	200	226	27	0	LRR
	gi_PIPE_391327659_PIPE_ref_PIPE_XP_003738314.1_PIPE_	227	250	24	0	LRR
	gi_PIPE_391327659_PIPE_ref_PIPE_XP_003738314.1_PIPE_	251	274	24	0	LRR
	gi_PIPE_391327659_PIPE_ref_PIPE_XP_003738314.1_PIPE_	275	298	24	0	LRR
	gi_PIPE_391327659_PIPE_ref_PIPE_XP_003738314.1_PIPE_	299	322	24	0	LRR
	gi_PIPE_391327659_PIPE_ref_PIPE_XP_003738314.1_PIPE_	323	348	26	0	LRR
	gi_PIPE_391327659_PIPE_ref_PIPE_XP_003738314.1_PIPE_	349	372	24	0	LRR
	gi_PIPE_391327659_PIPE_ref_PIPE_XP_003738314.1_PIPE_	373	396	24	0	LRR
	gi_PIPE_391327659_PIPE_ref_PIPE_XP_003738314.1_PIPE_	397	444	48	0	LRR
	gi_PIPE_391327659_PIPE_ref_PIPE_XP_003738314.1_PIPE_	445	467	23	0	LRR
	gi_PIPE_391327659_PIPE_ref_PIPE_XP_003738314.1_PIPE_	468	491	24	0	LRR
	gi_PIPE_391327659_PIPE_ref_PIPE_XP_003738314.1_PIPE_	492	515	24	0	LRR
	gi_PIPE_391327659_PIPE_ref_PIPE_XP_003738314.1_PIPE_	516	562	47	0	LRR
	gi_PIPE_391327659_PIPE_ref_PIPE_XP_003738314.1_PIPE_	563	584	22	0	LRR
	gi_PIPE_391327659_PIPE_ref_PIPE_XP_003738314.1_PIPE_	585	609	25	0	LRR
	gi_PIPE_391327659_PIPE_ref_PIPE_XP_003738314.1_PIPE_	610	631	22	0	LRR
	gi_PIPE_391327659_PIPE_ref_PIPE_XP_003738314.1_PIPE_	632	655	24	0.12	InLRR
	gi_PIPE_391327659_PIPE_ref_PIPE_XP_003738314.1_PIPE_	656	808	153	0.01	Potential LRRCT
	gi_PIPE_391327659_PIPE_ref_PIPE_XP_003738314.1_PIPE_	809	856	48	0	LRR
	gi_PIPE_391327659_PIPE_ref_PIPE_XP_003738314.1_PIPE_	857	880	24	0	LRR
	gi_PIPE_391327659_PIPE_ref_PIPE_XP_003738314.1_PIPE_	881	904	24	0	LRR
	gi_PIPE_391327659_PIPE_ref_PIPE_XP_003738314.1_PIPE_	905	928	24	0	LRR
	gi_PIPE_391327659_PIPE_ref_PIPE_XP_003738314.1_PIPE_	929	1250	322	0	Potential LRRCT
	gi_PIPE_391327659_PIPE_ref_PIPE_XP_003738314.1_PIPE_					
	gi_PIPE_391335441_PIPE_ref_PIPE_XP_003742102.1_PIPE_	123	176	54	0.08	InLRR
	gi_PIPE_391335441_PIPE_ref_PIPE_XP_003742102.1_PIPE_	177	200	24	0	LRR
	gi_PIPE_391335441_PIPE_ref_PIPE_XP_003742102.1_PIPE_	201	228	28	0.02	LRR
	gi_PIPE_391335441_PIPE_ref_PIPE_XP_003742102.1_PIPE_	229	252	24	0.01	LRR
	gi_PIPE_391335441_PIPE_ref_PIPE_XP_003742102.1_PIPE_	253	276	24	0	LRR

Clade	GeneID	Start	Stop	Length	E-value	Feature
	gi_PIPE_391335441_PIPE_ref_PIPE_XP_003742102.1_PIPE_	277	300	24	0	LRR
	gi_PIPE_391335441_PIPE_ref_PIPE_XP_003742102.1_PIPE_	301	324	24	0	LRR
	gi_PIPE_391335441_PIPE_ref_PIPE_XP_003742102.1_PIPE_	325	350	26	0	LRR
	gi_PIPE_391335441_PIPE_ref_PIPE_XP_003742102.1_PIPE_	351	374	24	0	LRR
	gi_PIPE_391335441_PIPE_ref_PIPE_XP_003742102.1_PIPE_	375	398	24	0	LRR
	gi_PIPE_391335441_PIPE_ref_PIPE_XP_003742102.1_PIPE_	399	422	24	0	LRR
	gi_PIPE_391335441_PIPE_ref_PIPE_XP_003742102.1_PIPE_	423	446	24	0.03	LRR
	gi_PIPE_391335441_PIPE_ref_PIPE_XP_003742102.1_PIPE_	447	469	23	0	LRR
	gi_PIPE_391335441_PIPE_ref_PIPE_XP_003742102.1_PIPE_	470	493	24	0	LRR
	gi_PIPE_391335441_PIPE_ref_PIPE_XP_003742102.1_PIPE_	494	517	24	0	LRR
	gi_PIPE_391335441_PIPE_ref_PIPE_XP_003742102.1_PIPE_	518	541	24	0	LRR
	gi_PIPE_391335441_PIPE_ref_PIPE_XP_003742102.1_PIPE_	542	564	23	0	LRR
	gi_PIPE_391335441_PIPE_ref_PIPE_XP_003742102.1_PIPE_	565	586	22	0.07	InLRR
	gi_PIPE_391335441_PIPE_ref_PIPE_XP_003742102.1_PIPE_	587	611	25	0	LRR
	gi_PIPE_391335441_PIPE_ref_PIPE_XP_003742102.1_PIPE_	612	633	22	0	LRR
	gi_PIPE_391335441_PIPE_ref_PIPE_XP_003742102.1_PIPE_	634	657	24	0.08	InLRR
	gi_PIPE_391335441_PIPE_ref_PIPE_XP_003742102.1_PIPE_	658	864	207	0	Potential LRRCT
	gi_PIPE_391335441_PIPE_ref_PIPE_XP_003742102.1_PIPE_	865	888	24	0	LRR
	gi_PIPE_391335441_PIPE_ref_PIPE_XP_003742102.1_PIPE_	889	912	24	0	LRR
	gi_PIPE_391335441_PIPE_ref_PIPE_XP_003742102.1_PIPE_	913	1219	307	0	Potential LRRCT
	gi_PIPE_391335441_PIPE_ref_PIPE_XP_003742102.1_PIPE_					
	gi_PIPE_391337512_PIPE_ref_PIPE_XP_003743111.1_PIPE_	150	262	113	0.03	LRR
	gi_PIPE_391337512_PIPE_ref_PIPE_XP_003743111.1_PIPE_	263	286	24	0.03	LRR
	gi_PIPE_391337512_PIPE_ref_PIPE_XP_003743111.1_PIPE_	287	310	24	0	LRR
	gi_PIPE_391337512_PIPE_ref_PIPE_XP_003743111.1_PIPE_	311	337	27	0	LRR
	gi_PIPE_391337512_PIPE_ref_PIPE_XP_003743111.1_PIPE_	338	361	24	0	LRR
	gi_PIPE_391337512_PIPE_ref_PIPE_XP_003743111.1_PIPE_	362	507	146	0	Potential LRRCT
	gi_PIPE_391337512_PIPE_ref_PIPE_XP_003743111.1_PIPE_	508	530	23	0.02	LRR
	gi_PIPE_391337512_PIPE_ref_PIPE_XP_003743111.1_PIPE_	531	554	24	0	LRR
	gi_PIPE_391337512_PIPE_ref_PIPE_XP_003743111.1_PIPE_	555	864	310	0	Potential LRRCT
	gi_PIPE_391337512_PIPE_ref_PIPE_XP_003743111.1_PIPE_					
	gi_PIPE_391342046_PIPE_ref_PIPE_XP_003745335.1_PIPE_	219	266	48	0	LRR

Clade	GeneID	Start	Stop	Length	E-value	Feature
	gi_PIPE_391342046_PIPE_ref_PIPE_XP_003745335.1_PIPE_	267	362	96	0	LRR
	gi_PIPE_391342046_PIPE_ref_PIPE_XP_003745335.1_PIPE_	363	386	24	0.02	LRR
	gi_PIPE_391342046_PIPE_ref_PIPE_XP_003745335.1_PIPE_	387	409	23	0	LRR
	gi_PIPE_391342046_PIPE_ref_PIPE_XP_003745335.1_PIPE_	410	433	24	0	LRR
	gi_PIPE_391342046_PIPE_ref_PIPE_XP_003745335.1_PIPE_	434	457	24	0	LRR
	gi_PIPE_391342046_PIPE_ref_PIPE_XP_003745335.1_PIPE_	458	483	26	0	LRR
	gi_PIPE_391342046_PIPE_ref_PIPE_XP_003745335.1_PIPE_	484	507	24	0	LRR
	gi_PIPE_391342046_PIPE_ref_PIPE_XP_003745335.1_PIPE_	508	655	148	0	Potential LRRCT
	gi_PIPE_391342046_PIPE_ref_PIPE_XP_003745335.1_PIPE_	656	678	23	0.02	LRR
	gi_PIPE_391342046_PIPE_ref_PIPE_XP_003745335.1_PIPE_	679	702	24	0	LRR
	gi_PIPE_391342046_PIPE_ref_PIPE_XP_003745335.1_PIPE_	703	1021	319	0	Potential LRRCT
	gi_PIPE_391342046_PIPE_ref_PIPE_XP_003745335.1_PIPE_					
	tetur09g04990.1	125	178	54	0.04	LRR
	tetur09g04990.1	179	202	24	0	LRR
	tetur09g04990.1	203	233	31	0	LRR
	tetur09g04990.1	234	257	24	0	LRR
	tetur09g04990.1	258	281	24	0	LRR
	tetur09g04990.1	282	305	24	0	LRR
	tetur09g04990.1	306	329	24	0	LRR
	tetur09g04990.1	330	355	26	0	LRR
	tetur09g04990.1	356	379	24	0	LRR
	tetur09g04990.1	380	403	24	0	LRR
	tetur09g04990.1	404	427	24	0	LRR
	tetur09g04990.1	428	451	24	0.01	LRR
	tetur09g04990.1	452	474	23	0	LRR
	tetur09g04990.1	475	509	35	0	LRR
	tetur09g04990.1	510	533	24	0	LRR
	tetur09g04990.1	534	558	25	0	LRR
	tetur09g04990.1	559	581	23	0.01	LRR
	tetur09g04990.1	582	603	22	0	LRR
	tetur09g04990.1	604	626	23	0.06	InLRR
	tetur09g04990.1	627	649	23	0.14	InLRR

Clade	GeneID	Start	Stop	Length	E-value	Feature
	tetur09g04990.1	650	673	24	0.03	LRR
	tetur09g04990.1	674	825	152	0	Potential LRRCT
	tetur09g04990.1	826	873	48	0	LRR
	tetur09g04990.1	874	897	24	0	LRR
	tetur09g04990.1	898	921	24	0	LRR
	tetur09g04990.1	922	1277	356	0	Potential LRRCT
	tetur09g04990.1					
	ISCW018193-RA	143	167	25	0.53	LRR
	ISCW018193-RA	168	192	25	0.14	LRR
	ISCW018193-RA	193	216	24	0.09	LRR
	ISCW018193-RA	217	240	24	0.94	LRR
	ISCW018193-RA	241	264	24	0.03	LRR
	ISCW018193-RA	265	288	24	0	LRR
	ISCW018193-RA	289	312	24	0	LRR
	ISCW018193-RA	313	337	25	1.35	LRR
	ISCW018193-RA	338	361	24	0.02	LRR
	ISCW018193-RA	362	385	24	0	LRR
	ISCW018193-RA	386	409	24	0	LRR
	ISCW018193-RA	410	433	24	0.01	LRR
	ISCW018193-RA	434	454	21	0	LRR
	ISCW018193-RA	455	482	28	15,933.75	LRR
	ISCW018193-RA	483	506	24	0	LRR
	ISCW018193-RA	507	528	22	0	Potential LRRCT
	ISCW018193-RA	649	669	21	0.69	LRR
	ISCW018193-RA	670	693	24	0.01	LRR
	ISCW018193-RA	694	715	22	0.05	LRR
	ISCW018193-RA	716	742	27	0	LRR
	ISCW018193-RA	743	766	24	0.05	Potential LRRCT
	ISCW018193-RA					
	ISCW007724-RA	77	106	30	0	LRR
	ISCW007724-RA	107	128	22	0	LRR
	ISCW007724-RA	129	151	23	0.01	LRR

Clade	GeneID	Start	Stop	Length	E-value	Feature
	ISCW007724-RA	152	175	24	0.15	Potential LRRCT
	ISCW007724-RA					
	ISCW022740-RA	262	285	24	0.01	LRR
	ISCW022740-RA	286	315	30	5.5	LRR
	ISCW022740-RA	316	339	24	0	LRR
	ISCW022740-RA	340	366	27	0.06	LRR
	ISCW022740-RA	367	390	24	0	LRR
	ISCW022740-RA	391	414	24	0	LRR
	ISCW022740-RA	415	438	24	0	LRR
	ISCW022740-RA	439	462	24	0	LRR
	ISCW022740-RA	463	488	26	0	LRR
	ISCW022740-RA	489	512	24	0	LRR
	ISCW022740-RA	513	536	24	0	LRR
	ISCW022740-RA	537	560	24	0	LRR
	ISCW022740-RA	561	584	24	0.06	LRR
	ISCW022740-RA	585	607	23	0	LRR
	ISCW022740-RA	608	631	24	0	LRR
	ISCW022740-RA	632	655	24	0	LRR
	ISCW022740-RA	656	679	24	0	LRR
	ISCW022740-RA	680	702	23	0.01	LRR
	ISCW022740-RA	703	724	22	0	LRR
	ISCW022740-RA	725	749	25	0	LRR
	ISCW022740-RA	750	771	22	0	LRR
	ISCW022740-RA	772	795	24	0.01	LRR
	ISCW022740-RA	796	824	29	0	LRR
	ISCW022740-RA	825	848	24	28.91	Potential LRRCT
	ISCW022740-RA	949	972	24	0	LRR
	ISCW022740-RA	973	996	24	0.11	LRR
	ISCW022740-RA	997	1020	24	0	LRR
	ISCW022740-RA	1021	1044	24	0	LRR
	ISCW022740-RA	1045	1068	24	0	LRR
	ISCW022740-RA	1069	1092	24	5.49	Potential LRRCT

Clade	GeneID	Start	Stop	Length	E-value	Feature
	ISCW022740-RA					
	ISCW020989-RA	123	146	24	0.02	LRR
	ISCW020989-RA	147	176	30	26.47	LRR
	ISCW020989-RA	177	200	24	0	LRR
	ISCW020989-RA	201	232	32	0	LRR
	ISCW020989-RA	233	256	24	0	LRR
	ISCW020989-RA	257	280	24	0	LRR
	ISCW020989-RA	281	304	24	0	LRR
	ISCW020989-RA	305	328	24	0	LRR
	ISCW020989-RA	329	354	26	0	LRR
	ISCW020989-RA	355	378	24	0	LRR
	ISCW020989-RA	379	402	24	0	LRR
	ISCW020989-RA	403	426	24	0	LRR
	ISCW020989-RA	427	450	24	0.06	LRR
	ISCW020989-RA	451	473	23	0	LRR
	ISCW020989-RA	474	497	24	0	LRR
	ISCW020989-RA	498	521	24	0	LRR
	ISCW020989-RA	522	545	24	0	LRR
	ISCW020989-RA	546	568	23	1.29	LRR
	ISCW020989-RA	569	590	22	0	LRR
	ISCW020989-RA	591	615	25	0	LRR
	ISCW020989-RA	616	637	22	0	LRR
	ISCW020989-RA	638	661	24	0	LRR
	ISCW020989-RA	662	690	29	0	LRR
	ISCW020989-RA	691	714	24	523.79	Potential LRRCT
	ISCW020989-RA	815	838	24	0	LRR
	ISCW020989-RA	839	862	24	0.02	LRR
	ISCW020989-RA	863	886	24	0	LRR
	ISCW020989-RA	887	910	24	0	LRR
	ISCW020989-RA	911	934	24	0	LRR
	ISCW020989-RA	935	958	24	0.01	Potential LRRCT
	ISCW020989-RA					

Clade	GeneID	Start	Stop	Length	E-value	Feature
	MMa46071	5	28	24	0.01	LRR
	MMa46071	29	52	24	0	LRR
	MMa46071	53	149	97	0	LRR
	MMa46071	150	173	24	0.01	LRR
	MMa46071	174	197	24	0	LRR
	MMa46071	198	221	24	0	LRR
	MMa46071	222	269	48	0	LRR
	MMa46071	270	291	22	0.03	LRR
	MMa46071	292	439	148	0.03	Potential LRRCT
	MMa46071	440	461	22	0.03	LRR
	MMa46071	462	946	485	0.02	Potential LRRCT
	MMa46071	947	970	24	0	LRR
	MMa46071	971	994	24	0.01	LRR
	MMa46071	995	1066	72	0	Potential LRRCT
	MMa46071	1067	1090	24	0	LRR
	MMa46071	1091	1114	24	0.17	InLRR
	MMa46071	1115	1138	24	0	LRR
	MMa46071	1139	1163	25	0	LRR
	MMa46071	1164	1186	23	0	LRR
	MMa46071	1187	1347	161	0.01	Potential LRRCT
	MMa46071	1348	1369	22	0	LRR
	MMa46071	1370	1395	26	0	LRR
	MMa46071	1396	1685	290	0.01	Potential LRRCT
	MMa46071					
	MMa52435	146	200	55	0.04	LRR
	MMa52435	201	224	24	0	LRR
	MMa52435	225	252	28	0	LRR
	MMa52435	253	276	24	0	LRR
	MMa52435	277	300	24	0	LRR
	MMa52435	301	324	24	0	LRR
	MMa52435	325	348	24	0	LRR
	MMa52435	349	374	26	0	LRR

Clade	GeneID	Start	Stop	Length	E-value	Feature
	MMa52435	375	398	24	0	LRR
	MMa52435	399	422	24	0	LRR
	MMa52435	423	446	24	0	LRR
	MMa52435	447	470	24	0	LRR
	MMa52435	471	493	23	0	LRR
	MMa52435	494	517	24	0	LRR
	MMa52435	518	541	24	0.01	LRR
	MMa52435	542	565	24	0	LRR
	MMa52435	566	588	23	0.04	LRR
	MMa52435	589	610	22	0	LRR
	MMa52435	611	635	25	0	LRR
	MMa52435	636	657	22	0	LRR
	MMa52435	658	681	24	0.04	LRR
	MMa52435	682	834	153	0	Potential LRRCT
	MMa52435	835	882	48	0	LRR
	MMa52435	883	906	24	0	LRR
	MMa52435	907	930	24	0	LRR
	MMa52435	931	954	24	0	LRR
	MMa52435	955	1234	280	0.02	Potential LRRCT
	MMa52435					
	MMa37636	168	191	24	0	LRR
	MMa37636	192	213	22	0	LRR
	MMa37636	214	234	21	0	LRR
	MMa37636	235	258	24	0	LRR
	MMa37636	259	282	24	0.02	LRR
	MMa37636	283	305	23	0.02	LRR
	MMa37636	306	331	26	0.01	LRR
	MMa37636	332	355	24	0	LRR
	MMa37636	356	379	24	0	LRR
	MMa37636	380	403	24	0	LRR
	MMa37636	404	427	24	0.01	LRR
	MMa37636	428	450	23	0	LRR

Clade	GeneID	Start	Stop	Length	E-value	Feature
	MMa37636	451	474	24	0	LRR
	MMa37636	475	498	24	0	LRR
	MMa37636	499	545	47	0.02	LRR
	MMa37636	546	567	22	0	LRR
	MMa37636	568	592	25	0	LRR
	MMa37636	593	614	22	0.01	LRR
	MMa37636	615	638	24	0	LRR
	MMa37636	639	788	150	0.01	Potential LRRCT
	MMa37636	789	836	48	0	LRR
	MMa37636	837	860	24	0	LRR
	MMa37636	861	884	24	0	LRR
	MMa37636	885	1194	310	0	Potential LRRCT
	MMa37636					
	MMa37517	118	141	24	0.15	InLRR
	MMa37517	142	165	24	0	LRR
	MMa37517	166	189	24	0	LRR
	MMa37517	190	214	25	0	LRR
	MMa37517	215	237	23	0.07	InLRR
	MMa37517	238	409	172	0.01	Potential LRRCT
	MMa37517	410	431	22	0	LRR
	MMa37517	432	739	308	0	Potential LRRCT
	MMa37517					
	MMa42574	170	193	24	0	LRR
	MMa42574	194	215	22	0.07	InLRR
	MMa42574	216	509	294	0	Potential LRRCT
	MMa42574					
	MMa11502	141	164	24	0.11	InLRR
	MMa11502	165	188	24	0	LRR
	MMa11502	189	212	24	0	LRR
	MMa11502	213	236	24	0	LRR
	MMa11502	237	260	24	0	LRR
	MMa11502	261	284	24	0.03	LRR

Clade	GeneID	Start	Stop	Length	E-value	Feature
	MMa11502	285	333	49	0	LRR
	MMa11502	334	381	48	0.01	LRR
	MMa11502	382	405	24	0	LRR
	MMa11502	406	445	40	0	LRR
	MMa11502	446	613	168	0	Potential LRRCT
	MMa11502	614	637	24	0	LRR
	MMa11502	638	659	22	0	LRR
	MMa11502	660	1029	370	0	Potential LRRCT
	MMa11502					
	MMa22782	149	223	75	0	LRR
	MMa22782	224	247	24	0.01	LRR
	MMa22782	248	271	24	0	LRR
	MMa22782	272	294	23	0	LRR
	MMa22782	295	321	27	0	LRR
	MMa22782	322	347	26	0	LRR
	MMa22782	348	571	224	0	Potential LRRCT
	MMa22782	572	876	305	0.12	Potential LRRCT
	MMa22782					
	MMa07778	89	142	54	0.01	LRR
	MMa07778	143	166	24	0	LRR
	MMa07778	167	196	30	0	LRR
	MMa07778	197	220	24	0	LRR
	MMa07778	221	244	24	0	LRR
	MMa07778	245	268	24	0	LRR
	MMa07778	269	292	24	0	LRR
	MMa07778	293	318	26	0	LRR
	MMa07778	319	342	24	0	LRR
	MMa07778	343	366	24	0	LRR
	MMa07778	367	404	38	0	LRR
	MMa07778	405	428	24	0	LRR
	MMa07778	429	451	23	0.11	InLRR
	MMa07778	452	473	22	0	LRR

Clade	GeneID	Start	Stop	Length	E-value	Feature
	MMa07778	474	498	25	0	LRR
	MMa07778	499	520	22	0	LRR
	MMa07778	521	544	24	0.05	InLRR
	MMa07778	545	697	153	0.03	Potential LRRCT
	MMa07778	698	721	24	0.01	LRR
	MMa07778	722	745	24	0.05	InLRR
	MMa07778	746	769	24	0	LRR
	MMa07778	770	793	24	0	LRR
	MMa07778	794	1110	317	0.01	Potential LRRCT
	MMa07778					
	MMa13223	99	152	54	0.08	InLRR
	MMa13223	153	176	24	0	LRR
	MMa13223	177	202	26	0.04	LRR
	MMa13223	203	226	24	0	LRR
	MMa13223	227	250	24	0	LRR
	MMa13223	251	274	24	0	LRR
	MMa13223	275	298	24	0	LRR
	MMa13223	299	324	26	0.01	LRR
	MMa13223	325	348	24	0	LRR
	MMa13223	349	372	24	0	LRR
	MMa13223	373	396	24	0	LRR
	MMa13223	397	420	24	0.06	InLRR
	MMa13223	421	443	23	0	LRR
	MMa13223	444	467	24	0	LRR
	MMa13223	468	491	24	0	LRR
	MMa13223	492	515	24	0	LRR
	MMa13223	516	538	23	0	LRR
	MMa13223	539	560	22	0	LRR
	MMa13223	561	585	25	0	LRR
	MMa13223	586	607	22	0	LRR
	MMa13223	608	631	24	0.05	InLRR
	MMa13223	632	784	153	0	Potential LRRCT

Clade	GeneID	Start	Stop	Length	E-value	Feature
	MMa13223	785	832	48	0	LRR
	MMa13223	833	856	24	0	LRR
	MMa13223	857	880	24	0	LRR
	MMa13223	881	1089	209	0	Potential LRRCT
	MMa13223	1090	1195	106	0.17	Potential LRRCT
	MMa13223					
	MMa40477	181	204	24	0.04	LRR
	MMa40477	205	228	24	0	LRR
	MMa40477	229	252	24	0	LRR
	MMa40477	253	276	24	0.01	LRR
	MMa40477	277	301	25	0	LRR
	MMa40477	302	325	24	0.01	LRR
	MMa40477	326	349	24	0.02	LRR
	MMa40477	350	372	23	0.01	LRR
	MMa40477	373	395	23	0	LRR
	MMa40477	396	419	24	0	LRR
	MMa40477	420	443	24	0	LRR
	MMa40477	444	466	23	0	LRR
	MMa40477	467	490	24	0	LRR
	MMa40477	491	513	23	0	LRR
	MMa40477	514	537	24	0	LRR
	MMa40477	538	632	95	0.02	Potential LRRCT
	MMa40477	633	655	23	0.01	LRR
	MMa40477	656	676	21	0	LRR
	MMa40477	677	699	23	0	LRR
	MMa40477	700	972	273	0	Potential LRRCT
	MMa40477					
	aug3.g494.t1	134	185	52	0.15	InLRR
	aug3.g494.t1	186	209	24	0.01	LRR
	aug3.g494.t1	210	239	30	0.17	InLRR
	aug3.g494.t1	240	263	24	0	LRR
	aug3.g494.t1	264	287	24	0	LRR

Clade	GeneID	Start	Stop	Length	E-value	Feature
	aug3.g494.t1	288	311	24	0	LRR
	aug3.g494.t1	312	335	24	0.01	LRR
	aug3.g494.t1	336	360	25	0	LRR
	aug3.g494.t1	361	384	24	0	LRR
	aug3.g494.t1	385	408	24	0	LRR
	aug3.g494.t1	409	432	24	0	LRR
	aug3.g494.t1	433	456	24	0	LRR
	aug3.g494.t1	457	479	23	0.01	LRR
	aug3.g494.t1	480	503	24	0	LRR
	aug3.g494.t1	504	527	24	0.04	LRR
	aug3.g494.t1	528	574	47	0	LRR
	aug3.g494.t1	575	596	22	0	LRR
	aug3.g494.t1	597	621	25	0	LRR
	aug3.g494.t1	622	667	46	0	LRR
	aug3.g494.t1	668	820	153	0	Potential LRRCT
	aug3.g494.t1	821	844	24	0.02	LRR
	aug3.g494.t1	845	868	24	0.01	LRR
	aug3.g494.t1	869	892	24	0	LRR
	aug3.g494.t1	893	916	24	0	LRR
	aug3.g494.t1	917	1236	320	0	Potential LRRCT
	aug3.g494.t1					
	aug3.g1692.t1	89	142	54	0.06	InLRR
	aug3.g1692.t1	143	166	24	0	LRR
	aug3.g1692.t1	167	198	32	0	LRR
	aug3.g1692.t1	199	222	24	0	LRR
	aug3.g1692.t1	223	246	24	0	LRR
	aug3.g1692.t1	247	270	24	0	LRR
	aug3.g1692.t1	271	294	24	0	LRR
	aug3.g1692.t1	295	319	25	0	LRR
	aug3.g1692.t1	320	343	24	0	LRR
	aug3.g1692.t1	344	367	24	0	LRR
	aug3.g1692.t1	368	391	24	0	LRR

Clade	GeneID	Start	Stop	Length	E-value	Feature
	aug3.g1692.t1	392	415	24	0	LRR
	aug3.g1692.t1	416	438	23	0	LRR
	aug3.g1692.t1	439	462	24	0	LRR
	aug3.g1692.t1	463	486	24	0	LRR
	aug3.g1692.t1	487	510	24	0	LRR
	aug3.g1692.t1	511	533	23	0.01	LRR
	aug3.g1692.t1	534	555	22	0.01	LRR
	aug3.g1692.t1	556	580	25	0	LRR
	aug3.g1692.t1	581	602	22	0.01	LRR
	aug3.g1692.t1	603	626	24	0.19	InLRR
	aug3.g1692.t1	627	779	153	0	Potential LRRCT
	aug3.g1692.t1	780	827	48	0	LRR
	aug3.g1692.t1	828	851	24	0	LRR
	aug3.g1692.t1	852	875	24	0	LRR
	aug3.g1692.t1	876	899	24	0	LRR
	aug3.g1692.t1	900	1166	267	0	Potential LRRCT
	aug3.g1692.t1					
	aug3.g2549.t1	113	166	54	0.04	LRR
	aug3.g2549.t1	167	190	24	0	LRR
	aug3.g2549.t1	191	220	30	0	LRR
	aug3.g2549.t1	221	244	24	0	LRR
	aug3.g2549.t1	245	268	24	0	LRR
	aug3.g2549.t1	269	292	24	0	LRR
	aug3.g2549.t1	293	316	24	0	LRR
	aug3.g2549.t1	317	342	26	0	LRR
	aug3.g2549.t1	343	366	24	0	LRR
	aug3.g2549.t1	367	390	24	0	LRR
	aug3.g2549.t1	391	414	24	0	LRR
	aug3.g2549.t1	415	438	24	0	LRR
	aug3.g2549.t1	439	461	23	0	LRR
	aug3.g2549.t1	462	485	24	0	LRR
	aug3.g2549.t1	486	509	24	0	LRR

Clade	GeneID	Start	Stop	Length	E-value	Feature
	aug3.g2549.t1	510	533	24	0	LRR
	aug3.g2549.t1	534	556	23	0	LRR
	aug3.g2549.t1	557	578	22	0	LRR
	aug3.g2549.t1	579	603	25	0	LRR
	aug3.g2549.t1	604	625	22	0	LRR
	aug3.g2549.t1	626	649	24	0.01	LRR
	aug3.g2549.t1	650	802	153	0.04	Potential LRRCT
	aug3.g2549.t1	803	850	48	0.02	LRR
	aug3.g2549.t1	851	874	24	0	LRR
	aug3.g2549.t1	875	898	24	0	LRR
	aug3.g2549.t1	899	922	24	0	LRR
	aug3.g2549.t1	923	1114	192	0.02	Potential LRRCT
	aug3.g2549.t1	1115	1239	125	0.14	Potential LRRCT
	aug3.g2549.t1					
	aug3.g6063.t1	152	175	24	0	LRR
	aug3.g6063.t1	176	205	30	0	LRR
	aug3.g6063.t1	206	229	24	0	LRR
	aug3.g6063.t1	230	253	24	0.02	LRR
	aug3.g6063.t1	254	277	24	0	LRR
	aug3.g6063.t1	278	301	24	0.01	LRR
	aug3.g6063.t1	302	327	26	0	LRR
	aug3.g6063.t1	328	351	24	0	LRR
	aug3.g6063.t1	352	375	24	0	LRR
	aug3.g6063.t1	376	399	24	0	LRR
	aug3.g6063.t1	400	423	24	0.01	LRR
	aug3.g6063.t1	424	446	23	0	LRR
	aug3.g6063.t1	447	470	24	0	LRR
	aug3.g6063.t1	471	494	24	0	LRR
	aug3.g6063.t1	495	518	24	0	LRR
	aug3.g6063.t1	519	541	23	0.07	InLRR
	aug3.g6063.t1	542	563	22	0	LRR
	aug3.g6063.t1	564	588	25	0	LRR

Clade	GeneID	Start	Stop	Length	E-value	Feature
	aug3.g6063.t1	589	610	22	0	LRR
	aug3.g6063.t1	611	634	24	0.06	lnLRR
	aug3.g6063.t1	635	663	29	0.01	LRR
	aug3.g6063.t1	664	787	124	0.01	Potential LRRCT
	aug3.g6063.t1	788	835	48	0.03	LRR
	aug3.g6063.t1	836	859	24	0	LRR
	aug3.g6063.t1	860	883	24	0	LRR
	aug3.g6063.t1	884	1202	319	0	Potential LRRCT
	aug3.g6063.t1					
	aug3.g11043.t2	555	600	46	0.17	lnLRR
	aug3.g11043.t2	601	624	24	0	LRR
	aug3.g11043.t2	625	648	24	0	LRR
	aug3.g11043.t2	649	670	22	0.02	LRR
	aug3.g11043.t2	671	693	23	0	LRR
	aug3.g11043.t2	694	719	26	0.01	LRR
	aug3.g11043.t2	720	743	24	0.13	lnLRR
	aug3.g11043.t2	744	767	24	0	LRR
	aug3.g11043.t2	768	790	23	0.12	lnLRR
	aug3.g11043.t2	791	845	55	0	LRR
	aug3.g11043.t2	846	871	26	0.01	LRR
	aug3.g11043.t2	872	895	24	0.13	lnLRR
	aug3.g11043.t2	896	919	24	0	LRR
	aug3.g11043.t2	920	942	23	0.12	lnLRR
	aug3.g11043.t2	943	965	23	0	LRR
	aug3.g11043.t2	966	989	24	0	LRR
	aug3.g11043.t2	990	1013	24	0.02	LRR
	aug3.g11043.t2	1014	1036	23	0	LRR
	aug3.g11043.t2	1037	1060	24	0	LRR
	aug3.g11043.t2	1061	1083	23	0.03	LRR
	aug3.g11043.t2	1084	1135	52	0	Potential LRRCT
	aug3.g11043.t2	1136	1208	73	0.01	Potential LRRCT
	aug3.g11043.t2	1209	1232	24	0.03	LRR

Clade	GeneID	Start	Stop	Length	E-value	Feature
	aug3.g11043.t2	1233	1253	21	0	LRR
	aug3.g11043.t2	1254	1274	21	0	LRR
	aug3.g11043.t2	1275	1559	285	0.05	Potential LRRCT
	aug3.g11043.t2					
	aug3.g12127.t1	54	77	24	0.04	LRR
	aug3.g12127.t1	78	98	21	0	LRR
	aug3.g12127.t1	99	119	21	0	LRR
	aug3.g12127.t1	120	409	290	0.01	Potential LRRCT
	aug3.g12127.t1					
	aug3.g12128.t3	18	63	46	0.02	LRR
	aug3.g12128.t3	64	87	24	0	LRR
	aug3.g12128.t3	88	111	24	0.01	LRR
	aug3.g12128.t3	112	133	22	0.14	InLRR
	aug3.g12128.t3	134	180	47	0	LRR
	aug3.g12128.t3	181	203	23	0	LRR
	aug3.g12128.t3	204	227	24	0	LRR
	aug3.g12128.t3	228	251	24	0	LRR
	aug3.g12128.t3	252	275	24	0.01	LRR
	aug3.g12128.t3	276	620	345	0.1	Potential LRRCT
	aug3.g12128.t3					
	aug3.g13589.t1	12	34	23	0	LRR
	aug3.g13589.t1	35	56	22	0	LRR
	aug3.g13589.t1	57	385	329	0	Potential LRRCT
	aug3.g13589.t1					
	aug3.g17600.t3	145	192	48	0.01	LRR
	aug3.g17600.t3	193	216	24	0.02	LRR
	aug3.g17600.t3	217	262	46	0	LRR
	aug3.g17600.t3	263	311	49	0	LRR
	aug3.g17600.t3	312	402	91	0	LRR
	aug3.g17600.t3	403	425	23	0.05	InLRR
	aug3.g17600.t3	426	449	24	0	LRR
	aug3.g17600.t3	450	472	23	0	LRR

Clade	GeneID	Start	Stop	Length	E-value	Feature
	aug3.g17600.t3	473	494	22	0	LRR
	aug3.g17600.t3	495	514	20	0	Potential LRRCT
	aug3.g17600.t3	515	613	99	0.06	Potential LRRCT
	aug3.g17600.t3	614	635	22	0.01	LRR
	aug3.g17600.t3	636	659	24	0	LRR
	aug3.g17600.t3	660	1154	495	0	Potential LRRCT
	aug3.g17600.t3	1155	1178	24	0	LRR
	aug3.g17600.t3	1179	1203	25	0	LRR
	aug3.g17600.t3	1204	1227	24	0.01	LRR
	aug3.g17600.t3	1228	1250	23	0	LRR
	aug3.g17600.t3	1251	1270	20	0	LRR
	aug3.g17600.t3	1271	1341	71	0	LRR
	aug3.g17600.t3	1342	1364	23	0	LRR
	aug3.g17600.t3	1365	1397	33	0	LRR
	aug3.g17600.t3	1398	1420	23	0.01	LRR
	aug3.g17600.t3	1421	1442	22	0	LRR
	aug3.g17600.t3	1443	1563	121	0	Potential LRRCT
	aug3.g17600.t3	1564	1585	22	0.04	LRR
	aug3.g17600.t3	1586	1889	304	0	Potential LRRCT
	aug3.g17600.t3					
	aug3.g18079.t1	181	306	126	0.05	Potential LRRCT
	aug3.g18079.t1	307	328	22	0.01	LRR
	aug3.g18079.t1	329	614	286	0	Potential LRRCT
	aug3.g18079.t1					
	aug3.g18263.t1	107	152	46	0.05	InLRR
	aug3.g18263.t1	153	176	24	0	LRR
	aug3.g18263.t1	177	200	24	0.01	LRR
	aug3.g18263.t1	201	247	47	0.14	InLRR
	aug3.g18263.t1	248	293	46	0.05	InLRR
	aug3.g18263.t1	294	317	24	0	LRR
	aug3.g18263.t1	318	341	24	0.01	LRR
	aug3.g18263.t1	342	363	22	0.18	InLRR

Clade	GeneID	Start	Stop	Length	E-value	Feature
	aug3.g18263.t1	364	387	24	0.01	LRR
	aug3.g18263.t1	388	410	23	0.06	InLRR
	aug3.g18263.t1	411	433	23	0	LRR
	aug3.g18263.t1	434	457	24	0	LRR
	aug3.g18263.t1	458	481	24	0	LRR
	aug3.g18263.t1	482	505	24	0.01	LRR
	aug3.g18263.t1	506	850	345	0.12	Potential LRRCT
	aug3.g18263.t1					
	aug3.g19333.t1	145	168	24	0	LRR
	aug3.g19333.t1	169	192	24	0	LRR
	aug3.g19333.t1	193	216	24	0.01	LRR
	aug3.g19333.t1	217	240	24	0	LRR
	aug3.g19333.t1	241	288	48	0	LRR
	aug3.g19333.t1	289	337	49	0.01	LRR
	aug3.g19333.t1	338	361	24	0	LRR
	aug3.g19333.t1	362	385	24	0.02	LRR
	aug3.g19333.t1	386	409	24	0.06	InLRR
	aug3.g19333.t1	410	433	24	0	LRR
	aug3.g19333.t1	434	454	21	0	LRR
	aug3.g19333.t1	455	478	24	0	LRR
	aug3.g19333.t1	479	643	165	0	Potential LRRCT
	aug3.g19333.t1	644	689	46	0.06	InLRR
	aug3.g19333.t1	690	1037	348	0	Potential LRRCT
	aug3.g19333.t1					
	aug3.g20004.t2	64	178	115	0.03	LRR
	aug3.g20004.t2	179	202	24	0	LRR
	aug3.g20004.t2	203	224	22	0	LRR
	aug3.g20004.t2	225	248	24	0	LRR
	aug3.g20004.t2	249	273	25	0	LRR
	aug3.g20004.t2	274	297	24	0	LRR
	aug3.g20004.t2	298	321	24	0	LRR
	aug3.g20004.t2	322	371	50	0.1	InLRR

Clade	GeneID	Start	Stop	Length	E-value	Feature
	aug3.g20004.t2	372	395	24	0	LRR
	aug3.g20004.t2	396	419	24	0	LRR
	aug3.g20004.t2	420	465	46	0	LRR
	aug3.g20004.t2	466	488	23	0	LRR
	aug3.g20004.t2	489	516	28	0	LRR
	aug3.g20004.t2	517	620	104	0.05	Potential LRRCT
	aug3.g20004.t2	621	643	23	0.12	InLRR
	aug3.g20004.t2	644	664	21	0	LRR
	aug3.g20004.t2	665	687	23	0	LRR
	aug3.g20004.t2	688	988	301	0.19	Potential LRRCT
	aug3.g20004.t2					
	aug3.g20240.t1	21	43	23	0.01	LRR
	aug3.g20240.t1	44	65	22	0	LRR
	aug3.g20240.t1	66	186	121	0	Potential LRRCT
	aug3.g20240.t1	187	208	22	0.05	InLRR
	aug3.g20240.t1	209	649	441	0	Potential LRRCT
	aug3.g20240.t1	650	673	24	0.08	InLRR
	aug3.g20240.t1	674	697	24	0.01	LRR
	aug3.g20240.t1	698	920	223	0	Potential LRRCT
	aug3.g20240.t1	921	944	24	0.08	InLRR
	aug3.g20240.t1	945	968	24	0.01	LRR
	aug3.g20240.t1	969	996	28	0	LRR
	aug3.g20240.t1	997	1020	24	0.01	LRR
	aug3.g20240.t1	1021	1066	46	0	LRR
	aug3.g20240.t1	1067	1091	25	0	LRR
	aug3.g20240.t1	1092	1116	25	0	LRR
	aug3.g20240.t1	1117	1162	46	0	LRR
	aug3.g20240.t1	1163	1206	44	0.01	LRR
	aug3.g20240.t1	1207	1230	24	0.01	LRR
	aug3.g20240.t1	1231	1253	23	0	LRR
	aug3.g20240.t1	1254	1290	37	0	LRR
	aug3.g20240.t1	1291	1312	22	0.02	LRR

Clade	GeneID	Start	Stop	Length	E-value	Feature
	aug3.g20240.t1	1313	1336	24	0	LRR
	aug3.g20240.t1	1337	1843	507	0	Potential LRRCT
	aug3.g20240.t1	1844	1867	24	0.06	InLRR
	aug3.g20240.t1	1868	1891	24	0	LRR
	aug3.g20240.t1	1892	1937	46	0	LRR
	aug3.g20240.t1	1938	1951	14	0	LRR
	aug3.g20240.t1					
	aug3.g25912.t1	158	181	24	0	LRR
	aug3.g25912.t1	182	213	32	0.01	LRR
	aug3.g25912.t1	214	237	24	0.02	LRR
	aug3.g25912.t1	238	261	24	0	LRR
	aug3.g25912.t1	262	285	24	0	LRR
	aug3.g25912.t1	286	309	24	0	LRR
	aug3.g25912.t1	310	335	26	0	LRR
	aug3.g25912.t1	336	359	24	0	LRR
	aug3.g25912.t1	360	383	24	0.01	LRR
	aug3.g25912.t1	384	407	24	0	LRR
	aug3.g25912.t1	408	431	24	0.16	InLRR
	aug3.g25912.t1	432	454	23	0.03	LRR
	aug3.g25912.t1	455	478	24	0	LRR
	aug3.g25912.t1	479	502	24	0	LRR
	aug3.g25912.t1	503	526	24	0	LRR
	aug3.g25912.t1	527	549	23	0.01	LRR
	aug3.g25912.t1	550	571	22	0	LRR
	aug3.g25912.t1	572	596	25	0	LRR
	aug3.g25912.t1	597	618	22	0	LRR
	aug3.g25912.t1	619	642	24	0.09	InLRR
	aug3.g25912.t1	643	795	153	0.01	Potential LRRCT
	aug3.g25912.t1	796	843	48	0.01	LRR
	aug3.g25912.t1	844	867	24	0	LRR
	aug3.g25912.t1	868	891	24	0	LRR
	aug3.g25912.t1	892	1202	311	0	Potential LRRCT

Clade	GeneID	Start	Stop	Length	E-value	Feature
	aug3.g25912.t1					
	TOL1_CELEG	85	109	25	161.8	LRR
	TOL1_CELEG	110	133	24	3.13	LRR
	TOL1_CELEG	134	161	28	1.05	LRR
	TOL1_CELEG	162	185	24	6.38	LRR
	TOL1_CELEG	186	209	24	0	LRR
	TOL1_CELEG	210	232	23	0	LRR
	TOL1_CELEG	233	254	22	0.46	LRR
	TOL1_CELEG	255	274	20	0	LRR
	TOL1_CELEG	275	297	23	0.15	LRR
	TOL1_CELEG	298	320	23	0	LRR
	TOL1_CELEG	321	345	25	0	LRR
	TOL1_CELEG	346	369	24	0.01	LRR
	TOL1_CELEG	370	393	24	0	LRR
	TOL1_CELEG	394	416	23	0.01	LRR
	TOL1_CELEG	417	439	23	0	LRR
	TOL1_CELEG	440	463	24	0	LRR
	TOL1_CELEG	464	487	24	0	LRR
	TOL1_CELEG	488	511	24	0	LRR
	TOL1_CELEG	512	534	23	0.02	LRR
	TOL1_CELEG	535	556	22	0	LRR
	TOL1_CELEG	557	581	25	0	LRR
	TOL1_CELEG	582	603	22	0	LRR
	TOL1_CELEG	604	627	24	0	LRR
	TOL1_CELEG	628	653	26	0	LRR
	TOL1_CELEG	654	653	0	96.99	Potential LRRCT
	TOL1_CELEG	800	823	24	0.03	LRR
	TOL1_CELEG	824	847	24	0	LRR
	TOL1_CELEG	848	871	24	0.02	LRR
	TOL1_CELEG	872	893	22	0	LRR
	TOL1_CELEG	894	893	0	0.01	Potential LRRCT
	TOL1_CELEG					

Clade	GeneID	Start	Stop	Length	E-value	Feature
	TOL1_CELEG	85	109	25	161.8	LRR
	TOL1_CELEG	110	133	24	3.13	LRR
	TOL1_CELEG	134	161	28	1.05	LRR
	TOL1_CELEG	162	185	24	6.38	LRR
	TOL1_CELEG	186	209	24	0	LRR
	TOL1_CELEG	210	232	23	0	LRR
	TOL1_CELEG	233	254	22	0.46	LRR
	TOL1_CELEG	255	274	20	0	LRR
	TOL1_CELEG	275	297	23	0.15	LRR
	TOL1_CELEG	298	320	23	0	LRR
	TOL1_CELEG	321	345	25	0	LRR
	TOL1_CELEG	346	369	24	0.01	LRR
	TOL1_CELEG	370	393	24	0	LRR
	TOL1_CELEG	394	416	23	0.01	LRR
	TOL1_CELEG	417	439	23	0	LRR
	TOL1_CELEG	440	463	24	0	LRR
	TOL1_CELEG	464	487	24	0	LRR
	TOL1_CELEG	488	511	24	0	LRR
	TOL1_CELEG	512	534	23	0.02	LRR
	TOL1_CELEG	535	556	22	0	LRR
	TOL1_CELEG	557	581	25	0	LRR
	TOL1_CELEG	582	603	22	0	LRR
	TOL1_CELEG	604	627	24	0	LRR
	TOL1_CELEG	628	653	26	0	LRR
	TOL1_CELEG	654	653	0	96.99	Potential LRRCT
	TOL1_CELEG	800	823	24	0.03	LRR
	TOL1_CELEG	824	847	24	0	LRR
	TOL1_CELEG	848	871	24	0.02	LRR
	TOL1_CELEG	872	893	22	0	LRR
	TOL1_CELEG	894	893	0	0.01	Potential LRRCT
	TOL1_CELEG					
	Tl Toll-1	160	183	24	215.82	LRR

Clade	GeneID	Start	Stop	Length	E-value	Feature
	Tl Toll-1	184	206	23	0.13	LRR
	Tl Toll-1	207	230	24	0.01	LRR
	Tl Toll-1	231	254	24	0	LRR
	Tl Toll-1	255	278	24	3.48	LRR
	Tl Toll-1	279	302	24	0.26	LRR
	Tl Toll-1	303	328	26	0.45	LRR
	Tl Toll-1	329	351	23	18.01	LRR
	Tl Toll-1	352	375	24	0.5	LRR
	Tl Toll-1	376	399	24	0	LRR
	Tl Toll-1	400	423	24	0.02	LRR
	Tl Toll-1	424	447	24	0	LRR
	Tl Toll-1	448	482	35	0	LRR
	Tl Toll-1	483	506	24	0	LRR
	Tl Toll-1	507	531	25	0	LRR
	Tl Toll-1	532	560	29	27.94	LRR
	Tl Toll-1	561	584	24	0.01	Potential LRRCT
	Tl Toll-1	654	677	24	93,259.23	LRR
	Tl Toll-1	678	701	24	0	LRR
	Tl Toll-1	702	723	22	0	LRR
	Tl Toll-1	724	750	27	0	LRR
	Tl Toll-1	751	774	24	0.32	Potential LRRCT
	Tl Toll-1					
	18w Toll-2	99	153	55	0.1	InLRR
	18w Toll-2	154	177	24	0	LRR
	18w Toll-2	178	218	41	0	LRR
	18w Toll-2	219	243	25	0	LRR
	18w Toll-2	244	267	24	0	LRR
	18w Toll-2	268	291	24	0	LRR
	18w Toll-2	292	315	24	0	LRR
	18w Toll-2	316	341	26	0	LRR
	18w Toll-2	342	365	24	0	LRR
	18w Toll-2	366	389	24	0	LRR

Clade	GeneID	Start	Stop	Length	E-value	Feature
	18w Toll-2	390	413	24	0	LRR
	18w Toll-2	414	437	24	0	LRR
	18w Toll-2	438	460	23	0	LRR
	18w Toll-2	461	484	24	0	LRR
	18w Toll-2	485	508	24	0	LRR
	18w Toll-2	509	532	24	0	LRR
	18w Toll-2	533	555	23	0.14	InLRR
	18w Toll-2	556	577	22	0	LRR
	18w Toll-2	578	602	25	0	LRR
	18w Toll-2	603	624	22	0	LRR
	18w Toll-2	625	800	176	0.13	Potential LRRCT
	18w Toll-2	801	824	24	0.01	LRR
	18w Toll-2	825	848	24	0.16	InLRR
	18w Toll-2	849	872	24	0	LRR
	18w Toll-2	873	896	24	0	LRR
	18w Toll-2	897	918	22	0	LRR
	18w Toll-2	919	1393	475	0.03	Potential LRRCT
	18w Toll-2					
	MstProx Toll-3	331	354	24	0.06	InLRR
	MstProx Toll-3	355	376	22	0	LRR
	MstProx Toll-3	377	570	194	0	Potential LRRCT
	MstProx Toll-3	571	592	22	0.04	LRR
	MstProx Toll-3	593	697	105	0	Potential LRRCT
	MstProx Toll-3	698	974	277	0	Potential LRRCT
	MstProx Toll-3					
	Toll-4	440	461	22	0	LRR
	Toll-4	462	637	176	0	Potential LRRCT
	Toll-4	638	664	27	0.11	InLRR
	Toll-4	665	686	22	0.02	LRR
	Toll-4	687	713	27	0	LRR
	Toll-4	714	822	109	0.06	Potential LRRCT
	Toll-4	823	842	20	0.02	LRR

Clade	GeneID	Start	Stop	Length	E-value	Feature
	Toll-4	843	1133	291	0	Potential LRRCT
	Toll-4					
	Tehao Toll-5	193	216	24	0.08	InLRR
	Tehao Toll-5	217	317	101	0.16	InLRR
	Tehao Toll-5	318	341	24	0	LRR
	Tehao Toll-5	342	365	24	0	LRR
	Tehao Toll-5	366	490	125	0	Potential LRRCT
	Tehao Toll-5	491	513	23	0.01	LRR
	Tehao Toll-5	514	540	27	0	LRR
	Tehao Toll-5	541	804	264	0	Potential LRRCT
	Tehao Toll-5					
	Toll-6	155	178	24	0.01	LRR
	Toll-6	179	208	30	0.13	InLRR
	Toll-6	209	232	24	0	LRR
	Toll-6	233	285	53	0	LRR
	Toll-6	286	309	24	0	LRR
	Toll-6	310	333	24	0	LRR
	Toll-6	334	358	25	0	LRR
	Toll-6	359	382	24	0.01	LRR
	Toll-6	383	405	23	0	LRR
	Toll-6	406	432	27	71.15	LRR
	Toll-6	433	456	24	0	LRR
	Toll-6	457	480	24	0	LRR
	Toll-6	481	504	24	0.01	LRR
	Toll-6	505	527	23	0	LRR
	Toll-6	528	551	24	0	LRR
	Toll-6	552	575	24	0.06	InLRR
	Toll-6	576	622	47	0	LRR
	Toll-6	623	644	22	0	LRR
	Toll-6	645	669	25	0	LRR
	Toll-6	670	691	22	0	LRR
	Toll-6	692	715	24	0.05	LRR

Clade	GeneID	Start	Stop	Length	E-value	Feature
	Toll-6	716	744	29	0	LRR
	Toll-6	745	744	0	0.14	Potential LRRCT
	Toll-6	868	915	48	0.05	InLRR
	Toll-6	916	939	24	0	LRR
	Toll-6	940	963	24	0	LRR
	Toll-6	964	987	24	0	LRR
	Toll-6	988	1522	535	0.05	Potential LRRCT
	Toll-6					
	Toll-7	143	197	55	0.01	LRR
	Toll-7	198	221	24	0	LRR
	Toll-7	222	255	34	0	LRR
	Toll-7	256	280	25	0	LRR
	Toll-7	281	304	24	0	LRR
	Toll-7	305	328	24	0	LRR
	Toll-7	329	352	24	0.02	LRR
	Toll-7	353	378	26	0	LRR
	Toll-7	379	402	24	0	LRR
	Toll-7	403	426	24	0	LRR
	Toll-7	427	450	24	0	LRR
	Toll-7	451	474	24	0	LRR
	Toll-7	475	497	23	0	LRR
	Toll-7	498	521	24	0	LRR
	Toll-7	522	545	24	0	LRR
	Toll-7	546	569	24	0	LRR
	Toll-7	570	592	23	0.02	LRR
	Toll-7	593	614	22	0	LRR
	Toll-7	615	639	25	0	LRR
	Toll-7	640	661	22	0	LRR
	Toll-7	662	714	53	0.04	LRR
	Toll-7	715	738	24	0.04	Potential LRRCT
	Toll-7	886	909	24	0	LRR
	Toll-7	910	933	24	0	LRR

Clade	GeneID	Start	Stop	Length	E-value	Feature
	Toll-7	934	960	27	0.04	LRR
	Toll-7	961	1454	494	0.01	Potential LRRCT
	Toll-7					
	Tollo Toll-8	108	161	54	0.02	LRR
	Tollo Toll-8	162	185	24	0	LRR
	Tollo Toll-8	186	219	34	0	LRR
	Tollo Toll-8	220	243	24	0	LRR
	Tollo Toll-8	244	267	24	0	LRR
	Tollo Toll-8	268	291	24	0	LRR
	Tollo Toll-8	292	315	24	0	LRR
	Tollo Toll-8	316	341	26	0	LRR
	Tollo Toll-8	342	365	24	0	LRR
	Tollo Toll-8	366	389	24	0	LRR
	Tollo Toll-8	390	413	24	0	LRR
	Tollo Toll-8	414	437	24	0	LRR
	Tollo Toll-8	438	460	23	0	LRR
	Tollo Toll-8	461	508	48	0	LRR
	Tollo Toll-8	509	532	24	0	LRR
	Tollo Toll-8	533	555	23	0.01	LRR
	Tollo Toll-8	556	577	22	0	LRR
	Tollo Toll-8	578	602	25	0	LRR
	Tollo Toll-8	603	624	22	0.02	LRR
	Tollo Toll-8	625	648	24	0	LRR
	Tollo Toll-8	649	800	152	0	Potential LRRCT
	Tollo Toll-8	801	824	24	0.05	InLRR
	Tollo Toll-8	825	848	24	0	LRR
	Tollo Toll-8	849	872	24	0	LRR
	Tollo Toll-8	873	896	24	0	LRR
	Tollo Toll-8	897	919	23	0	LRR
	Tollo Toll-8	920	1355	436	0.01	Potential LRRCT
	Tollo Toll-8					

Clade	GeneID	Start	Stop	Length	E-value	Feature
**Vertebrate TLR like**	DappuP314608	106	129	24	0.02	LRR
**sccTLR clade**	DappuP314608	130	153	24	0	LRR
	DappuP314608	154	179	26	0	LRR
	DappuP314608	180	205	26	0	LRR
	DappuP314608	206	229	24	0	LRR
	DappuP314608	230	256	27	0	LRR
	DappuP314608	257	283	27	0	LRR
	DappuP314608	284	307	24	0	LRR
	DappuP314608	308	331	24	0.02	LRR
	DappuP314608	332	354	23	0	LRR
	DappuP314608	355	387	33	0	LRR
	DappuP314608	388	411	24	0	LRR
	DappuP314608	412	435	24	0	LRR
	DappuP314608	436	461	26	0	LRR
	DappuP314608	462	486	25	0.01	LRR
	DappuP314608	487	511	25	0	LRR
	DappuP314608	512	535	24	0.01	LRR
	DappuP314608	536	559	24	0	LRR
	DappuP314608	560	779	220	0.01	Potential LRRCT
	DappuP314608	780	861	82	0.12	InLRR
	DappuP314608					
	SMAR004491-PA	101	124	24	0	LRR
	SMAR004491-PA	125	148	24	0	LRR
	SMAR004491-PA	149	201	53	0	LRR
	SMAR004491-PA	202	225	24	0.1	InLRR
	SMAR004491-PA	226	277	52	0	LRR
	SMAR004491-PA	278	301	24	0.04	LRR
	SMAR004491-PA	302	325	24	0	LRR
	SMAR004491-PA	326	348	23	0	LRR
	SMAR004491-PA	349	379	31	0.01	LRR
	SMAR004491-PA	380	450	71	0.02	LRR

Clade	GeneID	Start	Stop	Length	E-value	Feature
	SMAR004491-PA	451	475	25	0.02	LRR
	SMAR004491-PA	476	499	24	0	LRR
	SMAR004491-PA	500	824	325	0.03	Potential LRRCT
	SMAR004491-PA					
	tetur36g00940.1	175	199	25	0.04	LRR
	tetur36g00940.1	200	224	25	0	LRR
	tetur36g00940.1	225	368	144	0.08	InLRR
	tetur36g00940.1	369	393	25	0.01	LRR
	tetur36g00940.1	394	467	74	0.16	InLRR
	tetur36g00940.1	468	491	24	0.05	InLRR
	tetur36g00940.1	492	515	24	0	LRR
	tetur36g00940.1	516	546	31	0	LRR
	tetur36g00940.1	547	570	24	0	LRR
	tetur36g00940.1	571	623	53	0.01	LRR
	tetur36g00940.1	624	651	28	0.08	InLRR
	tetur36g00940.1	652	675	24	0	LRR
	tetur36g00940.1	676	700	25	0	LRR
	tetur36g00940.1	701	724	24	0	LRR
	tetur36g00940.1	725	1244	520	0	Potential LRRCT
	MMa34077	114	137	24	0	LRR
	MMa34077	138	161	24	0	LRR
	MMa34077	162	187	26	0	LRR
	MMa34077	188	213	26	0.04	LRR
	MMa34077	214	237	24	0.01	LRR
	MMa34077	238	289	52	0	LRR
	MMa34077	290	337	48	0	LRR
	MMa34077	338	389	52	0	LRR
	MMa34077	390	413	24	0.01	LRR
	MMa34077	414	437	24	0.04	LRR
	MMa34077	438	461	24	0	LRR
	MMa34077	462	486	25	0.02	LRR

Clade	GeneID	Start	Stop	Length	E-value	Feature
	MMa34077	487	510	24	0.05	InLRR
	MMa34077	511	559	49	0.04	LRR
	MMa34077	560	780	221	0	Potential LRRCT
	MMa34077					
	MMa02436	5	30	26	0	LRR
	MMa02436	31	54	24	0	LRR
	MMa02436	55	173	119	0	LRR
	MMa02436	174	201	28	0.02	LRR
	MMa02436	202	225	24	0	LRR
	MMa02436	226	249	24	0	LRR
	MMa02436	250	276	27	0.03	LRR
	MMa02436	277	301	25	0.15	InLRR
	MMa02436	302	325	24	0.03	LRR
	MMa02436	326	349	24	0.01	LRR
	MMa02436	350	373	24	0.04	LRR
	MMa02436	374	636	263	0	Potential LRRCT
	MMa02436					
	MMa51810	79	102	24	0.01	LRR
	MMa51810	103	126	24	0.04	LRR
	MMa51810	127	151	25	0.01	LRR
	MMa51810	152	178	27	0	LRR
	MMa51810	179	202	24	0.05	InLRR
	MMa51810	203	256	54	0	LRR
	MMa51810	257	302	46	0.01	LRR
	MMa51810	303	326	24	0.01	LRR
	MMa51810	327	355	29	0	LRR
	MMa51810	356	404	49	0.02	LRR
	MMa51810	405	428	24	0	LRR
	MMa51810	429	453	25	0	LRR
	MMa51810	454	476	23	0	LRR
	MMa51810	477	500	24	0	LRR
	MMa51810	501	791	291	0.18	Potential LRRCT

Clade	GeneID	Start	Stop	Length	E-value	Feature
	MMa51810					
	MMa34079	114	137	24	0	LRR
	MMa34079	138	161	24	0	LRR
	MMa34079	162	187	26	0	LRR
	MMa34079	188	213	26	0.04	LRR
	MMa34079	214	237	24	0.01	LRR
	MMa34079	238	289	52	0	LRR
	MMa34079	290	337	48	0	LRR
	MMa34079	338	389	52	0	LRR
	MMa34079	390	413	24	0.01	LRR
	MMa34079	414	437	24	0.04	LRR
	MMa34079	438	461	24	0	LRR
	MMa34079	462	486	25	0.02	LRR
	MMa34079	487	510	24	0.05	InLRR
	MMa34079	511	559	49	0.04	LRR
	MMa34079	560	819	260	0	Potential LRRCT
	MMa34079					
	aug3.g20941.t1	239	261	23	0	LRR
	aug3.g20941.t1	262	284	23	0	LRR
	aug3.g20941.t1	285	310	26	0	LRR
	aug3.g20941.t1	311	361	51	0.06	InLRR
	aug3.g20941.t1	362	415	54	0	LRR
	aug3.g20941.t1	416	437	22	0.02	LRR
	aug3.g20941.t1	438	461	24	0.05	InLRR
	aug3.g20941.t1	462	512	51	0	LRR
	aug3.g20941.t1	513	537	25	0	LRR
	aug3.g20941.t1	538	561	24	0.06	InLRR
	aug3.g20941.t1	562	585	24	0.01	LRR
	aug3.g20941.t1	586	610	25	0	LRR
	aug3.g20941.t1	611	634	24	0.01	LRR
	aug3.g20941.t1	635	658	24	0.02	LRR
	aug3.g20941.t1	659	683	25	0.17	InLRR

Clade	GeneID	Start	Stop	Length	E-value	Feature
	aug3.g20941.t1	684	976	293	0.04	Potential LRRCT
	aug3.g20941.t1					
	TLR1_CIONA	82	109	28	0	LRR
	TLR1_CIONA	110	133	24	0.1	LRR
	TLR1_CIONA	134	157	24	0	LRR
	TLR1_CIONA	158	181	24	0	LRR
	TLR1_CIONA	182	209	28	0	LRR
	TLR1_CIONA	348	371	24	0	LRR
	TLR1_CIONA	372	396	25	0	LRR
	TLR1_CIONA	397	427	31	0	LRR
	TLR1_CIONA	428	449	22	324.72	LRR
	TLR1_CIONA	450	475	26	0	LRR
	TLR1_CIONA	476	499	24	0	LRR
	TLR1_CIONA	500	525	26	0	LRR
	TLR1_CIONA	526	551	26	1,414.74	LRR
	TLR1_CIONA	552	575	24	4.67	LRR
	TLR1_CIONA	576	599	24	0	LRR
	TLR1_CIONA	600	623	24	0	LRR
	TLR1_CIONA	624	647	24	0	Potential LRRCT
	TLR1_CIONA					
	TLR2_CIONA	81	105	25	0.43	LRR
	TLR2_CIONA	106	128	23	0.01	LRR
	TLR2_CIONA	129	152	24	2.41	LRR
	TLR2_CIONA	153	173	21	0.5	LRR
	TLR2_CIONA	174	197	24	0	LRR
	TLR2_CIONA	198	218	21	0	LRR
	TLR2_CIONA	219	242	24	0	LRR
	TLR2_CIONA	243	264	22	0	LRR
	TLR2_CIONA	265	289	25	0.05	LRR
	TLR2_CIONA	407	427	21	0	LRR
	TLR2_CIONA	428	454	27	0	LRR
	TLR2_CIONA	455	478	24	0	LRR

Clade	GeneID	Start	Stop	Length	E-value	Feature
	TLR2_CIONA	479	502	24	0.02	Potential LRRCT
	TLR2_CIONA	608	629	22	0	LRR
	TLR2_CIONA	630	655	26	0.01	LRR
	TLR2_CIONA	656	961	306	0	Potential LRRCT
	TLR2_CIONA					
	TLR10_HUMAN	82	105	24	0	LRR
	TLR10_HUMAN	106	129	24	0	LRR
	TLR10_HUMAN	130	150	21	0	LRR
	TLR10_HUMAN	151	175	25	0	LRR
	TLR10_HUMAN	176	198	23	0	LRR
	TLR10_HUMAN	199	221	23	50.19	LRR
	TLR10_HUMAN	222	245	24	25,735.45	LRR
	TLR10_HUMAN	246	275	30	495.34	LRR
	TLR10_HUMAN	276	302	27	21.82	LRR
	TLR10_HUMAN	303	330	28	284.34	LRR
	TLR10_HUMAN	331	359	29	25.42	LRR
	TLR10_HUMAN	360	381	22	0.8	LRR
	TLR10_HUMAN	382	405	24	0.06	LRR
	TLR10_HUMAN	406	430	25	0	LRR
	TLR10_HUMAN	431	454	24	0	LRR
	TLR10_HUMAN	455	476	22	0.04	LRR
	TLR10_HUMAN	477	499	23	0	LRR
	TLR10_HUMAN	500	521	22	0	LRR
	TLR10_HUMAN	522	545	24	0.01	LRR
	TLR10_HUMAN	546	569	24	0.01	Potential LRRCT
	TLR10_HUMAN					
	TLR13_MOUSE	134	157	24	0	LRR
	TLR13_MOUSE	158	181	24	0.01	LRR
	TLR13_MOUSE	182	204	23	0	LRR
	TLR13_MOUSE	205	228	24	0	LRR
	TLR13_MOUSE	229	254	26	0	LRR
	TLR13_MOUSE	255	277	23	0	LRR

Clade	GeneID	Start	Stop	Length	E-value	Feature
	TLR13_MOUSE	278	300	23	0	LRR
	TLR13_MOUSE	301	324	24	0.06	LRR
	TLR13_MOUSE	325	347	23	0	LRR
	TLR13_MOUSE	348	377	30	0.32	LRR
	TLR13_MOUSE	378	401	24	0.25	LRR
	TLR13_MOUSE	402	426	25	0.01	LRR
	TLR13_MOUSE	427	450	24	0.01	LRR
	TLR13_MOUSE	451	474	24	0	LRR
	TLR13_MOUSE	475	498	24	0	LRR
	TLR13_MOUSE	499	522	24	0	LRR
	TLR13_MOUSE	523	546	24	0	LRR
	TLR13_MOUSE	547	570	24	0	LRR
	TLR13_MOUSE	571	594	24	0	LRR
	TLR13_MOUSE	595	623	29	0	LRR
	TLR13_MOUSE	624	649	26	0.19	LRR
	TLR13_MOUSE	650	673	24	0.03	LRR
	TLR13_MOUSE	674	701	28	0.01	LRR
	TLR13_MOUSE	702	725	24	0	LRR
	TLR13_MOUSE	726	749	24	0.01	LRR
	TLR13_MOUSE	750	749	0	0.09	Potential LRRCT
	TLR13_MOUSE					
	TLR12_MOUSE	101	123	23	1,137.90	LRR
	TLR12_MOUSE	124	146	23	22.29	LRR
	TLR12_MOUSE	147	173	27	3.4	LRR
	TLR12_MOUSE	174	195	22	0.09	LRR
	TLR12_MOUSE	196	230	35	23.54	LRR
	TLR12_MOUSE	231	254	24	0	LRR
	TLR12_MOUSE	255	277	23	0.02	LRR
	TLR12_MOUSE	278	299	22	1,073.56	LRR
	TLR12_MOUSE	300	323	24	0.22	LRR
	TLR12_MOUSE	324	347	24	0.07	LRR
	TLR12_MOUSE	348	373	26	0	LRR

Clade	GeneID	Start	Stop	Length	E-value	Feature
	TLR12_MOUSE	374	397	24	0	LRR
	TLR12_MOUSE	398	421	24	0	LRR
	TLR12_MOUSE	422	445	24	0	LRR
	TLR12_MOUSE	446	469	24	0.06	LRR
	TLR12_MOUSE	470	491	22	27.13	LRR
	TLR12_MOUSE	492	516	25	29.74	LRR
	TLR12_MOUSE	517	542	26	0.06	LRR
	TLR12_MOUSE	543	571	29	0.01	LRR
	TLR12_MOUSE	572	594	23	102.52	LRR
	TLR12_MOUSE	595	623	29	17.17	LRR
	TLR12_MOUSE	624	647	24	0.14	LRR
	TLR12_MOUSE	648	672	25	0.55	LRR
	TLR12_MOUSE	673	700	28	268.5	Potential LRRCT
	TLR12_MOUSE	701	937	237	0.01	Potential LRRCT
	TLR12_MOUSE					
	TLR1_MOUSE	80	103	24	0	LRR
	TLR1_MOUSE	104	127	24	0	LRR
	TLR1_MOUSE	128	148	21	0	LRR
	TLR1_MOUSE	149	173	25	0	LRR
	TLR1_MOUSE	174	196	23	0.02	LRR
	TLR1_MOUSE	197	221	25	########	LRR
	TLR1_MOUSE	222	245	24	1,004.76	LRR
	TLR1_MOUSE	246	278	33	40.8	LRR
	TLR1_MOUSE	279	305	27	5.13	LRR
	TLR1_MOUSE	306	331	26	1.09	LRR
	TLR1_MOUSE	332	360	29	12.69	LRR
	TLR1_MOUSE	361	382	22	15.06	LRR
	TLR1_MOUSE	383	406	24	11.71	LRR
	TLR1_MOUSE	407	432	26	0	LRR
	TLR1_MOUSE	433	457	25	0	LRR
	TLR1_MOUSE	458	479	22	0	LRR
	TLR1_MOUSE	480	502	23	0	LRR

Clade	GeneID	Start	Stop	Length	E-value	Feature
	TLR1_MOUSE	503	524	22	0	LRR
	TLR1_MOUSE	525	548	24	0.04	LRR
	TLR1_MOUSE	549	572	24	0.03	Potential LRRCT
	TLR1_MOUSE					
	TLR21_CHICK	96	119	24	0	LRR
	TLR21_CHICK	120	143	24	0	LRR
	TLR21_CHICK	144	167	24	0	LRR
	TLR21_CHICK	168	192	25	0	LRR
	TLR21_CHICK	193	216	24	0.08	LRR
	TLR21_CHICK	217	240	24	0.49	LRR
	TLR21_CHICK	241	264	24	1,554.60	LRR
	TLR21_CHICK	265	291	27	391.35	LRR
	TLR21_CHICK	292	319	28	3.06	LRR
	TLR21_CHICK	320	348	29	2.3	LRR
	TLR21_CHICK	349	377	29	0.71	LRR
	TLR21_CHICK	378	401	24	0.45	LRR
	TLR21_CHICK	402	428	27	0	LRR
	TLR21_CHICK	429	454	26	0	LRR
	TLR21_CHICK	455	477	23	0	LRR
	TLR21_CHICK	478	498	21	0	LRR
	TLR21_CHICK	499	518	20	0	LRR
	TLR21_CHICK	519	540	22	0	LRR
	TLR21_CHICK	541	564	24	0.05	LRR
	TLR21_CHICK	565	588	24	0.03	Potential LRRCT
	TLR21_CHICK					
	TLR1_HUMAN	76	99	24	0.01	LRR
	TLR1_HUMAN	100	123	24	0	LRR
	TLR1_HUMAN	124	144	21	0	LRR
	TLR1_HUMAN	145	169	25	0	LRR
	TLR1_HUMAN	170	192	23	0.05	LRR
	TLR1_HUMAN	193	217	25	########	LRR
	TLR1_HUMAN	218	241	24	2,293.13	LRR

Clade	GeneID	Start	Stop	Length	E-value	Feature
	TLR1_HUMAN	242	274	33	9.92	LRR
	TLR1_HUMAN	275	301	27	6.83	LRR
	TLR1_HUMAN	302	327	26	40.83	LRR
	TLR1_HUMAN	328	356	29	40.47	LRR
	TLR1_HUMAN	357	378	22	10.67	LRR
	TLR1_HUMAN	379	402	24	0.03	LRR
	TLR1_HUMAN	403	428	26	0	LRR
	TLR1_HUMAN	429	453	25	0	LRR
	TLR1_HUMAN	454	475	22	0.01	LRR
	TLR1_HUMAN	476	498	23	0	LRR
	TLR1_HUMAN	499	520	22	0	LRR
	TLR1_HUMAN	521	544	24	0.03	LRR
	TLR1_HUMAN	545	568	24	2.51	Potential LRRCT
	TLR1_HUMAN					
	TLR22_CHICK	86	109	24	0	LRR
	TLR22_CHICK	110	133	24	0	LRR
	TLR22_CHICK	134	157	24	0	LRR
	TLR22_CHICK	158	182	25	0	LRR
	TLR22_CHICK	183	206	24	0.12	LRR
	TLR22_CHICK	207	230	24	0.38	LRR
	TLR22_CHICK	231	254	24	841.94	LRR
	TLR22_CHICK	255	281	27	182.42	LRR
	TLR22_CHICK	282	309	28	190.92	LRR
	TLR22_CHICK	310	337	28	25.22	LRR
	TLR22_CHICK	338	366	29	0.15	LRR
	TLR22_CHICK	367	390	24	139.29	LRR
	TLR22_CHICK	391	417	27	0	LRR
	TLR22_CHICK	418	443	26	0	LRR
	TLR22_CHICK	444	466	23	0	LRR
	TLR22_CHICK	467	487	21	0	LRR
	TLR22_CHICK	488	507	20	0	LRR
	TLR22_CHICK	508	529	22	0	LRR

Clade	GeneID	Start	Stop	Length	E-value	Feature
	TLR22_CHICK	530	553	24	0.05	LRR
	TLR22_CHICK	554	577	24	0.02	Potential LRRCT
	TLR22_CHICK					
	TLR11_MOUSE	112	134	23	79.83	LRR
	TLR11_MOUSE	135	157	23	1,263.58	LRR
	TLR11_MOUSE	158	185	28	5.76	LRR
	TLR11_MOUSE	186	207	22	4.83	LRR
	TLR11_MOUSE	208	247	40	0.02	LRR
	TLR11_MOUSE	248	271	24	0	LRR
	TLR11_MOUSE	272	294	23	0.2	LRR
	TLR11_MOUSE	295	317	23	0.13	LRR
	TLR11_MOUSE	318	342	25	0	LRR
	TLR11_MOUSE	343	366	24	0	LRR
	TLR11_MOUSE	367	391	25	0	LRR
	TLR11_MOUSE	392	415	24	0	LRR
	TLR11_MOUSE	416	439	24	0	LRR
	TLR11_MOUSE	440	463	24	0	LRR
	TLR11_MOUSE	464	487	24	0.15	LRR
	TLR11_MOUSE	488	508	21	0.1	LRR
	TLR11_MOUSE	509	532	24	1,958.65	LRR
	TLR11_MOUSE	533	556	24	3.13	LRR
	TLR11_MOUSE	557	584	28	0.12	LRR
	TLR11_MOUSE	585	607	23	25,540.36	LRR
	TLR11_MOUSE	608	636	29	0.03	LRR
	TLR11_MOUSE	637	660	24	0.03	LRR
	TLR11_MOUSE	661	685	25	1,199.99	LRR
	TLR11_MOUSE	686	709	24	75.82	Potential LRRCT
	TLR11_MOUSE					
	TLR2_MOUSE	84	107	24	0	LRR
	TLR2_MOUSE	108	131	24	0.01	LRR
	TLR2_MOUSE	132	155	24	0	LRR
	TLR2_MOUSE	156	180	25	0	LRR

Clade	GeneID	Start	Stop	Length	E-value	Feature
	TLR2_MOUSE	181	205	25	0.17	LRR
	TLR2_MOUSE	206	229	24	9.23	LRR
	TLR2_MOUSE	230	253	24	322.31	LRR
	TLR2_MOUSE	254	280	27	0.03	LRR
	TLR2_MOUSE	281	308	28	36.77	LRR
	TLR2_MOUSE	309	338	30	49.64	LRR
	TLR2_MOUSE	339	367	29	15.21	LRR
	TLR2_MOUSE	368	391	24	70.29	LRR
	TLR2_MOUSE	392	418	27	0	LRR
	TLR2_MOUSE	419	444	26	0	LRR
	TLR2_MOUSE	445	467	23	0	LRR
	TLR2_MOUSE	468	488	21	0.02	LRR
	TLR2_MOUSE	489	508	20	0	LRR
	TLR2_MOUSE	509	530	22	0	LRR
	TLR2_MOUSE	531	554	24	7.04	LRR
	TLR2_MOUSE	555	554	0	0	Potential LRRCT
	TLR2_MOUSE					
	TLR2_HUMAN	83	106	24	0	LRR
	TLR2_HUMAN	107	130	24	0	LRR
	TLR2_HUMAN	131	154	24	0	LRR
	TLR2_HUMAN	155	179	25	0	LRR
	TLR2_HUMAN	180	204	25	0.73	LRR
	TLR2_HUMAN	205	228	24	0.54	LRR
	TLR2_HUMAN	229	252	24	111.06	LRR
	TLR2_HUMAN	253	279	27	0.59	LRR
	TLR2_HUMAN	280	307	28	8.58	LRR
	TLR2_HUMAN	308	337	30	2.85	LRR
	TLR2_HUMAN	338	366	29	0.72	LRR
	TLR2_HUMAN	367	390	24	101.89	LRR
	TLR2_HUMAN	391	417	27	0	LRR
	TLR2_HUMAN	418	443	26	0	LRR
	TLR2_HUMAN	444	466	23	0.01	LRR

Clade	GeneID	Start	Stop	Length	E-value	Feature
	TLR2_HUMAN	467	487	21	0	LRR
	TLR2_HUMAN	488	507	20	0	LRR
	TLR2_HUMAN	508	529	22	0	LRR
	TLR2_HUMAN	530	553	24	0	LRR
	TLR2_HUMAN	554	577	24	0	Potential LRRCT
	TLR2_HUMAN					
	TLR3_MOUSE	83	106	24	0	LRR
	TLR3_MOUSE	107	130	24	0	LRR
	TLR3_MOUSE	131	154	24	0	LRR
	TLR3_MOUSE	155	178	24	0	LRR
	TLR3_MOUSE	179	202	24	0	LRR
	TLR3_MOUSE	203	228	26	0	LRR
	TLR3_MOUSE	229	252	24	0	LRR
	TLR3_MOUSE	253	279	27	6.46	LRR
	TLR3_MOUSE	280	305	26	0.01	LRR
	TLR3_MOUSE	306	329	24	0	LRR
	TLR3_MOUSE	330	353	24	0	LRR
	TLR3_MOUSE	354	386	33	0.25	LRR
	TLR3_MOUSE	387	410	24	0	LRR
	TLR3_MOUSE	411	438	28	0.03	LRR
	TLR3_MOUSE	439	462	24	0	LRR
	TLR3_MOUSE	463	487	25	0	LRR
	TLR3_MOUSE	488	511	24	0.09	LRR
	TLR3_MOUSE	512	537	26	0.03	LRR
	TLR3_MOUSE	538	561	24	0	LRR
	TLR3_MOUSE	562	593	32	0	LRR
	TLR3_MOUSE	594	617	24	0	LRR
	TLR3_MOUSE	618	641	24	0.04	LRR
	TLR3_MOUSE	642	666	25	0	LRR
	TLR3_MOUSE	667	690	24	0	Potential LRRCT
	TLR3_MOUSE					
	TLR3_HUMAN	82	105	24	0	LRR

Clade	GeneID	Start	Stop	Length	E-value	Feature
	TLR3_HUMAN	106	129	24	0	LRR
	TLR3_HUMAN	130	153	24	0	LRR
	TLR3_HUMAN	154	177	24	0	LRR
	TLR3_HUMAN	178	201	24	0	LRR
	TLR3_HUMAN	202	227	26	0	LRR
	TLR3_HUMAN	228	251	24	0	LRR
	TLR3_HUMAN	252	278	27	6.58	LRR
	TLR3_HUMAN	279	304	26	0.05	LRR
	TLR3_HUMAN	305	328	24	0	LRR
	TLR3_HUMAN	329	352	24	0	LRR
	TLR3_HUMAN	353	385	33	0.64	LRR
	TLR3_HUMAN	386	409	24	0	LRR
	TLR3_HUMAN	410	437	28	0.02	LRR
	TLR3_HUMAN	438	461	24	0	LRR
	TLR3_HUMAN	462	486	25	0	LRR
	TLR3_HUMAN	487	510	24	0.05	LRR
	TLR3_HUMAN	511	536	26	0.02	LRR
	TLR3_HUMAN	537	560	24	0	LRR
	TLR3_HUMAN	561	592	32	0	LRR
	TLR3_HUMAN	593	616	24	0.01	LRR
	TLR3_HUMAN	617	640	24	0	LRR
	TLR3_HUMAN	641	665	25	0	LRR
	TLR3_HUMAN	666	689	24	0	Potential LRRCT
	TLR3_HUMAN					
	TLR4_HUMAN	85	108	24	0	LRR
	TLR4_HUMAN	109	132	24	0	LRR
	TLR4_HUMAN	133	156	24	0	LRR
	TLR4_HUMAN	157	180	24	2.57	LRR
	TLR4_HUMAN	181	205	25	0	LRR
	TLR4_HUMAN	206	233	28	0	LRR
	TLR4_HUMAN	234	256	23	0.24	LRR
	TLR4_HUMAN	257	283	27	0.01	LRR

Clade	GeneID	Start	Stop	Length	E-value	Feature
	TLR4_HUMAN	284	313	30	65.56	LRR
	TLR4_HUMAN	314	338	25	41.94	LRR
	TLR4_HUMAN	339	360	22	510.91	LRR
	TLR4_HUMAN	361	381	21	0.74	LRR
	TLR4_HUMAN	382	403	22	0.03	LRR
	TLR4_HUMAN	404	429	26	0	LRR
	TLR4_HUMAN	430	452	23	0	LRR
	TLR4_HUMAN	453	477	25	0.01	LRR
	TLR4_HUMAN	478	501	24	0.01	LRR
	TLR4_HUMAN	502	526	25	0	LRR
	TLR4_HUMAN	527	550	24	0	LRR
	TLR4_HUMAN	551	574	24	0	LRR
	TLR4_HUMAN	575	599	25	0	LRR
	TLR4_HUMAN	600	599	0	0.01	Potential LRRCT
	TLR4_HUMAN					
	TLR5_MOUSE	79	102	24	0.01	LRR
	TLR5_MOUSE	103	127	25	45.92	LRR
	TLR5_MOUSE	128	151	24	0	LRR
	TLR5_MOUSE	152	177	26	2.14	LRR
	TLR5_MOUSE	178	202	25	0	LRR
	TLR5_MOUSE	203	228	26	0.29	LRR
	TLR5_MOUSE	229	258	30	139.74	LRR
	TLR5_MOUSE	259	285	27	0	LRR
	TLR5_MOUSE	286	320	35	9.97	LRR
	TLR5_MOUSE	321	344	24	0.1	LRR
	TLR5_MOUSE	345	368	24	0	LRR
	TLR5_MOUSE	369	392	24	0	LRR
	TLR5_MOUSE	393	416	24	0.01	LRR
	TLR5_MOUSE	417	435	19	0	LRR
	TLR5_MOUSE	436	455	20	0.1	LRR
	TLR5_MOUSE	456	480	25	0.05	LRR
	TLR5_MOUSE	481	505	25	0	LRR

Clade	GeneID	Start	Stop	Length	E-value	Feature
	TLR5_MOUSE	506	534	29	0.01	LRR
	TLR5_MOUSE	535	558	24	0	LRR
	TLR5_MOUSE	559	580	22	0	LRR
	TLR5_MOUSE	581	601	21	0	LRR
	TLR5_MOUSE	602	625	24	0	Potential LRRCT
	TLR5_MOUSE					
	TLR5_HUMAN	77	100	24	0.01	LRR
	TLR5_HUMAN	101	125	25	2.75	LRR
	TLR5_HUMAN	126	149	24	0	LRR
	TLR5_HUMAN	150	175	26	0.44	LRR
	TLR5_HUMAN	176	200	25	0	LRR
	TLR5_HUMAN	201	226	26	0	LRR
	TLR5_HUMAN	227	256	30	0.36	LRR
	TLR5_HUMAN	257	283	27	0	LRR
	TLR5_HUMAN	284	318	35	306.76	LRR
	TLR5_HUMAN	319	342	24	0.03	LRR
	TLR5_HUMAN	343	366	24	0	LRR
	TLR5_HUMAN	367	390	24	0	LRR
	TLR5_HUMAN	391	414	24	0.02	LRR
	TLR5_HUMAN	415	433	19	0	LRR
	TLR5_HUMAN	434	453	20	0.72	LRR
	TLR5_HUMAN	454	478	25	1.18	LRR
	TLR5_HUMAN	479	503	25	0	LRR
	TLR5_HUMAN	504	532	29	0	LRR
	TLR5_HUMAN	533	556	24	0	LRR
	TLR5_HUMAN	557	578	22	0	LRR
	TLR5_HUMAN	579	599	21	0	LRR
	TLR5_HUMAN	600	623	24	0	Potential LRRCT
	TLR5_HUMAN					
	TLR6_MOUSE	84	107	24	0	LRR
	TLR6_MOUSE	108	131	24	0	LRR
	TLR6_MOUSE	132	152	21	0	LRR

Clade	GeneID	Start	Stop	Length	E-value	Feature
	TLR6_MOUSE	153	177	25	0	LRR
	TLR6_MOUSE	178	200	23	0.04	LRR
	TLR6_MOUSE	201	227	27	744.85	LRR
	TLR6_MOUSE	228	249	22	86.04	LRR
	TLR6_MOUSE	250	280	31	0.01	LRR
	TLR6_MOUSE	281	307	27	213.57	LRR
	TLR6_MOUSE	308	333	26	0.37	LRR
	TLR6_MOUSE	334	362	29	0.91	LRR
	TLR6_MOUSE	363	384	22	0.18	LRR
	TLR6_MOUSE	385	408	24	1.74	LRR
	TLR6_MOUSE	409	434	26	0	LRR
	TLR6_MOUSE	435	459	25	0	LRR
	TLR6_MOUSE	460	481	22	0	LRR
	TLR6_MOUSE	482	504	23	0	LRR
	TLR6_MOUSE	505	526	22	0	LRR
	TLR6_MOUSE	527	550	24	0.03	LRR
	TLR6_MOUSE	551	574	24	0.04	Potential LRRCT
	TLR6_MOUSE					
	TLR7_MOUSE	94	117	24	0.1	LRR
	TLR7_MOUSE	118	155	38	0.01	Potential LRRCT
	TLR7_MOUSE	156	176	21	0	LRR
	TLR7_MOUSE	177	200	24	0.01	LRR
	TLR7_MOUSE	201	232	32	0	LRR
	TLR7_MOUSE	233	253	21	0	LRR
	TLR7_MOUSE	254	277	24	0.01	LRR
	TLR7_MOUSE	278	318	41	0	LRR
	TLR7_MOUSE	319	342	24	0	LRR
	TLR7_MOUSE	343	368	26	0	LRR
	TLR7_MOUSE	369	398	30	0	LRR
	TLR7_MOUSE	399	425	27	0.88	LRR
	TLR7_MOUSE	426	449	24	0	LRR
	TLR7_MOUSE	450	524	75	0	LRR

Clade	GeneID	Start	Stop	Length	E-value	Feature
	TLR7_MOUSE	523	546	24	0	LRR
	TLR7_MOUSE	547	571	25	0	LRR
	TLR7_MOUSE	572	595	24	0	LRR
	TLR7_MOUSE	596	625	30	0	LRR
	TLR7_MOUSE	626	648	23	0.04	LRR
	TLR7_MOUSE	649	679	31	0	LRR
	TLR7_MOUSE	680	704	25	0	LRR
	TLR7_MOUSE	705	728	24	0	LRR
	TLR7_MOUSE	729	752	24	0	LRR
	TLR7_MOUSE	753	776	24	0	LRR
	TLR7_MOUSE	777	802	26	0	LRR
	TLR7_MOUSE	803	826	24	0	Potential LRRCT
	TLR7_MOUSE					
	TLR7_HUMAN	99	122	24	0.08	LRR
	TLR7_HUMAN	123	160	38	0.16	Potential LRRCT
	TLR7_HUMAN	161	181	21	0	LRR
	TLR7_HUMAN	182	205	24	0	LRR
	TLR7_HUMAN	206	237	32	0	LRR
	TLR7_HUMAN	238	258	21	0	LRR
	TLR7_HUMAN	259	282	24	0	LRR
	TLR7_HUMAN	283	323	41	0	LRR
	TLR7_HUMAN	324	347	24	0	LRR
	TLR7_HUMAN	348	373	26	0	LRR
	TLR7_HUMAN	374	403	30	0	LRR
	TLR7_HUMAN	404	430	27	0.14	LRR
	TLR7_HUMAN	431	454	24	0	LRR
	TLR7_HUMAN	455	529	75	0	LRR
	TLR7_HUMAN	527	550	24	0	LRR
	TLR7_HUMAN	551	575	25	0	LRR
	TLR7_HUMAN	576	599	24	0	LRR
	TLR7_HUMAN	600	629	30	0	LRR
	TLR7_HUMAN	630	652	23	0.01	LRR

Clade	GeneID	Start	Stop	Length	E-value	Feature
	TLR7_HUMAN	653	683	31	0	LRR
	TLR7_HUMAN	684	708	25	0	LRR
	TLR7_HUMAN	709	732	24	0	LRR
	TLR7_HUMAN	733	756	24	0	LRR
	TLR7_HUMAN	757	780	24	0	LRR
	TLR7_HUMAN	781	806	26	0	LRR
	TLR7_HUMAN	807	830	24	0	Potential LRRCT
	TLR7_HUMAN					
	TLR4_MOUSE	85	108	24	0	LRR
	TLR4_MOUSE	109	132	24	0	LRR
	TLR4_MOUSE	133	156	24	0	LRR
	TLR4_MOUSE	157	180	24	2.28	LRR
	TLR4_MOUSE	181	205	25	0	LRR
	TLR4_MOUSE	206	233	28	0	LRR
	TLR4_MOUSE	234	256	23	0.18	LRR
	TLR4_MOUSE	257	283	27	0	LRR
	TLR4_MOUSE	284	313	30	35.44	LRR
	TLR4_MOUSE	314	337	24	249.38	LRR
	TLR4_MOUSE	338	359	22	16.74	LRR
	TLR4_MOUSE	360	380	21	9.39	LRR
	TLR4_MOUSE	381	402	22	0.01	LRR
	TLR4_MOUSE	403	428	26	0	LRR
	TLR4_MOUSE	429	451	23	0	LRR
	TLR4_MOUSE	452	476	25	0.02	LRR
	TLR4_MOUSE	477	500	24	0.03	LRR
	TLR4_MOUSE	501	525	25	0.01	LRR
	TLR4_MOUSE	526	549	24	0	LRR
	TLR4_MOUSE	550	573	24	0	LRR
	TLR4_MOUSE	574	597	24	0	LRR
	TLR4_MOUSE	598	621	24	0.39	Potential LRRCT
	TLR4_MOUSE					
	TLR6_HUMAN	85	108	24	0	LRR

Clade	GeneID	Start	Stop	Length	E-value	Feature
	TLR6_HUMAN	109	132	24	0	LRR
	TLR6_HUMAN	133	153	21	0	LRR
	TLR6_HUMAN	154	178	25	0	LRR
	TLR6_HUMAN	179	201	23	0.1	LRR
	TLR6_HUMAN	202	226	25	247.99	LRR
	TLR6_HUMAN	227	250	24	35.72	LRR
	TLR6_HUMAN	251	281	31	0.81	LRR
	TLR6_HUMAN	282	308	27	675.85	LRR
	TLR6_HUMAN	309	334	26	1.26	LRR
	TLR6_HUMAN	335	363	29	5.56	LRR
	TLR6_HUMAN	364	385	22	3.56	LRR
	TLR6_HUMAN	386	409	24	1.21	LRR
	TLR6_HUMAN	410	435	26	0	LRR
	TLR6_HUMAN	436	460	25	0	LRR
	TLR6_HUMAN	461	482	22	0	LRR
	TLR6_HUMAN	483	505	23	0	LRR
	TLR6_HUMAN	506	527	22	0	LRR
	TLR6_HUMAN	528	551	24	0.02	LRR
	TLR6_HUMAN	552	575	24	2.64	Potential LRRCT
	TLR6_HUMAN					
	TLR8_MOUSE	92	115	24	0	LRR
	TLR8_MOUSE	116	149	34	0.09	LRR
	TLR8_MOUSE	150	170	21	0	LRR
	TLR8_MOUSE	171	194	24	0	LRR
	TLR8_MOUSE	195	224	30	0	LRR
	TLR8_MOUSE	225	245	21	0	LRR
	TLR8_MOUSE	246	269	24	0	LRR
	TLR8_MOUSE	270	310	41	0	LRR
	TLR8_MOUSE	311	334	24	0.02	LRR
	TLR8_MOUSE	335	360	26	0	LRR
	TLR8_MOUSE	361	390	30	0	LRR
	TLR8_MOUSE	391	417	27	0.04	LRR

Clade	GeneID	Start	Stop	Length	E-value	Feature
	TLR8_MOUSE	418	441	24	0.08	LRR
	TLR8_MOUSE	442	500	59	0	LRR
	TLR8_MOUSE	501	524	24	0	LRR
	TLR8_MOUSE	525	549	25	0.21	LRR
	TLR8_MOUSE	550	573	24	0	LRR
	TLR8_MOUSE	574	603	30	0	LRR
	TLR8_MOUSE	604	627	24	0	LRR
	TLR8_MOUSE	628	658	31	0	LRR
	TLR8_MOUSE	659	683	25	0	LRR
	TLR8_MOUSE	684	707	24	0	LRR
	TLR8_MOUSE	708	731	24	0	LRR
	TLR8_MOUSE	732	755	24	0	LRR
	TLR8_MOUSE	756	781	26	0	LRR
	TLR8_MOUSE	782	805	24	0	Potential LRRCT
	TLR8_MOUSE					
	TLR8_HUMAN	96	119	24	0	LRR
	TLR8_HUMAN	120	157	38	0.08	LRR
	TLR8_HUMAN	158	178	21	0	LRR
	TLR8_HUMAN	179	202	24	0	LRR
	TLR8_HUMAN	203	233	31	0	LRR
	TLR8_HUMAN	234	254	21	0	LRR
	TLR8_HUMAN	255	278	24	0	LRR
	TLR8_HUMAN	279	319	41	0	LRR
	TLR8_HUMAN	320	343	24	0	LRR
	TLR8_HUMAN	344	369	26	0	LRR
	TLR8_HUMAN	370	399	30	0	LRR
	TLR8_HUMAN	400	426	27	0.06	LRR
	TLR8_HUMAN	427	450	24	0.06	LRR
	TLR8_HUMAN	451	513	63	0	LRR
	TLR8_HUMAN	514	537	24	0	LRR
	TLR8_HUMAN	538	562	25	0.26	LRR
	TLR8_HUMAN	563	586	24	0	LRR

Clade	GeneID	Start	Stop	Length	E-value	Feature
	TLR8_HUMAN	587	616	30	0	LRR
	TLR8_HUMAN	617	640	24	0	LRR
	TLR8_HUMAN	641	671	31	0	LRR
	TLR8_HUMAN	672	696	25	0	LRR
	TLR8_HUMAN	697	720	24	0.01	LRR
	TLR8_HUMAN	721	744	24	0	LRR
	TLR8_HUMAN	745	768	24	0	LRR
	TLR8_HUMAN	769	794	26	0	LRR
	TLR8_HUMAN	795	818	24	0	Potential LRRCT
	TLR8_HUMAN					
	TLR9_MOUSE	95	118	24	0	LRR
	TLR9_MOUSE	119	154	36	0.01	LRR
	TLR9_MOUSE	155	174	20	0	LRR
	TLR9_MOUSE	175	198	24	0	LRR
	TLR9_MOUSE	199	230	32	0	LRR
	TLR9_MOUSE	231	251	21	0	LRR
	TLR9_MOUSE	252	275	24	0	LRR
	TLR9_MOUSE	276	315	40	0	LRR
	TLR9_MOUSE	316	339	24	0.37	LRR
	TLR9_MOUSE	340	365	26	0	LRR
	TLR9_MOUSE	366	395	30	0	LRR
	TLR9_MOUSE	396	422	27	0.02	LRR
	TLR9_MOUSE	423	446	24	0	LRR
	TLR9_MOUSE	447	504	58	0	LRR
	TLR9_MOUSE	505	528	24	0.73	LRR
	TLR9_MOUSE	529	553	25	0	LRR
	TLR9_MOUSE	554	577	24	0	LRR
	TLR9_MOUSE	578	607	30	0	LRR
	TLR9_MOUSE	608	630	23	0	LRR
	TLR9_MOUSE	631	660	30	0	LRR
	TLR9_MOUSE	661	685	25	0	LRR
	TLR9_MOUSE	686	709	24	0	LRR

Clade	GeneID	Start	Stop	Length	E-value	Feature
	TLR9_MOUSE	710	733	24	0	LRR
	TLR9_MOUSE	734	757	24	0	LRR
	TLR9_MOUSE	758	782	25	0	LRR
	TLR9_MOUSE	783	806	24	0	Potential LRRCT
	TLR9_MOUSE					
	TLR9_HUMAN	96	119	24	0	LRR
	TLR9_HUMAN	120	155	36	0.01	LRR
	TLR9_HUMAN	156	175	20	0	LRR
	TLR9_HUMAN	176	199	24	0	LRR
	TLR9_HUMAN	200	231	32	0.02	LRR
	TLR9_HUMAN	232	252	21	0	LRR
	TLR9_HUMAN	253	276	24	0	LRR
	TLR9_HUMAN	277	316	40	0	LRR
	TLR9_HUMAN	317	340	24	0.22	LRR
	TLR9_HUMAN	341	366	26	0	LRR
	TLR9_HUMAN	367	396	30	0	LRR
	TLR9_HUMAN	397	423	27	0	LRR
	TLR9_HUMAN	424	447	24	0	LRR
	TLR9_HUMAN	448	507	60	0	LRR
	TLR9_HUMAN	505	528	24	0.01	LRR
	TLR9_HUMAN	529	553	25	0	LRR
	TLR9_HUMAN	554	577	24	0	LRR
	TLR9_HUMAN	578	607	30	0	LRR
	TLR9_HUMAN	608	630	23	0	LRR
	TLR9_HUMAN	631	660	30	0	LRR
	TLR9_HUMAN	661	685	25	0	LRR
	TLR9_HUMAN	686	709	24	0	LRR
	TLR9_HUMAN	710	733	24	0	LRR
	TLR9_HUMAN	734	757	24	0	LRR
	TLR9_HUMAN	758	782	25	0	LRR
	TLR9_HUMAN	783	806	24	0	Potential LRRCT
	TLR9_HUMAN					

Clade	GeneID	Start	Stop	Length	E-value	Feature
	Toll-9	164	270	107	0	LRR
	Toll-9	271	294	24	0	LRR
	Toll-9	295	318	24	0	LRR
	Toll-9	319	363	45	0	LRR
	Toll-9	364	388	25	0	LRR
	Toll-9	389	411	23	0.07	InLRR
	Toll-9	412	438	27	0	LRR
	Toll-9	439	460	22	0.05	InLRR
	Toll-9	461	484	24	0	LRR
	Toll-9	485	508	24	0	LRR
	Toll-9	509	532	24	0	LRR
	Toll-9	533	908	376	0	Potential LRRCT
	Toll-9					

[END OF DOCUMENT]
